# Metabolomic Assessment Reveals Alteration in Polyols and Branched Chain Amino Acids Associated With Present and Future Renal Impairment in a Discovery Cohort of 637 Persons With Type 1 Diabetes

**DOI:** 10.3389/fendo.2019.00818

**Published:** 2019-11-22

**Authors:** Nete Tofte, Tommi Suvitaival, Kajetan Trost, Ismo Matias Mattila, Simone Theilade, Signe Abitz Winther, Tarunveer Singh Ahluwalia, Marie Frimodt-Møller, Cristina Legido-Quigley, Peter Rossing

**Affiliations:** ^1^Steno Diabetes Center Copenhagen, Gentofte, Denmark; ^2^Novo Nordisk A/S, Måløv, Denmark; ^3^Institute of Pharmaceutical Science, King's College London, London, United Kingdom; ^4^Department of Clinical Medicine, University of Copenhagen, Copenhagen, Denmark

**Keywords:** diabetic kidney disease, type 1 diabetes, metabolomics, end-stage renal disease, amino acids, polyols

## Abstract

**Background:** Improved understanding of the pathophysiology causing diabetic kidney disease (DKD) is imperative. The aim of this study was to uncover associations between serum metabolites and renal outcomes.

**Methods:** Non-targeted serum metabolomics analyses were performed in samples from 637 persons with type 1 diabetes using two-dimensional gas chromatography coupled to time-of-flight mass-spectrometry. Longitudinal data at follow-up (median 5.5 years) on renal events were obtained from national Danish health registries. A composite renal endpoint (*n* = 123) consisted of estimated glomerular filtration rate (eGFR) decline from baseline (≥30%), progression to end-stage renal disease and all-cause mortality. Metabolites with significant associations (*p* < 0.05) in any of the cross-sectional analyses with eGFR and albuminuria were analyzed for specific and composite endpoints. Adjustments included traditional cardiovascular risk factors and correction for multiple testing.

**Results:** A data-driven partial correlation analysis revealed a dense fabric of co-regulated metabolites and clinical variables dominated by eGFR. Ribonic acid and myo-inositol were inversely associated with eGFR, positively associated with macroalbuminuria (*p* < 0.02) and longitudinally associated with higher risk of eGFR decline ≥30% (HR 2.2–2.7, CI [1.3–4.3], *p* < 0.001). Ribonic acid was associated with a combined renal endpoint (HR 1.8, CI [1.3–2.3], *p* = 0.001). The hydroxy butyrate 3,4-dihydroxybutanoic acid was cross-sectionally associated with micro- and macroalbuminuria, urinary albumin excretion rate and inversely associated with eGFR (*p* < 0.04) while branched chain amino acids were associated with eGFR and lower risk of the combined renal endpoint (*p* < 0.02).

**Conclusions:** Alterations in serum metabolites, particularly polyols and amino acids, were associated with renal endpoints in type 1 diabetes highlighting molecular pathways associated with progression of kidney disease. External validation is needed to further assess their roles and potentials as future therapeutic targets.

## Introduction

With the worldwide increase in diabetes prevalence, there is also an increasing prevalence of diabetic kidney disease (DKD). Although control of classical cardiovascular risk factors delays disease progression, DKD remains the leading cause of end stage renal disease (ESRD) in the western world and is associated with a substantially higher risk of cardiovascular disease and mortality (1). This may partly be explained by late initiation of therapy and limited specific treatment options. DKD is often asymptomatic and the classical biomarkers estimated glomerular filtration rate (eGFR) and urinary albumin to creatinine ratio (UACR) used to diagnose and monitor disease, may not be altered until the later stages of the disease. Kidney biopsies which are invasive and require special physician skills and resources are rarely performed in individuals with diabetes. Omics-based technologies offer opportunities for an extensive characterization of metabolic traits associated with DKD. A better understanding of the underlying pathophysiology may lead to discovery of new therapeutic targets. Research on metabolomics in kidney disease is of particular interest as metabolite levels are influenced in various ways by the kidney function. Moreover, it has previously been demonstrated that different metabolite patterns are associated with different causes of chronic kidney disease (2). Several previous studies of untargeted metabolomics in DKD have been performed in individuals with type 2 diabetes (3–7). In a study by Haukka et al. in persons with type 1 diabetes, several uremic toxins, and carnitine metabolites were associated with progression from normo- to microalbuminuria (8) while a study from Joslin Diabetes Center revealed nine modified metabolites that were associated with eGFR slope and progression to ESRD (9), also in individuals with type 1 diabetes. In the present study, cross-sectional associations between metabolites in serum and measures of renal impairment in Danish individuals with type 1 diabetes were examined. Further, metabolites identified in cross-sectional analyses were tested for association to longitudinal outcomes including eGFR and albuminuria slopes, ESRD and all-cause mortality.

## Materials and Methods

### Study Inclusion

A study population consisting of 676 persons with type 1 diabetes from Steno Diabetes Center Copenhagen was included over 2 years from 2009 in a cross-sectional study, also including a biobank for future research. The details of the selection process have previously been described (10). Participants were classified as having normoalbuminuria if UAER was <30 mg/24 h or mg/g, microalbuminuria if UAER was or formerly had been recorded between 30 and 299 mg/24 h or mg/g, and macroalbuminuria if UAER was or formerly had been recorded ≥300 mg/24 h or mg/g in at least two of three consecutive measurements. Individuals classified as having normoalbuminuria did not have a history of former micro- or macroalbuminuria. Individuals with prior or present ESRD, defined as dialysis, renal transplantation or a glomerular filtration rate (GFR)/eGFR <15 mL/min/1.73 m^2^ were excluded. For the current metabolomics study, analyses were performed in the year 2018 on serum (at the research biobank) from 637 (94%) individuals. The study was performed in accordance with the Declaration of Helsinki. The Ethics Committee E, Region Hovedstaden, Denmark, approved the original and follow-up research protocol. Written informed consent was obtained from all participants for the original study and the biobank.

### Biochemical and Other Measures at Baseline

Hemoglobin A_1c_ (HbA_1c_), plasma (p)-cholesterol and p-triglyceride levels were measured by standardized methods in the routine laboratory at Steno Diabetes Center Copenhagen, serum creatinine was determined by an enzymatic reaction (IDMS). UAER was measured in three consecutive 24 h urine collections by enzyme immunoassay. The eGFR was calculated from serum creatinine using the Chronic Kidney Disease Epidemiology Collaboration (CKD-EPI) equation (11). Brachial blood pressure was measured in the sitting position after at least 10 min rest with an automatic oscillometric device and an appropriately sized cuff. Body mass index (BMI) was calculated as weight in kilograms divided by height in meters squared. Based on standardized questionnaires, current users of >1 cigarettes, cigars or pipes per day were classified as smokers and all others were classified as non-smokers. Information on medication was collected from electronic medical records at study baseline.

### Metabolomics Analyses

The serum samples were immediately stored at −80°C until analysis. For the analysis, a Leco Pegasus 4D GC × GC-TOFMS instrument (Leco Corp., MI, USA) was used. The method has previously been described in detail (12). The GC × GC-TOFMS data were processed (i.e., alignment and normalization) using Guineu (13).With the present metabolomics platform 75 metabolites were identified and included in data analyses. These metabolites included amino acids, free fatty acids, compounds from the energy metabolism pathways and polyols. Peak areas were normalized to spiked internal standards (glutamic acid-d5, heptadecanoic acid-d33, succinic acid-d4, uric acid-2N15, and valine-d8), and median-corrected for between batch variation in R. Metabolites with equal to or <20% missing/undetectable values were included and all missing values were imputed with the k-nearest neighbor algorithm and log2-transformed (14). The proportion of imputed values for each metabolite ranged from 0.1 to 7%. Per metabolite information on missing values is given in the Supplementary Table S1.

### Longitudinal Endpoints

All data regarding hospital admission and related ICD-10 diagnoses (www.who.int/classifications/icd/en/), procedural codes [according to the Nordic Classification of Surgical Procedures (NCSP); www.sst.dk] and date of death were obtained from the Danish National Health Register until December 31st 2016 (15, 16). Information concerning causes of death was available from the Danish National Death Register until December 31st 2015. Biochemical measurements (p-creatinine and UACR) were obtained from electronic laboratory records. We did not have access to information concerning changes in medication during follow-up.

Albuminuria (logUACR) slope was calculated based on all the available measurements from outpatient visits during follow-up, in participants with at least two measurements and a minimum follow-up of 3 years (*n* = 485). Decline in eGFR was assessed as time to the first occurrence of ≥30% decrease from baseline, as proposed by Coresh et al. (17) and as eGFR slope. An endpoint of ≥40% decrease in eGFR from baseline was computed for a sensitivity analysis. ESRD was defined as CKD stage 5 (ICD-10 code N18.5), chronic dialysis (procedural code BJFD2), kidney transplantation (procedural code KKAS 00, 10 and 20) or eGFR <15 ml/min/1.73 m^2^. A combined renal endpoint included ≥30% decline in eGFR, ESRD and all-cause mortality, with time to first event. A similar combined renal endpoint is often used in clinical outcome trials, although here modified in terms of eGFR decline since this is an observational study with no initial decline in eGFR caused by intervention.

### Statistical Analyses

Tests of clinical variables were done with SAS Enterprise Guide version 7.11. Continuous variables were reported as mean ± standard deviation (SD) for normally distributed data. Skewed data were reported as median (interquartile range, IQR) and were log2-transformed for analyses. Categorical variables were presented as total numbers with corresponding percentages. Comparisons of continuous and categorical variables between groups were performed using the analysis of variance (ANOVA) and *X*^2^-test, respectively.

All data analyses with metabolite data were done in R-3.4.2. Partial correlation network of metabolites and clinical variables was computed and visualized with R-package qgraph using the graphical LASSO algorithm and extended Bayesian information criterion to select the model complexity. Data were imputed and auto-scaled prior to model-fitting.

Cross-sectional associations between single metabolites and eGFR, logUAER or albuminuria groups were assessed with linear regression models adjusted to clinical variables using R-package limma. *P*-values for each analysis were corrected for multiple testing using the Benjamini–Hochberg method (*p*_BH_) (18, 19). Significant associations between clinical variables and metabolites were visualized as a bipartite network using R-package ggplot2.

Metabolites with *p*_BH_ < 0.05 in one or more of the adjusted cross-sectional models were further examined longitudinally using survival analysis with the Cox proportional hazards model using R-package survival. First, hazard ratios (HRs) with 95% CI on the log2-scale, were computed for the combined renal endpoint, both, in crude and adjusted models. Metabolites associating with the combined renal endpoint in the crude model (*p*_BH_ < 0.05) were included for testing of three specific endpoints: ≥30% decline in eGFR, ESRD and all-cause mortality. HRs of the metabolites were visualized in forest plots grouped by the dependent variable using R-package ggplot2. In the longitudinal analyses correction for multiple testing was based on the number of included metabolites in each analysis. Metabolites associating with the specific endpoints in the adjusted Cox models were visualized with the respective Kaplan-Meier curves and outcome-specific violin plots.

In addition to cross-sectional and survival analyses, metabolites with an association in one or more of the adjusted cross-sectional models were tested with adjusted linear models for association to eGFR and albuminuria slopes.

Clinical baseline variables that were included in cross-sectional and longitudinal multivariate models were age, sex, HbA_1c_, systolic blood pressure, smoking, statin treatment, body mass index, p-triglycerides, total p-cholesterol, eGFR, and UAER, as these variables may affect both the metabolites levels as well as the endpoints of interest (20). As a sensitivity analysis, associations between renin-angiotensin-aldosterone system (RAAS)-inhibition and metabolites were tested (data not shown).

Sensitivity analyses with further adjustment for previous cardiovascular disease and retinopathy were performed for the metabolites that were significantly associated with the endpoints in longitudinal analyses. In addition, an endpoint of eGFR decline ≥40% from baseline was tested.

Sample-size calculation was done to assess the sample size needed for reproducing the reported associations to the combined renal endpoint. To do this, each metabolite was dichotomized to a binary value representing a level above or below median of the metabolite. The crude hazard ratio for combined renal endpoint was re-estimated using the dichotomized metabolite variables. Finally, the required sample size for estimating the respective hazard ratio at alpha level of 0.05, power of 0.8 and observed probability of event at 0.19, was calculated.

## Results

The study included 637 Caucasian individuals with type 1 diabetes with a mean age of 55 ± 13 years, a median diabetes duration of 35 [25–44] years and 45% women (Table 1). Overall, 47% had persistent normoalbuminuria at baseline, 25 and 29% had a history of or current microalbuminuria and macroalbuminuria, respectively. At baseline mean eGFR was 81 ± 26 ml min^−1^ 1.73 m^−2^ and median UAER was 18 [8–65] mg/24-h. During follow-up, 91 participants experienced a decline in eGFR ≥ 30%, 21 individuals developed ESRD, 123 had at least one event in the combined renal endpoint and 58 died (Supplementary Figure S1). Median follow-up time with available measurements was 5.2 [2.7–6.2] years for ≥30% decline in eGFR, 5.2 [4.8–5.7] years for progression to ESRD and 6.2 [5.8–6.7] years for all-cause mortality. The eGFR and UACR slopes were based on medians of 6 measurements in 485 participants and 16 measurements in 484 participants, respectively. The mean yearly change in eGFR was −1.4 ± 3.6 ml/min/year and the median yearly change in UACR was 3.5 [−13.0 to 8.7]%.

**Table 1:** Baseline characteristics according to albuminuria group.

	**All participants** **(n = 637)**	**Normoalbuminuria** **(n = 297)**	**Microalbuminuria** **(n = 158)**	**Macroalbuminuria** **(n = 182)**	**Normo- vs. micro- vs.** **macro-albuminuria** **(*p*)**
Female	287 (45)	147 (49)	61 (39)	79 (43)	0.074
Age, years	55 ± 13	53 ± 14	58 ± 12	55 ± 10	<0.001
Diabetes duration, years	35 [25–44]	30 [9–41]	36 [26–48]	39 [31–45]	<0.001
Body mass index, kg/m^2^	25 ± 6	25 ± 4	26 ± 4	26 ± 9	0.042
Systolic blood pressure, mmHg	132 ± 17	129 ± 16	133 ± 18	134 ± 19	0.002
Diastolic blood pressure, mmHg	74 ± 9	75 ± 9	73 ± 9	74 ± 10	0.021
HbA_1c_, % (mmol/mol)	8.0 ± 1.1 (64 ± 13)	7.8 ± 1.0 (62 ± 12)	8.1 ± 1.2 (65 ± 13)	8.4 ± 1.2 (68 ± 13)	<0.001
Total cholesterol, mmol/l	4.7 ± 0.9	4.7 ± 0.8	4.7 ± 0.9	4.6 ± 1.0	0.333
LDL cholesterol, mmol/l	2.5 ± 0.8	2.5 ± 0.7	2.5 ± 0.8	2.5 ± 0.9	0.592
HDL cholesterol, mmol/l	1.7 ± 0.5	1.8 ± 0.6	1.7 ± 0.5	1.6 ± 0.5	0.001
Triglycerides, mmol/l	1.0 [0.7–1.3]	0.9 [0.7–1.2]	0.9 [0.7–1.4]	1.1 [0.8–1.5]	<0.001
eGFR, ml min^−1^ 1.73 m^−2^	81 ± 26	92 ± 18	81 ± 23	63 ± 28	<0.001
*UAER, mg/24-h	18 [8–65]	8 [6–13]	33 [18–62]	136 [32–473]	<0.001
Retinopathy grade Nil Simplex Proliferative Blind	135 (21) 262 (41) 218 (34) 19 (3)	104 (35) 139 (47) 48 (16) 3 (1)	24 (15) 75 (47) 55 (35) 4 (3)	7 (4) 48 (26) 115 (63) 12 (7)	<0.001
Smokers	133 (21)	56 (19)	31 (20)	46 (25)	0.221
RAAS inhibition treatment	426 (67)	126 (43)	129 (82)	171 (94)	<0.001
Statin treatment	480 (60)	122 (41)	106 (67)	152 (84)	<0.001
Follow-up					
Decline in eGFR ≥ 30 %	91 (14)	10 (3)	19 (12)	62 (34)	<0.001
End stage renal disease	21 (3)	0	2 (1)	19 (10)	<0.001
All-cause mortality	58 (9)	12 (4)	21 (13)	25 (14)	<0.001
Combined renal endpoint	123 (19)	16 (5)	29 (18)	78 (43)	<0.001

Data are n (%, rounded), mean ± SD or median [IQR]. HbA_1c_ hemoglobin A_1c_, eGFR estimated glomerular filtration rate, UAER urinary albumin excretion rate, RAAS renin-angiotensin-aldosterone system. The combined renal endpoint consisted of ≥ 30% decrease in eGFR, ESRD and all-cause mortality. *Some individuals with previous persistent micro- or macroalbuminuria had lower values at baseline presumably due to reno-protective medical therapy.

### Network of Metabolic Regulation

**Figure 1:**
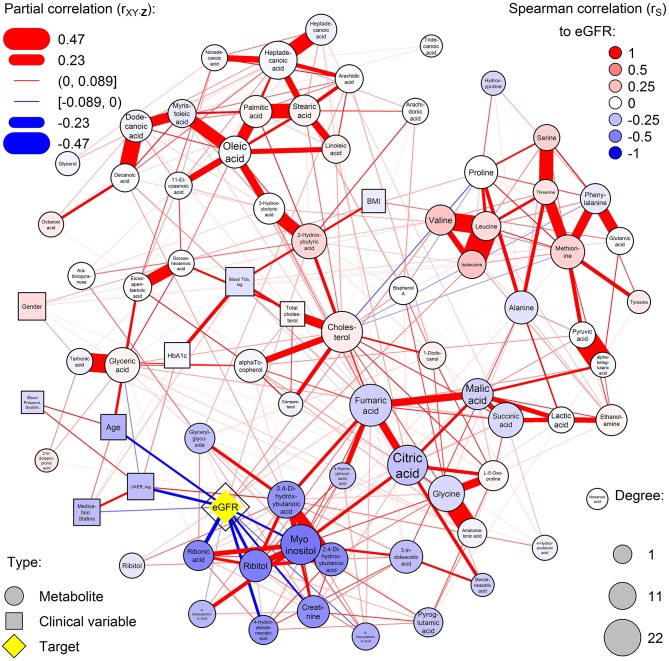
Partial correlation network of serum metabolites and clinical covariates. Nodes are measured variables and edges (lines) are inferred associations (width: strength; color: sign). Circular and rectangular nodes, respectively, are metabolites and clinical variables. Node size is the degree of the node (number of associations with the node). The highest-degree clinical node is highlighted in yellow and other nodes are colored by the respective Spearman correlation to the yellow node (red and blue for positive and inverse correlation, respectively). Blue color represents increased levels of the metabolites with deteriorating kidney function.

A dense co-regulation network of metabolites and clinical variables was computed (Figure 1). Metabolites partially correlated into four main clusters: free fatty acids (top-left), amino acids (top-right), glucose metabolism (bottom-right), and polyols (bottom-left). The highest number of associations between metabolites and clinical variables were with eGFR, which was associated with 11 metabolites and a central node. Particularly polyols ribonic acid, myo-inositol, and ribitol were inversely associated with eGFR but were independent of HbA_1c_. Also, the balance of hydroxybutyrates was disturbed, and amino acids valine and isoleucine were associated with eGFR.

### Cross-Sectional Associations Between Metabolites and Renal Function Measures

**Figure 2:**
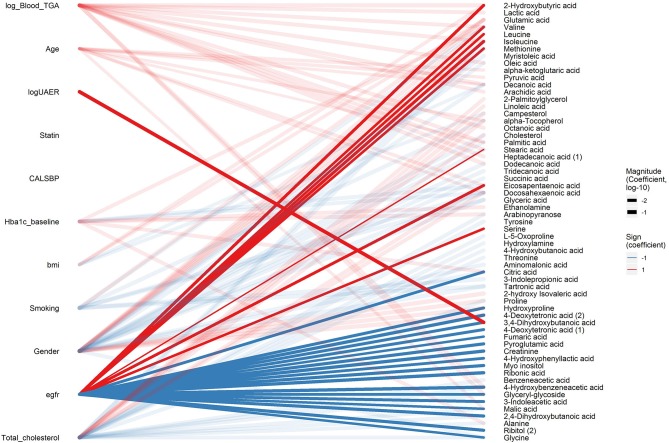
Cross-sectional associations between metabolites and clinical variables. Associations with p_BH_ < 0.05 between clinical variables (left) and metabolites (right) are shown in a bipartite network. Sign of the coefficient is shown in color (red and blue for positive and inverse association, respectively) and magnitude of the coefficient is shown in the width of the line. Associations related to eGFR and logUAER are highlighted with opaque lines while other associations are shown in transparent lines.

After the data-driven network analysis, we focused on detecting associations between metabolites and clinical variables. In cross-sectional analyses, 3,4-dihydroxybutanoicacid (3,4-DB) was positively associated with microalbuminuria and macroalbuminuria vs. normoalbuminuria, UAER and inversely associated with eGFR (*p*_BH_ < 0.04, Figure 2 and Supplementary Material, p. 13, 14, 25, and 56). Ribonic acid, myo-inositol, 2,4-dihydroxybutanoic (2,4-DB) acid and 4-hydroxybenzeneacetic acid were positively associated with macroalbuminuria vs. normoalbuminuria and inversely associated with eGFR (*p*_BH_ < 0.04). Additionally, 24 metabolites primarily from amino acids, carboxylic acids, and free fatty acids, were associated with eGFR (*p*_BH_ < 0.05). Although large part of the metabolomic associations were with eGFR, 33 other metabolites were associated with other clinical variables, such as HbA_1c_ (Figure 2).

### Metabolites Associated With eGFR or Albuminuria Slope

In longitudinal adjusted analyses of slopes, 4-hydroxybenzeneacetic acid (β = 0.68; *p*_BH_ = 0.004) and 4-deoxytetronic acid (β = 0.28; *p*_BH_ = 0.03) were positively associated with albuminuria slope (Supplementary Material, p. 243). Ribitol (β = −0.03; *p*_BH_ = 0.008) and octanoic acid (β = −0.02; *p*_BH_ = 0.008) were inversely associated with eGFR slope while succinic acid was positively associated with eGFR slope (β = 0.01; *p*_BH_ = 0.04; Supplementary Material, p. 247).

### Candidate Biomarkers for Risk of Longitudinal Renal Endpoints

**Figure 3:**
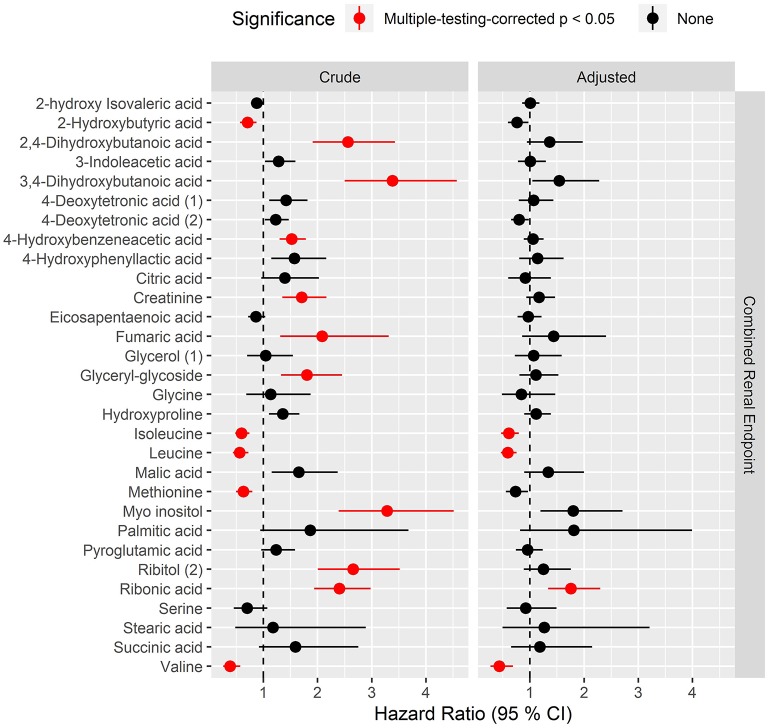
Hazard ratios of prioritized metabolites for progression to the combined renal endpoint. Hazard ratios (HRs) to the combined endpoint for metabolites that had *p*_BH_ < 0.05 in any of the cross-sectional analyses. The combined renal endpoint includes ≥30% decrease in eGFR, ESRD and all-cause mortality with time to first event. HRs are presented per 1 SD increase of the log2 metabolite and are shown for crude model (left) and for model adjusted for age, sex, HbA_1c_, systolic blood pressure, smoking, body mass index, statin treatment, p-triglycerides, total p-cholesterol, eGFR and logUAER (right).

Overall, higher levels of hydroxybutyrates and polyols were associated with higher risk while several amino acids were associated with lower risk of progression to the combined renal endpoint (*p*_BH_ <0.05) in crude models (Figure 3, left). After multivariate adjustment, ribonic acid remained significantly associated with higher risk and isoleucine, leucine and valine associated with lower risk of the combined renal endpoint (*p*_BH_ < 0.02, Figure 3, right).

**Figure 4:**
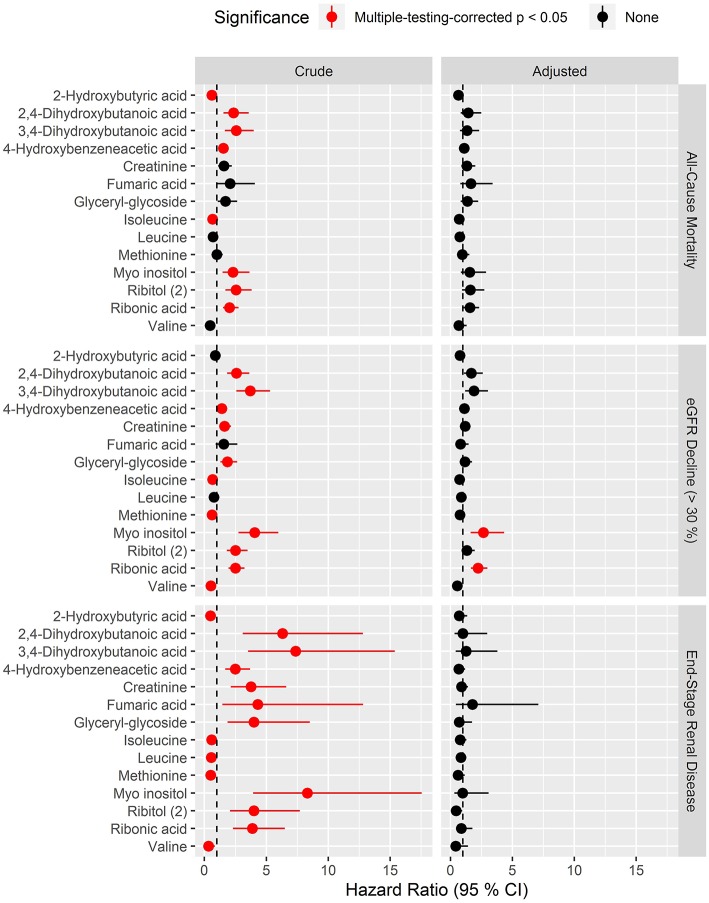
Hazard ratios of prioritized metabolites for progression to the three specific endpoints: all-cause mortality, eGFR decline (>30%) and end-stage renal disease. Hazard ratios (HRs) to the specific endpoints for metabolites that had pBH < 0.05 in crude survival models for the combined renal endpoint. Results for the three endpoints are shown in respective vertically arranged sub-figures. HRs are presented per 1 SD increase of the log2 metabolite and shown for crude model (left) and for model adjusted for age, sex, HbA_1c_, systolic blood pressure, smoking, body mass index, statin treatment, p-triglycerides, total p-cholesterol, eGFR, and logUAER (right).

**Figure 5:**
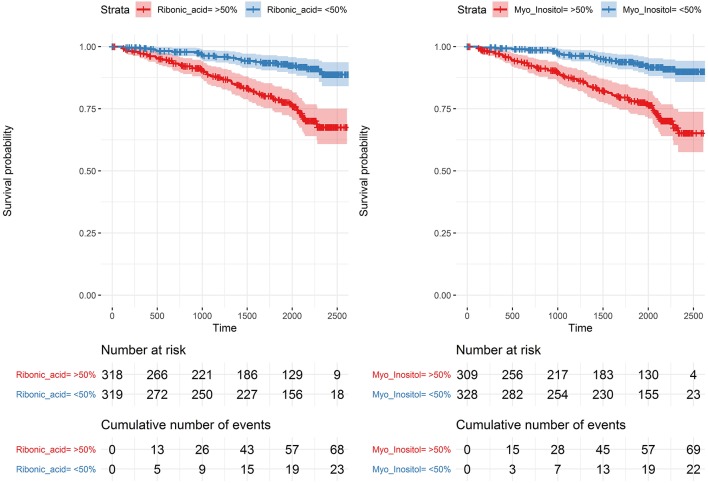
Kaplan–Meier curves for eGFR decline ≥ 30% for levels of myo-inositol and ribonic acid. Above (red) and below (blue) the median with 95% confidence intervals.

In analyses of each of the endpoints included in the combined renal endpoint several metabolites were associated with all-cause mortality, eGFR decline ≥30% and ESRD in crude models although most of these associations were attenuated after multivariate adjustments (Figure 4). Higher levels of the polyols ribonic acid and myo-inositol were though steadily associated with a higher risk of eGFR decline ≥30%, also in the multivariate Cox model (*p*_BH_ < 0.002). The baseline median level of both ribonic acid and myo-inositol in individuals with eGFR decline ≥30% was equal to 75% quartile in individuals who did not experience this event (Supplementary Material, p. 188). Kaplan–Meier curves for eGFR decline ≥30% demonstrated that individuals with values above the median levels of myo-inositol and ribonic acid had a higher risk of eGFR decline ≥30% (Figure 5).

In sample size calculation for replicating the reported associations to the combined renal endpoint, required sample sizes ranged from 96 to 753 with 3,4-dihydroxybutanoic acid at 96, ribitol at 98, ribonic acid at 115 and myo-inositol at 128. A full table of the estimated required sample sizes are reported in the Supplementary Material, p. 186.

### Sensitivity Analyses

Sensitivity analyses with additional adjustments to previous cardiovascular disease (*n* = 137) and retinopathy were performed (Supplementary Material, p. 129, 149). In a further sensitivity analysis, an endpoint of development of eGFR decline ≥40% (*n* = 54) was tested (Supplementary Material, p. 169). In brief, the findings reported in this study proved robust in all sensitivity analyses.

## Discussion

We examined serum metabolomic profiles in persons with type 1 diabetes to evaluate potential cross-sectional associations with renal function (eGFR) and albuminuria as the clinical features of DKD. Together with the follow up information of the cohort, we analyzed if metabolites were also associated with progressing renal function impairment, ESRD and mortality.

The main findings were: (1) Polyols ribonic acid and myo-inositol were inversely associated with eGFR and positively associated with macroalbuminuria in cross-sectional analyses. Both were also associated with higher risk of eGFR decline ≥30% and ribonic acid was associated with higher risk of the combined renal endpoint. (2) Among the investigated clinical variables, eGFR had the highest number of significant associations with metabolites. (3) Amino acids isoleucine, leucine and valine had significant positive cross-sectional associations with eGFR. Further, they were associated with lower risk of the combined renal endpoint in longitudinal analyses. (4) 3,4-DB had positive cross-sectional association with micro- and macroalbuminuria, UAER and inverse association with eGFR.

In the present study of individuals with type 1 diabetes, higher levels of myo-inositol and ribonic acid, also known as ribonate, were associated with a higher risk of all-cause mortality and ESRD, although only in crude models. In addition to ESRD, both the metabolites were associated with a high risk of eGFR decline ≥30%, and ribonic acid was associated with higher risk of the combined renal endpoint.

Interestingly, a previous study in individuals with CKD reported associations between ribonate, fumarate and allantoin and a higher risk of all-cause mortality (21), although only 10% in the discovery cohort and none in the replication cohort had diabetes. In another study from the same cohorts, four metabolites were associated with ESRD (22), however, there was no overlap between those findings and the current.

Ribonate can be synthesized from ribose which is involved in the pentose phosphate pathway. A previous study performed in rat models demonstrated that inducing diabetes resulted in a higher activity of key enzymes of the pathway, suggesting a potential role of the pentose phosphate pathway in early diabetes-induced renal hypertrophy (23). Whether this is also the case in humans is currently unknown.

However, four pathogenic molecular pathways in diabetes complications induced by high glucose influx into endothelial cells and production of reactive oxygen species causing accumulation of glycolysis intermediates have previously been proposed by Brownlee (24). The pentose phosphate pathway is not directly involved in the four pathways but is interconnected with glycolysis. Myo-inositol may though be directly affected by one of the four pathways, the polyol pathway (25).

Our results in type 1 diabetes replicate previous results in type 2 diabetes where higher levels of myo-inositol were also associated with higher risk of ESRD (3). In a cross-sectional study of retinopathy in type 2 diabetes, myo-inositol and metabolites of the pentose phosphate pathway were associated with diabetic non-proliferative retinopathy (26).

Increased amounts of amino acids isoleucine, leucine, and valine were associated with lower risk of ESRD in crude models and with lower risk of the combined renal endpoint in adjusted models. In a study from Joslin Diabetes Center in individuals with type 2 diabetes, comparing progressors to ESRD with non-progressors over 8–12 years follow-up, higher levels of leucine and valine were also associated with lower risk of progression to ESRD, although not significant after adjustment for HbA_1c_, albumin excretion, eGFR and multiple testing (3). In another study of circulating amino acids in individuals with type 2 diabetes from the ADVANCE study, higher levels of leucine were associated with a lower risk of all-cause mortality (7).

A possible link between BCAAs, such as leucine and isoleucine, development of diabetes (27), insulin resistance (28) and the gut microbiome (12), have previously been hypothesized. Gut dysbiosis has been proposed as a mechanism leading to inflammation, a leaky gut barrier and renal and endothelial damage by circulating gut bacteria derived metabolites (29).

The hydroxy butyrate 3,4-DB was significantly associated with all cross-sectional endpoints and 2,4-DB was associated with macroalbuminuria. Both these hydroxy butyrates were also associated with all-cause mortality, eGFR decline ≥30% and ESRD in crude models and have previously been shown to be associated with retinopathy (26). In the network analysis they were strongly correlated with creatinine and the polyols and they may reflect renal clearing.

Other short-chain fatty acids have previously been proposed to derive from the gut microbiota (30) and a study of fecal samples in type 1 diabetes demonstrated lower levels of the short-chain fatty acid butyrate and other related compounds when compared to healthy controls (31). In a study from Joslin Diabetes Center including individuals with type 1 diabetes, there were no overlapping results with the present study in terms of metabolites associated with eGFR slope or ESRD. Part of the explanation for these differing results may be that only metabolites associating significantly with eGFR slope were tested for association to ESRD and that the different platforms used to a large extent generate data on non-overlapping metabolites (9).

We recognize several limitations in our study. Data concerning potential changes in concomitant medication during follow-up were not available, although particularly changes in antihypertensives and statins would have been of relevance. Data regarding diet, gut microbiota composition or day-to-day variability were not available, although these factors may affect the measured metabolome (32, 33).

The main limitation in this study is the lack of a replication cohort. Validation in an independent cohort is a requirement for assessment of potential biomarkers. A general challenge in the metabolomics field, which also applies here, is the diversity of analysis platforms and the quantification on a relative scale as no analytic platform can capture all metabolites and thus we may have missed relevant metabolites with our platform. Despite this, we did identify polyols and amino acids that have also demonstrated significant associations to relevant outcomes in previous studies. The major strengths of this study are the large population of individuals with type 1 diabetes, the well-described comprehensive analyses of metabolites, and, the availability of longitudinal register data for up to 7 years.

In conclusion, a broad range of associations between metabolites, particularly polyols, amino acids, and hydroxybutyrates with renal endpoints in type 1 diabetes were revealed. These findings highlight molecular pathways associated with progression of kidney disease, however, need external validation to further assess their roles and potentials as future therapeutic targets.

## Data Availability Statement

The datasets for this manuscript are not publicly available but may be requested by researchers who have the relevant legal permissions from the data protection agency. Requests to access the datasets should be directed to PR, peter.rossing@regionh.dk.

## Ethics Statement

The studies involving human participants were reviewed and approved by The Ethics Committee E, Region Hovedstaden, Denmark. The patients/participants provided their written informed consent to participate in this study.

## Author Contributions

TS performed the statistical analysis. NT drafted the manuscript. NT and TS had full access to the data in the study. All authors conceived and designed the research, carefully interpreted the data, critically revised the manuscript, and approved the final version.

### Conflict of Interest

SW was employed by the company Novo Nordisk. PR reports personal shares in Novo Nordisk, having given lectures for Mundi Pharma, Eli Lilly and Boehringer Ingelheim, being in the advisory board for Novo Nordisk, MSD, Bayer, Astellas, AbbVie, Sanofi, and Boehringer Ingelheim, being a steering group member for Gilead, Astra Zeneca, Bayer and Novo Nordisk. All honorarium given to institution. The remaining authors declare that the research was conducted in the absence of any commercial or financial relationships that could be construed as a potential conflict of interest.
