# Supplementary material for: Metabolomic Assessment Reveals Alteration in Polyols and Branched Chain Amino Acids Associated With Present and Future Renal Impairment in a Discovery Cohort of 637 Persons With Type 1 Diabetes
Source: Front Endocrinol (Lausanne). 2019 Nov 22;10:818. doi: 10.3389/fendo.2019.00818 (PMC6883958; doi:10.3389/fendo.2019.00818)
Supplement: Supplementary file 1 [file Image_1.pdf]

Supplementary Figure S1 – Incidence of the combined renal endpoint and specific endpoints in persons with normo-, micro- and macroalbuminuria.

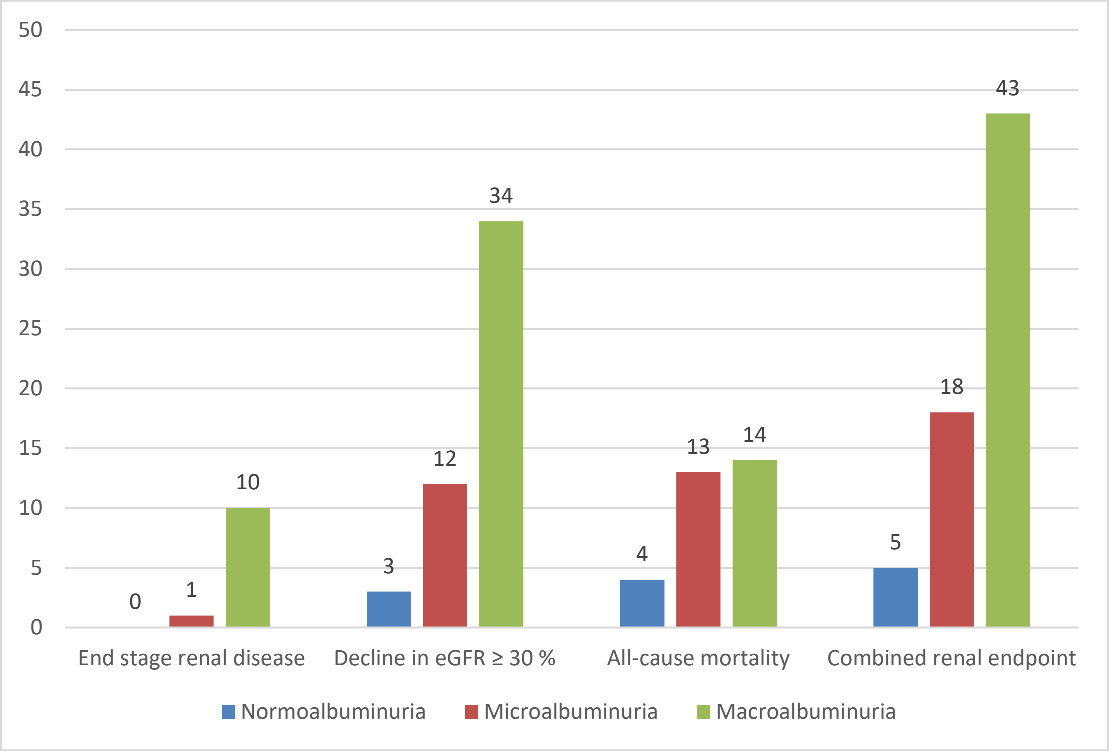


Numbers indicate percentage of individuals in each group reaching the endpoint.
