# Supplementary material for: Metabolomic Assessment Reveals Alteration in Polyols and Branched Chain Amino Acids Associated With Present and Future Renal Impairment in a Discovery Cohort of 637 Persons With Type 1 Diabetes
Source: Front Endocrinol (Lausanne). 2019 Nov 22;10:818. doi: 10.3389/fendo.2019.00818 (PMC6883958; doi:10.3389/fendo.2019.00818)
Supplement: Supplementary file 2 [file Table_1.docx]

**Supplementary table S1: Proportion of observations that were imputed**

| **Name** | **Proportion imputed (%)** |
| --- | --- |
| Stearic acid | 0.1 |
| Heptadecanoic acid | 0.1 |
| Oleic acid | 0.2 |
| Palmitic acid | 0.2 |
| Arachidic acid | 0.2 |
| Docosahexaenoic acid | 0.2 |
| Linoleic acid | 0.4 |
| Lactic acid | 0.5 |
| Alanine | 0.9 |
| Malic acid | 1.0 |
| Serine | 1.0 |
| Isoleucine | 1.0 |
| Leucine | 1.0 |
| Valine | 1.0 |
| Cholesterol | 1.0 |
| 4-Deoxytetronic acid | 1.0 |
| 1,3-Propanediol | 1.0 |
| Dodecanoic acid | 1.0 |
| L-5-Oxoproline | 1.0 |
| Nonadecanoic acid | 1.0 |
| Myo inositol | 2.0 |
| Citric acid | 2.0 |
| Glutamic acid | 2.0 |
| Fumaric acid | 2.0 |
| 3-Hydroxybutyric acid | 2.0 |
| Threonine | 2.0 |
| Phenylalanine | 2.0 |
| Methionine | 2.0 |
| Glycine | 2.0 |
| Proline | 2.0 |
| 2-Hydroxybutyric acid | 2.0 |
| alpha-Tocopherol | 2.0 |
| 3,4-Dihydroxybutanoic acid | 2.0 |
| 2,4-Dihydroxybutanoic acid | 2.0 |
| Glyceric acid | 2.0 |
| 3-Indoleacetic acid | 2.0 |
| 4-Hydroxybutanoic acid | 2.0 |
| 4-Hydroxyphenyllactic acid | 2.0 |
| Aminomalonic acid | 2.0 |
| Bisphenol A | 2.0 |
| Campesterol | 2.0 |
| Creatinine | 2.0 |
| Decanoic acid | 2.0 |
| Ethanolamine | 2.0 |
| Heptadecanoic acid | 2.0 |
| Pyroglutamic acid | 2.0 |
| Ribitol | 2.0 |
| Succinic acid | 3.0 |
| alpha-ketoglutaric acid | 3.0 |
| Arachidonic acid | 3.0 |
| 1-Dodecanol | 3.0 |
| 2-Palmitoylglycerol | 3.0 |
| Arabinopyranose | 3.0 |
| Eicosapentaenoic acid | 3.0 |
| Glycerol | 3.0 |
| Glyceryl-glycoside | 3.0 |
| Hydroxylamine | 3.0 |
| Nonanoic acid | 3.0 |
| Octanoic acid | 3.0 |
| Ribitol (2) | 3.0 |
| Ribonic acid | 3.0 |
| Tridecanoic acid | 3.0 |
| 11-Eicosenoic acid | 4.0 |
| 3-Indolepropionic acid | 4.0 |
| 4-Hydroxybenzeneacetic acid | 4.0 |
| Benzeneacetic acid | 4.0 |
| Hydroxyproline | 4.0 |
| Tartronic acid | 4.0 |
| Tyrosine | 4.0 |
| Pyruvic acid | 5.0 |
| 4-Deoxytetronic acid | 5.0 |
| 1-Monopalmitin | 5.0 |
| Glycerol | 5.0 |
| Myristoleic acid | 5.0 |
| 2-hydroxy Isovaleric acid | 7.0 |
