# Supplementary material for: Metabolomic Assessment Reveals Alteration in Polyols and Branched Chain Amino Acids Associated With Present and Future Renal Impairment in a Discovery Cohort of 637 Persons With Type 1 Diabetes
Source: Front Endocrinol (Lausanne). 2019 Nov 22;10:818. doi: 10.3389/fendo.2019.00818 (PMC6883958; doi:10.3389/fendo.2019.00818)
Supplement: Supplementary file 3 [file Data_Sheet_1.PDF]

0033_PROFIL_2017: Statistical Analysis of the Metabolome in Relation to Renal Complications

Tommi Suvitaival, tommi.raimo.leo.suvitaival@regionh.dk, Steno Diabetes Center Copenhagen

October 18, 2019

Abstract

This document is Supplementary Material to the paper *Sugar Derivatives and Branched-Chain Amino Acids Are Associated with Present and Future Renal Impairment in Type 1 Diabetes* by Nete Tofte, Tommi Suvitaival, Kajetan Trost, Ismo Mattila, Simone Theilade, Signe A. Winther, Tarun S. Ahluwalia, Marie Frimodt-M{o}ller, Cristina Legido-Quigley and Peter Rossing.

Contents

1 Step 1: Cross-Sectional Analysis of All Metabolites . . . 6
1.1 Albuminuria Groups . . . 6
1.1.1 Crude Model . . . 6
1.1.1.1 Table of Model Coefficients . . . 7
1.1.2 Adjusted Model . . . 12
1.1.2.1 Table . . . 13
1.1.2.2 Figure . . . 17
1.1.2.2.1 Heatmap of Model Coefficients . . . 17
1.1.2.2.2 Bipartite Graph of Model Coefficients . . . 19
1.2 eGFR . . . 21
1.2.1 Crude Model . . . 21
1.2.1.1 Table . . . 22
1.2.2 Adjusted Model . . . 24
1.2.2.1 Table . . . 25
1.2.2.2 Figures . . . 42
1.2.2.2.1 Heatmap of Model Coefficients . . . 42
1.2.2.2.2 Bipartite Graph of Model Coefficients . . . 44
1.2.2.2.3 Forest Plot of Model Coefficients . . . 46
1.2.3 Technical-Adjusted Model . . . 47
1.2.3.1 Table . . . 50
1.3 logUAER – Continuous Albuminuria . . . 52
1.3.1 Crude Model . . . 52
1.3.1.1 Table . . . 53
1.3.2 Adjusted Model . . . 55
1.3.2.1 Table . . . 56

2 Step 2: Survival Analysis of Combined Renal Endpoint in Relation to Prioritized Metabolites from Step 1 . . . 58
2.1 Step 2A: Crude Model . . . 58
2.1.1 Table . . . 59
2.2 Step 2B: Adjusted Model . . . 60
2.2.1 Table . . . 61
2.2.2 Forest Plot . . . 62
2.3 Combined Forest Plot from Crude and Adjusted Models . . . 63

3 Step 3: Survival Analysis of Specific Renal Endpoints in Relation to Prioritized Metabolites from Step 3A 64
3.1 Step 3A: All-Cause Mortality . . . 64
3.1.1 Crude Model . . . 64
3.1.1.1 Table . . . 65
3.1.1.2 Forest Plot . . . 66
3.1.2 Adjusted Model . . . 67
3.1.2.1 Table . . . 68
3.1.2.2 Forest Plot . . . 69
3.1.3 Combined Forest Plot from Crude and Adjusted Models . . . 70
3.2 Step 3B: eGFR Decline > 30 % . . . 71
3.2.1 Crude Model . . . 71
3.2.1.1 Table . . . 72
3.2.2 Forest Plot . . . 73
3.2.3 Adjusted Model . . . 74
3.2.3.1 Table . . . 75
3.2.3.2 Forest Plot . . . 76
3.2.4 Combined Forest Plot from Crude and Adjusted Models . . . 77
3.3 Step 3C: End-Stage Renal Disease . . . 78
3.3.1 Crude Model . . . 78
3.3.1.1 Table . . . 79
3.3.1.2 Forest Plot . . . 80
3.3.2 Adjusted Model . . . 81
3.3.2.1 Table . . . 82
3.3.2.2 Forest Plot . . . 83
3.3.3 Combined Forest Plot from Crude and Adjusted Models . . . 84
3.4 Compilation Forest Plot from Steps 3A-C . . . 85

4 Step 4: Detailed Assessment of the Top-Metabolites in Relation to Outcomes 89
4.1 Step 4.1: First Top-Metabolite in Relation to eGFR Decline . . . 89
4.1.1 Step 4.1A: Analysis of Full Cohort . . . 89
4.1.1.1 Survival Model with Details . . . 89
4.1.1.1.1 Forest Plot with Clinical Variables . . . 90
4.1.1.1.2 Diagnostics of the Survival Model . . . 92
4.1.1.1.3 Kaplan-Maier Curve with Median Cutpoint . . . 94
4.1.1.2 Other Model Fits . . . 95
4.1.2 Step 4.1B: Analysis of a Blood Pressure, HbA1c and logUAER-Matched Subcohort . . 98
4.1.2.1 Survival Model with Details . . . 101
4.1.2.1.1 Forest Plot with Clinical Variables . . . 103
4.1.2.1.2 Diagnostics of the Survival Model . . . 105
4.1.2.1.3 Kaplan-Maier Curve with Median Cutpoint . . . 107
4.1.2.1.4 Boxplots . . . 108
4.2 Step 4.2: Second Top-Metabolite in Relation to eGFR Decline (> 30 %) . . . 109
4.2.1 Step 4.2A: Analysis of Full Cohort . . . 109
4.2.1.1 Survival Model with Details . . . 109
4.2.1.1.1 Forest Plot with Clinical Variables . . . 110
4.2.1.1.2 Diagnostics of the Survival Model . . . 112
4.2.1.1.3 Kaplan-Maier Curve with Median Cutpoint . . . 114
4.2.1.2 Other Model Fits . . . 115
4.2.2 Step 4.1B: Analysis of a Blood Pressure, HbA1c and logUAER-Matched Subcohort . . 118
4.2.2.1 Survival Model with Details . . . 121
4.2.2.1.1 Forest Plot with Clinical Variables . . . 123
4.2.2.1.2 Diagnostics of the Survival Model . . . 125
4.2.2.1.3 Kaplan-Maier Curve with Median Cutpoint . . . 127

4.2.2.1.4 Boxplots . . . 128

5 Sensitivity Analysis **129**
5.1 Adjustment to Previous Cardiovascular Disease . . . 129
5.1.1 Step 2B: Adjusted Model . . . 129
5.1.1.1 Table . . . 130
5.1.1.2 Forest Plot . . . 131
5.1.2 Step 3: Survival Analysis of Specific Renal Endpoints in Relation to Prioritized Metabolites from Step 2A . . . 132
5.1.2.1 Step 3A: All-Cause Mortality . . . 132
5.1.2.1.1 Table . . . 133
5.1.2.1.2 Forest Plot . . . 134
5.1.2.2 Step 3B: eGFR Decline > 30 % . . . 135
5.1.2.2.1 Table . . . 136
5.1.2.2.2 Forest Plot . . . 137
5.1.2.3 Step 3C: End-Stage Renal Disease . . . 138
5.1.2.3.1 Adjusted Model . . . 138
5.1.3 Compilation Forest Plot from Steps 3A-C . . . 141
5.1.4 Step 4: Detailed Assessment of the Top-Metabolites in Relation to Outcomes . . . 143
5.1.4.1 Ribonic Acid . . . 143
5.1.4.1.1 Forest Plot with Clinical Variables . . . 144
5.1.4.2 Myo-Inositol . . . 146
5.1.4.2.1 Forest Plot with Clinical Variables . . . 147
5.2 Adjustment to Any Retinopathy . . . 149
5.2.1 Step 2B: Adjusted Model . . . 149
5.2.1.1 Table . . . 150
5.2.1.2 Forest Plot . . . 151
5.2.2 Step 3: Survival Analysis of Specific Renal Endpoints in Relation to Prioritized Metabolites from Step 2A . . . 152
5.2.2.1 Step 3A: All-Cause Mortality . . . 152
5.2.2.1.1 Table . . . 153
5.2.2.1.2 Forest Plot . . . 154
5.2.2.2 Step 3B: eGFR Decline > 30 % . . . 155
5.2.2.2.1 Table . . . 156
5.2.2.2.2 Forest Plot . . . 157
5.2.2.3 Step 3C: End-Stage Renal Disease . . . 158
5.2.2.3.1 Adjusted Model . . . 158
5.2.3 Compilation Forest Plot from Steps 3A-C . . . 161
5.2.4 Step 4: Detailed Assessment of the Top-Metabolites in Relation to Outcomes . . . 163
5.2.4.1 Ribonic Acid . . . 163
5.2.4.1.1 Forest Plot with Clinical Variables . . . 164
5.2.4.2 Myo-Inositol . . . 166
5.2.4.2.1 Forest Plot with Clinical Variables . . . 167
5.3 eGFR Decline > 40 % . . . 169
5.3.1 Step 3B . . . 169
5.3.1.1 Crude Model . . . 169
5.3.1.1.1 Table . . . 170
5.3.1.1.2 Forest Plot . . . 171
5.3.1.2 Adjusted Model . . . 172
5.3.1.2.1 Table . . . 173
5.3.1.2.2 Forest Plot . . . 174
5.3.1.3 Combined Forest Plot from Crude and Adjusted Models . . . 175
5.3.2 Step 4: Detailed Assessment of the Top-Metabolites in Relation to Outcomes . . . 176
5.3.2.1 Step 4.1: First Top-Metabolite in Relation to eGFR Decline (> 40 %) . . . 176

5.3.2.1.1 Step 4.1A: Analysis of Full Cohort 176
5.3.2.1.2 Kaplan-Maier Curve with Median Cutpoint 178
5.3.2.2 Step 4.2: Second Top-Metabolite in Relation to eGFR Decline (> 40 %) . . 179
5.3.2.2.1 Step 4.2A: Analysis of Full Cohort 179
5.3.2.2.2 Forest Plot with Clinical Variables 180
5.3.2.2.3 Kaplan-Maier Curve with Median Cutpoint 181
5.3.2.3 Step 4.3: Third Top-Metabolite in Relation to eGFR Decline (> 40 %) . . . 182
5.3.2.3.1 Step 4.3A: Analysis of Full Cohort 182
5.3.2.3.2 Forest Plot with Clinical Variables 183
5.3.2.3.3 Kaplan-Maier Curve with Median Cutpoint 184

6 Sample Size Calculation for Combined Renal Endpoint 185
6.0.3 Table 186

7 Boxplots 188
7.1 Entire Cohort 188

8 Correlation Matrix 189

9 PCA 190
9.1 Groups 190
9.2 eGFR 191
9.3 logUAER 192

10 Scatter Plots 193

11 Combined Subfigures for the Two Metabolites of Interest 217

12 Partial Correlation Network 221
12.1 Full Network 222
12.2 Connected Network 224
12.2.1 Connected Network – Publication 226
12.2.2 Connected Network – Publication with Legends 228
12.2.3 Partial Correlations with eGFR 231

13 Supplementary Step 1: Crude Associations of All Metabolites to Clinical Changes Over Time 232
13.1 eGFR Slope 232
13.1.1 Table 233
13.2 Albuminuria Slope 237
13.2.1 Table 238

14 Supplementary Step 2: Adjusted Associations to Clinical Changes Over Time with Prioritized Metabolites 242
14.1 Supplementary Step 2A: Adjusted Model for Albuminuria Slope with Prioritized Metabolites 242
14.1.1 Table 243
14.2 Supplementary Step 2B: Adjusted Model for eGFR Slope with Prioritized Metabolites 246
14.2.1 Table 247

15 Clinical Characteristics 257

16 Quality Control Table of Metabolite Measurements 261

17 Appendix 263

```
# data <- data[ data$"Group" == "T1D Control", ] # Normo-albuminuria
```

1 Step 1: Cross-Sectional Analysis of All Metabolites

1.1 Albuminuria Groups

1.1.1 Crude Model

```
## [1] 665   4

##      (Intercept) GroupT1D Micro GroupT1D Macro GroupHealthy Control
## [1,]           1              0              0                    0
## [2,]           1              1              1                    1

##
##                                  Overall
##   n                                665
##   Group (%)
##      T1D Control                   290 (43.6)
##      T1D Micro                     152 (22.9)
##      T1D Macro                     178 (26.8)
##      Healthy Control                45 ( 6.8)
##   Age (mean (sd))                54.07 (12.74)
##   Gender (mean (sd))              0.54 (0.50)
##   Hba1c_baseline (mean (sd))      7.88 (1.31)
##   egfr (mean (sd))               83.67 (27.64)
##   CALSBP (mean (sd))            131.45 (17.41)
##   bmi (mean (sd))                25.24 (4.00)
##   Smoking (mean (sd))             0.20 (0.40)
##   Statin (mean (sd))              0.56 (0.50)
##   log_Blood_TGA (mean (sd))       0.00 (0.69)
##   Total_cholesterol (mean (sd))   4.71 (0.87)
```

1.1.1.1 Table of Model Coefficients

```
##
##
## Table: GroupT1D Micro
##
## Name                             Effect       CI.L      CI.R   AveExpr    P.Value   adj.P.Val
## --------------------------  ---------  ---------  --------  --------  ---------  ----------
## 3,4-Dihydroxybutanoic aci      0.26500    0.15700    0.3720      15.9   1.70e-06    0.000126
## 2,4-Dihydroxybutanoic aci      0.24200    0.12200    0.3630      16.5   9.08e-05    0.003410
## Ribitol; 70                    0.24100    0.11400    0.3690      20.2   2.17e-04    0.005430
## Ribitol; 71                    0.24100    0.11000    0.3710      23.3   3.04e-04    0.005710
## 4-Hydroxybenzeneacetic ac      0.40800    0.16600    0.6510      19.4   1.00e-03    0.012400
## 4-Deoxytetronic acid; 32       0.23800    0.09510    0.3810      21.6   1.13e-03    0.012400
## Myo inositol 6TMS; 1           0.17400    0.06930    0.2780      19.0   1.15e-03    0.012400
## Creatinine; 50                 0.24500    0.09040    0.4000      21.6   1.96e-03    0.018400
## Ribonic acid; 72               0.25700    0.07560    0.4380      20.0   5.53e-03    0.046100
## Heptadecanoic acid; 60        -0.13500   -0.23500   -0.0350      21.8   8.22e-03    0.061600
## Glutamic acid, 3TMS; 8         0.13800    0.02960    0.2460      23.0   1.27e-02    0.086300
## 4-Hydroxyphenyllactic aci      0.14200    0.01710    0.2670      20.8   2.59e-02    0.162000
## Pyroglutamic acid; 69          0.17900    0.01510    0.3430      22.9   3.23e-02    0.186000
## Threonine, 3TMS; 12           -0.10800   -0.20800   -0.0071      28.6   3.59e-02    0.192000
## Malic acid, 3TMS; 11           0.09650   -0.00439    0.1970      20.0   6.08e-02    0.293000
## Alanine, 2TMS; 25              0.07220   -0.00381    0.1480      22.8   6.26e-02    0.293000
## 2-hydroxy Isovaleric acid     -0.22500   -0.47100    0.0211      22.2   7.31e-02    0.323000
## Fumaric acid, 2TMS; 9          0.06750   -0.01040    0.1450      24.1   8.92e-02    0.366000
## Nonanoic acid; 67             -0.06840   -0.14900    0.0122      24.4   9.60e-02    0.366000
## alpha-ketoglutaric acid,       0.15400   -0.02830    0.3370      20.3   9.76e-02    0.366000
## 11-Eicosenoic acid; 35         0.12900   -0.02980    0.2870      21.9   1.11e-01    0.394000
## Arachidic acid; 46            -0.04890   -0.11100    0.0132      21.8   1.22e-01    0.394000
## Glycine, 3TMS; 17              0.05470   -0.01480    0.1240      21.4   1.23e-01    0.394000
## Linoleic acid, TMS; 4         -0.05130   -0.11800    0.0153      25.8   1.31e-01    0.394000
## Glyceryl-glycoside; 59         0.10900   -0.03390    0.2520      20.8   1.34e-01    0.394000
## Isoleucine, 2TMS; 18          -0.08810   -0.20500    0.0291      26.0   1.41e-01    0.394000
## 3-Indoleacetic acid; 40        0.11900   -0.04090    0.2790      20.5   1.44e-01    0.394000
## Dodecanoic acid; 54            0.09310   -0.03280    0.2190      22.7   1.47e-01    0.394000
## Succinic acid, 2TMS; 7         0.04800   -0.01790    0.1140      22.7   1.53e-01    0.396000
## Valine, 2TMS; 20              -0.05300   -0.12800    0.0224      25.1   1.68e-01    0.419000
## Hydroxyproline; 64             0.12000   -0.05510    0.2950      20.6   1.79e-01    0.433000
## Serine, 3TMS; 14              -0.04980   -0.12600    0.0265      26.0   2.01e-01    0.466000
## Heptadecanoic acid; 61        -0.04470   -0.11500    0.0253      23.4   2.10e-01    0.466000
## Cholesterol, TMS; 23          -0.04200   -0.10800    0.0239      21.7   2.11e-01    0.466000
## Stearic acid, TMS; 2          -0.02400   -0.06310    0.0151      17.4   2.29e-01    0.487000
## Pyruvic acid; 31               0.11900   -0.07680    0.3140      19.5   2.34e-01    0.487000
## Leucine, 2TMS; 19             -0.06130   -0.16700    0.0439      21.1   2.53e-01    0.493000
## Methionine, 2TMS; 16          -0.07110   -0.19400    0.0516      20.3   2.56e-01    0.493000
## Palmitic acid, TMS; 5         -0.02930   -0.08000    0.0214      21.4   2.57e-01    0.493000
## Bisphenol A; 48               -0.06170   -0.17000    0.0465      21.2   2.63e-01    0.493000
## Benzeneacetic acid; 47         0.11100   -0.08920    0.3100      20.3   2.77e-01    0.508000
## Lactic acid; 29                0.03800   -0.03180    0.1080      25.4   2.85e-01    0.509000
## Campesterol; 49                0.07570   -0.07330    0.2250      21.8   3.19e-01    0.535000
## Phenylalanine, 2TMS; 13        0.03280   -0.03200    0.0977      24.3   3.20e-01    0.535000
## Tyrosine; 75                  -0.12000   -0.36000    0.1200      23.3   3.26e-01    0.535000
## Docosahexaenoic acid; 53      -0.07220   -0.21700    0.0726      24.2   3.28e-01    0.535000
```

```
## Glycerol; 57                -0.04080   -0.13200    0.0501      28.7   3.79e-01    0.599000
## 1-Monopalmitin; 37           0.03760   -0.04700    0.1220      29.2   3.83e-01    0.599000
## 2-Hydroxybutyric acid, 2T   -0.06300   -0.21200    0.0861      20.7   4.07e-01    0.623000
## Arachidonic acid, TMS; 24   -0.03060   -0.11200    0.0508      22.7   4.61e-01    0.691000
## Citric acid, 4TMS; 6         0.02850   -0.06960    0.1270      20.8   5.68e-01    0.835000
## Glycerol; 58                -0.07040   -0.31900    0.1790      24.9   5.79e-01    0.835000
## Ethanolamine; 56             0.02260   -0.06200    0.1070      22.7   6.00e-01    0.849000
## Tridecanoic acid; 74         0.02640   -0.08560    0.1380      20.0   6.44e-01    0.871000
## Aminomalonic acid; 45        0.02830   -0.09190    0.1480      24.3   6.44e-01    0.871000
## Arabinopyranose; 51          0.03630   -0.12100    0.1940      20.1   6.51e-01    0.871000
## Eicosapentaenoic acid; 55    0.03440   -0.14400    0.2120      23.0   7.04e-01    0.914000
## Myristoleic acid; 65         0.03920   -0.17100    0.2500      19.9   7.15e-01    0.914000
## 3-Indolepropionic acid; 4   -0.04440   -0.28700    0.1980      19.5   7.19e-01    0.914000
## 3-Hydroxybutyric acid, 2T   -0.02760   -0.19100    0.1360      21.6   7.40e-01    0.919000
## Oleic acid, TMS; 3          -0.01340   -0.09670    0.0698      17.1   7.51e-01    0.919000
## Decanoic acid; 52            0.01850   -0.10000    0.1370      22.4   7.60e-01    0.919000
## 4-Deoxytetronic acid; 33     0.02620   -0.17700    0.2290      22.2   8.01e-01    0.935000
## Octanoic acid; 68           -0.00912   -0.08060    0.0623      24.2   8.02e-01    0.935000
## Hydroxylamine; 62            0.01220   -0.08730    0.1120      27.7   8.10e-01    0.935000
## 2-Palmitoylglycerol; 39      0.00866   -0.07010    0.0874      25.9   8.29e-01    0.940000
## 1,3-Propanediol; 34          0.01040   -0.09090    0.1120      24.2   8.40e-01    0.940000
## 1-Dodecanol; 36             -0.00680   -0.09530    0.0817      20.2   8.80e-01    0.971000
## alpha-Tocopherol; 26        -0.00875   -0.15000    0.1320      18.8   9.03e-01    0.980000
## 4-Hydroxybutanoic acid; 4    0.00226   -0.03960    0.0441      27.3   9.15e-01    0.980000
## Proline, 2TMS; 21            0.00486   -0.10100    0.1110      20.8   9.28e-01    0.980000
## Nonadecanoic acid; 66        0.00332   -0.09800    0.1050      20.0   9.49e-01    0.982000
## Glyceric acid; 30            0.00317   -0.10900    0.1160      20.4   9.56e-01    0.982000
## L-5-Oxoproline; 63          -0.00119   -0.07200    0.0696      27.5   9.74e-01    0.985000
## Tartronic acid; 73           0.00169   -0.17900    0.1820      21.3   9.85e-01    0.985000
##
##
## Table: GroupT1D Macro
##
## Name                           Effect       CI.L       CI.R   AveExpr    P.Value   adj.P.Val
## -------------------------  ---------  ---------  ---------  --------  ---------  ----------
## 3,4-Dihydroxybutanoic aci     0.51600    0.41400    0.61900      15.9   0.00e+00    0.00e+00
## Ribonic acid; 72              0.77000    0.59800    0.94200      20.0   0.00e+00    0.00e+00
## Myo inositol 6TMS; 1          0.43700    0.33800    0.53700      19.0   0.00e+00    0.00e+00
## 2,4-Dihydroxybutanoic aci     0.49500    0.38000    0.60900      16.5   0.00e+00    0.00e+00
## Ribitol; 71                   0.45800    0.33400    0.58100      23.3   0.00e+00    0.00e+00
## 4-Hydroxybenzeneacetic ac     0.83000    0.59900    1.06000      19.4   0.00e+00    0.00e+00
## 4-Deoxytetronic acid; 32      0.34900    0.21300    0.48400      21.6   6.00e-07    5.80e-06
## 4-Deoxytetronic acid; 33      0.49600    0.30200    0.68900      22.2   6.00e-07    5.80e-06
## Creatinine; 50                0.35000    0.20300    0.49800      21.6   3.80e-06    3.13e-05
## Glyceryl-glycoside; 59        0.31300    0.17700    0.44900      20.8   7.50e-06    5.66e-05
## Hydroxyproline; 64            0.36500    0.19900    0.53200      20.6   1.88e-05    1.28e-04
## Valine, 2TMS; 20             -0.14900   -0.22100   -0.07750      25.1   4.93e-05    3.08e-04
## 3-Indolepropionic acid; 4    -0.46800   -0.69800   -0.23700      19.5   7.45e-05    4.30e-04
## Methionine, 2TMS; 16         -0.22000   -0.33600   -0.10300      20.3   2.38e-04    1.27e-03
## Docosahexaenoic acid; 53     -0.24300   -0.38100   -0.10500      24.2   5.60e-04    2.80e-03
## Ribitol; 70                   0.20500    0.08410    0.32700      20.2   9.30e-04    4.36e-03
## Cholesterol, TMS; 23         -0.10400   -0.16600   -0.04110      21.7   1.20e-03    5.03e-03
## Octanoic acid; 68            -0.11300   -0.18000   -0.04460      24.2   1.21e-03    5.03e-03
## 2-hydroxy Isovaleric acid    -0.38100   -0.61500   -0.14700      22.2   1.43e-03    5.65e-03
```

```
## Threonine, 3TMS; 12          -0.15100   -0.24700   -0.05560       28.6   1.98e-03    7.43e-03
## Eicosapentaenoic acid; 55    -0.26300   -0.43200   -0.09340       23.0   2.40e-03    8.56e-03
## Serine, 3TMS; 14             -0.10600   -0.17900   -0.03390       26.0   4.11e-03    1.40e-02
## 4-Hydroxyphenyllactic aci     0.17000    0.05160    0.28900       20.8   4.99e-03    1.58e-02
## 3-Indoleacetic acid; 40       0.21800    0.06580    0.37000       20.5   5.06e-03    1.58e-02
## Proline, 2TMS; 21             0.13900    0.03820    0.23900       20.8   6.90e-03    2.05e-02
## Tyrosine; 75                 -0.31300   -0.54100   -0.08550       23.3   7.11e-03    2.05e-02
## Isoleucine, 2TMS; 18         -0.14900   -0.26100   -0.03780       26.0   8.78e-03    2.44e-02
## Citric acid, 4TMS; 6          0.12000    0.02680    0.21300       20.8   1.17e-02    3.14e-02
## L-5-Oxoproline; 63           -0.08240   -0.15000   -0.01510       27.5   1.65e-02    4.27e-02
## Glyceric acid; 30            -0.13000   -0.23700   -0.02300       20.4   1.73e-02    4.33e-02
## Fumaric acid, 2TMS; 9         0.08940    0.01540    0.16300       24.1   1.79e-02    4.33e-02
## Aminomalonic acid; 45        -0.13400   -0.24800   -0.01980       24.3   2.16e-02    5.05e-02
## Pyroglutamic acid; 69         0.17500    0.01980    0.33100       22.9   2.72e-02    6.18e-02
## Malic acid, 3TMS; 11          0.10500    0.00943    0.20100       20.0   3.14e-02    6.93e-02
## 1-Dodecanol; 36              -0.08730   -0.17100   -0.00316       20.2   4.20e-02    9.00e-02
## Glutamic acid, 3TMS; 8        0.10500    0.00243    0.20800       23.0   4.48e-02    9.34e-02
## Linoleic acid, TMS; 4        -0.05980   -0.12300    0.00352       25.8   6.41e-02    1.30e-01
## alpha-Tocopherol; 26         -0.11500   -0.24900    0.01930       18.8   9.33e-02    1.84e-01
## Nonadecanoic acid; 66        -0.08010   -0.17600    0.01630       20.0   1.03e-01    1.98e-01
## Tridecanoic acid; 74         -0.08750   -0.19400    0.01890       20.0   1.07e-01    2.00e-01
## 4-Hydroxybutanoic acid; 4     0.03230   -0.00745    0.07210       27.3   1.11e-01    2.03e-01
## Leucine, 2TMS; 19            -0.07820   -0.17800    0.02190       21.1   1.26e-01    2.21e-01
## Benzeneacetic acid; 47        0.14800   -0.04200    0.33800       20.3   1.27e-01    2.21e-01
## 2-Hydroxybutyric acid, 2T    -0.10800   -0.24900    0.03430       20.7   1.37e-01    2.34e-01
## Heptadecanoic acid; 60       -0.06930   -0.16400    0.02570       21.8   1.52e-01    2.54e-01
## Glycerol; 58                  0.16400   -0.07260    0.40100       24.9   1.74e-01    2.83e-01
## Succinic acid, 2TMS; 7        0.04310   -0.01960    0.10600       22.7   1.77e-01    2.83e-01
## Arachidic acid; 46           -0.03730   -0.09630    0.02170       21.8   2.15e-01    3.36e-01
## 3-Hydroxybutyric acid, 2T     0.08840   -0.06690    0.24400       21.6   2.64e-01    4.00e-01
## Heptadecanoic acid; 61       -0.03770   -0.10400    0.02890       23.4   2.67e-01    4.00e-01
## alpha-ketoglutaric acid,      0.08550   -0.08830    0.25900       20.3   3.34e-01    4.92e-01
## Hydroxylamine; 62             0.04460   -0.05000    0.13900       27.7   3.55e-01    5.12e-01
## Ethanolamine; 56             -0.03650   -0.11700    0.04390       22.7   3.73e-01    5.18e-01
## Arabinopyranose; 51           0.06620   -0.08350    0.21600       20.1   3.86e-01    5.18e-01
## Alanine, 2TMS; 25             0.03170   -0.04060    0.10400       22.8   3.90e-01    5.18e-01
## Bisphenol A; 48              -0.04500   -0.14800    0.05790       21.2   3.91e-01    5.18e-01
## Glycine, 3TMS; 17             0.02870   -0.03740    0.09490       21.4   3.94e-01    5.18e-01
## Lactic acid; 29               0.02500   -0.04140    0.09130       25.4   4.60e-01    5.89e-01
## Arachidonic acid, TMS; 24     0.02860   -0.04880    0.10600       22.7   4.69e-01    5.89e-01
## 11-Eicosenoic acid; 35        0.05540   -0.09550    0.20600       21.9   4.71e-01    5.89e-01
## Glycerol; 57                 -0.02850   -0.11500    0.05790       28.7   5.17e-01    6.35e-01
## 1-Monopalmitin; 37           -0.02610   -0.10700    0.05440       29.2   5.25e-01    6.35e-01
## Pyruvic acid; 31              0.05130   -0.13500    0.23700       19.5   5.88e-01    6.84e-01
## Tartronic acid; 73           -0.04730   -0.21900    0.12400       21.3   5.89e-01    6.84e-01
## Decanoic acid; 52            -0.03080   -0.14400    0.08230       22.4   5.93e-01    6.84e-01
## Palmitic acid, TMS; 5        -0.01230   -0.06050    0.03590       21.4   6.16e-01    7.00e-01
## Myristoleic acid; 65          0.04800   -0.15200    0.24800       19.9   6.38e-01    7.14e-01
## Nonanoic acid; 67            -0.01260   -0.08920    0.06410       24.4   7.48e-01    8.15e-01
## Stearic acid, TMS; 2         -0.00605   -0.04320    0.03110       17.4   7.50e-01    8.15e-01
## Campesterol; 49               0.01400   -0.12800    0.15600       21.8   8.46e-01    9.04e-01
## Oleic acid, TMS; 3            0.00731   -0.07180    0.08640       17.1   8.56e-01    9.04e-01
## 1,3-Propanediol; 34           0.00581   -0.09060    0.10200       24.2   9.06e-01    9.24e-01
## Dodecanoic acid; 54           0.00698   -0.11300    0.12700       22.7   9.09e-01    9.24e-01
```

```
## Phenylalanine, 2TMS; 13      -0.00348   -0.06510    0.05820      24.3   9.12e-01    9.24e-01
## 2-Palmitoylglycerol; 39       0.00251   -0.07240    0.07740      25.9   9.48e-01    9.48e-01
##
##
## Table: GroupHealthy Control
##
## Name                           Effect       CI.L       CI.R   AveExpr    P.Value    adj.P.Val
## -------------------------  ----------  ---------  ---------  --------  ---------  ----------
## 2,4-Dihydroxybutanoic aci    -0.447000   -0.64000   -0.25300      16.5   6.90e-06    0.000516
## Arabinopyranose; 51          -0.528000   -0.78000   -0.27600      20.1   4.28e-05    0.001600
## Campesterol; 49              -0.447000   -0.68500   -0.20900      21.8   2.49e-04    0.006220
## 3-Hydroxybutyric acid, 2T    -0.450000   -0.71100   -0.18800      21.6   7.71e-04    0.014000
## Glycine, 3TMS; 17            -0.188000   -0.30000   -0.07720      21.4   9.30e-04    0.014000
## 1-Dodecanol; 36               0.220000    0.07880    0.36200      20.2   2.33e-03    0.029100
## 4-Hydroxybenzeneacetic ac    -0.581000   -0.97000   -0.19300      19.4   3.39e-03    0.036400
## Malic acid, 3TMS; 11         -0.229000   -0.39000   -0.06740      20.0   5.54e-03    0.052000
## Ribitol; 71                  -0.288000   -0.49600   -0.07940      23.3   6.86e-03    0.057200
## 3,4-Dihydroxybutanoic aci    -0.224000   -0.39700   -0.05240      15.9   1.06e-02    0.079800
## Decanoic acid; 52            -0.220000   -0.41000   -0.02930      22.4   2.38e-02    0.151000
## 2-Palmitoylglycerol; 39       0.145000    0.01910    0.27100      25.9   2.41e-02    0.151000
## Ethanolamine; 56             -0.149000   -0.28400   -0.01320      22.7   3.15e-02    0.182000
## Lactic acid; 29              -0.121000   -0.23200   -0.00892      25.4   3.43e-02    0.184000
## Myo inositol 6TMS; 1         -0.169000   -0.33600   -0.00132      19.0   4.82e-02    0.241000
## Tridecanoic acid; 74         -0.176000   -0.35600    0.00273      20.0   5.36e-02    0.251000
## Arachidonic acid, TMS; 24     0.123000   -0.00719    0.25300      22.7   6.40e-02    0.282000
## Citric acid, 4TMS; 6         -0.145000   -0.30200    0.01170      20.8   6.97e-02    0.286000
## Creatinine; 50                0.227000   -0.02130    0.47500      21.6   7.31e-02    0.286000
## Valine, 2TMS; 20              0.108000   -0.01250    0.22900      25.1   7.89e-02    0.286000
## Aminomalonic acid; 45        -0.170000   -0.36200    0.02230      24.3   8.31e-02    0.286000
## Arachidic acid; 46           -0.087500   -0.18700    0.01180      21.8   8.39e-02    0.286000
## Glyceric acid; 30             0.156000   -0.02350    0.33600      20.4   8.83e-02    0.288000
## 1,3-Propanediol; 34           0.136000   -0.02660    0.29800      24.2   1.01e-01    0.316000
## Fumaric acid, 2TMS; 9        -0.102000   -0.22600    0.02300      24.1   1.10e-01    0.329000
## 1-Monopalmitin; 37            0.107000   -0.02850    0.24200      29.2   1.21e-01    0.340000
## Serine, 3TMS; 14             -0.096300   -0.21800    0.02590      26.0   1.22e-01    0.340000
## Glutamic acid, 3TMS; 8        0.134000   -0.03870    0.30700      23.0   1.28e-01    0.343000
## 4-Hydroxyphenyllactic aci     0.146000   -0.05390    0.34600      20.8   1.52e-01    0.393000
## Myristoleic acid; 65          0.239000   -0.09820    0.57600      19.9   1.65e-01    0.407000
## Leucine, 2TMS; 19             0.118000   -0.05000    0.28700      21.1   1.68e-01    0.407000
## Octanoic acid; 68            -0.076100   -0.19000    0.03830      24.2   1.92e-01    0.449000
## Isoleucine, 2TMS; 18          0.122000   -0.06580    0.31000      26.0   2.03e-01    0.461000
## Oleic acid, TMS; 3           -0.085100   -0.21800    0.04800      17.1   2.10e-01    0.463000
## Stearic acid, TMS; 2          0.038100   -0.02450    0.10100      17.4   2.33e-01    0.499000
## Benzeneacetic acid; 47       -0.189000   -0.50900    0.13100      20.3   2.47e-01    0.514000
## Cholesterol, TMS; 23          0.056600   -0.04880    0.16200      21.7   2.92e-01    0.592000
## Proline, 2TMS; 21            -0.087900   -0.25700    0.08130      20.8   3.08e-01    0.608000
## Nonadecanoic acid; 66         0.081700   -0.08040    0.24400      20.0   3.23e-01    0.614000
## 4-Deoxytetronic acid; 32      0.114000   -0.11500    0.34300      21.6   3.27e-01    0.614000
## Glycerol; 58                 -0.194000   -0.59300    0.20500      24.9   3.39e-01    0.620000
## 11-Eicosenoic acid; 35       -0.122000   -0.37500    0.13200      21.9   3.47e-01    0.620000
## 3-Indoleacetic acid; 40      -0.119000   -0.37500    0.13700      20.5   3.61e-01    0.630000
## Eicosapentaenoic acid; 55     0.127000   -0.15800    0.41200      23.0   3.81e-01    0.640000
## Pyruvic acid; 31             -0.136000   -0.44900    0.17600      19.5   3.92e-01    0.640000
## 3-Indolepropionic acid; 4     0.165000   -0.22300    0.55300      19.5   4.03e-01    0.640000
```

```
## Succinic acid, 2TMS; 7       -0.044800   -0.15000    0.06060       22.7   4.04e-01    0.640000
## 4-Deoxytetronic acid; 33     -0.136000   -0.46100    0.19000       22.2   4.13e-01    0.640000
## Bisphenol A; 48               0.071400   -0.10200    0.24500       21.2   4.18e-01    0.640000
## alpha-Tocopherol; 26         -0.087000   -0.31300    0.13900       18.8   4.49e-01    0.662000
## Hydroxylamine; 62             0.061200   -0.09800    0.22000       27.7   4.50e-01    0.662000
## 2-Hydroxybutyric acid, 2T    -0.075900   -0.31500    0.16300       20.7   5.32e-01    0.768000
## Hydroxyproline; 64            0.085500   -0.19500    0.36600       20.6   5.49e-01    0.771000
## Tartronic acid; 73            0.086200   -0.20300    0.37500       21.3   5.58e-01    0.771000
## L-5-Oxoproline; 63            0.033200   -0.08020    0.14600       27.5   5.66e-01    0.771000
## 2-hydroxy Isovaleric acid     0.112000   -0.28200    0.50500       22.2   5.77e-01    0.773000
## 4-Hydroxybutanoic acid; 4    -0.017300   -0.08420    0.04970       27.3   6.13e-01    0.806000
## Pyroglutamic acid; 69         0.049900   -0.21200    0.31200       22.9   7.08e-01    0.866000
## Docosahexaenoic acid; 53     -0.043400   -0.27500    0.18800       24.2   7.13e-01    0.866000
## Glyceryl-glycoside; 59       -0.041800   -0.27100    0.18700       20.8   7.20e-01    0.866000
## Tyrosine; 75                  0.069100   -0.31400    0.45300       23.3   7.24e-01    0.866000
## Glycerol; 57                  0.024900   -0.12100    0.17000       28.7   7.36e-01    0.866000
## Dodecanoic acid; 54           0.034300   -0.16700    0.23600       22.7   7.38e-01    0.866000
## Linoleic acid, TMS; 4        -0.018100   -0.12500    0.08840       25.8   7.39e-01    0.866000
## Ribitol; 70                  -0.023200   -0.22700    0.18100       20.2   8.23e-01    0.950000
## Alanine, 2TMS; 25             0.010900   -0.11100    0.13300       22.8   8.61e-01    0.961000
## Palmitic acid, TMS; 5        -0.006080   -0.08720    0.07500       21.4   8.83e-01    0.961000
## Heptadecanoic acid; 61        0.007980   -0.10400    0.12000       23.4   8.89e-01    0.961000
## Threonine, 3TMS; 12           0.010600   -0.15000    0.17200       28.6   8.97e-01    0.961000
## Methionine, 2TMS; 16         -0.010900   -0.20700    0.18500       20.3   9.13e-01    0.961000
## Phenylalanine, 2TMS; 13      -0.005680   -0.10900    0.09810       24.3   9.14e-01    0.961000
## Ribonic acid; 72             -0.012700   -0.30200    0.27700       20.0   9.31e-01    0.961000
## Heptadecanoic acid; 60       -0.006570   -0.16600    0.15300       21.8   9.36e-01    0.961000
## alpha-ketoglutaric acid,      0.003530   -0.28900    0.29600       20.3   9.81e-01    0.994000
## Nonanoic acid; 67            -0.000414   -0.12900    0.12900       24.4   9.95e-01    0.995000
```

1.1.2 Adjusted Model

```
## [1] 665  14

##      (Intercept) GroupT1D Micro GroupT1D Macro GroupHealthy Control   Age
## [1,]           1              0              0                    0 19.39
## [2,]           1              1              1                    1 85.23
##      Gender Hba1c_baseline      egfr CALSBP   bmi Smoking Statin
## [1,]      0            4.7  11.03376     91 16.98       0      0
## [2,]      1           15.0 167.62905    191 43.29       1      1
##      log_Blood_TGA Total_cholesterol
## [1,]     -2.643856               2.3
## [2,]      2.720278               9.2

##
##                                Overall
##   n                               665
##   Group (%)
##      T1D Control                  290 (43.6)
##      T1D Micro                    152 (22.9)
##      T1D Macro                    178 (26.8)
##      Healthy Control               45 ( 6.8)
##   Age (mean (sd))               54.07 (12.74)
##   Gender (mean (sd))             0.54 (0.50)
##   Hba1c_baseline (mean (sd))     7.88 (1.31)
##   egfr (mean (sd))              83.67 (27.64)
##   CALSBP (mean (sd))           131.45 (17.41)
##   bmi (mean (sd))               25.24 (4.00)
##   Smoking (mean (sd))            0.20 (0.40)
##   Statin (mean (sd))             0.56 (0.50)
##   log_Blood_TGA (mean (sd))      0.00 (0.69)
##   Total_cholesterol (mean (sd))  4.71 (0.87)
```

1.1.2.1 Table

```
##
##
## Table: GroupT1D Micro
##
## Name                          Effect      CI.L      CI.R   AveExpr    P.Value   adj.P.Val
## --------------------------  ---------  ---------  --------  --------  ---------  ----------
## 3,4-Dihydroxybutanoic aci     0.19200    0.08400    0.3000      15.9   0.000509      0.0382
## 4-Deoxytetronic acid; 32      0.22700    0.08050    0.3740      21.6   0.002450      0.0918
## Ribitol; 70                   0.19400    0.06270    0.3260      20.2   0.003880      0.0969
## Heptadecanoic acid; 60       -0.14400   -0.24700   -0.0402      21.8   0.006560      0.1230
## 2,4-Dihydroxybutanoic aci     0.14300    0.02540    0.2600      16.5   0.017200      0.2580
## Creatinine; 50                0.16000    0.00835    0.3110      21.6   0.038700      0.4830
## Dodecanoic acid; 54           0.11700   -0.01090    0.2440      22.7   0.072900      0.5770
## Threonine, 3TMS; 12          -0.09280   -0.19800    0.0120      28.6   0.082700      0.5770
## 4-Hydroxybenzeneacetic ac     0.21100   -0.02910    0.4520      19.4   0.084800      0.5770
## Myo inositol 6TMS; 1          0.08760   -0.01290    0.1880      19.0   0.087300      0.5770
## Ribitol; 71                   0.10800   -0.01690    0.2320      23.3   0.090200      0.5770
## 11-Eicosenoic acid; 35        0.14100   -0.02330    0.3060      21.9   0.092300      0.5770
## Glycine, 3TMS; 17             0.05610   -0.01370    0.1260      21.4   0.115000      0.6230
## Ribonic acid; 72              0.13800   -0.03800    0.3140      20.0   0.124000      0.6230
## Pyroglutamic acid; 69         0.13100   -0.03640    0.2990      22.9   0.125000      0.6230
## 4-Hydroxyphenyllactic aci     0.09400   -0.03400    0.2220      20.8   0.150000      0.6410
## Isoleucine, 2TMS; 18         -0.08190   -0.19700    0.0328      26.0   0.161000      0.6410
## Malic acid, 3TMS; 11          0.07440   -0.02980    0.1790      20.0   0.161000      0.6410
## Glutamic acid, 3TMS; 8        0.07470   -0.03120    0.1810      23.0   0.166000      0.6410
## 2-hydroxy Isovaleric acid    -0.17700   -0.43100    0.0767      22.2   0.171000      0.6410
## Valine, 2TMS; 20             -0.04850   -0.12200    0.0250      25.1   0.196000      0.6810
## Methionine, 2TMS; 16         -0.08170   -0.20700    0.0433      20.3   0.200000      0.6810
## alpha-ketoglutaric acid,      0.12000   -0.06910    0.3090      20.3   0.213000      0.6850
## Succinic acid, 2TMS; 7        0.04280   -0.02580    0.1110      22.7   0.221000      0.6850
## Fumaric acid, 2TMS; 9         0.04890   -0.03170    0.1290      24.1   0.234000      0.6850
## Tyrosine; 75                 -0.14800   -0.39600    0.1000      23.3   0.243000      0.6850
## Tridecanoic acid; 74          0.06700   -0.04910    0.1830      20.0   0.258000      0.6850
## Arachidic acid; 46           -0.03690   -0.10100    0.0274      21.8   0.260000      0.6850
## Lactic acid; 29               0.03970   -0.03190    0.1110      25.4   0.276000      0.6850
## Glycerol; 57                 -0.05130   -0.14600    0.0435      28.7   0.288000      0.6850
## Leucine, 2TMS; 19            -0.05580   -0.16100    0.0495      21.1   0.298000      0.6850
## Docosahexaenoic acid; 53     -0.07640   -0.22100    0.0683      24.2   0.300000      0.6850
## Nonanoic acid; 67            -0.04390   -0.12800    0.0406      24.4   0.308000      0.6850
## Arachidonic acid, TMS; 24    -0.04310   -0.12800    0.0415      22.7   0.317000      0.6850
## Alanine, 2TMS; 25             0.03950   -0.03850    0.1180      22.8   0.320000      0.6850
## Palmitic acid, TMS; 5        -0.02460   -0.07600    0.0268      21.4   0.348000      0.6930
## 2-Hydroxybutyric acid, 2T    -0.06960   -0.21600    0.0772      20.7   0.352000      0.6930
## 1-Monopalmitin; 37            0.04140   -0.04720    0.1300      29.2   0.359000      0.6930
## Decanoic acid; 52             0.05430   -0.06600    0.1750      22.4   0.376000      0.6930
## Heptadecanoic acid; 61       -0.03270   -0.10500    0.0399      23.4   0.377000      0.6930
## 4-Deoxytetronic acid; 33     -0.08650   -0.29000    0.1170      22.2   0.403000      0.6930
## Benzeneacetic acid; 47        0.08710   -0.11700    0.2920      20.3   0.403000      0.6930
## Glyceric acid; 30             0.04680   -0.06510    0.1590      20.4   0.412000      0.6930
## Proline, 2TMS; 21            -0.04320   -0.15000    0.0639      20.8   0.429000      0.6930
## Glycerol; 58                 -0.10200   -0.36400    0.1600      24.9   0.446000      0.6930
## Aminomalonic acid; 45         0.04660   -0.07510    0.1680      24.3   0.452000      0.6930
```

```
## Bisphenol A; 48            -0.04340   -0.15700    0.0705      21.2   0.455000      0.6930
## Linoleic acid, TMS; 4      -0.02590   -0.09440    0.0426      25.8   0.458000      0.6930
## Pyruvic acid; 31            0.07590   -0.12500    0.2770      19.5   0.459000      0.6930
## Stearic acid, TMS; 2       -0.01500   -0.05520    0.0251      17.4   0.462000      0.6930
## Hydroxyproline; 64          0.06500   -0.11700    0.2470      20.6   0.484000      0.7110
## Glyceryl-glycoside; 59      0.04690   -0.10100    0.1940      20.8   0.533000      0.7560
## 3-Hydroxybutyric acid, 2T  -0.05420   -0.22600    0.1170      21.6   0.535000      0.7560
## Tartronic acid; 73          0.05510   -0.12600    0.2360      21.3   0.551000      0.7580
## 4-Hydroxybutanoic acid; 4   0.01310   -0.03040    0.0566      27.3   0.556000      0.7580
## Campesterol; 49             0.04180   -0.10500    0.1890      21.8   0.576000      0.7710
## 1-Dodecanol; 36             0.02530   -0.06710    0.1180      20.2   0.591000      0.7710
## 3-Indolepropionic acid; 4   0.06740   -0.18200    0.3170      19.5   0.596000      0.7710
## Serine, 3TMS; 14           -0.02020   -0.09900    0.0586      26.0   0.614000      0.7770
## 1,3-Propanediol; 34         0.02620   -0.07990    0.1320      24.2   0.628000      0.7770
## L-5-Oxoproline; 63          0.01790   -0.05550    0.0913      27.5   0.632000      0.7770
## 3-Indoleacetic acid; 40     0.02960   -0.13300    0.1920      20.5   0.720000      0.8560
## 2-Palmitoylglycerol; 39     0.01450   -0.06740    0.0964      25.9   0.729000      0.8560
## Arabinopyranose; 51        -0.02840   -0.19000    0.1330      20.1   0.731000      0.8560
## Citric acid, 4TMS; 6        0.01510   -0.08270    0.1130      20.8   0.762000      0.8790
## Oleic acid, TMS; 3         -0.00980   -0.09480    0.0752      17.1   0.821000      0.8900
## Myristoleic acid; 65        0.02360   -0.19000    0.2370      19.9   0.828000      0.8900
## Ethanolamine; 56            0.00882   -0.07910    0.0967      22.7   0.844000      0.8900
## Octanoic acid; 68           0.00715   -0.06680    0.0811      24.2   0.850000      0.8900
## alpha-Tocopherol; 26       -0.01350   -0.15300    0.1260      18.8   0.850000      0.8900
## Phenylalanine, 2TMS; 13     0.00647   -0.06100    0.0739      24.3   0.851000      0.8900
## Cholesterol, TMS; 23       -0.00525   -0.06710    0.0566      21.7   0.868000      0.8900
## Hydroxylamine; 62          -0.00862   -0.11200    0.0949      27.7   0.870000      0.8900
## Eicosapentaenoic acid; 55  -0.01370   -0.18900    0.1620      23.0   0.878000      0.8900
## Nonadecanoic acid; 66       0.00189   -0.10300    0.1070      20.0   0.972000      0.9720
##
##
## Table: GroupT1D Macro
##
## Name                           Effect        CI.L        CI.R   AveExpr    P.Value   adj.P.Val
## -------------------------  ----------  ----------  ---------  --------  ---------  ----------
## 3,4-Dihydroxybutanoic aci    0.311000    0.192000    0.43000      15.9   4.00e-07    2.91e-05
## Ribonic acid; 72             0.419000    0.225000    0.61400      20.0   2.61e-05    9.78e-04
## 2,4-Dihydroxybutanoic aci    0.243000    0.114000    0.37200      16.5   2.46e-04    6.14e-03
## Myo inositol 6TMS; 1         0.194000    0.082900    0.30500      19.0   6.38e-04    1.20e-02
## 4-Hydroxybenzeneacetic ac    0.413000    0.148000    0.67900      19.4   2.33e-03    3.49e-02
## 4-Deoxytetronic acid; 32     0.219000    0.056900    0.38100      21.6   8.16e-03    1.02e-01
## Valine, 2TMS; 20            -0.106000   -0.187000   -0.02450      25.1   1.08e-02    1.16e-01
## Threonine, 3TMS; 12         -0.137000   -0.253000   -0.02180      28.6   1.99e-02    1.86e-01
## Methionine, 2TMS; 16        -0.156000   -0.294000   -0.01790      20.3   2.68e-02    2.24e-01
## Docosahexaenoic acid; 53    -0.169000   -0.328000   -0.00891      24.2   3.85e-02    2.89e-01
## 3-Indolepropionic acid; 4   -0.282000   -0.558000   -0.00632      19.5   4.50e-02    3.07e-01
## Ribitol; 70                  0.146000    0.000343    0.29100      20.2   4.95e-02    3.09e-01
## Tyrosine; 75                -0.268000   -0.542000    0.00597      23.3   5.52e-02    3.18e-01
## 4-Hydroxybutanoic acid; 4    0.045000   -0.002970    0.09310      27.3   6.59e-02    3.53e-01
## Hydroxyproline; 64           0.183000   -0.018200    0.38400      20.6   7.45e-02    3.73e-01
## 2-hydroxy Isovaleric acid   -0.241000   -0.521000    0.03930      22.2   9.18e-02    4.18e-01
## Proline, 2TMS; 21            0.101000   -0.017500    0.21900      20.8   9.50e-02    4.18e-01
## Aminomalonic acid; 45       -0.112000   -0.247000    0.02190      24.3   1.01e-01    4.18e-01
## Nonadecanoic acid; 66       -0.095900   -0.212000    0.02040      20.0   1.06e-01    4.18e-01
```

```
## Heptadecanoic acid; 60      -0.091400   -0.206000    0.02270      21.8   1.16e-01    4.36e-01
## 4-Deoxytetronic acid; 33     0.176000   -0.048600    0.40000      22.2   1.25e-01    4.45e-01
## Eicosapentaenoic acid; 55   -0.148000   -0.342000    0.04510      23.0   1.33e-01    4.52e-01
## Octanoic acid; 68           -0.060800   -0.143000    0.02080      24.2   1.44e-01    4.69e-01
## 1-Dodecanol; 36             -0.073300   -0.175000    0.02870      20.2   1.59e-01    4.82e-01
## Phenylalanine, 2TMS; 13     -0.052700   -0.127000    0.02170      24.3   1.65e-01    4.82e-01
## Ribitol; 71                  0.096100   -0.041400    0.23300      23.3   1.70e-01    4.82e-01
## Glyceryl-glycoside; 59       0.113000   -0.049800    0.27600      20.8   1.73e-01    4.82e-01
## 11-Eicosenoic acid; 35       0.109000   -0.072900    0.29000      21.9   2.40e-01    6.44e-01
## Malic acid, 3TMS; 11         0.061600   -0.053400    0.17700      20.0   2.93e-01    7.10e-01
## Stearic acid, TMS; 2         0.023300   -0.021000    0.06770      17.4   3.02e-01    7.10e-01
## Cholesterol, TMS; 23        -0.034600   -0.103000    0.03360      21.7   3.20e-01    7.10e-01
## 4-Hydroxyphenyllactic aci    0.071400   -0.069900    0.21300      20.8   3.22e-01    7.10e-01
## 2-Palmitoylglycerol; 39      0.045100   -0.045300    0.13600      25.9   3.27e-01    7.10e-01
## Ethanolamine; 56            -0.048400   -0.145000    0.04870      22.7   3.28e-01    7.10e-01
## L-5-Oxoproline; 63          -0.040100   -0.121000    0.04090      27.5   3.31e-01    7.10e-01
## Arabinopyranose; 51         -0.083000   -0.262000    0.09570      20.1   3.62e-01    7.16e-01
## Fumaric acid, 2TMS; 9        0.040800   -0.048100    0.13000      24.1   3.68e-01    7.16e-01
## alpha-ketoglutaric acid,     0.095600   -0.113000    0.30400      20.3   3.69e-01    7.16e-01
## Tridecanoic acid; 74        -0.057100   -0.185000    0.07100      20.0   3.82e-01    7.16e-01
## Glutamic acid, 3TMS; 8       0.052100   -0.064800    0.16900      23.0   3.82e-01    7.16e-01
## Succinic acid, 2TMS; 7       0.032600   -0.043100    0.10800      22.7   3.98e-01    7.28e-01
## Lactic acid; 29              0.033200   -0.045800    0.11200      25.4   4.10e-01    7.32e-01
## Isoleucine, 2TMS; 18        -0.051800   -0.178000    0.07470      26.0   4.22e-01    7.33e-01
## Campesterol; 49             -0.063700   -0.226000    0.09850      21.8   4.41e-01    7.33e-01
## Alanine, 2TMS; 25           -0.033000   -0.119000    0.05310      22.8   4.52e-01    7.33e-01
## Citric acid, 4TMS; 6         0.040700   -0.067200    0.14900      20.8   4.59e-01    7.33e-01
## Arachidic acid; 46          -0.026800   -0.097800    0.04420      21.8   4.59e-01    7.33e-01
## Decanoic acid; 52            0.046800   -0.086100    0.18000      22.4   4.90e-01    7.44e-01
## Linoleic acid, TMS; 4       -0.026200   -0.102000    0.04940      25.8   4.96e-01    7.44e-01
## Glycerol; 58                 0.100000   -0.189000    0.39000      24.9   4.96e-01    7.44e-01
## alpha-Tocopherol; 26        -0.051800   -0.206000    0.10200      18.8   5.10e-01    7.44e-01
## 3-Hydroxybutyric acid, 2T    0.062600   -0.127000    0.25200      21.6   5.16e-01    7.44e-01
## Benzeneacetic acid; 47       0.068300   -0.157000    0.29400      20.3   5.53e-01    7.82e-01
## Serine, 3TMS; 14            -0.024300   -0.111000    0.06260      26.0   5.83e-01    7.94e-01
## Tartronic acid; 73           0.054900   -0.145000    0.25500      21.3   5.90e-01    7.94e-01
## Pyruvic acid; 31             0.060400   -0.161000    0.28200      19.5   5.93e-01    7.94e-01
## Dodecanoic acid; 54          0.035800   -0.105000    0.17700      22.7   6.17e-01    8.12e-01
## Oleic acid, TMS; 3           0.021600   -0.072200    0.11500      17.1   6.52e-01    8.30e-01
## 3-Indoleacetic acid; 40      0.041100   -0.138000    0.22000      20.5   6.53e-01    8.30e-01
## 1,3-Propanediol; 34          0.020800   -0.096200    0.13800      24.2   7.27e-01    8.88e-01
## Palmitic acid, TMS; 5        0.009820   -0.046900    0.06650      21.4   7.34e-01    8.88e-01
## Heptadecanoic acid; 61      -0.013400   -0.093600    0.06680      23.4   7.43e-01    8.88e-01
## Leucine, 2TMS; 19           -0.018100   -0.134000    0.09810      21.1   7.60e-01    8.88e-01
## Myristoleic acid; 65         0.036500   -0.199000    0.27200      19.9   7.61e-01    8.88e-01
## Nonanoic acid; 67            0.013900   -0.079300    0.10700      24.4   7.69e-01    8.88e-01
## 1-Monopalmitin; 37          -0.013500   -0.111000    0.08430      29.2   7.86e-01    8.94e-01
## 2-Hydroxybutyric acid, 2T   -0.021000   -0.183000    0.14100      20.7   7.99e-01    8.94e-01
## Creatinine; 50               0.019100   -0.148000    0.18600      21.6   8.22e-01    9.07e-01
## Pyroglutamic acid; 69        0.017400   -0.168000    0.20200      22.9   8.53e-01    9.16e-01
## Hydroxylamine; 62           -0.010600   -0.125000    0.10400      27.7   8.55e-01    9.16e-01
## Bisphenol A; 48             -0.009890   -0.136000    0.11600      21.2   8.77e-01    9.20e-01
## Glycerol; 57                -0.007810   -0.112000    0.09680      28.7   8.84e-01    9.20e-01
## Arachidonic acid, TMS; 24   -0.001560   -0.094900    0.09180      22.7   9.74e-01    9.98e-01
```

```
## Glycine, 3TMS; 17           -0.000363   -0.077400    0.07670      21.4   9.93e-01    9.98e-01
## Glyceric acid; 30             0.000147   -0.123000    0.12400      20.4   9.98e-01    9.98e-01
##
##
## Table: GroupHealthy Control
##
## Name                           Effect        CI.L        CI.R   AveExpr    P.Value   adj.P.Val
## --------------------------  ---------   ---------  ----------  --------  ---------  ----------
## Tridecanoic acid; 74         -0.36700    -0.57800   -0.156000      20.0   0.000674      0.0268
## 2,4-Dihydroxybutanoic aci    -0.36800    -0.58100   -0.156000      16.5   0.000715      0.0268
## Decanoic acid; 52            -0.36200    -0.58000   -0.143000      22.4   0.001200      0.0301
## 3-Hydroxybutyric acid, 2T    -0.41300    -0.72400   -0.102000      21.6   0.009330      0.1370
## Campesterol; 49              -0.35200    -0.61900   -0.085100      21.8   0.009800      0.1370
## Valine, 2TMS; 20              0.17400     0.04000    0.307000      25.1   0.011000      0.1370
## 2-Palmitoylglycerol; 39       0.16800     0.01900    0.316000      25.9   0.027100      0.2900
## Arachidic acid; 46           -0.12500    -0.24200   -0.008630      21.8   0.035300      0.3210
## Octanoic acid; 68            -0.14000    -0.27400   -0.005670      24.2   0.041100      0.3210
## 1-Dodecanol; 36               0.17200     0.00461    0.340000      20.2   0.044100      0.3210
## 11-Eicosenoic acid; 35       -0.30200    -0.60100   -0.003160      21.9   0.047600      0.3210
## Glycine, 3TMS; 17            -0.12600    -0.25300    0.000744      21.4   0.051400      0.3210
## Isoleucine, 2TMS; 18          0.20200    -0.00661    0.410000      26.0   0.057700      0.3330
## Malic acid, 3TMS; 11         -0.17700    -0.36600    0.012300      20.0   0.066800      0.3540
## 4-Deoxytetronic acid; 33     -0.33700    -0.70500    0.031900      22.2   0.073300      0.3540
## 4-Hydroxybenzeneacetic ac    -0.38100    -0.81800    0.055300      19.4   0.086800      0.3540
## Alanine, 2TMS; 25             0.12200    -0.01980    0.263000      22.8   0.091800      0.3540
## Arachidonic acid, TMS; 24     0.13000    -0.02330    0.284000      22.7   0.096300      0.3540
## Leucine, 2TMS; 19             0.16200    -0.02920    0.353000      21.1   0.096700      0.3540
## Oleic acid, TMS; 3           -0.13100    -0.28500    0.023700      17.1   0.097100      0.3540
## 4-Hydroxybutanoic acid; 4    -0.06640    -0.14500    0.012500      27.3   0.099100      0.3540
## 3,4-Dihydroxybutanoic aci    -0.16000    -0.35600    0.035600      15.9   0.109000      0.3700
## Arabinopyranose; 51          -0.23700    -0.53100    0.057100      20.1   0.114000      0.3720
## Ribitol; 71                  -0.17700    -0.40300    0.048800      23.3   0.124000      0.3880
## Tyrosine; 75                  0.34600    -0.10500    0.796000      23.3   0.132000      0.3970
## Docosahexaenoic acid; 53     -0.19400    -0.45700    0.068300      24.2   0.147000      0.4050
## Fumaric acid, 2TMS; 9        -0.10700    -0.25400    0.039000      24.1   0.150000      0.4050
## Creatinine; 50                0.20100    -0.07370    0.476000      21.6   0.151000      0.4050
## Glutamic acid, 3TMS; 8        0.12700    -0.06500    0.319000      23.0   0.194000      0.4960
## alpha-Tocopherol; 26         -0.16600    -0.42000    0.088000      18.8   0.200000      0.4960
## Aminomalonic acid; 45        -0.14100    -0.36200    0.080200      24.3   0.211000      0.4960
## Linoleic acid, TMS; 4        -0.07750    -0.20200    0.046900      25.8   0.222000      0.4960
## Glycerol; 57                  0.10600    -0.06570    0.278000      28.7   0.225000      0.4960
## Nonadecanoic acid; 66         0.11700    -0.07420    0.308000      20.0   0.230000      0.4960
## Myo inositol 6TMS; 1         -0.11000    -0.29300    0.072000      19.0   0.235000      0.4960
## Lactic acid; 29              -0.07810    -0.20800    0.051800      25.4   0.238000      0.4960
## Hydroxylamine; 62             0.10700    -0.08080    0.295000      27.7   0.264000      0.5340
## Succinic acid, 2TMS; 7       -0.05720    -0.18200    0.067300      22.7   0.368000      0.7250
## Pyruvic acid; 31             -0.15700    -0.52200    0.208000      19.5   0.399000      0.7680
## Serine, 3TMS; 14             -0.05630    -0.19900    0.086800      26.0   0.440000      0.8250
## Citric acid, 4TMS; 6         -0.06540    -0.24300    0.112000      20.8   0.470000      0.8460
## Ethanolamine; 56             -0.05450    -0.21400    0.105000      22.7   0.503000      0.8460
## Ribitol; 70                  -0.08080    -0.32000    0.158000      20.2   0.507000      0.8460
## 1-Monopalmitin; 37            0.05420    -0.10700    0.215000      29.2   0.509000      0.8460
## Myristoleic acid; 65          0.12200    -0.26500    0.509000      19.9   0.536000      0.8460
## Heptadecanoic acid; 61       -0.04080    -0.17300    0.091100      23.4   0.544000      0.8460
```

```
## Phenylalanine, 2TMS; 13        0.03780   -0.08460   0.160000      24.3   0.544000      0.8460
## 1,3-Propanediol; 34            0.05940   -0.13300   0.252000      24.2   0.544000      0.8460
## Bisphenol A; 48                0.06250   -0.14400   0.269000      21.2   0.553000      0.8460
## Stearic acid, TMS; 2           0.02090   -0.05200   0.093800      17.4   0.574000      0.8520
## 3-Indoleacetic acid; 40       -0.08340   -0.37800   0.212000      20.5   0.579000      0.8520
## Glyceryl-glycoside; 59         0.06900   -0.19900   0.337000      20.8   0.613000      0.8840
## Benzeneacetic acid; 47        -0.09170   -0.46300   0.280000      20.3   0.628000      0.8890
## Nonanoic acid; 67             -0.03490   -0.18800   0.118000      24.4   0.655000      0.8920
## Pyroglutamic acid; 69          0.06730   -0.23700   0.372000      22.9   0.664000      0.8920
## 4-Hydroxyphenyllactic aci      0.05090   -0.18200   0.283000      20.8   0.668000      0.8920
## 4-Deoxytetronic acid; 32      -0.05540   -0.32200   0.211000      21.6   0.683000      0.8920
## Cholesterol, TMS; 23          -0.02290   -0.13500   0.089400      21.7   0.689000      0.8920
## alpha-ketoglutaric acid,      -0.06400   -0.40800   0.280000      20.3   0.715000      0.9080
## Tartronic acid; 73            -0.04910   -0.37800   0.280000      21.3   0.770000      0.9420
## Glyceric acid; 30              0.02960   -0.17400   0.233000      20.4   0.775000      0.9420
## Palmitic acid, TMS; 5         -0.01290   -0.10600   0.080400      21.4   0.787000      0.9420
## Heptadecanoic acid; 60         0.02410   -0.16400   0.212000      21.8   0.801000      0.9420
## Glycerol; 58                  -0.05620   -0.53200   0.420000      24.9   0.817000      0.9420
## Hydroxyproline; 64             0.03740   -0.29300   0.368000      20.6   0.824000      0.9420
## Proline, 2TMS; 21              0.02140   -0.17300   0.216000      20.8   0.829000      0.9420
## L-5-Oxoproline; 63             0.01070   -0.12300   0.144000      27.5   0.874000      0.9500
## Threonine, 3TMS; 12           -0.01500   -0.20500   0.175000      28.6   0.877000      0.9500
## Methionine, 2TMS; 16           0.01740   -0.21000   0.244000      20.3   0.880000      0.9500
## Ribonic acid; 72              -0.01980   -0.34000   0.300000      20.0   0.903000      0.9500
## Dodecanoic acid; 54            0.01370   -0.21800   0.245000      22.7   0.907000      0.9500
## 2-Hydroxybutyric acid, 2T      0.01460   -0.25200   0.281000      20.7   0.915000      0.9500
## Eicosapentaenoic acid; 55     -0.01540   -0.33400   0.303000      23.0   0.924000      0.9500
## 2-hydroxy Isovaleric acid     -0.00527   -0.46600   0.456000      22.2   0.982000      0.9910
## 3-Indolepropionic acid; 4      0.00270   -0.45100   0.456000      19.5   0.991000      0.9910
```

1.1.2.2 Figure

1.1.2.2.1 Heatmap of Model Coefficients

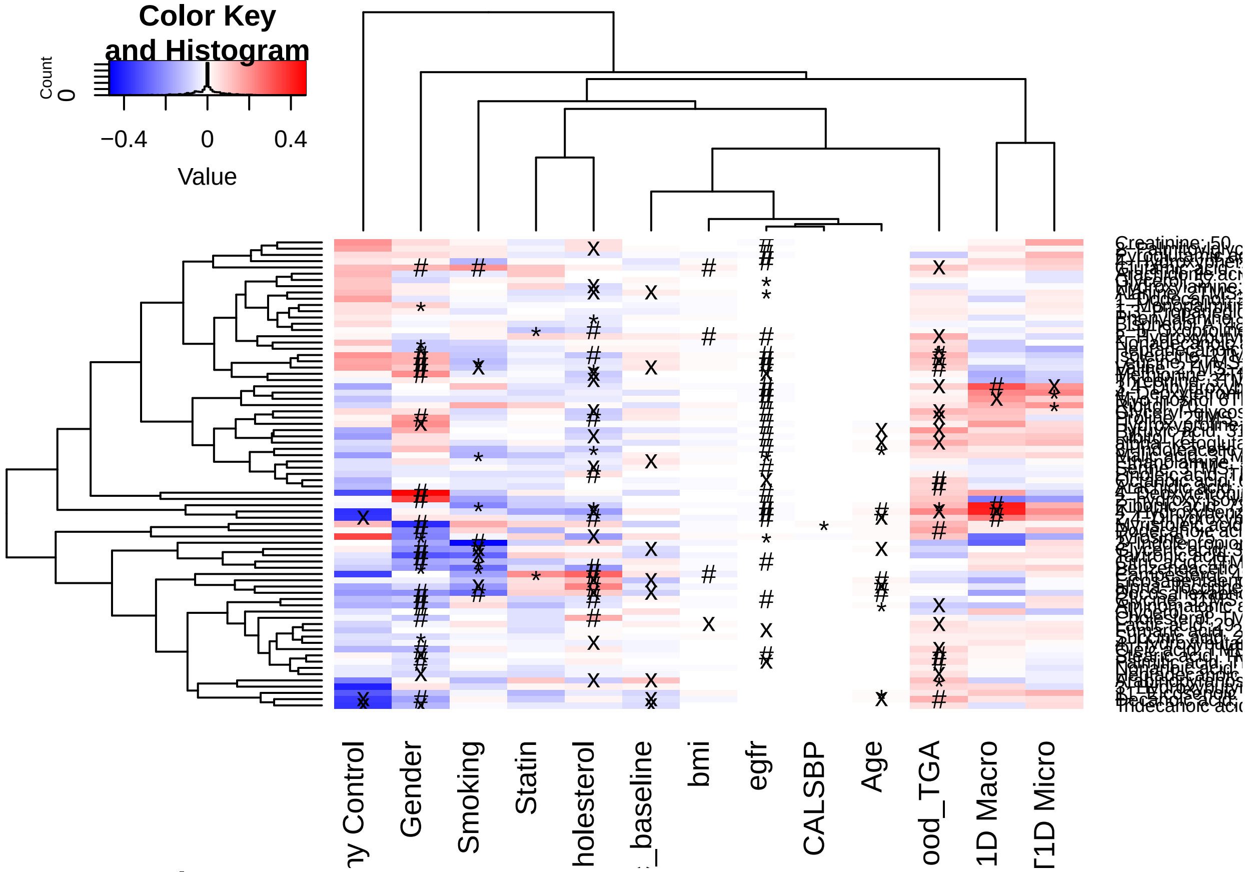
Color Key
and Histogram
Count
−0.4
0
0.4
Value
Gender
Smoking
Statin
holesterol
bmi
egfr
CALSBP
Age
ood_TGA
1D Macro
Γ1D Micro
Creatinine; 50
Undecanoic acid

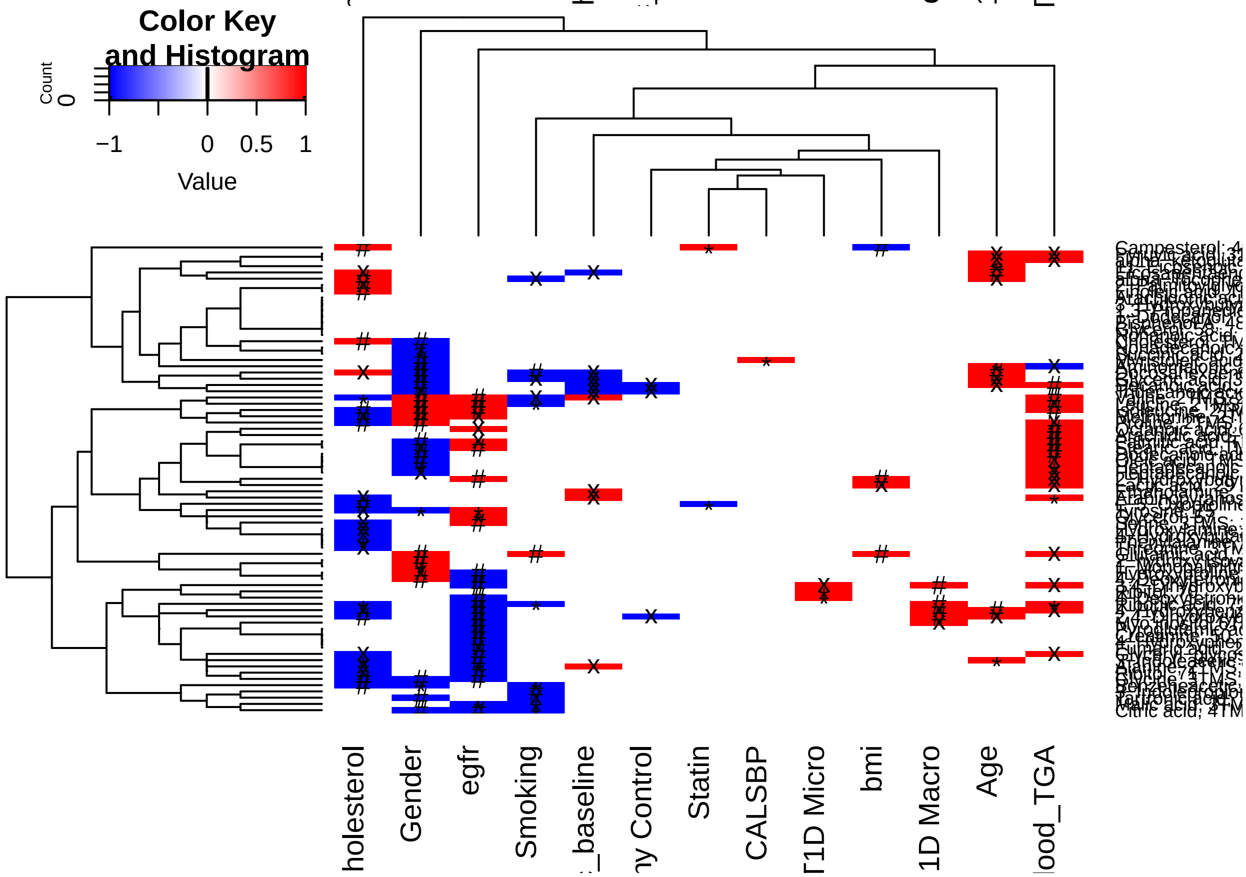
Color Key
and Histogram
Count
−1
0
0.5
1
Value
holesterol
Gender
egfr
Smoking
baseline
Statin
CALSBP
Γ1D Micro
bmi
1D Macro
Age
lood_TGA
Campesterol; 4
Citric acid; 4TM

1.1.2.2.2 Bipartite Graph of Model Coefficients

```
## Warning: Removed 2 rows containing missing values (geom_segment).
## Warning: Removed 1 rows containing missing values (geom_text).
```

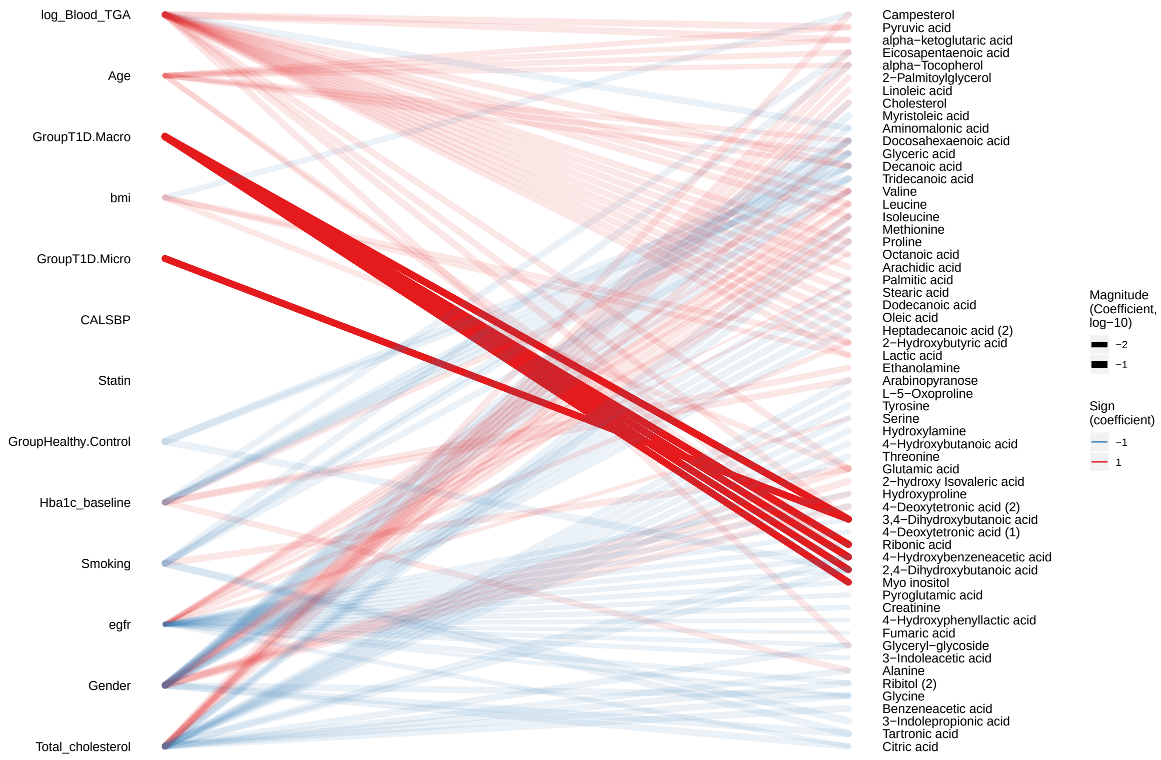


```
## Warning: Removed 2 rows containing missing values (geom_segment).

## Warning: Removed 1 rows containing missing values (geom_text).
```

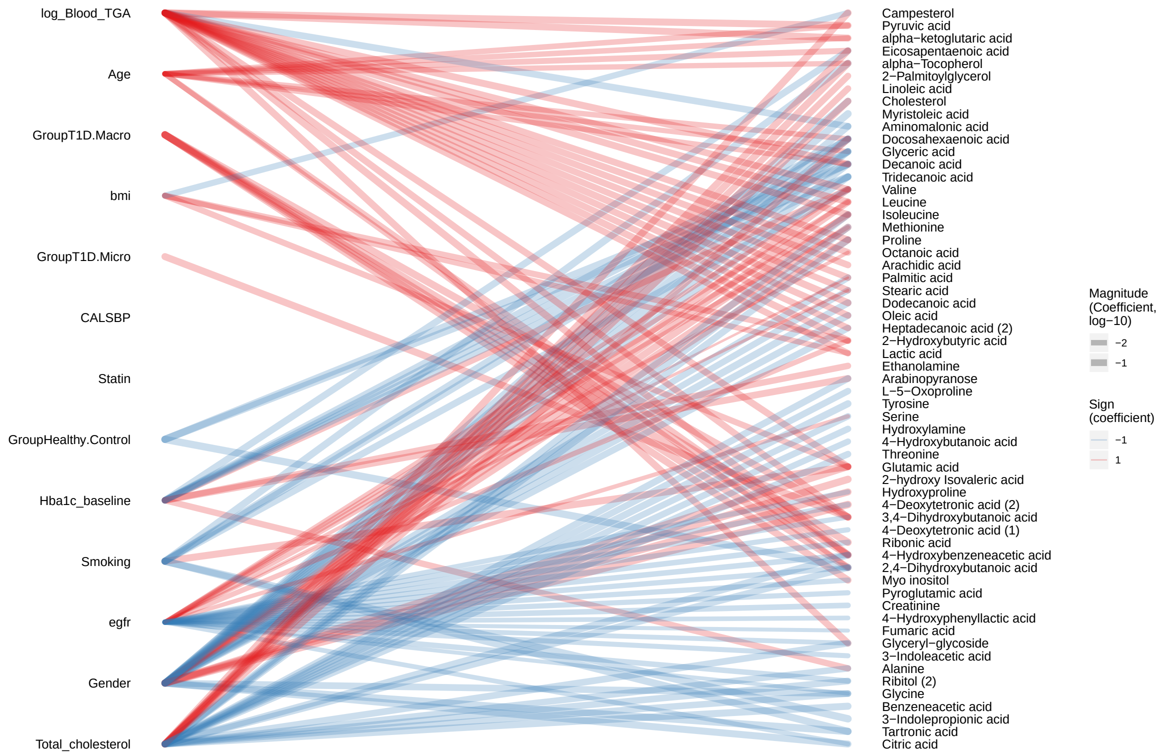

log_Blood_TGA
Age
GroupT1D.Macro
bmi
GroupT1D.Micro
CALSBP
Statin
GroupHealthy.Control
Hba1c_baseline
Smoking
egfr
Gender
Total_cholesterol
Campesterol
Pyruvic acid
alpha−ketoglutaric acid
Eicosapentaenoic acid
alpha−Tocopherol
2−Palmitoylglycerol
Linoleic acid
Cholesterol
Myristoleic acid
Aminomalonic acid
Docosahexaenoic acid
Glyceric acid
Decanoic acid
Tridecanoic acid
Valine
Leucine
Isoleucine
Methionine
Proline
Octanoic acid
Arachidic acid
Palmitic acid
Stearic acid
Dodecanoic acid
Oleic acid
Heptadecanoic acid (2)
2−Hydroxybutyric acid
Lactic acid
Ethanolamine
Arabinopyranose
L−5−Oxoproline
Tyrosine
Serine
Hydroxylamine
4−Hydroxybutanoic acid
Threonine
Glutamic acid
2−hydroxy Isovaleric acid
Hydroxyproline
4−Deoxytetronic acid (2)
3,4−Dihydroxybutanoic acid
4−Deoxytetronic acid (1)
Ribonic acid
4−Hydroxybenzeneacetic acid
2,4−Dihydroxybutanoic acid
Myo inositol
Pyroglutamic acid
Creatinine
4−Hydroxyphenyllactic acid
Fumaric acid
Glyceryl−glycoside
3−Indoleacetic acid
Alanine
Ribitol (2)
Glycine
Benzeneacetic acid
3−Indolepropionic acid
Tartronic acid
Citric acid
Magnitude
(Coefficient,
log−10)
−2
−1
Sign
(coefficient)
−1
1

1.2 eGFR

1.2.1 Crude Model

```
## [1] 586   2
```

```
##      (Intercept)      egfr
## [1,]           1  11.03376
## [2,]           1 167.62905
```

```
##
##                                  Overall
##   n                                586
##   egfr (mean (sd))               82.04 (28.24)
##   Age (mean (sd))                55.37 (12.11)
##   Gender (mean (sd))              0.54 (0.50)
##   Hba1c_baseline (mean (sd))      8.03 (1.16)
##   logUAER (mean (sd))             4.73 (2.31)
##   CALSBP (mean (sd))            131.91 (17.48)
##   bmi (mean (sd))                25.20 (4.06)
##   Smoking (mean (sd))             0.21 (0.41)
##   Statin (mean (sd))              0.61 (0.49)
##   log_Blood_TGA (mean (sd))       0.01 (0.69)
##   Total_cholesterol (mean (sd))   4.69 (0.88)
```

1.2.1.1 Table

```
##
##
## Table: egfr
##
## Name                            Effect        CI.L        CI.R   AveExpr    P.Value    adj.P.Val
## -------------------------  ----------  ----------  ----------  --------  ---------  ----------
## Myo inositol 6TMS; 1         -1.03e-02   -1.17e-02   -0.008900      19.1   0.00e+00    0.00e+00
## Ribitol; 71                  -1.22e-02   -1.39e-02   -0.010400      23.3   0.00e+00    0.00e+00
## 2,4-Dihydroxybutanoic aci    -1.03e-02   -1.19e-02   -0.008710      16.6   0.00e+00    0.00e+00
## Ribonic acid; 72             -1.63e-02   -1.89e-02   -0.013800      20.0   0.00e+00    0.00e+00
## Creatinine; 50               -1.21e-02   -1.42e-02   -0.009940      21.6   0.00e+00    0.00e+00
## 3,4-Dihydroxybutanoic aci    -8.51e-03   -1.01e-02   -0.006940      15.9   0.00e+00    0.00e+00
## 4-Hydroxybenzeneacetic ac    -1.70e-02   -2.04e-02   -0.013500      19.5   0.00e+00    0.00e+00
## 4-Deoxytetronic acid; 33     -1.14e-02   -1.44e-02   -0.008460      22.2   0.00e+00    0.00e+00
## 4-Deoxytetronic acid; 32     -7.41e-03   -9.39e-03   -0.005430      21.6   0.00e+00    0.00e+00
## Isoleucine, 2TMS; 18          5.61e-03    3.91e-03    0.007310      26.0   0.00e+00    0.00e+00
## Valine, 2TMS; 20              3.55e-03    2.47e-03    0.004640      25.1   0.00e+00    0.00e+00
## Citric acid, 4TMS; 6         -4.37e-03   -5.75e-03   -0.003000      20.8   0.00e+00    0.00e+00
## 3-Indoleacetic acid; 40      -7.15e-03   -9.41e-03   -0.004880      20.5   0.00e+00    0.00e+00
## 4-Hydroxyphenyllactic aci    -5.43e-03   -7.20e-03   -0.003660      20.8   0.00e+00    0.00e+00
## Pyroglutamic acid; 69        -6.73e-03   -9.08e-03   -0.004390      22.9   0.00e+00    1.00e-07
## Glyceryl-glycoside; 59       -5.30e-03   -7.32e-03   -0.003280      20.8   4.00e-07    1.70e-06
## 2-Hydroxybutyric acid, 2T     5.19e-03    3.03e-03    0.007350      20.7   2.90e-06    1.29e-05
## Serine, 3TMS; 14              2.61e-03    1.50e-03    0.003730      26.0   5.20e-06    2.18e-05
## Fumaric acid, 2TMS; 9        -2.62e-03   -3.75e-03   -0.001480      24.1   6.80e-06    2.70e-05
## Leucine, 2TMS; 19             3.50e-03    1.96e-03    0.005040      21.1   9.90e-06    3.72e-05
## Hydroxyproline; 64           -5.59e-03   -8.14e-03   -0.003040      20.6   2.01e-05    7.17e-05
## Methionine, 2TMS; 16          3.73e-03    1.92e-03    0.005540      20.3   5.80e-05    1.98e-04
## 2-hydroxy Isovaleric acid     7.02e-03    3.44e-03    0.010600      22.2   1.32e-04    4.30e-04
## Malic acid, 3TMS; 11         -2.83e-03   -4.29e-03   -0.001380      20.0   1.45e-04    4.53e-04
## Glycine, 3TMS; 17            -1.81e-03   -2.81e-03   -0.000802      21.4   4.48e-04    1.34e-03
## Benzeneacetic acid; 47       -4.90e-03   -7.88e-03   -0.001930      20.4   1.27e-03    3.68e-03
## Octanoic acid; 68             1.60e-03    5.49e-04    0.002660      24.2   2.95e-03    8.20e-03
## Succinic acid, 2TMS; 7       -1.40e-03   -2.36e-03   -0.000436      22.7   4.47e-03    1.20e-02
## Ribitol; 70                  -2.72e-03   -4.63e-03   -0.000810      20.2   5.30e-03    1.37e-02
## Phenylalanine, 2TMS; 13      -1.19e-03   -2.15e-03   -0.000238      24.3   1.44e-02    3.60e-02
## Alanine, 2TMS; 25            -1.33e-03   -2.42e-03   -0.000248      22.8   1.62e-02    3.91e-02
## Cholesterol, TMS; 23          1.19e-03    2.16e-04    0.002160      21.6   1.67e-02    3.92e-02
## Tyrosine; 75                  4.01e-03    4.53e-04    0.007570      23.3   2.72e-02    6.04e-02
## Glycerol; 58                 -4.02e-03   -7.59e-03   -0.000450      24.9   2.74e-02    6.04e-02
## 2-Palmitoylglycerol; 39       1.27e-03    9.20e-05    0.002440      25.9   3.46e-02    7.41e-02
## Threonine, 3TMS; 12           1.60e-03    9.68e-05    0.003100      28.6   3.70e-02    7.70e-02
## 3-Indolepropionic acid; 4     3.60e-03   -1.22e-04    0.007310      19.5   5.80e-02    1.18e-01
## Stearic acid, TMS; 2          5.45e-04   -4.16e-05    0.001130      17.4   6.85e-02    1.35e-01
## 11-Eicosenoic acid; 35       -2.05e-03   -4.29e-03    0.000179      21.9   7.13e-02    1.37e-01
## Myristoleic acid; 65         -2.30e-03   -5.44e-03    0.000829      19.9   1.49e-01    2.80e-01
## alpha-ketoglutaric acid,     -1.85e-03   -4.56e-03    0.000863      20.3   1.81e-01    3.30e-01
## Glycerol; 57                  9.24e-04   -4.44e-04    0.002290      28.7   1.85e-01    3.30e-01
## Aminomalonic acid; 45        -1.16e-03   -2.96e-03    0.000634      24.3   2.04e-01    3.57e-01
## Bisphenol A; 48               9.94e-04   -5.82e-04    0.002570      21.2   2.16e-01    3.63e-01
## Tartronic acid; 73           -1.63e-03   -4.22e-03    0.000962      21.3   2.18e-01    3.63e-01
## Heptadecanoic acid; 60       -8.89e-04   -2.33e-03    0.000555      21.8   2.27e-01    3.70e-01
```

```
## Ethanolamine; 56             7.27e-04   -5.15e-04   0.001970      22.7   2.51e-01    4.00e-01
## L-5-Oxoproline; 63           6.10e-04   -4.56e-04   0.001680      27.5   2.62e-01    4.09e-01
## Palmitic acid, TMS; 5        4.09e-04   -3.44e-04   0.001160      21.4   2.86e-01    4.38e-01
## Nonadecanoic acid; 66       -7.27e-04   -2.21e-03   0.000761      20.0   3.38e-01    5.03e-01
## 1-Dodecanol; 36              6.43e-04   -6.85e-04   0.001970      20.2   3.42e-01    5.03e-01
## Heptadecanoic acid; 61      -4.82e-04   -1.51e-03   0.000546      23.4   3.58e-01    5.07e-01
## Campesterol; 49             -1.02e-03   -3.21e-03   0.001180      21.8   3.63e-01    5.07e-01
## Oleic acid, TMS; 3          -5.66e-04   -1.79e-03   0.000661      17.1   3.65e-01    5.07e-01
## Dodecanoic acid; 54         -8.26e-04   -2.67e-03   0.001020      22.7   3.79e-01    5.16e-01
## Eicosapentaenoic acid; 55    1.13e-03   -1.44e-03   0.003710      23.0   3.88e-01    5.19e-01
## 4-Hydroxybutanoic acid; 4   -2.55e-04   -8.51e-04   0.000341      27.3   4.01e-01    5.27e-01
## Linoleic acid, TMS; 4        4.24e-04   -5.81e-04   0.001430      25.7   4.07e-01    5.27e-01
## Glyceric acid; 30            6.08e-04   -1.05e-03   0.002270      20.4   4.73e-01    6.01e-01
## Decanoic acid; 52           -4.86e-04   -2.23e-03   0.001260      22.4   5.85e-01    7.25e-01
## Lactic acid; 29              2.86e-04   -7.55e-04   0.001330      25.4   5.89e-01    7.25e-01
## 1-Monopalmitin; 37           3.44e-04   -9.46e-04   0.001630      29.2   6.01e-01    7.27e-01
## Pyruvic acid; 31            -6.51e-04   -3.42e-03   0.002120      19.6   6.44e-01    7.61e-01
## Hydroxylamine; 62           -3.44e-04   -1.83e-03   0.001140      27.7   6.50e-01    7.61e-01
## Proline, 2TMS; 21           -3.16e-04   -1.88e-03   0.001250      20.8   6.91e-01    7.97e-01
## 3-Hydroxybutyric acid, 2T   -4.63e-04   -2.91e-03   0.001980      21.7   7.10e-01    8.07e-01
## Arabinopyranose; 51         -3.86e-04   -2.74e-03   0.001960      20.2   7.47e-01    8.31e-01
## Nonanoic acid; 67           -1.90e-04   -1.38e-03   0.000999      24.4   7.54e-01    8.31e-01
## Arachidonic acid, TMS; 24   -1.70e-04   -1.39e-03   0.001050      22.7   7.84e-01    8.52e-01
## Glutamic acid, 3TMS; 8      -1.98e-04   -1.80e-03   0.001400      23.0   8.08e-01    8.66e-01
## Tridecanoic acid; 74        -1.36e-04   -1.74e-03   0.001470      20.0   8.68e-01    9.05e-01
## 1,3-Propanediol; 34         -1.26e-04   -1.66e-03   0.001400      24.2   8.72e-01    9.05e-01
## Arachidic acid; 46           6.99e-05   -8.46e-04   0.000986      21.8   8.81e-01    9.05e-01
## Docosahexaenoic acid; 53     9.60e-05   -2.06e-03   0.002260      24.2   9.30e-01    9.43e-01
## alpha-Tocopherol; 26        -2.41e-05   -2.12e-03   0.002070      18.8   9.82e-01    9.82e-01
```

1.2.2 Adjusted Model

```
## [1] 586  12

##      (Intercept)      egfr   Age Gender Hba1c_baseline    logUAER CALSBP
## [1,]           1  11.03376 19.39      0            5.2  0.5849625     92
## [2,]           1 167.62905 85.23      1           15.0 13.0138461    191
##        bmi Smoking Statin log_Blood_TGA Total_cholesterol
## [1,] 16.98       0      0     -2.643856               2.3
## [2,] 43.29       1      1      2.720278               9.2

##
##                                 Overall
##   n                                586
##   egfr (mean (sd))               82.04 (28.24)
##   Age (mean (sd))                55.37 (12.11)
##   Gender (mean (sd))              0.54 (0.50)
##   Hba1c_baseline (mean (sd))      8.03 (1.16)
##   logUAER (mean (sd))             4.73 (2.31)
##   CALSBP (mean (sd))            131.91 (17.48)
##   bmi (mean (sd))                25.20 (4.06)
##   Smoking (mean (sd))             0.21 (0.41)
##   Statin (mean (sd))              0.61 (0.49)
##   log_Blood_TGA (mean (sd))       0.01 (0.69)
##   Total_cholesterol (mean (sd))   4.69 (0.88)
```

1.2.2.1 Table

```
##
##
## Table: egfr
##
## Name                             Effect        CI.L        CI.R   AveExpr    P.Value    adj.P.Val
## --------------------------  ----------  ----------  ----------  --------  ---------  ----------
## Myo inositol 6TMS; 1          -9.16e-03   -0.010800   -7.51e-03      19.1   0.00e+00    0.00e+00
## Ribitol; 71                   -1.07e-02   -0.012800   -8.65e-03      23.3   0.00e+00    0.00e+00
## 2,4-Dihydroxybutanoic aci     -9.14e-03   -0.011000   -7.30e-03      16.6   0.00e+00    0.00e+00
## Creatinine; 50                -1.24e-02   -0.015000   -9.86e-03      21.6   0.00e+00    0.00e+00
## Ribonic acid; 72              -1.36e-02   -0.016600   -1.06e-02      20.0   0.00e+00    0.00e+00
## 3,4-Dihydroxybutanoic aci     -6.77e-03   -0.008590   -4.96e-03      15.9   0.00e+00    0.00e+00
## 4-Hydroxybenzeneacetic ac     -1.44e-02   -0.018400   -1.04e-02      19.5   0.00e+00    0.00e+00
## 4-Deoxytetronic acid; 33      -1.21e-02   -0.015500   -8.67e-03      22.2   0.00e+00    0.00e+00
## Isoleucine, 2TMS; 18           5.96e-03    0.004020    7.90e-03      26.0   0.00e+00    0.00e+00
## 4-Deoxytetronic acid; 32      -6.49e-03   -0.008830   -4.15e-03      21.6   1.00e-07    6.00e-07
## Citric acid, 4TMS; 6          -4.34e-03   -0.005940   -2.75e-03      20.8   1.00e-07    9.00e-07
## Pyroglutamic acid; 69         -7.47e-03   -0.010200   -4.71e-03      22.9   2.00e-07    1.00e-06
## 2-Hydroxybutyric acid, 2T      6.06e-03    0.003640    8.49e-03      20.7   1.20e-06    6.90e-06
## Valine, 2TMS; 20               2.96e-03    0.001740    4.19e-03      25.1   2.70e-06    1.44e-05
## 4-Hydroxyphenyllactic aci     -4.94e-03   -0.007030   -2.85e-03      20.8   4.20e-06    2.11e-05
## 3-Indoleacetic acid; 40       -6.09e-03   -0.008750   -3.43e-03      20.5   8.50e-06    3.99e-05
## Glyceryl-glycoside; 59        -4.64e-03   -0.007010   -2.27e-03      20.8   1.35e-04    5.94e-04
## Hydroxyproline; 64            -5.86e-03   -0.008860   -2.85e-03      20.6   1.46e-04    6.07e-04
## Leucine, 2TMS; 19              3.40e-03    0.001630    5.17e-03      21.1   1.80e-04    7.11e-04
## Serine, 3TMS; 14               2.45e-03    0.001130    3.77e-03      26.0   2.83e-04    1.06e-03
## Glycine, 3TMS; 17             -2.08e-03   -0.003230   -9.21e-04      21.4   4.52e-04    1.61e-03
## Fumaric acid, 2TMS; 9         -2.33e-03   -0.003670   -9.91e-04      24.1   6.74e-04    2.30e-03
## Eicosapentaenoic acid; 55      4.61e-03    0.001770    7.45e-03      23.0   1.51e-03    4.91e-03
## Methionine, 2TMS; 16           3.16e-03    0.001060    5.25e-03      20.3   3.20e-03    9.99e-03
## Stearic acid, TMS; 2           1.00e-03    0.000322    1.69e-03      17.4   4.01e-03    1.20e-02
## Malic acid, 3TMS; 11          -2.19e-03   -0.003900   -4.87e-04      20.0   1.18e-02    3.42e-02
## Benzeneacetic acid; 47        -4.18e-03   -0.007650   -7.05e-04      20.4   1.85e-02    5.13e-02
## Octanoic acid; 68              1.42e-03    0.000186    2.65e-03      24.2   2.41e-02    6.31e-02
## Palmitic acid, TMS; 5          9.94e-04    0.000129    1.86e-03      21.4   2.44e-02    6.31e-02
## Tyrosine; 75                   4.63e-03    0.000472    8.79e-03      23.3   2.92e-02    7.29e-02
## Aminomalonic acid; 45         -2.23e-03   -0.004280   -1.87e-04      24.3   3.25e-02    7.86e-02
## Glycerol; 57                   1.76e-03    0.000136    3.38e-03      28.7   3.37e-02    7.90e-02
## Cholesterol, TMS; 23           1.10e-03    0.000077    2.13e-03      21.6   3.51e-02    7.99e-02
## Alanine, 2TMS; 25             -1.33e-03   -0.002590   -6.44e-05      22.8   3.94e-02    8.70e-02
## 2-hydroxy Isovaleric acid      3.94e-03   -0.000288    8.16e-03      22.2   6.77e-02    1.45e-01
## Glycerol; 58                  -3.85e-03   -0.008110    4.09e-04      24.9   7.63e-02    1.59e-01
## 2-Palmitoylglycerol; 39        1.24e-03   -0.000148    2.63e-03      25.9   7.98e-02    1.62e-01
## Campesterol; 49               -2.09e-03   -0.004520    3.44e-04      21.8   9.23e-02    1.81e-01
## Glutamic acid, 3TMS; 8         1.52e-03   -0.000259    3.29e-03      23.0   9.39e-02    1.81e-01
## Succinic acid, 2TMS; 7        -9.45e-04   -0.002080    1.88e-04      22.7   1.02e-01    1.91e-01
## Docosahexaenoic acid; 53       1.69e-03   -0.000718    4.11e-03      24.2   1.68e-01    3.02e-01
## Phenylalanine, 2TMS; 13       -7.92e-04   -0.001920    3.38e-04      24.3   1.69e-01    3.02e-01
## Glyceric acid; 30              1.08e-03   -0.000773    2.93e-03      20.4   2.53e-01    4.42e-01
## alpha-Tocopherol; 26           1.09e-03   -0.001240    3.42e-03      18.8   3.58e-01    6.10e-01
## Decanoic acid; 52              8.78e-04   -0.001130    2.89e-03      22.4   3.90e-01    6.51e-01
## Threonine, 3TMS; 12            7.48e-04   -0.001020    2.52e-03      28.6   4.07e-01    6.63e-01
```

```
## Oleic acid, TMS; 3            5.73e-04   -0.000850    2.00e-03       17.1   4.29e-01    6.85e-01
## Ribitol; 70                  -8.25e-04   -0.003060    1.40e-03       20.2   4.68e-01    7.31e-01
## Bisphenol A; 48               5.85e-04   -0.001290    2.46e-03       21.2   5.41e-01    7.98e-01
## 1,3-Propanediol; 34           5.48e-04   -0.001260    2.35e-03       24.2   5.51e-01    7.98e-01
## Pyruvic acid; 31              9.43e-04   -0.002290    4.18e-03       19.6   5.67e-01    7.98e-01
## Myristoleic acid; 65          1.03e-03   -0.002560    4.63e-03       19.9   5.72e-01    7.98e-01
## Heptadecanoic acid; 60       -4.85e-04   -0.002180    1.21e-03       21.8   5.75e-01    7.98e-01
## 3-Indolepropionic acid; 4     1.23e-03   -0.003080    5.54e-03       19.5   5.77e-01    7.98e-01
## Nonanoic acid; 67            -3.88e-04   -0.001800    1.02e-03       24.4   5.89e-01    7.98e-01
## 11-Eicosenoic acid; 35       -7.13e-04   -0.003350    1.93e-03       21.9   5.96e-01    7.98e-01
## 1-Monopalmitin; 37            4.00e-04   -0.001130    1.93e-03       29.2   6.08e-01    8.00e-01
## Tridecanoic acid; 74         -4.71e-04   -0.002350    1.41e-03       20.0   6.24e-01    8.07e-01
## Hydroxylamine; 62            -4.02e-04   -0.002160    1.35e-03       27.7   6.53e-01    8.30e-01
## Ethanolamine; 56              3.20e-04   -0.001140    1.78e-03       22.7   6.67e-01    8.34e-01
## Linoleic acid, TMS; 4         2.18e-04   -0.000948    1.38e-03       25.7   7.13e-01    8.77e-01
## alpha-ketoglutaric acid,      5.43e-04   -0.002640    3.73e-03       20.3   7.38e-01    8.93e-01
## Heptadecanoic acid; 61       -1.77e-04   -0.001380    1.03e-03       23.4   7.73e-01    9.12e-01
## L-5-Oxoproline; 63            1.79e-04   -0.001070    1.43e-03       27.5   7.78e-01    9.12e-01
## Arabinopyranose; 51           2.95e-04   -0.002430    3.02e-03       20.2   8.32e-01    9.35e-01
## Lactic acid; 29               1.29e-04   -0.001080    1.34e-03       25.4   8.34e-01    9.35e-01
## Tartronic acid; 73            2.75e-04   -0.002660    3.21e-03       21.3   8.54e-01    9.35e-01
## Arachidic acid; 46            9.70e-05   -0.000974    1.17e-03       21.8   8.59e-01    9.35e-01
## Proline, 2TMS; 21             1.56e-04   -0.001620    1.93e-03       20.8   8.64e-01    9.35e-01
## Arachidonic acid, TMS; 24     1.16e-04   -0.001310    1.54e-03       22.7   8.73e-01    9.35e-01
## 4-Hydroxybutanoic acid; 4     4.58e-05   -0.000655    7.47e-04       27.3   8.98e-01    9.48e-01
## Dodecanoic acid; 54           1.02e-04   -0.002020    2.23e-03       22.7   9.25e-01    9.53e-01
## Nonadecanoic acid; 66         7.85e-05   -0.001670    1.82e-03       20.0   9.30e-01    9.53e-01
## 1-Dodecanol; 36              -5.98e-05   -0.001630    1.51e-03       20.2   9.41e-01    9.53e-01
## 3-Hydroxybutyric acid, 2T     3.13e-05   -0.002880    2.94e-03       21.7   9.83e-01    9.83e-01
## [1] 24
## [1] 26
## [1] 34
##
##
## Table: Age
##
## Name                            Effect        CI.L       CI.R   AveExpr    P.Value   adj.P.Val
## --------------------------  ----------  ----------  ---------  --------  ---------  ----------
## Eicosapentaenoic acid; 55     0.022900    1.64e-02   0.029300      23.0   0.000000      0.0000
## Docosahexaenoic acid; 53      0.010400    4.97e-03   0.015900      24.2   0.000195      0.0073
## alpha-ketoglutaric acid,      0.011900    4.72e-03   0.019200      20.3   0.001220      0.0306
## Pyruvic acid; 31              0.011500    4.21e-03   0.018900      19.6   0.002080      0.0387
## alpha-Tocopherol; 26          0.008130    2.85e-03   0.013400      18.8   0.002580      0.0387
## Malic acid, 3TMS; 11          0.005580    1.71e-03   0.009450      20.0   0.004820      0.0483
## Palmitic acid, TMS; 5         0.002810    8.51e-04   0.004770      21.4   0.005030      0.0483
## Decanoic acid; 52             0.006510    1.96e-03   0.011100      22.4   0.005160      0.0483
## 4-Hydroxybenzeneacetic ac     0.012500    3.38e-03   0.021700      19.5   0.007340      0.0612
## 3-Indoleacetic acid; 40       0.008140    2.10e-03   0.014200      20.5   0.008320      0.0624
## Nonadecanoic acid; 66         0.005200    1.25e-03   0.009160      20.0   0.009990      0.0681
## Glyceric acid; 30             0.005420    1.23e-03   0.009620      20.4   0.011400      0.0713
## Ribitol; 70                   0.006200    1.15e-03   0.011300      20.2   0.016300      0.0901
## Oleic acid, TMS; 3            0.003920    6.94e-04   0.007140      17.1   0.017300      0.0901
## Myristoleic acid; 65          0.009840    1.69e-03   0.018000      19.9   0.018000      0.0901
## 2,4-Dihydroxybutanoic aci     0.004850    6.67e-04   0.009030      16.6   0.023100      0.1080
```

```
## Glutamic acid, 3TMS; 8        0.004560   5.31e-04  0.008580      23.0  0.026600     0.1160
## Succinic acid, 2TMS; 7        0.002880   3.15e-04  0.005450      22.7  0.027800     0.1160
## 11-Eicosenoic acid; 35        0.006290   3.08e-04  0.012300      21.9  0.039400     0.1490
## Stearic acid, TMS; 2          0.001620   7.42e-05  0.003170      17.4  0.040000     0.1490
## Myo inositol 6TMS; 1          0.003840   1.06e-04  0.007570      19.1  0.043800     0.1490
## Fumaric acid, 2TMS; 9         0.003120   8.27e-05  0.006150      24.1  0.044100     0.1490
## Tartronic acid; 73            0.006760   1.00e-04  0.013400      21.3  0.046700     0.1490
## Heptadecanoic acid; 61        0.002760   2.97e-05  0.005490      23.4  0.047600     0.1490
## Alanine, 2TMS; 25             0.002860  -5.00e-06  0.005730      22.8  0.050400     0.1510
## Tyrosine; 75                  0.009330  -9.99e-05  0.018800      23.3  0.052500     0.1510
## Dodecanoic acid; 54           0.004640  -1.84e-04  0.009460      22.7  0.059400     0.1640
## Glycerol; 57                  0.003510  -1.68e-04  0.007180      28.7  0.061400     0.1640
## Ribitol; 71                   0.004400  -3.41e-04  0.009150      23.3  0.068800     0.1780
## Hydroxylamine; 62            -0.003550  -7.53e-03  0.000427      27.7  0.080100     0.2000
## Ribonic acid; 72              0.005740  -1.07e-03  0.012500      20.0  0.098400     0.2380
## 1-Dodecanol; 36              -0.002960  -6.53e-03  0.000604      20.2  0.103000     0.2420
## 3,4-Dihydroxybutanoic aci     0.003310  -8.07e-04  0.007430      15.9  0.115000     0.2600
## 4-Hydroxyphenyllactic aci     0.003780  -9.60e-04  0.008510      20.8  0.118000     0.2600
## Methionine, 2TMS; 16          0.003740  -1.00e-03  0.008490      20.3  0.122000     0.2610
## Phenylalanine, 2TMS; 13       0.001970  -5.88e-04  0.004530      24.3  0.131000     0.2720
## L-5-Oxoproline; 63            0.002090  -7.38e-04  0.004920      27.5  0.147000     0.2930
## Campesterol; 49              -0.004020  -9.53e-03  0.001490      21.8  0.153000     0.2930
## Aminomalonic acid; 45         0.003380  -1.26e-03  0.008020      24.3  0.154000     0.2930
## Arabinopyranose; 51          -0.004470  -1.06e-02  0.001710      20.2  0.156000     0.2930
## 2-Palmitoylglycerol; 39       0.002250  -8.93e-04  0.005400      25.9  0.160000     0.2930
## Heptadecanoic acid; 60        0.002710  -1.13e-03  0.006560      21.8  0.166000     0.2970
## Hydroxyproline; 64           -0.004610  -1.14e-02  0.002210      20.6  0.185000     0.3220
## Tridecanoic acid; 74         -0.002680  -6.95e-03  0.001600      20.0  0.219000     0.3730
## 2-hydroxy Isovaleric acid    -0.005580  -1.52e-02  0.004000      22.2  0.253000     0.4210
## Threonine, 3TMS; 12          -0.002310  -6.32e-03  0.001700      28.6  0.258000     0.4210
## Citric acid, 4TMS; 6          0.002040  -1.58e-03  0.005650      20.8  0.269000     0.4290
## Octanoic acid; 68             0.001550  -1.24e-03  0.004340      24.2  0.276000     0.4310
## 3-Indolepropionic acid; 4    -0.005210  -1.50e-02  0.004560      19.5  0.295000     0.4520
## Glycine, 3TMS; 17             0.001380  -1.24e-03  0.004000      21.4  0.302000     0.4540
## Pyroglutamic acid; 69         0.003210  -3.05e-03  0.009470      22.9  0.314000     0.4620
## Benzeneacetic acid; 47        0.003850  -4.03e-03  0.011700      20.4  0.338000     0.4870
## Valine, 2TMS; 20             -0.001340  -4.12e-03  0.001450      25.1  0.346000     0.4900
## 4-Deoxytetronic acid; 33     -0.003660  -1.14e-02  0.004100      22.2  0.354000     0.4920
## 1-Monopalmitin; 37            0.001540  -1.94e-03  0.005010      29.2  0.385000     0.5250
## Arachidic acid; 46            0.001010  -1.42e-03  0.003440      21.8  0.414000     0.5540
## Lactic acid; 29               0.001090  -1.65e-03  0.003840      25.4  0.434000     0.5710
## Proline, 2TMS; 21             0.001480  -2.55e-03  0.005510      20.8  0.471000     0.5960
## Nonanoic acid; 67            -0.001150  -4.35e-03  0.002040      24.4  0.479000     0.5960
## Arachidonic acid, TMS; 24    -0.001150  -4.39e-03  0.002080      22.7  0.484000     0.5960
## Leucine, 2TMS; 19            -0.001430  -5.45e-03  0.002590      21.1  0.484000     0.5960
## Bisphenol A; 48              -0.001200  -5.46e-03  0.003060      21.2  0.579000     0.6930
## 1,3-Propanediol; 34           0.001150  -2.95e-03  0.005240      24.2  0.582000     0.6930
## Cholesterol, TMS; 23          0.000497  -1.83e-03  0.002820      21.6  0.675000     0.7910
## 2-Hydroxybutyric acid, 2T     0.001130  -4.37e-03  0.006630      20.7  0.686000     0.7920
## Glyceryl-glycoside; 59       -0.000916  -6.29e-03  0.004460      20.8  0.738000     0.8390
## Creatinine; 50               -0.000823  -6.63e-03  0.004980      21.6  0.781000     0.8640
## Linoleic acid, TMS; 4        -0.000370  -3.01e-03  0.002270      25.7  0.783000     0.8640
## Ethanolamine; 56              0.000347  -2.96e-03  0.003660      22.7  0.837000     0.9070
## 3-Hydroxybutyric acid, 2T    -0.000604  -7.20e-03  0.005990      21.7  0.857000     0.9070
```

```
## Isoleucine, 2TMS; 18          0.000399   -4.01e-03   0.004800      26.0   0.859000      0.9070
## 4-Hydroxybutanoic acid; 4    -0.000122   -1.71e-03   0.001470      27.3   0.880000      0.9170
## Glycerol; 58                 -0.000592   -1.03e-02   0.009070      24.9   0.904000      0.9290
## 4-Deoxytetronic acid; 32     -0.000222   -5.53e-03   0.005090      21.6   0.934000      0.9380
## Serine, 3TMS; 14              0.000118   -2.87e-03   0.003110      26.0   0.938000      0.9380
## [1] 2
## [1] 8
## [1] 15
##
##
## Table: Gender
##
## Name                              Effect        CI.L        CI.R   AveExpr     P.Value   adj.P.Val
## --------------------------  ---------  ---------  ---------  --------  ---------  ----------
## 4-Deoxytetronic acid; 33       0.43600     0.26200     0.60900      22.2    1.00e-06    0.000078
## Tartronic acid; 73            -0.34800    -0.49700    -0.19900      21.3    5.20e-06    0.000197
## Glyceric acid; 30             -0.21100    -0.30400    -0.11700      20.4    1.18e-05    0.000294
## Proline, 2TMS; 21              0.19600     0.10600     0.28600      20.8    2.19e-05    0.000355
## Methionine, 2TMS; 16           0.23000     0.12400     0.33600      20.3    2.37e-05    0.000355
## Myristoleic acid; 65          -0.38900    -0.57000    -0.20700      19.9    3.16e-05    0.000392
## Valine, 2TMS; 20               0.13200     0.06950     0.19400      25.1    3.66e-05    0.000392
## Cholesterol, TMS; 23          -0.10300    -0.15500    -0.05080      21.6    1.13e-04    0.001060
## Leucine, 2TMS; 19              0.17000     0.08080     0.26000      21.1    2.08e-04    0.001730
## Oleic acid, TMS; 3            -0.12800    -0.20000    -0.05610      17.1    5.12e-04    0.003840
## Citric acid, 4TMS; 6          -0.14200    -0.22300    -0.06180      20.8    5.64e-04    0.003850
## Dodecanoic acid; 54           -0.18800    -0.29600    -0.08030      22.7    6.49e-04    0.004060
## Glycine, 3TMS; 17             -0.09850    -0.15700    -0.04000      21.4    9.98e-04    0.005760
## Isoleucine, 2TMS; 18           0.16300     0.06460     0.26100      26.0    1.20e-03    0.006430
## Hydroxyproline; 64             0.24000     0.08760     0.39200      20.6    2.07e-03    0.010300
## Docosahexaenoic acid; 53      -0.18500    -0.30700    -0.06280      24.2    3.05e-03    0.013500
## Aminomalonic acid; 45         -0.15700    -0.26100    -0.05330      24.3    3.07e-03    0.013500
## 2-hydroxy Isovaleric acid      0.32000     0.10700     0.53400      22.2    3.38e-03    0.013600
## Decanoic acid; 52             -0.15200    -0.25400    -0.05040      22.4    3.44e-03    0.013600
## Stearic acid, TMS; 2          -0.04970    -0.08420    -0.01510      17.4    4.95e-03    0.018600
## Glutamic acid, 3TMS; 8         0.12500     0.03520     0.21500      23.0    6.46e-03    0.023100
## Succinic acid, 2TMS; 7        -0.07610    -0.13300    -0.01880      22.7    9.36e-03    0.031100
## Palmitic acid, TMS; 5         -0.05790    -0.10200    -0.01420      21.4    9.55e-03    0.031100
## Tridecanoic acid; 74          -0.12300    -0.21900    -0.02820      20.0    1.12e-02    0.035000
## Nonadecanoic acid; 66         -0.10700    -0.19500    -0.01860      20.0    1.77e-02    0.053200
## Heptadecanoic acid; 61        -0.07220    -0.13300    -0.01120      23.4    2.04e-02    0.058800
## Heptadecanoic acid; 60        -0.09470    -0.18100    -0.00898      21.8    3.04e-02    0.084600
## Tyrosine; 75                  -0.22600    -0.43700    -0.01590      23.3    3.51e-02    0.094000
## Benzeneacetic acid; 47        -0.17200    -0.34800     0.00354      20.4    5.48e-02    0.142000
## Glycerol; 57                  -0.07930    -0.16100     0.00271      28.7    5.80e-02    0.145000
## 1-Monopalmitin; 37             0.07360    -0.00389     0.15100      29.2    6.26e-02    0.152000
## Arachidonic acid, TMS; 24     -0.06140    -0.13400     0.01080      22.7    9.54e-02    0.224000
## 11-Eicosenoic acid; 35        -0.11200    -0.24500     0.02190      21.9    1.01e-01    0.230000
## Nonanoic acid; 67             -0.05890    -0.13000     0.01250      24.4    1.06e-01    0.233000
## Myo inositol 6TMS; 1          -0.06240    -0.14600     0.02080      19.1    1.41e-01    0.303000
## Linoleic acid, TMS; 4         -0.04320    -0.10200     0.01580      25.7    1.51e-01    0.315000
## Pyruvic acid; 31               0.11700    -0.04650     0.28100      19.6    1.60e-01    0.325000
## alpha-Tocopherol; 26          -0.08030    -0.19800     0.03750      18.8    1.81e-01    0.358000
## Threonine, 3TMS; 12            0.05980    -0.02970     0.14900      28.6    1.90e-01    0.365000
## Ethanolamine; 56               0.04790    -0.02600     0.12200      22.7    2.03e-01    0.381000
```

```
## 2-Palmitoylglycerol; 39       0.04020   -0.03000    0.11000      25.9   2.61e-01    0.454000
## 2,4-Dihydroxybutanoic aci      0.05290   -0.04040    0.14600      16.6   2.66e-01    0.454000
## 2-Hydroxybutyric acid, 2T     -0.06880   -0.19200    0.05400      20.7   2.72e-01    0.454000
## 3-Indolepropionic acid; 4     -0.12200   -0.34000    0.09610      19.5   2.72e-01    0.454000
## Alanine, 2TMS; 25              0.03580   -0.02820    0.09980      22.8   2.73e-01    0.454000
## 4-Hydroxybutanoic acid; 4     -0.01780   -0.05330    0.01770      27.3   3.25e-01    0.530000
## Ribonic acid; 72              -0.07180   -0.22400    0.08020      20.0   3.54e-01    0.562000
## 4-Hydroxyphenyllactic aci      0.04920   -0.05650    0.15500      20.8   3.61e-01    0.562000
## 1-Dodecanol; 36               -0.03660   -0.11600    0.04300      20.2   3.67e-01    0.562000
## Creatinine; 50                 0.05820   -0.07140    0.18800      21.6   3.78e-01    0.567000
## 4-Hydroxybenzeneacetic ac      0.08420   -0.12000    0.28800      19.5   4.18e-01    0.607000
## 4-Deoxytetronic acid; 32      -0.04830   -0.16700    0.07020      21.6   4.24e-01    0.607000
## 3-Indoleacetic acid; 40        0.05430   -0.08040    0.18900      20.5   4.29e-01    0.607000
## Eicosapentaenoic acid; 55     -0.05510   -0.19900    0.08850      23.0   4.51e-01    0.627000
## Serine, 3TMS; 14              -0.02470   -0.09140    0.04210      26.0   4.68e-01    0.629000
## Octanoic acid; 68              0.02290   -0.03930    0.08520      24.2   4.70e-01    0.629000
## Campesterol; 49                0.04180   -0.08120    0.16500      21.8   5.05e-01    0.665000
## Ribitol; 71                    0.03520   -0.07070    0.14100      23.3   5.14e-01    0.665000
## Lactic acid; 29               -0.01910   -0.08030    0.04210      25.4   5.41e-01    0.686000
## Pyroglutamic acid; 69          0.04260   -0.09710    0.18200      22.9   5.50e-01    0.686000
## Arachidic acid; 46            -0.01620   -0.07040    0.03800      21.8   5.58e-01    0.686000
## Bisphenol A; 48               -0.02660   -0.12200    0.06850      21.2   5.83e-01    0.705000
## 3-Hydroxybutyric acid, 2T      0.03720   -0.11000    0.18400      21.7   6.20e-01    0.738000
## Glycerol; 58                  -0.05140   -0.26700    0.16400      24.9   6.40e-01    0.750000
## 1,3-Propanediol; 34           -0.01790   -0.10900    0.07350      24.2   7.00e-01    0.808000
## Fumaric acid, 2TMS; 9         -0.01240   -0.08010    0.05530      24.1   7.20e-01    0.818000
## Ribitol; 70                    0.01860   -0.09430    0.13100      20.2   7.47e-01    0.836000
## Malic acid, 3TMS; 11          -0.01250   -0.09890    0.07390      20.0   7.76e-01    0.856000
## Glyceryl-glycoside; 59         0.01450   -0.10500    0.13400      20.8   8.13e-01    0.883000
## alpha-ketoglutaric acid,       0.01030   -0.15100    0.17100      20.3   9.00e-01    0.956000
## Arabinopyranose; 51           -0.00731   -0.14500    0.13100      20.2   9.17e-01    0.956000
## 3,4-Dihydroxybutanoic aci      0.00445   -0.08750    0.09640      15.9   9.24e-01    0.956000
## Hydroxylamine; 62             -0.00395   -0.09270    0.08480      27.7   9.30e-01    0.956000
## L-5-Oxoproline; 63            -0.00147   -0.06460    0.06170      27.5   9.63e-01    0.968000
## Phenylalanine, 2TMS; 13       -0.00116   -0.05830    0.05600      24.3   9.68e-01    0.968000
## [1] 14
## [1] 24
## [1] 28
##
##
## Table: Hba1c_baseline
##
## Name                            Effect        CI.L        CI.R   AveExpr     P.Value   adj.P.Val
## --------------------------  ----------  ----------  ----------  --------  ----------  ----------
## Valine, 2TMS; 20              0.051900    0.024100     0.07960      25.1    0.000265      0.0135
## Ethanolamine; 56              0.058300    0.025200     0.09130      22.7    0.000566      0.0135
## Eicosapentaenoic acid; 55    -0.113000   -0.177000    -0.04860      23.0    0.000600      0.0135
## Alanine, 2TMS; 25             0.048800    0.020200     0.07740      22.8    0.000852      0.0135
## Arabinopyranose; 51           0.105000    0.043100     0.16600      20.2    0.000900      0.0135
## Docosahexaenoic acid; 53     -0.083200   -0.138000    -0.02860      24.2    0.002860      0.0357
## Citric acid, 4TMS; 6          0.053400    0.017400     0.08940      20.8    0.003740      0.0401
## Glyceric acid; 30            -0.054600   -0.096400    -0.01270      20.4    0.010700      0.1000
## Decanoic acid; 52            -0.057700   -0.103000    -0.01230      22.4    0.012800      0.1070
## 4-Hydroxybutanoic acid; 4    -0.019500   -0.035400    -0.00370      27.3    0.015700      0.1070
```

```
## Tridecanoic acid; 74        -0.052500   -0.095100   -0.00993      20.0   0.015700      0.1070
## Campesterol; 49              0.064800    0.009880    0.12000      21.8   0.020800      0.1300
## Glyceryl-glycoside; 59       0.060200    0.006570    0.11400      20.8   0.027900      0.1590
## Lactic acid; 29              0.030100    0.002800    0.05750      25.4   0.030800      0.1590
## Leucine, 2TMS; 19            0.043800    0.003690    0.08380      21.1   0.032400      0.1590
## 4-Deoxytetronic acid; 33    -0.083800   -0.161000   -0.00635      22.2   0.034000      0.1590
## Myristoleic acid; 65        -0.083900   -0.165000   -0.00261      19.9   0.043100      0.1860
## Ribitol; 70                 -0.051600   -0.102000   -0.00124      20.2   0.044600      0.1860
## Isoleucine, 2TMS; 18         0.044200    0.000313    0.08820      26.0   0.048400      0.1910
## Tartronic acid; 73          -0.065100   -0.132000    0.00134      21.3   0.054800      0.2050
## 1,3-Propanediol; 34         -0.038100   -0.079000    0.00270      24.2   0.067100      0.2400
## alpha-ketoglutaric acid,    -0.065200   -0.137000    0.00681      20.3   0.075900      0.2580
## Proline, 2TMS; 21            0.036000   -0.004210    0.07620      20.8   0.079200      0.2580
## Glycine, 3TMS; 17            0.022800   -0.003280    0.04900      21.4   0.086500      0.2700
## 4-Deoxytetronic acid; 32    -0.045400   -0.098300    0.00757      21.6   0.092900      0.2730
## Glutamic acid, 3TMS; 8      -0.034100   -0.074300    0.00599      23.0   0.095300      0.2730
## Malic acid, 3TMS; 11         0.032500   -0.006100    0.07110      20.0   0.098800      0.2730
## Tyrosine; 75                 0.078500   -0.015600    0.17200      23.3   0.102000      0.2730
## 2-Hydroxybutyric acid, 2T    0.043000   -0.011900    0.09780      20.7   0.124000      0.3220
## 11-Eicosenoic acid; 35      -0.045900   -0.106000    0.01380      21.9   0.132000      0.3290
## 1-Monopalmitin; 37          -0.024100   -0.058700    0.01050      29.2   0.173000      0.4180
## Oleic acid, TMS; 3          -0.021700   -0.053900    0.01040      17.1   0.185000      0.4340
## Octanoic acid; 68           -0.018300   -0.046100    0.00949      24.2   0.196000      0.4460
## 3,4-Dihydroxybutanoic aci    0.024300   -0.016700    0.06540      15.9   0.245000      0.5400
## 4-Hydroxybenzeneacetic ac    0.051800   -0.039300    0.14300      19.5   0.264000      0.5400
## Myo inositol 6TMS; 1         0.021000   -0.016200    0.05820      19.1   0.268000      0.5400
## Serine, 3TMS; 14             0.016600   -0.013200    0.04650      26.0   0.273000      0.5400
## alpha-Tocopherol; 26        -0.029300   -0.081900    0.02320      18.8   0.274000      0.5400
## Pyruvic acid; 31            -0.040100   -0.113000    0.03300      19.6   0.282000      0.5420
## 2-Palmitoylglycerol; 39      0.016800   -0.014600    0.04810      25.9   0.294000      0.5520
## 4-Hydroxyphenyllactic aci   -0.023300   -0.070500    0.02390      20.8   0.332000      0.6070
## Benzeneacetic acid; 47       0.035400   -0.043100    0.11400      20.4   0.376000      0.6710
## Ribitol; 71                  0.019200   -0.028100    0.06650      23.3   0.426000      0.7290
## Heptadecanoic acid; 61      -0.010800   -0.038100    0.01640      23.4   0.435000      0.7290
## 2,4-Dihydroxybutanoic aci    0.016500   -0.025200    0.05820      16.6   0.438000      0.7290
## Cholesterol, TMS; 23        -0.008740   -0.031900    0.01440      21.6   0.459000      0.7450
## Nonanoic acid; 67           -0.011800   -0.043700    0.02010      24.4   0.467000      0.7450
## Glycerol; 57                 0.012900   -0.023700    0.04960      28.7   0.489000      0.7630
## Threonine, 3TMS; 12         -0.013000   -0.053000    0.02700      28.6   0.524000      0.8020
## Phenylalanine, 2TMS; 13      0.007990   -0.017500    0.03350      24.3   0.539000      0.8090
## Glycerol; 58                 0.027700   -0.068700    0.12400      24.9   0.573000      0.8260
## 3-Hydroxybutyric acid, 2T    0.018600   -0.047100    0.08440      21.7   0.578000      0.8260
## Fumaric acid, 2TMS; 9        0.008240   -0.022000    0.03850      24.1   0.593000      0.8260
## Dodecanoic acid; 54          0.013000   -0.035100    0.06110      22.7   0.595000      0.8260
## 1-Dodecanol; 36             -0.008930   -0.044500    0.02660      20.2   0.622000      0.8310
## Nonadecanoic acid; 66        0.009760   -0.029700    0.04920      20.0   0.627000      0.8310
## Stearic acid, TMS; 2        -0.003780   -0.019200    0.01170      17.4   0.631000      0.8310
## Hydroxylamine; 62            0.007800   -0.031800    0.04740      27.7   0.699000      0.8910
## Succinic acid, 2TMS; 7      -0.004890   -0.030500    0.02070      22.7   0.708000      0.8910
## Arachidonic acid, TMS; 24   -0.005640   -0.037900    0.02660      22.7   0.732000      0.8910
## Arachidic acid; 46          -0.004130   -0.028300    0.02010      21.8   0.738000      0.8910
## 3-Indolepropionic acid; 4   -0.016500   -0.114000    0.08090      19.5   0.740000      0.8910
## Heptadecanoic acid; 60       0.006250   -0.032100    0.04460      21.8   0.749000      0.8910
## Palmitic acid, TMS; 5        0.002820   -0.016700    0.02240      21.4   0.777000      0.9100
```

```
## Linoleic acid, TMS; 4        -0.003370   -0.029700    0.02300       25.7   0.802000       0.9250
## 3-Indoleacetic acid; 40       0.006210   -0.054000    0.06640       20.5   0.840000       0.9310
## Bisphenol A; 48               0.004200   -0.038300    0.04670       21.2   0.846000       0.9310
## 2-hydroxy Isovaleric acid     0.009280   -0.086200    0.10500       22.2   0.849000       0.9310
## Methionine, 2TMS; 16         -0.004370   -0.051700    0.04300       20.3   0.856000       0.9310
## Ribonic acid; 72             -0.005250   -0.073100    0.06260       20.0   0.879000       0.9420
## Hydroxyproline; 64           -0.003960   -0.071900    0.06400       20.6   0.909000       0.9430
## Creatinine; 50               -0.003190   -0.061100    0.05470       21.6   0.914000       0.9430
## L-5-Oxoproline; 63           -0.001480   -0.029700    0.02670       27.5   0.918000       0.9430
## Pyroglutamic acid; 69        -0.001070   -0.063500    0.06130       22.9   0.973000       0.9790
## Aminomalonic acid; 45         0.000616   -0.045700    0.04690       24.3   0.979000       0.9790
## [1] 0
## [1] 7
## [1] 7
##
##
## Table: logUAER
##
## Name                             Effect        CI.L         CI.R    AveExpr    P.Value   adj.P.Val
## -------------------------  ----------  ---------  ----------  --------  ---------  ----------
## 3,4-Dihydroxybutanoic aci     0.043800    0.02110    0.066500       15.9   0.000166       0.0125
## 4-Deoxytetronic acid; 32      0.046000    0.01670    0.075300       21.6   0.002120       0.0794
## Campesterol; 49              -0.042400   -0.07280   -0.012000       21.8   0.006310       0.1580
## Ribonic acid; 72              0.047700    0.01020    0.085200       20.0   0.012800       0.2250
## Octanoic acid; 68            -0.019000   -0.03440   -0.003650       24.2   0.015400       0.2250
## Glyceric acid; 30            -0.027400   -0.05050   -0.004230       20.4   0.020500       0.2250
## Docosahexaenoic acid; 53     -0.034600   -0.06470   -0.004420       24.2   0.024700       0.2250
## 2-Hydroxybutyric acid, 2T    -0.033600   -0.06400   -0.003280       20.7   0.029900       0.2250
## Aminomalonic acid; 45        -0.027900   -0.05350   -0.002350       24.3   0.032400       0.2250
## 2-hydroxy Isovaleric acid    -0.057600   -0.11000   -0.004780       22.2   0.032600       0.2250
## 2,4-Dihydroxybutanoic aci     0.024900    0.00187    0.048000       16.6   0.034100       0.2250
## Tyrosine; 75                 -0.055600   -0.10800   -0.003640       23.3   0.036000       0.2250
## Glutamic acid, 3TMS; 8        0.023300    0.00107    0.045500       23.0   0.040000       0.2310
## Arachidonic acid, TMS; 24    -0.016900   -0.03470    0.000927       22.7   0.063100       0.3380
## 3-Indolepropionic acid; 4    -0.050100   -0.10400    0.003730       19.5   0.068100       0.3400
## Ribitol; 71                   0.023900   -0.00225    0.050100       23.3   0.073100       0.3430
## Tridecanoic acid; 74         -0.021100   -0.04460    0.002480       20.0   0.079500       0.3510
## Valine, 2TMS; 20             -0.013300   -0.02860    0.002100       25.1   0.090600       0.3770
## Ethanolamine; 56             -0.014800   -0.03310    0.003420       22.7   0.111000       0.4350
## Lactic acid; 29              -0.012100   -0.02720    0.003050       25.4   0.117000       0.4350
## L-5-Oxoproline; 63           -0.012300   -0.02790    0.003290       27.5   0.122000       0.4350
## Ribitol; 70                   0.020000   -0.00788    0.047900       20.2   0.159000       0.5260
## 1,3-Propanediol; 34           0.016100   -0.00645    0.038700       24.2   0.161000       0.5260
## Hydroxyproline; 64            0.025200   -0.01240    0.062800       20.6   0.188000       0.5660
## Methionine, 2TMS; 16         -0.017300   -0.04350    0.008860       20.3   0.194000       0.5660
## 4-Hydroxybutanoic acid; 4     0.005770   -0.00299    0.014500       27.3   0.196000       0.5660
## 1-Dodecanol; 36              -0.012500   -0.03210    0.007190       20.2   0.213000       0.5770
## Proline, 2TMS; 21             0.013700   -0.00851    0.036000       20.8   0.226000       0.5770
## Heptadecanoic acid; 61       -0.009140   -0.02420    0.005920       23.4   0.234000       0.5770
## Pyroglutamic acid; 69        -0.020900   -0.05540    0.013600       22.9   0.235000       0.5770
## alpha-Tocopherol; 26         -0.017100   -0.04610    0.012000       18.8   0.250000       0.5770
## Myo inositol 6TMS; 1          0.011900   -0.00868    0.032400       19.1   0.257000       0.5770
## Creatinine; 50                0.018100   -0.01390    0.050100       21.6   0.267000       0.5770
## Glyceryl-glycoside; 59        0.016100   -0.01350    0.045700       20.8   0.286000       0.5770
```

```
## Arachidic acid; 46         -0.007270   -0.02070   0.006120      21.8   0.287000      0.5770
## Glycerol; 58                0.028900   -0.02440   0.082200      24.9   0.287000      0.5770
## Heptadecanoic acid; 60     -0.011300   -0.03250   0.009870      21.8   0.295000      0.5770
## 4-Hydroxyphenyllactic aci  -0.013900   -0.04010   0.012200      20.8   0.295000      0.5770
## Cholesterol, TMS; 23       -0.006760   -0.01960   0.006050      21.6   0.300000      0.5770
## Succinic acid, 2TMS; 7     -0.006760   -0.02090   0.007410      22.7   0.349000      0.6550
## Threonine, 3TMS; 12        -0.009900   -0.03200   0.012200      28.6   0.380000      0.6650
## Linoleic acid, TMS; 4      -0.006490   -0.02110   0.008090      25.7   0.382000      0.6650
## Glycine, 3TMS; 17          -0.006380   -0.02080   0.008070      21.4   0.386000      0.6650
## 1-Monopalmitin; 37          0.008300   -0.01080   0.027400      29.2   0.394000      0.6650
## Citric acid, 4TMS; 6       -0.008560   -0.02850   0.011400      20.8   0.399000      0.6650
## 4-Deoxytetronic acid; 33    0.016600   -0.02630   0.059400      22.2   0.448000      0.7160
## alpha-ketoglutaric acid,    0.015400   -0.02440   0.055200      20.3   0.449000      0.7160
## 4-Hydroxybenzeneacetic ac   0.017700   -0.03270   0.068100      19.5   0.491000      0.7680
## Decanoic acid; 52           0.007340   -0.01780   0.032400      22.4   0.566000      0.8630
## 3-Indoleacetic acid; 40     0.009500   -0.02380   0.042800      20.5   0.575000      0.8630
## Palmitic acid, TMS; 5      -0.002710   -0.01350   0.008100      21.4   0.622000      0.9080
## Arabinopyranose; 51         0.007880   -0.02620   0.041900      20.2   0.650000      0.9080
## 2-Palmitoylglycerol; 39     0.003750   -0.01360   0.021100      25.9   0.671000      0.9080
## Isoleucine, 2TMS; 18        0.004830   -0.01950   0.029100      26.0   0.696000      0.9080
## Malic acid, 3TMS; 11        0.004200   -0.01710   0.025500      20.0   0.699000      0.9080
## Alanine, 2TMS; 25          -0.003040   -0.01880   0.012800      22.8   0.706000      0.9080
## Nonadecanoic acid; 66       0.004190   -0.01760   0.026000      20.0   0.706000      0.9080
## Dodecanoic acid; 54        -0.005030   -0.03160   0.021600      22.7   0.710000      0.9080
## Benzeneacetic acid; 47     -0.008090   -0.05150   0.035300      20.4   0.714000      0.9080
## Fumaric acid, 2TMS; 9      -0.002930   -0.01970   0.013800      24.1   0.731000      0.9140
## Stearic acid, TMS; 2       -0.001380   -0.00992   0.007160      17.4   0.751000      0.9210
## Hydroxylamine; 62          -0.003130   -0.02510   0.018800      27.7   0.779000      0.9210
## Tartronic acid; 73          0.005000   -0.03170   0.041700      21.3   0.789000      0.9210
## Serine, 3TMS; 14            0.002030   -0.01450   0.018500      26.0   0.809000      0.9210
## Oleic acid, TMS; 3          0.002190   -0.01560   0.020000      17.1   0.809000      0.9210
## Myristoleic acid; 65       -0.004910   -0.04990   0.040000      19.9   0.830000      0.9210
## Nonanoic acid; 67           0.001890   -0.01570   0.019500      24.4   0.833000      0.9210
## Pyruvic acid; 31           -0.004170   -0.04460   0.036300      19.6   0.840000      0.9210
## 3-Hydroxybutyric acid, 2T  -0.003570   -0.03990   0.032800      21.7   0.847000      0.9210
## 11-Eicosenoic acid; 35      0.002600   -0.03040   0.035600      21.9   0.877000      0.9400
## Leucine, 2TMS; 19           0.001260   -0.02090   0.023400      21.1   0.911000      0.9620
## Phenylalanine, 2TMS; 13    -0.000668   -0.01480   0.013500      24.3   0.926000      0.9650
## Bisphenol A; 48             0.000787   -0.02270   0.024300      21.2   0.948000      0.9650
## Eicosapentaenoic acid; 55   0.001090   -0.03440   0.036600      23.0   0.952000      0.9650
## Glycerol; 57                0.000456   -0.01980   0.020700      28.7   0.965000      0.9650
## [1] 0
## [1] 1
## [1] 2
##
##
## Table: CALSBP
##
## Name                            Effect        CI.L        CI.R   AveExpr    P.Value   adj.P.Val
## --------------------------  ----------  ----------  ----------  --------  --------  ----------
## Myristoleic acid; 65          8.49e-03    3.13e-03    1.39e-02      19.9   0.00195       0.147
## 3-Indoleacetic acid; 40      -4.92e-03   -8.90e-03   -9.52e-04      20.5   0.01520       0.498
## Dodecanoic acid; 54           3.47e-03    3.00e-04    6.64e-03      22.7   0.03200       0.498
## Creatinine; 50               -4.05e-03   -7.87e-03   -2.29e-04      21.6   0.03780       0.498
```

```
## Tridecanoic acid; 74          2.97e-03   1.57e-04   5.78e-03      20.0  0.03860        0.498
## Alanine, 2TMS; 25            -1.92e-03  -3.81e-03  -3.69e-05      22.8  0.04570        0.498
## Hydroxylamine; 62             2.66e-03   4.11e-05   5.27e-03      27.7  0.04650        0.498
## Arachidic acid; 46            1.56e-03  -4.15e-05   3.15e-03      21.8  0.05620        0.522
## 2-Hydroxybutyric acid, 2T     3.38e-03  -2.35e-04   7.00e-03      20.7  0.06680        0.522
## Glyceric acid; 30            -2.54e-03  -5.30e-03   2.25e-04      20.4  0.07170        0.522
## Oleic acid, TMS; 3            1.87e-03  -2.52e-04   3.99e-03      17.1  0.08400        0.522
## Palmitic acid, TMS; 5         1.10e-03  -1.93e-04   2.39e-03      21.4  0.09550        0.522
## Proline, 2TMS; 21            -2.24e-03  -4.89e-03   4.14e-04      20.8  0.09800        0.522
## 3-Hydroxybutyric acid, 2T     3.62e-03  -7.15e-04   7.96e-03      21.7  0.10200        0.522
## Linoleic acid, TMS; 4         1.44e-03  -2.99e-04   3.18e-03      25.7  0.10400        0.522
## 4-Deoxytetronic acid; 32     -2.82e-03  -6.31e-03   6.70e-04      21.6  0.11300        0.530
## Nonadecanoic acid; 66        -2.04e-03  -4.64e-03   5.61e-04      20.0  0.12400        0.547
## Cholesterol, TMS; 23          1.12e-03  -4.07e-04   2.65e-03      21.6  0.15000        0.553
## 2-Palmitoylglycerol; 39      -1.52e-03  -3.59e-03   5.54e-04      25.9  0.15100        0.553
## Campesterol; 49               2.64e-03  -9.85e-04   6.26e-03      21.8  0.15300        0.553
## 1-Monopalmitin; 37           -1.65e-03  -3.94e-03   6.30e-04      29.2  0.15500        0.553
## Methionine, 2TMS; 16         -2.22e-03  -5.35e-03   8.98e-04      20.3  0.16200        0.553
## Tyrosine; 75                 -4.08e-03  -1.03e-02   2.12e-03      23.3  0.19700        0.642
## Decanoic acid; 52             1.87e-03  -1.13e-03   4.86e-03      22.4  0.22100        0.679
## Tartronic acid; 73           -2.70e-03  -7.09e-03   1.68e-03      21.3  0.22600        0.679
## Glycerol; 57                  1.44e-03  -9.81e-04   3.85e-03      28.7  0.24400        0.703
## Serine, 3TMS; 14             -1.07e-03  -3.04e-03   8.95e-04      26.0  0.28500        0.751
## Phenylalanine, 2TMS; 13      -8.56e-04  -2.54e-03   8.29e-04      24.3  0.31900        0.751
## Docosahexaenoic acid; 53      1.77e-03  -1.83e-03   5.37e-03      24.2  0.33500        0.751
## Malic acid, 3TMS; 11         -1.21e-03  -3.76e-03   1.34e-03      20.0  0.35100        0.751
## 4-Hydroxybutanoic acid; 4     4.91e-04  -5.55e-04   1.54e-03      27.3  0.35700        0.751
## Ribitol; 70                  -1.55e-03  -4.88e-03   1.77e-03      20.2  0.35900        0.751
## Heptadecanoic acid; 61        8.19e-04  -9.79e-04   2.62e-03      23.4  0.37100        0.751
## Stearic acid, TMS; 2          4.58e-04  -5.60e-04   1.48e-03      17.4  0.37700        0.751
## 4-Deoxytetronic acid; 33     -2.30e-03  -7.40e-03   2.81e-03      22.2  0.37800        0.751
## L-5-Oxoproline; 63            8.23e-04  -1.04e-03   2.68e-03      27.5  0.38600        0.751
## Glycerol; 58                 -2.79e-03  -9.14e-03   3.57e-03      24.9  0.39000        0.751
## Octanoic acid; 68             7.81e-04  -1.05e-03   2.62e-03      24.2  0.40400        0.751
## Valine, 2TMS; 20             -7.60e-04  -2.59e-03   1.07e-03      25.1  0.41600        0.751
## alpha-Tocopherol; 26          1.43e-03  -2.04e-03   4.90e-03      18.8  0.41700        0.751
## Succinic acid, 2TMS; 7       -6.92e-04  -2.38e-03   9.98e-04      22.7  0.42200        0.751
## Ribitol; 71                  -1.28e-03  -4.40e-03   1.84e-03      23.3  0.42200        0.751
## Glutamic acid, 3TMS; 8       -1.06e-03  -3.71e-03   1.59e-03      23.0  0.43100        0.751
## Bisphenol A; 48               1.06e-03  -1.75e-03   3.86e-03      21.2  0.45900        0.756
## Ethanolamine; 56              7.98e-04  -1.38e-03   2.98e-03      22.7  0.47200        0.756
## Ribonic acid; 72              1.64e-03  -2.84e-03   6.12e-03      20.0  0.47200        0.756
## 4-Hydroxybenzeneacetic ac     2.20e-03  -3.82e-03   8.21e-03      19.5  0.47400        0.756
## Aminomalonic acid; 45        -1.07e-03  -4.13e-03   1.98e-03      24.3  0.49100        0.757
## Fumaric acid, 2TMS; 9         6.76e-04  -1.32e-03   2.67e-03      24.1  0.50600        0.757
## 3,4-Dihydroxybutanoic aci     9.12e-04  -1.80e-03   3.62e-03      15.9  0.50900        0.757
## Eicosapentaenoic acid; 55     1.33e-03  -2.91e-03   5.56e-03      23.0  0.53800        0.757
## Isoleucine, 2TMS; 18         -8.98e-04  -3.80e-03   2.00e-03      26.0  0.54300        0.757
## 11-Eicosenoic acid; 35        1.20e-03  -2.74e-03   5.13e-03      21.9  0.55000        0.757
## 4-Hydroxyphenyllactic aci     9.32e-04  -2.18e-03   4.05e-03      20.8  0.55700        0.757
## Lactic acid; 29              -5.35e-04  -2.34e-03   1.27e-03      25.4  0.56100        0.757
## Nonanoic acid; 67            -6.16e-04  -2.72e-03   1.49e-03      24.4  0.56500        0.757
## 1,3-Propanediol; 34          -7.68e-04  -3.46e-03   1.93e-03      24.2  0.57600        0.758
## Heptadecanoic acid; 60        6.15e-04  -1.91e-03   3.14e-03      21.8  0.63300        0.819
```

```
## Glycine, 3TMS; 17            3.85e-04   -1.34e-03    2.11e-03       21.4   0.66100         0.840
## 3-Indolepropionic acid; 4   -1.38e-03   -7.81e-03    5.05e-03       19.5   0.67400         0.842
## Hydroxyproline; 64          -9.22e-04   -5.41e-03    3.56e-03       20.6   0.68700         0.844
## 1-Dodecanol; 36              4.31e-04   -1.92e-03    2.78e-03       20.2   0.71800         0.869
## Citric acid, 4TMS; 6         3.70e-04   -2.01e-03    2.75e-03       20.8   0.76000         0.893
## Arabinopyranose; 51          6.12e-04   -3.45e-03    4.68e-03       20.2   0.76800         0.893
## alpha-ketoglutaric acid,    -6.93e-04   -5.44e-03    4.06e-03       20.3   0.77400         0.893
## Myo inositol 6TMS; 1         3.07e-04   -2.15e-03    2.76e-03       19.1   0.80600         0.916
## Pyruvic acid; 31             3.76e-04   -4.45e-03    5.20e-03       19.6   0.87800         0.943
## Pyroglutamic acid; 69       -3.19e-04   -4.44e-03    3.80e-03       22.9   0.87900         0.943
## Glyceryl-glycoside; 59       2.48e-04   -3.29e-03    3.78e-03       20.8   0.89100         0.943
## 2,4-Dihydroxybutanoic aci   -1.89e-04   -2.94e-03    2.56e-03       16.6   0.89300         0.943
## Leucine, 2TMS; 19            1.55e-04   -2.49e-03    2.80e-03       21.1   0.90900         0.943
## Threonine, 3TMS; 12         -1.51e-04   -2.79e-03    2.49e-03       28.6   0.91000         0.943
## Benzeneacetic acid; 47      -2.73e-04   -5.45e-03    4.91e-03       20.4   0.91800         0.943
## Arachidonic acid, TMS; 24   -2.47e-05   -2.15e-03    2.10e-03       22.7   0.98200         0.988
## 2-hydroxy Isovaleric acid    4.67e-05   -6.25e-03    6.35e-03       22.2   0.98800         0.988
## [1] 0
## [1] 0
## [1] 0
##
##
## Table: bmi
##
## Name                             Effect        CI.L        CI.R   AveExpr    P.Value   adj.P.Val
## --------------------------  ----------  ----------  ----------  --------  ---------  ----------
## Glutamic acid, 3TMS; 8        0.025000    0.013800    0.036300      23.0   1.38e-05     0.00103
## 2-Hydroxybutyric acid, 2T     0.032100    0.016800    0.047400      20.7   4.44e-05     0.00167
## Campesterol; 49              -0.030400   -0.045700   -0.015000      21.8   1.13e-04     0.00282
## Decanoic acid; 52            -0.021600   -0.034300   -0.008910      22.4   8.78e-04     0.01650
## 2,4-Dihydroxybutanoic aci    -0.017300   -0.029000   -0.005670      16.6   3.64e-03     0.05460
## Lactic acid; 29               0.010700    0.003090    0.018400      25.4   5.97e-03     0.06750
## Pyruvic acid; 31             -0.027900   -0.048300   -0.007430      19.6   7.59e-03     0.06750
## 1,3-Propanediol; 34          -0.015500   -0.026900   -0.004090      24.2   7.86e-03     0.06750
## alpha-Tocopherol; 26         -0.019900   -0.034600   -0.005190      18.8   8.10e-03     0.06750
## Arachidic acid; 46           -0.008770   -0.015500   -0.002000      21.8   1.12e-02     0.08370
## Pyroglutamic acid; 69        -0.021500   -0.038900   -0.004020      22.9   1.60e-02     0.10600
## Citric acid, 4TMS; 6         -0.012100   -0.022200   -0.002080      20.8   1.81e-02     0.10600
## Nonadecanoic acid; 66        -0.013300   -0.024300   -0.002250      20.0   1.84e-02     0.10600
## Dodecanoic acid; 54          -0.015400   -0.028800   -0.001950      22.7   2.49e-02     0.13300
## Isoleucine, 2TMS; 18          0.013800    0.001490    0.026000      26.0   2.80e-02     0.14000
## 11-Eicosenoic acid; 35        0.018300    0.001680    0.035000      21.9   3.11e-02     0.14600
## Tartronic acid; 73           -0.020100   -0.038700   -0.001550      21.3   3.37e-02     0.14600
## alpha-ketoglutaric acid,     -0.021600   -0.041700   -0.001510      20.3   3.51e-02     0.14600
## Glycine, 3TMS; 17            -0.007690   -0.015000   -0.000388      21.4   3.90e-02     0.15400
## Ribitol; 70                   0.014700    0.000605    0.028800      20.2   4.10e-02     0.15400
## Octanoic acid; 68            -0.007600   -0.015400    0.000176      24.2   5.54e-02     0.19800
## Ribonic acid; 72             -0.017900   -0.036800    0.001080      20.0   6.45e-02     0.22000
## Phenylalanine, 2TMS; 13       0.006540   -0.000594    0.013700      24.3   7.23e-02     0.22900
## Alanine, 2TMS; 25             0.007300   -0.000688    0.015300      22.8   7.32e-02     0.22900
## Aminomalonic acid; 45        -0.011600   -0.024600    0.001300      24.3   7.79e-02     0.23000
## Proline, 2TMS; 21             0.010000   -0.001210    0.021300      20.8   8.03e-02     0.23000
## 3,4-Dihydroxybutanoic aci    -0.010200   -0.021600    0.001320      15.9   8.27e-02     0.23000
## Valine, 2TMS; 20              0.006630   -0.001120    0.014400      25.1   9.36e-02     0.24700
```

```
## Glyceryl-glycoside; 59       -0.012700   -0.027700    0.002250       20.8   9.56e-02      0.24700
## Leucine, 2TMS; 19             0.009350   -0.001840    0.020500       21.1   1.01e-01      0.25300
## Stearic acid, TMS; 2         -0.003300   -0.007620    0.001010       17.4   1.33e-01      0.31700
## Heptadecanoic acid; 60       -0.008150   -0.018900    0.002550       21.8   1.35e-01      0.31700
## Glycerol; 57                 -0.007300   -0.017500    0.002940       28.7   1.62e-01      0.36300
## Ribitol; 71                  -0.009260   -0.022500    0.003950       23.3   1.69e-01      0.36300
## 1-Monopalmitin; 37           -0.006780   -0.016400    0.002890       29.2   1.69e-01      0.36300
## 1-Dodecanol; 36              -0.006480   -0.016400    0.003460       20.2   2.01e-01      0.41900
## Myristoleic acid; 65         -0.014200   -0.036900    0.008520       19.9   2.20e-01      0.44700
## Nonanoic acid; 67            -0.005470   -0.014400    0.003430       24.4   2.28e-01      0.45000
## Benzeneacetic acid; 47       -0.013200   -0.035100    0.008730       20.4   2.37e-01      0.45700
## Linoleic acid, TMS; 4        -0.004300   -0.011700    0.003060       25.7   2.52e-01      0.47200
## 3-Indolepropionic acid; 4    -0.015500   -0.042700    0.011700       19.5   2.63e-01      0.48100
## Arachidonic acid, TMS; 24     0.004630   -0.004380    0.013600       22.7   3.13e-01      0.55500
## Malic acid, 3TMS; 11         -0.005490   -0.016300    0.005300       20.0   3.18e-01      0.55500
## Myo inositol 6TMS; 1         -0.005040   -0.015400    0.005350       19.1   3.41e-01      0.58100
## Heptadecanoic acid; 61       -0.003360   -0.011000    0.004250       23.4   3.86e-01      0.64400
## Arabinopyranose; 51          -0.007300   -0.024500    0.009910       20.2   4.05e-01      0.66100
## Tyrosine; 75                  0.010100   -0.016200    0.036400       23.3   4.50e-01      0.71300
## Glycerol; 58                  0.010100   -0.016800    0.037000       24.9   4.62e-01      0.71300
## Succinic acid, 2TMS; 7        0.002640   -0.004520    0.009800       22.7   4.69e-01      0.71300
## Ethanolamine; 56              0.003300   -0.005930    0.012500       22.7   4.83e-01      0.71300
## Creatinine; 50               -0.005630   -0.021800    0.010500       21.6   4.94e-01      0.71300
## 4-Hydroxyphenyllactic aci     0.004570   -0.008620    0.017800       20.8   4.96e-01      0.71300
## L-5-Oxoproline; 63           -0.002680   -0.010600    0.005200       27.5   5.04e-01      0.71300
## Hydroxyproline; 64            0.005930   -0.013100    0.024900       20.6   5.40e-01      0.72600
## Fumaric acid, 2TMS; 9        -0.002630   -0.011100    0.005820       24.1   5.41e-01      0.72600
## 3-Indoleacetic acid; 40       0.005220   -0.011600    0.022000       20.5   5.42e-01      0.72600
## Glyceric acid; 30            -0.002990   -0.014700    0.008700       20.4   6.16e-01      0.80200
## 3-Hydroxybutyric acid, 2T    -0.004640   -0.023000    0.013700       21.7   6.20e-01      0.80200
## Tridecanoic acid; 74         -0.002850   -0.014700    0.009050       20.0   6.39e-01      0.80900
## 2-Palmitoylglycerol; 39      -0.002040   -0.010800    0.006720       25.9   6.47e-01      0.80900
## Hydroxylamine; 62             0.002490   -0.008590    0.013600       27.7   6.59e-01      0.81100
## Docosahexaenoic acid; 53     -0.002860   -0.018100    0.012400       24.2   7.12e-01      0.84700
## Cholesterol, TMS; 23          0.001190   -0.005280    0.007660       21.6   7.18e-01      0.84700
## Bisphenol A; 48              -0.002130   -0.014000    0.009730       21.2   7.24e-01      0.84700
## Threonine, 3TMS; 12           0.001940   -0.009240    0.013100       28.6   7.34e-01      0.84700
## Serine, 3TMS; 14             -0.001370   -0.009700    0.006960       26.0   7.47e-01      0.84800
## 4-Hydroxybutanoic acid; 4    -0.000697   -0.005130    0.003730       27.3   7.57e-01      0.84800
## Palmitic acid, TMS; 5        -0.000757   -0.006220    0.004710       21.4   7.86e-01      0.86700
## Oleic acid, TMS; 3           -0.000747   -0.009730    0.008240       17.1   8.70e-01      0.93500
## 4-Deoxytetronic acid; 32      0.001210   -0.013600    0.016000       21.6   8.73e-01      0.93500
## 4-Hydroxybenzeneacetic ac     0.001790   -0.023700    0.027300       19.5   8.90e-01      0.94000
## Methionine, 2TMS; 16         -0.000787   -0.014000    0.012400       20.3   9.07e-01      0.94500
## 4-Deoxytetronic acid; 33     -0.000997   -0.022600    0.020600       22.2   9.28e-01      0.95300
## 2-hydroxy Isovaleric acid    -0.000251   -0.026900    0.026400       22.2   9.85e-01      0.99000
## Eicosapentaenoic acid; 55    -0.000112   -0.018000    0.017800       23.0   9.90e-01      0.99000
## [1] 3
## [1] 4
## [1] 10
##
##
## Table: Smoking
##
```

```
## Name                          Effect       CI.L        CI.R   AveExpr    P.Value   adj.P.Val
## --------------------------  ----------  ---------  ----------  --------  ---------  ----------
## 3-Indolepropionic acid; 4    -0.457000   -0.71700   -0.198000      19.5   0.000573      0.0141
## Docosahexaenoic acid; 53     -0.254000   -0.39900   -0.109000      24.2   0.000633      0.0141
## Glutamic acid, 3TMS; 8        0.186000    0.07880    0.293000      23.0   0.000692      0.0141
## Tartronic acid; 73           -0.305000   -0.48200   -0.128000      21.3   0.000750      0.0141
## Valine, 2TMS; 20             -0.120000   -0.19400   -0.045700      25.1   0.001560      0.0235
## alpha-Tocopherol; 26         -0.215000   -0.35500   -0.074800      18.8   0.002700      0.0292
## Glyceric acid; 30            -0.171000   -0.28200   -0.059400      20.4   0.002720      0.0292
## Ribonic acid; 72             -0.243000   -0.42400   -0.062300      20.0   0.008490      0.0736
## Citric acid, 4TMS; 6         -0.128000   -0.22300   -0.031600      20.8   0.009280      0.0736
## Benzeneacetic acid; 47       -0.275000   -0.48500   -0.066300      20.4   0.009920      0.0736
## Leucine, 2TMS; 19            -0.138000   -0.24500   -0.031600      21.1   0.011100      0.0736
## Malic acid, 3TMS; 11         -0.132000   -0.23500   -0.029500      20.0   0.011800      0.0736
## Campesterol; 49              -0.174000   -0.32100   -0.028100      21.8   0.019600      0.1130
## 3-Indoleacetic acid; 40      -0.167000   -0.32800   -0.007110      20.5   0.040700      0.2180
## 4-Hydroxyphenyllactic aci    -0.128000   -0.25300   -0.002060      20.8   0.046400      0.2320
## Isoleucine, 2TMS; 18         -0.116000   -0.23300    0.000661      26.0   0.051300      0.2400
## Lactic acid; 29               0.070300   -0.00249    0.143000      25.4   0.058300      0.2570
## Heptadecanoic acid; 61       -0.068600   -0.14100    0.003950      23.4   0.063800      0.2640
## Heptadecanoic acid; 60       -0.094200   -0.19600    0.007830      21.8   0.070300      0.2640
## Eicosapentaenoic acid; 55    -0.156000   -0.32700    0.014900      23.0   0.073500      0.2640
## Ribitol; 70                   0.122000   -0.01240    0.256000      20.2   0.075200      0.2640
## Nonadecanoic acid; 66        -0.094500   -0.20000    0.010500      20.0   0.077500      0.2640
## Arabinopyranose; 51          -0.144000   -0.30800    0.020500      20.2   0.086200      0.2810
## Linoleic acid, TMS; 4        -0.052100   -0.12200    0.018100      25.7   0.145000      0.4540
## 3,4-Dihydroxybutanoic aci     0.080000   -0.02940    0.189000      15.9   0.151000      0.4540
## Ethanolamine; 56             -0.058100   -0.14600    0.029800      22.7   0.195000      0.5620
## 1,3-Propanediol; 34          -0.070500   -0.17900    0.038300      24.2   0.204000      0.5660
## Myo inositol 6TMS; 1         -0.063000   -0.16200    0.036000      19.1   0.212000      0.5680
## alpha-ketoglutaric acid,     -0.115000   -0.30600    0.076800      20.3   0.240000      0.6140
## 2-hydroxy Isovaleric acid    -0.150000   -0.40400    0.104000      22.2   0.247000      0.6140
## 2-Palmitoylglycerol; 39       0.048300   -0.03520    0.132000      25.9   0.256000      0.6140
## 4-Deoxytetronic acid; 32     -0.078800   -0.22000    0.062100      21.6   0.273000      0.6140
## Octanoic acid; 68            -0.041000   -0.11500    0.033100      24.2   0.278000      0.6140
## Arachidic acid; 46           -0.035400   -0.09980    0.029100      21.8   0.282000      0.6140
## Glycine, 3TMS; 17             0.037600   -0.03200    0.107000      21.4   0.289000      0.6140
## 4-Deoxytetronic acid; 33     -0.110000   -0.31600    0.096100      22.2   0.295000      0.6140
## Myristoleic acid; 65          0.114000   -0.10300    0.330000      19.9   0.303000      0.6140
## Aminomalonic acid; 45         0.062400   -0.06090    0.186000      24.3   0.321000      0.6330
## Stearic acid, TMS; 2         -0.019300   -0.06040    0.021800      17.4   0.357000      0.6700
## Dodecanoic acid; 54           0.059800   -0.06830    0.188000      22.7   0.360000      0.6700
## 3-Hydroxybutyric acid, 2T    -0.080000   -0.25500    0.095000      21.7   0.370000      0.6700
## Glyceryl-glycoside; 59       -0.064500   -0.20700    0.078200      20.8   0.375000      0.6700
## 11-Eicosenoic acid; 35       -0.058600   -0.21700    0.100000      21.9   0.469000      0.8190
## Methionine, 2TMS; 16         -0.045100   -0.17100    0.080900      20.3   0.482000      0.8220
## Proline, 2TMS; 21            -0.032000   -0.13900    0.075100      20.8   0.557000      0.9150
## 4-Hydroxybutanoic acid; 4    -0.011900   -0.05410    0.030300      27.3   0.580000      0.9150
## Succinic acid, 2TMS; 7       -0.018300   -0.08650    0.049900      22.7   0.599000      0.9150
## L-5-Oxoproline; 63            0.019700   -0.05540    0.094800      27.5   0.606000      0.9150
## Ribitol; 71                  -0.032900   -0.15900    0.093100      23.3   0.608000      0.9150
## Phenylalanine, 2TMS; 13      -0.015700   -0.08370    0.052300      24.3   0.651000      0.9150
## 1-Monopalmitin; 37           -0.020200   -0.11200    0.072000      29.2   0.667000      0.9150
## Oleic acid, TMS; 3            0.018600   -0.06700    0.104000      17.1   0.670000      0.9150
```

```
## Pyroglutamic acid; 69         0.035700   -0.13100   0.202000      22.9   0.673000       0.9150
## Tridecanoic acid; 74         -0.023200   -0.13700   0.090200      20.0   0.688000       0.9150
## Alanine, 2TMS; 25             0.014700   -0.06140   0.090800      22.8   0.704000       0.9150
## 1-Dodecanol; 36              -0.017200   -0.11200   0.077500      20.2   0.722000       0.9150
## 2,4-Dihydroxybutanoic aci     0.019800   -0.09120   0.131000      16.6   0.726000       0.9150
## Glycerol; 58                  0.044000   -0.21200   0.301000      24.9   0.736000       0.9150
## 4-Hydroxybenzeneacetic ac    -0.040900   -0.28400   0.202000      19.5   0.741000       0.9150
## Hydroxyproline; 64           -0.029900   -0.21100   0.151000      20.6   0.746000       0.9150
## Creatinine; 50               -0.024400   -0.17900   0.130000      21.6   0.756000       0.9150
## Arachidonic acid, TMS; 24    -0.013600   -0.09950   0.072300      22.7   0.756000       0.9150
## 2-Hydroxybutyric acid, 2T     0.017300   -0.12900   0.163000      20.7   0.816000       0.9670
## Bisphenol A; 48               0.012100   -0.10100   0.125000      21.2   0.834000       0.9670
## Palmitic acid, TMS; 5        -0.005430   -0.05750   0.046600      21.4   0.838000       0.9670
## Pyruvic acid; 31             -0.017500   -0.21200   0.177000      19.6   0.860000       0.9700
## Decanoic acid; 52             0.010200   -0.11100   0.131000      22.4   0.869000       0.9700
## Threonine, 3TMS; 12           0.008210   -0.09830   0.115000      28.6   0.880000       0.9700
## Glycerol; 57                  0.006220   -0.09130   0.104000      28.7   0.900000       0.9730
## Serine, 3TMS; 14             -0.004410   -0.08380   0.075000      26.0   0.913000       0.9730
## Cholesterol, TMS; 23         -0.003060   -0.06480   0.058600      21.6   0.922000       0.9730
## Nonanoic acid; 67            -0.003570   -0.08840   0.081300      24.4   0.934000       0.9730
## Tyrosine; 75                  0.007370   -0.24300   0.258000      23.3   0.954000       0.9800
## Hydroxylamine; 62             0.001390   -0.10400   0.107000      27.7   0.979000       0.9860
## Fumaric acid, 2TMS; 9         0.000743   -0.07980   0.081300      24.1   0.986000       0.9860
## [1] 0
## [1] 7
## [1] 12
##
##
## Table: Statin
##
## Name                             Effect       CI.L       CI.R   AveExpr   P.Value   adj.P.Val
## --------------------------  ----------  ---------  ---------  --------  --------  ----------
## Campesterol; 49                0.220000    0.08410    0.35600      21.8   0.00156       0.106
## L-5-Oxoproline; 63            -0.104000   -0.17400   -0.03390      27.5   0.00365       0.106
## Arachidonic acid, TMS; 24      0.117000    0.03680    0.19700      22.7   0.00426       0.106
## Glutamic acid, 3TMS; 8         0.130000    0.03100    0.23000      23.0   0.01020       0.192
## Malic acid, 3TMS; 11          -0.112000   -0.20800   -0.01650      20.0   0.02160       0.324
## Glycine, 3TMS; 17             -0.071400   -0.13600   -0.00668      21.4   0.03060       0.344
## Citric acid, 4TMS; 6          -0.097600   -0.18700   -0.00836      20.8   0.03210       0.344
## Aminomalonic acid; 45         -0.121000   -0.23600   -0.00657      24.3   0.03830       0.359
## Cholesterol, TMS; 23          -0.055200   -0.11300    0.00220      21.6   0.05940       0.480
## Ribitol; 70                    0.115000   -0.00958    0.24000      20.2   0.07030       0.480
## Arachidic acid; 46            -0.055300   -0.11500    0.00467      21.8   0.07070       0.480
## Linoleic acid, TMS; 4         -0.058900   -0.12400    0.00635      25.7   0.07670       0.480
## Heptadecanoic acid; 61        -0.059100   -0.12700    0.00839      23.4   0.08600       0.484
## Dodecanoic acid; 54           -0.103000   -0.22200    0.01630      22.7   0.09040       0.484
## Bisphenol A; 48               -0.079800   -0.18500    0.02530      21.2   0.13600       0.673
## Fumaric acid, 2TMS; 9         -0.053600   -0.12900    0.02120      24.1   0.16000       0.673
## 2-Hydroxybutyric acid, 2T      0.097300   -0.03850    0.23300      20.7   0.16000       0.673
## 4-Deoxytetronic acid; 32      -0.093300   -0.22400    0.03780      21.6   0.16300       0.673
## Decanoic acid; 52             -0.076000   -0.18800    0.03630      22.4   0.18400       0.673
## Hydroxylamine; 62              0.065700   -0.03250    0.16400      27.7   0.18900       0.673
## Docosahexaenoic acid; 53       0.089900   -0.04510    0.22500      24.2   0.19200       0.673
## Nonanoic acid; 67             -0.051900   -0.13100    0.02710      24.4   0.19700       0.673
```

```
## Alanine, 2TMS; 25            -0.044900   -0.11600   0.02580     22.8   0.21300        0.694
## Arabinopyranose; 51           0.092100   -0.06040   0.24500     20.2   0.23600        0.708
## Lactic acid; 29              -0.040900   -0.10900   0.02680     25.4   0.23600        0.708
## Ethanolamine; 56             -0.046000   -0.12800   0.03580     22.7   0.27000        0.750
## 11-Eicosenoic acid; 35       -0.081800   -0.23000   0.06590     21.9   0.27700        0.750
## Nonadecanoic acid; 66        -0.051900   -0.15000   0.04570     20.0   0.29700        0.750
## Tartronic acid; 73            0.087300   -0.07720   0.25200     21.3   0.29800        0.750
## Palmitic acid, TMS; 5        -0.025600   -0.07400   0.02280     21.4   0.30000        0.750
## alpha-Tocopherol; 26          0.066800   -0.06340   0.19700     18.8   0.31400        0.760
## Glyceric acid; 30             0.049900   -0.05370   0.15300     20.4   0.34500        0.808
## Myristoleic acid; 65          0.090100   -0.11100   0.29100     19.9   0.37900        0.829
## 1-Dodecanol; 36              -0.039100   -0.12700   0.04900     20.2   0.38400        0.829
## 2-hydroxy Isovaleric acid    -0.104000   -0.34000   0.13300     22.2   0.38900        0.829
## 3-Indoleacetic acid; 40      -0.062900   -0.21200   0.08610     20.5   0.40700        0.829
## Eicosapentaenoic acid; 55     0.063900   -0.09500   0.22300     23.0   0.43000        0.829
## Ribonic acid; 72             -0.067500   -0.23500   0.10100     20.0   0.43100        0.829
## Heptadecanoic acid; 60       -0.037300   -0.13200   0.05760     21.8   0.44000        0.829
## Valine, 2TMS; 20             -0.026700   -0.09550   0.04200     25.1   0.44500        0.829
## Serine, 3TMS; 14             -0.027000   -0.10100   0.04680     26.0   0.47300        0.829
## 4-Hydroxybenzeneacetic ac    -0.079700   -0.30500   0.14600     19.5   0.48800        0.829
## Octanoic acid; 68            -0.023800   -0.09270   0.04510     24.2   0.49800        0.829
## 3-Indolepropionic acid; 4    -0.081900   -0.32300   0.15900     19.5   0.50500        0.829
## Benzeneacetic acid; 47       -0.064300   -0.25900   0.13000     20.4   0.51600        0.829
## Threonine, 3TMS; 12          -0.029500   -0.12900   0.06950     28.6   0.55900        0.829
## Glycerol; 58                 -0.069800   -0.30800   0.16900     24.9   0.56500        0.829
## 2-Palmitoylglycerol; 39      -0.022100   -0.09970   0.05560     25.9   0.57700        0.829
## Proline, 2TMS; 21             0.027700   -0.07180   0.12700     20.8   0.58500        0.829
## Tyrosine; 75                  0.062400   -0.17000   0.29500     23.3   0.59900        0.829
## 4-Hydroxybutanoic acid; 4    -0.010400   -0.04970   0.02880     27.3   0.60200        0.829
## 4-Hydroxyphenyllactic aci     0.031000   -0.08590   0.14800     20.8   0.60300        0.829
## Leucine, 2TMS; 19            -0.025900   -0.12500   0.07330     21.1   0.60900        0.829
## 3-Hydroxybutyric acid, 2T     0.042300   -0.12100   0.20500     21.7   0.61000        0.829
## 1-Monopalmitin; 37           -0.021800   -0.10800   0.06380     29.2   0.61700        0.829
## Pyruvic acid; 31              0.045600   -0.13500   0.22700     19.6   0.62100        0.829
## Oleic acid, TMS; 3           -0.019500   -0.09910   0.06010     17.1   0.63000        0.829
## Tridecanoic acid; 74         -0.024000   -0.12900   0.08150     20.0   0.65600        0.837
## Creatinine; 50               -0.032100   -0.17500   0.11100     21.6   0.66000        0.837
## Ribitol; 71                   0.025400   -0.09170   0.14300     23.3   0.67000        0.837
## Isoleucine, 2TMS; 18         -0.020400   -0.12900   0.08840     26.0   0.71300        0.877
## Phenylalanine, 2TMS; 13       0.011200   -0.05200   0.07450     24.3   0.72700        0.879
## Myo inositol 6TMS; 1         -0.015500   -0.10800   0.07660     19.1   0.74100        0.882
## 1,3-Propanediol; 34          -0.013000   -0.11400   0.08810     24.2   0.80000        0.913
## Succinic acid, 2TMS; 7       -0.007870   -0.07130   0.05560     22.7   0.80700        0.913
## 3,4-Dihydroxybutanoic aci    -0.012500   -0.11400   0.08910     15.9   0.80900        0.913
## alpha-ketoglutaric acid,      0.021200   -0.15700   0.19900     20.3   0.81500        0.913
## Glycerol; 57                  0.008380   -0.08230   0.09910     28.7   0.85600        0.944
## 4-Deoxytetronic acid; 33      0.015400   -0.17600   0.20700     22.2   0.87500        0.951
## Glyceryl-glycoside; 59       -0.008220   -0.14100   0.12400     20.8   0.90300        0.968
## Hydroxyproline; 64            0.007160   -0.16100   0.17600     20.6   0.93300        0.986
## Stearic acid, TMS; 2          0.001070   -0.03720   0.03930     17.4   0.95600        0.989
## Methionine, 2TMS; 16         -0.002780   -0.12000   0.11400     20.3   0.96300        0.989
## 2,4-Dihydroxybutanoic aci    -0.001350   -0.10500   0.10200     16.6   0.98000        0.993
## Pyroglutamic acid; 69        -0.000294   -0.15500   0.15400     22.9   0.99700        0.997
## [1] 0
```

```
## [1] 0
## [1] 0
##
##
## Table: log_Blood_TGA
##
## Name                          Effect        CI.L        CI.R   AveExpr    P.Value   adj.P.Val
## -------------------------  ---------  ----------  ----------  --------  ---------  ----------
## Palmitic acid, TMS; 5        0.07330    0.038700    0.108000      21.4   3.67e-05     0.00275
## Octanoic acid; 68            0.09840    0.049100    0.148000      24.2   9.84e-05     0.00281
## Dodecanoic acid; 54          0.16800    0.083000    0.253000      22.7   1.18e-04     0.00281
## Arachidic acid; 46           0.08330    0.040400    0.126000      21.8   1.50e-04     0.00281
## Stearic acid, TMS; 2         0.05230    0.025000    0.079700      17.4   1.90e-04     0.00285
## 2-Hydroxybutyric acid, 2T    0.18200    0.085200    0.280000      20.7   2.49e-04     0.00311
## Decanoic acid; 52            0.14600    0.065300    0.226000      22.4   4.03e-04     0.00432
## Oleic acid, TMS; 3           0.09070    0.033800    0.148000      17.1   1.85e-03     0.01730
## Isoleucine, 2TMS; 18         0.11500    0.036700    0.192000      26.0   3.99e-03     0.02800
## Valine, 2TMS; 20             0.07220    0.023000    0.121000      25.1   4.07e-03     0.02800
## Lactic acid; 29              0.07100    0.022600    0.119000      25.4   4.11e-03     0.02800
## Glyceryl-glycoside; 59       0.13700    0.041700    0.232000      20.8   4.87e-03     0.03040
## 4-Hydroxybenzeneacetic ac    0.21600    0.054400    0.377000      19.5   8.87e-03     0.04780
## Heptadecanoic acid; 60       0.09070    0.022800    0.159000      21.8   8.92e-03     0.04780
## Ribonic acid; 72             0.15700    0.037300    0.278000      20.0   1.03e-02     0.05170
## Arabinopyranose; 51          0.14100    0.031800    0.250000      20.2   1.15e-02     0.05380
## 3,4-Dihydroxybutanoic aci    0.09150    0.018800    0.164000      15.9   1.37e-02     0.06040
## Myristoleic acid; 65         0.17900    0.035400    0.323000      19.9   1.47e-02     0.06040
## Aminomalonic acid; 45       -0.10200   -0.184000   -0.019600      24.3   1.53e-02     0.06040
## Glutamic acid, 3TMS; 8       0.08650    0.015400    0.158000      23.0   1.72e-02     0.06440
## Pyruvic acid; 31             0.15400    0.025000    0.284000      19.6   1.95e-02     0.06510
## alpha-ketoglutaric acid,     0.15200    0.024400    0.279000      20.3   1.96e-02     0.06510
## Leucine, 2TMS; 19            0.08430    0.013300    0.155000      21.1   2.00e-02     0.06510
## Fumaric acid, 2TMS; 9        0.06150    0.007940    0.115000      24.1   2.45e-02     0.07430
## Heptadecanoic acid; 61       0.05530    0.007050    0.104000      23.4   2.48e-02     0.07430
## Arachidonic acid, TMS; 24    0.06300    0.005900    0.120000      22.7   3.07e-02     0.08840
## Malic acid, 3TMS; 11         0.07170    0.003380    0.140000      20.0   3.97e-02     0.11000
## Ribitol; 71                  0.08550    0.001740    0.169000      23.3   4.54e-02     0.12000
## Nonadecanoic acid; 66        0.07100    0.001170    0.141000      20.0   4.63e-02     0.12000
## Glyceric acid; 30           -0.07450   -0.149000   -0.000355      20.4   4.89e-02     0.12200
## Succinic acid, 2TMS; 7       0.04480   -0.000611    0.090100      22.7   5.32e-02     0.12900
## 4-Hydroxybutanoic acid; 4    0.02730   -0.000782    0.055400      27.3   5.67e-02     0.13300
## Proline, 2TMS; 21            0.06840   -0.002820    0.140000      20.8   5.98e-02     0.13300
## 1,3-Propanediol; 34          0.06930   -0.003010    0.142000      24.2   6.03e-02     0.13300
## 3-Indolepropionic acid; 4   -0.15800   -0.331000    0.014400      19.5   7.24e-02     0.15500
## Tartronic acid; 73          -0.10300   -0.221000    0.014800      21.3   8.64e-02     0.17400
## 4-Deoxytetronic acid; 33     0.11900   -0.017800    0.256000      22.2   8.80e-02     0.17400
## Glycine, 3TMS; 17           -0.04020   -0.086500    0.006040      21.4   8.82e-02     0.17400
## Linoleic acid, TMS; 4        0.03680   -0.009900    0.083500      25.7   1.22e-01     0.23500
## Myo inositol 6TMS; 1         0.05060   -0.015200    0.116000      19.1   1.32e-01     0.24700
## Eicosapentaenoic acid; 55   -0.08660   -0.200000    0.027100      23.0   1.35e-01     0.24700
## 3-Hydroxybutyric acid, 2T    0.08500   -0.031400    0.201000      21.7   1.52e-01     0.27100
## Serine, 3TMS; 14            -0.03690   -0.089700    0.015900      26.0   1.71e-01     0.29800
## Tridecanoic acid; 74         0.05050   -0.024900    0.126000      20.0   1.89e-01     0.32200
## 1-Dodecanol; 36              0.04090   -0.022100    0.104000      20.2   2.03e-01     0.33800
## Pyroglutamic acid; 69       -0.07030   -0.181000    0.040200      22.9   2.12e-01     0.33800
```

```
## Ribitol; 70                  0.05680   -0.032500   0.146000      20.2   2.12e-01     0.33800
## Hydroxylamine; 62           -0.04250   -0.113000   0.027800      27.7   2.35e-01     0.35400
## Cholesterol, TMS; 23         0.02480   -0.016300   0.065800      21.6   2.36e-01     0.35400
## Glycerol; 57                 0.03900   -0.025900   0.104000      28.7   2.39e-01     0.35400
## 3-Indoleacetic acid; 40      0.06380   -0.042800   0.170000      20.5   2.41e-01     0.35400
## 2,4-Dihydroxybutanoic aci    0.04240   -0.031500   0.116000      16.6   2.60e-01     0.37500
## Nonanoic acid; 67            0.03020   -0.026300   0.086600      24.4   2.94e-01     0.41600
## 2-hydroxy Isovaleric acid    0.08620   -0.082900   0.255000      22.2   3.17e-01     0.43300
## Tyrosine; 75                 0.08490   -0.081700   0.251000      23.3   3.17e-01     0.43300
## Ethanolamine; 56             0.02780   -0.030700   0.086300      22.7   3.51e-01     0.46900
## Alanine, 2TMS; 25            0.02380   -0.026800   0.074400      22.8   3.56e-01     0.46900
## 1-Monopalmitin; 37           0.02650   -0.034800   0.087700      29.2   3.97e-01     0.50900
## Phenylalanine, 2TMS; 13      0.01940   -0.025900   0.064600      24.3   4.01e-01     0.50900
## Glycerol; 58                -0.06480   -0.235000   0.106000      24.9   4.56e-01     0.57000
## Campesterol; 49             -0.03590   -0.133000   0.061400      21.8   4.68e-01     0.57600
## 11-Eicosenoic acid; 35       0.03510   -0.070500   0.141000      21.9   5.14e-01     0.62200
## Threonine, 3TMS; 12         -0.02220   -0.093000   0.048700      28.6   5.39e-01     0.64200
## Hydroxyproline; 64           0.03310   -0.087300   0.154000      20.6   5.89e-01     0.69100
## L-5-Oxoproline; 63           0.01340   -0.036600   0.063300      27.5   5.99e-01     0.69200
## Bisphenol A; 48             -0.01760   -0.092900   0.057600      21.2   6.45e-01     0.73300
## 2-Palmitoylglycerol; 39      0.01250   -0.043100   0.068000      25.9   6.59e-01     0.73800
## Citric acid, 4TMS; 6         0.01200   -0.051800   0.075900      20.8   7.11e-01     0.78000
## 4-Hydroxyphenyllactic aci    0.01540   -0.068200   0.099000      20.8   7.18e-01     0.78000
## Docosahexaenoic acid; 53    -0.01680   -0.113000   0.079800      24.2   7.33e-01     0.78500
## 4-Deoxytetronic acid; 32     0.01350   -0.080300   0.107000      21.6   7.78e-01     0.81500
## alpha-Tocopherol; 26         0.01310   -0.080000   0.106000      18.8   7.82e-01     0.81500
## Benzeneacetic acid; 47      -0.01270   -0.152000   0.126000      20.4   8.58e-01     0.88200
## Creatinine; 50              -0.00802   -0.111000   0.094600      21.6   8.78e-01     0.89000
## Methionine, 2TMS; 16         0.00301   -0.080800   0.086800      20.3   9.44e-01     0.94400
## [1] 7
## [1] 14
## [1] 26
##
##
## Table: Total_cholesterol
##
## Name                            Effect       CI.L         CI.R   AveExpr    P.Value   adj.P.Val
## --------------------------  ----------  ---------  -----------  --------  ---------  ----------
## Cholesterol, TMS; 23          0.152000    0.12200     0.183000      21.6   0.000000     0.00000
## Campesterol; 49               0.287000    0.21500     0.359000      21.8   0.000000     0.00000
## alpha-Tocopherol; 26          0.236000    0.16700     0.305000      18.8   0.000000     0.00000
## Benzeneacetic acid; 47       -0.197000   -0.30000    -0.093700      20.4   0.000193     0.00362
## Linoleic acid, TMS; 4         0.062900    0.02840     0.097500      25.7   0.000377     0.00566
## Isoleucine, 2TMS; 18         -0.101000   -0.15800    -0.043300      26.0   0.000623     0.00779
## 4-Hydroxybutanoic acid; 4    -0.035400   -0.05620    -0.014600      27.3   0.000877     0.00929
## L-5-Oxoproline; 63           -0.062300   -0.09930    -0.025300      27.5   0.000991     0.00929
## Eicosapentaenoic acid; 55     0.139000    0.05480     0.223000      23.0   0.001250     0.00978
## Proline, 2TMS; 21            -0.086700   -0.13900    -0.034000      20.8   0.001300     0.00978
## Tyrosine; 75                 -0.194000   -0.31700    -0.070500      23.3   0.002110     0.01320
## Methionine, 2TMS; 16         -0.097500   -0.16000    -0.035500      20.3   0.002120     0.01320
## Docosahexaenoic acid; 53      0.111000    0.03930     0.182000      24.2   0.002450     0.01410
## Glycine, 3TMS; 17            -0.052700   -0.08690    -0.018400      21.4   0.002650     0.01420
## Threonine, 3TMS; 12          -0.075100   -0.12800    -0.022700      28.6   0.005080     0.02540
## 4-Hydroxybenzeneacetic ac    -0.168000   -0.28800    -0.048800      19.5   0.005850     0.02590
```

```
## Alanine, 2TMS; 25          -0.052800  -0.09020  -0.015300     22.8  0.005860    0.02590
## 2,4-Dihydroxybutanoic aci  -0.075800  -0.13000  -0.021100     16.6  0.006650    0.02770
## Glyceryl-glycoside; 59     -0.094500  -0.16500  -0.024200     20.8  0.008470    0.03200
## Arabinopyranose; 51        -0.109000  -0.18900  -0.027700     20.2  0.008550    0.03200
## Malic acid, 3TMS; 11       -0.067500  -0.11800  -0.016900     20.0  0.009050    0.03230
## 2-Palmitoylglycerol; 39     0.051600   0.01040   0.092700     25.9  0.014100    0.04800
## Hydroxylamine; 62          -0.064600  -0.11700  -0.012600     27.7  0.014900    0.04870
## Serine, 3TMS; 14           -0.047300  -0.08640  -0.008220     26.0  0.017800    0.05550
## 3-Indoleacetic acid; 40    -0.093400  -0.17200  -0.014500     20.5  0.020500    0.06140
## Ribitol; 71                -0.072300  -0.13400  -0.010300     23.3  0.022300    0.06430
## Ribonic acid; 72           -0.102000  -0.19100  -0.013000     20.0  0.024800    0.06900
## 3,4-Dihydroxybutanoic aci  -0.055700  -0.11000  -0.001890     15.9  0.042500    0.11400
## Phenylalanine, 2TMS; 13    -0.033200  -0.06670   0.000232     24.3  0.051600    0.13300
## Pyruvic acid; 31           -0.092700  -0.18900   0.003160     19.6  0.058000    0.14500
## Palmitic acid, TMS; 5       0.024600  -0.00108   0.050200     21.4  0.060400    0.14600
## Ethanolamine; 56           -0.040600  -0.08390   0.002620     22.7  0.065600    0.15400
## Leucine, 2TMS; 19          -0.048200  -0.10100   0.004290     21.1  0.071800    0.16300
## 3-Indolepropionic acid; 4   0.112000  -0.01550   0.240000     19.5  0.084900    0.18700
## Creatinine; 50              0.063100  -0.01280   0.139000     21.6  0.103000    0.22000
## Valine, 2TMS; 20           -0.030000  -0.06640   0.006370     25.1  0.106000    0.22000
## 2-Hydroxybutyric acid, 2T   0.058100  -0.01380   0.130000     20.7  0.113000    0.22900
## 1-Dodecanol; 36            -0.035600  -0.08220   0.011000     20.2  0.134000    0.26500
## Oleic acid, TMS; 3          0.031400  -0.01070   0.073600     17.1  0.143000    0.27600
## 1,3-Propanediol; 34        -0.039100  -0.09260   0.014500     24.2  0.152000    0.28600
## 4-Deoxytetronic acid; 32   -0.050100  -0.12000   0.019300     21.6  0.157000    0.28600
## Nonanoic acid; 67          -0.028700  -0.07050   0.013100     24.4  0.178000    0.31800
## Aminomalonic acid; 45      -0.041100  -0.10200   0.019600     24.3  0.184000    0.32100
## Myo inositol 6TMS; 1       -0.032600  -0.08140   0.016100     19.1  0.189000    0.32200
## 11-Eicosenoic acid; 35     -0.051200  -0.12900   0.027000     21.9  0.199000    0.33200
## Citric acid, 4TMS; 6       -0.030500  -0.07780   0.016700     20.8  0.205000    0.33400
## Bisphenol A; 48            -0.031700  -0.08730   0.024000     21.2  0.264000    0.41700
## Pyroglutamic acid; 69      -0.046300  -0.12800   0.035500     22.9  0.267000    0.41700
## Myristoleic acid; 65        0.058500  -0.04810   0.165000     19.9  0.282000    0.43100
## Tridecanoic acid; 74       -0.030000  -0.08580   0.025800     20.0  0.291000    0.43300
## Glutamic acid, 3TMS; 8      0.027800  -0.02480   0.080400     23.0  0.300000    0.43300
## Fumaric acid, 2TMS; 9       0.020900  -0.01870   0.060600     24.1  0.300000    0.43300
## Heptadecanoic acid; 60      0.025200  -0.02500   0.075400     21.8  0.325000    0.46000
## Heptadecanoic acid; 61     -0.017400  -0.05310   0.018300     23.4  0.340000    0.47200
## Hydroxyproline; 64         -0.042400  -0.13200   0.046800     20.6  0.351000    0.47800
## Tartronic acid; 73          0.038900  -0.04820   0.126000     21.3  0.380000    0.50900
## Stearic acid, TMS; 2        0.008730  -0.01150   0.029000     17.4  0.397000    0.52300
## Lactic acid; 29            -0.014100  -0.04990   0.021700     25.4  0.440000    0.55700
## 2-hydroxy Isovaleric acid  -0.048900  -0.17400   0.076300     22.2  0.443000    0.55700
## Decanoic acid; 52           0.023100  -0.03640   0.082600     22.4  0.446000    0.55700
## alpha-ketoglutaric acid,   -0.031000  -0.12500   0.063300     20.3  0.519000    0.63800
## 3-Hydroxybutyric acid, 2T   0.027600  -0.05860   0.114000     21.7  0.529000    0.64000
## Octanoic acid; 68           0.008090  -0.02840   0.044600     24.2  0.663000    0.79000
## Nonadecanoic acid; 66       0.009250  -0.04250   0.060900     20.0  0.725000    0.85000
## Glyceric acid; 30          -0.007450  -0.06230   0.047400     20.4  0.790000    0.91100
## 4-Deoxytetronic acid; 33    0.010400  -0.09110   0.112000     22.2  0.841000    0.95500
## Dodecanoic acid; 54         0.005870  -0.05720   0.068900     22.7  0.855000    0.95700
## Ribitol; 70                 0.004020  -0.06210   0.070100     20.2  0.905000    0.97100
## 4-Hydroxyphenyllactic aci   0.003710  -0.05820   0.065600     20.8  0.906000    0.97100
## 1-Monopalmitin; 37         -0.002650  -0.04800   0.042700     29.2  0.909000    0.97100
```

```
## Succinic acid, 2TMS; 7        0.001660   -0.03190    0.035200      22.7   0.923000      0.97100
## Glycerol; 57                 -0.001720   -0.04970    0.046300      28.7   0.944000      0.97100
## Arachidonic acid, TMS; 24     0.000967   -0.04130    0.043300      22.7   0.964000      0.97100
## Glycerol; 58                  0.002500   -0.12400    0.129000      24.9   0.969000      0.97100
## Arachidic acid; 46           -0.000593   -0.03230    0.031100      21.8   0.971000      0.97100
## [1] 10
## [1] 23
## [1] 27
```

```
names.egfr.metabolites <-
  rownames( limma::topTable( fit=mEbFit, coef="egfr",
                            confint=TRUE, number=Inf, adjust.method="BH",
                            p.value = 0.01 ) )

names.egfr.clinical <- colnames( design.test )
```

1.2.2.2 Figures

1.2.2.2.1 Heatmap of Model Coefficients

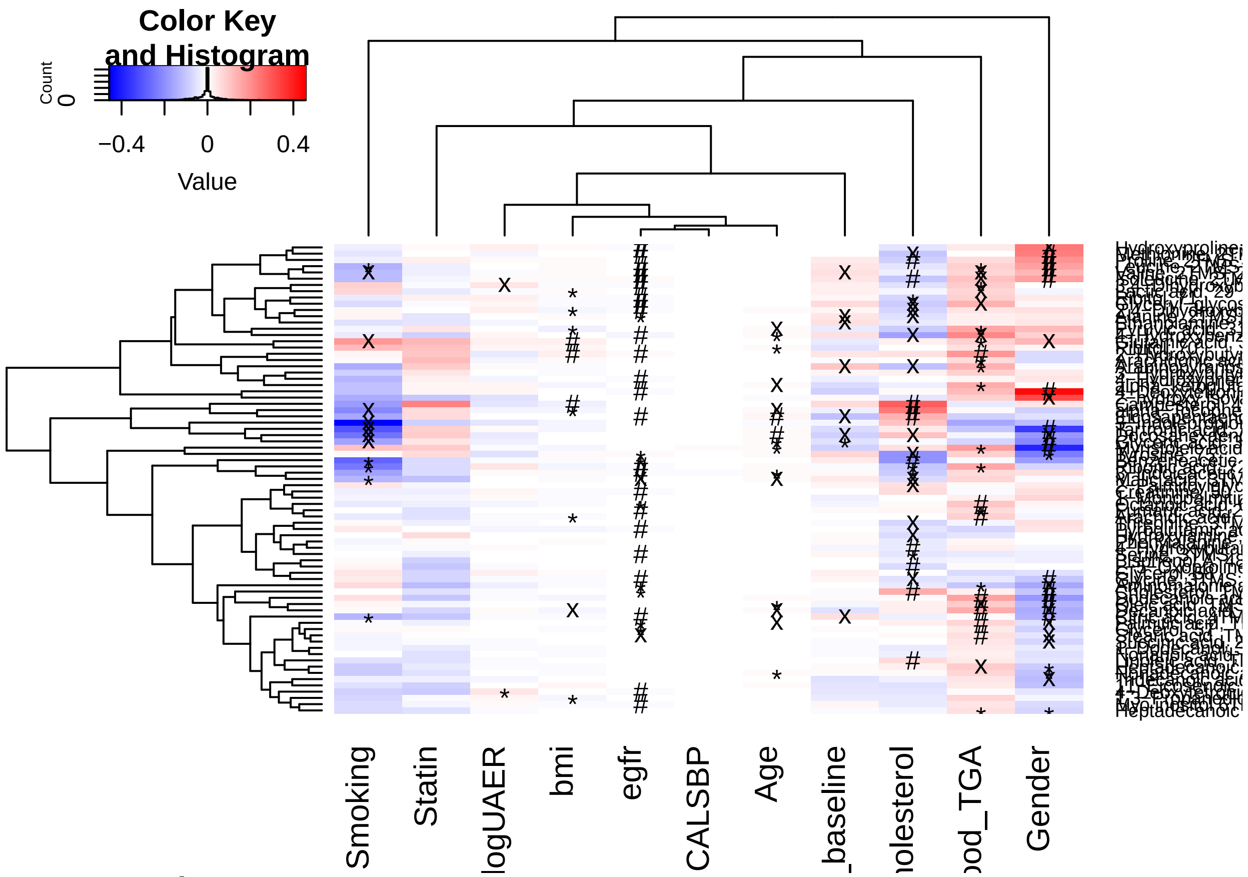
Color Key
and Histogram
Count
0
−0.4
0
0.4
Value
Smoking
Statin
logUAER
bmi
egfr
CALSBP
Age
_baseline
olesterol
ood_TGA
Gender

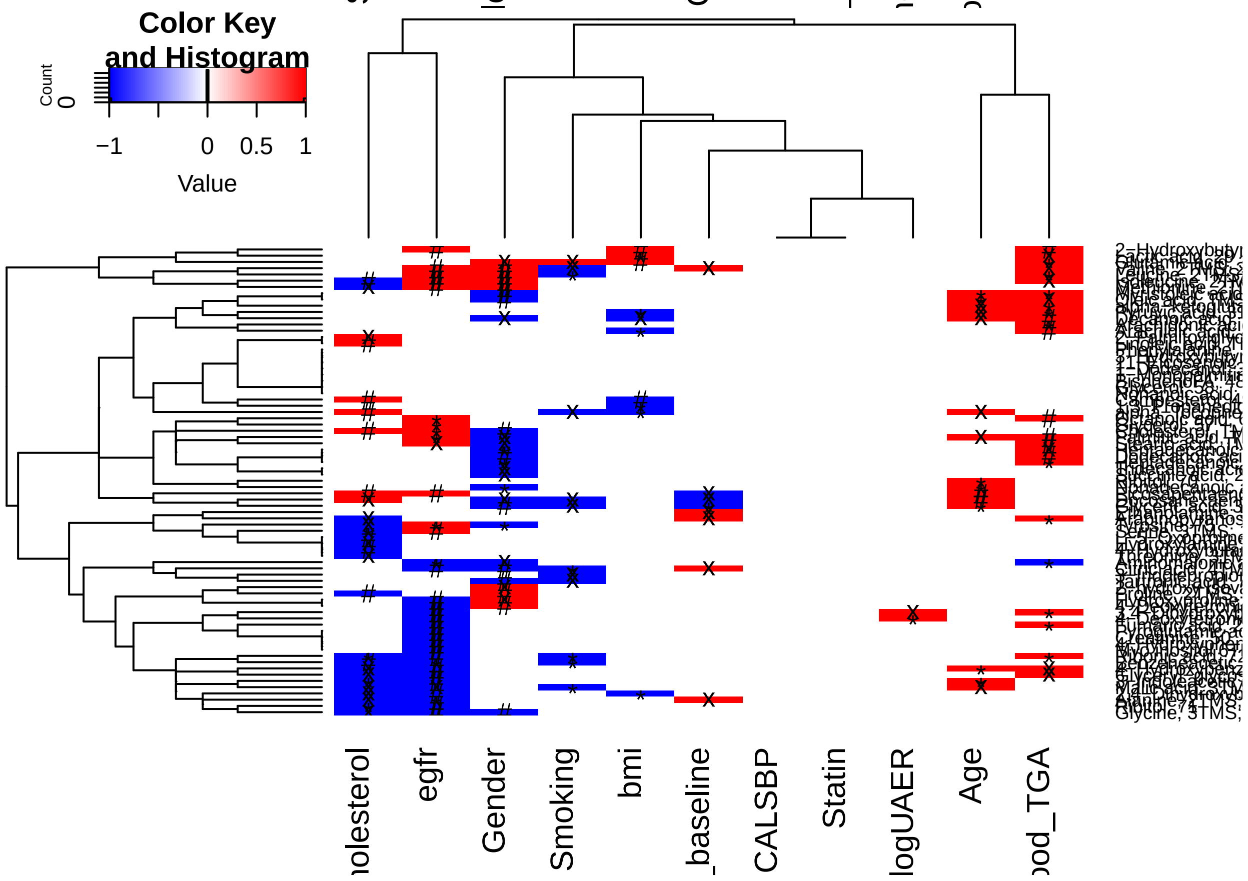
Color Key
and Histogram
Count
0
−1
0
0.5
1
Value
olesterol
egfr
Gender
Smoking
bmi
_baseline
CALSBP
Statin
logUAER
Age
ood_TGA

1.2.2.2.2 Bipartite Graph of Model Coefficients

```
## Warning: Removed 2 rows containing missing values (geom_segment).
```

```
## Warning: Removed 1 rows containing missing values (geom_text).
```

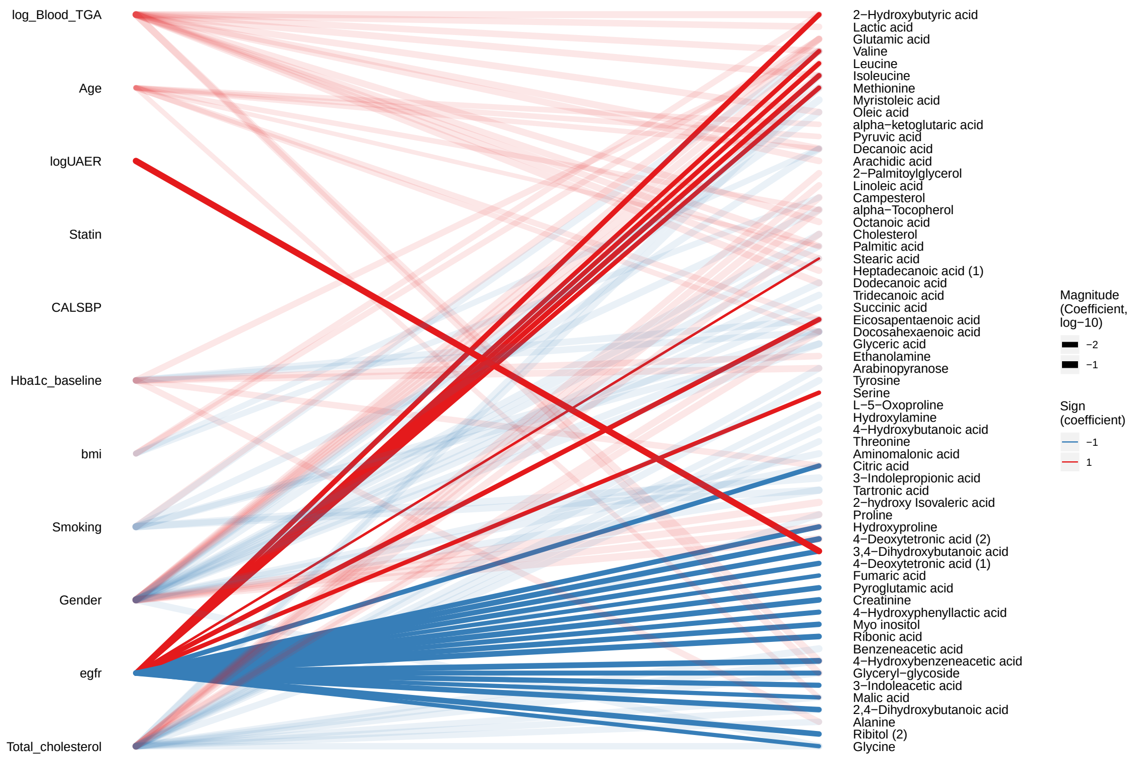


```
## Warning: Removed 2 rows containing missing values (geom_segment).
```

```
## Warning: Removed 1 rows containing missing values (geom_text).
```

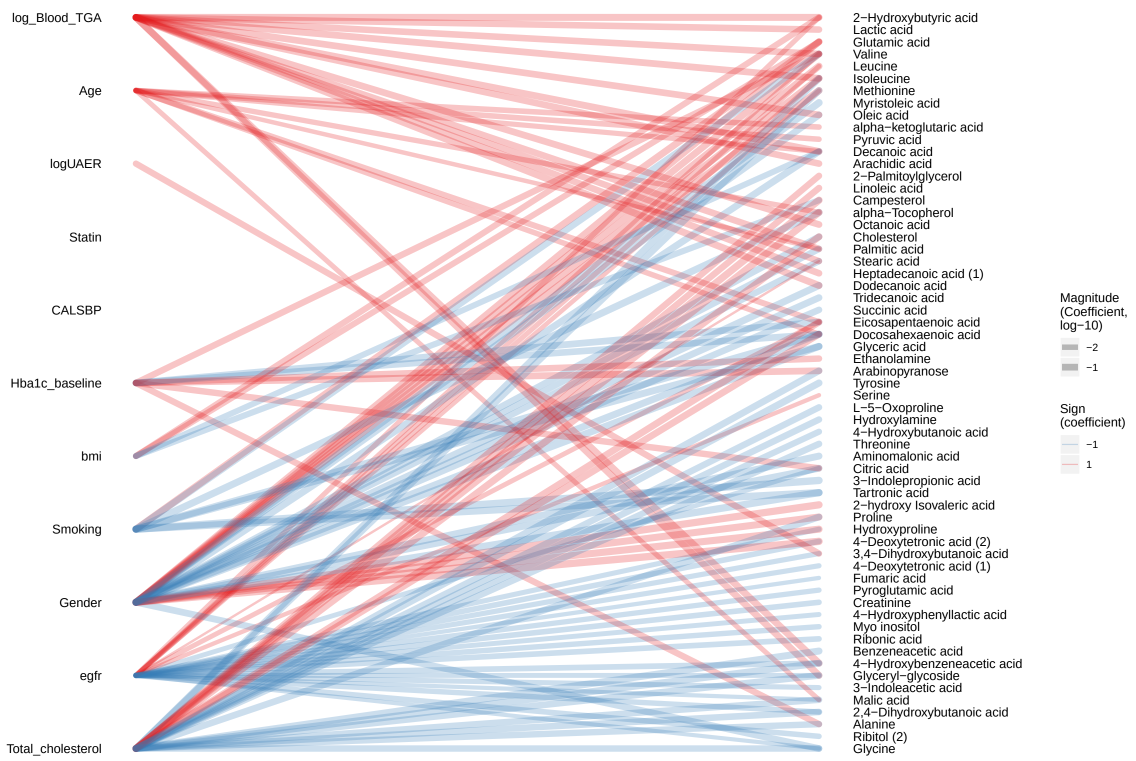
log_Blood_TGA
Age
logUAER
Statin
CALSBP
Hba1c_baseline
bmi
Smoking
Gender
egfr
Total_cholesterol
2−Hydroxybutyric acid
Lactic acid
Glutamic acid
Valine
Leucine
Isoleucine
Methionine
Myristoleic acid
Oleic acid
alpha−ketoglutaric acid
Pyruvic acid
Decanoic acid
Arachidic acid
2−Palmitoylglycerol
Linoleic acid
Campesterol
alpha−Tocopherol
Octanoic acid
Cholesterol
Palmitic acid
Stearic acid
Heptadecanoic acid (1)
Dodecanoic acid
Tridecanoic acid
Succinic acid
Eicosapentaenoic acid
Docosahexaenoic acid
Glyceric acid
Ethanolamine
Arabinopyranose
Tyrosine
Serine
L−5−Oxoproline
Hydroxylamine
4−Hydroxybutanoic acid
Threonine
Aminomalonic acid
Citric acid
3−Indolepropionic acid
Tartronic acid
2−hydroxy Isovaleric acid
Proline
Hydroxyproline
4−Deoxytetronic acid (2)
3,4−Dihydroxybutanoic acid
4−Deoxytetronic acid (1)
Fumaric acid
Pyroglutamic acid
Creatinine
4−Hydroxyphenyllactic acid
Myo inositol
Ribonic acid
Benzeneacetic acid
4−Hydroxybenzeneacetic acid
Glyceryl−glycoside
3−Indoleacetic acid
Malic acid
2,4−Dihydroxybutanoic acid
Alanine
Ribitol (2)
Glycine
Magnitude
(Coefficient,
log−10)
−2
−1
Sign
(coefficient)
−1
1

1.2.2.2.3 Forest Plot of Model Coefficients

```
## Warning: Ignoring unknown aesthetics: x
```

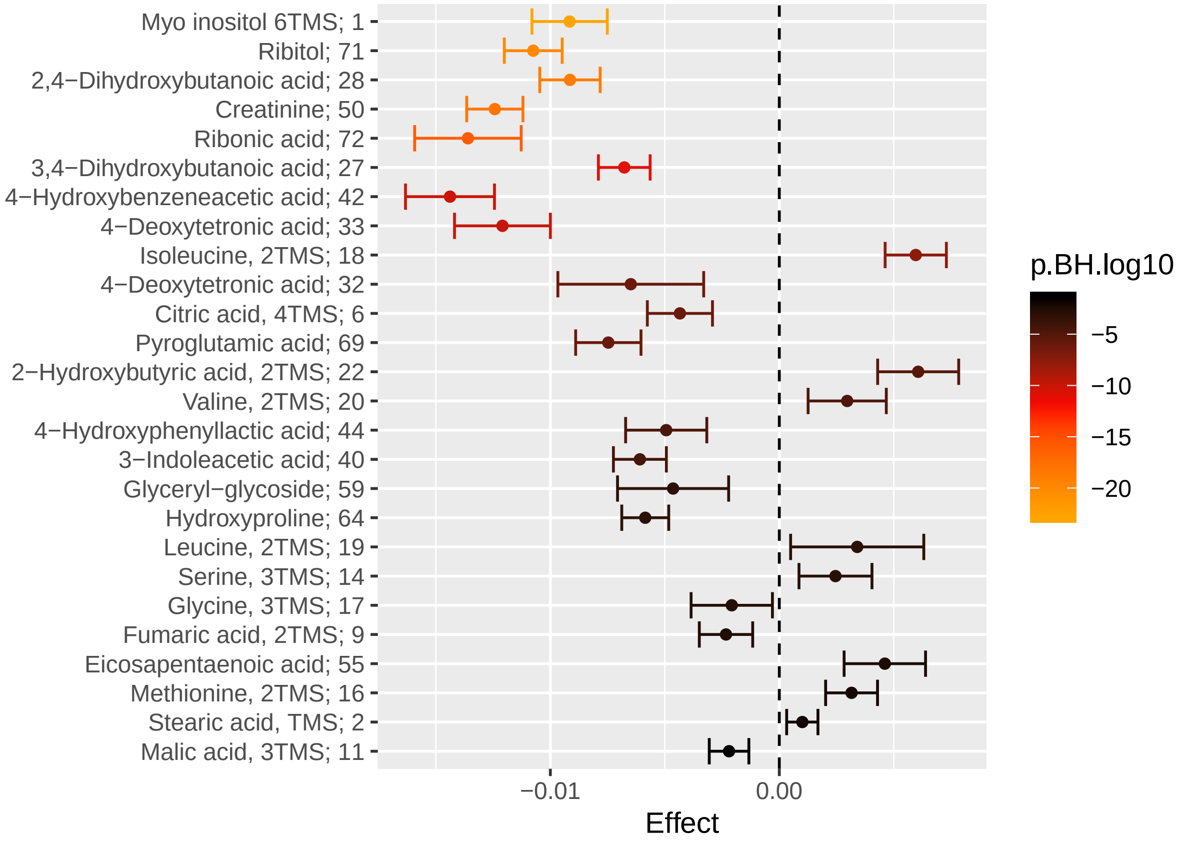

1.2.3 Technical-Adjusted Model

```
## [1] 585  81

##      (Intercept)      egfr   Age Gender Hba1c_baseline    logUAER CALSBP
## [1,]           1  11.03376 19.39      0            5.2  0.5849625     92
## [2,]           1 167.62905 85.23      1           15.0 13.0138461    191
##        bmi Smoking Statin log_Blood_TGA Total_cholesterol
## [1,] 16.98       0      0     -2.643856               2.3
## [2,] 43.29       1      1      2.720278               9.2
##      Batch.Manual[121,151) Batch.Manual[151,176) Batch.Manual[176,191)
## [1,]                     0                     0                     0
## [2,]                     1                     1                     1
##      Batch.Manual[191,206) Batch.Manual[206,225) Batch.Manual[225,248)
## [1,]                     0                     0                     0
## [2,]                     1                     1                     1
##      Batch.Manual[248,279) Batch.Manual[279,322) Batch.Manual[322,348)
## [1,]                     0                     0                     0
## [2,]                     1                     1                     1
##      Batch.Manual[348,369) Batch.Manual[369,381) Batch.Manual[381,390)
## [1,]                     0                     0                     0
## [2,]                     1                     1                     1
##      Batch.Manual[390,420) Batch.Manual[420,464) Batch.Manual[464,474)
## [1,]                     0                     0                     0
## [2,]                     1                     1                     1
##      Batch.Manual[474,479) Batch.Manual[479,485) Batch.Manual[485,501)
## [1,]                     0                     0                     0
## [2,]                     1                     1                     1
##      Batch.Manual[501,535) Batch.Manual[535,546) Batch.Manual[546,591)
## [1,]                     0                     0                     0
## [2,]                     1                     1                     1
##      Batch.Manual[591,594) Batch.Manual[594,635) Batch.Manual[635,649)
## [1,]                     0                     0                     0
## [2,]                     1                     1                     1
##      Batch.Manual[680,694) Batch.Manual[694,751) Batch.Manual[751,766)
## [1,]                     0                     0                     0
## [2,]                     1                     1                     1
##      Batch.Manual[766,807) Batch.Manual[807,891) Batch.Manual[83,121)
## [1,]                     0                     0                    0
## [2,]                     1                     1                    1
##      Batch.Manual[891,907) Batch.Manual[907,917) Batch.Manual[917,941)
## [1,]                     0                     0                     0
## [2,]                     1                     1                     1
##      Batch.Manual[941, Inf) Run.Number Batch.Manual[121,151):Run.Number
## [1,]                      0         75                                0
## [2,]                      1        949                              150
##      Batch.Manual[151,176):Run.Number Batch.Manual[176,191):Run.Number
## [1,]                                0                                0
## [2,]                              175                              190
##      Batch.Manual[191,206):Run.Number Batch.Manual[206,225):Run.Number
## [1,]                                0                                0
## [2,]                              205                              223
##      Batch.Manual[225,248):Run.Number Batch.Manual[248,279):Run.Number
## [1,]                                0                                0
```

```
## [2,]                             245                              278
##      Batch.Manual[279,322):Run.Number Batch.Manual[322,348):Run.Number
## [1,]                               0                                0
## [2,]                             321                              347
##      Batch.Manual[348,369):Run.Number Batch.Manual[369,381):Run.Number
## [1,]                               0                                0
## [2,]                             368                              380
##      Batch.Manual[381,390):Run.Number Batch.Manual[390,420):Run.Number
## [1,]                               0                                0
## [2,]                             389                              419
##      Batch.Manual[420,464):Run.Number Batch.Manual[464,474):Run.Number
## [1,]                               0                                0
## [2,]                             460                              473
##      Batch.Manual[474,479):Run.Number Batch.Manual[479,485):Run.Number
## [1,]                               0                                0
## [2,]                             478                              484
##      Batch.Manual[485,501):Run.Number Batch.Manual[501,535):Run.Number
## [1,]                               0                                0
## [2,]                             500                              533
##      Batch.Manual[535,546):Run.Number Batch.Manual[546,591):Run.Number
## [1,]                               0                                0
## [2,]                             545                              590
##      Batch.Manual[591,594):Run.Number Batch.Manual[594,635):Run.Number
## [1,]                               0                                0
## [2,]                             593                              634
##      Batch.Manual[635,649):Run.Number Batch.Manual[680,694):Run.Number
## [1,]                               0                                0
## [2,]                             647                              693
##      Batch.Manual[694,751):Run.Number Batch.Manual[751,766):Run.Number
## [1,]                               0                                0
## [2,]                             750                              765
##      Batch.Manual[766,807):Run.Number Batch.Manual[807,891):Run.Number
## [1,]                               0                                0
## [2,]                             804                              888
##      Batch.Manual[83,121):Run.Number Batch.Manual[891,907):Run.Number
## [1,]                              0                                0
## [2,]                            119                              906
##      Batch.Manual[907,917):Run.Number Batch.Manual[917,941):Run.Number
## [1,]                               0                                0
## [2,]                             916                              940
##      Batch.Manual[941, Inf):Run.Number
## [1,]                                0
## [2,]                              949
```

```
##
##                                Overall
##   n                               585
##   egfr (mean (sd))              82.04 (28.26)
##   Age (mean (sd))               55.38 (12.12)
##   Gender (mean (sd))             0.54 (0.50)
##   Hba1c_baseline (mean (sd))     8.03 (1.16)
##   logUAER (mean (sd))            4.73 (2.31)
##   CALSBP (mean (sd))           131.91 (17.50)
##   bmi (mean (sd))               25.19 (4.05)
```

```
##   Smoking (mean (sd))             0.21 (0.41)
##   Statin (mean (sd))              0.61 (0.49)
##   log_Blood_TGA (mean (sd))       0.01 (0.69)
##   Total_cholesterol (mean (sd))   4.69 (0.88)
##   Batch.Manual (%)
##      [-Inf,83)                      7 (1.2)
##      [121,151)                     18 (3.1)
##      [151,176)                     18 (3.1)
##      [176,191)                     13 (2.2)
##      [191,206)                     10 (1.7)
##      [206,225)                     16 (2.7)
##      [225,248)                     16 (2.7)
##      [248,279)                     22 (3.8)
##      [279,322)                     30 (5.1)
##      [322,348)                     16 (2.7)
##      [348,369)                     14 (2.4)
##      [369,381)                     10 (1.7)
##      [381,390)                      8 (1.4)
##      [390,420)                     20 (3.4)
##      [420,464)                     23 (3.9)
##      [464,474)                      7 (1.2)
##      [474,479)                      3 (0.5)
##      [479,485)                      4 (0.7)
##      [485,501)                     10 (1.7)
##      [501,535)                     17 (2.9)
##      [535,546)                      8 (1.4)
##      [546,591)                     22 (3.8)
##      [591,594)                      3 (0.5)
##      [594,635)                     24 (4.1)
##      [635,649)                      9 (1.5)
##      [680,694)                     12 (2.1)
##      [694,751)                     40 (6.8)
##      [751,766)                     13 (2.2)
##      [766,807)                     24 (4.1)
##      [807,891)                     53 (9.1)
##      [83,121)                      26 (4.4)
##      [891,907)                     23 (3.9)
##      [907,917)                     16 (2.7)
##      [917,941)                     23 (3.9)
##      [941, Inf)                     7 (1.2)
##   Run.Number (mean (sd))        519.35 (269.10)
```

1.2.3.1 Table

```
##
##
## Table: egfr
##
## Name                            Effect        CI.L        CI.R   AveExpr    P.Value   adj.P.Val
## --------------------------  ----------  ----------  ----------  --------  ---------  ----------
## Myo inositol 6TMS; 1         -9.48e-03   -1.12e-02   -0.007780      19.1   0.00e+00    0.00e+00
## Creatinine; 50               -1.33e-02   -1.59e-02   -0.010800      21.6   0.00e+00    0.00e+00
## Ribitol; 71                  -1.10e-02   -1.31e-02   -0.008790      23.3   0.00e+00    0.00e+00
## 2,4-Dihydroxybutanoic aci    -9.36e-03   -1.12e-02   -0.007480      16.6   0.00e+00    0.00e+00
## Ribonic acid; 72             -1.46e-02   -1.77e-02   -0.011500      20.0   0.00e+00    0.00e+00
## 3,4-Dihydroxybutanoic aci    -7.09e-03   -8.95e-03   -0.005220      15.9   0.00e+00    0.00e+00
## 4-Hydroxybenzeneacetic ac    -1.55e-02   -1.97e-02   -0.011300      19.5   0.00e+00    0.00e+00
## 4-Deoxytetronic acid; 33     -1.29e-02   -1.64e-02   -0.009330      22.2   0.00e+00    0.00e+00
## 4-Deoxytetronic acid; 32     -7.50e-03   -9.92e-03   -0.005080      21.6   0.00e+00    0.00e+00
## Pyroglutamic acid; 69        -7.61e-03   -1.03e-02   -0.004880      22.9   1.00e-07    5.00e-07
## Citric acid, 4TMS; 6         -4.47e-03   -6.09e-03   -0.002840      20.8   1.00e-07    7.00e-07
## Isoleucine, 2TMS; 18          5.48e-03    3.45e-03    0.007510      26.0   2.00e-07    1.10e-06
## 2-Hydroxybutyric acid, 2T     5.98e-03    3.42e-03    0.008540      20.7   5.60e-06    3.26e-05
## Valine, 2TMS; 20              3.01e-03    1.71e-03    0.004310      25.1   7.10e-06    3.81e-05
## 4-Hydroxyphenyllactic aci    -4.73e-03   -6.89e-03   -0.002570      20.8   1.98e-05    9.89e-05
## 3-Indoleacetic acid; 40      -6.03e-03   -8.81e-03   -0.003250      20.5   2.39e-05    1.12e-04
## Serine, 3TMS; 14              2.92e-03    1.54e-03    0.004310      26.0   4.01e-05    1.77e-04
## Glyceryl-glycoside; 59       -4.74e-03   -7.05e-03   -0.002420      20.8   6.88e-05    2.87e-04
## Leucine, 2TMS; 19             3.58e-03    1.74e-03    0.005430      21.1   1.51e-04    5.94e-04
## Methionine, 2TMS; 16          3.88e-03    1.73e-03    0.006030      20.3   4.21e-04    1.58e-03
## Hydroxyproline; 64           -5.52e-03   -8.70e-03   -0.002350      20.6   6.83e-04    2.44e-03
## Eicosapentaenoic acid; 55     4.83e-03    1.87e-03    0.007780      23.0   1.41e-03    4.82e-03
## Fumaric acid, 2TMS; 9        -2.22e-03   -3.60e-03   -0.000840      24.1   1.67e-03    5.43e-03
## Glycine, 3TMS; 17            -1.81e-03   -3.01e-03   -0.000623      21.4   2.91e-03    9.08e-03
## Stearic acid, TMS; 2          1.02e-03    3.13e-04    0.001730      17.4   4.83e-03    1.45e-02
## Malic acid, 3TMS; 11         -2.33e-03   -4.13e-03   -0.000535      20.0   1.11e-02    3.21e-02
## 2-hydroxy Isovaleric acid     5.66e-03    1.21e-03    0.010100      22.2   1.29e-02    3.57e-02
## Palmitic acid, TMS; 5         1.12e-03    2.20e-04    0.002030      21.4   1.49e-02    4.00e-02
## Glycerol; 57                  2.08e-03    3.83e-04    0.003780      28.7   1.64e-02    4.23e-02
## Succinic acid, 2TMS; 7       -1.35e-03   -2.49e-03   -0.000215      22.7   1.99e-02    4.97e-02
## Tyrosine; 75                  5.06e-03    7.38e-04    0.009380      23.3   2.18e-02    5.28e-02
## Benzeneacetic acid; 47       -4.05e-03   -7.67e-03   -0.000419      20.4   2.89e-02    6.77e-02
## Aminomalonic acid; 45        -2.43e-03   -4.63e-03   -0.000227      24.3   3.07e-02    6.97e-02
## Octanoic acid; 68             1.40e-03    8.75e-05    0.002710      24.2   3.66e-02    8.07e-02
## 2-Palmitoylglycerol; 39       1.39e-03    5.60e-06    0.002770      25.9   4.91e-02    1.05e-01
## Cholesterol, TMS; 23          1.01e-03   -8.50e-06    0.002030      21.6   5.19e-02    1.08e-01
## Threonine, 3TMS; 12           1.65e-03   -1.19e-04    0.003410      28.6   6.75e-02    1.37e-01
## Alanine, 2TMS; 25            -1.20e-03   -2.54e-03    0.000135      22.8   7.79e-02    1.54e-01
## Glycerol; 58                 -4.02e-03   -8.59e-03    0.000555      24.9   8.49e-02    1.63e-01
## Decanoic acid; 52             1.76e-03   -2.84e-04    0.003810      22.4   9.13e-02    1.71e-01
## Glutamic acid, 3TMS; 8        1.41e-03   -3.90e-04    0.003220      23.0   1.24e-01    2.27e-01
## Myristoleic acid; 65          2.79e-03   -9.80e-04    0.006560      19.9   1.47e-01    2.62e-01
## Campesterol; 49              -1.88e-03   -4.46e-03    0.000708      21.8   1.54e-01    2.69e-01
## Hydroxylamine; 62            -1.30e-03   -3.12e-03    0.000516      27.7   1.60e-01    2.73e-01
## Docosahexaenoic acid; 53      1.62e-03   -7.59e-04    0.003990      24.2   1.82e-01    3.03e-01
## Oleic acid, TMS; 3            8.26e-04   -6.74e-04    0.002330      17.1   2.80e-01    4.56e-01
```

```
## Glyceric acid; 30            9.72e-04   -9.53e-04    0.002900       20.4   3.22e-01    5.13e-01
## Phenylalanine, 2TMS; 13     -5.56e-04   -1.72e-03    0.000607       24.3   3.48e-01    5.44e-01
## alpha-Tocopherol; 26         1.12e-03   -1.33e-03    0.003560       18.8   3.70e-01    5.66e-01
## Nonanoic acid; 67           -4.88e-04   -1.97e-03    0.000994       24.4   5.18e-01    7.77e-01
## 1-Monopalmitin; 37           4.68e-04   -1.15e-03    0.002090       29.2   5.71e-01    8.39e-01
## Ribitol; 70                 -6.54e-04   -3.01e-03    0.001700       20.2   5.86e-01    8.45e-01
## Bisphenol A; 48              4.79e-04   -1.39e-03    0.002350       21.2   6.15e-01    8.69e-01
## Heptadecanoic acid; 60      -4.26e-04   -2.18e-03    0.001320       21.8   6.32e-01    8.69e-01
## Dodecanoic acid; 54          5.26e-04   -1.75e-03    0.002800       22.7   6.49e-01    8.69e-01
## 3-Indolepropionic acid; 4    1.05e-03   -3.50e-03    0.005610       19.5   6.50e-01    8.69e-01
## Proline, 2TMS; 21            4.22e-04   -1.47e-03    0.002310       20.8   6.61e-01    8.69e-01
## 3-Hydroxybutyric acid, 2T    6.32e-04   -2.46e-03    0.003720       21.7   6.88e-01    8.82e-01
## Ethanolamine; 56             3.07e-04   -1.23e-03    0.001840       22.7   6.94e-01    8.82e-01
## Nonadecanoic acid; 66        3.43e-04   -1.49e-03    0.002180       20.0   7.13e-01    8.85e-01
## 1-Dodecanol; 36              2.83e-04   -1.32e-03    0.001890       20.2   7.30e-01    8.85e-01
## Linoleic acid, TMS; 4        2.16e-04   -1.02e-03    0.001450       25.7   7.32e-01    8.85e-01
## 11-Eicosenoic acid; 35      -3.78e-04   -3.12e-03    0.002360       21.9   7.87e-01    9.24e-01
## Arachidonic acid, TMS; 24    1.98e-04   -1.26e-03    0.001650       22.7   7.89e-01    9.24e-01
## Lactic acid; 29              1.55e-04   -1.12e-03    0.001430       25.4   8.11e-01    9.36e-01
## Arabinopyranose; 51         -3.05e-04   -3.17e-03    0.002560       20.2   8.34e-01    9.48e-01
## Tartronic acid; 73          -2.52e-04   -3.31e-03    0.002810       21.3   8.71e-01    9.75e-01
## alpha-ketoglutaric acid,    -1.88e-04   -3.33e-03    0.002950       20.3   9.07e-01    9.78e-01
## Arachidic acid; 46           6.20e-05   -1.05e-03    0.001180       21.8   9.13e-01    9.78e-01
## L-5-Oxoproline; 63          -7.00e-05   -1.35e-03    0.001210       27.5   9.14e-01    9.78e-01
## Pyruvic acid; 31             1.61e-04   -3.26e-03    0.003580       19.6   9.26e-01    9.78e-01
## 4-Hydroxybutanoic acid; 4   -1.44e-05   -7.22e-04    0.000693       27.3   9.68e-01    9.97e-01
## 1,3-Propanediol; 34         -1.76e-05   -1.93e-03    0.001890       24.2   9.86e-01    9.97e-01
## Tridecanoic acid; 74         1.74e-05   -1.90e-03    0.001940       20.0   9.86e-01    9.97e-01
## Heptadecanoic acid; 61      -2.20e-06   -1.28e-03    0.001280       23.4   9.97e-01    9.97e-01
## [1] 24
## [1] 30
## [1] 34
```

1.3 logUAER – Continuous Albuminuria

1.3.1 Crude Model

```
## [1] 586   2

##      (Intercept)    logUAER
## [1,]           1  0.5849625
## [2,]           1 13.0138461

##
##                                 Overall
##   n                               586
##   egfr (mean (sd))              82.04 (28.24)
##   Age (mean (sd))               55.37 (12.11)
##   Gender (mean (sd))             0.54 (0.50)
##   Hba1c_baseline (mean (sd))     8.03 (1.16)
##   logUAER (mean (sd))            4.73 (2.31)
##   CALSBP (mean (sd))           131.91 (17.48)
##   bmi (mean (sd))               25.20 (4.06)
##   Smoking (mean (sd))            0.21 (0.41)
##   Statin (mean (sd))             0.61 (0.49)
##   log_Blood_TGA (mean (sd))      0.01 (0.69)
##   Total_cholesterol (mean (sd))   4.69 (0.88)
```

1.3.1.1 Table

```
##
##
## Table: logUAER
##
## Name                            Effect       CI.L        CI.R   AveExpr    P.Value   adj.P.Val
## -------------------------  ----------  ---------  ----------  --------  ---------  ----------
## 3,4-Dihydroxybutanoic aci     0.080600    0.06070    0.100000      15.9   0.00e+00    0.00e+00
## 2,4-Dihydroxybutanoic aci     0.064700    0.04360    0.085900      16.6   0.00e+00    1.00e-07
## Ribitol; 71                   0.072500    0.04850    0.096600      23.3   0.00e+00    1.00e-07
## Ribonic acid; 72              0.098600    0.06430    0.133000      20.0   0.00e+00    4.00e-07
## Glyceric acid; 30            -0.055300   -0.07510   -0.035500      20.4   1.00e-07    9.00e-07
## Myo inositol 6TMS; 1          0.050100    0.03080    0.069400      19.1   5.00e-07    5.90e-06
## 4-Deoxytetronic acid; 32      0.053800    0.02890    0.078700      21.6   2.50e-05    2.42e-04
## 4-Hydroxybenzeneacetic ac     0.096100    0.05160    0.141000      19.5   2.58e-05    2.42e-04
## Docosahexaenoic acid; 53     -0.055300   -0.08130   -0.029300      24.2   3.35e-05    2.79e-04
## Creatinine; 50                0.059000    0.03050    0.087500      21.6   5.37e-05    3.89e-04
## 3-Indolepropionic acid; 4    -0.092600   -0.13700   -0.047800      19.5   5.70e-05    3.89e-04
## Glutamic acid, 3TMS; 8        0.038300    0.01900    0.057600      23.0   1.06e-04    6.62e-04
## 4-Deoxytetronic acid; 33      0.070100    0.03280    0.107000      22.2   2.44e-04    1.41e-03
## Octanoic acid; 68            -0.023800   -0.03660   -0.010900      24.2   2.99e-04    1.60e-03
## Hydroxyproline; 64            0.057100    0.02580    0.088400      20.6   3.64e-04    1.71e-03
## Aminomalonic acid; 45        -0.039600   -0.06130   -0.017900      24.3   3.66e-04    1.71e-03
## Glyceryl-glycoside; 59        0.045300    0.02030    0.070200      20.8   3.93e-04    1.73e-03
## Tyrosine; 75                 -0.070000   -0.11300   -0.026800      23.3   1.54e-03    6.41e-03
## 2-hydroxy Isovaleric acid    -0.069400   -0.11300   -0.025600      22.2   1.96e-03    7.75e-03
## Ribitol; 70                   0.032100    0.00880    0.055400      20.2   6.99e-03    2.62e-02
## L-5-Oxoproline; 63           -0.017700   -0.03060   -0.004770      27.5   7.40e-03    2.64e-02
## Methionine, 2TMS; 16         -0.030200   -0.05240   -0.007910      20.3   7.98e-03    2.72e-02
## Proline, 2TMS; 21             0.025300    0.00633    0.044200      20.8   9.02e-03    2.94e-02
## Cholesterol, TMS; 23         -0.014800   -0.02670   -0.002940      21.6   1.46e-02    4.55e-02
## Valine, 2TMS; 20             -0.016800   -0.03050   -0.003160      25.1   1.59e-02    4.77e-02
## Tridecanoic acid; 74         -0.022900   -0.04250   -0.003420      20.0   2.13e-02    6.15e-02
## alpha-Tocopherol; 26         -0.029200   -0.05460   -0.003780      18.8   2.44e-02    6.79e-02
## Eicosapentaenoic acid; 55    -0.034300   -0.06560   -0.002940      23.0   3.21e-02    8.59e-02
## Heptadecanoic acid; 61       -0.013500   -0.02600   -0.000970      23.4   3.47e-02    8.98e-02
## Tartronic acid; 73           -0.031700   -0.06320   -0.000181      21.3   4.87e-02    1.22e-01
## 1-Dodecanol; 36              -0.015800   -0.03190    0.000410      20.2   5.61e-02    1.34e-01
## Serine, 3TMS; 14             -0.013400   -0.02720    0.000406      26.0   5.71e-02    1.34e-01
## Arabinopyranose; 51           0.027300   -0.00128    0.055900      20.2   6.11e-02    1.39e-01
## 2-Hydroxybutyric acid, 2T    -0.025300   -0.05200    0.001440      20.7   6.37e-02    1.40e-01
## Threonine, 3TMS; 12          -0.015600   -0.03390    0.002710      28.6   9.47e-02    2.03e-01
## Glycerol; 58                  0.036600   -0.00699    0.080200      24.9   9.97e-02    2.05e-01
## Linoleic acid, TMS; 4        -0.010200   -0.02240    0.002060      25.7   1.03e-01    2.05e-01
## 3-Indoleacetic acid; 40       0.023600   -0.00486    0.052100      20.5   1.04e-01    2.05e-01
## Campesterol; 49              -0.018900   -0.04560    0.007800      21.8   1.65e-01    3.17e-01
## Stearic acid, TMS; 2         -0.004880   -0.01200    0.002290      17.4   1.82e-01    3.39e-01
## Heptadecanoic acid; 60       -0.011900   -0.02950    0.005730      21.8   1.86e-01    3.39e-01
## Arachidic acid; 46           -0.007460   -0.01860    0.003700      21.8   1.90e-01    3.39e-01
## Ethanolamine; 56             -0.008830   -0.02400    0.006330      22.7   2.53e-01    4.39e-01
## Fumaric acid, 2TMS; 9         0.008100   -0.00594    0.022100      24.1   2.57e-01    4.39e-01
## Arachidonic acid, TMS; 24    -0.008360   -0.02320    0.006460      22.7   2.68e-01    4.39e-01
## 4-Hydroxybutanoic acid; 4     0.004090   -0.00318    0.011400      27.3   2.69e-01    4.39e-01
```

```
## Palmitic acid, TMS; 5        -0.003960   -0.01320    0.005240      21.4   3.99e-01    6.24e-01
## Alanine, 2TMS; 25             0.005710   -0.00758    0.019000      22.8   3.99e-01    6.24e-01
## Succinic acid, 2TMS; 7       -0.004900   -0.01670    0.006910      22.7   4.15e-01    6.25e-01
## Lactic acid; 29              -0.005240   -0.01790    0.007460      25.4   4.18e-01    6.25e-01
## Phenylalanine, 2TMS; 13       0.004620   -0.00706    0.016300      24.3   4.38e-01    6.25e-01
## Nonadecanoic acid; 66        -0.007130   -0.02530    0.011000      20.0   4.41e-01    6.25e-01
## Glycine, 3TMS; 17            -0.004860   -0.01730    0.007540      21.4   4.42e-01    6.25e-01
## Hydroxylamine; 62             0.006870   -0.01120    0.025000      27.7   4.57e-01    6.34e-01
## 3-Hydroxybutyric acid, 2T     0.010900   -0.01900    0.040700      21.7   4.75e-01    6.48e-01
## 4-Hydroxyphenyllactic aci     0.007820   -0.01440    0.030000      20.8   4.89e-01    6.53e-01
## Malic acid, 3TMS; 11          0.006230   -0.01170    0.024200      20.0   4.96e-01    6.53e-01
## Isoleucine, 2TMS; 18         -0.007140   -0.02860    0.014300      26.0   5.14e-01    6.65e-01
## Bisphenol A; 48              -0.006140   -0.02540    0.013100      21.2   5.32e-01    6.76e-01
## Nonanoic acid; 67            -0.003850   -0.01830    0.010600      24.4   6.02e-01    7.43e-01
## 1,3-Propanediol; 34           0.004920   -0.01370    0.023600      24.2   6.04e-01    7.43e-01
## Decanoic acid; 52            -0.005170   -0.02650    0.016100      22.4   6.34e-01    7.67e-01
## Glycerol; 57                 -0.003750   -0.02050    0.013000      28.7   6.60e-01    7.85e-01
## Benzeneacetic acid; 47       -0.007690   -0.04430    0.028900      20.4   6.80e-01    7.97e-01
## alpha-ketoglutaric acid,      0.006250   -0.02690    0.039400      20.3   7.11e-01    8.21e-01
## Pyruvic acid; 31             -0.004170   -0.03790    0.029600      19.6   8.08e-01    9.19e-01
## Pyroglutamic acid; 69         0.002820   -0.02650    0.032200      22.9   8.50e-01    9.40e-01
## Oleic acid, TMS; 3            0.001360   -0.01360    0.016400      17.1   8.58e-01    9.40e-01
## Dodecanoic acid; 54          -0.001950   -0.02440    0.020500      22.7   8.65e-01    9.40e-01
## 2-Palmitoylglycerol; 39      -0.001130   -0.01550    0.013200      25.9   8.77e-01    9.40e-01
## Leucine, 2TMS; 19            -0.001290   -0.02040    0.017800      21.1   8.95e-01    9.43e-01
## Myristoleic acid; 65          0.001980   -0.03630    0.040300      19.9   9.19e-01    9.43e-01
## 1-Monopalmitin; 37            0.000772   -0.01500    0.016500      29.2   9.23e-01    9.43e-01
## 11-Eicosenoic acid; 35       -0.001210   -0.02850    0.026100      21.9   9.31e-01    9.43e-01
## Citric acid, 4TMS; 6          0.000411   -0.01690    0.017800      20.8   9.63e-01    9.63e-01
```

1.3.2 Adjusted Model

```
## [1] 586  12

##      (Intercept)    logUAER   Age Gender Hba1c_baseline      egfr CALSBP
## [1,]           1  0.5849625 19.39      0            5.2  11.03376     92
## [2,]           1 13.0138461 85.23      1           15.0 167.62905    191
##        bmi Smoking Statin log_Blood_TGA Total_cholesterol
## [1,] 16.98       0      0     -2.643856               2.3
## [2,] 43.29       1      1      2.720278               9.2

##
##                                 Overall
##   n                                586
##   egfr (mean (sd))               82.04 (28.24)
##   Age (mean (sd))                55.37 (12.11)
##   Gender (mean (sd))              0.54 (0.50)
##   Hba1c_baseline (mean (sd))      8.03 (1.16)
##   logUAER (mean (sd))             4.73 (2.31)
##   CALSBP (mean (sd))            131.91 (17.48)
##   bmi (mean (sd))                25.20 (4.06)
##   Smoking (mean (sd))             0.21 (0.41)
##   Statin (mean (sd))              0.61 (0.49)
##   log_Blood_TGA (mean (sd))       0.01 (0.69)
##   Total_cholesterol (mean (sd))   4.69 (0.88)
```

1.3.2.1 Table

```
##
##
## Table: logUAER
##
## Name                              Effect       CI.L        CI.R   AveExpr    P.Value   adj.P.Val
## --------------------------  ----------  ---------  ----------  --------  ---------  ----------
## 3,4-Dihydroxybutanoic aci     0.043800    0.02110    0.066500      15.9   0.000166      0.0125
## 4-Deoxytetronic acid; 32      0.046000    0.01670    0.075300      21.6   0.002120      0.0794
## Campesterol; 49              -0.042400   -0.07280   -0.012000      21.8   0.006310      0.1580
## Ribonic acid; 72              0.047700    0.01020    0.085200      20.0   0.012800      0.2250
## Octanoic acid; 68            -0.019000   -0.03440   -0.003650      24.2   0.015400      0.2250
## Glyceric acid; 30            -0.027400   -0.05050   -0.004230      20.4   0.020500      0.2250
## Docosahexaenoic acid; 53     -0.034600   -0.06470   -0.004420      24.2   0.024700      0.2250
## 2-Hydroxybutyric acid, 2T    -0.033600   -0.06400   -0.003280      20.7   0.029900      0.2250
## Aminomalonic acid; 45        -0.027900   -0.05350   -0.002350      24.3   0.032400      0.2250
## 2-hydroxy Isovaleric acid    -0.057600   -0.11000   -0.004780      22.2   0.032600      0.2250
## 2,4-Dihydroxybutanoic aci     0.024900    0.00187    0.048000      16.6   0.034100      0.2250
## Tyrosine; 75                 -0.055600   -0.10800   -0.003640      23.3   0.036000      0.2250
## Glutamic acid, 3TMS; 8        0.023300    0.00107    0.045500      23.0   0.040000      0.2310
## Arachidonic acid, TMS; 24    -0.016900   -0.03470    0.000927      22.7   0.063100      0.3380
## 3-Indolepropionic acid; 4    -0.050100   -0.10400    0.003730      19.5   0.068100      0.3400
## Ribitol; 71                   0.023900   -0.00225    0.050100      23.3   0.073100      0.3430
## Tridecanoic acid; 74         -0.021100   -0.04460    0.002480      20.0   0.079500      0.3510
## Valine, 2TMS; 20             -0.013300   -0.02860    0.002100      25.1   0.090600      0.3770
## Ethanolamine; 56             -0.014800   -0.03310    0.003420      22.7   0.111000      0.4350
## Lactic acid; 29              -0.012100   -0.02720    0.003050      25.4   0.117000      0.4350
## L-5-Oxoproline; 63           -0.012300   -0.02790    0.003290      27.5   0.122000      0.4350
## Ribitol; 70                   0.020000   -0.00788    0.047900      20.2   0.159000      0.5260
## 1,3-Propanediol; 34           0.016100   -0.00645    0.038700      24.2   0.161000      0.5260
## Hydroxyproline; 64            0.025200   -0.01240    0.062800      20.6   0.188000      0.5660
## Methionine, 2TMS; 16         -0.017300   -0.04350    0.008860      20.3   0.194000      0.5660
## 4-Hydroxybutanoic acid; 4     0.005770   -0.00299    0.014500      27.3   0.196000      0.5660
## 1-Dodecanol; 36              -0.012500   -0.03210    0.007190      20.2   0.213000      0.5770
## Proline, 2TMS; 21             0.013700   -0.00851    0.036000      20.8   0.226000      0.5770
## Heptadecanoic acid; 61       -0.009140   -0.02420    0.005920      23.4   0.234000      0.5770
## Pyroglutamic acid; 69        -0.020900   -0.05540    0.013600      22.9   0.235000      0.5770
## alpha-Tocopherol; 26         -0.017100   -0.04610    0.012000      18.8   0.250000      0.5770
## Myo inositol 6TMS; 1          0.011900   -0.00868    0.032400      19.1   0.257000      0.5770
## Creatinine; 50                0.018100   -0.01390    0.050100      21.6   0.267000      0.5770
## Glyceryl-glycoside; 59        0.016100   -0.01350    0.045700      20.8   0.286000      0.5770
## Arachidic acid; 46           -0.007270   -0.02070    0.006120      21.8   0.287000      0.5770
## Glycerol; 58                  0.028900   -0.02440    0.082200      24.9   0.287000      0.5770
## Heptadecanoic acid; 60       -0.011300   -0.03250    0.009870      21.8   0.295000      0.5770
## 4-Hydroxyphenyllactic aci    -0.013900   -0.04010    0.012200      20.8   0.295000      0.5770
## Cholesterol, TMS; 23         -0.006760   -0.01960    0.006050      21.6   0.300000      0.5770
## Succinic acid, 2TMS; 7       -0.006760   -0.02090    0.007410      22.7   0.349000      0.6550
## Threonine, 3TMS; 12          -0.009900   -0.03200    0.012200      28.6   0.380000      0.6650
## Linoleic acid, TMS; 4        -0.006490   -0.02110    0.008090      25.7   0.382000      0.6650
## Glycine, 3TMS; 17            -0.006380   -0.02080    0.008070      21.4   0.386000      0.6650
## 1-Monopalmitin; 37            0.008300   -0.01080    0.027400      29.2   0.394000      0.6650
## Citric acid, 4TMS; 6         -0.008560   -0.02850    0.011400      20.8   0.399000      0.6650
## 4-Deoxytetronic acid; 33      0.016600   -0.02630    0.059400      22.2   0.448000      0.7160
```

```
## alpha-ketoglutaric acid,      0.015400   -0.02440    0.055200       20.3   0.449000        0.7160
## 4-Hydroxybenzeneacetic ac     0.017700   -0.03270    0.068100       19.5   0.491000        0.7680
## Decanoic acid; 52             0.007340   -0.01780    0.032400       22.4   0.566000        0.8630
## 3-Indoleacetic acid; 40       0.009500   -0.02380    0.042800       20.5   0.575000        0.8630
## Palmitic acid, TMS; 5        -0.002710   -0.01350    0.008100       21.4   0.622000        0.9080
## Arabinopyranose; 51           0.007880   -0.02620    0.041900       20.2   0.650000        0.9080
## 2-Palmitoylglycerol; 39       0.003750   -0.01360    0.021100       25.9   0.671000        0.9080
## Isoleucine, 2TMS; 18          0.004830   -0.01950    0.029100       26.0   0.696000        0.9080
## Malic acid, 3TMS; 11          0.004200   -0.01710    0.025500       20.0   0.699000        0.9080
## Alanine, 2TMS; 25            -0.003040   -0.01880    0.012800       22.8   0.706000        0.9080
## Nonadecanoic acid; 66         0.004190   -0.01760    0.026000       20.0   0.706000        0.9080
## Dodecanoic acid; 54          -0.005030   -0.03160    0.021600       22.7   0.710000        0.9080
## Benzeneacetic acid; 47       -0.008090   -0.05150    0.035300       20.4   0.714000        0.9080
## Fumaric acid, 2TMS; 9        -0.002930   -0.01970    0.013800       24.1   0.731000        0.9140
## Stearic acid, TMS; 2         -0.001380   -0.00992    0.007160       17.4   0.751000        0.9210
## Hydroxylamine; 62            -0.003130   -0.02510    0.018800       27.7   0.779000        0.9210
## Tartronic acid; 73            0.005000   -0.03170    0.041700       21.3   0.789000        0.9210
## Serine, 3TMS; 14              0.002030   -0.01450    0.018500       26.0   0.809000        0.9210
## Oleic acid, TMS; 3            0.002190   -0.01560    0.020000       17.1   0.809000        0.9210
## Myristoleic acid; 65         -0.004910   -0.04990    0.040000       19.9   0.830000        0.9210
## Nonanoic acid; 67             0.001890   -0.01570    0.019500       24.4   0.833000        0.9210
## Pyruvic acid; 31             -0.004170   -0.04460    0.036300       19.6   0.840000        0.9210
## 3-Hydroxybutyric acid, 2T    -0.003570   -0.03990    0.032800       21.7   0.847000        0.9210
## 11-Eicosenoic acid; 35        0.002600   -0.03040    0.035600       21.9   0.877000        0.9400
## Leucine, 2TMS; 19             0.001260   -0.02090    0.023400       21.1   0.911000        0.9620
## Phenylalanine, 2TMS; 13      -0.000668   -0.01480    0.013500       24.3   0.926000        0.9650
## Bisphenol A; 48               0.000787   -0.02270    0.024300       21.2   0.948000        0.9650
## Eicosapentaenoic acid; 55     0.001090   -0.03440    0.036600       23.0   0.952000        0.9650
## Glycerol; 57                  0.000456   -0.01980    0.020700       28.7   0.965000        0.9650
```

2 Step 2: Survival Analysis of Combined Renal Endpoint in Relation to Prioritized Metabolites from Step 1

2.1 Step 2A: Crude Model

2.1.1 Table

Table 1: Crude survival model for combined renal endpoint.

Name	exp(coef)	lower .95	upper .95	Pr(>|z|)	p.adj
Ribonic acid; 72	2.400	1.940	2.980	0.00e+00	0.00e+00
3,4-Dihydroxybutanoic acid; 27	3.380	2.500	4.570	0.00e+00	0.00e+00
Myo inositol 6TMS; 1	3.280	2.390	4.510	0.00e+00	0.00e+00
Ribitol; 71	2.660	2.000	3.520	0.00e+00	0.00e+00
2,4-Dihydroxybutanoic acid; 28	2.560	1.910	3.430	0.00e+00	0.00e+00
4-Hydroxybenzeneacetic acid; 42	1.520	1.300	1.780	2.00e-07	4.10e-06
Isoleucine, 2TMS; 18	0.597	0.481	0.741	2.90e-06	6.95e-05
Valine, 2TMS; 20	0.384	0.257	0.573	2.90e-06	6.95e-05
Leucine, 2TMS; 19	0.564	0.439	0.725	8.00e-06	1.77e-04
Creatinine; 50	1.710	1.350	2.160	8.80e-06	1.84e-04
Methionine, 2TMS; 16	0.630	0.498	0.795	1.04e-04	2.09e-03
Glyceryl-glycoside; 59	1.800	1.330	2.450	1.66e-04	3.15e-03
2-Hydroxybutyric acid, 2TMS; 22	0.706	0.571	0.874	1.33e-03	2.40e-02
Fumaric acid, 2TMS; 9	2.080	1.310	3.310	1.92e-03	3.26e-02
Hydroxyproline; 64	1.360	1.110	1.660	3.50e-03	5.60e-02
4-Hydroxyphenyllactic acid; 44	1.570	1.150	2.160	5.13e-03	7.69e-02
4-Deoxytetronic acid; 32	1.420	1.110	1.820	5.70e-03	7.98e-02
Malic acid, 3TMS; 11	1.650	1.150	2.370	6.39e-03	8.31e-02
4-Deoxytetronic acid; 33	1.230	1.030	1.470	2.55e-02	3.06e-01
3-Indoleacetic acid; 40	1.280	1.030	1.590	2.66e-02	3.06e-01
2-hydroxy Isovaleric acid; 38	0.876	0.769	0.997	4.56e-02	4.56e-01
Palmitic acid, TMS; 5	1.860	0.944	3.680	7.28e-02	6.55e-01
Citric acid, 4TMS; 6	1.400	0.964	2.030	7.78e-02	6.55e-01
Pyroglutamic acid; 69	1.230	0.963	1.580	9.58e-02	6.70e-01
Succinic acid, 2TMS; 7	1.590	0.921	2.750	9.58e-02	6.70e-01
Serine, 3TMS; 14	0.699	0.456	1.070	1.01e-01	6.70e-01
Eicosapentaenoic acid; 55	0.863	0.719	1.030	1.12e-01	6.70e-01
Glycine, 3TMS; 17	1.130	0.686	1.870	6.26e-01	1.00e+00
Stearic acid, TMS; 2	1.180	0.480	2.890	7.20e-01	1.00e+00
Glycerol; 57	1.040	0.700	1.550	8.44e-01	1.00e+00

2.2 Step 2B: Adjusted Model

2.2.1 Table

Table 2: Adjusted survival model for combined renal endpoint.

Name	exp(coef)	lower .95	upper .95	Pr(>|z|)	p.adj
Leucine, 2TMS; 19	0.591	0.463	0.753	2.06e-05	0.000618
Ribonic acid; 72	1.750	1.340	2.300	4.62e-05	0.001340
Isoleucine, 2TMS; 18	0.611	0.470	0.794	2.36e-04	0.006620
Valine, 2TMS; 20	0.432	0.271	0.689	4.26e-04	0.011500
Myo inositol 6TMS; 1	1.800	1.190	2.710	4.96e-03	0.129000
Methionine, 2TMS; 16	0.734	0.559	0.964	2.60e-02	0.650000
2-Hydroxybutyric acid, 2TMS; 22	0.761	0.596	0.971	2.82e-02	0.677000
3,4-Dihydroxybutanoic acid; 27	1.540	1.040	2.280	2.94e-02	0.677000
4-Deoxytetronic acid; 33	0.799	0.652	0.980	3.13e-02	0.688000
2,4-Dihydroxybutanoic acid; 28	1.360	0.943	1.980	9.89e-02	1.000000
Palmitic acid, TMS; 5	1.810	0.819	3.990	1.43e-01	1.000000
Malic acid, 3TMS; 11	1.340	0.896	2.000	1.55e-01	1.000000
Fumaric acid, 2TMS; 9	1.430	0.856	2.400	1.70e-01	1.000000
Creatinine; 50	1.170	0.933	1.460	1.77e-01	1.000000
Ribitol; 71	1.250	0.887	1.750	2.04e-01	1.000000
Hydroxyproline; 64	1.110	0.894	1.390	3.37e-01	1.000000
4-Hydroxyphenyllactic acid; 44	1.140	0.803	1.620	4.63e-01	1.000000
Glyceryl-glycoside; 59	1.110	0.806	1.520	5.26e-01	1.000000
4-Hydroxybenzeneacetic acid; 42	1.060	0.889	1.250	5.37e-01	1.000000
Glycine, 3TMS; 17	0.843	0.483	1.470	5.46e-01	1.000000
Succinic acid, 2TMS; 7	1.180	0.653	2.150	5.77e-01	1.000000
Stearic acid, TMS; 2	1.260	0.497	3.210	6.25e-01	1.000000
4-Deoxytetronic acid; 32	1.070	0.793	1.430	6.69e-01	1.000000
Citric acid, 4TMS; 6	0.913	0.601	1.390	6.70e-01	1.000000
Pyroglutamic acid; 69	0.957	0.742	1.240	7.37e-01	1.000000
Glycerol; 57	1.070	0.721	1.590	7.41e-01	1.000000
Serine, 3TMS; 14	0.924	0.572	1.490	7.46e-01	1.000000
Eicosapentaenoic acid; 55	0.968	0.773	1.210	7.80e-01	1.000000
2-hydroxy Isovaleric acid; 38	1.000	0.857	1.180	9.54e-01	1.000000
3-Indoleacetic acid; 40	1.000	0.780	1.290	9.70e-01	1.000000

2.2.2 Forest Plot

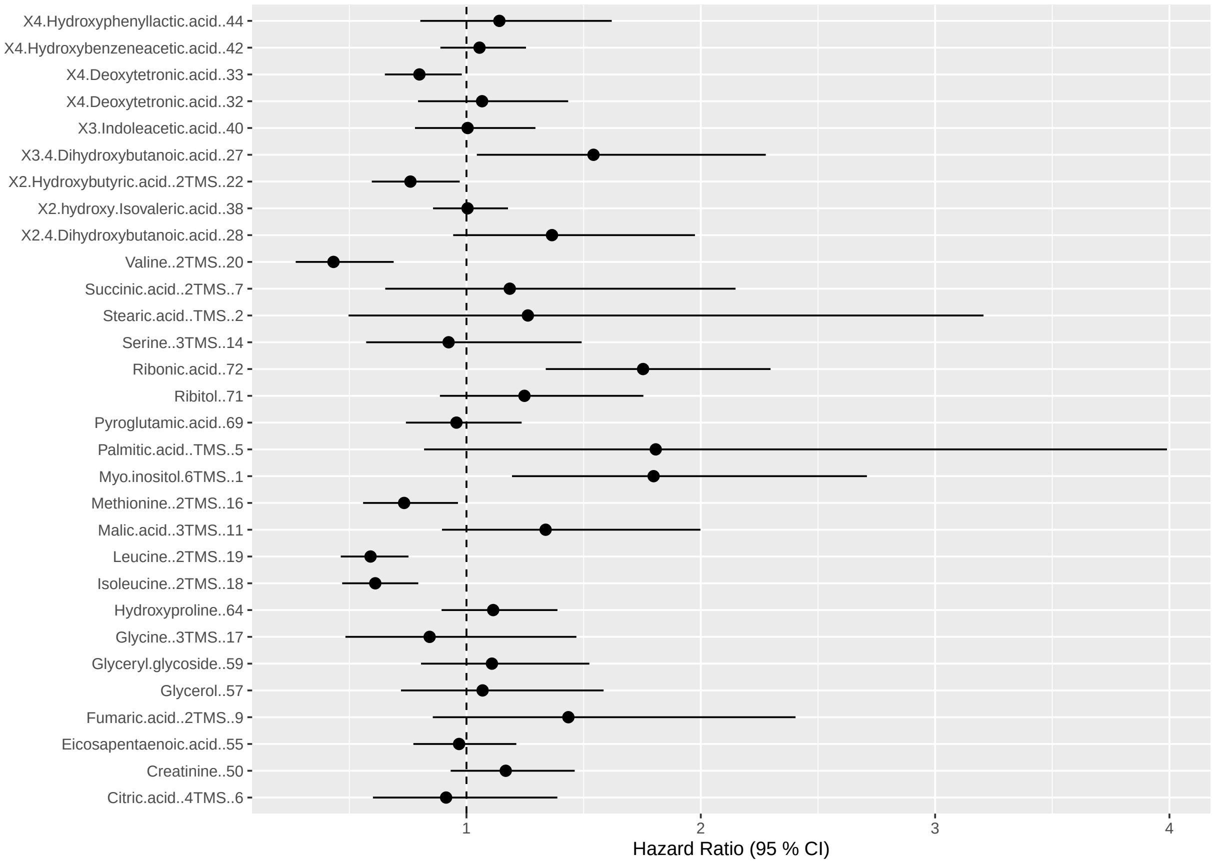

2.3 Combined Forest Plot from Crude and Adjusted Models

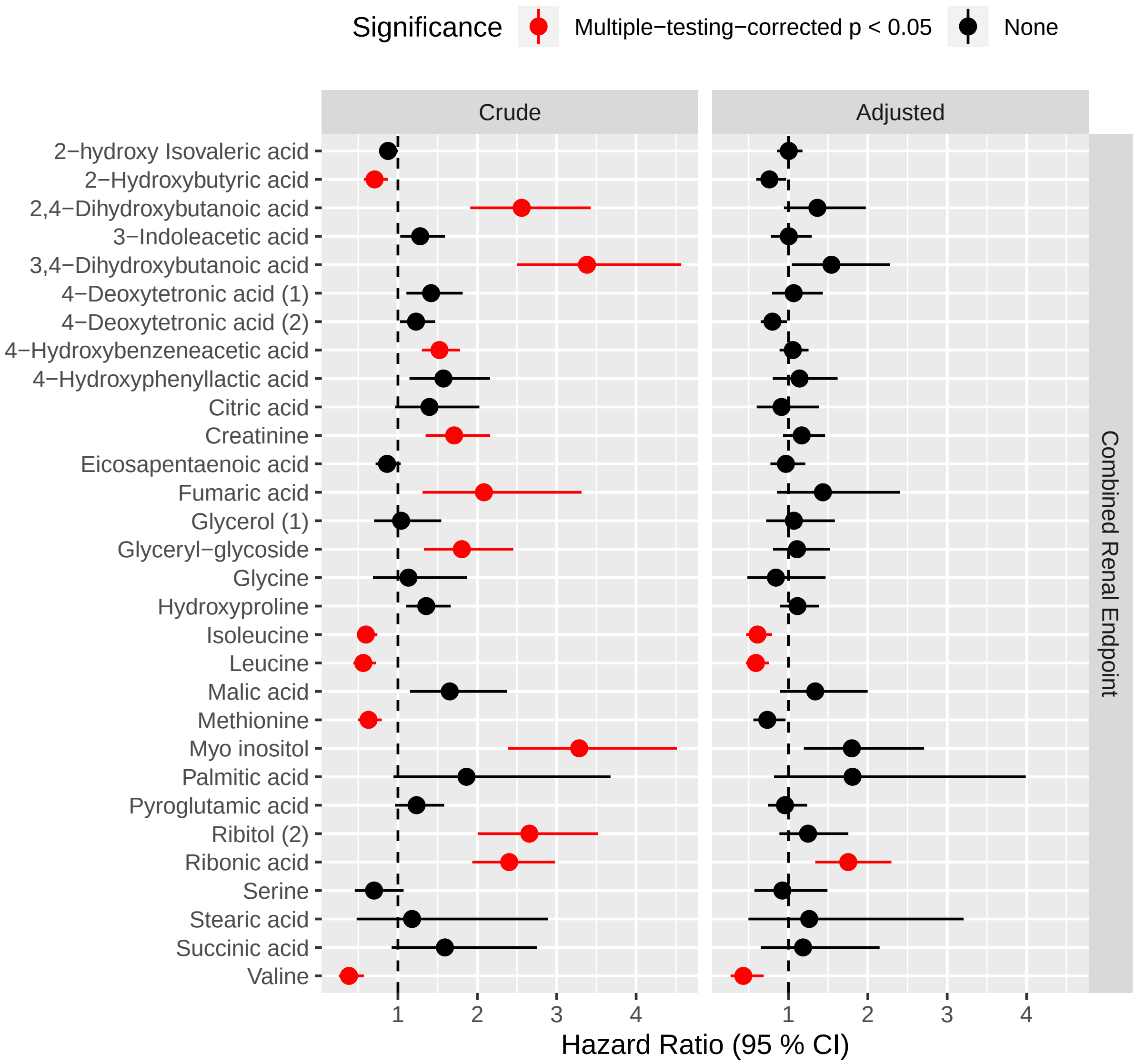

3 Step 3: Survival Analysis of Specific Renal Endpoints in Relation to Prioritized Metabolites from Step 3A

3.1 Step 3A: All-Cause Mortality

3.1.1 Crude Model

3.1.1.1 Table

Table 3: Crude survival model for all-cause mortality.

Name	exp(coef)	lower .95	upper .95	Pr(>|z|)	p.adj
Ribonic.acid..72	2.030	1.500	2.760	5.40e-06	7.58e-05
Ribitol..71	2.550	1.700	3.810	5.60e-06	7.58e-05
X3.4.Dihydroxybutanoic.acid..27	2.570	1.660	3.980	2.47e-05	2.97e-04
X2.4.Dihydroxybutanoic.acid..28	2.360	1.550	3.570	5.61e-05	6.18e-04
X4.Hydroxybenzeneacetic.acid..42	1.530	1.220	1.920	2.38e-04	2.38e-03
Myo.inositol.6TMS..1	2.310	1.460	3.640	3.30e-04	2.97e-03
X2.Hydroxybutyric.acid..2TMS..22	0.601	0.449	0.805	6.36e-04	5.09e-03
Isoleucine..2TMS..18	0.654	0.490	0.873	3.94e-03	2.76e-02
Creatinine..50	1.570	1.120	2.210	9.26e-03	5.56e-02
Valine..2TMS..20	0.458	0.252	0.830	1.00e-02	5.56e-02
Glyceryl.glycoside..59	1.690	1.080	2.640	2.04e-02	8.18e-02
Fumaric.acid..2TMS..9	2.080	1.060	4.070	3.33e-02	9.98e-02
Leucine..2TMS..19	0.699	0.494	0.988	4.23e-02	9.98e-02
Methionine..2TMS..16	0.996	0.669	1.480	9.85e-01	9.85e-01

3.1.1.2 Forest Plot

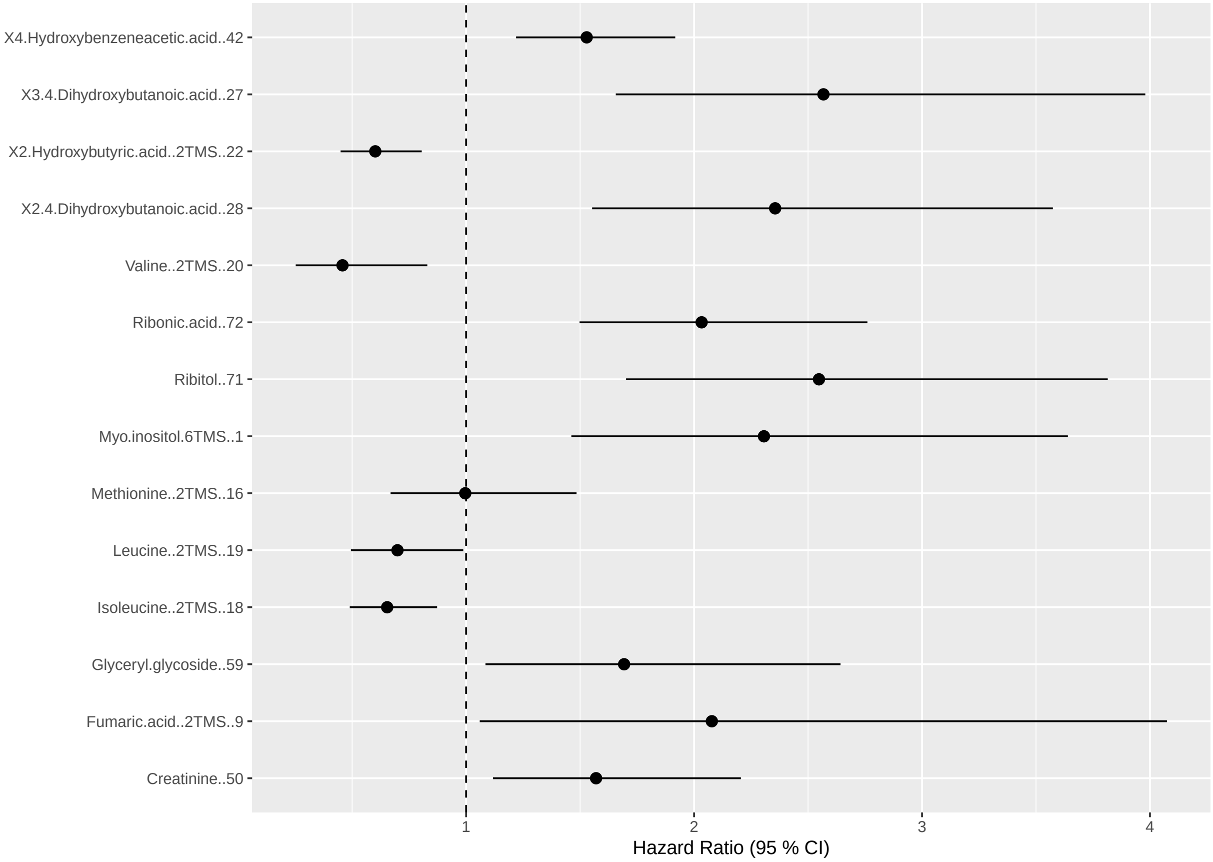

3.1.2 Adjusted Model

3.1.2.1 Table

Table 4: Adjusted survival model for all-cause mortality.

Name	exp(coef)	lower .95	upper .95	Pr(>|z|)	p.adj
X2.Hydroxybutyric.acid..2TMS..22	0.646	0.460	0.907	0.0117	0.163
Ribonic.acid..72	1.570	1.060	2.320	0.0233	0.303
Isoleucine..2TMS..18	0.694	0.477	1.010	0.0568	0.681
Ribitol..71	1.600	0.930	2.740	0.0901	0.991
Leucine..2TMS..19	0.750	0.505	1.110	0.1530	1.000
Myo.inositol.6TMS..1	1.560	0.846	2.880	0.1540	1.000
Fumaric.acid..2TMS..9	1.640	0.795	3.400	0.1800	1.000
Creatinine..50	1.320	0.866	2.000	0.1990	1.000
X2.4.Dihydroxybutanoic.acid..28	1.430	0.824	2.490	0.2030	1.000
Glyceryl.glycoside..59	1.360	0.828	2.240	0.2230	1.000
Valine..2TMS..20	0.668	0.341	1.310	0.2390	1.000
X3.4.Dihydroxybutanoic.acid..27	1.340	0.769	2.320	0.3040	1.000
X4.Hydroxybenzeneacetic.acid..42	1.100	0.857	1.410	0.4590	1.000
Methionine..2TMS..16	0.952	0.598	1.520	0.8370	1.000

3.1.2.2 Forest Plot

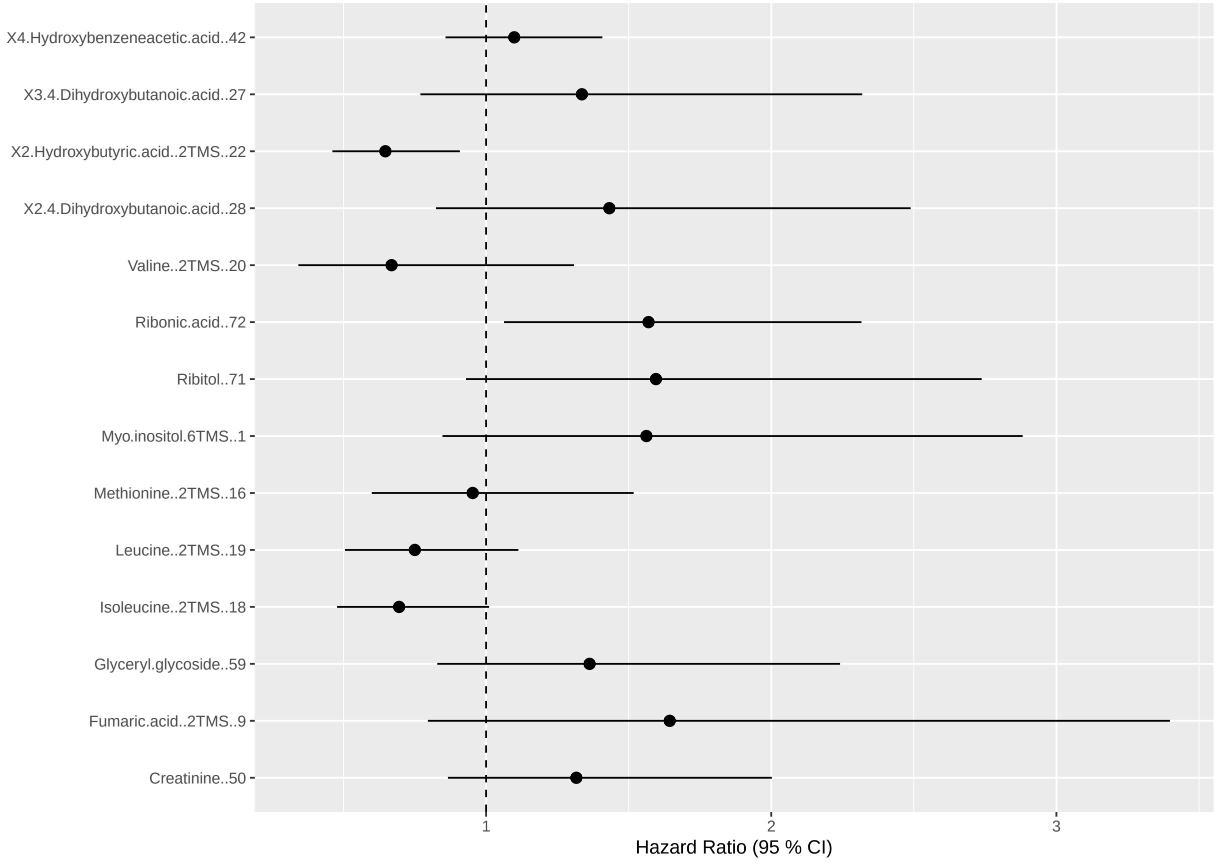

3.1.3 Combined Forest Plot from Crude and Adjusted Models

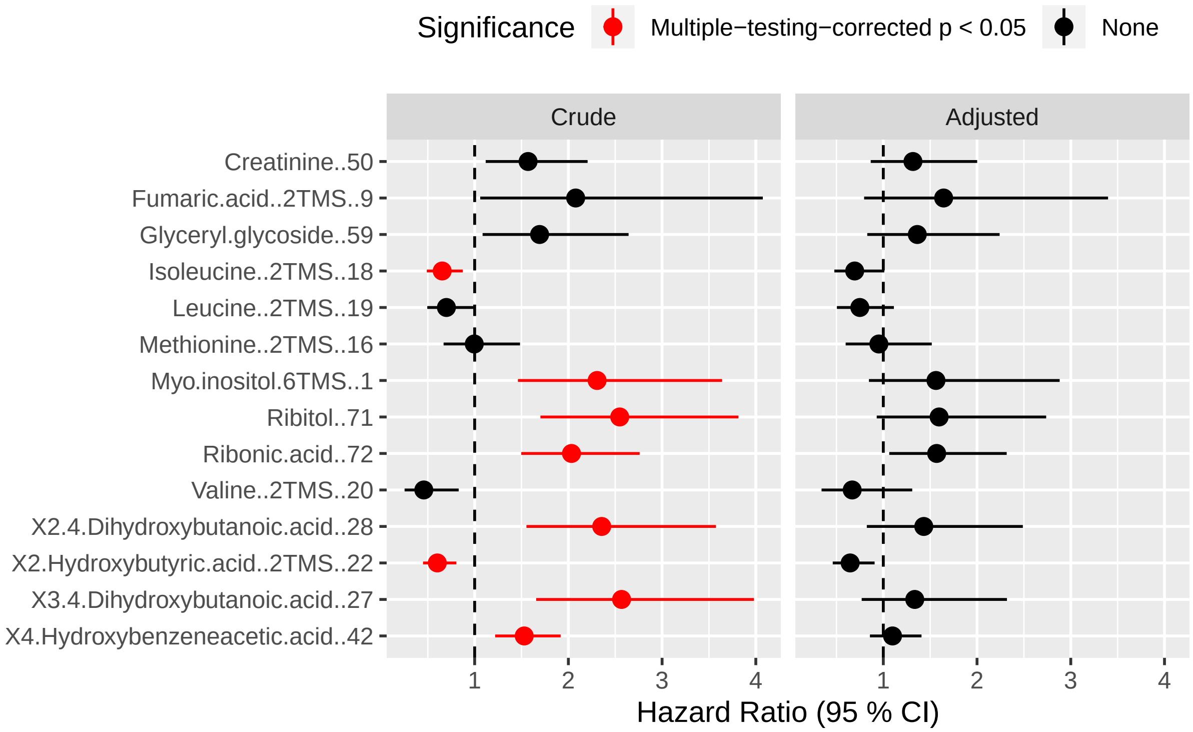

3.2 Step 3B: eGFR Decline > 30 %

3.2.1 Crude Model

3.2.1.1 Table

Table 5: Crude survival model for eGFR decline > 30 %

Name	exp(coef)	lower .95	upper .95	Pr(>|z|)	p.adj
Ribonic.acid..72	2.510	1.950	3.230	0.000000	0.00e+00
Myo.inositol.6TMS..1	4.060	2.760	5.970	0.000000	0.00e+00
X3.4.Dihydroxybutanoic.acid..27	3.700	2.580	5.300	0.000000	0.00e+00
Ribitol..71	2.510	1.810	3.480	0.000000	3.00e-07
X2.4.Dihydroxybutanoic.acid..28	2.580	1.840	3.620	0.000000	4.00e-07
X4.Hydroxybenzeneacetic.acid..42	1.410	1.170	1.690	0.000218	1.96e-03
Methionine..2TMS..16	0.611	0.466	0.800	0.000345	2.76e-03
Glyceryl.glycoside..59	1.850	1.300	2.640	0.000705	4.94e-03
Creatinine..50	1.620	1.230	2.150	0.000734	4.94e-03
Isoleucine..2TMS..18	0.664	0.512	0.861	0.002010	1.01e-02
Valine..2TMS..20	0.515	0.309	0.857	0.010700	4.28e-02
Leucine..2TMS..19	0.765	0.557	1.050	0.096100	2.88e-01
Fumaric.acid..2TMS..9	1.560	0.910	2.670	0.106000	2.88e-01
X2.Hydroxybutyric.acid..2TMS..22	0.874	0.667	1.150	0.332000	3.32e-01

3.2.2 Forest Plot

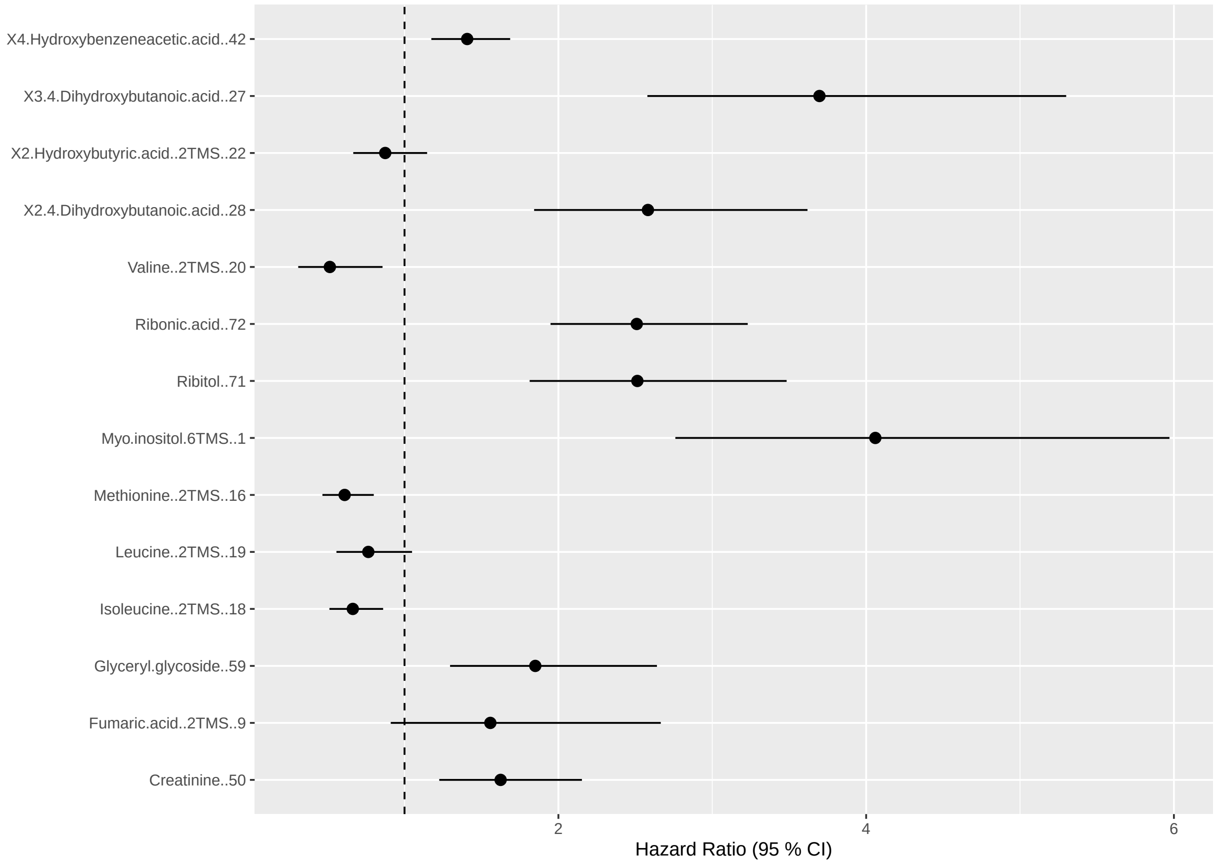

3.2.3 Adjusted Model

3.2.3.1 Table

Table 6: Adjusted survival model for eGFR decline > 30 %.

Name	exp(coef)	lower .95	upper .95	Pr(>|z|)	p.adj
Ribonic.acid..72	2.220	1.640	2.990	2.00e-07	2.60e-06
Myo.inositol.6TMS..1	2.660	1.630	4.340	8.69e-05	1.13e-03
X3.4.Dihydroxybutanoic.acid..27	1.890	1.180	3.030	8.34e-03	1.00e-01
X2.4.Dihydroxybutanoic.acid..28	1.680	1.080	2.610	2.05e-02	2.25e-01
Isoleucine..2TMS..18	0.739	0.556	0.983	3.75e-02	3.75e-01
Valine..2TMS..20	0.552	0.313	0.975	4.05e-02	3.75e-01
X2.Hydroxybutyric.acid..2TMS..22	0.764	0.548	1.070	1.13e-01	9.01e-01
Methionine..2TMS..16	0.769	0.544	1.090	1.35e-01	9.48e-01
Ribitol..71	1.320	0.886	1.950	1.74e-01	1.00e+00
Creatinine..50	1.180	0.920	1.520	1.90e-01	1.00e+00
X4.Hydroxybenzeneacetic.acid..42	1.120	0.921	1.370	2.49e-01	1.00e+00
Leucine..2TMS..19	0.880	0.655	1.180	3.98e-01	1.00e+00
Glyceryl.glycoside..59	1.160	0.778	1.740	4.63e-01	1.00e+00
Fumaric.acid..2TMS..9	0.804	0.441	1.470	4.76e-01	1.00e+00

3.2.3.2 Forest Plot

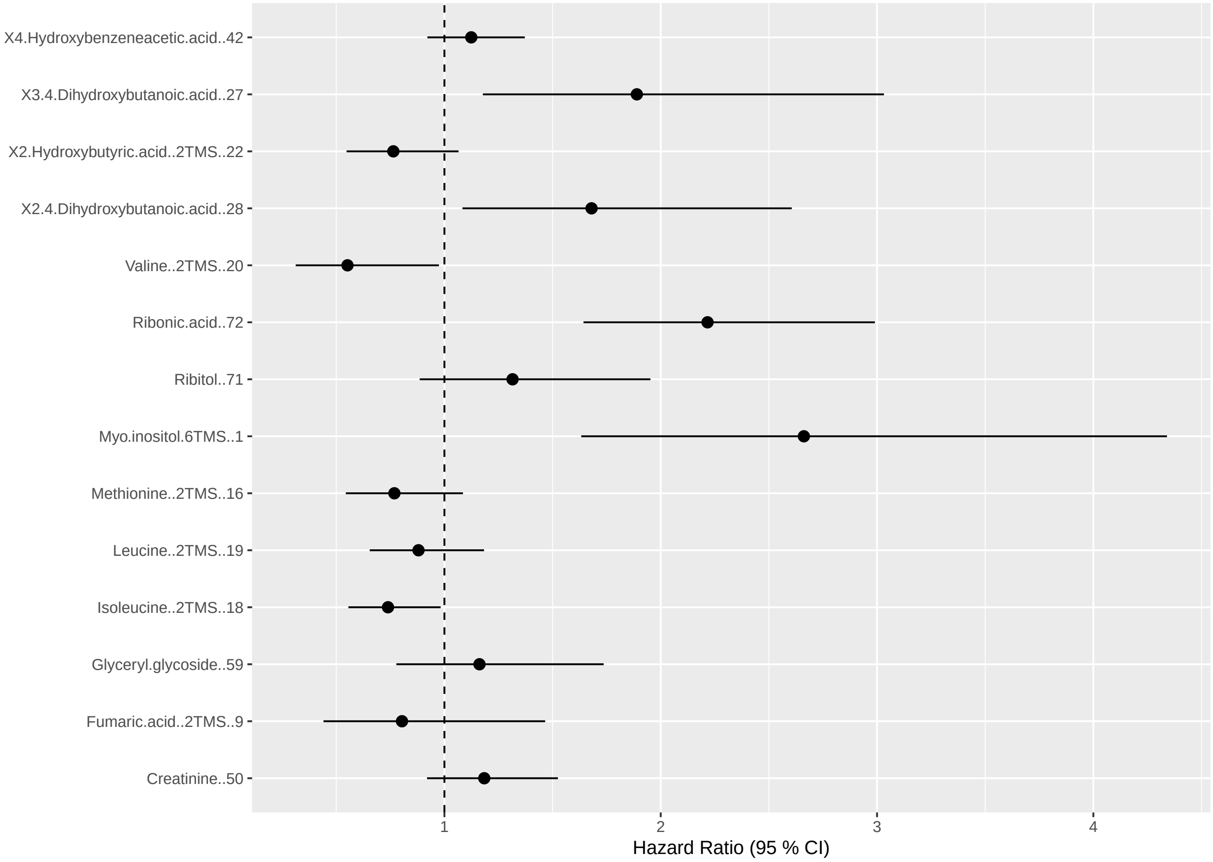

3.2.4 Combined Forest Plot from Crude and Adjusted Models

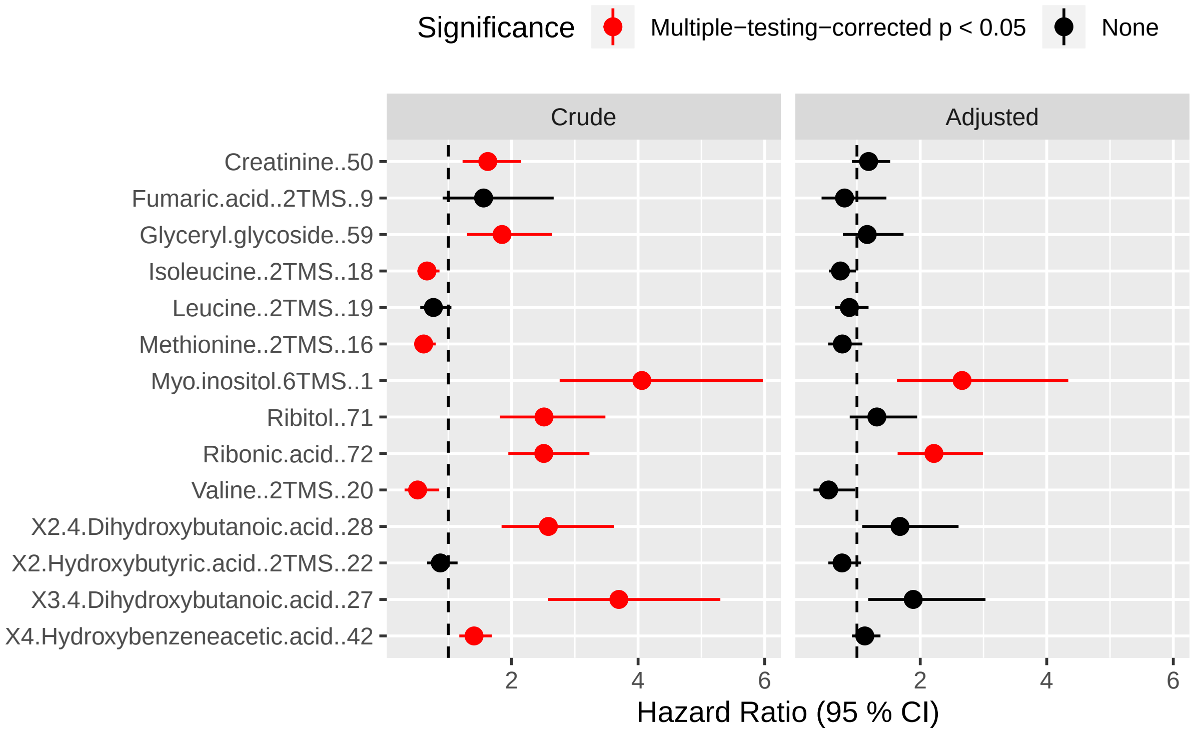

3.3 Step 3C: End-Stage Renal Disease

3.3.1 Crude Model

3.3.1.1 Table

Table 7: Crude survival model for end-stage renal disease.

Name	exp(coef)	lower .95	upper .95	Pr(>|z|)	p.adj
Myo.inositol.6TMS..1	8.310	3.930	17.500	0.00e+00	4.00e-07
X3.4.Dihydroxybutanoic.acid..27	7.360	3.530	15.400	1.00e-07	1.40e-06
Ribonic.acid..72	3.870	2.310	6.500	3.00e-07	3.70e-06
X2.4.Dihydroxybutanoic.acid..28	6.300	3.100	12.800	3.00e-07	3.80e-06
Creatinine..50	3.760	2.140	6.600	4.10e-06	4.08e-05
X4.Hydroxybenzeneacetic.acid..42	2.490	1.680	3.680	4.90e-06	4.42e-05
Ribitol..71	4.000	2.070	7.710	3.66e-05	2.92e-04
Glyceryl.glycoside..59	4.000	1.880	8.510	3.28e-04	2.29e-03
X2.Hydroxybutyric.acid..2TMS..22	0.492	0.326	0.740	6.69e-04	4.01e-03
Methionine..2TMS..16	0.500	0.307	0.813	5.23e-03	2.61e-02
Isoleucine..2TMS..18	0.576	0.383	0.868	8.34e-03	3.34e-02
Fumaric.acid..2TMS..9	4.310	1.450	12.800	8.58e-03	3.34e-02
Valine..2TMS..20	0.340	0.137	0.840	1.94e-02	3.88e-02
Leucine..2TMS..19	0.557	0.333	0.931	2.56e-02	3.88e-02

3.3.1.2 Forest Plot

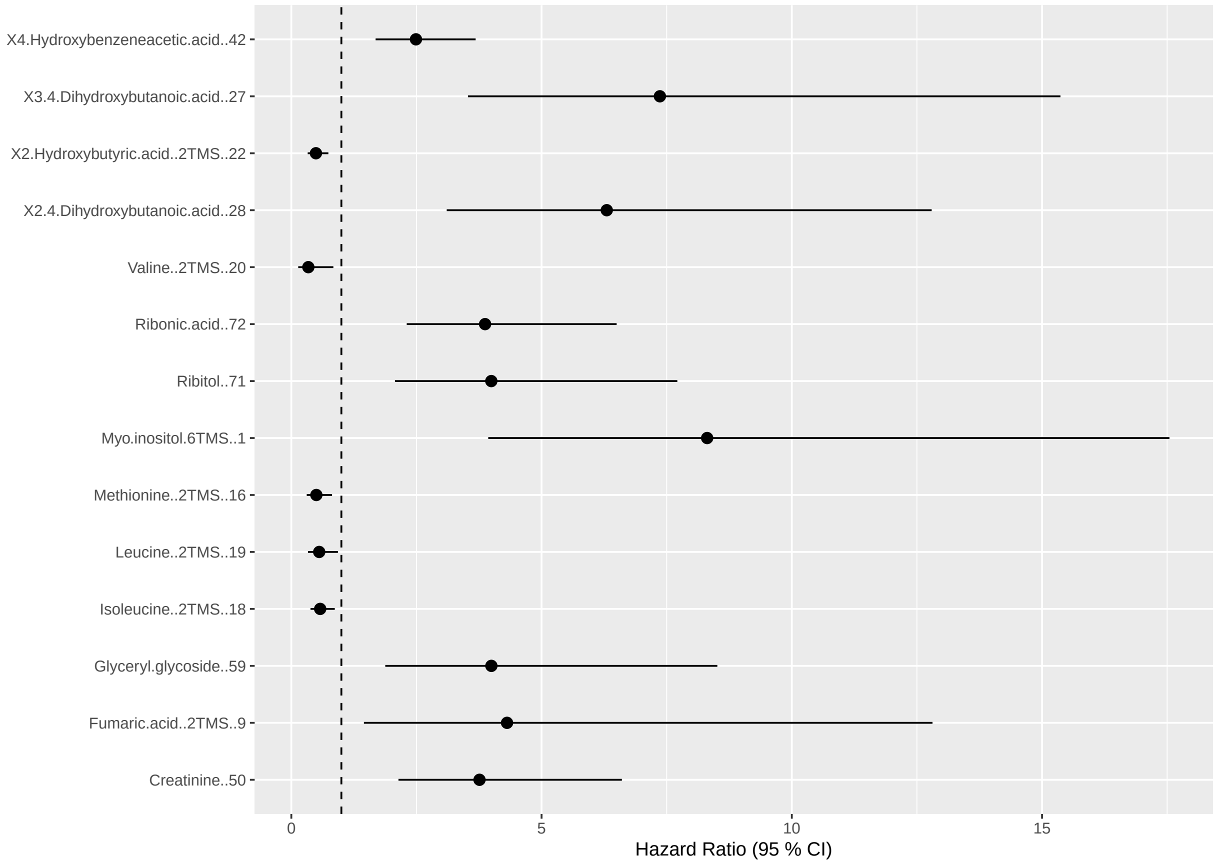

3.3.2 Adjusted Model

3.3.2.1 Table

Table 8: Adjusted survival model for end-stage renal disease.

Name	exp(coef)	lower .95	upper .95	Pr(>|z|)	p.adj
Ribitol..71	0.459	0.222	0.946	0.0349	0.489
Methionine..2TMS..16	0.606	0.320	1.150	0.1250	1.000
Valine..2TMS..20	0.423	0.126	1.420	0.1640	1.000
X4.Hydroxybenzeneacetic.acid..42	0.664	0.373	1.180	0.1640	1.000
X2.Hydroxybutyric.acid..2TMS..22	0.699	0.360	1.360	0.2900	1.000
Isoleucine..2TMS..18	0.778	0.467	1.290	0.3330	1.000
Fumaric.acid..2TMS..9	1.770	0.443	7.090	0.4180	1.000
Leucine..2TMS..19	0.840	0.546	1.290	0.4280	1.000
Glyceryl.glycoside..59	0.705	0.284	1.750	0.4500	1.000
Creatinine..50	0.890	0.561	1.410	0.6210	1.000
X3.4.Dihydroxybutanoic.acid..27	1.260	0.421	3.780	0.6770	1.000
Ribonic.acid..72	0.878	0.434	1.770	0.7170	1.000
Myo.inositol.6TMS..1	0.978	0.310	3.080	0.9700	1.000
X2.4.Dihydroxybutanoic.acid..28	0.991	0.331	2.960	0.9870	1.000

3.3.2.2 Forest Plot

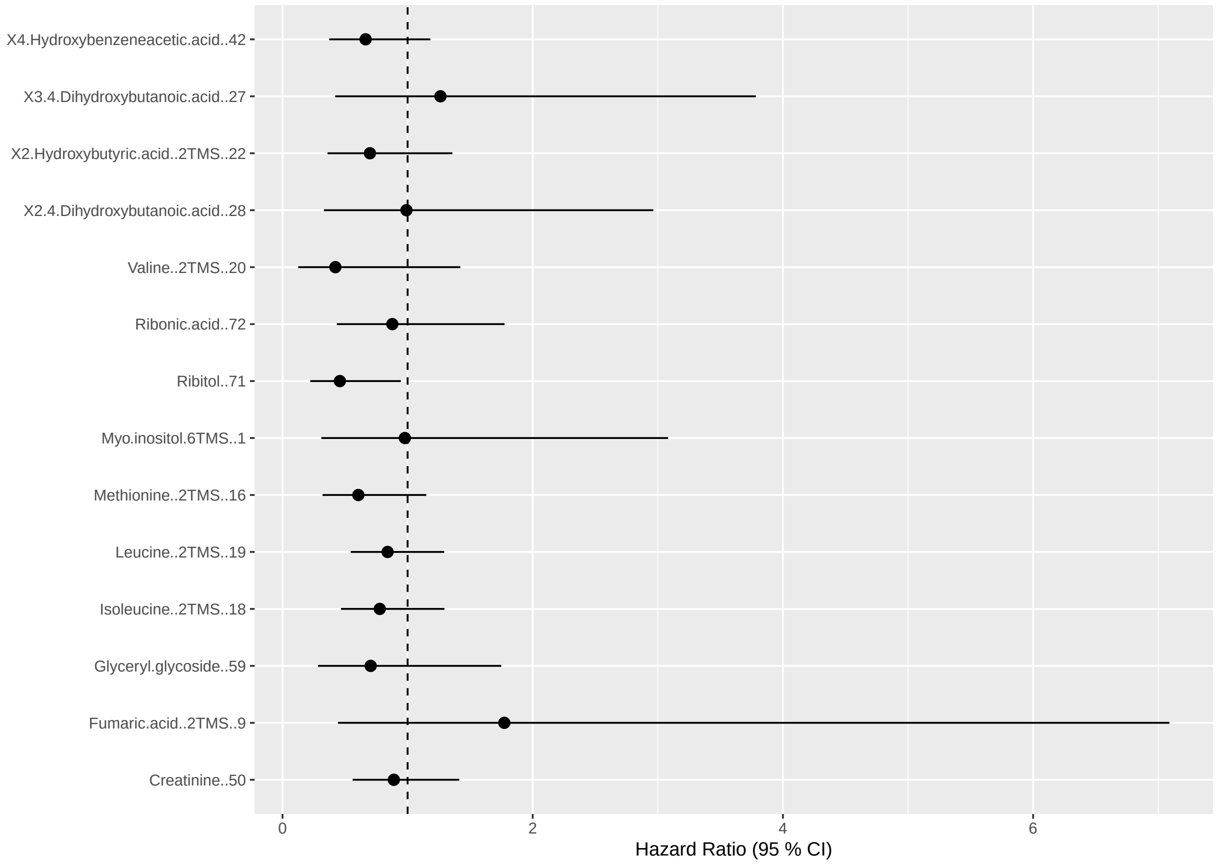

3.3.3 Combined Forest Plot from Crude and Adjusted Models

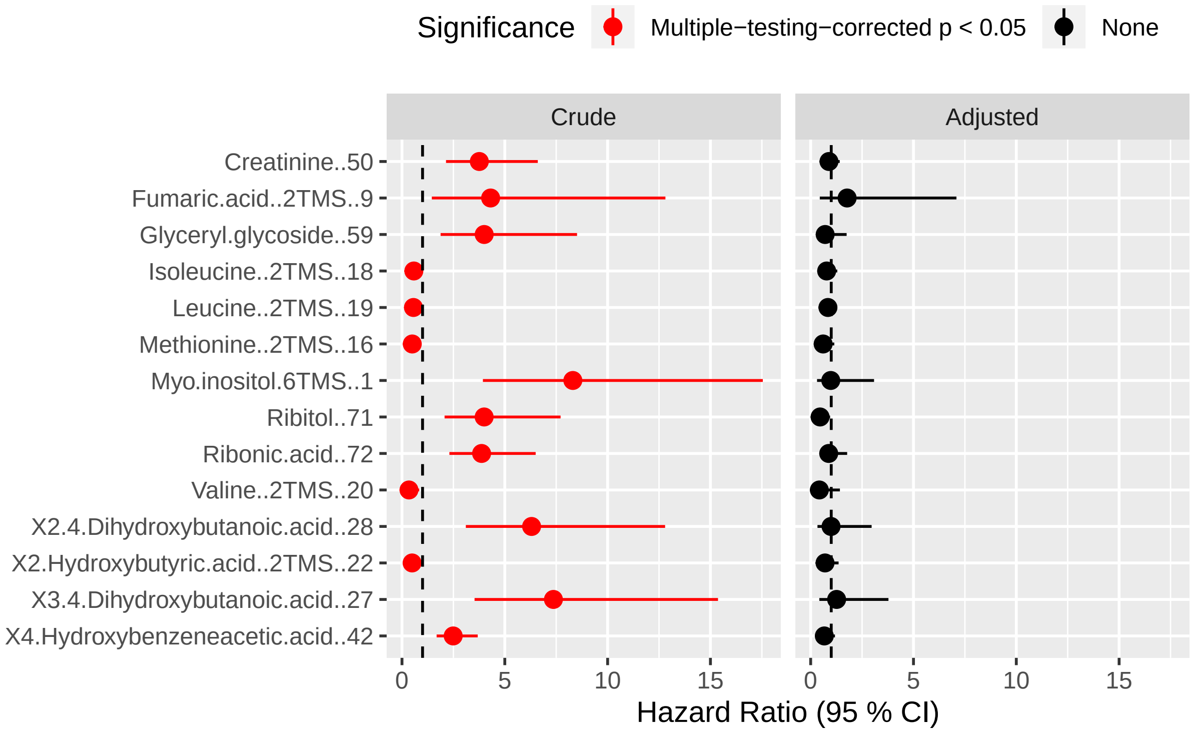

3.4 Compilation Forest Plot from Steps 3A-C

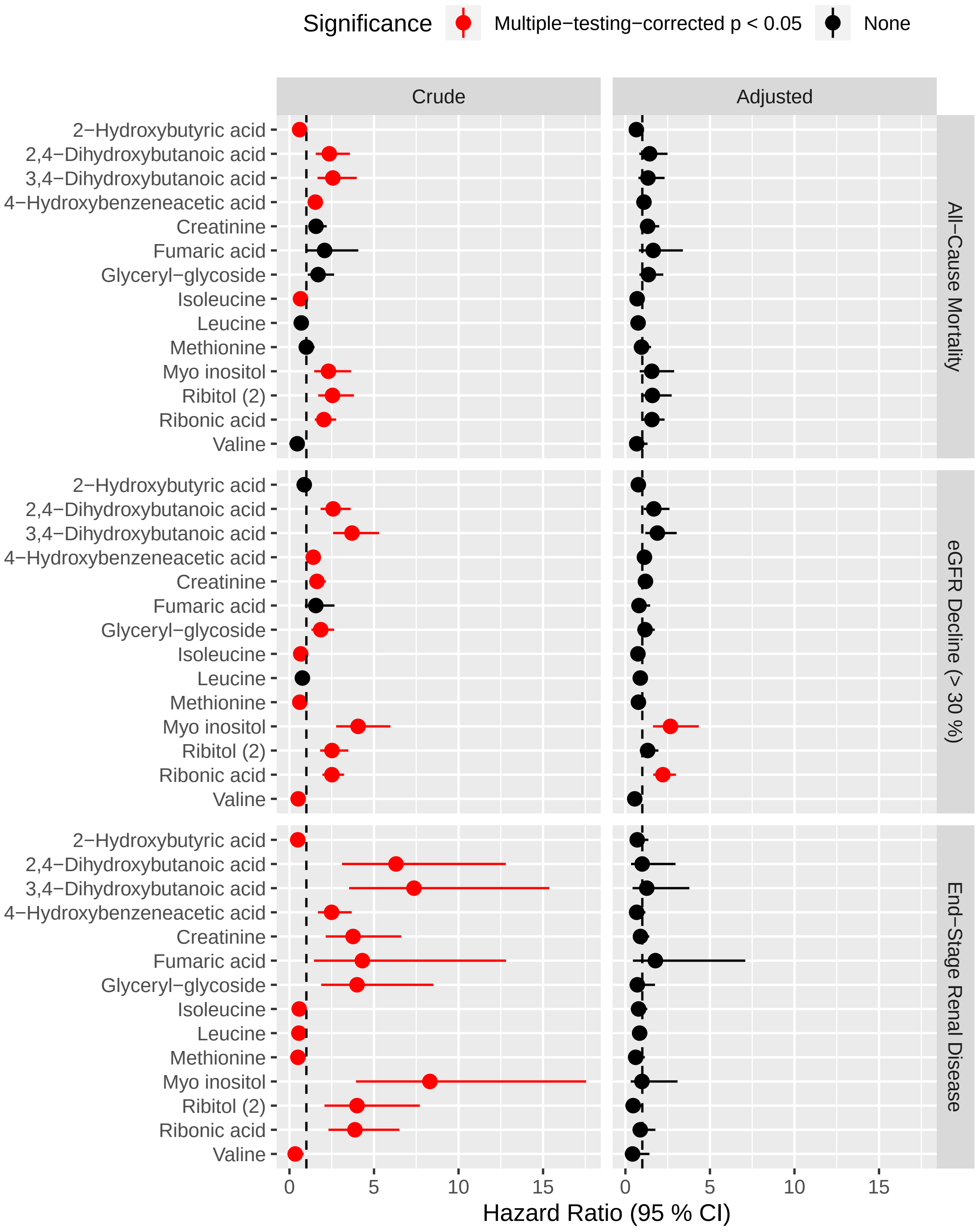

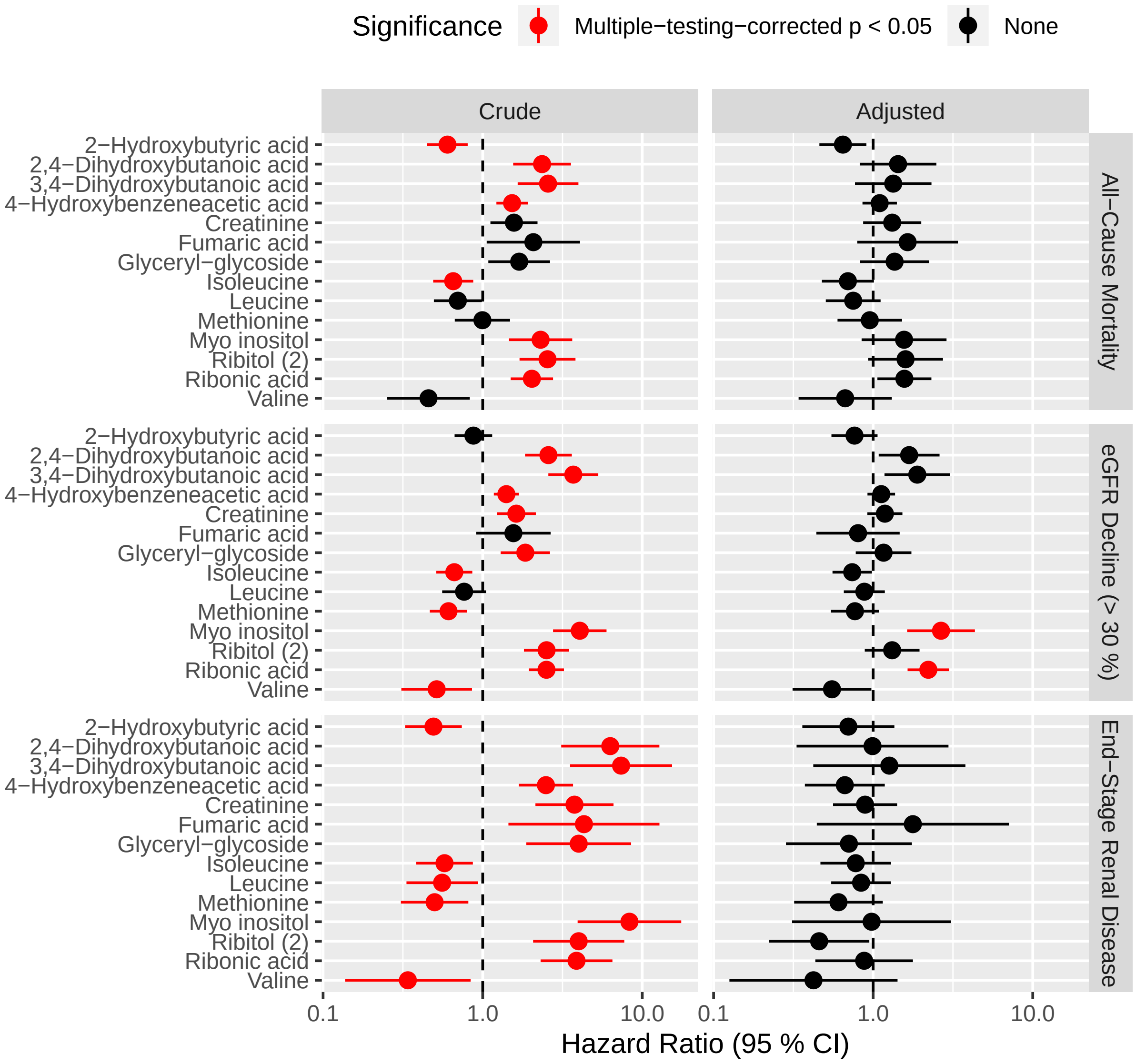
Significance
Multiple−testing−corrected p < 0.05
None
Crude
Adjusted
All−Cause Mortality
eGFR Decline (> 30 %)
End−Stage Renal Disease
2−Hydroxybutyric acid
2,4−Dihydroxybutanoic acid
3,4−Dihydroxybutanoic acid
4−Hydroxybenzeneacetic acid
Creatinine
Fumaric acid
Glyceryl−glycoside
Isoleucine
Leucine
Methionine
Myo inositol
Ribitol (2)
Ribonic acid
Valine
0.1
1.0
10.0
Hazard Ratio (95 % CI)

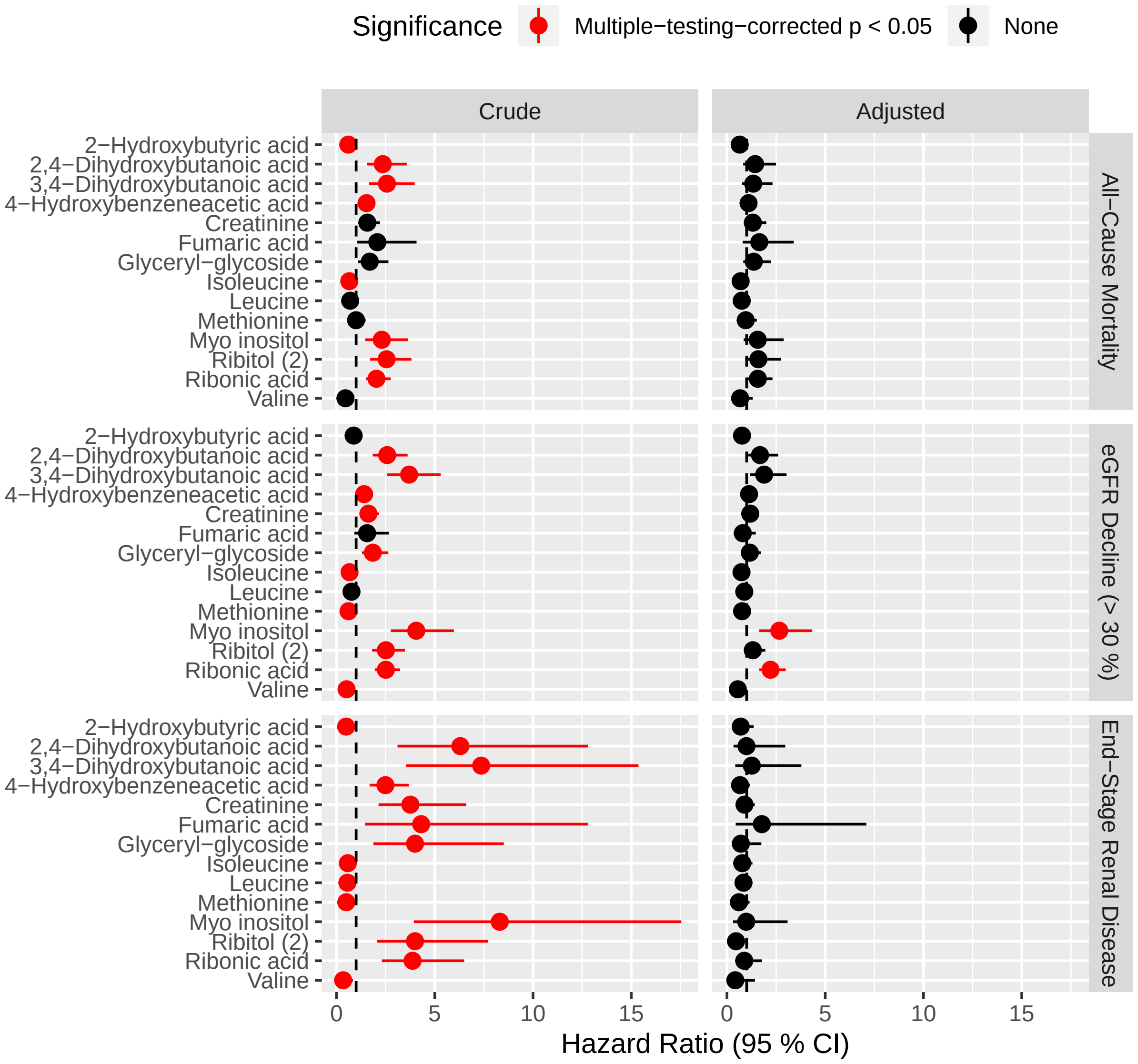

Significance
Multiple−testing−corrected p < 0.05
None
Crude
Adjusted
All−Cause Mortality
eGFR Decline (> 30 %)
End−Stage Renal Disease
2−Hydroxybutyric acid
2,4−Dihydroxybutanoic acid
3,4−Dihydroxybutanoic acid
4−Hydroxybenzeneacetic acid
Creatinine
Fumaric acid
Glyceryl−glycoside
Isoleucine
Leucine
Methionine
Myo inositol
Ribitol (2)
Ribonic acid
Valine
0
5
10
15
Hazard Ratio (95 % CI)

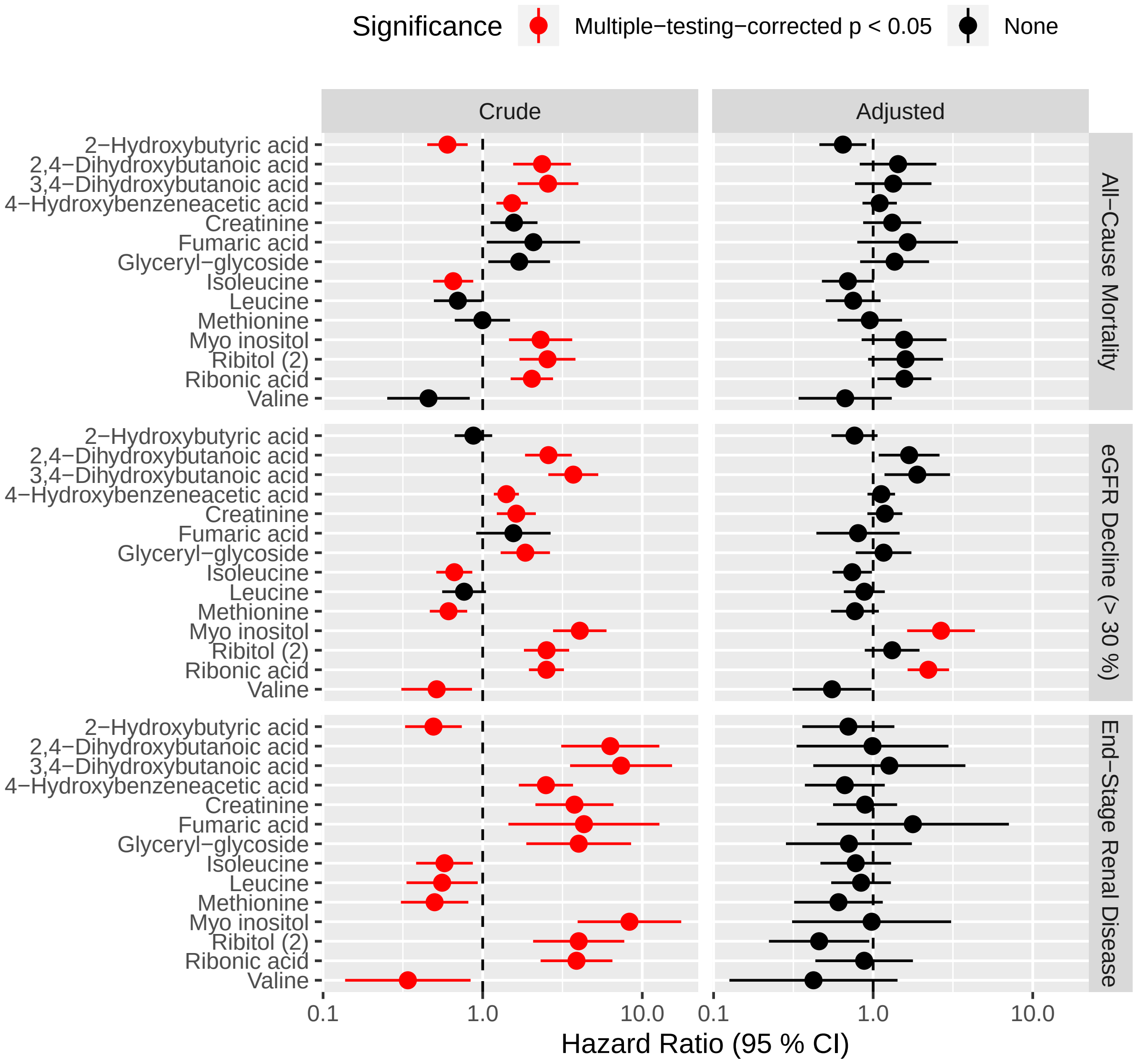
Significance
Multiple−testing−corrected p < 0.05
None
Crude
Adjusted
2−Hydroxybutyric acid
2,4−Dihydroxybutanoic acid
3,4−Dihydroxybutanoic acid
4−Hydroxybenzeneacetic acid
Creatinine
Fumaric acid
Glyceryl−glycoside
Isoleucine
Leucine
Methionine
Myo inositol
Ribitol (2)
Ribonic acid
Valine
All−Cause Mortality
eGFR Decline (> 30 %)
End−Stage Renal Disease
0.1
1.0
10.0
Hazard Ratio (95 % CI)

4 Step 4: Detailed Assessment of the Top-Metabolites in Relation to Outcomes

4.1 Step 4.1: First Top-Metabolite in Relation to eGFR Decline

4.1.1 Step 4.1A: Analysis of Full Cohort

4.1.1.1 Survival Model with Details

```
## Call:
## survival::coxph(formula = survival::Surv(time = t_gfrfald30_p,
##     event = censor_gfrfald30_p.reversed.numeric) ~ Ribonic_acid +
##     Age + BMI + BP_Systolic + Cholesterol + eGFR + HbA1c + Medication_Statins +
##     Sex + Smoking + TG_total_log + UAER_log, data = data.survival)
##
##   n= 586, number of events= 87
##    (51 observations deleted due to missingness)
##
##                          coef exp(coef)  se(coef)      z Pr(>|z|)
## Ribonic_acid        0.795932  2.216505  0.152741  5.211 1.88e-07 ***
## Age                -0.013717  0.986377  0.012771 -1.074 0.282806
## BMI                -0.004863  0.995149  0.029311 -0.166 0.868227
## BP_Systolic         0.025752  1.026086  0.006728  3.828 0.000129 ***
## Cholesterol         0.166837  1.181562  0.132443  1.260 0.207782
## eGFR               -0.008983  0.991057  0.004990 -1.800 0.071824 .
## HbA1c               0.421594  1.524389  0.084607  4.983 6.26e-07 ***
## Medication_Statins  0.631948  1.881271  0.312823  2.020 0.043368 *
## Sex                 0.160840  1.174496  0.241443  0.666 0.505309
## Smoking             0.520134  1.682253  0.259836  2.002 0.045309 *
## TG_total_log       -0.133156  0.875328  0.156426 -0.851 0.394635
## UAER_log            0.250605  1.284803  0.049331  5.080 3.77e-07 ***
## ---
## Signif. codes:  0 '***' 0.001 '**' 0.01 '*' 0.05 '.' 0.1 ' ' 1
##
##                    exp(coef) exp(-coef) lower .95 upper .95
## Ribonic_acid          2.2165     0.4512    1.6431     2.990
## Age                   0.9864     1.0138    0.9620     1.011
## BMI                   0.9951     1.0049    0.9396     1.054
## BP_Systolic           1.0261     0.9746    1.0126     1.040
## Cholesterol           1.1816     0.8463    0.9114     1.532
## eGFR                  0.9911     1.0090    0.9814     1.001
## HbA1c                 1.5244     0.6560    1.2915     1.799
## Medication_Statins    1.8813     0.5316    1.0190     3.473
## Sex                   1.1745     0.8514    0.7317     1.885
## Smoking               1.6823     0.5944    1.0109     2.799
## TG_total_log          0.8753     1.1424    0.6442     1.189
## UAER_log              1.2848     0.7783    1.1664     1.415
##
## Concordance= 0.856  (se = 0.032 )
## Rsquare= 0.25   (max possible= 0.828 )
## Likelihood ratio test= 168.2  on 12 df,   p=0
## Wald test            = 148.9  on 12 df,   p=0
## Score (logrank) test = 193  on 12 df,   p=0
```

4.1.1.1.1 Forest Plot with Clinical Variables

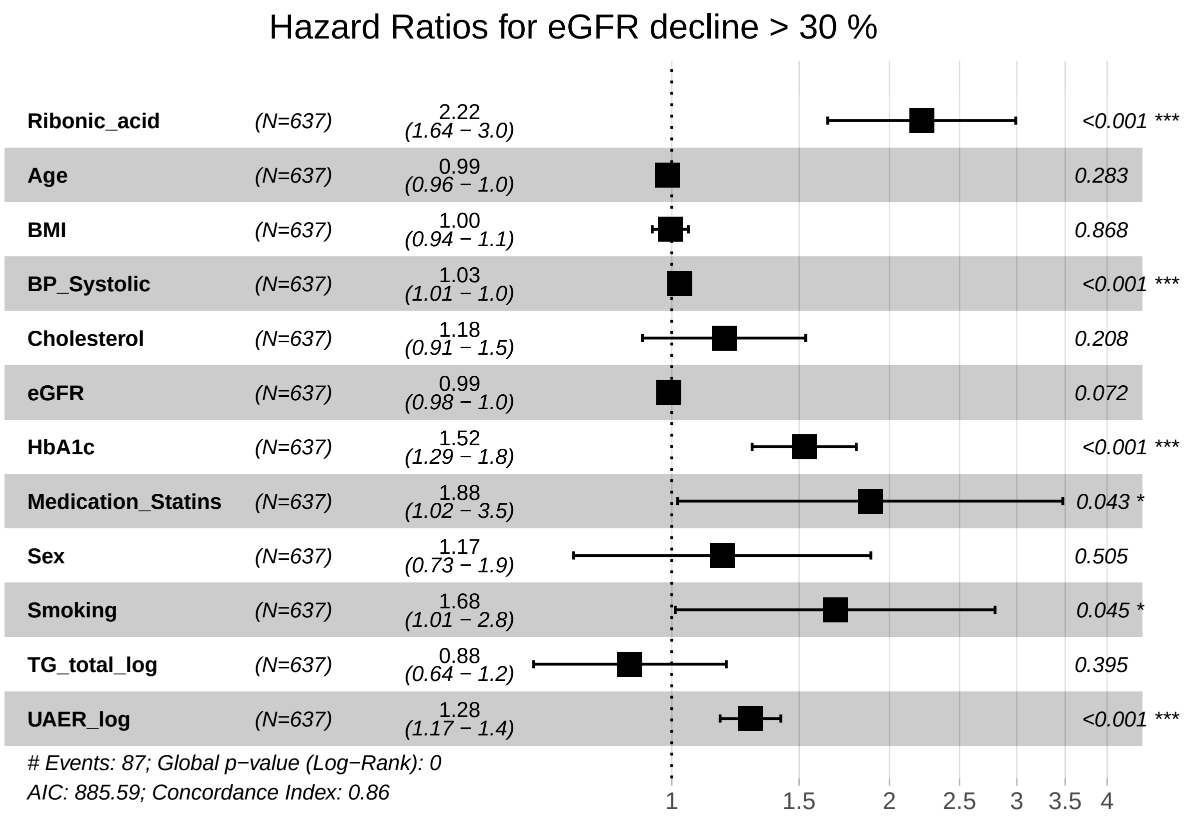
Hazard Ratios for eGFR decline > 30 %
Ribonic_acid (N=637) 2.22 (1.64 − 3.0) <0.001 ***
Age (N=637) 0.99 (0.96 − 1.0) 0.283
BMI (N=637) 1.00 (0.94 − 1.1) 0.868
BP_Systolic (N=637) 1.03 (1.01 − 1.0) <0.001 ***
Cholesterol (N=637) 1.18 (0.91 − 1.5) 0.208
eGFR (N=637) 0.99 (0.98 − 1.0) 0.072
HbA1c (N=637) 1.52 (1.29 − 1.8) <0.001 ***
Medication_Statins (N=637) 1.88 (1.02 − 3.5) 0.043 *
Sex (N=637) 1.17 (0.73 − 1.9) 0.505
Smoking (N=637) 1.68 (1.01 − 2.8) 0.045 *
TG_total_log (N=637) 0.88 (0.64 − 1.2) 0.395
UAER_log (N=637) 1.28 (1.17 − 1.4) <0.001 ***
Events: 87; Global p−value (Log−Rank): 0
AIC: 885.59; Concordance Index: 0.86
1 1.5 2 2.5 3 3.5 4

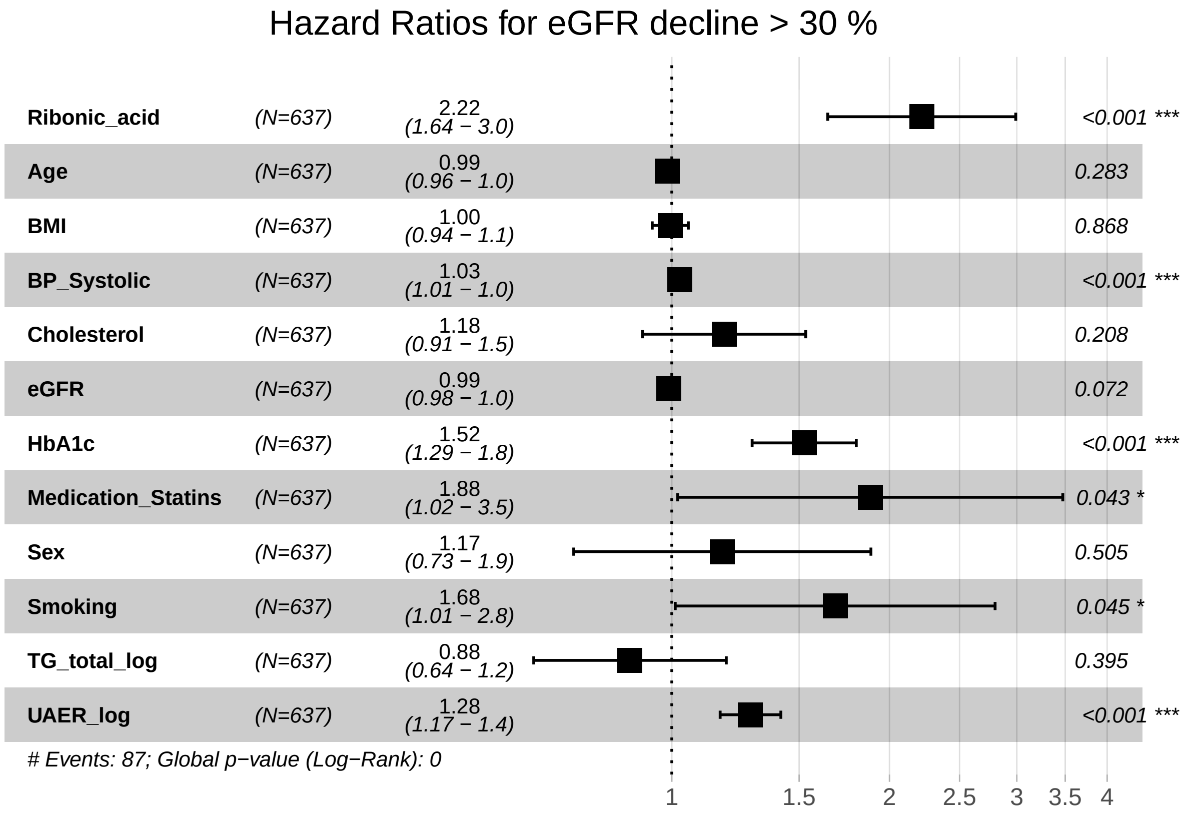
Hazard Ratios for eGFR decline > 30 %
Ribonic_acid (N=637) 2.22 (1.64 − 3.0) <0.001 ***
Age (N=637) 0.99 (0.96 − 1.0) 0.283
BMI (N=637) 1.00 (0.94 − 1.1) 0.868
BP_Systolic (N=637) 1.03 (1.01 − 1.0) <0.001 ***
Cholesterol (N=637) 1.18 (0.91 − 1.5) 0.208
eGFR (N=637) 0.99 (0.98 − 1.0) 0.072
HbA1c (N=637) 1.52 (1.29 − 1.8) <0.001 ***
Medication_Statins (N=637) 1.88 (1.02 − 3.5) 0.043 *
Sex (N=637) 1.17 (0.73 − 1.9) 0.505
Smoking (N=637) 1.68 (1.01 − 2.8) 0.045 *
TG_total_log (N=637) 0.88 (0.64 − 1.2) 0.395
UAER_log (N=637) 1.28 (1.17 − 1.4) <0.001 ***
Events: 87; Global p−value (Log−Rank): 0
1 1.5 2 2.5 3 3.5 4

4.1.1.1.2 Diagnostics of the Survival Model

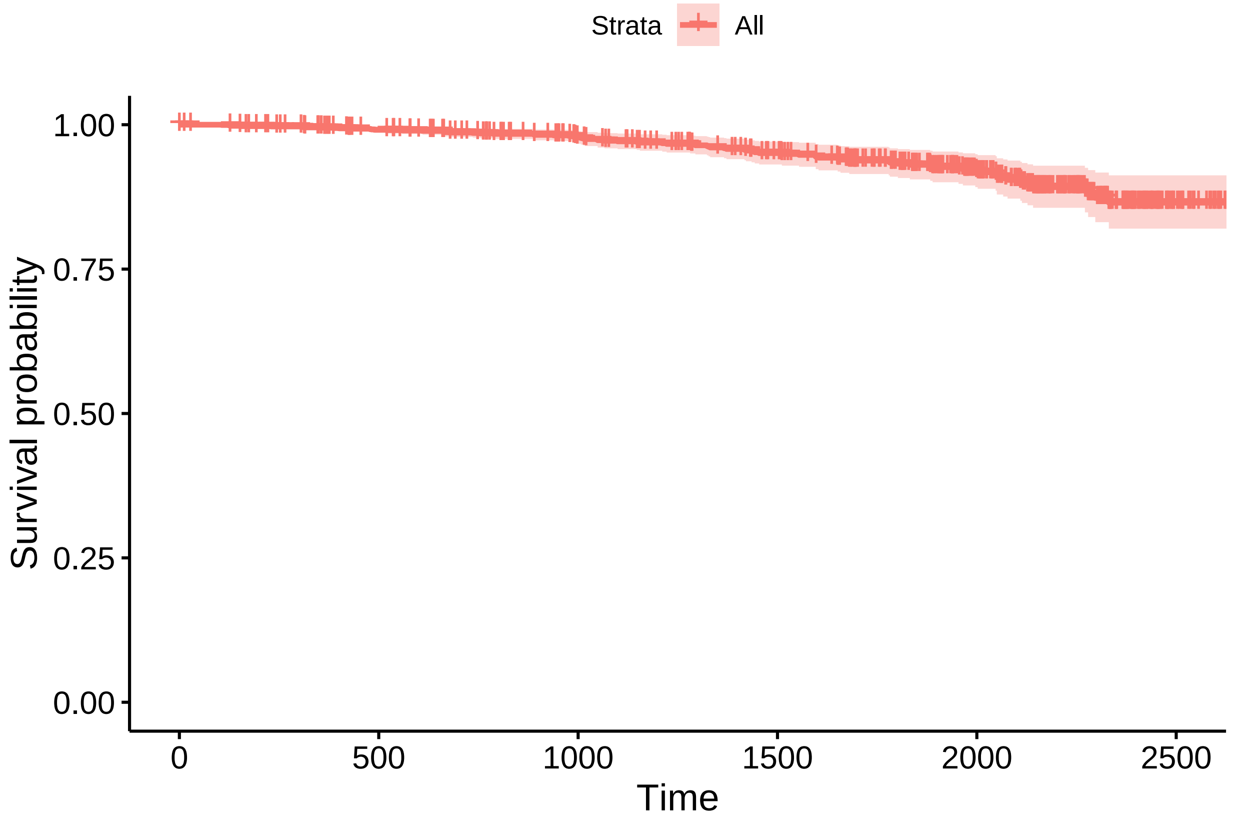

Strata
All
Survival probability
1.00
0.75
0.50
0.25
0.00
0
500
1000
1500
2000
2500
Time

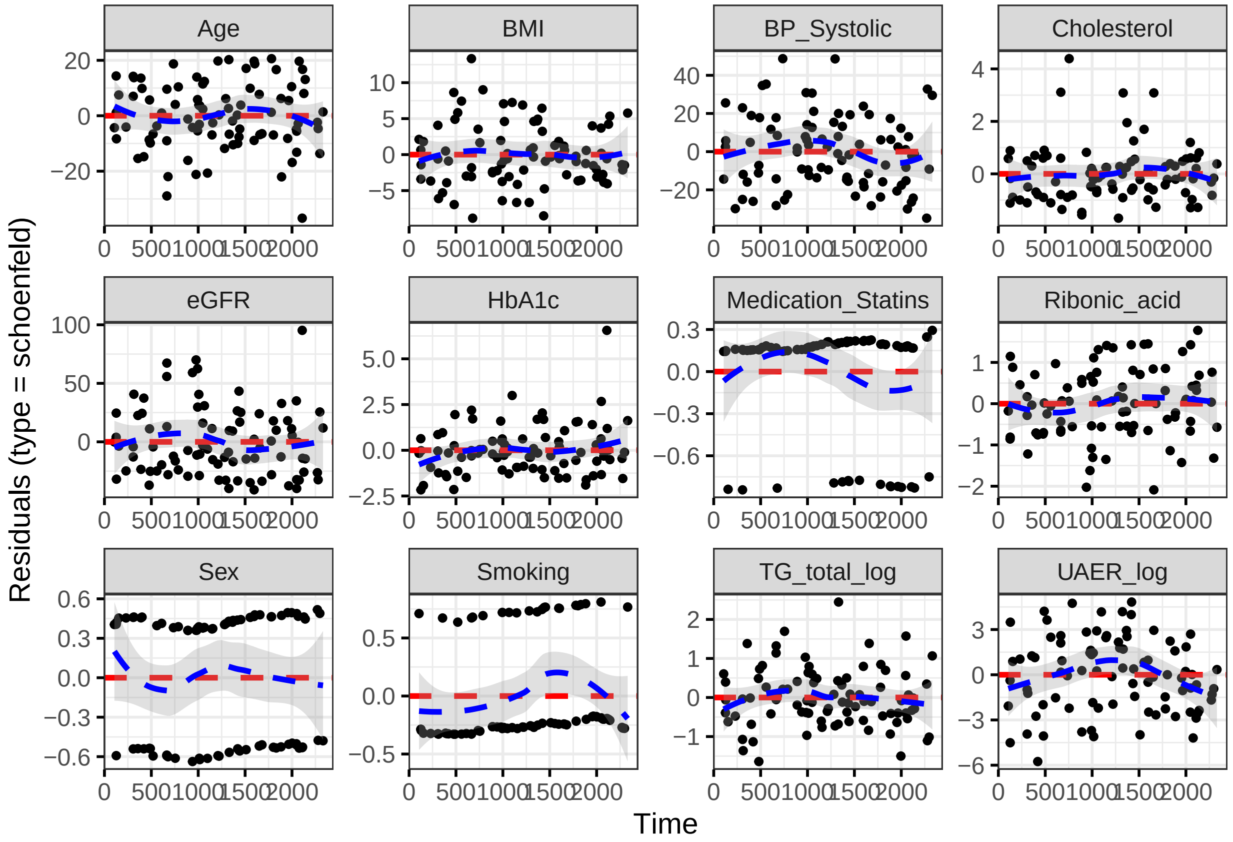

Age
BMI
BP_Systolic
Cholesterol
eGFR
HbA1c
Medication_Statins
Ribonic_acid
Sex
Smoking
TG_total_log
UAER_log
Residuals (type = schoenfeld)
Time

4.1.1.1.3 Kaplan-Maier Curve with Median Cutpoint

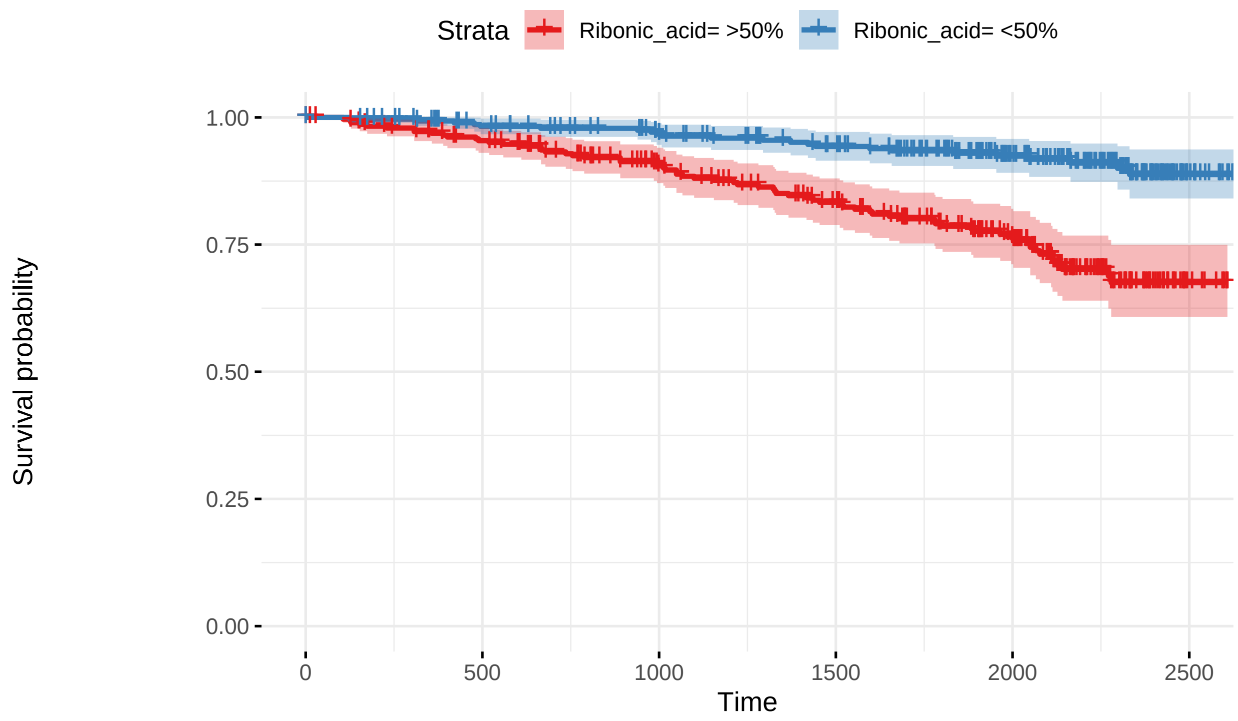

Number at risk

	0	500	1000	1500	2000	2500
Ribonic_acid= >50%	318	266	221	186	129	9
Ribonic_acid= <50%	319	272	250	227	156	18

Cumulative number of events

	0	500	1000	1500	2000	2500
Ribonic_acid= >50%	0	13	26	43	57	68
Ribonic_acid= <50%	0	5	9	15	19	23

4.1.1.2 Other Model Fits

```
## Call:
## survival::coxph(formula = survival::Surv(time = t_gfrfald30_p,
##     event = censor_gfrfald30_p.reversed.numeric) ~ Ribonic_acid,
##     data = data.km)
##
##   n= 637, number of events= 91
##
##                       coef exp(coef) se(coef)      z Pr(>|z|)
## Ribonic_acid <50% -1.2489    0.2868   0.2416 -5.168 2.36e-07 ***
## ---
## Signif. codes:  0 '***' 0.001 '**' 0.01 '*' 0.05 '.' 0.1 ' ' 1
##
##                   exp(coef) exp(-coef) lower .95 upper .95
## Ribonic_acid <50%    0.2868      3.486    0.1786    0.4606
##
## Concordance= 0.643  (se = 0.027 )
## Rsquare= 0.048   (max possible= 0.82 )
## Likelihood ratio test= 31.16  on 1 df,   p=2.373e-08
## Wald test            = 26.71  on 1 df,   p=2.362e-07
## Score (logrank) test = 30.32  on 1 df,   p=3.657e-08
```

```
##
## Call:
## glm(formula = censor_gfrfald30_p.reversed.numeric ~ Ribonic_acid +
##     Age + BMI + BP_Systolic + Cholesterol + eGFR + HbA1c + Medication_Statins +
##     Sex + Smoking + TG_total_log + UAER_log, data = data.survival)
##
## Deviance Residuals:
##      Min        1Q    Median        3Q       Max
## -0.61675  -0.18069  -0.07320   0.03048   0.97468
##
## Coefficients:
##                      Estimate Std. Error t value Pr(>|t|)
## (Intercept)        -1.6578319  0.3755304  -4.415 1.21e-05 ***
## Ribonic_acid        0.0535506  0.0152068   3.521 0.000463 ***
## Age                -0.0015186  0.0012683  -1.197 0.231660
## BMI                 0.0001000  0.0035353   0.028 0.977441
## BP_Systolic         0.0020542  0.0008328   2.467 0.013929 *
## Cholesterol         0.0133323  0.0166123   0.803 0.422565
## eGFR               -0.0011053  0.0005952  -1.857 0.063817 .
## HbA1c               0.0483914  0.0126149   3.836 0.000139 ***
## Medication_Statins  0.0309564  0.0312543   0.990 0.322363
## Sex                 0.0026786  0.0282635   0.095 0.924530
## Smoking             0.0158713  0.0337962   0.470 0.638806
## TG_total_log        0.0042657  0.0224742   0.190 0.849528
## UAER_log            0.0342759  0.0070140   4.887 1.33e-06 ***
## ---
## Signif. codes:  0 '***' 0.001 '**' 0.01 '*' 0.05 '.' 0.1 ' ' 1
##
## (Dispersion parameter for gaussian family taken to be 0.1029239)
##
##     Null deviance: 74.084  on 585  degrees of freedom
## Residual deviance: 58.975  on 573  degrees of freedom
```

```
##   (51 observations deleted due to missingness)
## AIC: 345.42
##
## Number of Fisher Scoring iterations: 2
```

```
##
## Call:
## lm(formula = Ribonic_acid ~ censor_gfrfald30_p.reversed.numeric +
##     Age + BMI + BP_Systolic + Cholesterol + eGFR + HbA1c + Medication_Statins +
##     Sex + Smoking + TG_total_log + UAER_log, data = data.survival)
##
## Residuals:
##     Min      1Q  Median      3Q     Max
## -4.7948 -0.4246  0.0734  0.5288  2.4406
##
## Coefficients:
##                                      Estimate Std. Error t value Pr(>|t|)
## (Intercept)                         21.6465792  0.5093357  42.500  < 2e-16
## censor_gfrfald30_p.reversed.numeric  0.3955822  0.1123335   3.521 0.000463
## Age                                  0.0062157  0.0034417   1.806 0.071438
## BMI                                 -0.0175379  0.0095806  -1.831 0.067686
## BP_Systolic                          0.0007921  0.0022752   0.348 0.727843
## Cholesterol                         -0.1050340  0.0449626  -2.336 0.019834
## eGFR                                -0.0128740  0.0015308  -8.410 3.27e-16
## HbA1c                               -0.0242801  0.0347090  -0.700 0.484502
## Medication_Statins                  -0.0782850  0.0849562  -0.921 0.357190
## Sex                                 -0.0712927  0.0767606  -0.929 0.353401
## Smoking                             -0.2440951  0.0913053  -2.673 0.007723
## TG_total_log                         0.1524392  0.0607520   2.509 0.012375
## UAER_log                             0.0331404  0.0194074   1.708 0.088249
##
## (Intercept)                         ***
## censor_gfrfald30_p.reversed.numeric ***
## Age                                 .
## BMI                                 .
## BP_Systolic
## Cholesterol                         *
## eGFR                                ***
## HbA1c
## Medication_Statins
## Sex
## Smoking                             **
## TG_total_log                        *
## UAER_log                            .
## ---
## Signif. codes:  0 '***' 0.001 '**' 0.01 '*' 0.05 '.' 0.1 ' ' 1
##
## Residual standard error: 0.872 on 573 degrees of freedom
##   (51 observations deleted due to missingness)
## Multiple R-squared:  0.2593, Adjusted R-squared:  0.2438
## F-statistic: 16.71 on 12 and 573 DF,  p-value: < 2.2e-16
```

4.1.2 Step 4.1B: Analysis of a Blood Pressure, HbA1c and logUAER-Matched Subcohort

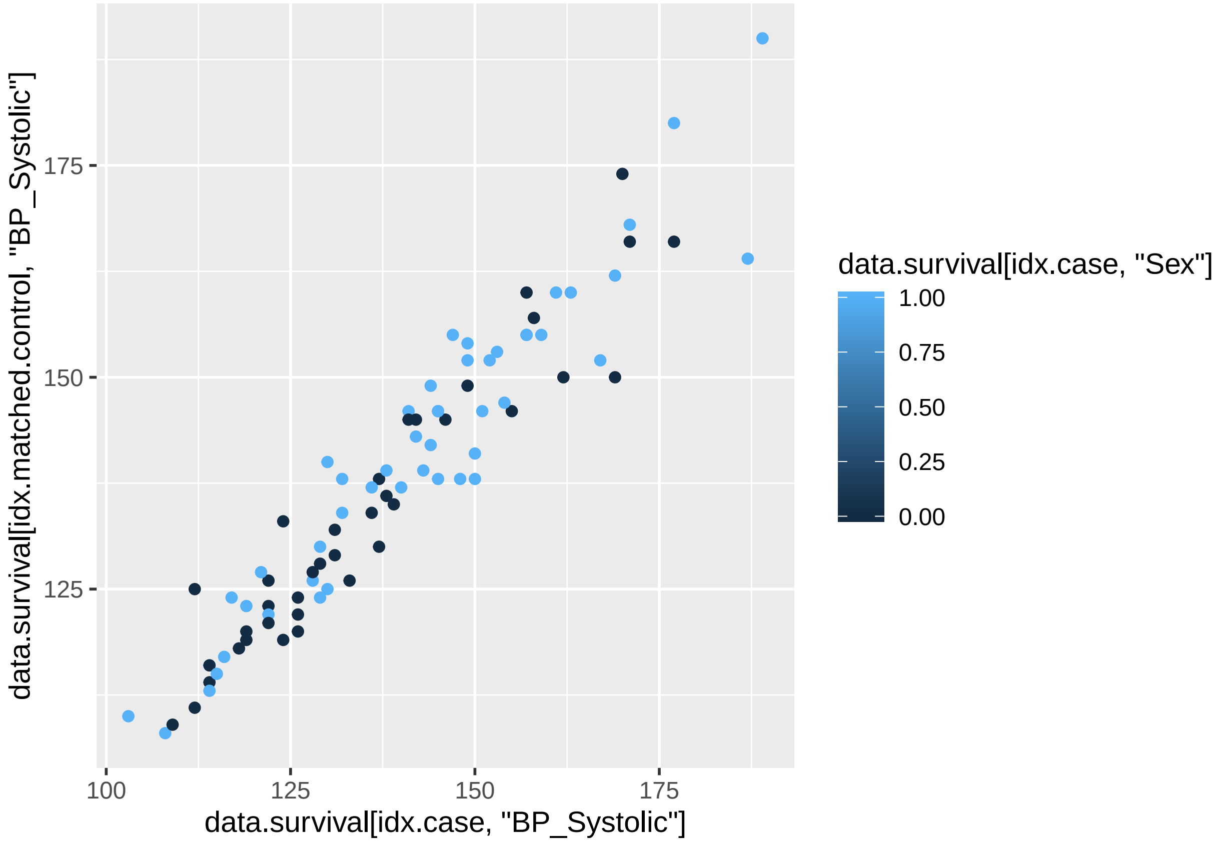

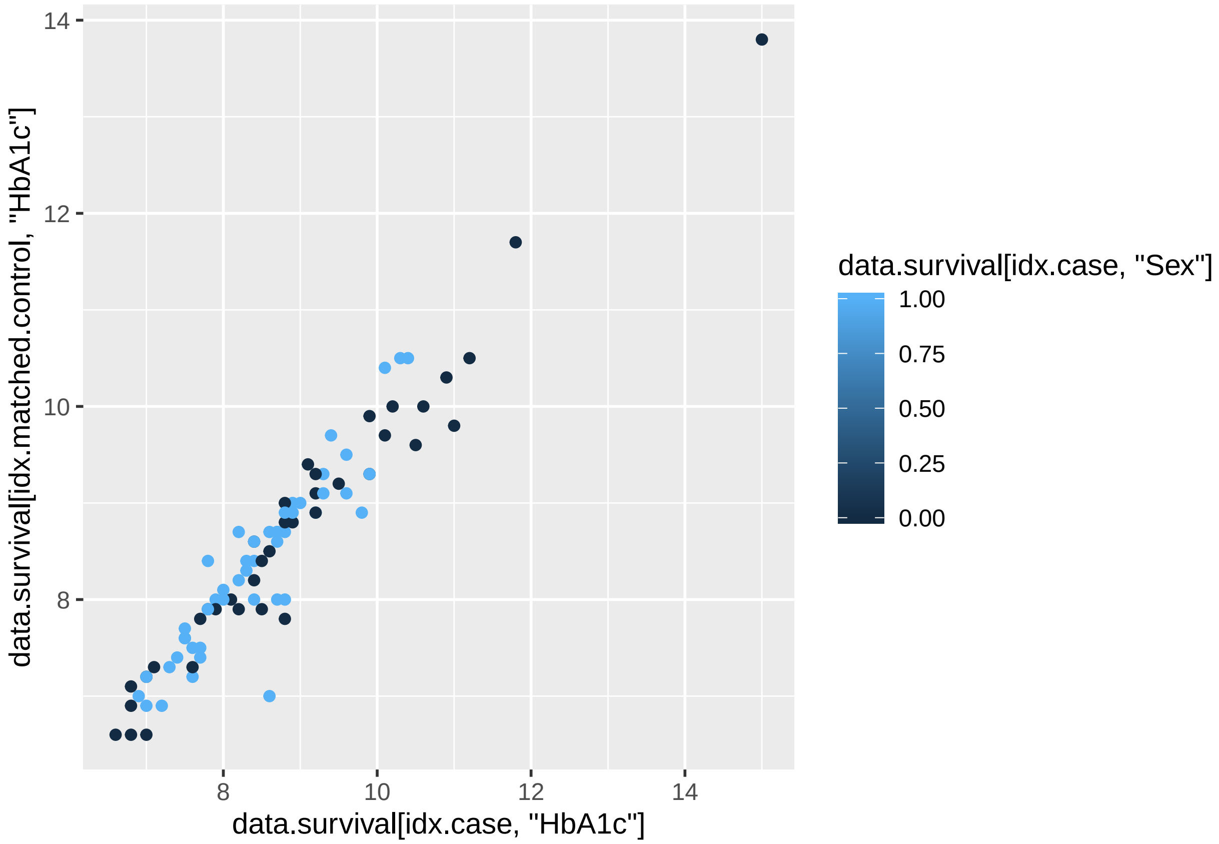

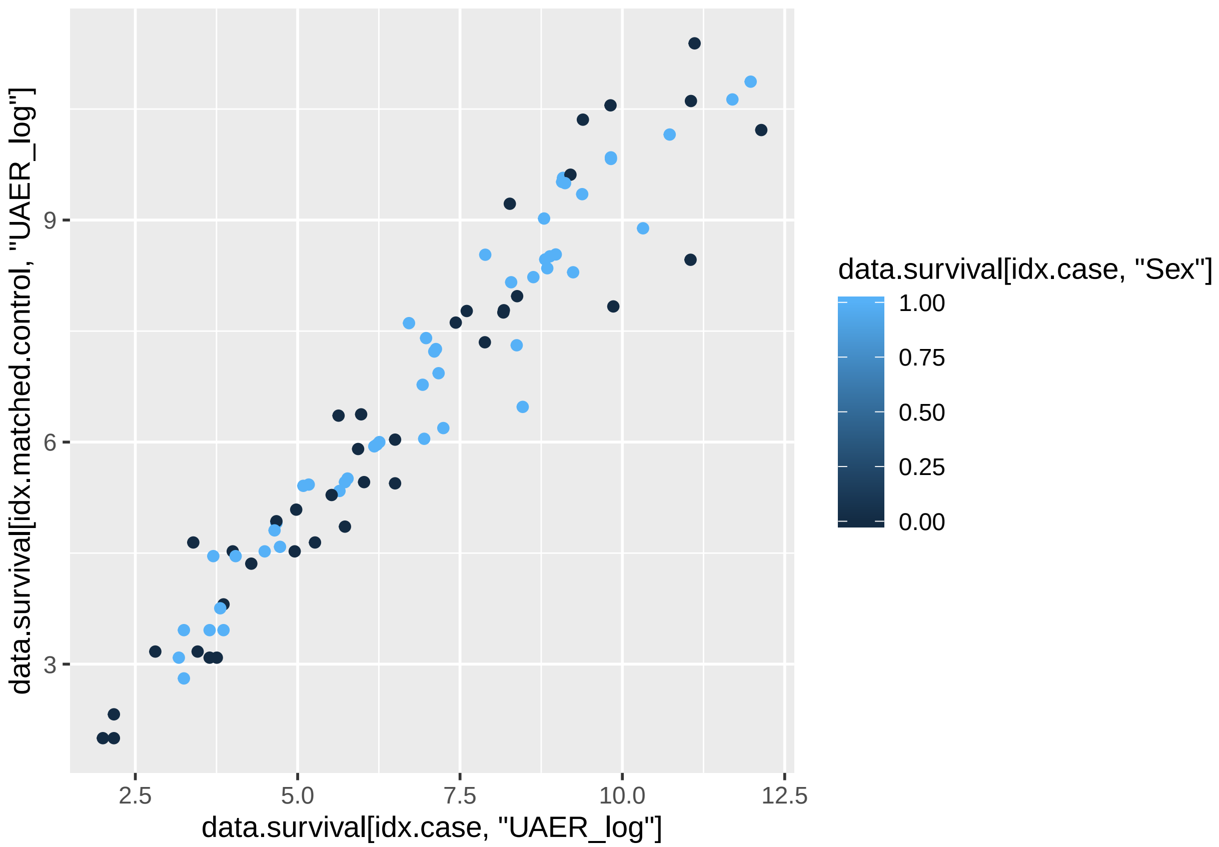


```
##
##  Paired t-test
##
## data:  data.survival[idx.case, "BP_Systolic"] and data.survival[idx.matched.control, "BP_Systolic"]
## t = 1.9697, df = 86, p-value = 0.05209
## alternative hypothesis: true difference in means is not equal to 0
## 95 percent confidence interval:
##  -0.01150946  2.49426809
## sample estimates:
## mean of the differences
##                1.241379

##
##  Paired t-test
##
## data:  data.survival[idx.case, "HbA1c"] and data.survival[idx.matched.control, "HbA1c"]
## t = 3.5465, df = 86, p-value = 0.0006347
## alternative hypothesis: true difference in means is not equal to 0
## 95 percent confidence interval:
##  0.06465628 0.22959659
## sample estimates:
## mean of the differences
##               0.1471264

##
##  Paired t-test
##
```

```
## data:  data.survival[idx.case, "UAER_log"] and data.survival[idx.matched.control, "UAER_log"]
## t = 2.6454, df = 86, p-value = 0.0097
## alternative hypothesis: true difference in means is not equal to 0
## 95 percent confidence interval:
##  0.04758177 0.33532528
## sample estimates:
## mean of the differences
##               0.1914535
```

```
##
## Call:
## glm(formula = censor_gfrfald30_p.reversed.numeric ~ Ribonic_acid +
##     Age + BMI + BP_Systolic + Cholesterol + eGFR + HbA1c + Medication_Statins +
##     Sex + Smoking + TG_total_log + UAER_log, data = data.survival.stratified)
##
## Deviance Residuals:
##     Min       1Q   Median       3Q      Max
## -0.8455  -0.4372   0.1147   0.4262   0.8077
##
## Coefficients:
##                      Estimate Std. Error t value Pr(>|t|)
## (Intercept)        -4.0993491  1.1131140  -3.683 0.000315 ***
## Ribonic_acid        0.1837293  0.0452519   4.060 7.64e-05 ***
## Age                -0.0030646  0.0037464  -0.818 0.414560
## BMI                 0.0072583  0.0098849   0.734 0.463844
## BP_Systolic         0.0017581  0.0022430   0.784 0.434311
## Cholesterol         0.0735053  0.0449518   1.635 0.103961
## eGFR                0.0003542  0.0015430   0.230 0.818732
## HbA1c               0.0228773  0.0322882   0.709 0.479639
## Medication_Statins  0.1708596  0.0954080   1.791 0.075199 .
## Sex                -0.0138969  0.0793396  -0.175 0.861176
## Smoking            -0.0090528  0.0889141  -0.102 0.919030
## TG_total_log       -0.0582056  0.0653695  -0.890 0.374575
## UAER_log           -0.0092884  0.0175963  -0.528 0.598322
## ---
## Signif. codes:  0 '***' 0.001 '**' 0.01 '*' 0.05 '.' 0.1 ' ' 1
##
## (Dispersion parameter for gaussian family taken to be 0.2334169)
##
##     Null deviance: 43.50  on 173  degrees of freedom
## Residual deviance: 37.58  on 161  degrees of freedom
## AIC: 255.12
##
## Number of Fisher Scoring iterations: 2
```

```
##
## Call:
## lm(formula = Ribonic_acid ~ censor_gfrfald30_p.reversed.numeric +
##     Age + BMI + BP_Systolic + Cholesterol + eGFR + HbA1c + Medication_Statins +
##     Sex + Smoking + TG_total_log + UAER_log, data = data.survival.stratified)
##
## Residuals:
##      Min       1Q   Median       3Q      Max
## -3.12668 -0.50130  0.06296  0.56076  1.76793
##
```

```
## Coefficients:
##                                   Estimate Std. Error t value Pr(>|t|)
## (Intercept)                      21.594329   0.894405  24.144  < 2e-16
## censor_gfrfald30_p.reversed.numeric  0.505526   0.124509   4.060 7.64e-05
## Age                               0.013026   0.006142   2.121   0.0355
## BMI                              -0.007618   0.016413  -0.464   0.6432
## BP_Systolic                      -0.004314   0.003712  -1.162   0.2469
## Cholesterol                      -0.131182   0.074467  -1.762   0.0800
## eGFR                             -0.014280   0.002299  -6.211 4.32e-09
## HbA1c                             0.027012   0.053599   0.504   0.6150
## Medication_Statins               -0.332646   0.157662  -2.110   0.0364
## Sex                               0.038533   0.131583   0.293   0.7700
## Smoking                           0.024815   0.147479   0.168   0.8666
## TG_total_log                      0.166543   0.107903   1.543   0.1247
## UAER_log                          0.012077   0.029198   0.414   0.6797
##
## (Intercept)                      ***
## censor_gfrfald30_p.reversed.numeric ***
## Age                              *
## BMI
## BP_Systolic
## Cholesterol                      .
## eGFR                             ***
## HbA1c
## Medication_Statins               *
## Sex
## Smoking
## TG_total_log
## UAER_log
## ---
## Signif. codes:  0 '***' 0.001 '**' 0.01 '*' 0.05 '.' 0.1 ' ' 1
##
## Residual standard error: 0.8014 on 161 degrees of freedom
## Multiple R-squared:  0.3672, Adjusted R-squared:  0.3201
## F-statistic: 7.787 on 12 and 161 DF,  p-value: 2.516e-11
```

4.1.2.1 Survival Model with Details

```
## Call:
## survival::coxph(formula = survival::Surv(time = t_gfrfald30_p,
##     event = censor_gfrfald30_p.reversed.numeric) ~ Ribonic_acid +
##     Age + BMI + BP_Systolic + Cholesterol + eGFR + HbA1c + Medication_Statins +
##     Sex + Smoking + TG_total_log + UAER_log, data = data.survival.stratified)
##
##   n= 174, number of events= 87
##
##                         coef exp(coef)  se(coef)      z Pr(>|z|)
## Ribonic_acid        0.647424  1.910612  0.139489  4.641 3.46e-06 ***
## Age                -0.022737  0.977520  0.012590 -1.806   0.0709 .
## BMI                 0.017895  1.018056  0.030824  0.581   0.5615
## BP_Systolic         0.012267  1.012342  0.006593  1.861   0.0628 .
## Cholesterol         0.302785  1.353623  0.141856  2.134   0.0328 *
## eGFR               -0.005625  0.994390  0.004827 -1.165   0.2439
## HbA1c               0.092383  1.096785  0.090216  1.024   0.3058
```

```
## Medication_Statins  0.739185  2.094229  0.306796  2.409   0.0160 *
## Sex                 0.074701  1.077561  0.237519  0.315   0.7531
## Smoking             0.340014  1.404967  0.267334  1.272   0.2034
## TG_total_log       -0.245832  0.782054  0.166331 -1.478   0.1394
## UAER_log            0.071898  1.074546  0.049898  1.441   0.1496
## ---
## Signif. codes:  0 '***' 0.001 '**' 0.01 '*' 0.05 '.' 0.1 ' ' 1
##
##                    exp(coef) exp(-coef) lower .95 upper .95
## Ribonic_acid          1.9106     0.5234    1.4536     2.511
## Age                   0.9775     1.0230    0.9537     1.002
## BMI                   1.0181     0.9823    0.9584     1.081
## BP_Systolic           1.0123     0.9878    0.9993     1.026
## Cholesterol           1.3536     0.7388    1.0251     1.787
## eGFR                  0.9944     1.0056    0.9850     1.004
## HbA1c                 1.0968     0.9118    0.9190     1.309
## Medication_Statins    2.0942     0.4775    1.1478     3.821
## Sex                   1.0776     0.9280    0.6765     1.716
## Smoking               1.4050     0.7118    0.8320     2.373
## TG_total_log          0.7821     1.2787    0.5645     1.083
## UAER_log              1.0745     0.9306    0.9744     1.185
##
## Concordance= 0.692  (se = 0.034 )
## Rsquare= 0.229   (max possible= 0.988 )
## Likelihood ratio test= 45.2  on 12 df,   p=9.511e-06
## Wald test            = 40.35  on 12 df,   p=6.3e-05
## Score (logrank) test = 41.67  on 12 df,   p=3.786e-05
```

4.1.2.1.1 Forest Plot with Clinical Variables

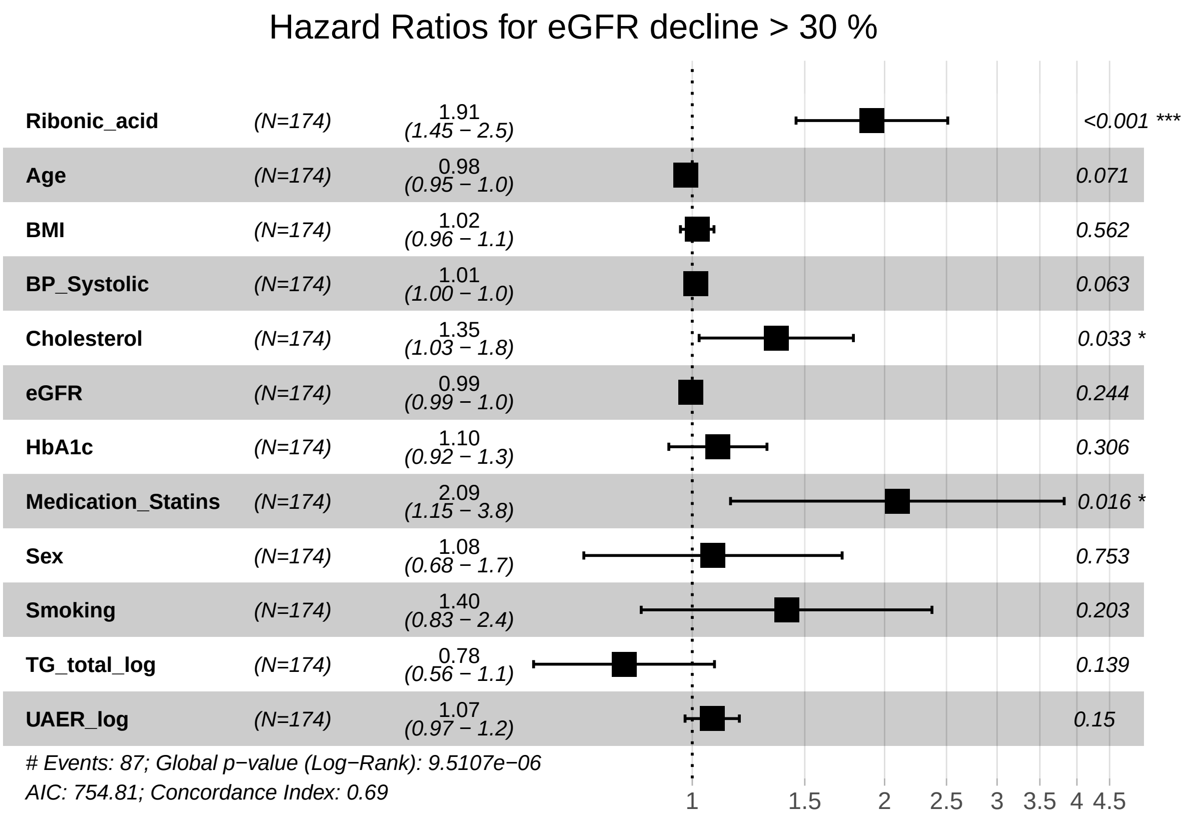
Hazard Ratios for eGFR decline > 30 %
Ribonic_acid (N=174) 1.91 (1.45 − 2.5) <0.001 ***
Age (N=174) 0.98 (0.95 − 1.0) 0.071
BMI (N=174) 1.02 (0.96 − 1.1) 0.562
BP_Systolic (N=174) 1.01 (1.00 − 1.0) 0.063
Cholesterol (N=174) 1.35 (1.03 − 1.8) 0.033 *
eGFR (N=174) 0.99 (0.99 − 1.0) 0.244
HbA1c (N=174) 1.10 (0.92 − 1.3) 0.306
Medication_Statins (N=174) 2.09 (1.15 − 3.8) 0.016 *
Sex (N=174) 1.08 (0.68 − 1.7) 0.753
Smoking (N=174) 1.40 (0.83 − 2.4) 0.203
TG_total_log (N=174) 0.78 (0.56 − 1.1) 0.139
UAER_log (N=174) 1.07 (0.97 − 1.2) 0.15
Events: 87; Global p−value (Log−Rank): 9.5107e−06
AIC: 754.81; Concordance Index: 0.69
1 1.5 2 2.5 3 3.5 4 4.5

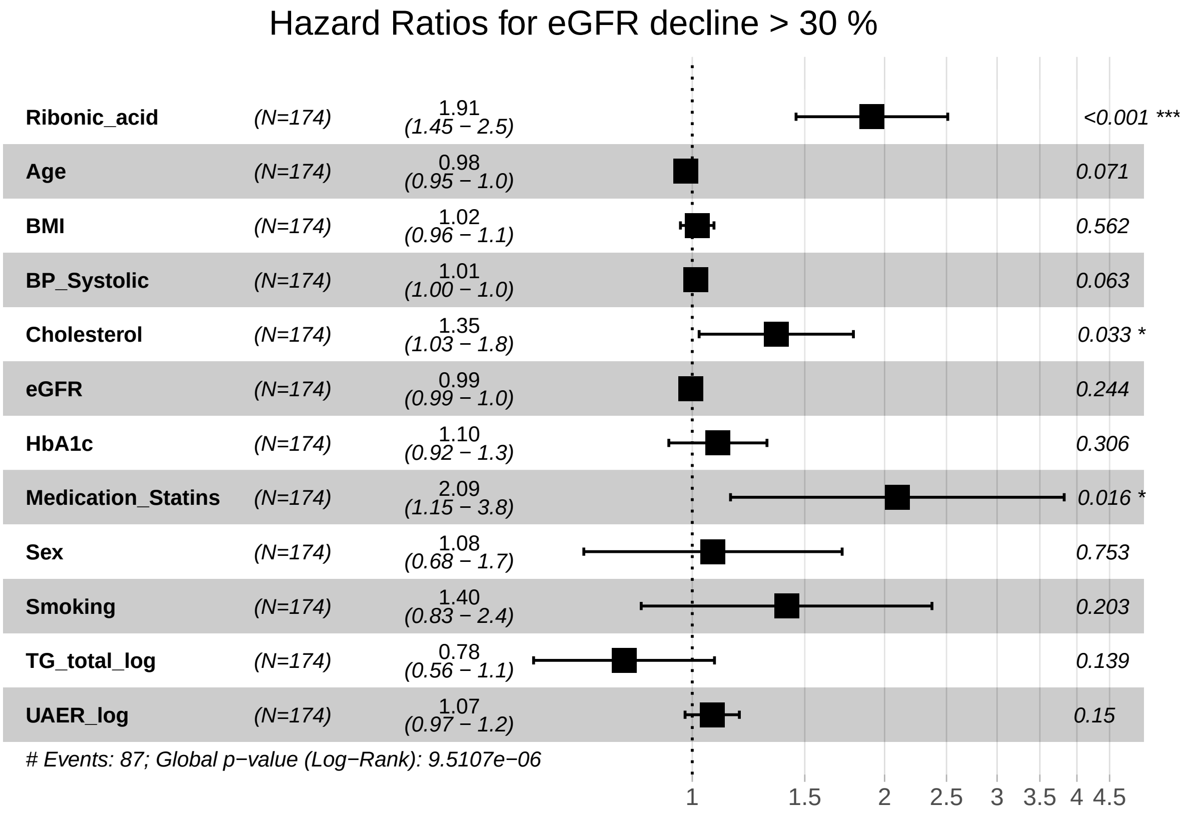
Hazard Ratios for eGFR decline > 30 %
Ribonic_acid (N=174) 1.91 (1.45 − 2.5) <0.001 ***
Age (N=174) 0.98 (0.95 − 1.0) 0.071
BMI (N=174) 1.02 (0.96 − 1.1) 0.562
BP_Systolic (N=174) 1.01 (1.00 − 1.0) 0.063
Cholesterol (N=174) 1.35 (1.03 − 1.8) 0.033 *
eGFR (N=174) 0.99 (0.99 − 1.0) 0.244
HbA1c (N=174) 1.10 (0.92 − 1.3) 0.306
Medication_Statins (N=174) 2.09 (1.15 − 3.8) 0.016 *
Sex (N=174) 1.08 (0.68 − 1.7) 0.753
Smoking (N=174) 1.40 (0.83 − 2.4) 0.203
TG_total_log (N=174) 0.78 (0.56 − 1.1) 0.139
UAER_log (N=174) 1.07 (0.97 − 1.2) 0.15
Events: 87; Global p−value (Log−Rank): 9.5107e−06
1 1.5 2 2.5 3 3.5 4 4.5

4.1.2.1.2 Diagnostics of the Survival Model

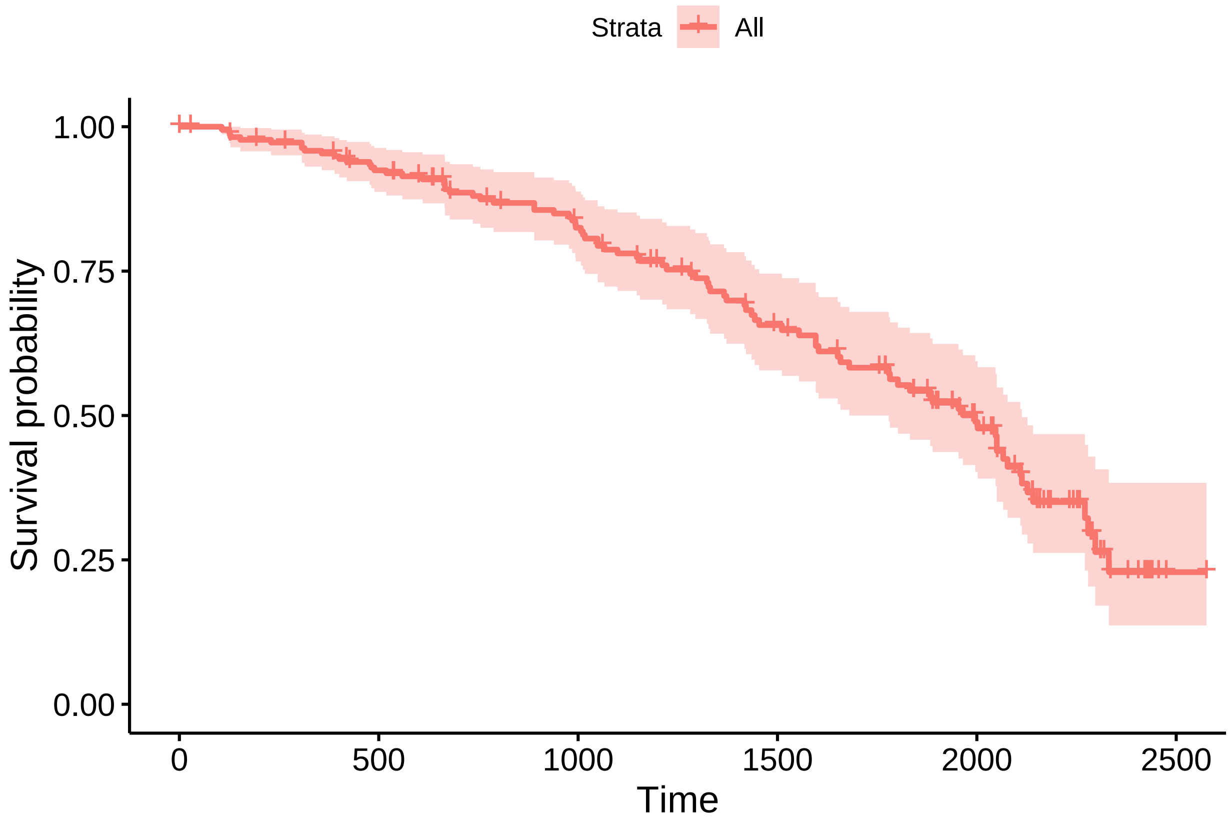
Strata
All
Survival probability
1.00
0.75
0.50
0.25
0.00
0
500
1000
1500
2000
2500
Time

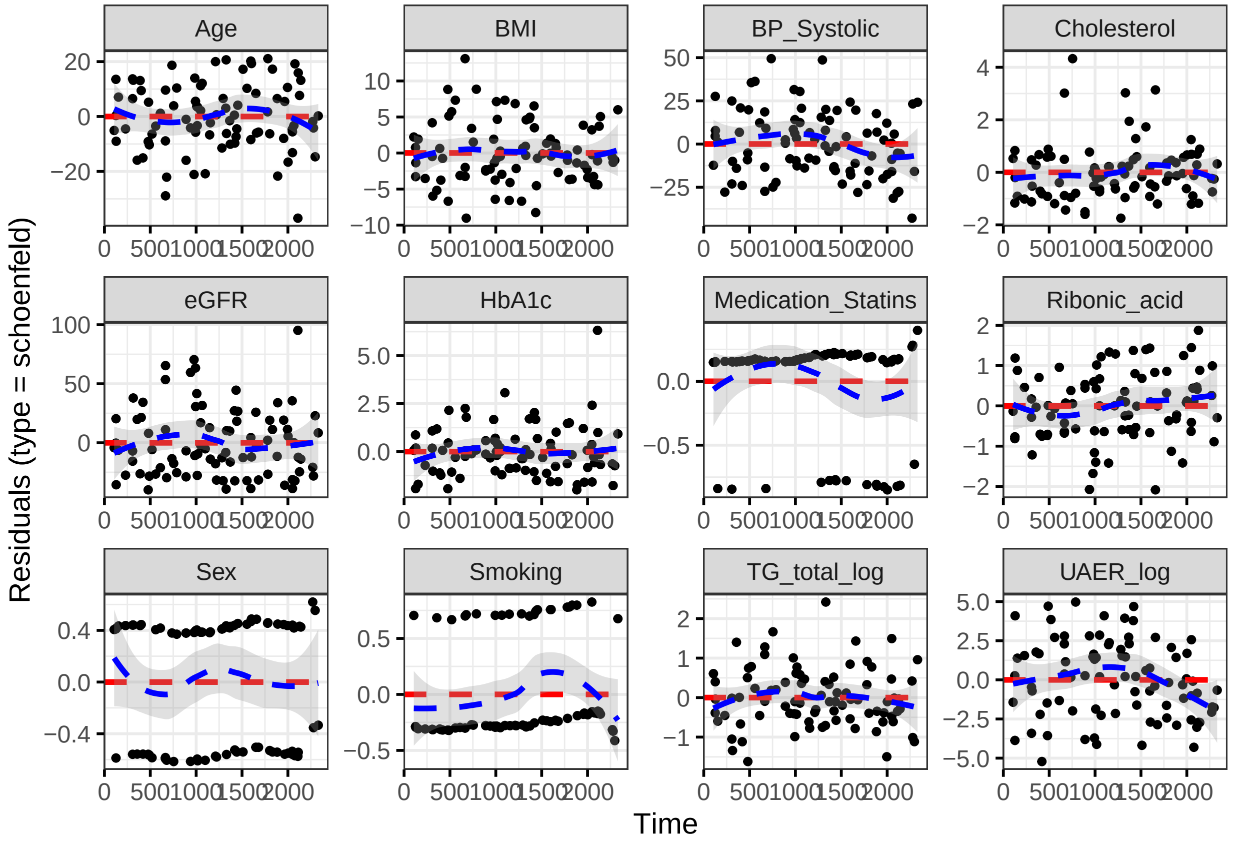
Age
BMI
BP_Systolic
Cholesterol
eGFR
HbA1c
Medication_Statins
Ribonic_acid
Sex
Smoking
TG_total_log
UAER_log
Residuals (type = schoenfeld)
Time

4.1.2.1.3 Kaplan-Maier Curve with Median Cutpoint

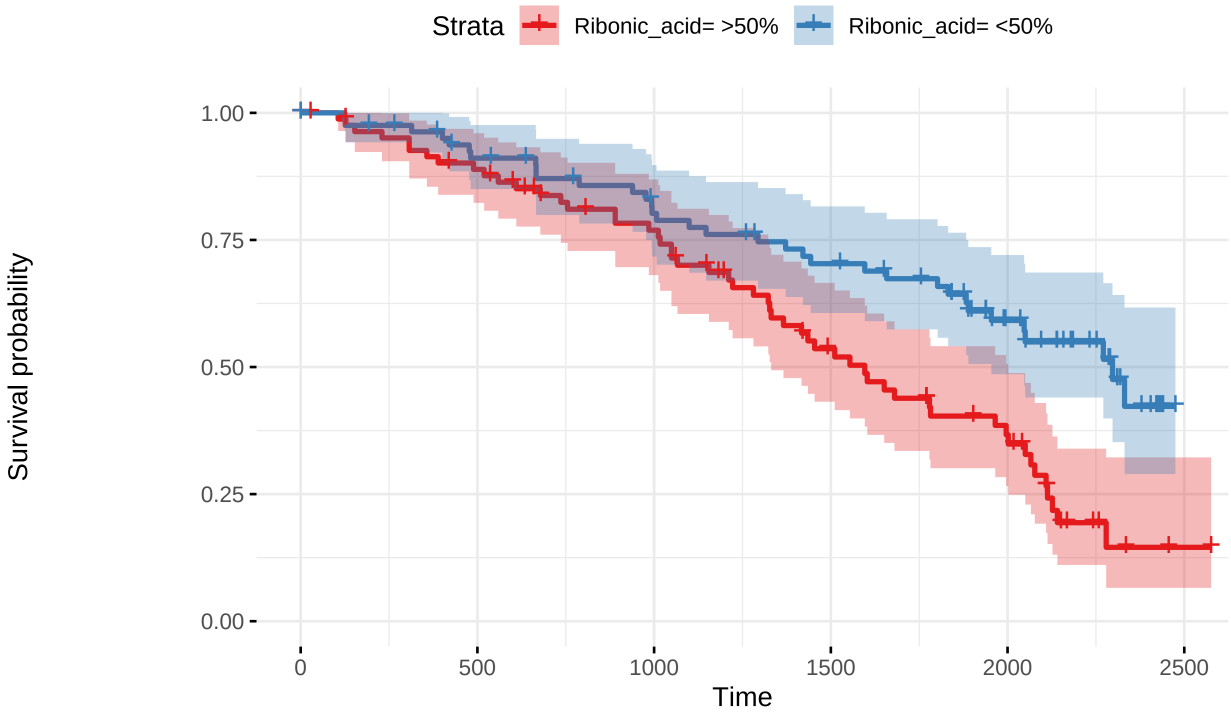

Number at risk

	0	500	1000	1500	2000	2500
Ribonic_acid= >50%	87	71	56	33	20	1
Ribonic_acid= <50%	87	70	58	49	29	0

Cumulative number of events

	0	500	1000	1500	2000	2500
Ribonic_acid= >50%	0	9	18	34	44	53
Ribonic_acid= <50%	0	7	15	22	29	34

4.1.2.1.4 Boxplots

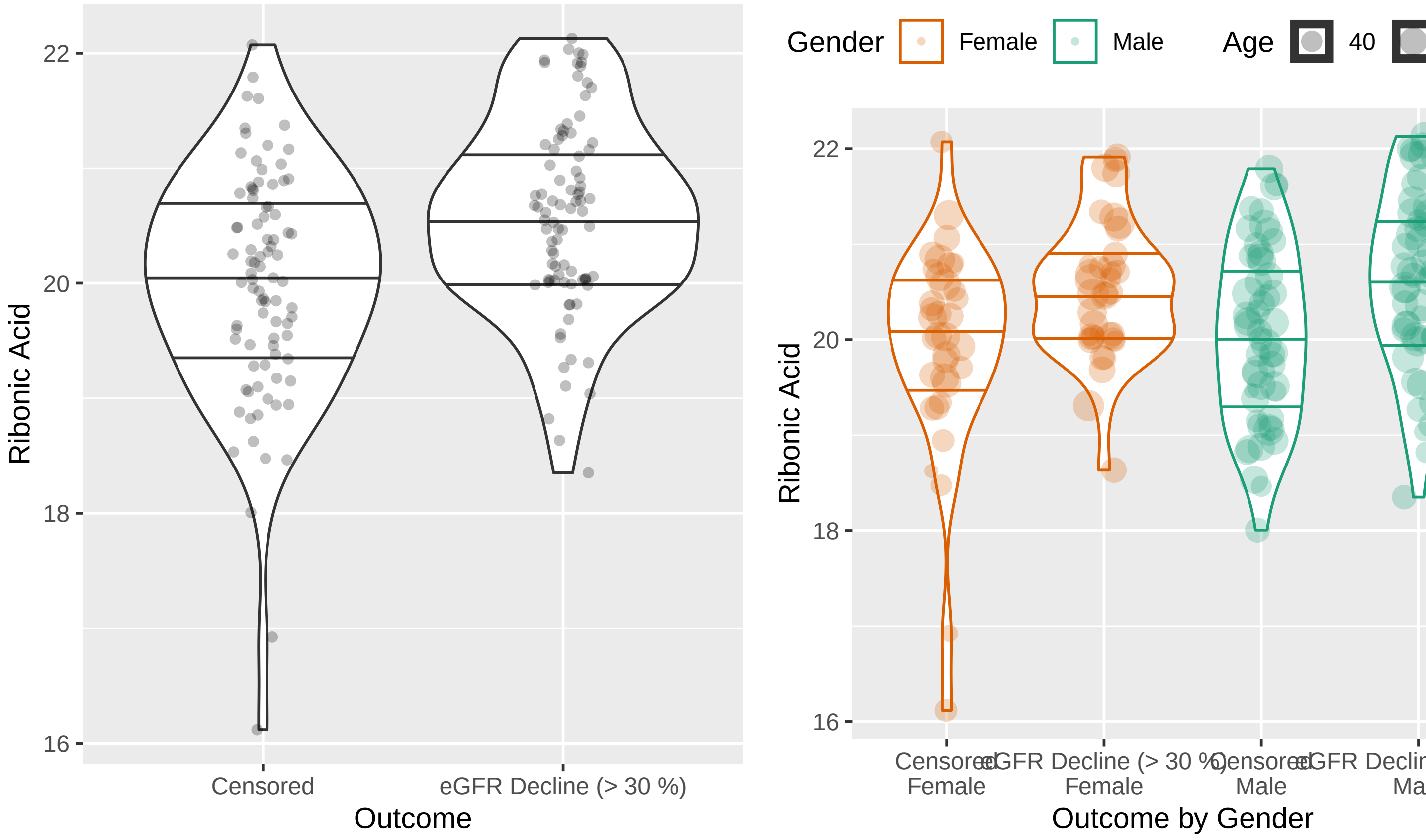

4.2 Step 4.2: Second Top-Metabolite in Relation to eGFR Decline (> 30 %)

4.2.1 Step 4.2A: Analysis of Full Cohort

4.2.1.1 Survival Model with Details

```
## Call:
## survival::coxph(formula = survival::Surv(time = t_gfrfald30_p,
##     event = censor_gfrfald30_p.reversed.numeric) ~ Myo_Inositol +
##     Age + BMI + BP_Systolic + Cholesterol + eGFR + HbA1c + Medication_Statins +
##     Sex + Smoking + TG_total_log + UAER_log, data = data.survival)
##
##   n= 586, number of events= 87
##    (51 observations deleted due to missingness)
##
##                          coef exp(coef)  se(coef)      z Pr(>|z|)
## Myo_Inositol        0.979015  2.661832  0.249455  3.925 8.69e-05 ***
## Age                -0.010428  0.989626  0.012502 -0.834 0.404198
## BMI                -0.002433  0.997570  0.028700 -0.085 0.932436
## BP_Systolic         0.022613  1.022870  0.006742  3.354 0.000796 ***
## Cholesterol         0.088395  1.092420  0.125917  0.702 0.482674
## eGFR               -0.010354  0.989699  0.005316 -1.948 0.051459 .
## HbA1c               0.358775  1.431575  0.087124  4.118 3.82e-05 ***
## Medication_Statins  0.523254  1.687511  0.310927  1.683 0.092398 .
## Sex                 0.182704  1.200459  0.235690  0.775 0.438229
## Smoking             0.417360  1.517950  0.258445  1.615 0.106335
## TG_total_log       -0.088135  0.915637  0.163414 -0.539 0.589652
## UAER_log            0.237250  1.267759  0.049452  4.798 1.61e-06 ***
## ---
## Signif. codes:  0 '***' 0.001 '**' 0.01 '*' 0.05 '.' 0.1 ' ' 1
##
##                    exp(coef) exp(-coef) lower .95 upper .95
## Myo_Inositol          2.6618     0.3757    1.6325     4.340
## Age                   0.9896     1.0105    0.9657     1.014
## BMI                   0.9976     1.0024    0.9430     1.055
## BP_Systolic           1.0229     0.9776    1.0094     1.036
## Cholesterol           1.0924     0.9154    0.8535     1.398
## eGFR                  0.9897     1.0104    0.9794     1.000
## HbA1c                 1.4316     0.6985    1.2069     1.698
## Medication_Statins    1.6875     0.5926    0.9175     3.104
## Sex                   1.2005     0.8330    0.7564     1.905
## Smoking               1.5179     0.6588    0.9147     2.519
## TG_total_log          0.9156     1.0921    0.6647     1.261
## UAER_log              1.2678     0.7888    1.1506     1.397
##
## Concordance= 0.847  (se = 0.032 )
## Rsquare= 0.232   (max possible= 0.828 )
## Likelihood ratio test= 154.8  on 12 df,   p=0
## Wald test            = 152.3  on 12 df,   p=0
## Score (logrank) test = 189.5  on 12 df,   p=0
```

4.2.1.1.1 Forest Plot with Clinical Variables

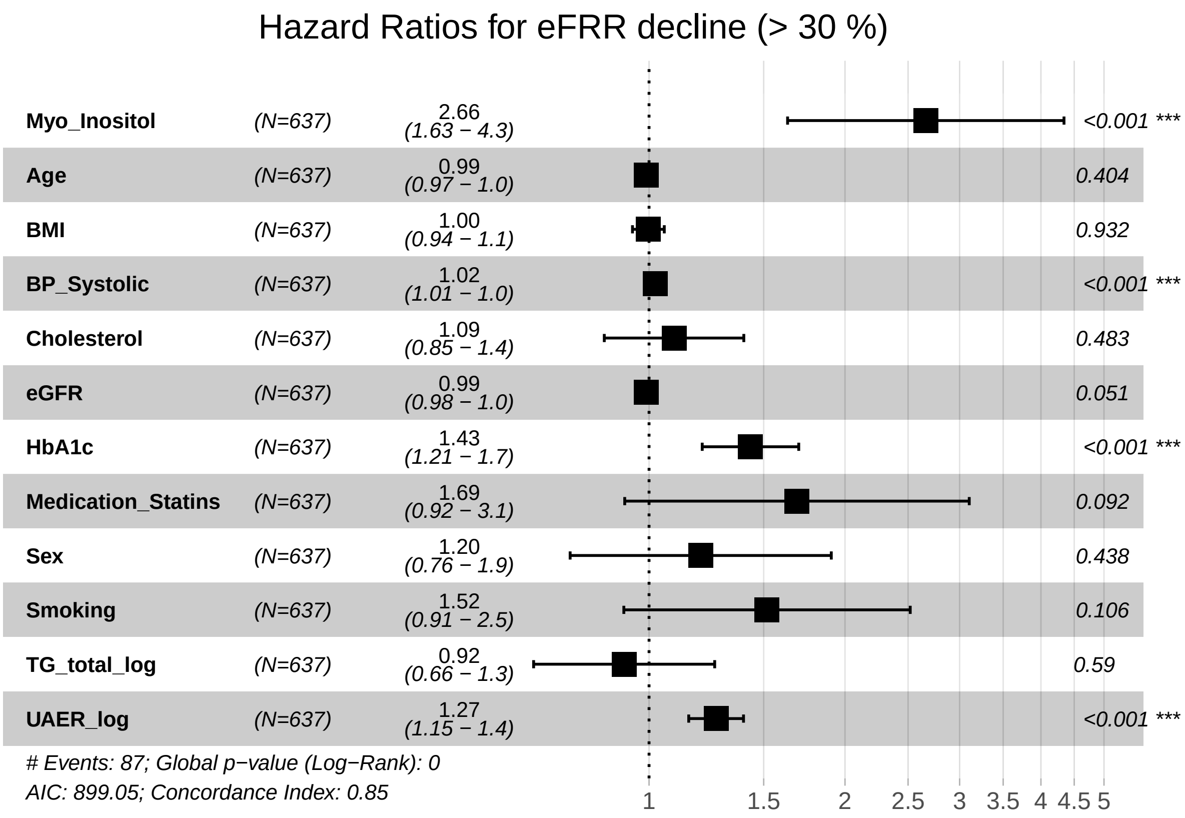
Hazard Ratios for eFRR decline (> 30 %)
Myo_Inositol (N=637) 2.66 (1.63 − 4.3) <0.001 ***
Age (N=637) 0.99 (0.97 − 1.0) 0.404
BMI (N=637) 1.00 (0.94 − 1.1) 0.932
BP_Systolic (N=637) 1.02 (1.01 − 1.0) <0.001 ***
Cholesterol (N=637) 1.09 (0.85 − 1.4) 0.483
eGFR (N=637) 0.99 (0.98 − 1.0) 0.051
HbA1c (N=637) 1.43 (1.21 − 1.7) <0.001 ***
Medication_Statins (N=637) 1.69 (0.92 − 3.1) 0.092
Sex (N=637) 1.20 (0.76 − 1.9) 0.438
Smoking (N=637) 1.52 (0.91 − 2.5) 0.106
TG_total_log (N=637) 0.92 (0.66 − 1.3) 0.59
UAER_log (N=637) 1.27 (1.15 − 1.4) <0.001 ***
Events: 87; Global p−value (Log−Rank): 0
AIC: 899.05; Concordance Index: 0.85
1 1.5 2 2.5 3 3.5 4 4.5 5

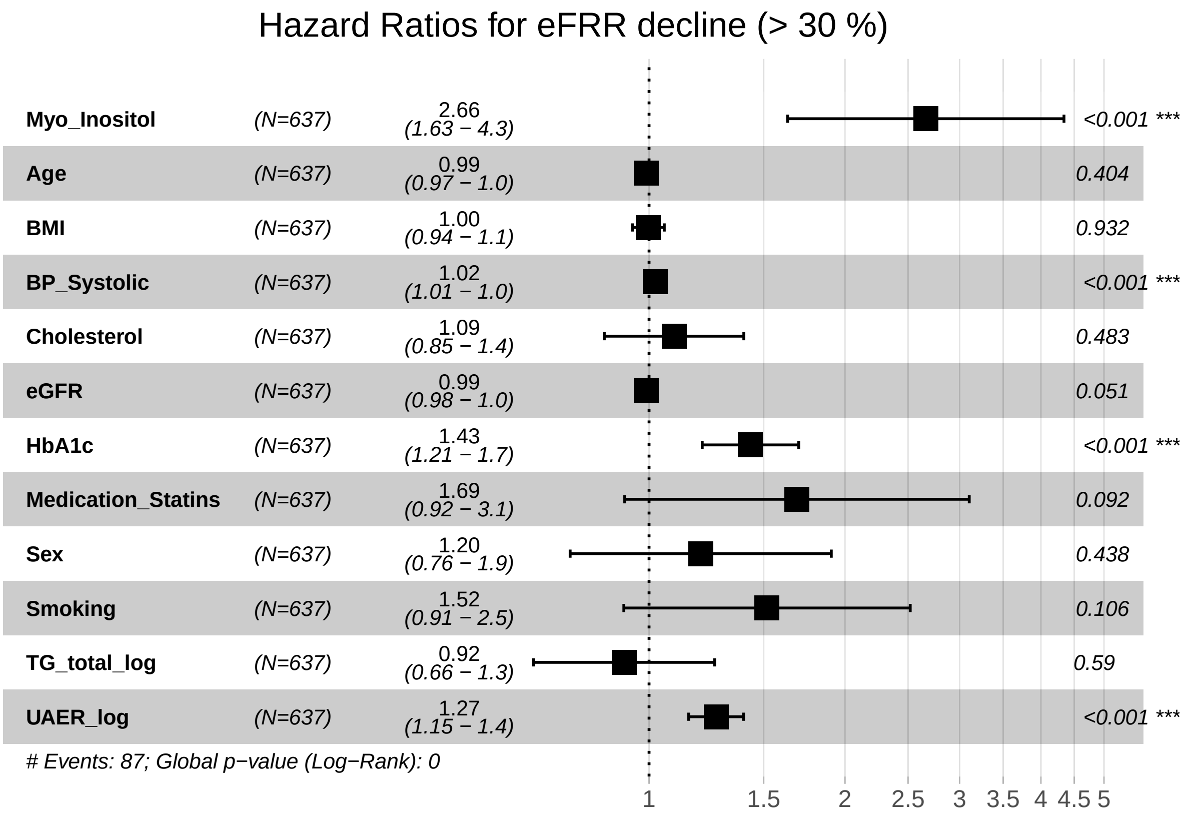
Hazard Ratios for eFRR decline (> 30 %)
Myo_Inositol (N=637) 2.66 (1.63 − 4.3) <0.001 ***
Age (N=637) 0.99 (0.97 − 1.0) 0.404
BMI (N=637) 1.00 (0.94 − 1.1) 0.932
BP_Systolic (N=637) 1.02 (1.01 − 1.0) <0.001 ***
Cholesterol (N=637) 1.09 (0.85 − 1.4) 0.483
eGFR (N=637) 0.99 (0.98 − 1.0) 0.051
HbA1c (N=637) 1.43 (1.21 − 1.7) <0.001 ***
Medication_Statins (N=637) 1.69 (0.92 − 3.1) 0.092
Sex (N=637) 1.20 (0.76 − 1.9) 0.438
Smoking (N=637) 1.52 (0.91 − 2.5) 0.106
TG_total_log (N=637) 0.92 (0.66 − 1.3) 0.59
UAER_log (N=637) 1.27 (1.15 − 1.4) <0.001 ***
Events: 87; Global p−value (Log−Rank): 0
1 1.5 2 2.5 3 3.5 4 4.5 5

4.2.1.1.2 Diagnostics of the Survival Model

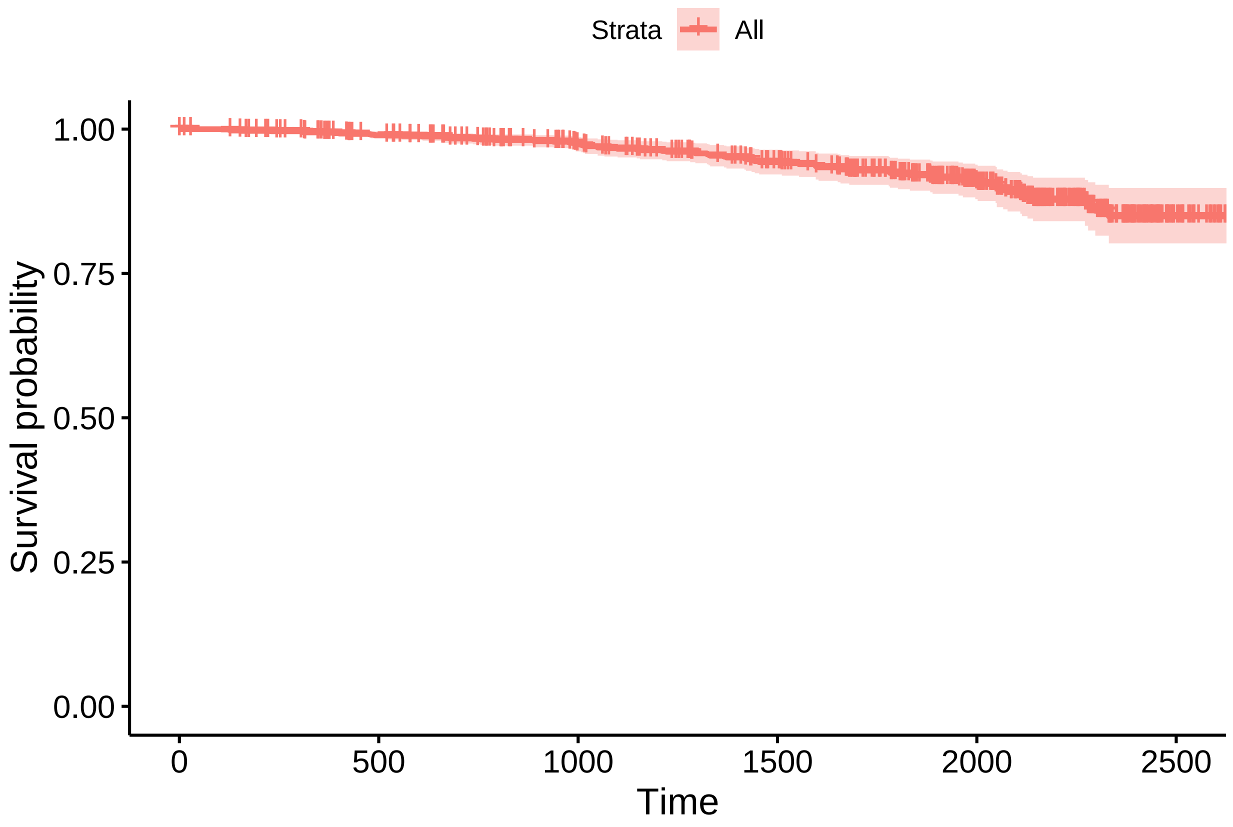

Strata
All
Survival probability
1.00
0.75
0.50
0.25
0.00
0
500
1000
1500
2000
2500
Time

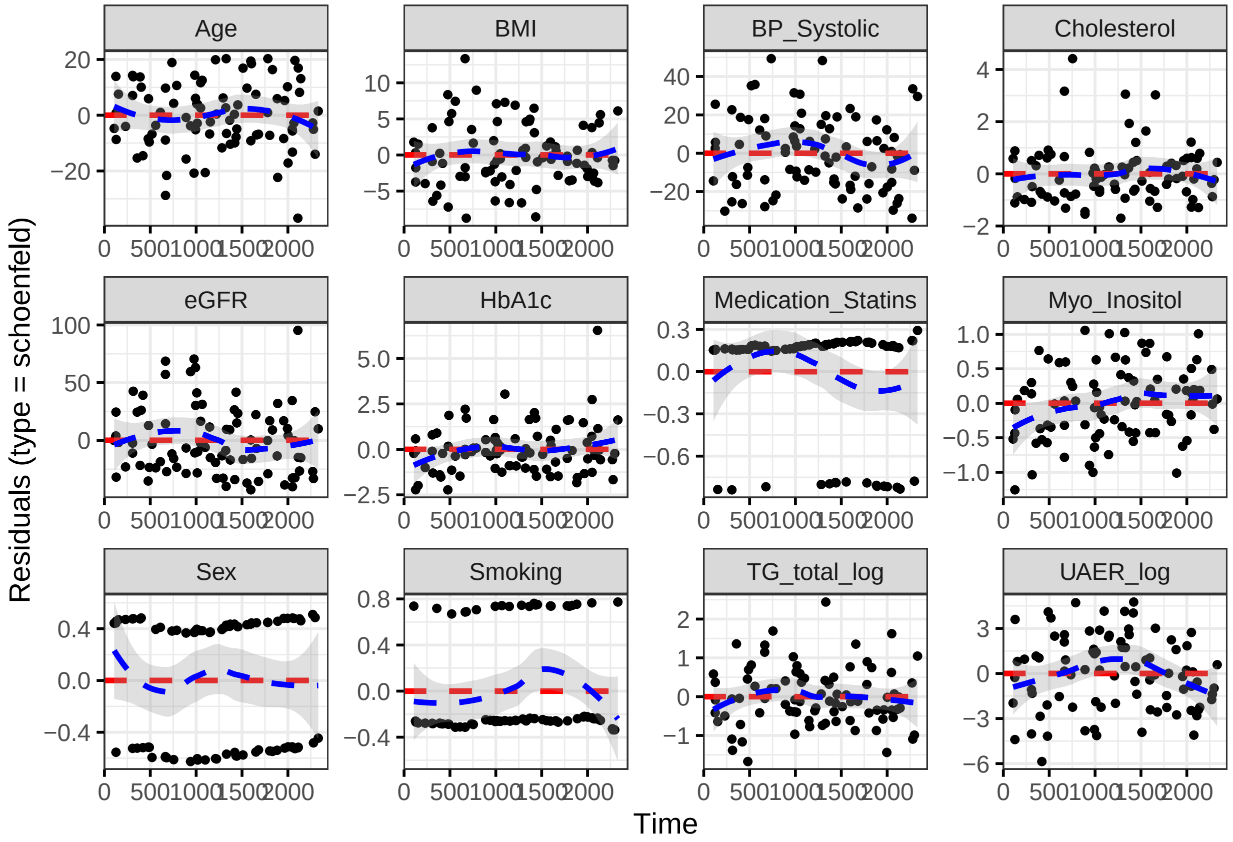

Residuals (type = schoenfeld)
Age
BMI
BP_Systolic
Cholesterol
eGFR
HbA1c
Medication_Statins
Myo_Inositol
Sex
Smoking
TG_total_log
UAER_log
Time

4.2.1.1.3 Kaplan-Maier Curve with Median Cutpoint

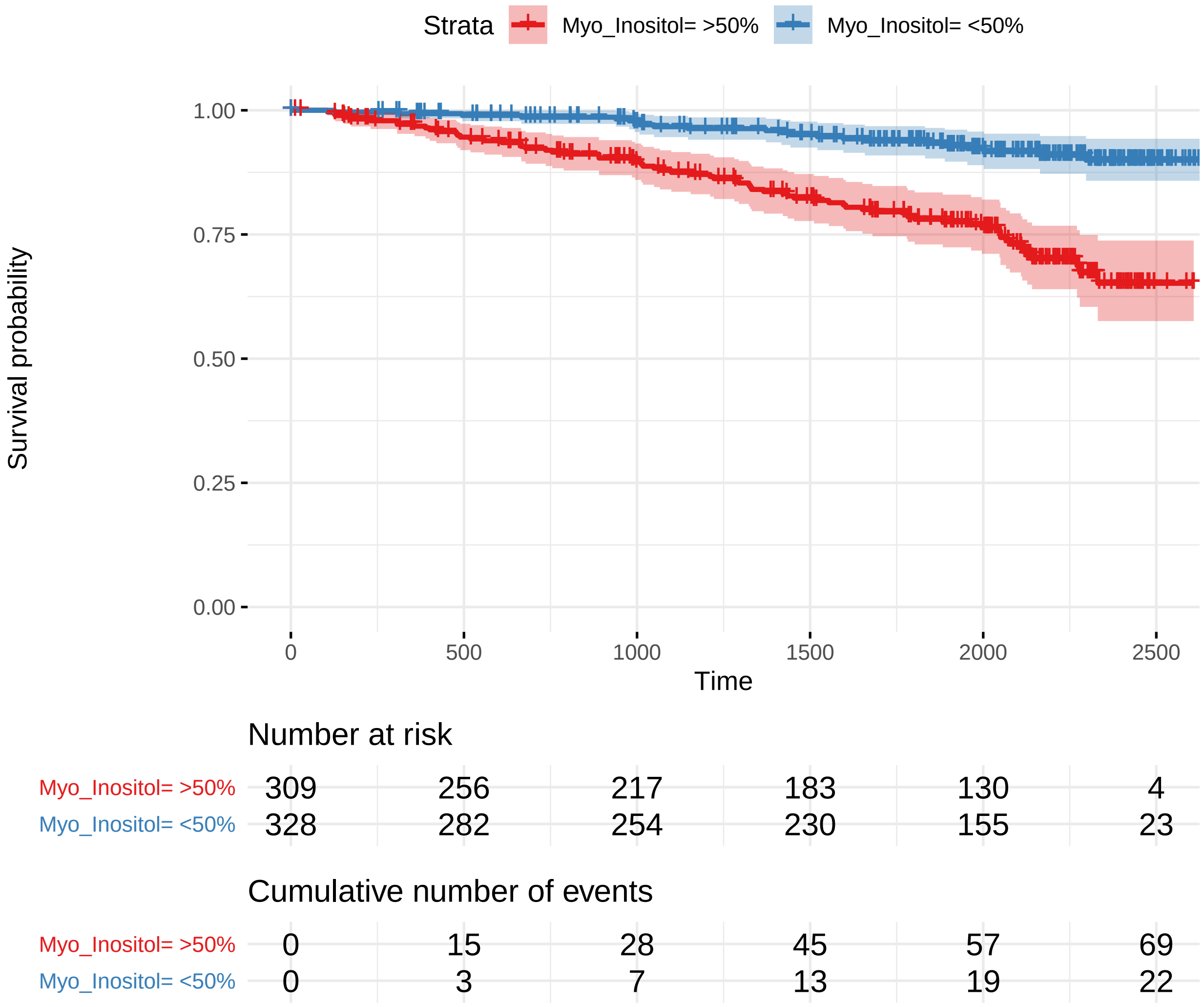

4.2.1.2 Other Model Fits

```
## Call:
## survival::coxph(formula = survival::Surv(time = t_gfrfald30_p,
##     event = censor_gfrfald30_p.reversed.numeric) ~ Myo_Inositol,
##     data = data.km)
##
##   n= 637, number of events= 91
##
##                       coef exp(coef) se(coef)      z Pr(>|z|)
## Myo_Inositol <50% -1.3443    0.2607   0.2454 -5.477 4.32e-08 ***
## ---
## Signif. codes:  0 '***' 0.001 '**' 0.01 '*' 0.05 '.' 0.1 ' ' 1
##
##                   exp(coef) exp(-coef) lower .95 upper .95
## Myo_Inositol <50%    0.2607      3.836    0.1612    0.4218
##
## Concordance= 0.653  (se = 0.027 )
## Rsquare= 0.054   (max possible= 0.82 )
## Likelihood ratio test= 35.66  on 1 df,   p=2.352e-09
## Wald test            = 30  on 1 df,   p=4.324e-08
## Score (logrank) test = 34.72  on 1 df,   p=3.811e-09
```

```
##
## Call:
## glm(formula = censor_gfrfald30_p.reversed.numeric ~ Myo_Inositol +
##     Age + BMI + BP_Systolic + Cholesterol + eGFR + HbA1c + Medication_Statins +
##     Sex + Smoking + TG_total_log + UAER_log, data = data.survival)
##
## Deviance Residuals:
##      Min        1Q    Median        3Q       Max
## -0.64186  -0.17645  -0.07273   0.03479   1.01620
##
## Coefficients:
##                      Estimate Std. Error t value Pr(>|t|)
## (Intercept)        -2.4903314  0.5778984  -4.309 1.93e-05 ***
## Myo_Inositol        0.1006377  0.0277956   3.621 0.000320 ***
## Age                -0.0015975  0.0012690  -1.259 0.208611
## BMI                -0.0003500  0.0035255  -0.099 0.920953
## BP_Systolic         0.0021111  0.0008320   2.538 0.011426 *
## Cholesterol         0.0111586  0.0165547   0.674 0.500557
## eGFR               -0.0009119  0.0006131  -1.487 0.137493
## HbA1c               0.0459983  0.0126206   3.645 0.000292 ***
## Medication_Statins  0.0289055  0.0312215   0.926 0.354929
## Sex                 0.0051173  0.0282786   0.181 0.856464
## Smoking             0.0091989  0.0336190   0.274 0.784474
## TG_total_log        0.0076032  0.0223770   0.340 0.734150
## UAER_log            0.0356352  0.0069800   5.105 4.50e-07 ***
## ---
## Signif. codes:  0 '***' 0.001 '**' 0.01 '*' 0.05 '.' 0.1 ' ' 1
##
## (Dispersion parameter for gaussian family taken to be 0.1027996)
##
##     Null deviance: 74.084  on 585  degrees of freedom
## Residual deviance: 58.904  on 573  degrees of freedom
```

```
##   (51 observations deleted due to missingness)
## AIC: 344.71
##
## Number of Fisher Scoring iterations: 2

##
## Call:
## lm(formula = Myo_Inositol ~ censor_gfrfald30_p.reversed.numeric +
##     Age + BMI + BP_Systolic + Cholesterol + eGFR + HbA1c + Medication_Statins +
##     Sex + Smoking + TG_total_log + UAER_log, data = data.survival)
##
## Residuals:
##      Min       1Q   Median       3Q      Max
## -2.01490 -0.28018 -0.00498  0.25886  1.88430
##
## Coefficients:
##                                       Estimate Std. Error t value Pr(>|t|)
## (Intercept)                         19.7966675  0.2783172  71.130  < 2e-16
## censor_gfrfald30_p.reversed.numeric  0.2222437  0.0613826   3.621  0.00032
## Age                                  0.0041051  0.0018806   2.183  0.02945
## BMI                                 -0.0048505  0.0052352  -0.927  0.35457
## BP_Systolic                         -0.0001693  0.0012432  -0.136  0.89175
## Cholesterol                         -0.0343832  0.0245690  -1.399  0.16222
## eGFR                                -0.0087509  0.0008365 -10.462  < 2e-16
## HbA1c                                0.0102942  0.0189661   0.543  0.58750
## Medication_Statins                  -0.0215989  0.0464228  -0.465  0.64192
## Sex                                 -0.0621546  0.0419444  -1.482  0.13893
## Smoking                             -0.0636181  0.0498921  -1.275  0.20279
## TG_total_log                         0.0478027  0.0331968   1.440  0.15042
## UAER_log                             0.0036953  0.0106048   0.348  0.72763
##
## (Intercept)                         ***
## censor_gfrfald30_p.reversed.numeric ***
## Age                                 *
## BMI
## BP_Systolic
## Cholesterol
## eGFR                                ***
## HbA1c
## Medication_Statins
## Sex
## Smoking
## TG_total_log
## UAER_log
## ---
## Signif. codes:  0 '***' 0.001 '**' 0.01 '*' 0.05 '.' 0.1 ' ' 1
##
## Residual standard error: 0.4765 on 573 degrees of freedom
##   (51 observations deleted due to missingness)
## Multiple R-squared:  0.2976, Adjusted R-squared:  0.2829
## F-statistic: 20.24 on 12 and 573 DF,  p-value: < 2.2e-16
```

4.2.2 Step 4.1B: Analysis of a Blood Pressure, HbA1c and logUAER-Matched Subcohort

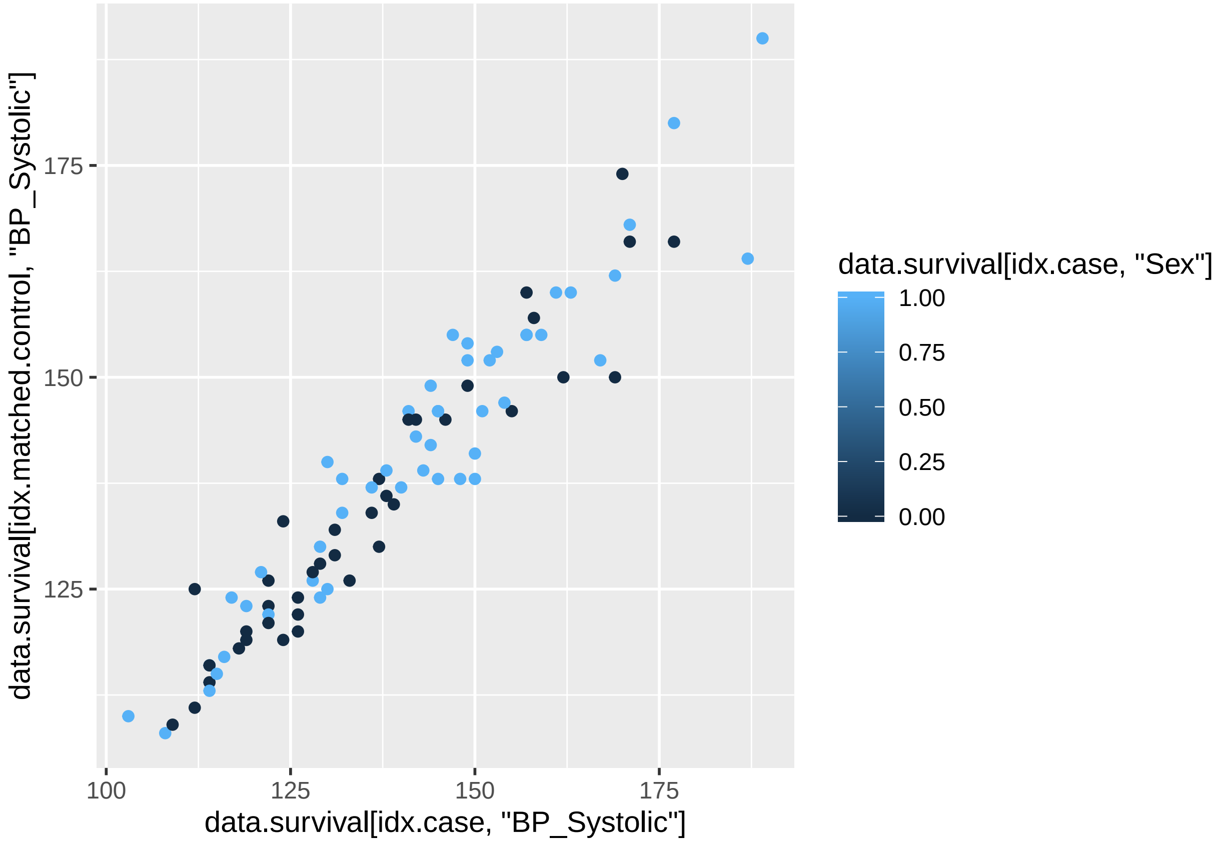

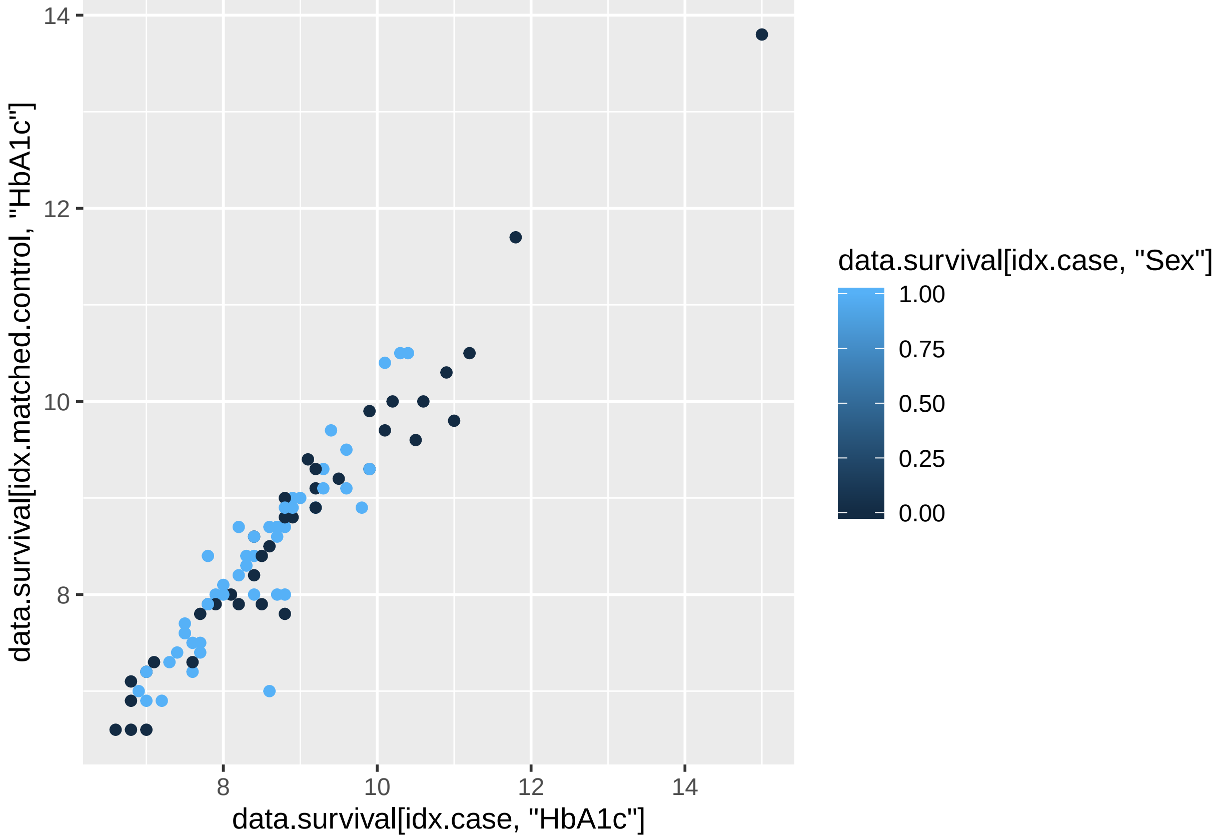

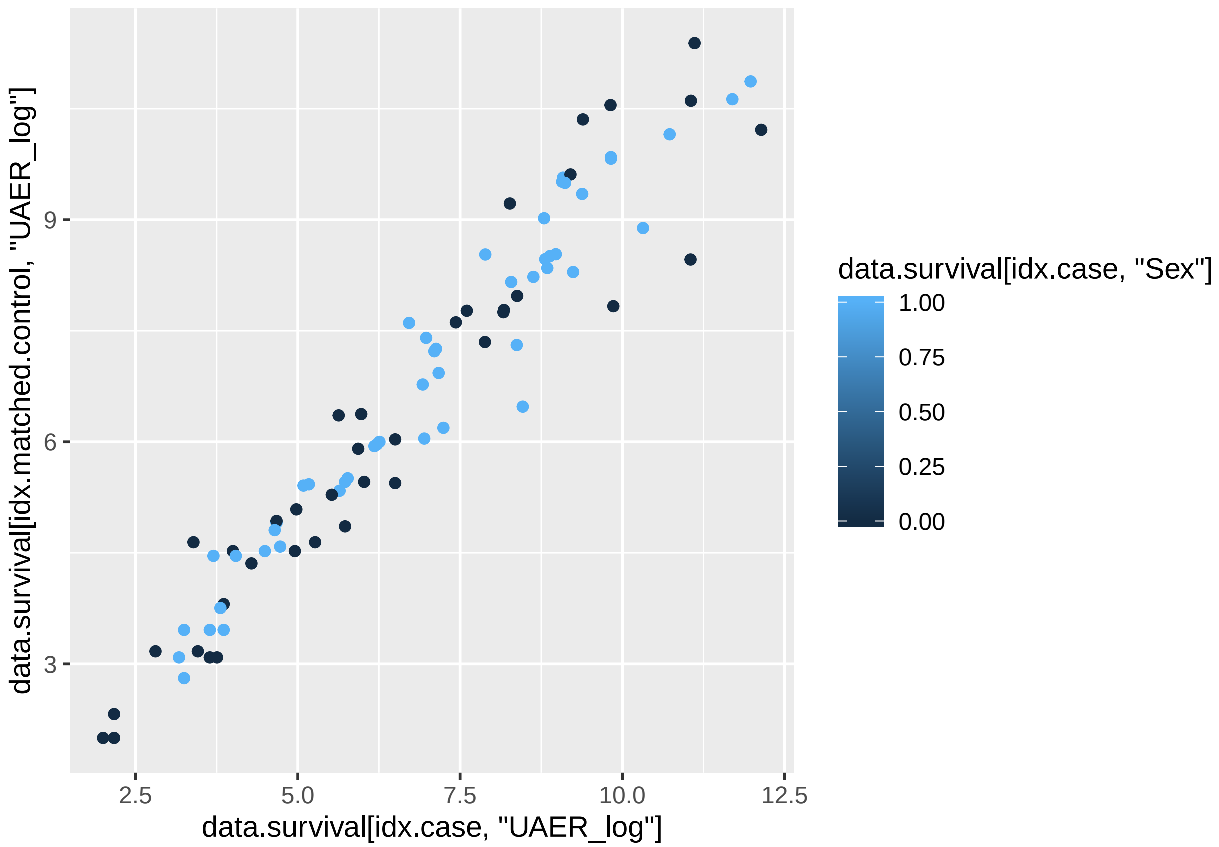


```
##
##  Paired t-test
##
## data:  data.survival[idx.case, "BP_Systolic"] and data.survival[idx.matched.control, "BP_Systolic"]
## t = 1.9697, df = 86, p-value = 0.05209
## alternative hypothesis: true difference in means is not equal to 0
## 95 percent confidence interval:
##  -0.01150946  2.49426809
## sample estimates:
## mean of the differences
##                1.241379
```

```
##
##  Paired t-test
##
## data:  data.survival[idx.case, "HbA1c"] and data.survival[idx.matched.control, "HbA1c"]
## t = 3.5465, df = 86, p-value = 0.0006347
## alternative hypothesis: true difference in means is not equal to 0
## 95 percent confidence interval:
##  0.06465628 0.22959659
## sample estimates:
## mean of the differences
##               0.1471264
```

```
##
##  Paired t-test
##
```

```
## data:  data.survival[idx.case, "UAER_log"] and data.survival[idx.matched.control, "UAER_log"]
## t = 2.6454, df = 86, p-value = 0.0097
## alternative hypothesis: true difference in means is not equal to 0
## 95 percent confidence interval:
##  0.04758177 0.33532528
## sample estimates:
## mean of the differences
##               0.1914535
```

```
##
## Call:
## glm(formula = censor_gfrfald30_p.reversed.numeric ~ Myo_Inositol +
##     Age + BMI + BP_Systolic + Cholesterol + eGFR + HbA1c + Medication_Statins +
##     Sex + Smoking + TG_total_log + UAER_log, data = data.survival.stratified)
##
## Deviance Residuals:
##     Min       1Q   Median       3Q      Max
## -0.8055  -0.4603   0.0940   0.4648   0.7881
##
## Coefficients:
##                      Estimate Std. Error t value Pr(>|t|)
## (Intercept)        -5.7226697  1.6058638  -3.564 0.000482 ***
## Myo_Inositol        0.2886231  0.0781982   3.691 0.000305 ***
## Age                -0.0022160  0.0037539  -0.590 0.555813
## BMI                 0.0055595  0.0099665   0.558 0.577746
## BP_Systolic         0.0012697  0.0022555   0.563 0.574266
## Cholesterol         0.0558232  0.0450728   1.239 0.217329
## eGFR                0.0007326  0.0016386   0.447 0.655412
## HbA1c               0.0132720  0.0328348   0.404 0.686598
## Medication_Statins  0.1293175  0.0954127   1.355 0.177205
## Sex                -0.0012794  0.0799892  -0.016 0.987258
## Smoking             0.0061972  0.0896848   0.069 0.944996
## TG_total_log       -0.0387143  0.0655796  -0.590 0.555790
## UAER_log           -0.0065286  0.0177393  -0.368 0.713337
## ---
## Signif. codes:  0 '***' 0.001 '**' 0.01 '*' 0.05 '.' 0.1 ' ' 1
##
## (Dispersion parameter for gaussian family taken to be 0.2372424)
##
##     Null deviance: 43.500  on 173  degrees of freedom
## Residual deviance: 38.196  on 161  degrees of freedom
## AIC: 257.95
##
## Number of Fisher Scoring iterations: 2
```

```
##
## Call:
## lm(formula = Myo_Inositol ~ censor_gfrfald30_p.reversed.numeric +
##     Age + BMI + BP_Systolic + Cholesterol + eGFR + HbA1c + Medication_Statins +
##     Sex + Smoking + TG_total_log + UAER_log, data = data.survival.stratified)
##
## Residuals:
##     Min      1Q  Median      3Q     Max
## -1.8629 -0.2757 -0.0401  0.2915  1.3326
##
```

```
## Coefficients:
##                                  Estimate Std. Error t value Pr(>|t|)
## (Intercept)                     19.363219   0.526059  36.808  < 2e-16
## censor_gfrfald30_p.reversed.numeric  0.270294   0.073232   3.691 0.000305
## Age                              0.005314   0.003612   1.471 0.143248
## BMI                              0.001369   0.009654   0.142 0.887393
## BP_Systolic                     -0.000999   0.002183  -0.458 0.647883
## Cholesterol                     -0.019438   0.043799  -0.444 0.657776
## eGFR                            -0.010530   0.001352  -7.787 7.92e-13
## HbA1c                            0.052056   0.031525   1.651 0.100640
## Medication_Statins              -0.061589   0.092732  -0.664 0.507533
## Sex                             -0.019574   0.077392  -0.253 0.800651
## Smoking                         -0.037295   0.086742  -0.430 0.667801
## TG_total_log                     0.036917   0.063465   0.582 0.561594
## UAER_log                        -0.002276   0.017173  -0.133 0.894743
##
## (Intercept)                     ***
## censor_gfrfald30_p.reversed.numeric ***
## Age
## BMI
## BP_Systolic
## Cholesterol
## eGFR                            ***
## HbA1c
## Medication_Statins
## Sex
## Smoking
## TG_total_log
## UAER_log
## ---
## Signif. codes:  0 '***' 0.001 '**' 0.01 '*' 0.05 '.' 0.1 ' ' 1
##
## Residual standard error: 0.4714 on 161 degrees of freedom
## Multiple R-squared:  0.4139, Adjusted R-squared:  0.3702
## F-statistic: 9.476 on 12 and 161 DF,  p-value: 9.388e-14
```

4.2.2.1 Survival Model with Details

```
## Call:
## survival::coxph(formula = survival::Surv(time = t_gfrfald30_p,
##     event = censor_gfrfald30_p.reversed.numeric) ~ Myo_Inositol +
##     Age + BMI + BP_Systolic + Cholesterol + eGFR + HbA1c + Medication_Statins +
##     Sex + Smoking + TG_total_log + UAER_log, data = data.survival.stratified)
##
##   n= 174, number of events= 87
##
##                         coef exp(coef)  se(coef)      z Pr(>|z|)
## Myo_Inositol        0.974990  2.651140  0.248029  3.931 8.46e-05 ***
## Age                -0.015145  0.984969  0.012083 -1.253    0.210
## BMI                 0.023558  1.023837  0.031015  0.760    0.448
## BP_Systolic         0.008866  1.008905  0.006846  1.295    0.195
## Cholesterol         0.192384  1.212136  0.135369  1.421    0.155
## eGFR               -0.004974  0.995038  0.005090 -0.977    0.328
## HbA1c               0.064293  1.066405  0.090056  0.714    0.475
```

```
## Medication_Statins  0.442379  1.556406  0.298951  1.480    0.139
## Sex                 0.051528  1.052879  0.236473  0.218    0.828
## Smoking             0.329798  1.390687  0.264734  1.246    0.213
## TG_total_log       -0.217046  0.804893  0.173527 -1.251    0.211
## UAER_log            0.073467  1.076233  0.050074  1.467    0.142
## ---
## Signif. codes:  0 '***' 0.001 '**' 0.01 '*' 0.05 '.' 0.1 ' ' 1
##
##                    exp(coef) exp(-coef) lower .95 upper .95
## Myo_Inositol          2.6511     0.3772    1.6305     4.311
## Age                   0.9850     1.0153    0.9619     1.009
## BMI                   1.0238     0.9767    0.9635     1.088
## BP_Systolic           1.0089     0.9912    0.9955     1.023
## Cholesterol           1.2121     0.8250    0.9297     1.580
## eGFR                  0.9950     1.0050    0.9852     1.005
## HbA1c                 1.0664     0.9377    0.8939     1.272
## Medication_Statins    1.5564     0.6425    0.8663     2.796
## Sex                   1.0529     0.9498    0.6624     1.674
## Smoking               1.3907     0.7191    0.8277     2.337
## TG_total_log          0.8049     1.2424    0.5728     1.131
## UAER_log              1.0762     0.9292    0.9756     1.187
##
## Concordance= 0.67  (se = 0.034 )
## Rsquare= 0.197   (max possible= 0.988 )
## Likelihood ratio test= 38.1  on 12 df,   p=0.0001481
## Wald test            = 35.21  on 12 df,   p=0.0004341
## Score (logrank) test = 36.58  on 12 df,   p=0.0002617
```

4.2.2.1.1 Forest Plot with Clinical Variables

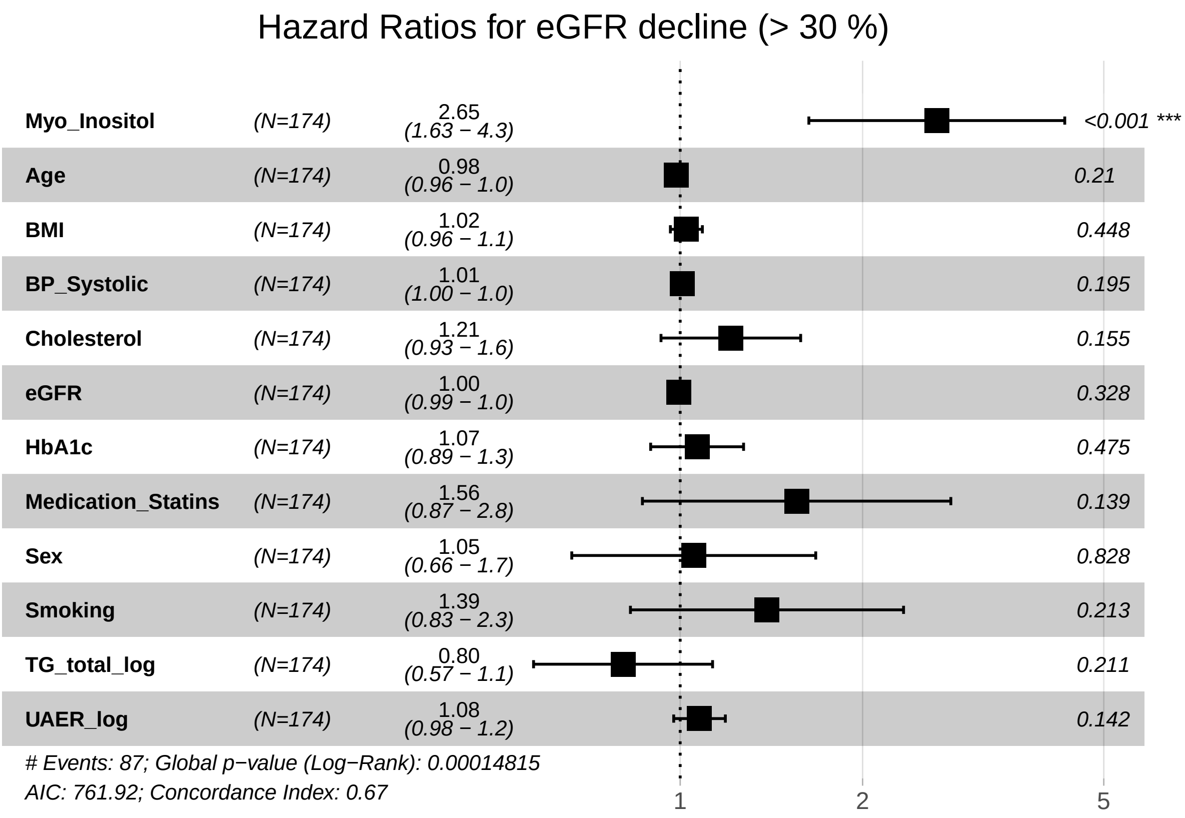
Hazard Ratios for eGFR decline (> 30 %)
Myo_Inositol (N=174) 2.65 (1.63 − 4.3) <0.001 ***
Age (N=174) 0.98 (0.96 − 1.0) 0.21
BMI (N=174) 1.02 (0.96 − 1.1) 0.448
BP_Systolic (N=174) 1.01 (1.00 − 1.0) 0.195
Cholesterol (N=174) 1.21 (0.93 − 1.6) 0.155
eGFR (N=174) 1.00 (0.99 − 1.0) 0.328
HbA1c (N=174) 1.07 (0.89 − 1.3) 0.475
Medication_Statins (N=174) 1.56 (0.87 − 2.8) 0.139
Sex (N=174) 1.05 (0.66 − 1.7) 0.828
Smoking (N=174) 1.39 (0.83 − 2.3) 0.213
TG_total_log (N=174) 0.80 (0.57 − 1.1) 0.211
UAER_log (N=174) 1.08 (0.98 − 1.2) 0.142
Events: 87; Global p−value (Log−Rank): 0.00014815
AIC: 761.92; Concordance Index: 0.67
1
2
5

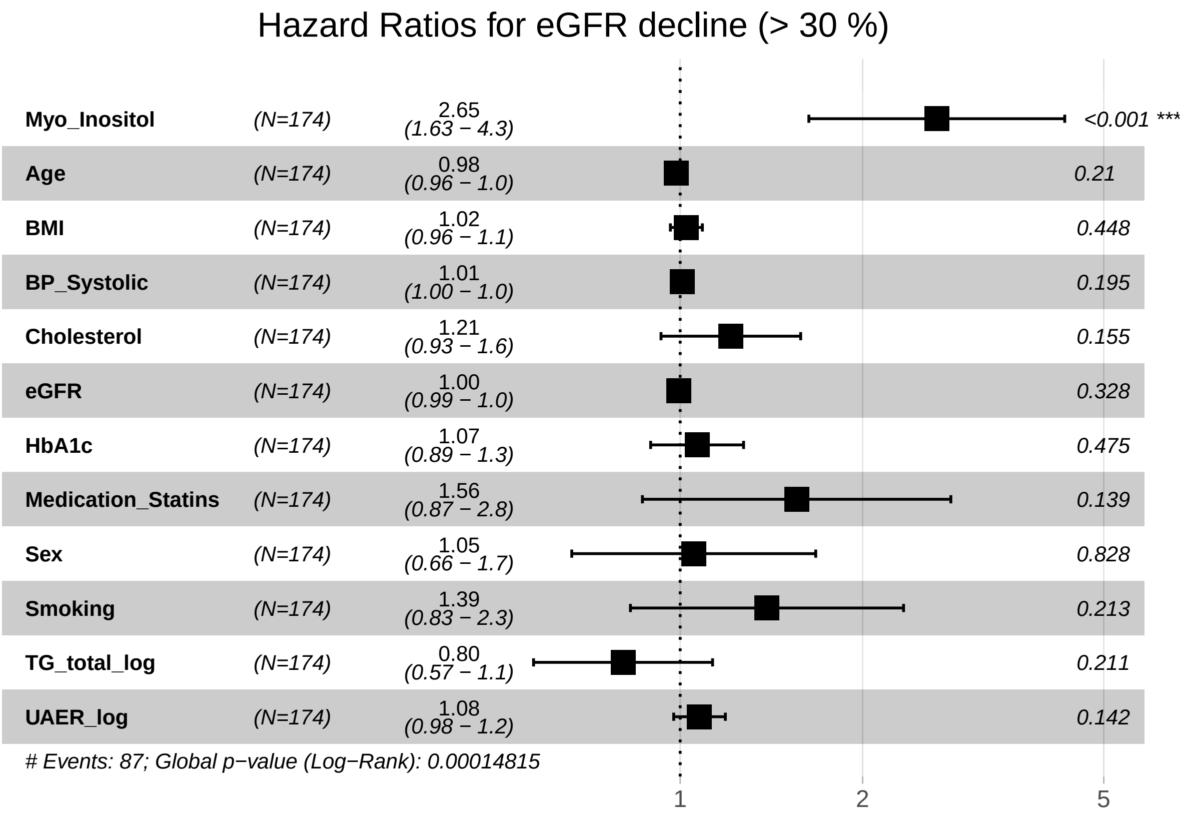
Hazard Ratios for eGFR decline (> 30 %)
Myo_Inositol (N=174) 2.65 (1.63 − 4.3) <0.001 ***
Age (N=174) 0.98 (0.96 − 1.0) 0.21
BMI (N=174) 1.02 (0.96 − 1.1) 0.448
BP_Systolic (N=174) 1.01 (1.00 − 1.0) 0.195
Cholesterol (N=174) 1.21 (0.93 − 1.6) 0.155
eGFR (N=174) 1.00 (0.99 − 1.0) 0.328
HbA1c (N=174) 1.07 (0.89 − 1.3) 0.475
Medication_Statins (N=174) 1.56 (0.87 − 2.8) 0.139
Sex (N=174) 1.05 (0.66 − 1.7) 0.828
Smoking (N=174) 1.39 (0.83 − 2.3) 0.213
TG_total_log (N=174) 0.80 (0.57 − 1.1) 0.211
UAER_log (N=174) 1.08 (0.98 − 1.2) 0.142
Events: 87; Global p−value (Log−Rank): 0.00014815
1
2
5

4.2.2.1.2 Diagnostics of the Survival Model

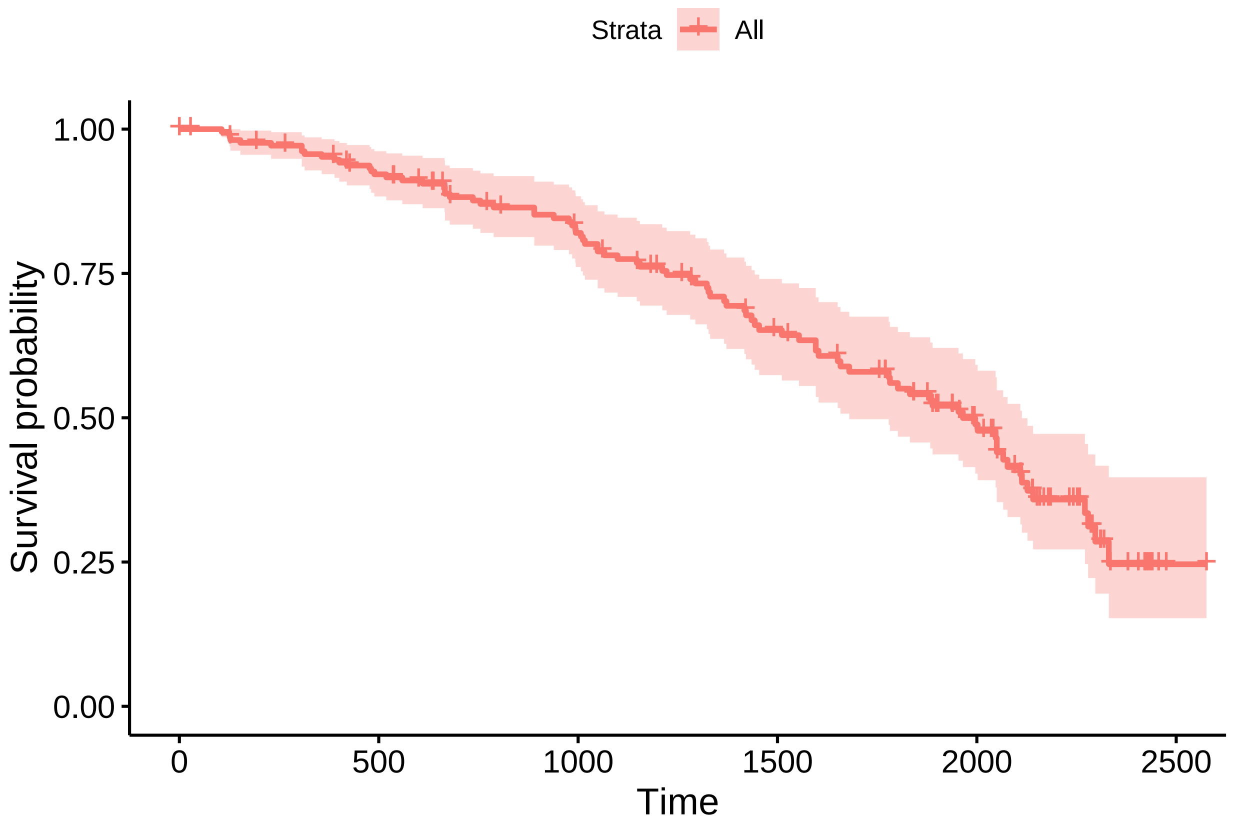
Strata
All
Survival probability
1.00
0.75
0.50
0.25
0.00
0
500
1000
1500
2000
2500
Time

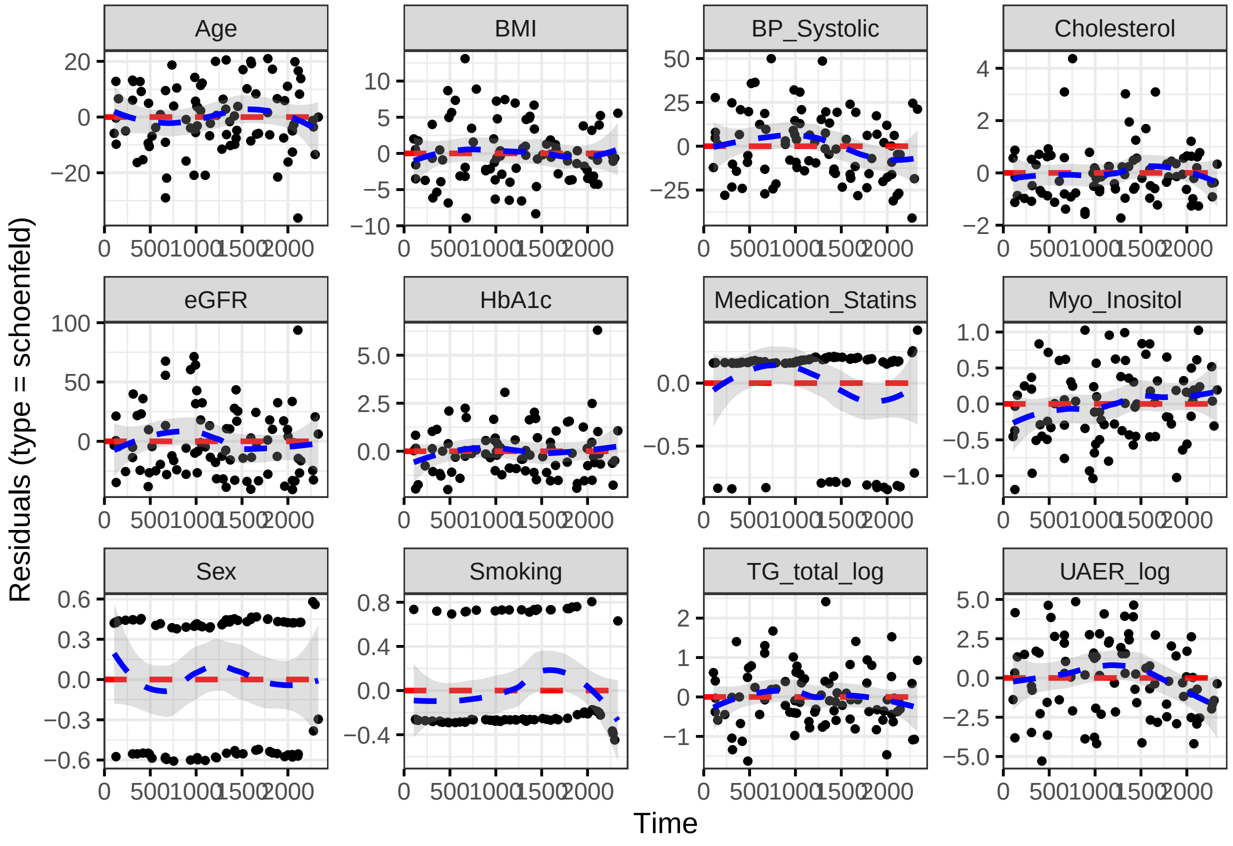
Age
BMI
BP_Systolic
Cholesterol
eGFR
HbA1c
Medication_Statins
Myo_Inositol
Sex
Smoking
TG_total_log
UAER_log
Residuals (type = schoenfeld)
Time

4.2.2.1.3 Kaplan-Maier Curve with Median Cutpoint

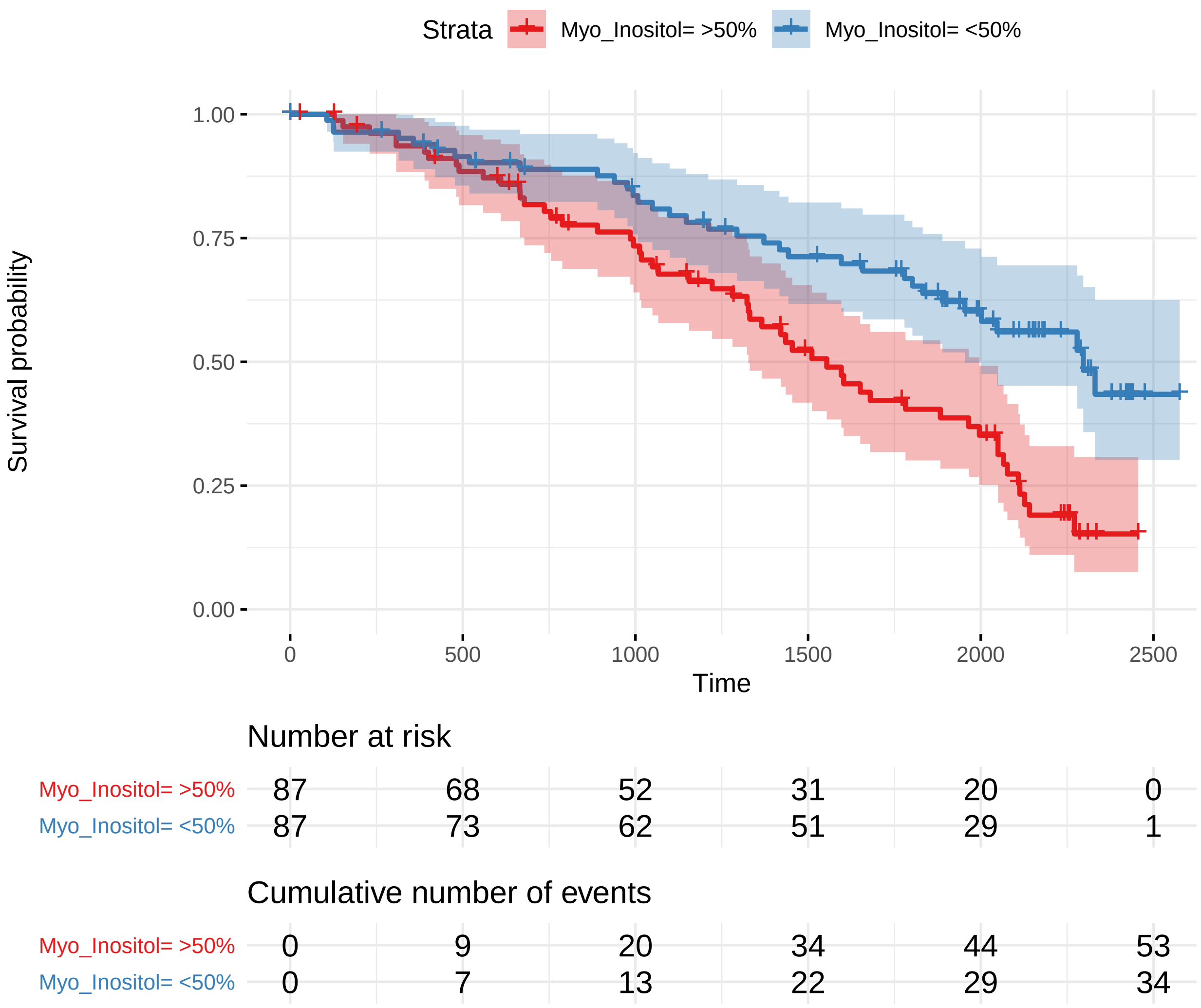

4.2.2.1.4 Boxplots

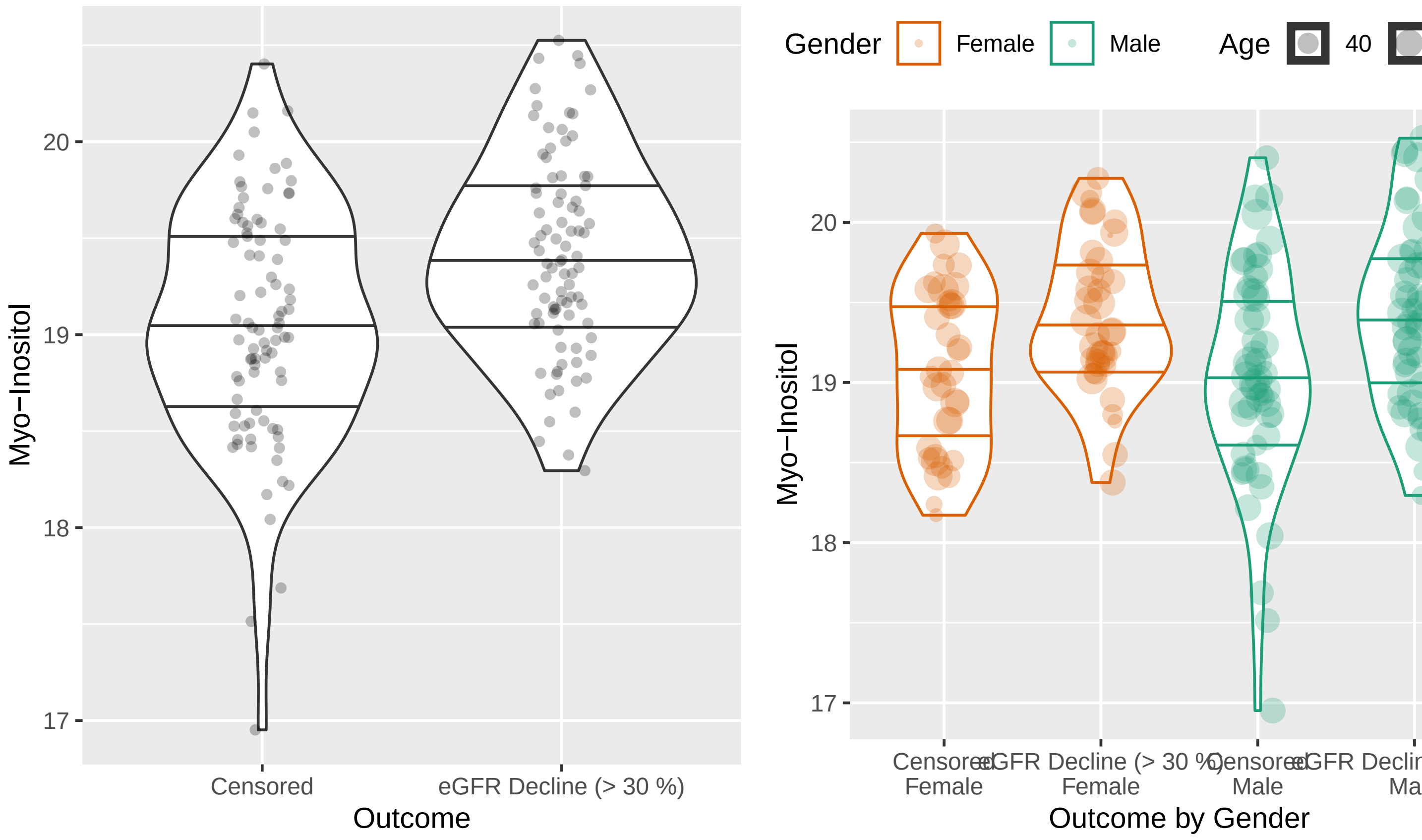

5 Sensitivity Analysis

5.1 Adjustment to Previous Cardiovascular Disease

5.1.1 Step 2B: Adjusted Model

5.1.1.1 Table

Table 9: Crude survival model for combined renal endpoint.

Name	exp(coef)	lower .95	upper .95	Pr(>|z|)	p.adj
Leucine, 2TMS; 19	0.605	0.475	0.771	4.67e-05	0.00140
Ribonic acid; 72	1.750	1.330	2.290	5.10e-05	0.00148
Isoleucine, 2TMS; 18	0.622	0.479	0.806	3.44e-04	0.00963
Valine, 2TMS; 20	0.437	0.275	0.692	4.30e-04	0.01160
Myo inositol 6TMS; 1	1.800	1.190	2.730	5.35e-03	0.13900
3,4-Dihydroxybutanoic acid; 27	1.600	1.070	2.370	2.08e-02	0.51900
Methionine, 2TMS; 16	0.730	0.556	0.958	2.33e-02	0.55900
4-Deoxytetronic acid; 33	0.801	0.651	0.985	3.56e-02	0.81800
2-Hydroxybutyric acid, 2TMS; 22	0.777	0.607	0.994	4.45e-02	0.98000
2,4-Dihydroxybutanoic acid; 28	1.410	0.971	2.040	7.14e-02	1.00000
Malic acid, 3TMS; 11	1.420	0.950	2.130	8.74e-02	1.00000
Palmitic acid, TMS; 5	1.910	0.863	4.210	1.10e-01	1.00000
Creatinine; 50	1.190	0.952	1.490	1.27e-01	1.00000
Fumaric acid, 2TMS; 9	1.480	0.877	2.480	1.43e-01	1.00000
Ribitol; 71	1.230	0.880	1.720	2.24e-01	1.00000
4-Hydroxyphenyllactic acid; 44	1.180	0.823	1.680	3.74e-01	1.00000
Hydroxyproline; 64	1.100	0.880	1.370	4.05e-01	1.00000
Glycine, 3TMS; 17	0.795	0.454	1.390	4.23e-01	1.00000
4-Hydroxybenzeneacetic acid; 42	1.070	0.896	1.270	4.68e-01	1.00000
Glyceryl-glycoside; 59	1.120	0.812	1.550	4.83e-01	1.00000
Stearic acid, TMS; 2	1.330	0.516	3.410	5.58e-01	1.00000
Succinic acid, 2TMS; 7	1.150	0.629	2.100	6.52e-01	1.00000
Glycerol; 57	1.090	0.729	1.620	6.88e-01	1.00000
Serine, 3TMS; 14	0.907	0.562	1.460	6.90e-01	1.00000
Eicosapentaenoic acid; 55	0.962	0.766	1.210	7.36e-01	1.00000
Citric acid, 4TMS; 6	0.934	0.613	1.420	7.48e-01	1.00000
Pyroglutamic acid; 69	0.962	0.747	1.240	7.62e-01	1.00000
4-Deoxytetronic acid; 32	1.040	0.778	1.400	7.79e-01	1.00000
3-Indoleacetic acid; 40	1.030	0.797	1.320	8.42e-01	1.00000
2-hydroxy Isovaleric acid; 38	1.000	0.855	1.180	9.72e-01	1.00000

5.1.1.2 Forest Plot

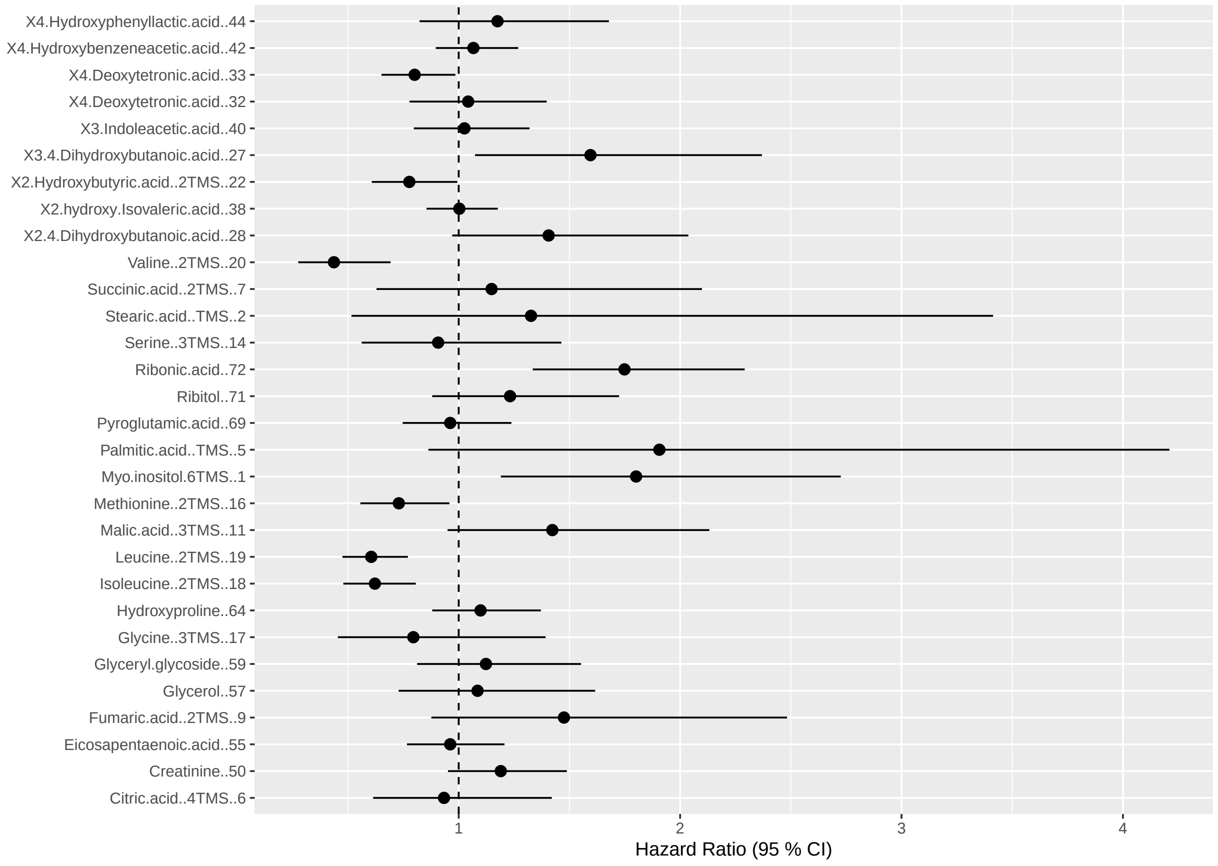

5.1.2 Step 3: Survival Analysis of Specific Renal Endpoints in Relation to Prioritized Metabolites from Step 2A

5.1.2.1 Step 3A: All-Cause Mortality

5.1.2.1.1 Table

Table 10: Adjusted survival model for all-cause mortality.

Name	exp(coef)	lower .95	upper .95	Pr(>|z|)	p.adj
X2.Hydroxybutyric.acid..2TMS..22	0.669	0.475	0.944	0.0222	0.311
Ribonic.acid..72	1.550	1.060	2.270	0.0245	0.318
Isoleucine..2TMS..18	0.710	0.497	1.010	0.0599	0.719
Ribitol..71	1.570	0.922	2.660	0.0969	1.000
Leucine..2TMS..19	0.757	0.522	1.100	0.1430	1.000
Fumaric.acid..2TMS..9	1.710	0.813	3.600	0.1570	1.000
Creatinine..50	1.360	0.887	2.070	0.1600	1.000
Myo.inositol.6TMS..1	1.550	0.830	2.910	0.1680	1.000
X2.4.Dihydroxybutanoic.acid..28	1.470	0.850	2.540	0.1690	1.000
Valine..2TMS..20	0.675	0.354	1.290	0.2330	1.000
Glyceryl.glycoside..59	1.360	0.819	2.260	0.2350	1.000
X3.4.Dihydroxybutanoic.acid..27	1.390	0.792	2.460	0.2490	1.000
X4.Hydroxybenzeneacetic.acid..42	1.100	0.859	1.410	0.4510	1.000
Methionine..2TMS..16	0.941	0.596	1.490	0.7950	1.000

5.1.2.1.2 Forest Plot

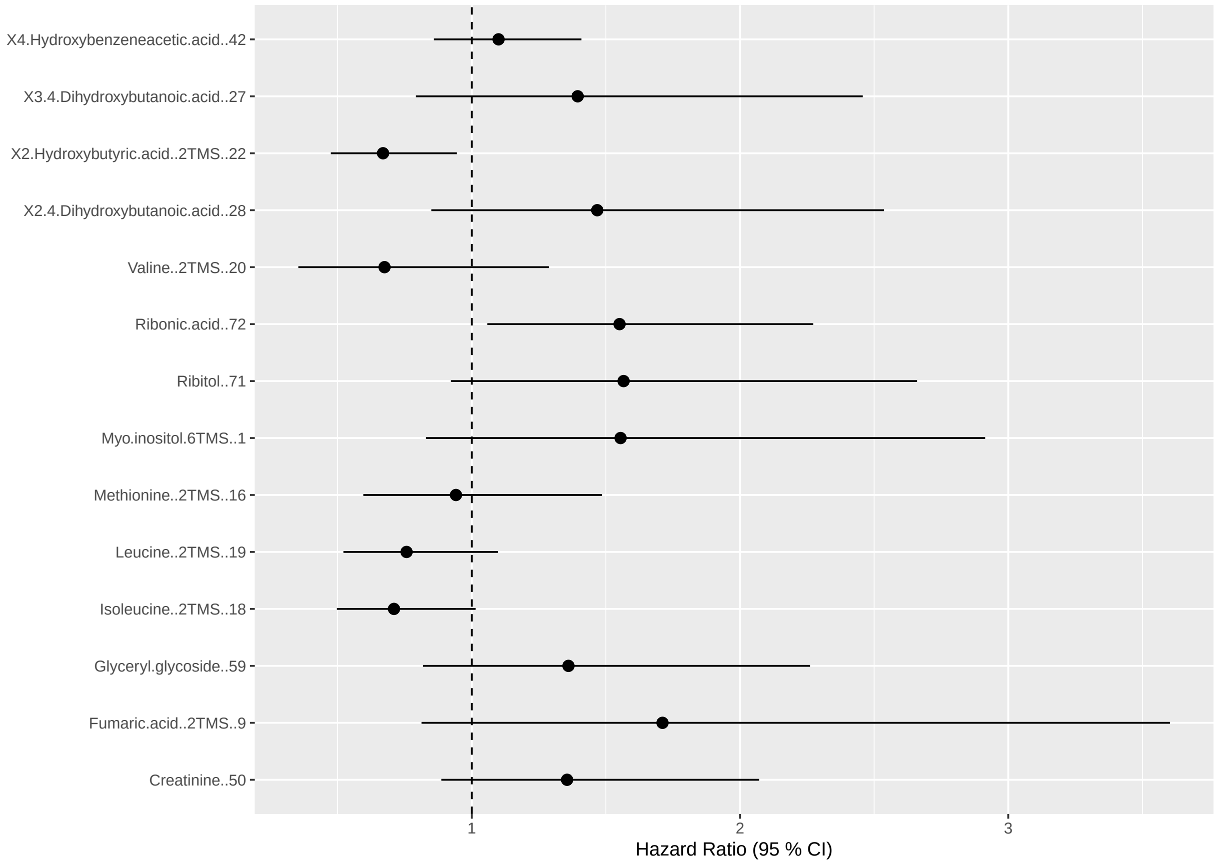

5.1.2.2 Step 3B: eGFR Decline > 30 %

5.1.2.2.1 Table

Table 11: Adjusted survival model for eGFR decline > 30 %.

Name	exp(coef)	lower .95	upper .95	Pr(>|z|)	p.adj
Ribonic.acid..72	2.220	1.640	2.990	2.00e-07	2.60e-06
Myo.inositol.6TMS..1	2.660	1.630	4.350	8.70e-05	1.13e-03
X3.4.Dihydroxybutanoic.acid..27	1.900	1.180	3.060	8.13e-03	9.75e-02
X2.4.Dihydroxybutanoic.acid..28	1.680	1.080	2.610	2.02e-02	2.23e-01
Isoleucine..2TMS..18	0.735	0.551	0.981	3.68e-02	3.68e-01
Valine..2TMS..20	0.552	0.312	0.975	4.05e-02	3.68e-01
X2.Hydroxybutyric.acid..2TMS..22	0.761	0.545	1.060	1.11e-01	8.86e-01
Methionine..2TMS..16	0.769	0.544	1.090	1.35e-01	9.47e-01
Ribitol..71	1.320	0.885	1.960	1.74e-01	1.00e+00
Creatinine..50	1.190	0.921	1.530	1.83e-01	1.00e+00
X4.Hydroxybenzeneacetic.acid..42	1.120	0.921	1.370	2.49e-01	1.00e+00
Leucine..2TMS..19	0.878	0.651	1.180	3.95e-01	1.00e+00
Glyceryl.glycoside..59	1.160	0.778	1.740	4.63e-01	1.00e+00
Fumaric.acid..2TMS..9	0.804	0.441	1.470	4.76e-01	1.00e+00

5.1.2.2.2 Forest Plot

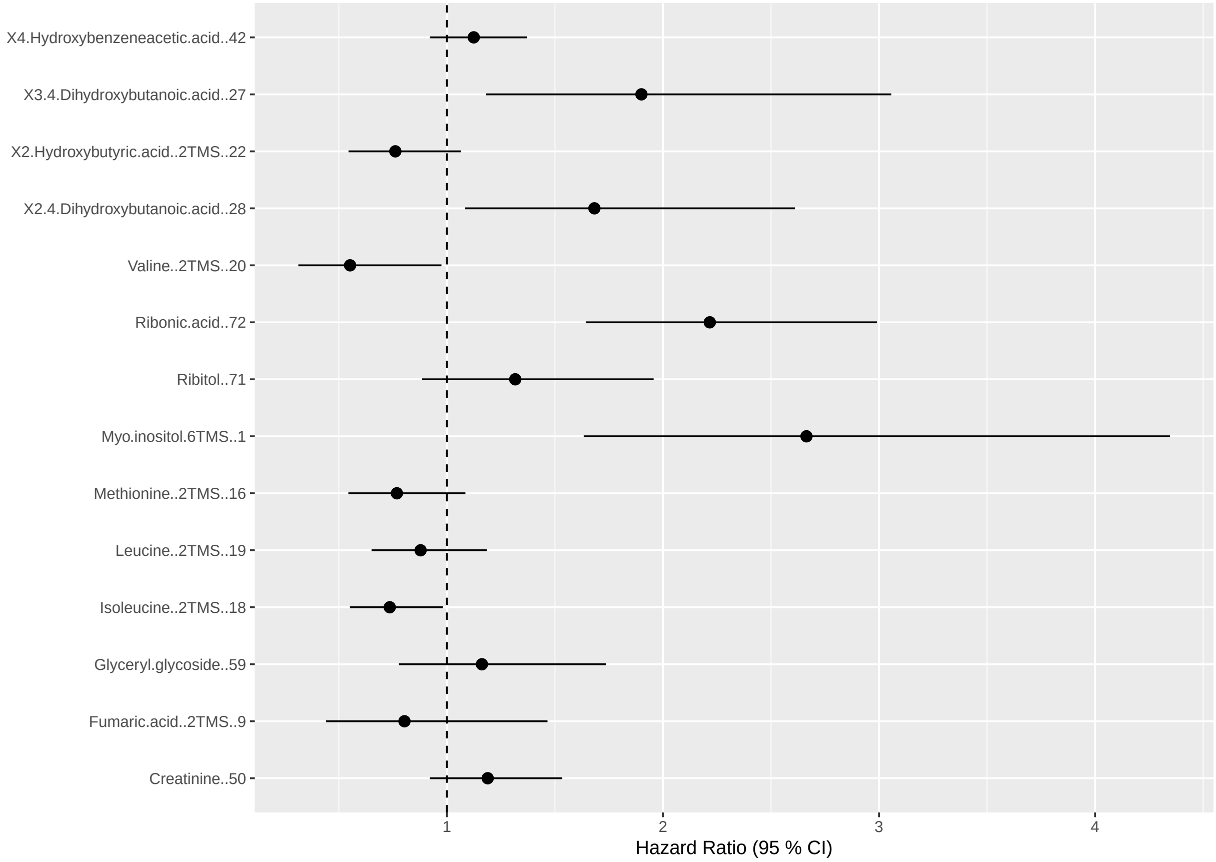

5.1.2.3 Step 3C: End-Stage Renal Disease

5.1.2.3.1 Adjusted Model

Table

Table 12: Adjusted survival model for end-stage renal disease.

Name	exp(coef)	lower .95	upper .95	Pr(>|z|)	p.adj
Ribitol..71	0.457	0.221	0.944	0.0345	0.483
Methionine..2TMS..16	0.606	0.319	1.150	0.1260	1.000
X4.Hydroxybenzeneacetic.acid..42	0.661	0.370	1.180	0.1620	1.000
Valine..2TMS..20	0.416	0.122	1.430	0.1630	1.000
X2.Hydroxybutyric.acid..2TMS..22	0.698	0.355	1.370	0.2970	1.000
Isoleucine..2TMS..18	0.778	0.467	1.300	0.3370	1.000
Fumaric.acid..2TMS..9	1.800	0.450	7.190	0.4070	1.000
Leucine..2TMS..19	0.841	0.546	1.290	0.4310	1.000
Glyceryl.glycoside..59	0.704	0.279	1.780	0.4580	1.000
Creatinine..50	0.891	0.554	1.440	0.6360	1.000
X3.4.Dihydroxybutanoic.acid..27	1.280	0.427	3.850	0.6570	1.000
Ribonic.acid..72	0.876	0.433	1.770	0.7120	1.000
Myo.inositol.6TMS..1	0.976	0.309	3.080	0.9670	1.000
X2.4.Dihydroxybutanoic.acid..28	1.010	0.323	3.170	0.9830	1.000

Forest Plot

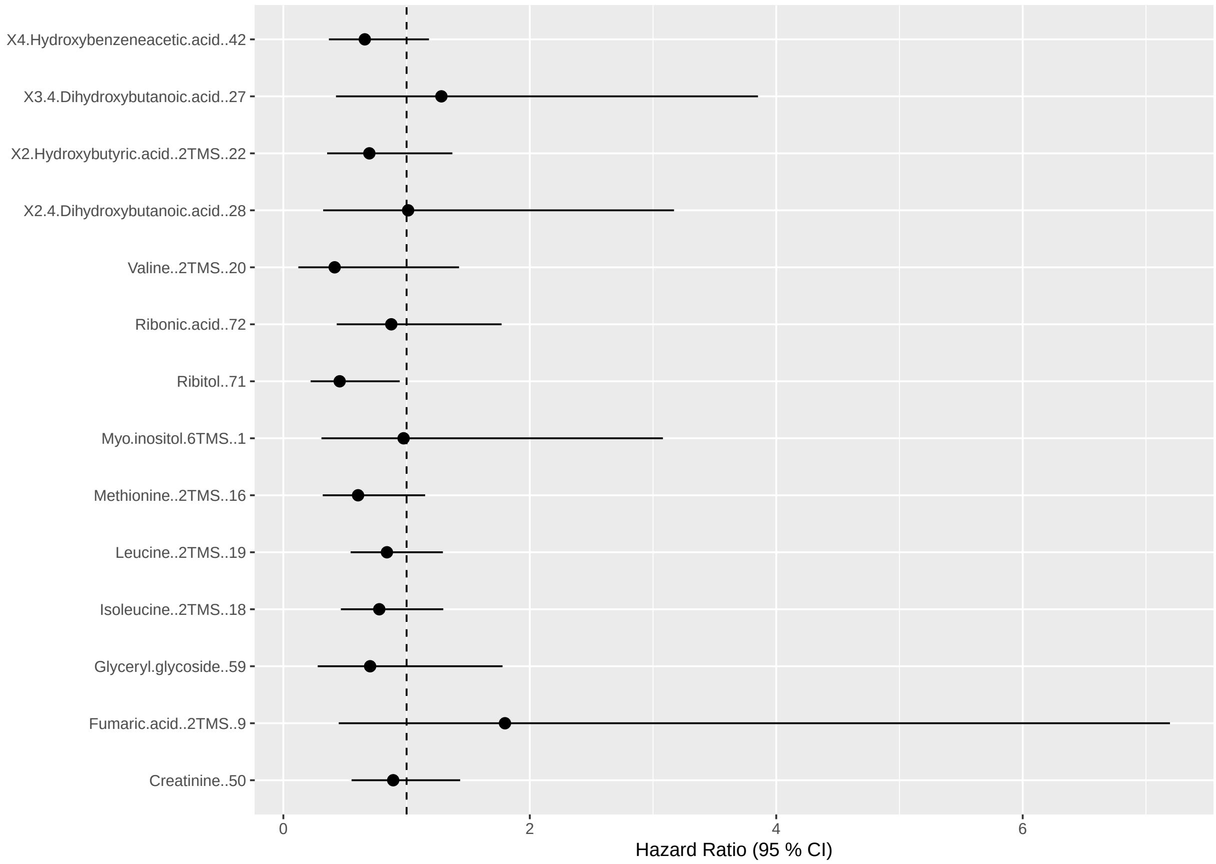
X4.Hydroxybenzeneacetic.acid..42
X3.4.Dihydroxybutanoic.acid..27
X2.Hydroxybutyric.acid..2TMS..22
X2.4.Dihydroxybutanoic.acid..28
Valine..2TMS..20
Ribonic.acid..72
Ribitol..71
Myo.inositol.6TMS..1
Methionine..2TMS..16
Leucine..2TMS..19
Isoleucine..2TMS..18
Glyceryl.glycoside..59
Fumaric.acid..2TMS..9
Creatinine..50
0
2
4
6
Hazard Ratio (95 % CI)

5.1.3 Compilation Forest Plot from Steps 3A-C

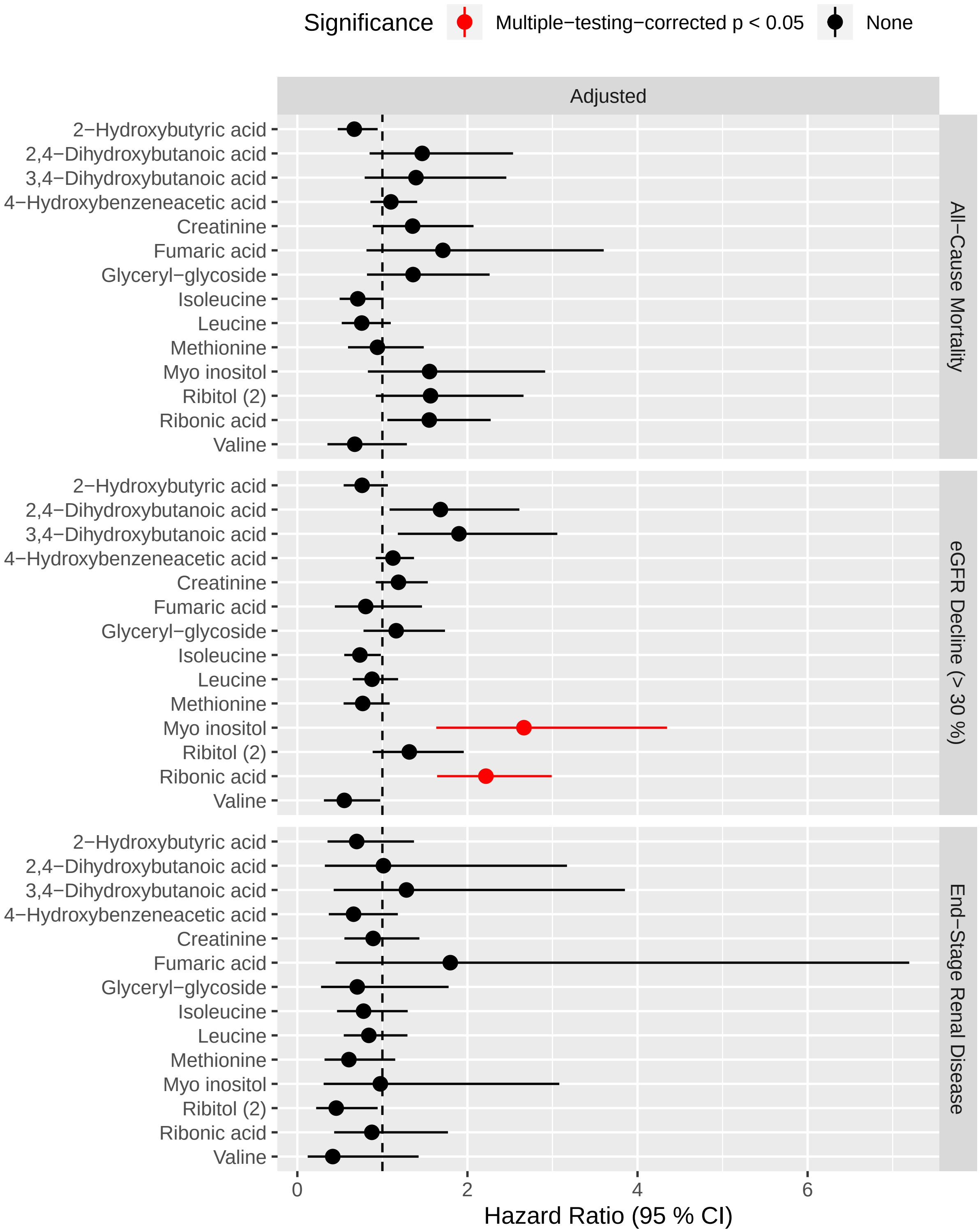

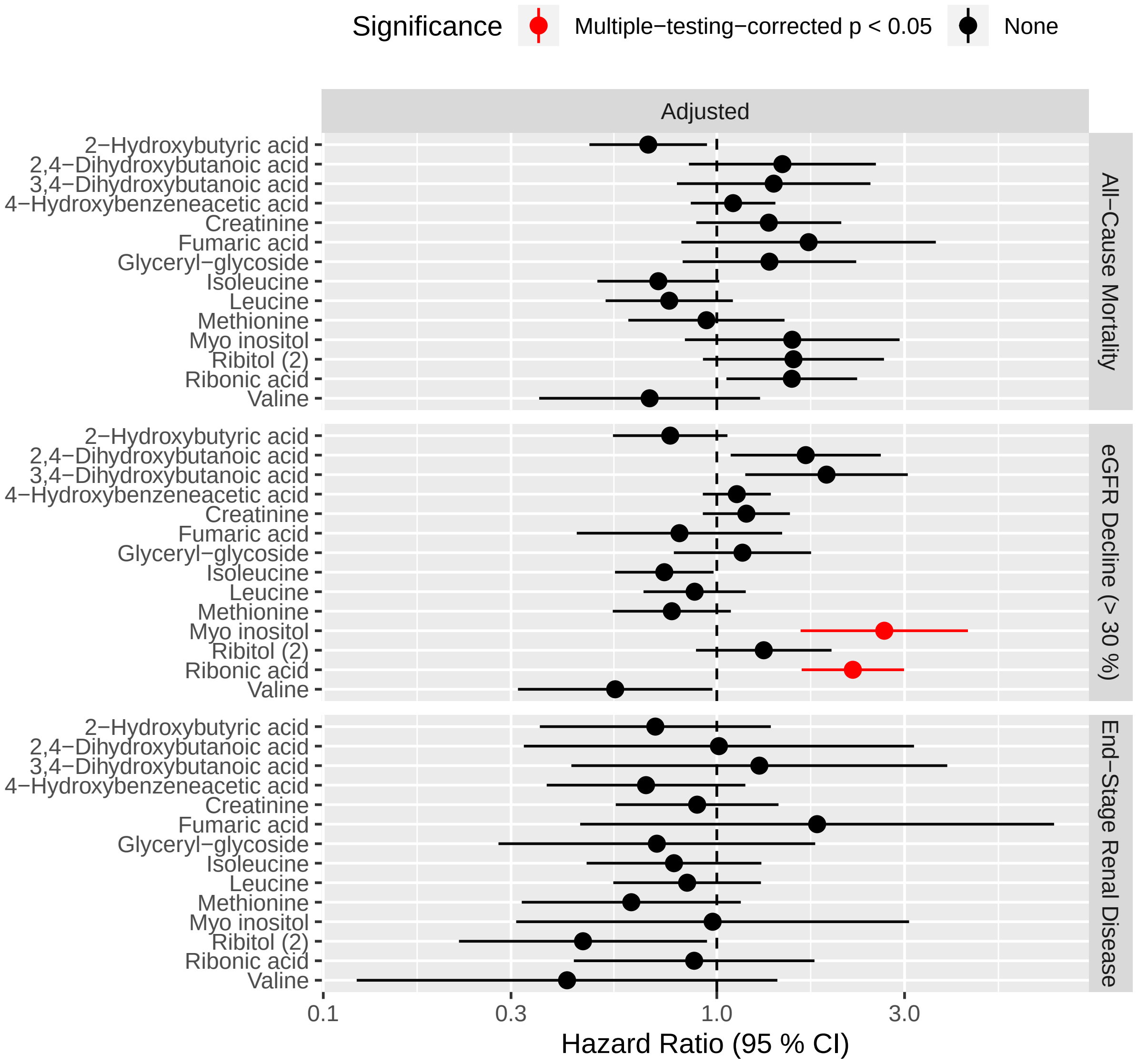
Significance
Multiple−testing−corrected p < 0.05
None
Adjusted
2−Hydroxybutyric acid
2,4−Dihydroxybutanoic acid
3,4−Dihydroxybutanoic acid
4−Hydroxybenzeneacetic acid
Creatinine
Fumaric acid
Glyceryl−glycoside
Isoleucine
Leucine
Methionine
Myo inositol
Ribitol (2)
Ribonic acid
Valine
All−Cause Mortality
eGFR Decline (> 30 %)
End−Stage Renal Disease
0.1
0.3
1.0
3.0
Hazard Ratio (95 % CI)

5.1.4 Step 4: Detailed Assessment of the Top-Metabolites in Relation to Outcomes

5.1.4.1 Ribonic Acid

```
## Call:
## survival::coxph(formula = survival::Surv(time = t_gfrfald30_p,
##     event = censor_gfrfald30_p.reversed.numeric) ~ Ribonic_acid +
##     Age + BMI + BP_Systolic + Cholesterol + eGFR + HbA1c + Medication_Statins +
##     Sex + Smoking + TG_total_log + UAER_log + Previous_CVD, data = data.survival)
##
##   n= 586, number of events= 87
##    (51 observations deleted due to missingness)
##
##                         coef exp(coef)  se(coef)      z Pr(>|z|)
## Ribonic_acid        0.796049  2.216765  0.152748  5.212 1.87e-07 ***
## Age                -0.013455  0.986636  0.012939 -1.040 0.298403
## BMI                -0.005670  0.994346  0.030023 -0.189 0.850213
## BP_Systolic         0.025769  1.026104  0.006725  3.832 0.000127 ***
## Cholesterol         0.166442  1.181095  0.132475  1.256 0.208969
## eGFR               -0.008965  0.991075  0.004994 -1.795 0.072634 .
## HbA1c               0.422584  1.525900  0.084905  4.977 6.45e-07 ***
## Medication_Statins  0.637401  1.891559  0.315796  2.018 0.043550 *
## Sex                 0.164956  1.179342  0.243917  0.676 0.498863
## Smoking             0.520189  1.682345  0.259799  2.002 0.045255 *
## TG_total_log       -0.131764  0.876548  0.156648 -0.841 0.400267
## UAER_log            0.251041  1.285362  0.049516  5.070 3.98e-07 ***
## Previous_CVD       -0.030432  0.970027  0.248030 -0.123 0.902349
## ---
## Signif. codes:  0 '***' 0.001 '**' 0.01 '*' 0.05 '.' 0.1 ' ' 1
##
##                    exp(coef) exp(-coef) lower .95 upper .95
## Ribonic_acid          2.2168     0.4511    1.6432     2.990
## Age                   0.9866     1.0135    0.9619     1.012
## BMI                   0.9943     1.0057    0.9375     1.055
## BP_Systolic           1.0261     0.9746    1.0127     1.040
## Cholesterol           1.1811     0.8467    0.9110     1.531
## eGFR                  0.9911     1.0090    0.9814     1.001
## HbA1c                 1.5259     0.6554    1.2920     1.802
## Medication_Statins    1.8916     0.5287    1.0186     3.513
## Sex                   1.1793     0.8479    0.7312     1.902
## Smoking               1.6823     0.5944    1.0111     2.799
## TG_total_log          0.8765     1.1408    0.6448     1.192
## UAER_log              1.2854     0.7780    1.1665     1.416
## Previous_CVD          0.9700     1.0309    0.5966     1.577
##
## Concordance= 0.856  (se = 0.032 )
## Rsquare= 0.25   (max possible= 0.828 )
## Likelihood ratio test= 168.2  on 13 df,   p=0
## Wald test            = 149  on 13 df,   p=0
## Score (logrank) test = 193.3  on 13 df,   p=0
```

5.1.4.1.1 Forest Plot with Clinical Variables

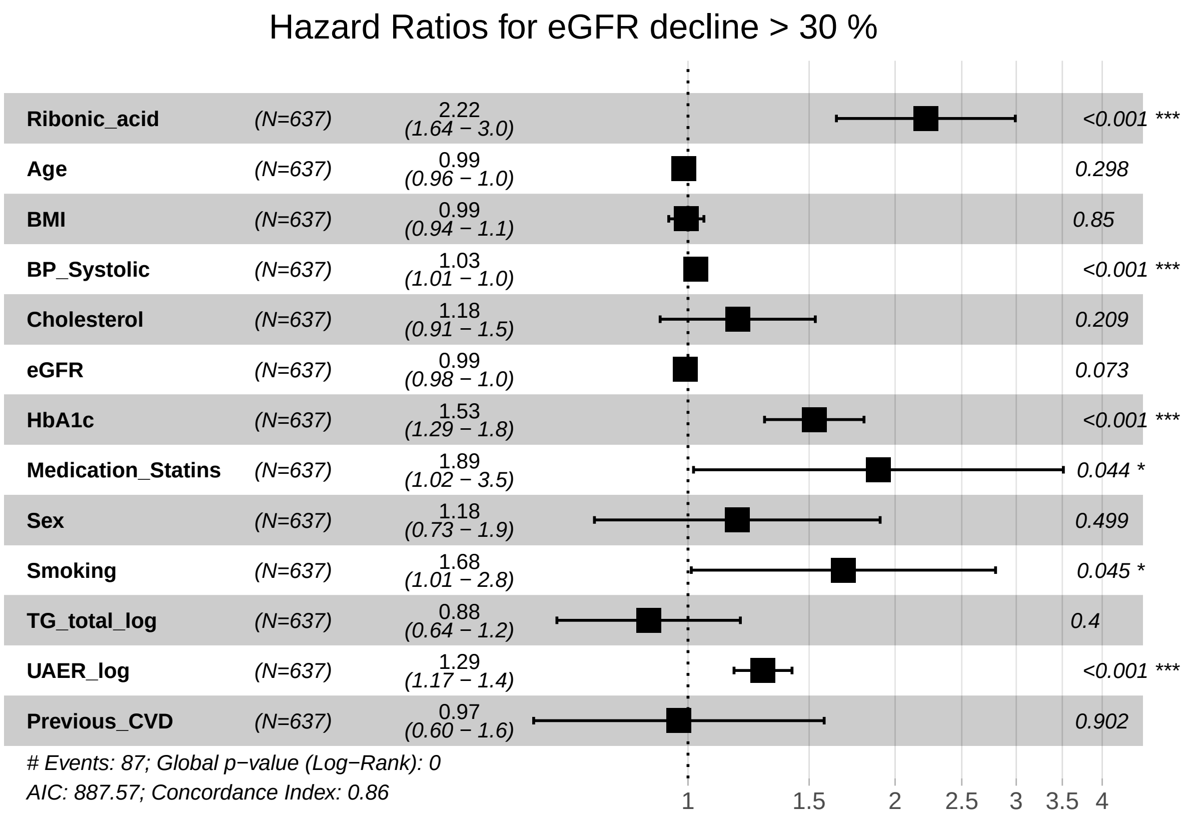
Hazard Ratios for eGFR decline > 30 %
Ribonic_acid (N=637) 2.22 (1.64 − 3.0) <0.001 ***
Age (N=637) 0.99 (0.96 − 1.0) 0.298
BMI (N=637) 0.99 (0.94 − 1.1) 0.85
BP_Systolic (N=637) 1.03 (1.01 − 1.0) <0.001 ***
Cholesterol (N=637) 1.18 (0.91 − 1.5) 0.209
eGFR (N=637) 0.99 (0.98 − 1.0) 0.073
HbA1c (N=637) 1.53 (1.29 − 1.8) <0.001 ***
Medication_Statins (N=637) 1.89 (1.02 − 3.5) 0.044 *
Sex (N=637) 1.18 (0.73 − 1.9) 0.499
Smoking (N=637) 1.68 (1.01 − 2.8) 0.045 *
TG_total_log (N=637) 0.88 (0.64 − 1.2) 0.4
UAER_log (N=637) 1.29 (1.17 − 1.4) <0.001 ***
Previous_CVD (N=637) 0.97 (0.60 − 1.6) 0.902
Events: 87; Global p−value (Log−Rank): 0
AIC: 887.57; Concordance Index: 0.86
1 1.5 2 2.5 3 3.5 4

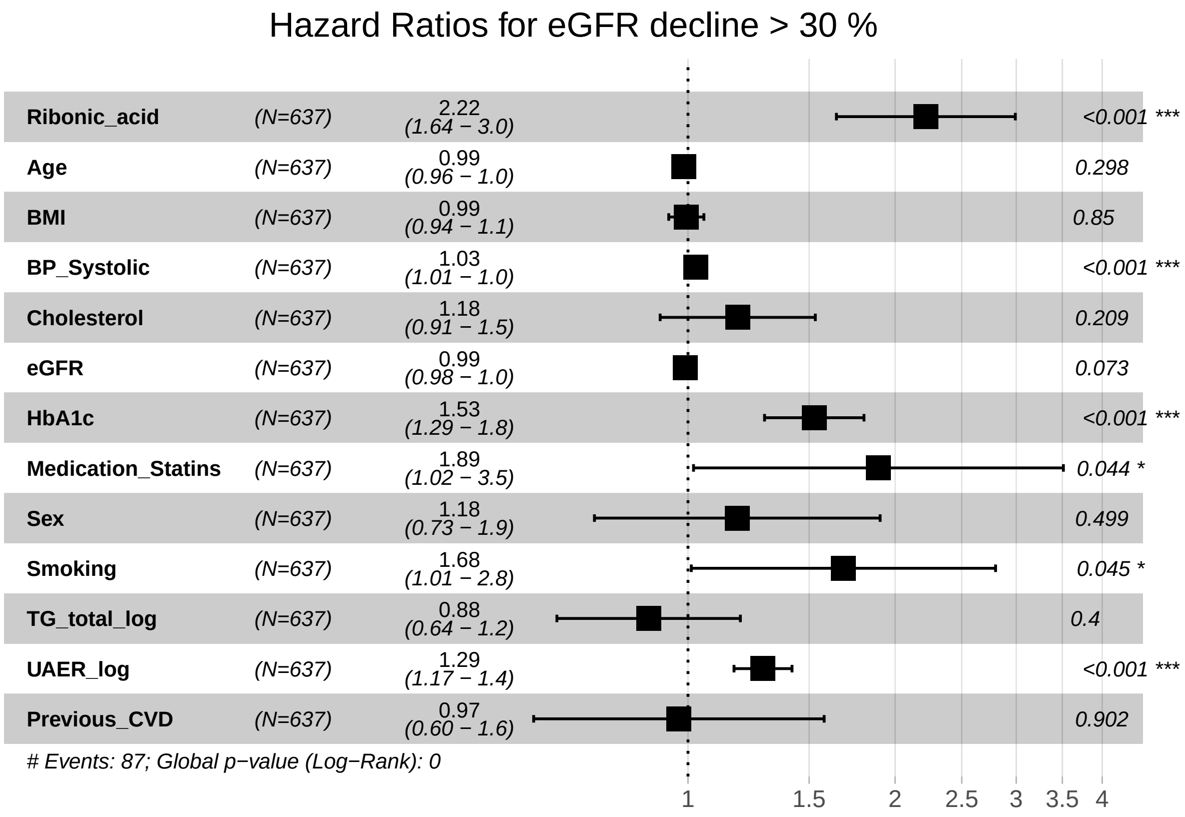
Hazard Ratios for eGFR decline > 30 %
Ribonic_acid (N=637) 2.22 (1.64 − 3.0) <0.001 ***
Age (N=637) 0.99 (0.96 − 1.0) 0.298
BMI (N=637) 0.99 (0.94 − 1.1) 0.85
BP_Systolic (N=637) 1.03 (1.01 − 1.0) <0.001 ***
Cholesterol (N=637) 1.18 (0.91 − 1.5) 0.209
eGFR (N=637) 0.99 (0.98 − 1.0) 0.073
HbA1c (N=637) 1.53 (1.29 − 1.8) <0.001 ***
Medication_Statins (N=637) 1.89 (1.02 − 3.5) 0.044 *
Sex (N=637) 1.18 (0.73 − 1.9) 0.499
Smoking (N=637) 1.68 (1.01 − 2.8) 0.045 *
TG_total_log (N=637) 0.88 (0.64 − 1.2) 0.4
UAER_log (N=637) 1.29 (1.17 − 1.4) <0.001 ***
Previous_CVD (N=637) 0.97 (0.60 − 1.6) 0.902
Events: 87; Global p−value (Log−Rank): 0
1 1.5 2 2.5 3 3.5 4

5.1.4.2 Myo-Inositol

```
## Call:
## survival::coxph(formula = survival::Surv(time = t_gfrfald30_p,
##     event = censor_gfrfald30_p.reversed.numeric) ~ Myo_Inositol +
##     Age + BMI + BP_Systolic + Cholesterol + eGFR + HbA1c + Medication_Statins +
##     Sex + Smoking + TG_total_log + UAER_log + Previous_CVD, data = data.survival)
##
##   n= 586, number of events= 87
##    (51 observations deleted due to missingness)
##
##                         coef exp(coef)  se(coef)      z Pr(>|z|)
## Myo_Inositol        0.979924  2.664253  0.249716  3.924 8.70e-05 ***
## Age                -0.010192  0.989859  0.012700 -0.803 0.422249
## BMI                -0.003143  0.996862  0.029513 -0.106 0.915196
## BP_Systolic         0.022611  1.022868  0.006736  3.357 0.000789 ***
## Cholesterol         0.088591  1.092634  0.125923  0.704 0.481724
## eGFR               -0.010352  0.989701  0.005319 -1.946 0.051632 .
## HbA1c               0.359808  1.433054  0.087661  4.105 4.05e-05 ***
## Medication_Statins  0.529355  1.697836  0.316443  1.673 0.094361 .
## Sex                 0.185536  1.203863  0.237420  0.781 0.434528
## Smoking             0.419864  1.521755  0.259588  1.617 0.105786
## TG_total_log       -0.086854  0.916810  0.163787 -0.530 0.595912
## UAER_log            0.237217  1.267716  0.049501  4.792 1.65e-06 ***
## Previous_CVD       -0.025960  0.974374  0.252518 -0.103 0.918119
## ---
## Signif. codes:  0 '***' 0.001 '**' 0.01 '*' 0.05 '.' 0.1 ' ' 1
##
##                    exp(coef) exp(-coef) lower .95 upper .95
## Myo_Inositol          2.6643     0.3753    1.6331     4.346
## Age                   0.9899     1.0102    0.9655     1.015
## BMI                   0.9969     1.0031    0.9408     1.056
## BP_Systolic           1.0229     0.9776    1.0095     1.036
## Cholesterol           1.0926     0.9152    0.8537     1.398
## eGFR                  0.9897     1.0104    0.9794     1.000
## HbA1c                 1.4331     0.6978    1.2068     1.702
## Medication_Statins    1.6978     0.5890    0.9131     3.157
## Sex                   1.2039     0.8307    0.7559     1.917
## Smoking               1.5218     0.6571    0.9149     2.531
## TG_total_log          0.9168     1.0907    0.6651     1.264
## UAER_log              1.2677     0.7888    1.1505     1.397
## Previous_CVD          0.9744     1.0263    0.5940     1.598
##
## Concordance= 0.847  (se = 0.032 )
## Rsquare= 0.232   (max possible= 0.828 )
## Likelihood ratio test= 154.8  on 13 df,   p=0
## Wald test            = 152.5  on 13 df,   p=0
## Score (logrank) test = 189.9  on 13 df,   p=0
```

5.1.4.2.1 Forest Plot with Clinical Variables

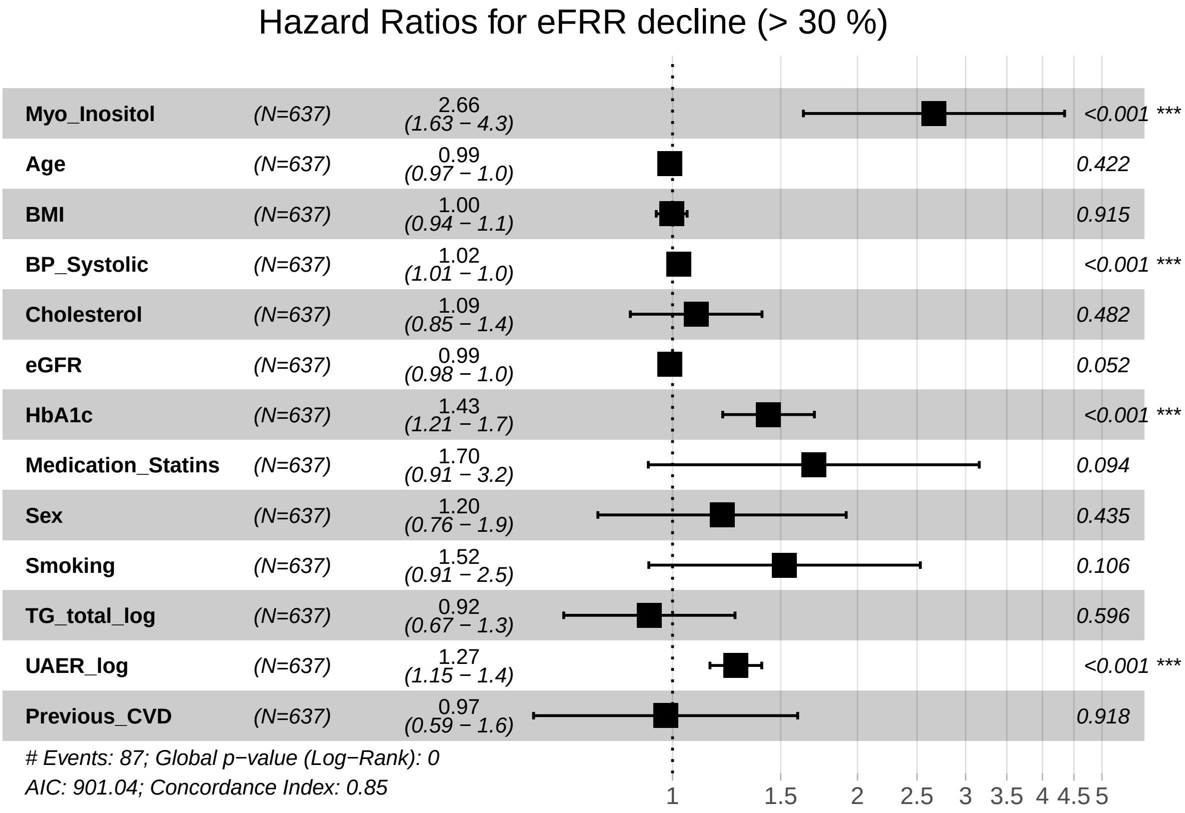
Hazard Ratios for eFRR decline (> 30 %)
Myo_Inositol (N=637) 2.66 (1.63 − 4.3) <0.001 ***
Age (N=637) 0.99 (0.97 − 1.0) 0.422
BMI (N=637) 1.00 (0.94 − 1.1) 0.915
BP_Systolic (N=637) 1.02 (1.01 − 1.0) <0.001 ***
Cholesterol (N=637) 1.09 (0.85 − 1.4) 0.482
eGFR (N=637) 0.99 (0.98 − 1.0) 0.052
HbA1c (N=637) 1.43 (1.21 − 1.7) <0.001 ***
Medication_Statins (N=637) 1.70 (0.91 − 3.2) 0.094
Sex (N=637) 1.20 (0.76 − 1.9) 0.435
Smoking (N=637) 1.52 (0.91 − 2.5) 0.106
TG_total_log (N=637) 0.92 (0.67 − 1.3) 0.596
UAER_log (N=637) 1.27 (1.15 − 1.4) <0.001 ***
Previous_CVD (N=637) 0.97 (0.59 − 1.6) 0.918
Events: 87; Global p−value (Log−Rank): 0
AIC: 901.04; Concordance Index: 0.85
1 1.5 2 2.5 3 3.5 4 4.5 5

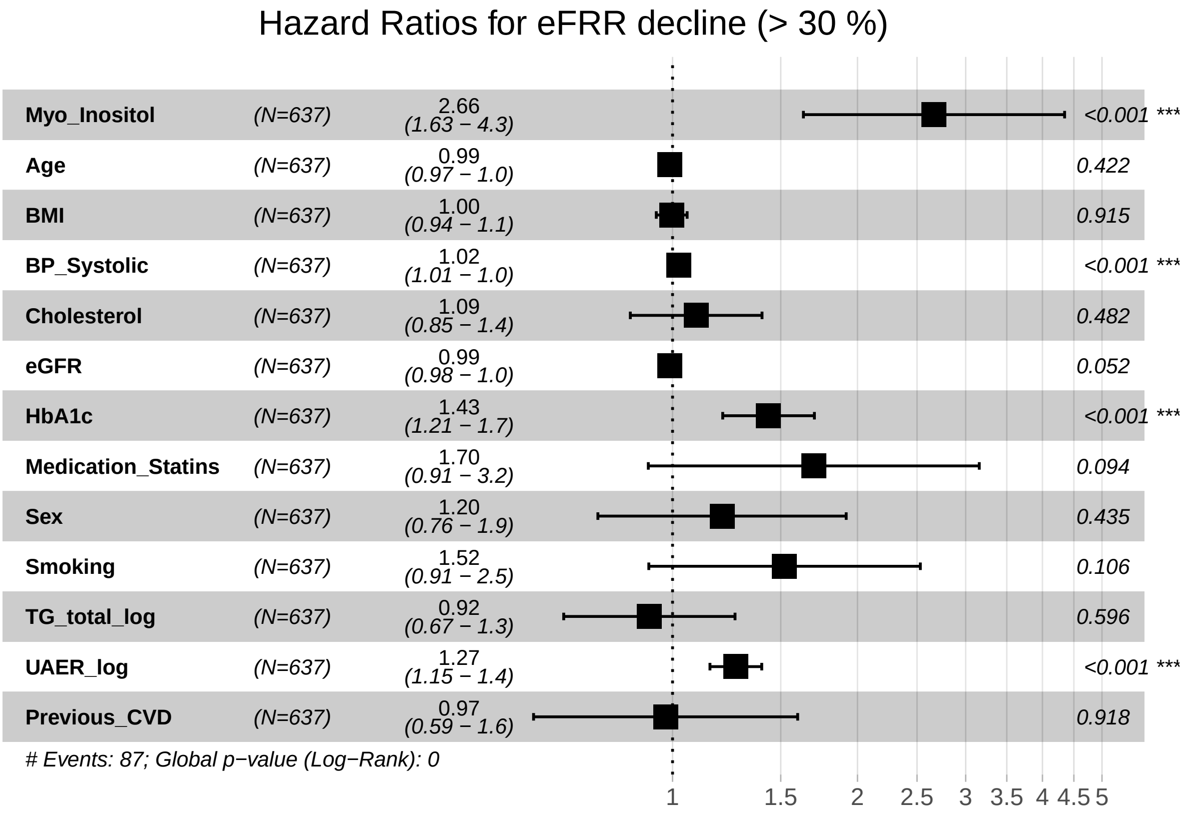
Hazard Ratios for eFRR decline (> 30 %)
Myo_Inositol (N=637) 2.66 (1.63 − 4.3) <0.001 ***
Age (N=637) 0.99 (0.97 − 1.0) 0.422
BMI (N=637) 1.00 (0.94 − 1.1) 0.915
BP_Systolic (N=637) 1.02 (1.01 − 1.0) <0.001 ***
Cholesterol (N=637) 1.09 (0.85 − 1.4) 0.482
eGFR (N=637) 0.99 (0.98 − 1.0) 0.052
HbA1c (N=637) 1.43 (1.21 − 1.7) <0.001 ***
Medication_Statins (N=637) 1.70 (0.91 − 3.2) 0.094
Sex (N=637) 1.20 (0.76 − 1.9) 0.435
Smoking (N=637) 1.52 (0.91 − 2.5) 0.106
TG_total_log (N=637) 0.92 (0.67 − 1.3) 0.596
UAER_log (N=637) 1.27 (1.15 − 1.4) <0.001 ***
Previous_CVD (N=637) 0.97 (0.59 − 1.6) 0.918
Events: 87; Global p−value (Log−Rank): 0
1 1.5 2 2.5 3 3.5 4 4.5 5

5.2 Adjustment to Any Retinopathy

5.2.1 Step 2B: Adjusted Model

5.2.1.1 Table

Table 13: Crude survival model for combined renal endpoint.

Name	exp(coef)	lower .95	upper .95	Pr(>|z|)	p.adj
Leucine, 2TMS; 19	0.591	0.464	0.753	2.15e-05	0.000646
Ribonic acid; 72	1.760	1.340	2.300	4.41e-05	0.001280
Isoleucine, 2TMS; 18	0.610	0.469	0.794	2.39e-04	0.006690
Valine, 2TMS; 20	0.433	0.271	0.692	4.68e-04	0.012600
Myo inositol 6TMS; 1	1.800	1.190	2.710	4.98e-03	0.130000
Methionine, 2TMS; 16	0.734	0.558	0.964	2.64e-02	0.660000
2-Hydroxybutyric acid, 2TMS; 22	0.760	0.595	0.970	2.73e-02	0.660000
4-Deoxytetronic acid; 33	0.799	0.651	0.979	3.04e-02	0.700000
3,4-Dihydroxybutanoic acid; 27	1.540	1.040	2.270	3.08e-02	0.700000
2,4-Dihydroxybutanoic acid; 28	1.360	0.942	1.970	1.01e-01	1.000000
Palmitic acid, TMS; 5	1.810	0.819	3.980	1.43e-01	1.000000
Malic acid, 3TMS; 11	1.340	0.896	2.000	1.54e-01	1.000000
Fumaric acid, 2TMS; 9	1.430	0.854	2.400	1.74e-01	1.000000
Creatinine; 50	1.170	0.932	1.460	1.79e-01	1.000000
Ribitol; 71	1.250	0.889	1.760	1.99e-01	1.000000
Hydroxyproline; 64	1.110	0.893	1.390	3.43e-01	1.000000
4-Hydroxyphenyllactic acid; 44	1.140	0.802	1.620	4.67e-01	1.000000
Glycine, 3TMS; 17	0.835	0.478	1.460	5.27e-01	1.000000
Glyceryl-glycoside; 59	1.110	0.806	1.520	5.29e-01	1.000000
4-Hydroxybenzeneacetic acid; 42	1.050	0.888	1.250	5.46e-01	1.000000
Succinic acid, 2TMS; 7	1.180	0.652	2.140	5.81e-01	1.000000
Stearic acid, TMS; 2	1.260	0.497	3.200	6.27e-01	1.000000
4-Deoxytetronic acid; 32	1.070	0.795	1.440	6.59e-01	1.000000
Citric acid, 4TMS; 6	0.916	0.602	1.390	6.80e-01	1.000000
Glycerol; 57	1.070	0.723	1.590	7.24e-01	1.000000
Pyroglutamic acid; 69	0.956	0.741	1.230	7.32e-01	1.000000
Serine, 3TMS; 14	0.926	0.574	1.500	7.54e-01	1.000000
Eicosapentaenoic acid; 55	0.969	0.774	1.210	7.82e-01	1.000000
2-hydroxy Isovaleric acid; 38	1.000	0.857	1.180	9.53e-01	1.000000
3-Indoleacetic acid; 40	1.000	0.780	1.290	9.72e-01	1.000000

5.2.1.2 Forest Plot

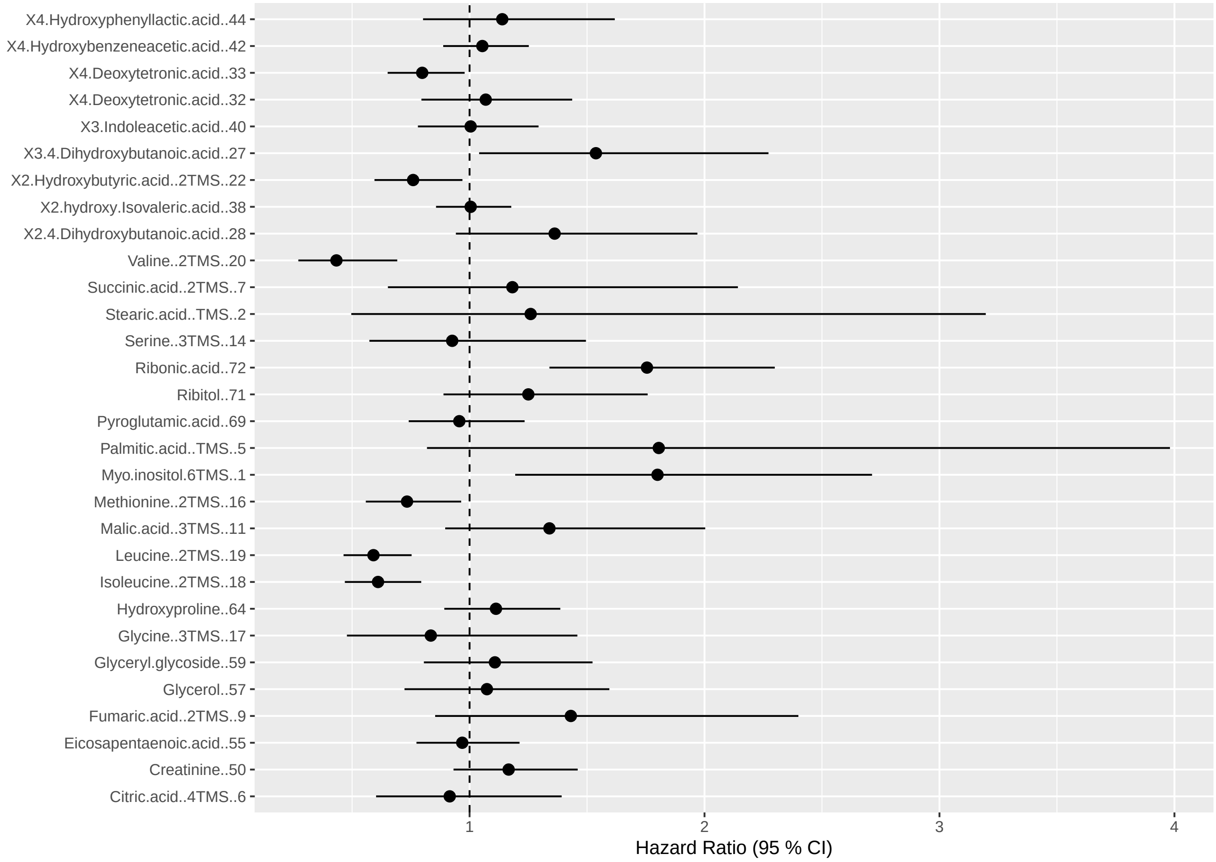

5.2.2 Step 3: Survival Analysis of Specific Renal Endpoints in Relation to Prioritized Metabolites from Step 2A

5.2.2.1 Step 3A: All-Cause Mortality

5.2.2.1.1 Table

Table 14: Adjusted survival model for all-cause mortality.

Name	exp(coef)	lower .95	upper .95	Pr(>|z|)	p.adj
X2.Hydroxybutyric.acid..2TMS..22	0.641	0.457	0.901	0.0104	0.146
Ribonic.acid..72	1.590	1.070	2.340	0.0209	0.272
Isoleucine..2TMS..18	0.695	0.477	1.010	0.0571	0.686
Ribitol..71	1.600	0.931	2.740	0.0892	0.981
Leucine..2TMS..19	0.750	0.505	1.110	0.1530	1.000
Myo.inositol.6TMS..1	1.560	0.846	2.890	0.1540	1.000
Fumaric.acid..2TMS..9	1.640	0.791	3.400	0.1830	1.000
Creatinine..50	1.310	0.863	2.000	0.2030	1.000
X2.4.Dihydroxybutanoic.acid..28	1.430	0.819	2.480	0.2100	1.000
Glyceryl.glycoside..59	1.360	0.826	2.240	0.2270	1.000
Valine..2TMS..20	0.671	0.342	1.320	0.2460	1.000
X3.4.Dihydroxybutanoic.acid..27	1.330	0.765	2.310	0.3120	1.000
X4.Hydroxybenzeneacetic.acid..42	1.100	0.856	1.410	0.4630	1.000
Methionine..2TMS..16	0.954	0.598	1.520	0.8430	1.000

5.2.2.1.2 Forest Plot

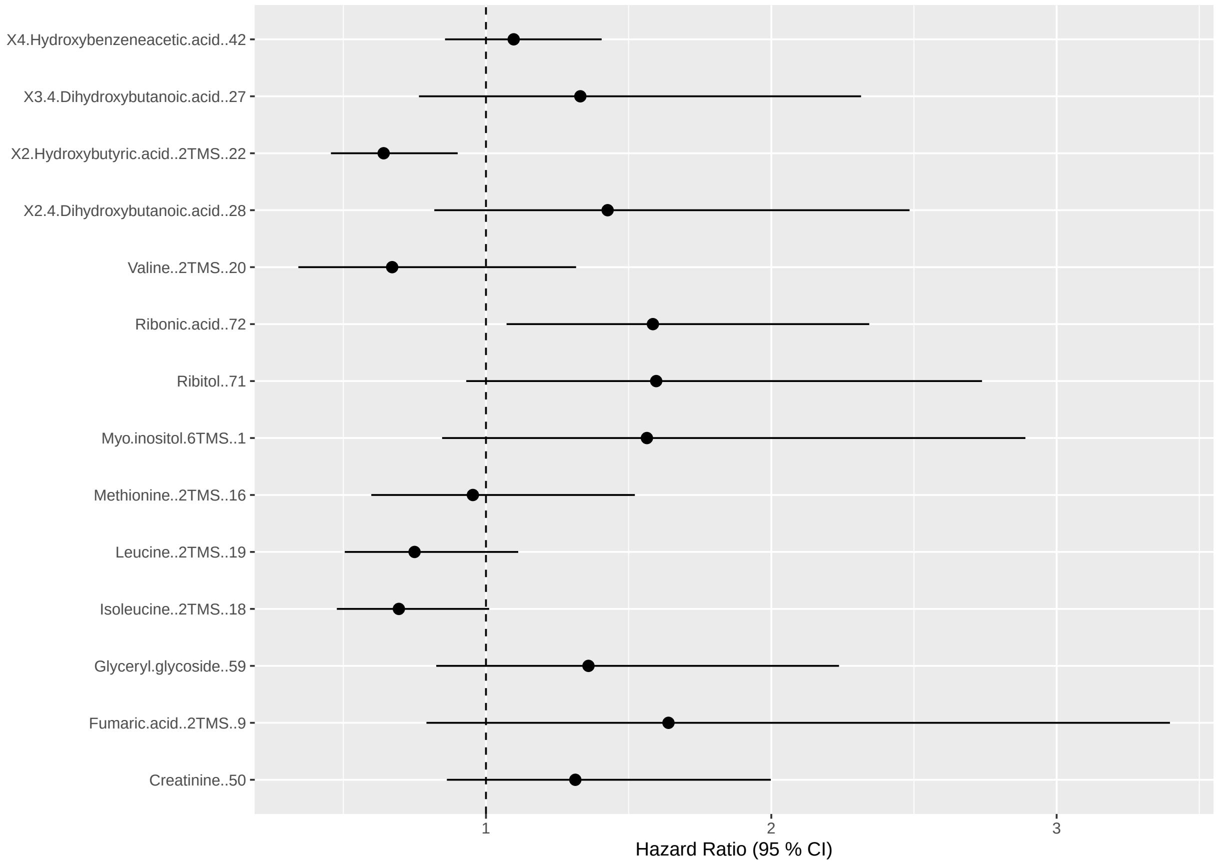

5.2.2.2 Step 3B: eGFR Decline > 30 %

5.2.2.2.1 Table

Table 15: Adjusted survival model for eGFR decline > 30 %.

Name	exp(coef)	lower .95	upper .95	Pr(>|z|)	p.adj
Ribonic.acid..72	2.220	1.650	3.000	2.00e-07	2.50e-06
Myo.inositol.6TMS..1	2.670	1.640	4.340	7.43e-05	9.66e-04
X3.4.Dihydroxybutanoic.acid..27	1.930	1.200	3.090	6.46e-03	7.75e-02
X2.4.Dihydroxybutanoic.acid..28	1.710	1.100	2.660	1.74e-02	1.91e-01
Isoleucine..2TMS..18	0.735	0.553	0.977	3.40e-02	3.40e-01
Valine..2TMS..20	0.542	0.306	0.961	3.60e-02	3.40e-01
X2.Hydroxybutyric.acid..2TMS..22	0.771	0.551	1.080	1.27e-01	1.00e+00
Methionine..2TMS..16	0.779	0.554	1.100	1.52e-01	1.00e+00
Creatinine..50	1.190	0.923	1.530	1.80e-01	1.00e+00
Ribitol..71	1.310	0.881	1.950	1.82e-01	1.00e+00
X4.Hydroxybenzeneacetic.acid..42	1.130	0.925	1.380	2.30e-01	1.00e+00
Leucine..2TMS..19	0.874	0.650	1.170	3.70e-01	1.00e+00
Glyceryl.glycoside..59	1.170	0.783	1.750	4.39e-01	1.00e+00
Fumaric.acid..2TMS..9	0.815	0.447	1.490	5.06e-01	1.00e+00

5.2.2.2.2 Forest Plot

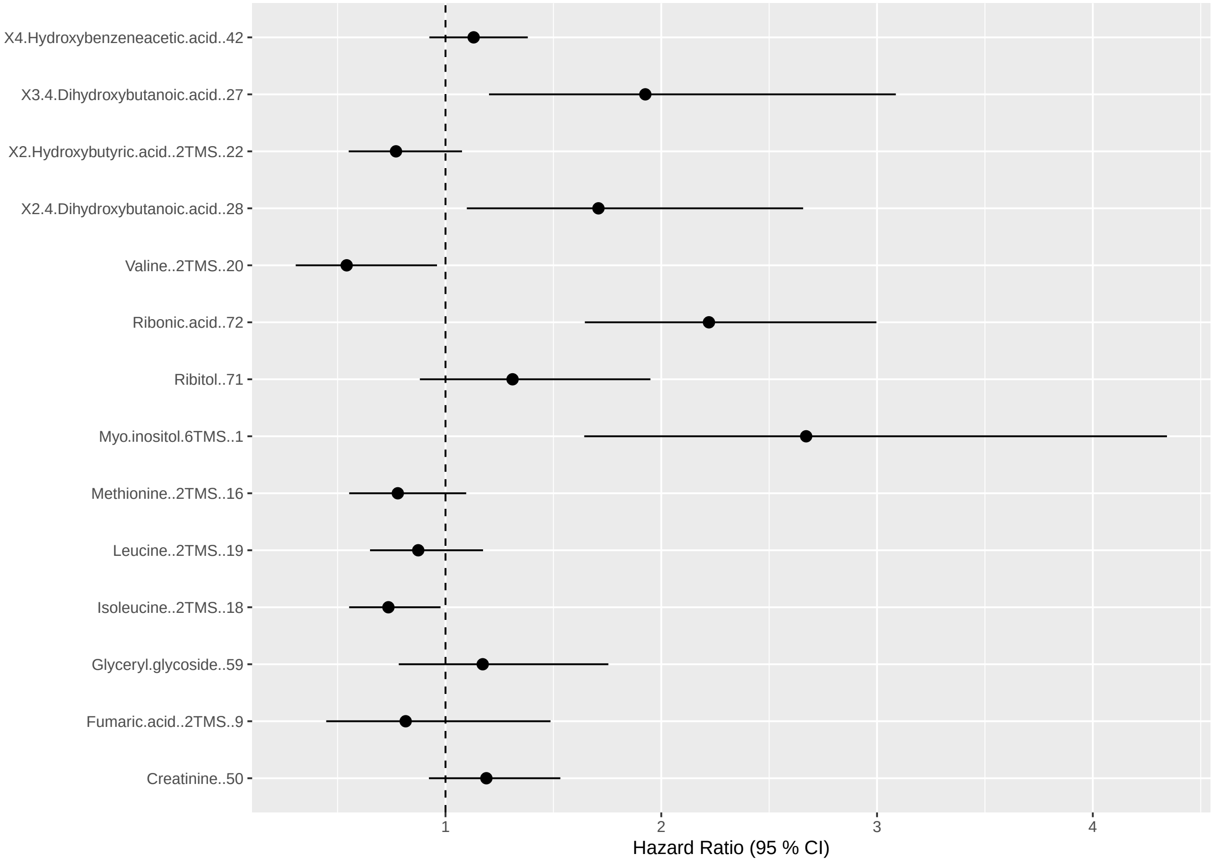

5.2.2.3 Step 3C: End-Stage Renal Disease

5.2.2.3.1 Adjusted Model

Table

Table 16: Adjusted survival model for end-stage renal disease.

Name	exp(coef)	lower .95	upper .95	Pr(>|z|)	p.adj
Ribitol..71	0.438	0.208	0.922	0.0297	0.415
Methionine..2TMS..16	0.583	0.305	1.110	0.1030	1.000
X4.Hydroxybenzeneacetic.acid..42	0.653	0.361	1.180	0.1570	1.000
Valine..2TMS..20	0.410	0.118	1.420	0.1590	1.000
Isoleucine..2TMS..18	0.773	0.463	1.290	0.3230	1.000
X2.Hydroxybutyric.acid..2TMS..22	0.719	0.364	1.420	0.3420	1.000
Fumaric.acid..2TMS..9	1.960	0.487	7.900	0.3430	1.000
Leucine..2TMS..19	0.835	0.541	1.290	0.4140	1.000
Glyceryl.glycoside..59	0.708	0.283	1.770	0.4600	1.000
Creatinine..50	0.879	0.551	1.400	0.5900	1.000
X3.4.Dihydroxybutanoic.acid..27	1.310	0.433	3.970	0.6320	1.000
Ribonic.acid..72	0.850	0.417	1.730	0.6540	1.000
X2.4.Dihydroxybutanoic.acid..28	1.010	0.335	3.060	0.9830	1.000
Myo.inositol.6TMS..1	0.997	0.315	3.160	0.9960	1.000

Forest Plot

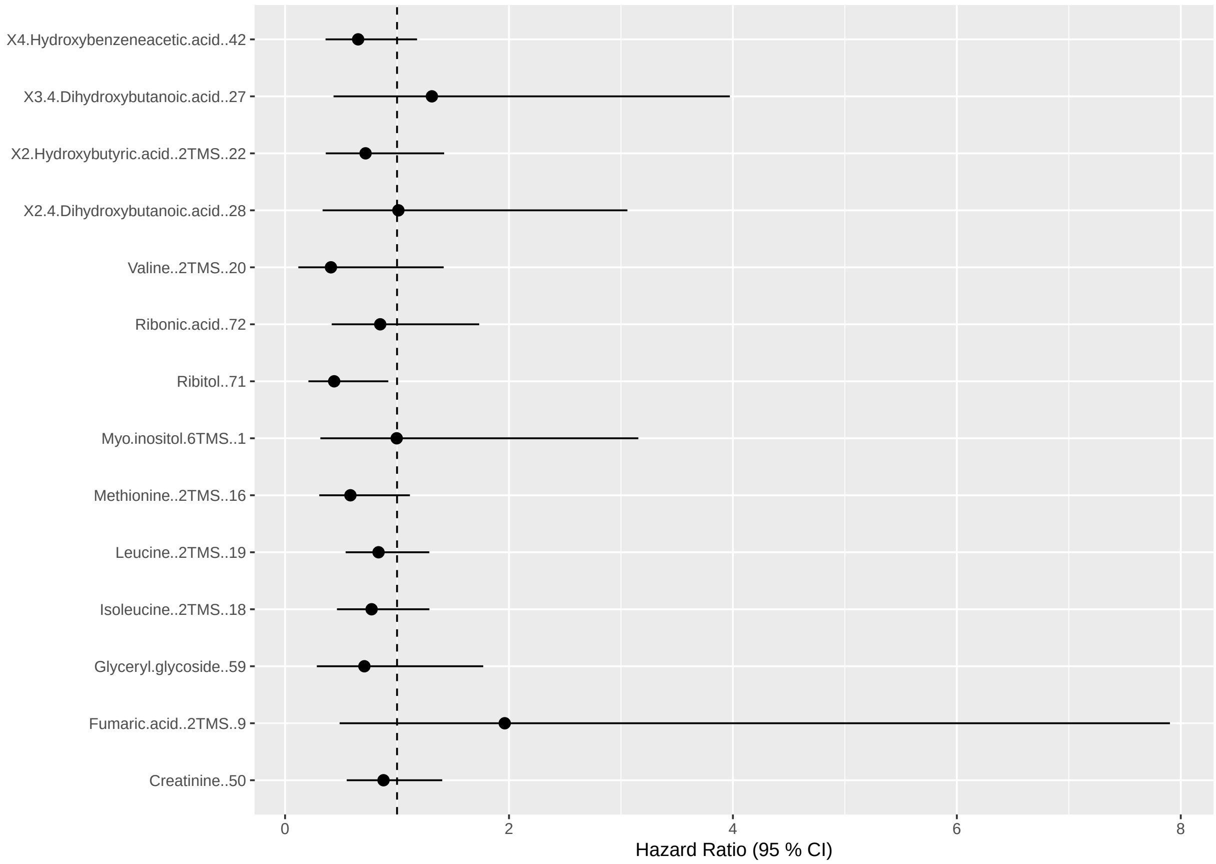

X4.Hydroxybenzeneacetic.acid..42
X3.4.Dihydroxybutanoic.acid..27
X2.Hydroxybutyric.acid..2TMS..22
X2.4.Dihydroxybutanoic.acid..28
Valine..2TMS..20
Ribonic.acid..72
Ribitol..71
Myo.inositol.6TMS..1
Methionine..2TMS..16
Leucine..2TMS..19
Isoleucine..2TMS..18
Glyceryl.glycoside..59
Fumaric.acid..2TMS..9
Creatinine..50
0
2
4
6
8
Hazard Ratio (95 % CI)

5.2.3 Compilation Forest Plot from Steps 3A-C

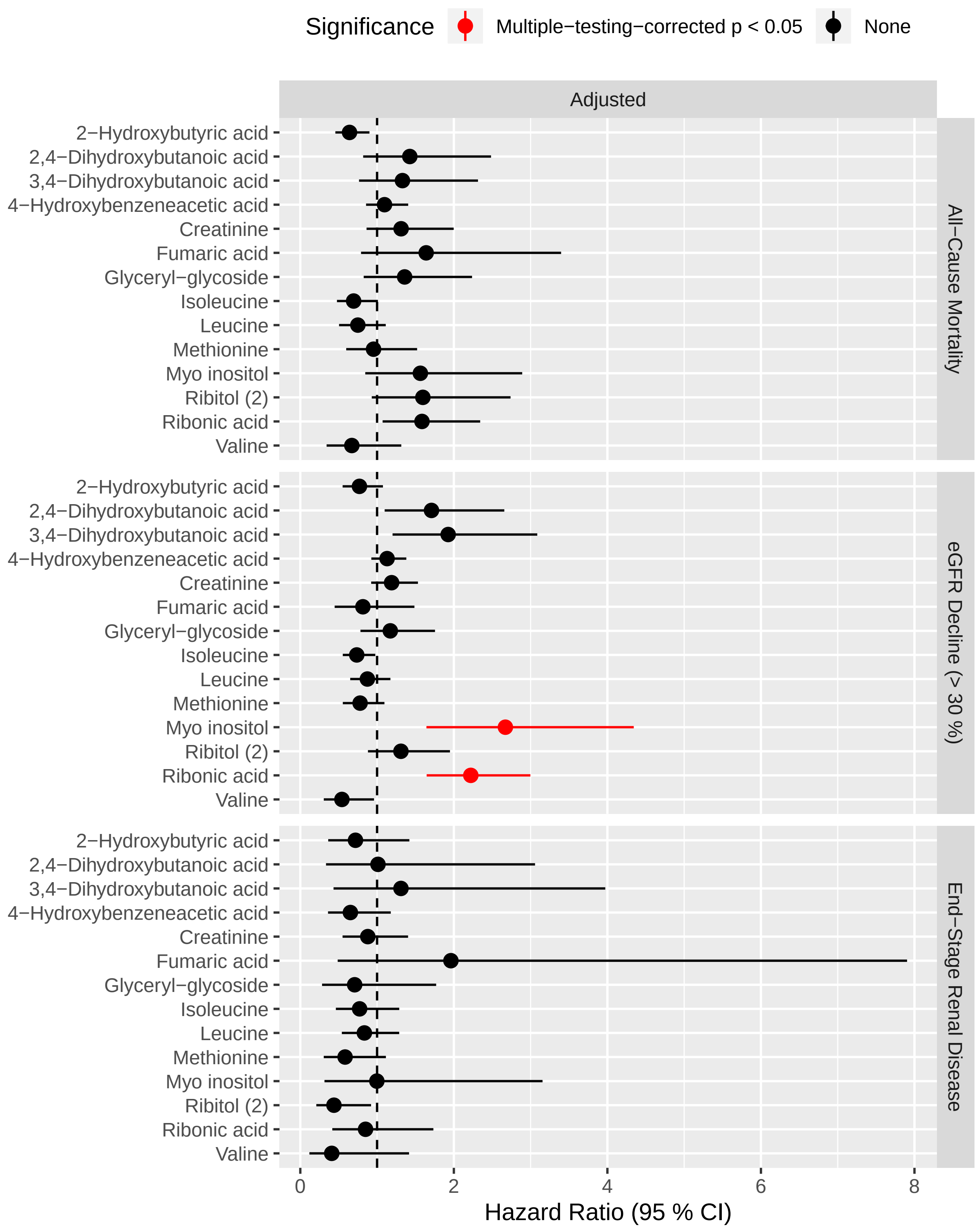

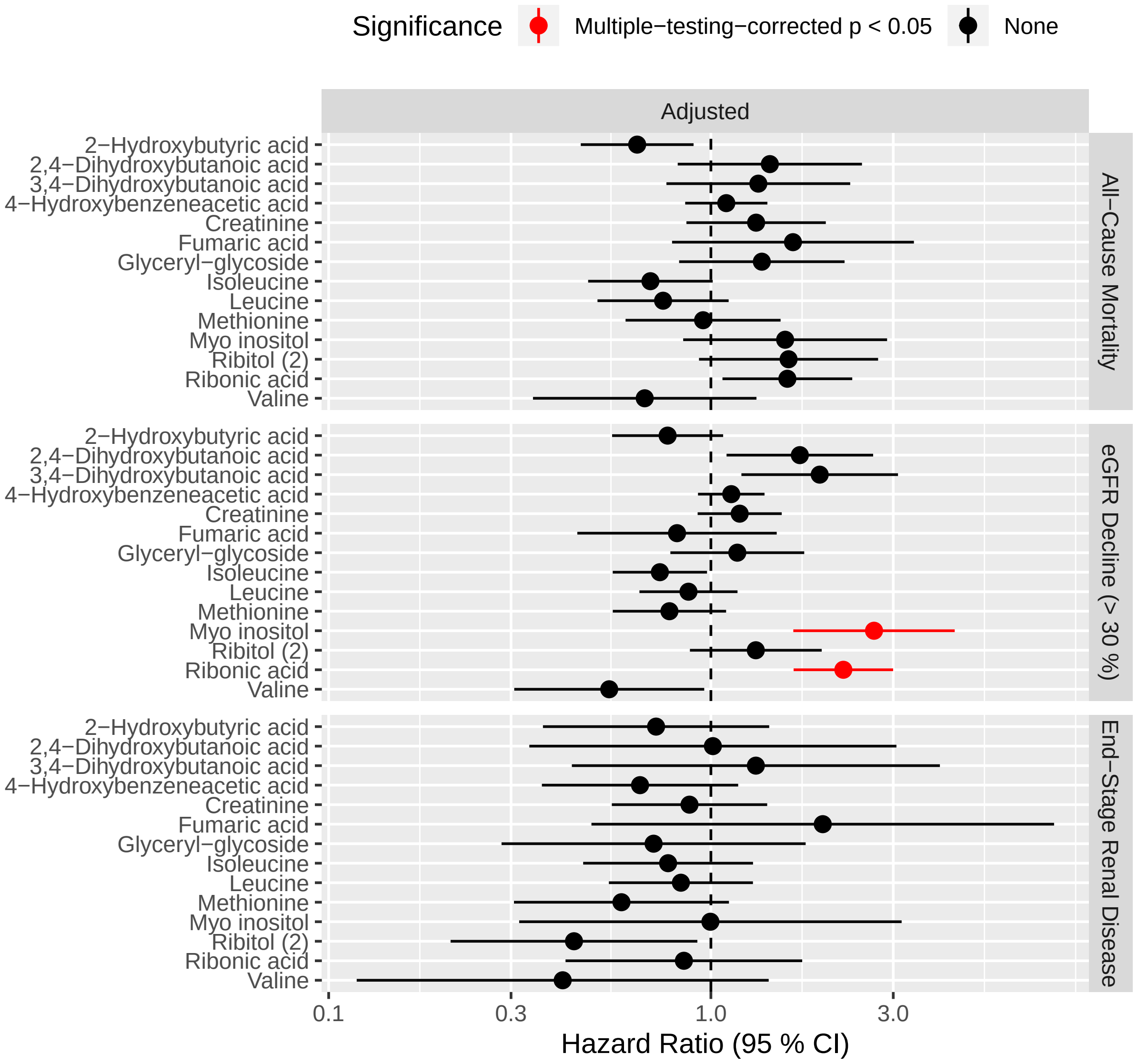
Significance
Multiple−testing−corrected p < 0.05
None
Adjusted
All−Cause Mortality
eGFR Decline (> 30 %)
End−Stage Renal Disease
2−Hydroxybutyric acid
2,4−Dihydroxybutanoic acid
3,4−Dihydroxybutanoic acid
4−Hydroxybenzeneacetic acid
Creatinine
Fumaric acid
Glyceryl−glycoside
Isoleucine
Leucine
Methionine
Myo inositol
Ribitol (2)
Ribonic acid
Valine
0.1
0.3
1.0
3.0
Hazard Ratio (95 % CI)

5.2.4 Step 4: Detailed Assessment of the Top-Metabolites in Relation to Outcomes

5.2.4.1 Ribonic Acid

```
## Call:
## survival::coxph(formula = survival::Surv(time = t_gfrfald30_p,
##     event = censor_gfrfald30_p.reversed.numeric) ~ Ribonic_acid +
##     Age + BMI + BP_Systolic + Cholesterol + eGFR + HbA1c + Medication_Statins +
##     Sex + Smoking + TG_total_log + UAER_log + Retinopathy_Any,
##     data = data.survival)
##
##   n= 586, number of events= 87
##    (51 observations deleted due to missingness)
##
##                         coef exp(coef)  se(coef)      z Pr(>|z|)
## Ribonic_acid        0.797929  2.220937  0.152909  5.218 1.81e-07 ***
## Age                -0.012569  0.987510  0.012716 -0.988 0.322964
## BMI                -0.003676  0.996331  0.029456 -0.125 0.900691
## BP_Systolic         0.025383  1.025708  0.006689  3.795 0.000148 ***
## Cholesterol         0.162334  1.176253  0.133532  1.216 0.224103
## eGFR               -0.009391  0.990653  0.005010 -1.874 0.060889 .
## HbA1c               0.422031  1.525056  0.083897  5.030 4.90e-07 ***
## Medication_Statins  0.686473  1.986697  0.317809  2.160 0.030771 *
## Sex                 0.158591  1.171859  0.241232  0.657 0.510908
## Smoking             0.522183  1.685704  0.259605  2.011 0.044278 *
## TG_total_log       -0.127349  0.880427  0.156714 -0.813 0.416436
## UAER_log            0.259575  1.296379  0.049892  5.203 1.96e-07 ***
## Retinopathy_Any    -0.336197  0.714482  0.331369 -1.015 0.310311
## ---
## Signif. codes:  0 '***' 0.001 '**' 0.01 '*' 0.05 '.' 0.1 ' ' 1
##
##                    exp(coef) exp(-coef) lower .95 upper .95
## Ribonic_acid          2.2209     0.4503    1.6458     2.997
## Age                   0.9875     1.0126    0.9632     1.012
## BMI                   0.9963     1.0037    0.9404     1.056
## BP_Systolic           1.0257     0.9749    1.0123     1.039
## Cholesterol           1.1763     0.8502    0.9054     1.528
## eGFR                  0.9907     1.0094    0.9810     1.000
## HbA1c                 1.5251     0.6557    1.2938     1.798
## Medication_Statins    1.9867     0.5033    1.0656     3.704
## Sex                   1.1719     0.8533    0.7304     1.880
## Smoking               1.6857     0.5932    1.0135     2.804
## TG_total_log          0.8804     1.1358    0.6476     1.197
## UAER_log              1.2964     0.7714    1.1756     1.430
## Retinopathy_Any       0.7145     1.3996    0.3732     1.368
##
## Concordance= 0.856  (se = 0.032 )
## Rsquare= 0.251   (max possible= 0.828 )
## Likelihood ratio test= 169.2  on 13 df,   p=0
## Wald test            = 148.9  on 13 df,   p=0
## Score (logrank) test = 193.3  on 13 df,   p=0
```

5.2.4.1.1 Forest Plot with Clinical Variables

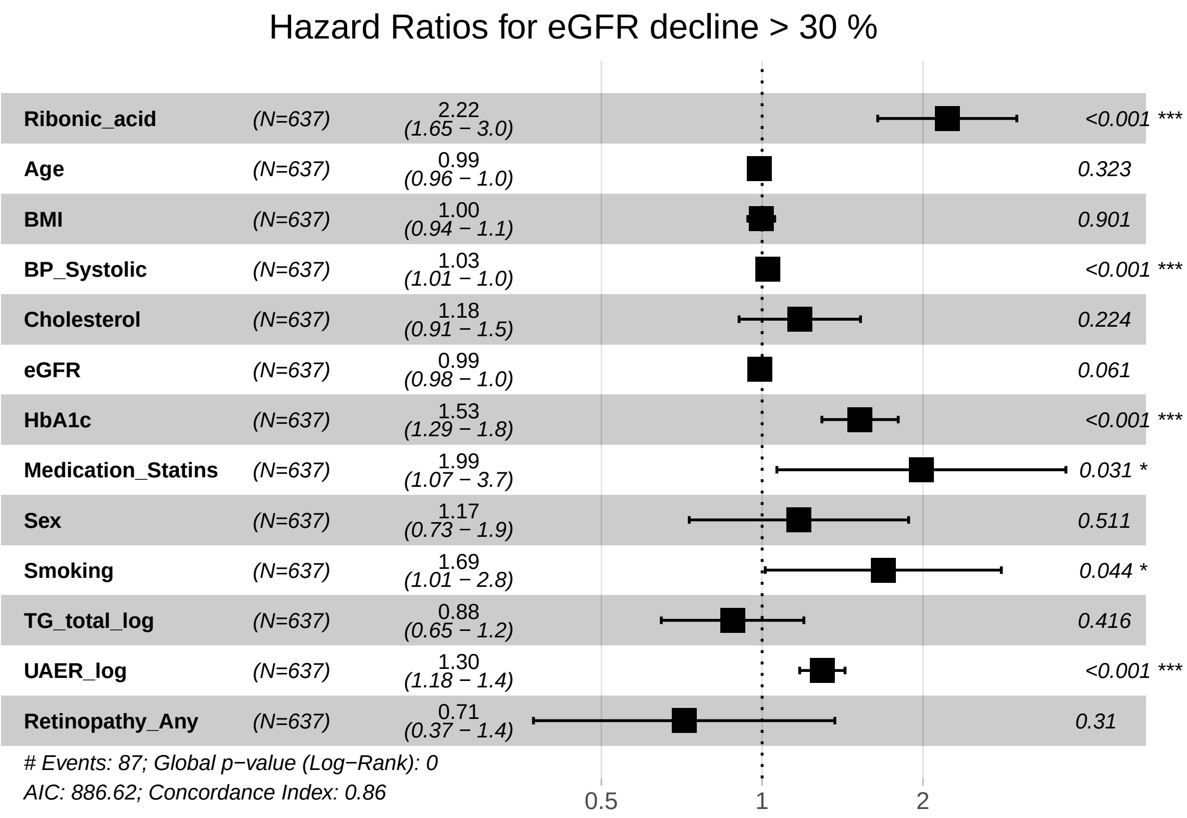
Hazard Ratios for eGFR decline > 30 %
Ribonic_acid (N=637) 2.22 (1.65 − 3.0) <0.001 ***
Age (N=637) 0.99 (0.96 − 1.0) 0.323
BMI (N=637) 1.00 (0.94 − 1.1) 0.901
BP_Systolic (N=637) 1.03 (1.01 − 1.0) <0.001 ***
Cholesterol (N=637) 1.18 (0.91 − 1.5) 0.224
eGFR (N=637) 0.99 (0.98 − 1.0) 0.061
HbA1c (N=637) 1.53 (1.29 − 1.8) <0.001 ***
Medication_Statins (N=637) 1.99 (1.07 − 3.7) 0.031 *
Sex (N=637) 1.17 (0.73 − 1.9) 0.511
Smoking (N=637) 1.69 (1.01 − 2.8) 0.044 *
TG_total_log (N=637) 0.88 (0.65 − 1.2) 0.416
UAER_log (N=637) 1.30 (1.18 − 1.4) <0.001 ***
Retinopathy_Any (N=637) 0.71 (0.37 − 1.4) 0.31
Events: 87; Global p−value (Log−Rank): 0
AIC: 886.62; Concordance Index: 0.86
0.5
1
2

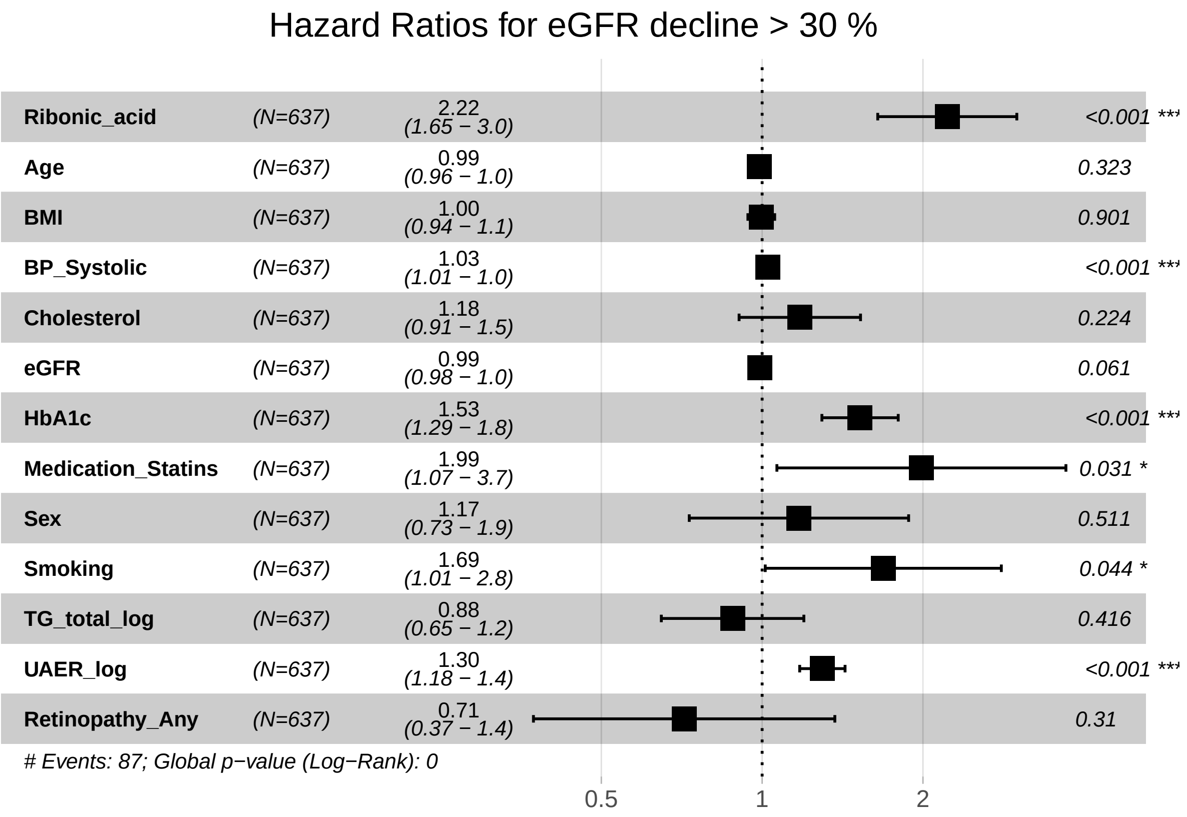
Hazard Ratios for eGFR decline > 30 %
Ribonic_acid (N=637) 2.22 (1.65 − 3.0) <0.001 ***
Age (N=637) 0.99 (0.96 − 1.0) 0.323
BMI (N=637) 1.00 (0.94 − 1.1) 0.901
BP_Systolic (N=637) 1.03 (1.01 − 1.0) <0.001 ***
Cholesterol (N=637) 1.18 (0.91 − 1.5) 0.224
eGFR (N=637) 0.99 (0.98 − 1.0) 0.061
HbA1c (N=637) 1.53 (1.29 − 1.8) <0.001 ***
Medication_Statins (N=637) 1.99 (1.07 − 3.7) 0.031 *
Sex (N=637) 1.17 (0.73 − 1.9) 0.511
Smoking (N=637) 1.69 (1.01 − 2.8) 0.044 *
TG_total_log (N=637) 0.88 (0.65 − 1.2) 0.416
UAER_log (N=637) 1.30 (1.18 − 1.4) <0.001 ***
Retinopathy_Any (N=637) 0.71 (0.37 − 1.4) 0.31
Events: 87; Global p−value (Log−Rank): 0
0.5
1
2

5.2.4.2 Myo-Inositol

```
## Call:
## survival::coxph(formula = survival::Surv(time = t_gfrfald30_p,
##     event = censor_gfrfald30_p.reversed.numeric) ~ Myo_Inositol +
##     Age + BMI + BP_Systolic + Cholesterol + eGFR + HbA1c + Medication_Statins +
##     Sex + Smoking + TG_total_log + UAER_log + Retinopathy_Any,
##     data = data.survival)
##
##   n= 586, number of events= 87
##    (51 observations deleted due to missingness)
##
##                         coef exp(coef)  se(coef)      z Pr(>|z|)
## Myo_Inositol        0.982619  2.671444  0.248010  3.962 7.43e-05 ***
## Age                -0.009415  0.990629  0.012421 -0.758 0.448456
## BMI                -0.001529  0.998472  0.028902 -0.053 0.957797
## BP_Systolic         0.022522  1.022777  0.006691  3.366 0.000763 ***
## Cholesterol         0.084799  1.088498  0.127218  0.667 0.505050
## eGFR               -0.010885  0.989174  0.005337 -2.040 0.041379 *
## HbA1c               0.357736  1.430087  0.086273  4.147 3.37e-05 ***
## Medication_Statins  0.595016  1.813060  0.318412  1.869 0.061665 .
## Sex                 0.185984  1.204403  0.235677  0.789 0.430026
## Smoking             0.424707  1.529142  0.258864  1.641 0.100869
## TG_total_log       -0.087589  0.916138  0.163568 -0.535 0.592312
## UAER_log            0.246614  1.279685  0.049898  4.942 7.72e-07 ***
## Retinopathy_Any    -0.391504  0.676040  0.332915 -1.176 0.239600
## ---
## Signif. codes:  0 '***' 0.001 '**' 0.01 '*' 0.05 '.' 0.1 ' ' 1
##
##                    exp(coef) exp(-coef) lower .95 upper .95
## Myo_Inositol          2.6714     0.3743    1.6430    4.3436
## Age                   0.9906     1.0095    0.9668    1.0150
## BMI                   0.9985     1.0015    0.9435    1.0567
## BP_Systolic           1.0228     0.9777    1.0095    1.0363
## Cholesterol           1.0885     0.9187    0.8483    1.3967
## eGFR                  0.9892     1.0109    0.9789    0.9996
## HbA1c                 1.4301     0.6993    1.2076    1.6935
## Medication_Statins    1.8131     0.5516    0.9714    3.3841
## Sex                   1.2044     0.8303    0.7589    1.9115
## Smoking               1.5291     0.6540    0.9207    2.5398
## TG_total_log          0.9161     1.0915    0.6649    1.2624
## UAER_log              1.2797     0.7814    1.1605    1.4112
## Retinopathy_Any       0.6760     1.4792    0.3520    1.2982
##
## Concordance= 0.846  (se = 0.032 )
## Rsquare= 0.234   (max possible= 0.828 )
## Likelihood ratio test= 156.1  on 13 df,   p=0
## Wald test            = 152.6  on 13 df,   p=0
## Score (logrank) test = 190.3  on 13 df,   p=0
```

5.2.4.2.1 Forest Plot with Clinical Variables

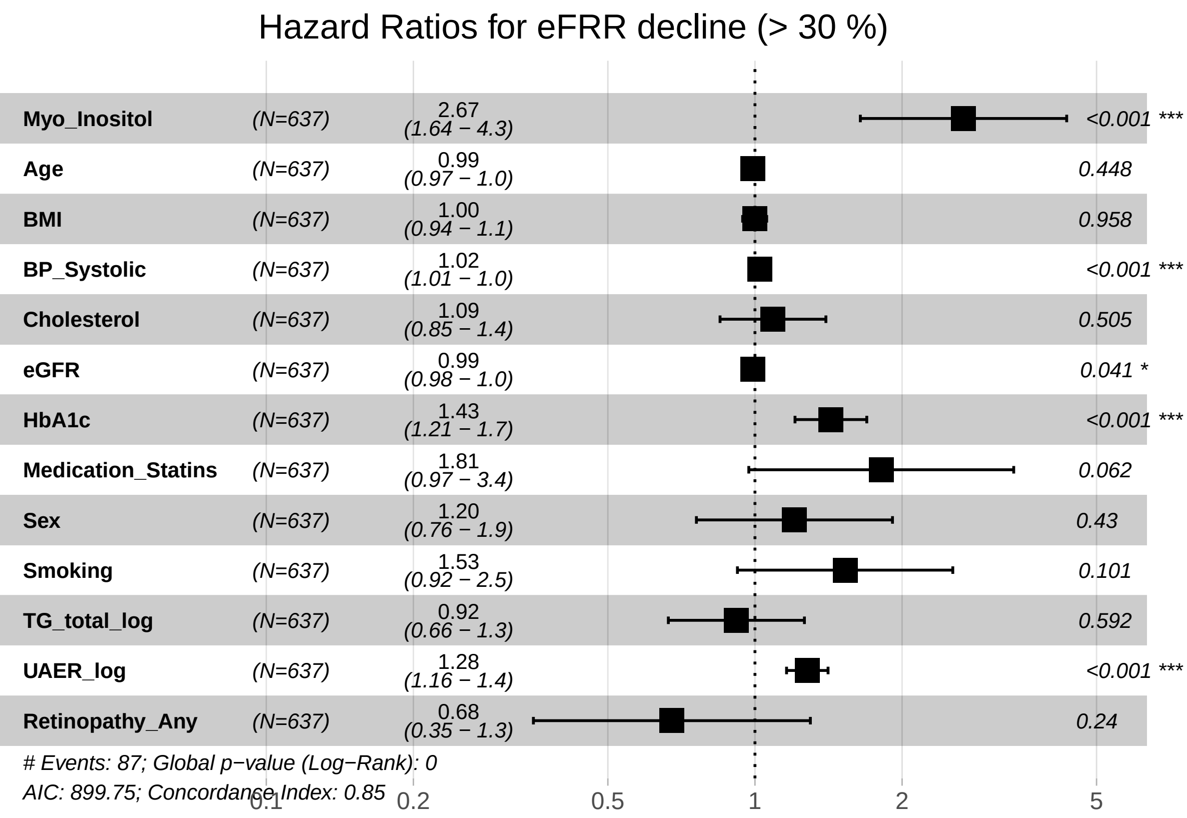

Hazard Ratios for eFRR decline (> 30 %)
Myo_Inositol (N=637) 2.67 (1.64 − 4.3) <0.001 ***
Age (N=637) 0.99 (0.97 − 1.0) 0.448
BMI (N=637) 1.00 (0.94 − 1.1) 0.958
BP_Systolic (N=637) 1.02 (1.01 − 1.0) <0.001 ***
Cholesterol (N=637) 1.09 (0.85 − 1.4) 0.505
eGFR (N=637) 0.99 (0.98 − 1.0) 0.041 *
HbA1c (N=637) 1.43 (1.21 − 1.7) <0.001 ***
Medication_Statins (N=637) 1.81 (0.97 − 3.4) 0.062
Sex (N=637) 1.20 (0.76 − 1.9) 0.43
Smoking (N=637) 1.53 (0.92 − 2.5) 0.101
TG_total_log (N=637) 0.92 (0.66 − 1.3) 0.592
UAER_log (N=637) 1.28 (1.16 − 1.4) <0.001 ***
Retinopathy_Any (N=637) 0.68 (0.35 − 1.3) 0.24
Events: 87; Global p−value (Log−Rank): 0
AIC: 899.75; Concordance Index: 0.85
0.1 0.2 0.5 1 2 5

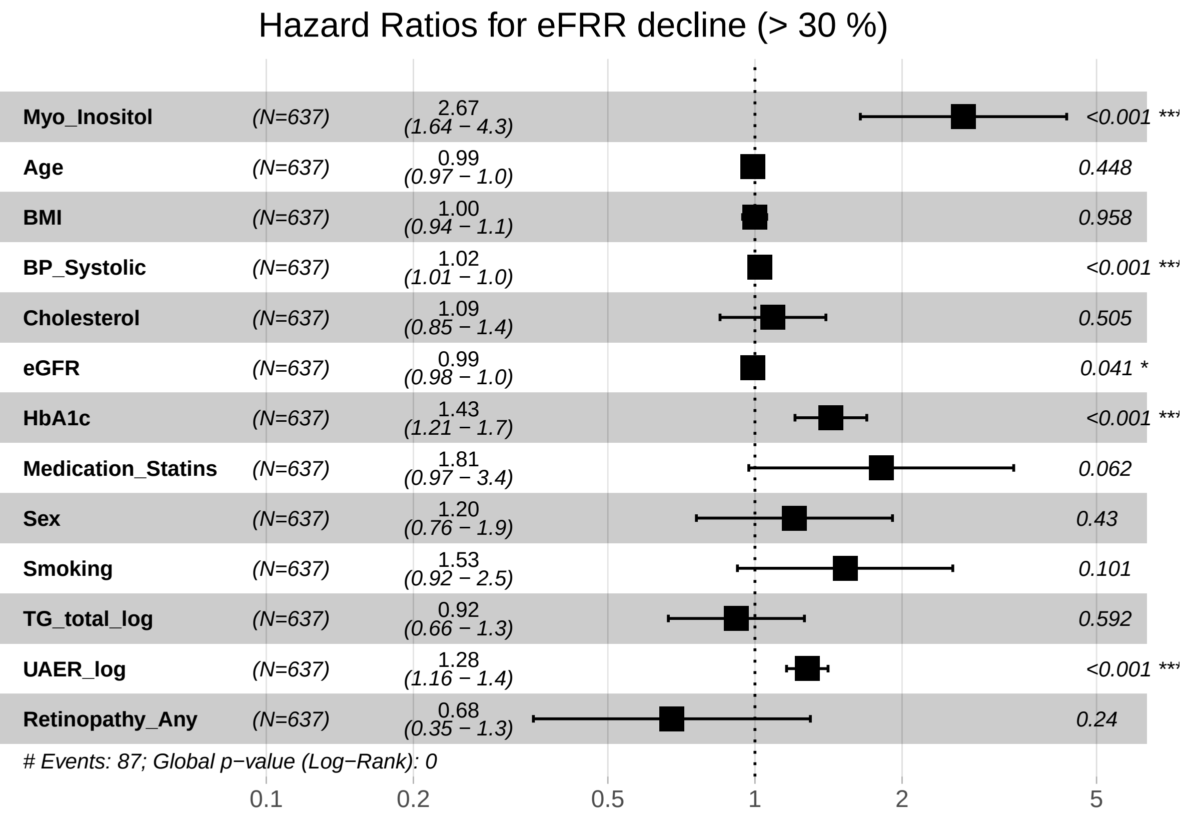

Hazard Ratios for eFRR decline (> 30 %)
Myo_Inositol (N=637) 2.67 (1.64 − 4.3) <0.001 ***
Age (N=637) 0.99 (0.97 − 1.0) 0.448
BMI (N=637) 1.00 (0.94 − 1.1) 0.958
BP_Systolic (N=637) 1.02 (1.01 − 1.0) <0.001 ***
Cholesterol (N=637) 1.09 (0.85 − 1.4) 0.505
eGFR (N=637) 0.99 (0.98 − 1.0) 0.041 *
HbA1c (N=637) 1.43 (1.21 − 1.7) <0.001 ***
Medication_Statins (N=637) 1.81 (0.97 − 3.4) 0.062
Sex (N=637) 1.20 (0.76 − 1.9) 0.43
Smoking (N=637) 1.53 (0.92 − 2.5) 0.101
TG_total_log (N=637) 0.92 (0.66 − 1.3) 0.592
UAER_log (N=637) 1.28 (1.16 − 1.4) <0.001 ***
Retinopathy_Any (N=637) 0.68 (0.35 − 1.3) 0.24
Events: 87; Global p−value (Log−Rank): 0
0.1 0.2 0.5 1 2 5

5.3 eGFR Decline > 40 %

5.3.1 Step 3B

5.3.1.1 Crude Model

5.3.1.1.1 Table

Table 17: Crude survival model for eGFR decline > 30 %

Name	exp(coef)	lower .95	upper .95	Pr(>|z|)	p.adj
Myo.inositol.6TMS..1	4.530	2.740	7.470	0.00e+00	0.00e+00
Ribonic.acid..72	2.510	1.810	3.490	0.00e+00	6.00e-07
Ribitol..71	3.230	2.110	4.950	1.00e-07	9.00e-07
X3.4.Dihydroxybutanoic.acid..27	3.610	2.250	5.780	1.00e-07	1.20e-06
X2.4.Dihydroxybutanoic.acid..28	2.680	1.730	4.150	1.07e-05	1.07e-04
Creatinine..50	2.110	1.450	3.070	9.10e-05	8.19e-04
Valine..2TMS..20	0.309	0.165	0.576	2.26e-04	1.81e-03
X4.Hydroxybenzeneacetic.acid..42	1.430	1.130	1.810	3.16e-03	2.21e-02
Methionine..2TMS..16	0.621	0.434	0.889	9.22e-03	5.53e-02
Glyceryl.glycoside..59	1.840	1.150	2.950	1.04e-02	5.53e-02
Isoleucine..2TMS..18	0.681	0.480	0.967	3.15e-02	1.26e-01
Leucine..2TMS..19	0.753	0.509	1.120	1.57e-01	4.71e-01
X2.Hydroxybutyric.acid..2TMS..22	0.781	0.553	1.100	1.61e-01	4.71e-01
Fumaric.acid..2TMS..9	1.580	0.781	3.200	2.03e-01	4.71e-01

5.3.1.1.2 Forest Plot

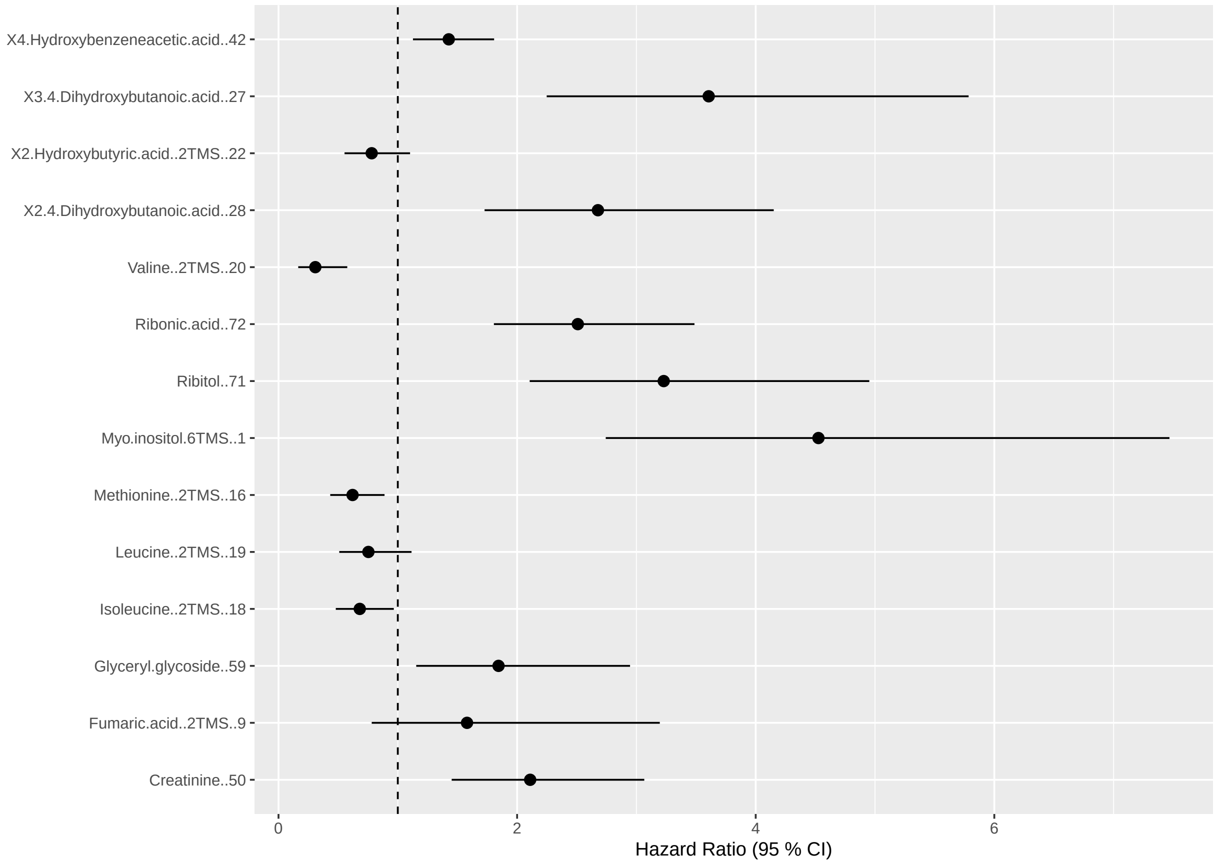

5.3.1.2 Adjusted Model

5.3.1.2.1 Table

Table 18: Adjusted survival model for eGFR decline > 30 %.

Name	exp(coef)	lower .95	upper .95	Pr(>|z|)	p.adj
Ribonic.acid..72	2.140	1.440	3.190	0.000174	0.00244
Valine..2TMS..20	0.274	0.129	0.585	0.000819	0.01060
Myo.inositol.6TMS..1	2.590	1.390	4.830	0.002810	0.03370
Ribitol..71	1.770	0.990	3.150	0.054100	0.59500
Creatinine..50	1.470	0.984	2.190	0.059700	0.59700
X2.4.Dihydroxybutanoic.acid..28	1.670	0.951	2.950	0.074100	0.66700
Isoleucine..2TMS..18	0.723	0.485	1.080	0.112000	0.89900
X2.Hydroxybutyric.acid..2TMS..22	0.700	0.442	1.110	0.127000	0.89900
Methionine..2TMS..16	0.744	0.468	1.180	0.212000	1.00000
Leucine..2TMS..19	0.803	0.551	1.170	0.254000	1.00000
X3.4.Dihydroxybutanoic.acid..27	1.450	0.754	2.770	0.268000	1.00000
X4.Hydroxybenzeneacetic.acid..42	1.090	0.849	1.410	0.487000	1.00000
Fumaric.acid..2TMS..9	0.864	0.381	1.960	0.726000	1.00000
Glyceryl.glycoside..59	1.100	0.621	1.950	0.742000	1.00000

5.3.1.2.2 Forest Plot

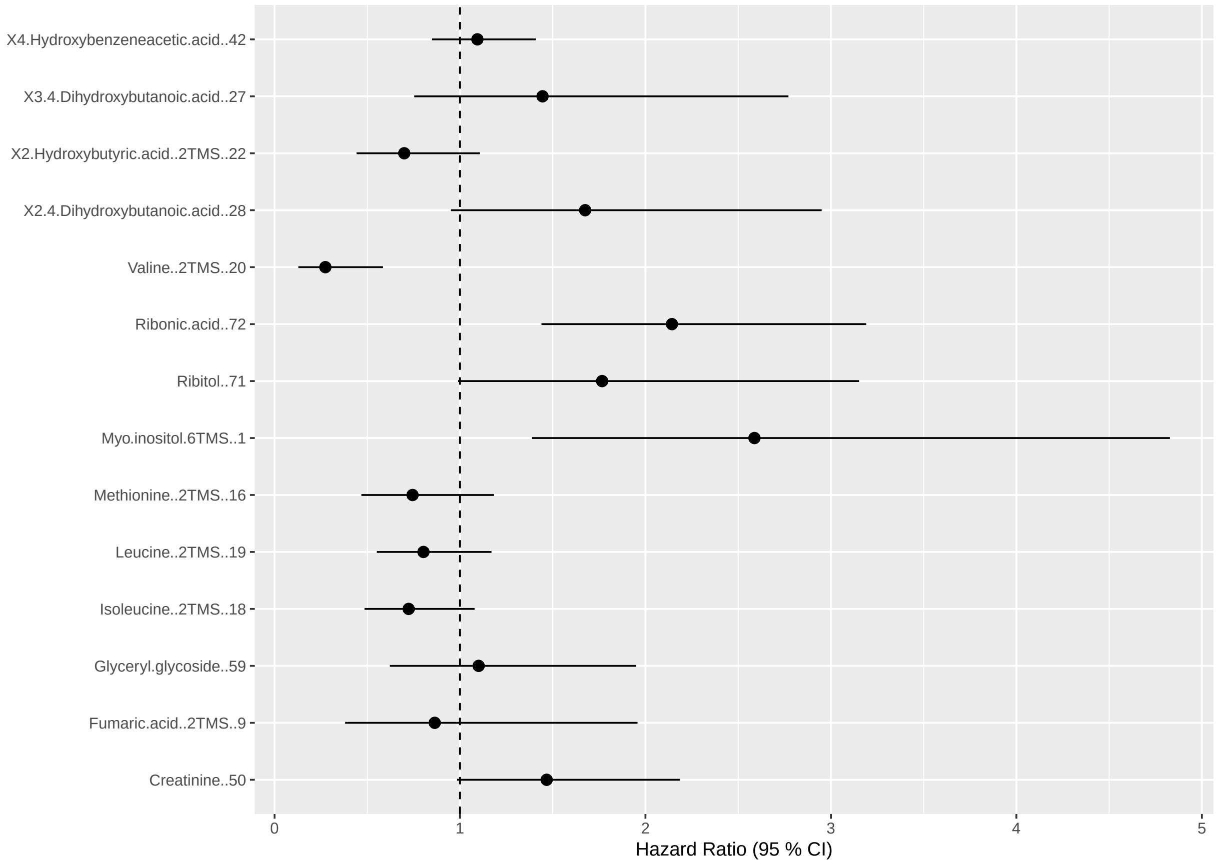

5.3.1.3 Combined Forest Plot from Crude and Adjusted Models

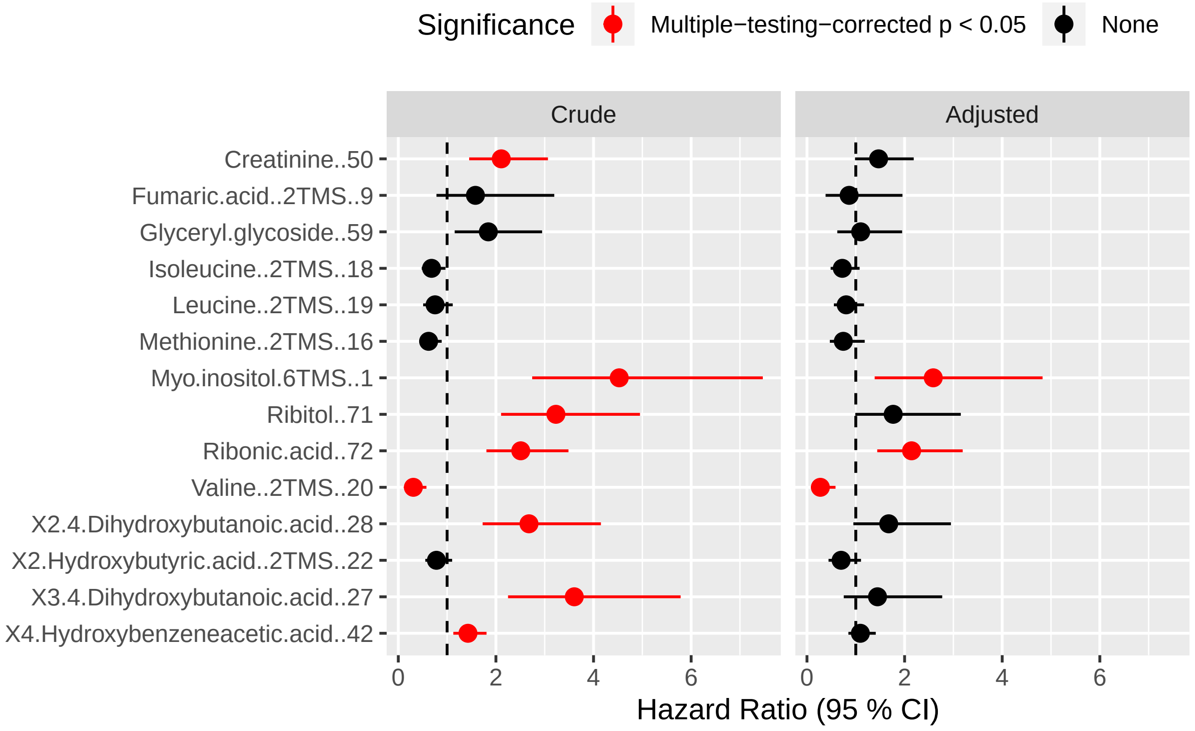

5.3.2 Step 4: Detailed Assessment of the Top-Metabolites in Relation to Outcomes

5.3.2.1 Step 4.1: First Top-Metabolite in Relation to eGFR Decline (> 40 %)

5.3.2.1.1 Step 4.1A: Analysis of Full Cohort

Survival Model with Details

```
## Call:
## survival::coxph(formula = survival::Surv(time = t_gfrfald40_p,
##     event = censor_gfrfald40_p.reversed.numeric) ~ Ribonic_acid +
##     Age + BMI + BP_Systolic + Cholesterol + eGFR + HbA1c + Medication_Statins +
##     Sex + Smoking + TG_total_log + UAER_log, data = data.survival)
##
##   n= 586, number of events= 50
##    (51 observations deleted due to missingness)
##
##                         coef exp(coef)  se(coef)      z Pr(>|z|)
## Ribonic_acid        0.762219  2.143026  0.203057  3.754 0.000174 ***
## Age                -0.025703  0.974624  0.017000 -1.512 0.130549
## BMI                -0.046082  0.954963  0.041053 -1.123 0.261646
## BP_Systolic         0.028213  1.028615  0.008595  3.282 0.001029 **
## Cholesterol         0.128966  1.137651  0.180036  0.716 0.473787
## eGFR               -0.007508  0.992521  0.006536 -1.149 0.250699
## HbA1c               0.415378  1.514944  0.118128  3.516 0.000438 ***
## Medication_Statins  0.673058  1.960222  0.417599  1.612 0.107020
## Sex                 0.434845  1.544723  0.332016  1.310 0.190294
## Smoking             0.510371  1.665909  0.350296  1.457 0.145124
## TG_total_log       -0.237178  0.788851  0.204406 -1.160 0.245915
## UAER_log            0.298286  1.347547  0.069527  4.290 1.78e-05 ***
## ---
## Signif. codes:  0 '***' 0.001 '**' 0.01 '*' 0.05 '.' 0.1 ' ' 1
##
##                    exp(coef) exp(-coef) lower .95 upper .95
## Ribonic_acid          2.1430     0.4666    1.4394     3.191
## Age                   0.9746     1.0260    0.9427     1.008
## BMI                   0.9550     1.0472    0.8811     1.035
## BP_Systolic           1.0286     0.9722    1.0114     1.046
## Cholesterol           1.1377     0.8790    0.7994     1.619
## eGFR                  0.9925     1.0075    0.9799     1.005
## HbA1c                 1.5149     0.6601    1.2018     1.910
## Medication_Statins    1.9602     0.5101    0.8647     4.444
## Sex                   1.5447     0.6474    0.8058     2.961
## Smoking               1.6659     0.6003    0.8385     3.310
## TG_total_log          0.7889     1.2677    0.5285     1.178
## UAER_log              1.3475     0.7421    1.1759     1.544
##
## Concordance= 0.887  (se = 0.042 )
## Rsquare= 0.185   (max possible= 0.638 )
## Likelihood ratio test= 119.7  on 12 df,   p=0
## Wald test            = 107.9  on 12 df,   p=0
## Score (logrank) test = 149  on 12 df,   p=0
```

Forest Plot with Clinical Variables

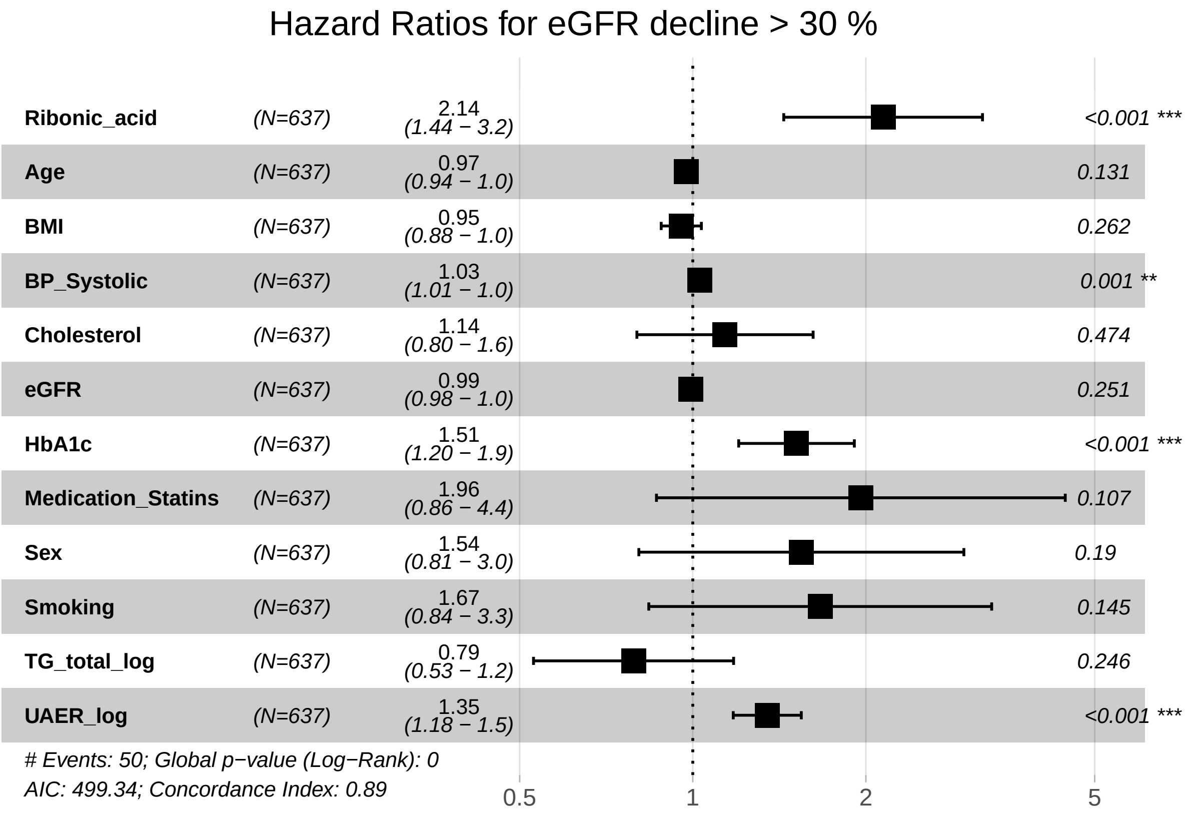

5.3.2.1.2 Kaplan-Maier Curve with Median Cutpoint

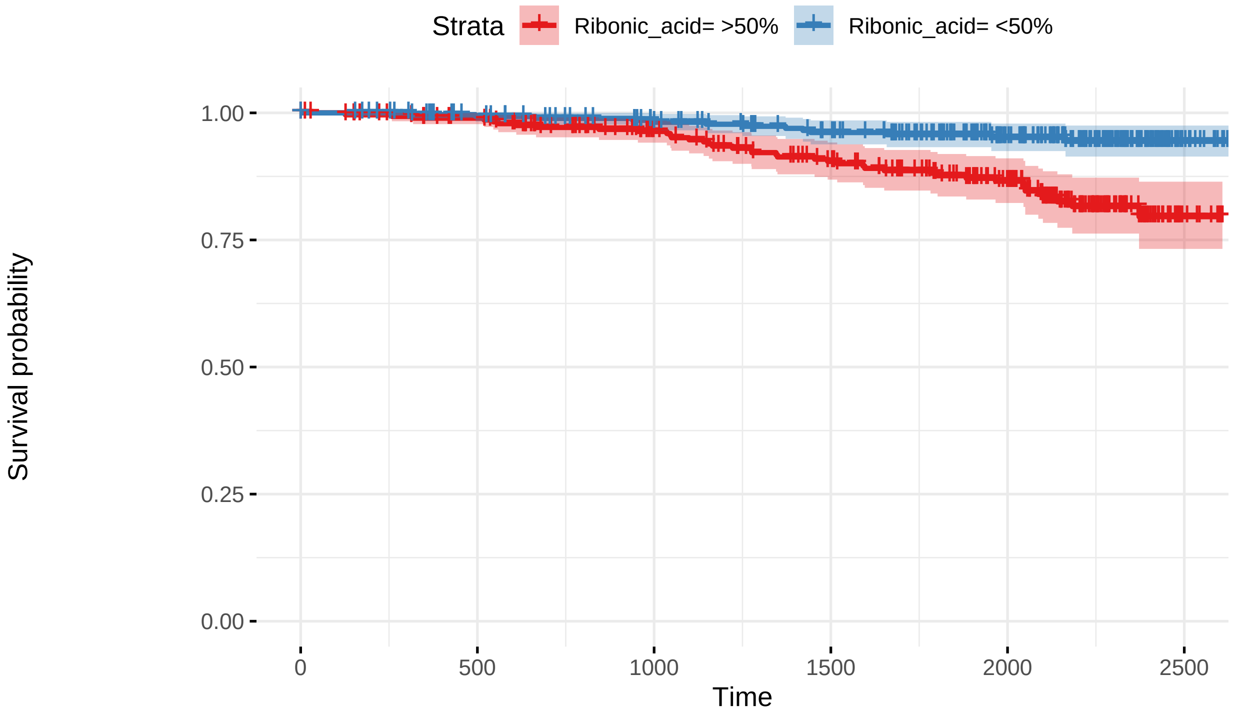

Number at risk

Ribonic_acid= >50%	318	276	236	203	149	10
Ribonic_acid= <50%	319	275	254	232	161	18

Cumulative number of events

Ribonic_acid= >50%	0	3	10	24	32	40
Ribonic_acid= <50%	0	2	5	10	12	13

5.3.2.2 Step 4.2: Second Top-Metabolite in Relation to eGFR Decline (> 40 %)

5.3.2.2.1 Step 4.2A: Analysis of Full Cohort

Survival Model with Details

```
## Call:
## survival::coxph(formula = survival::Surv(time = t_gfrfald40_p,
##     event = censor_gfrfald40_p.reversed.numeric) ~ Myo_Inositol +
##     Age + BMI + BP_Systolic + Cholesterol + eGFR + HbA1c + Medication_Statins +
##     Sex + Smoking + TG_total_log + UAER_log, data = data.survival)
##
##   n= 586, number of events= 50
##    (51 observations deleted due to missingness)
##
##                         coef exp(coef)  se(coef)      z Pr(>|z|)
## Myo_Inositol        0.950735  2.587612  0.318197  2.988  0.00281 **
## Age                -0.023959  0.976325  0.016912 -1.417  0.15656
## BMI                -0.045257  0.955751  0.040694 -1.112  0.26608
## BP_Systolic         0.025781  1.026116  0.008505  3.031  0.00243 **
## Cholesterol         0.057771  1.059472  0.167717  0.344  0.73050
## eGFR               -0.008329  0.991706  0.006868 -1.213  0.22527
## HbA1c               0.339497  1.404241  0.120699  2.813  0.00491 **
## Medication_Statins  0.622658  1.863876  0.420560  1.481  0.13873
## Sex                 0.453594  1.573959  0.326691  1.388  0.16500
## Smoking             0.440837  1.554008  0.349995  1.260  0.20783
## TG_total_log       -0.222902  0.800193  0.214573 -1.039  0.29889
## UAER_log            0.297606  1.346632  0.070272  4.235 2.28e-05 ***
## ---
## Signif. codes:  0 '***' 0.001 '**' 0.01 '*' 0.05 '.' 0.1 ' ' 1
##
##                    exp(coef) exp(-coef) lower .95 upper .95
## Myo_Inositol          2.5876     0.3865    1.3869     4.828
## Age                   0.9763     1.0242    0.9445     1.009
## BMI                   0.9558     1.0463    0.8825     1.035
## BP_Systolic           1.0261     0.9745    1.0092     1.043
## Cholesterol           1.0595     0.9439    0.7627     1.472
## eGFR                  0.9917     1.0084    0.9784     1.005
## HbA1c                 1.4042     0.7121    1.1084     1.779
## Medication_Statins    1.8639     0.5365    0.8174     4.250
## Sex                   1.5740     0.6353    0.8297     2.986
## Smoking               1.5540     0.6435    0.7826     3.086
## TG_total_log          0.8002     1.2497    0.5255     1.219
## UAER_log              1.3466     0.7426    1.1734     1.545
##
## Concordance= 0.876  (se = 0.042 )
## Rsquare= 0.176   (max possible= 0.638 )
## Likelihood ratio test= 113.7  on 12 df,   p=0
## Wald test            = 108.2  on 12 df,   p=0
## Score (logrank) test = 148.8  on 12 df,   p=0
```

5.3.2.2.2 Forest Plot with Clinical Variables

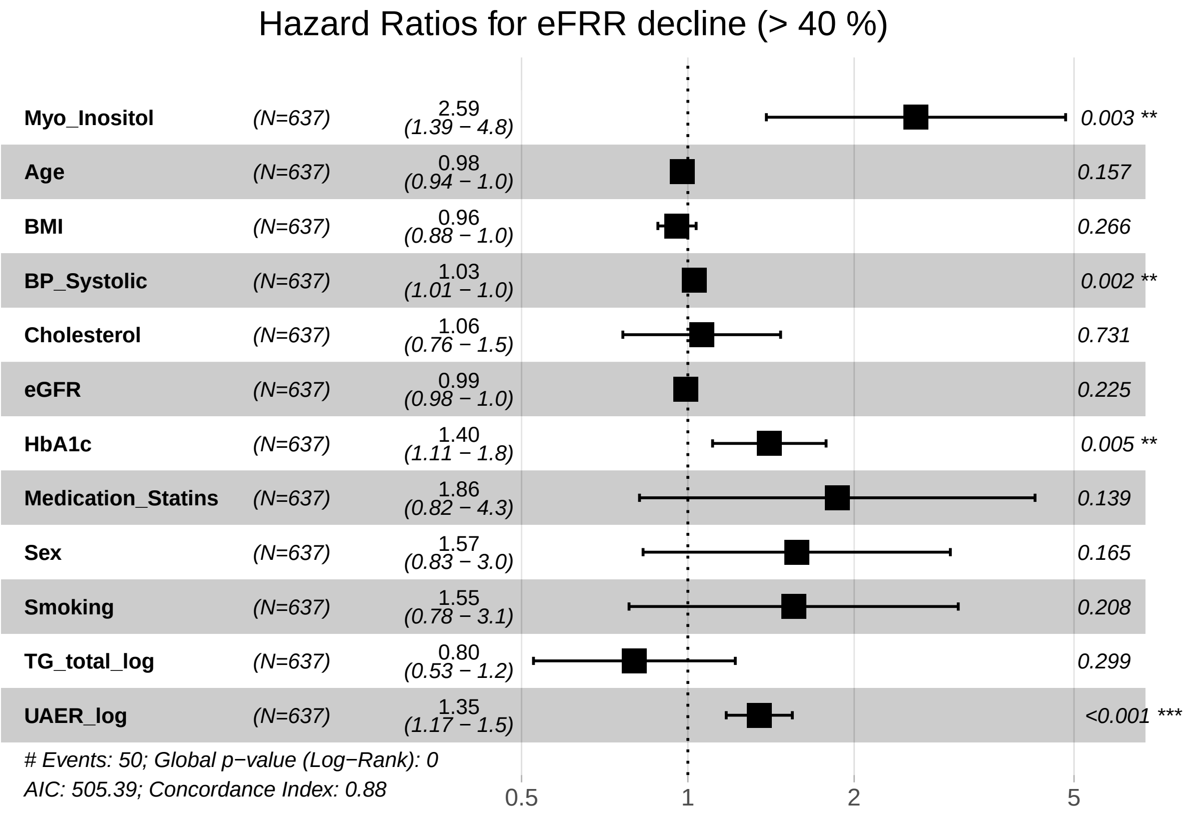

5.3.2.2.3 Kaplan-Maier Curve with Median Cutpoint

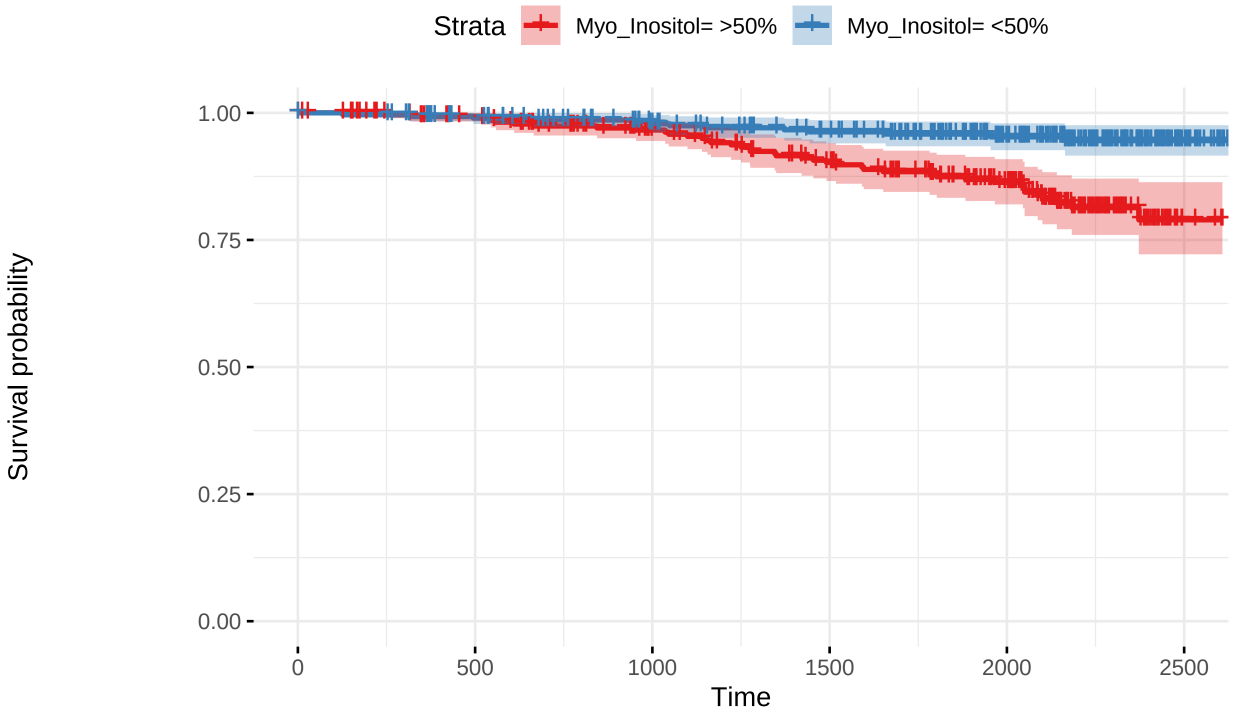

Number at risk

	0	500	1000	1500	2000	2500
Myo_Inositol= >50%	309	269	235	202	149	4
Myo_Inositol= <50%	328	282	255	233	161	24

Cumulative number of events

	0	500	1000	1500	2000	2500
Myo_Inositol= >50%	0	2	9	24	32	40
Myo_Inositol= <50%	0	3	6	10	12	13

5.3.2.3 Step 4.3: Third Top-Metabolite in Relation to eGFR Decline (> 40 %)

5.3.2.3.1 Step 4.3A: Analysis of Full Cohort

Survival Model with Details

```
## Call:
## survival::coxph(formula = survival::Surv(time = t_gfrfald40_p,
##     event = censor_gfrfald40_p.reversed.numeric) ~ Valine + Age +
##     BMI + BP_Systolic + Cholesterol + eGFR + HbA1c + Medication_Statins +
##     Sex + Smoking + TG_total_log + UAER_log, data = data.survival)
##
##   n= 586, number of events= 50
##    (51 observations deleted due to missingness)
##
##                         coef exp(coef)  se(coef)      z Pr(>|z|)
## Valine             -1.293743  0.274242  0.386614 -3.346 0.000819 ***
## Age                -0.020635  0.979577  0.016377 -1.260 0.207689
## BMI                -0.047474  0.953635  0.041257 -1.151 0.249862
## BP_Systolic         0.028158  1.028558  0.008513  3.307 0.000942 ***
## Cholesterol         0.032140  1.032662  0.163396  0.197 0.844063
## eGFR               -0.012859  0.987223  0.006292 -2.044 0.040989 *
## HbA1c               0.397169  1.487607  0.129431  3.069 0.002151 **
## Medication_Statins  0.614195  1.848169  0.414478  1.482 0.138380
## Sex                 0.719086  2.052555  0.338993  2.121 0.033902 *
## Smoking             0.261179  1.298460  0.352684  0.741 0.458968
## TG_total_log       -0.027911  0.972475  0.208268 -0.134 0.893390
## UAER_log            0.296194  1.344731  0.068809  4.305 1.67e-05 ***
## ---
## Signif. codes:  0 '***' 0.001 '**' 0.01 '*' 0.05 '.' 0.1 ' ' 1
##
##                    exp(coef) exp(-coef) lower .95 upper .95
## Valine                0.2742     3.6464    0.1285    0.5851
## Age                   0.9796     1.0208    0.9486    1.0115
## BMI                   0.9536     1.0486    0.8796    1.0340
## BP_Systolic           1.0286     0.9722    1.0115    1.0459
## Cholesterol           1.0327     0.9684    0.7497    1.4225
## eGFR                  0.9872     1.0129    0.9751    0.9995
## HbA1c                 1.4876     0.6722    1.1543    1.9172
## Medication_Statins    1.8482     0.5411    0.8202    4.1643
## Sex                   2.0526     0.4872    1.0562    3.9888
## Smoking               1.2985     0.7701    0.6505    2.5920
## TG_total_log          0.9725     1.0283    0.6465    1.4627
## UAER_log              1.3447     0.7436    1.1751    1.5389
##
## Concordance= 0.879  (se = 0.042 )
## Rsquare= 0.179   (max possible= 0.638 )
## Likelihood ratio test= 115.7  on 12 df,   p=0
## Wald test            = 108.4  on 12 df,   p=0
## Score (logrank) test = 152.1  on 12 df,   p=0
```

5.3.2.3.2 Forest Plot with Clinical Variables

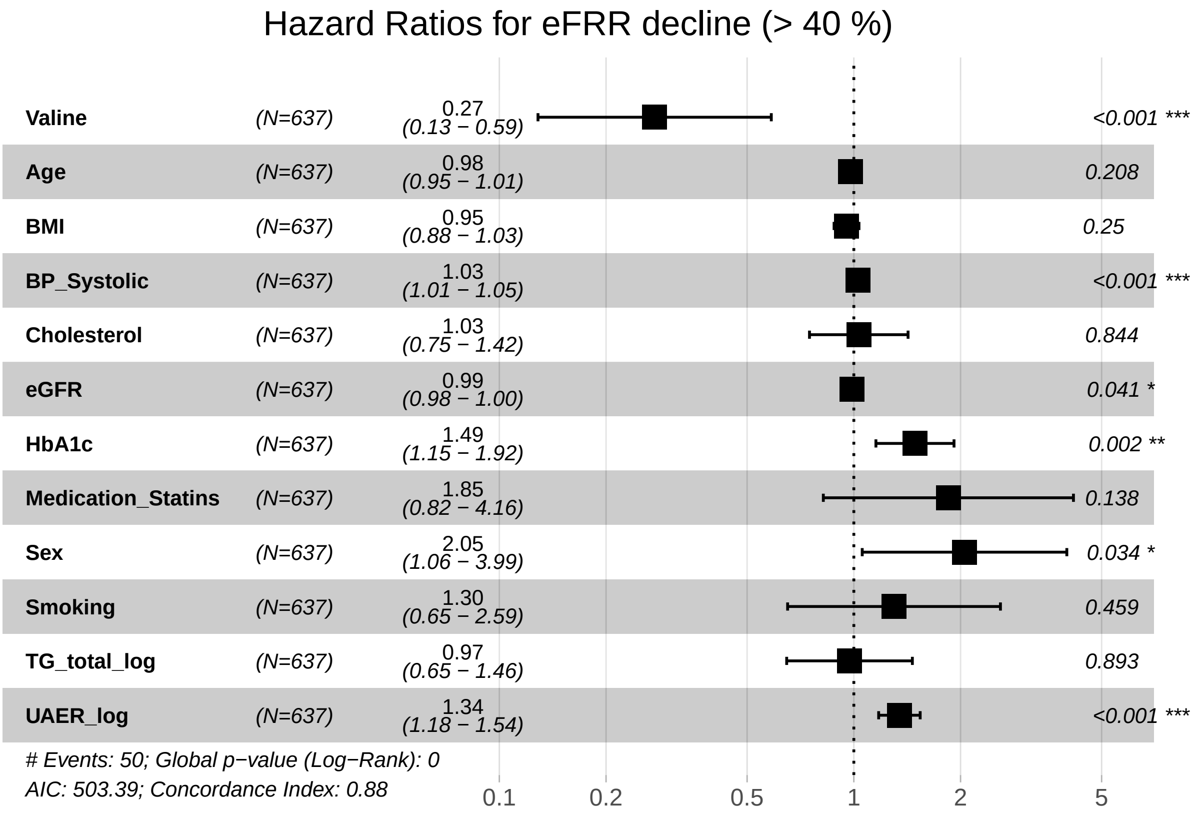

5.3.2.3.3 Kaplan-Maier Curve with Median Cutpoint

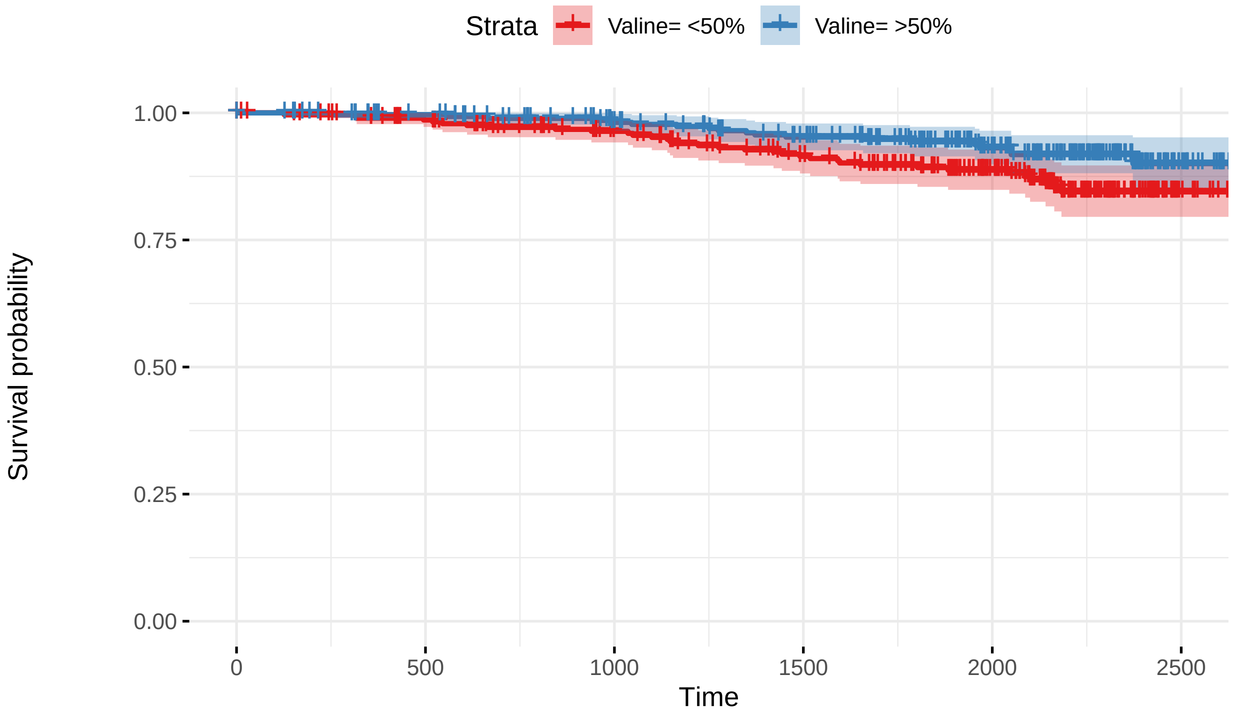

Number at risk

	0	500	1000	1500	2000	2500
Valine= <50%	319	275	243	213	158	10
Valine= >50%	318	276	247	222	152	18

Cumulative number of events

	0	500	1000	1500	2000	2500
Valine= <50%	0	4	10	22	28	34
Valine= >50%	0	1	5	12	16	19

6 Sample Size Calculation for Combined Renal Endpoint

6.0.3 Table

Table 19: Calculation of the number of samples needed for replicating the HRs of the crude survival model for the combined renal endpoint.

Name	HR	Pr(>|z|)	Power	N
3,4-Dihydroxybutanoic acid; 27	3.680	0.00e+00	0.8	96
Ribitol; 71	3.630	0.00e+00	0.8	98
Ribonic acid; 72	3.300	0.00e+00	0.8	115
Myo inositol 6TMS; 1	3.090	0.00e+00	0.8	128
2,4-Dihydroxybutanoic acid; 28	2.970	1.00e-07	0.8	138
4-Hydroxybenzeneacetic acid; 42	2.500	2.70e-06	0.8	194
Creatinine; 50	2.110	8.80e-05	0.8	293
Valine, 2TMS; 20	0.554	1.55e-03	0.8	467
Methionine, 2TMS; 16	0.570	2.59e-03	0.8	516
Isoleucine, 2TMS; 18	0.620	9.48e-03	0.8	712
Glyceryl-glycoside; 59	1.590	1.14e-02	0.8	753
2-Hydroxybutyric acid, 2TMS; 22	0.735	9.04e-02	NA	NA
Leucine, 2TMS; 19	0.753	1.19e-01	NA	NA
Fumaric acid, 2TMS; 9	1.330	1.20e-01	NA	NA

7 Boxplots

7.1 Entire Cohort

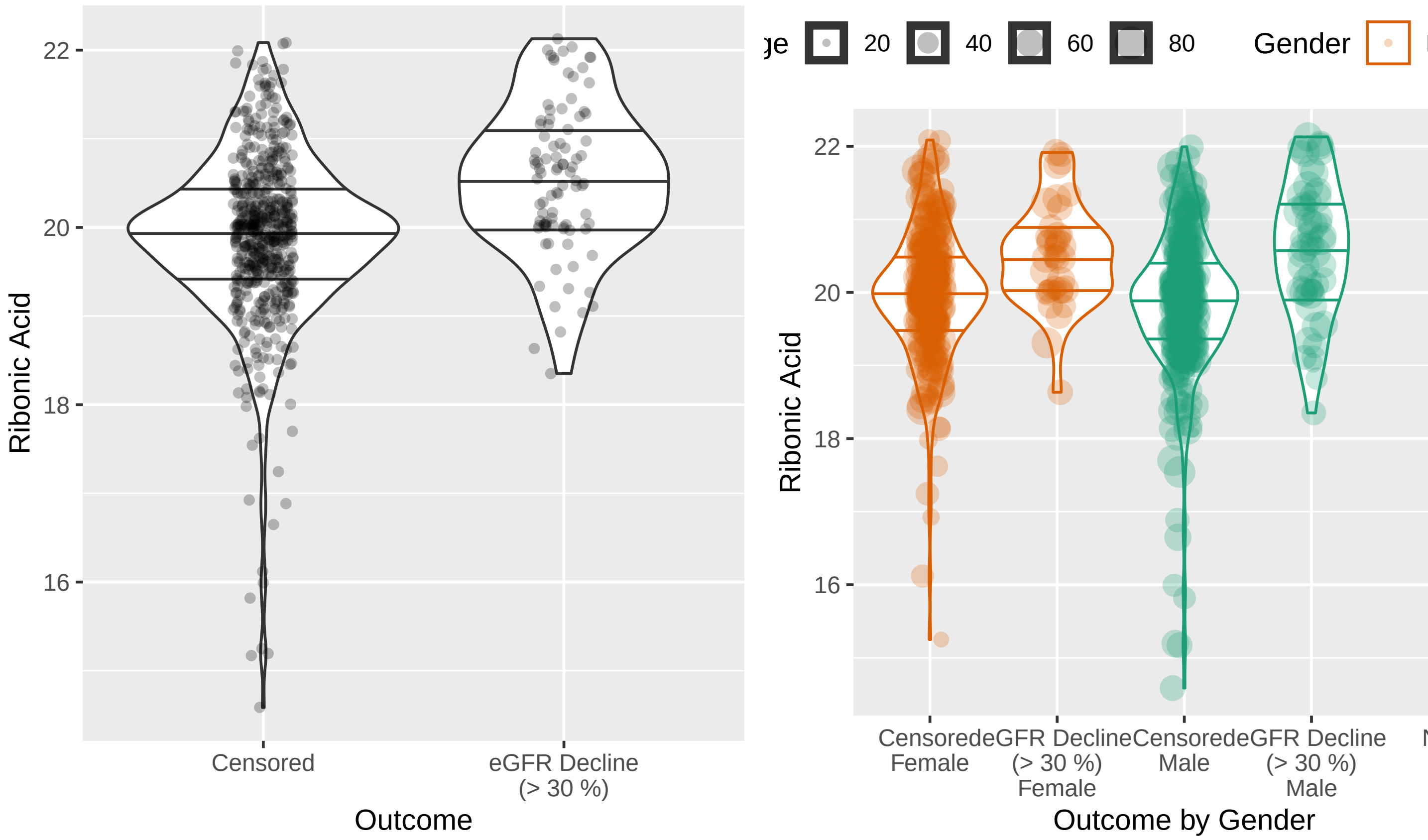

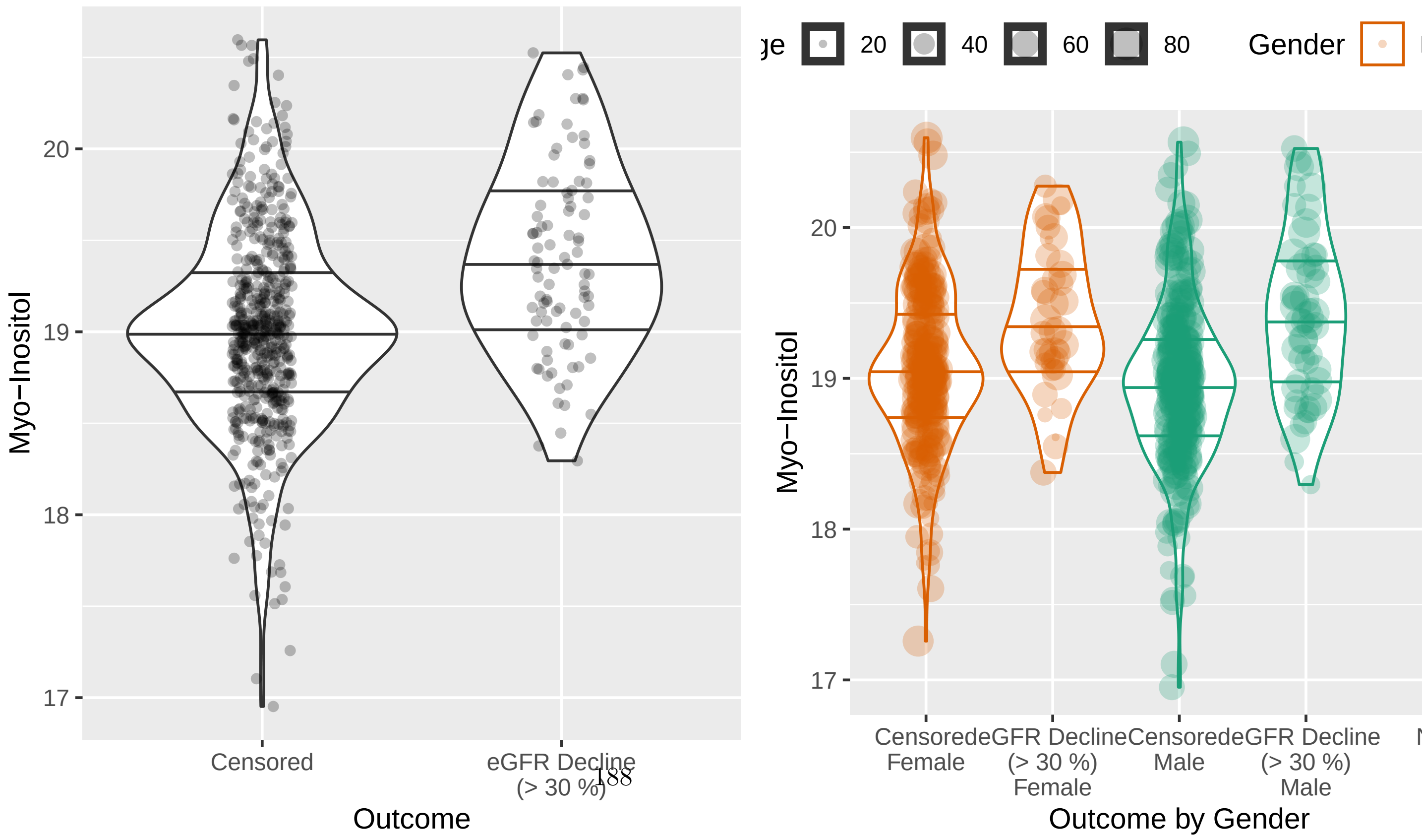

8 Correlation Matrix

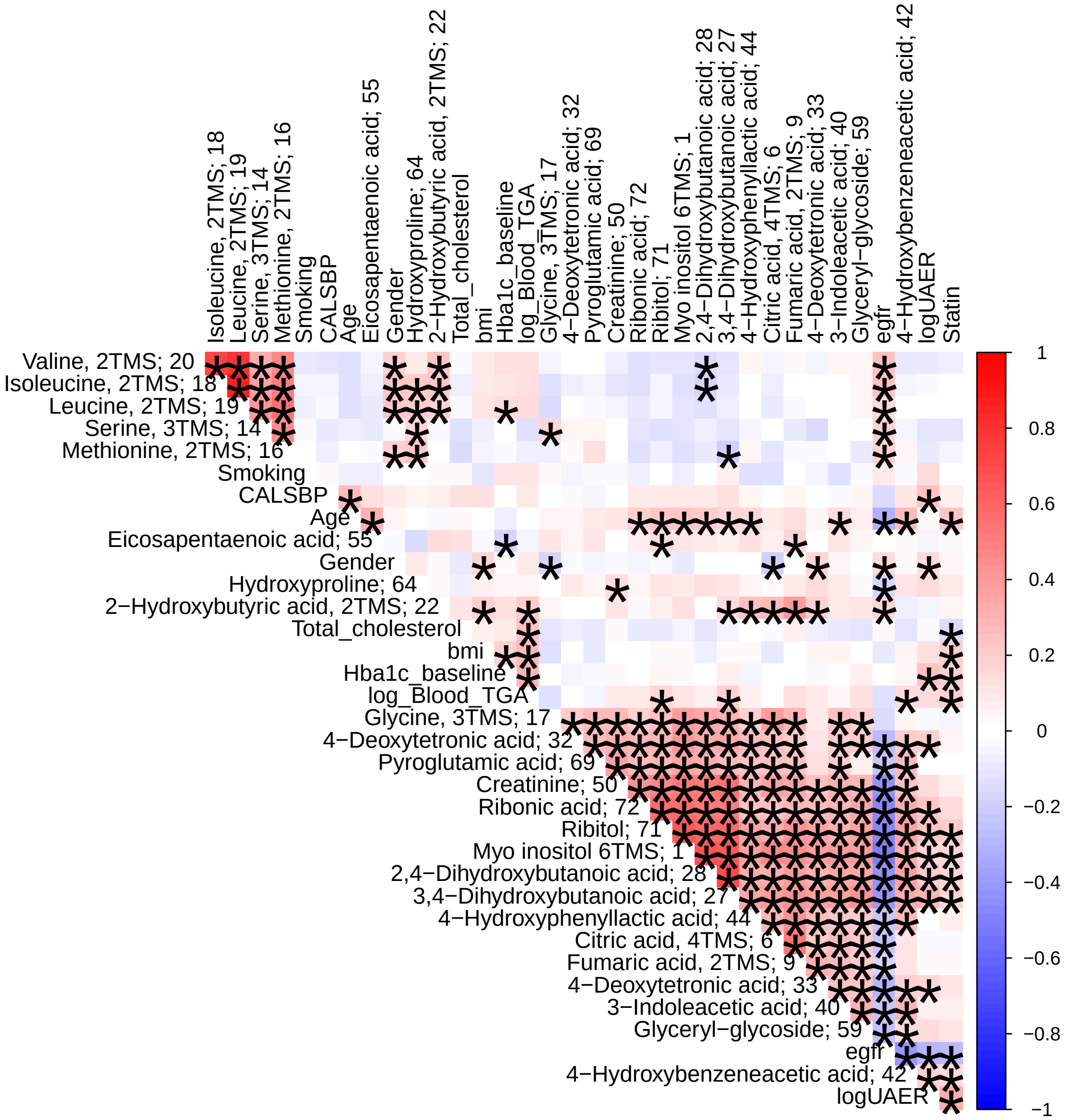

9 PCA

9.1 Groups

```
## Warning: package 'scales' was built under R version 3.4.4
```

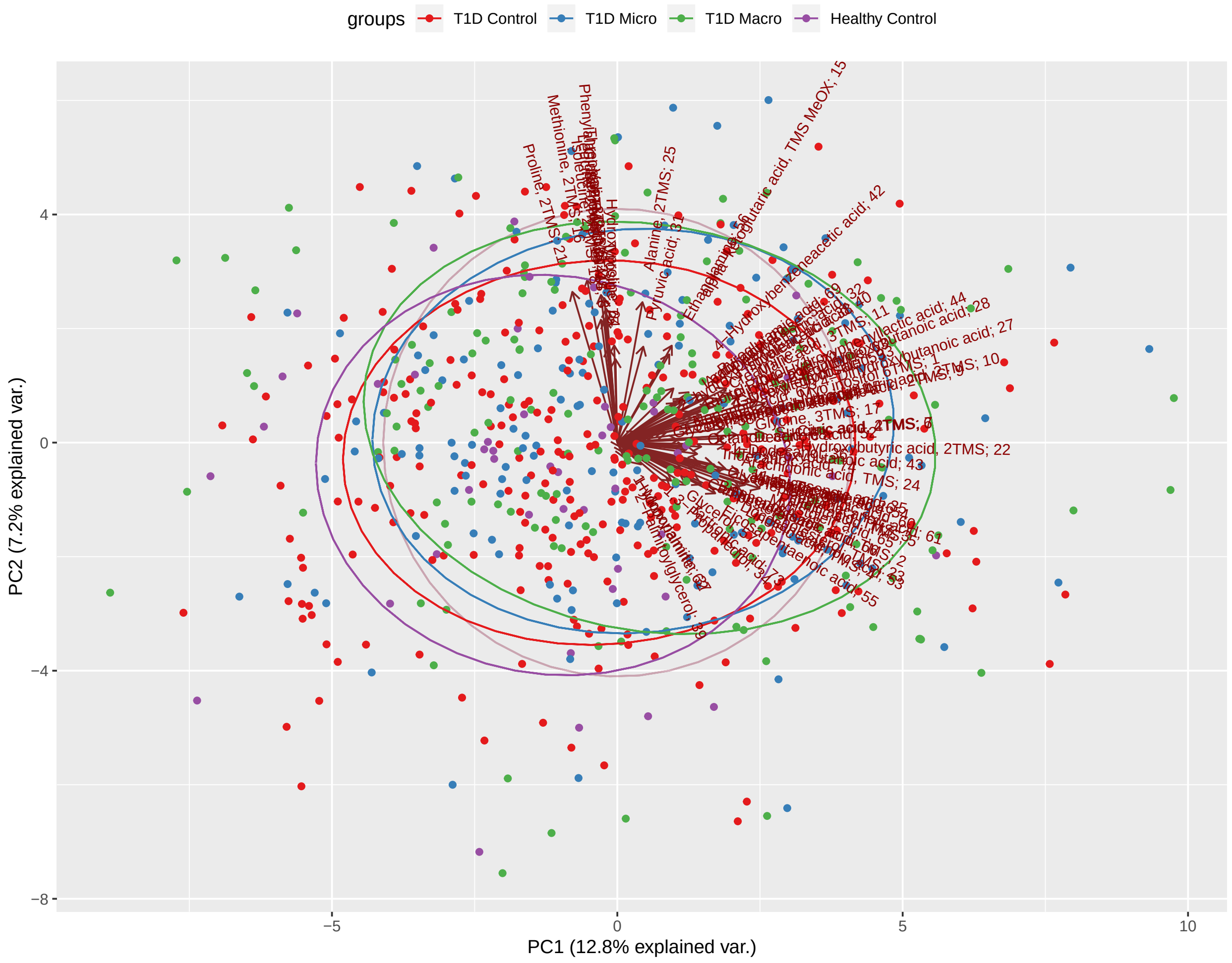

9.2 eGFR

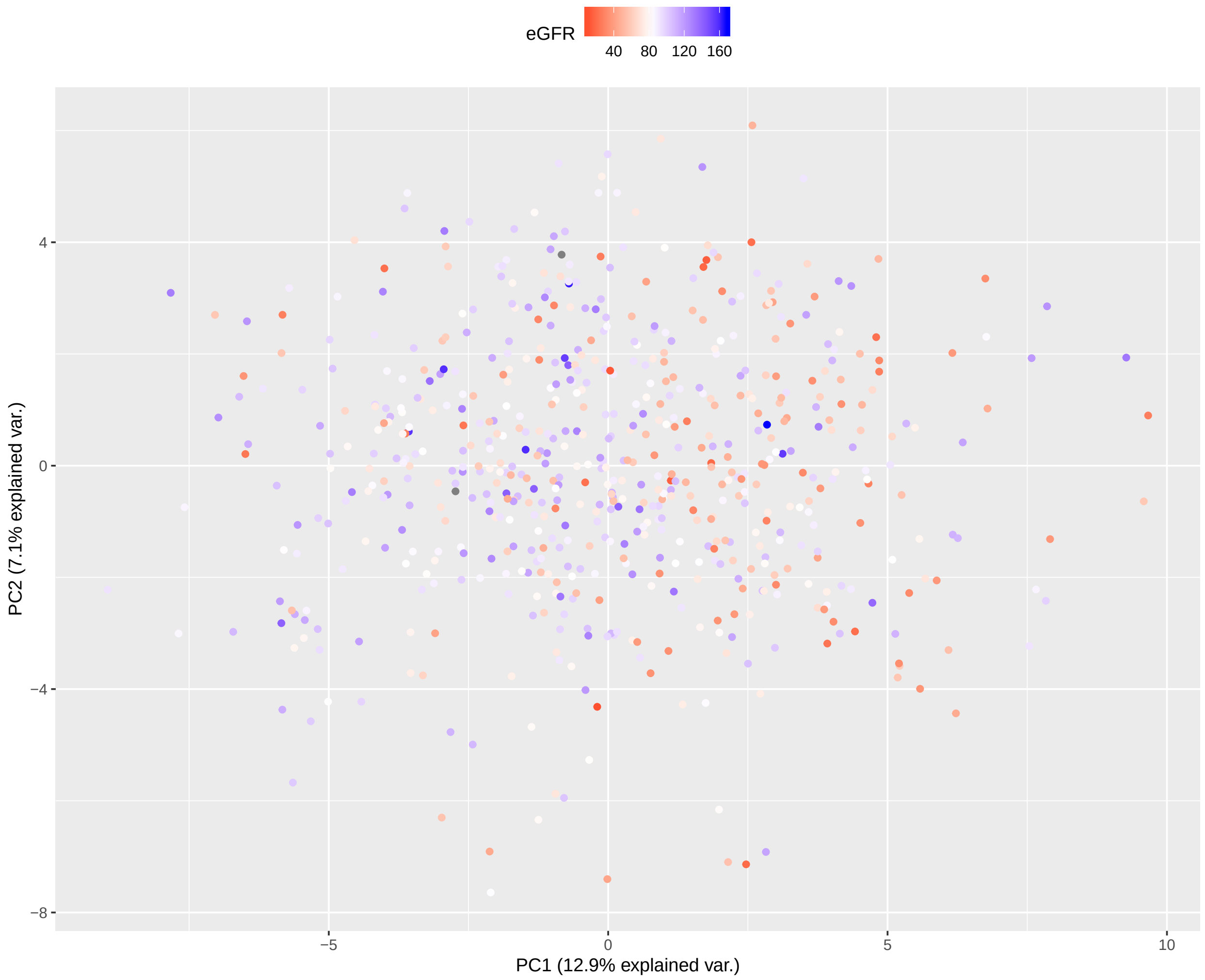

eGFR
40
80
120
160
4
0
−4
−8
PC2 (7.1% explained var.)
−5
0
5
10
PC1 (12.9% explained var.)

9.3 logUAER

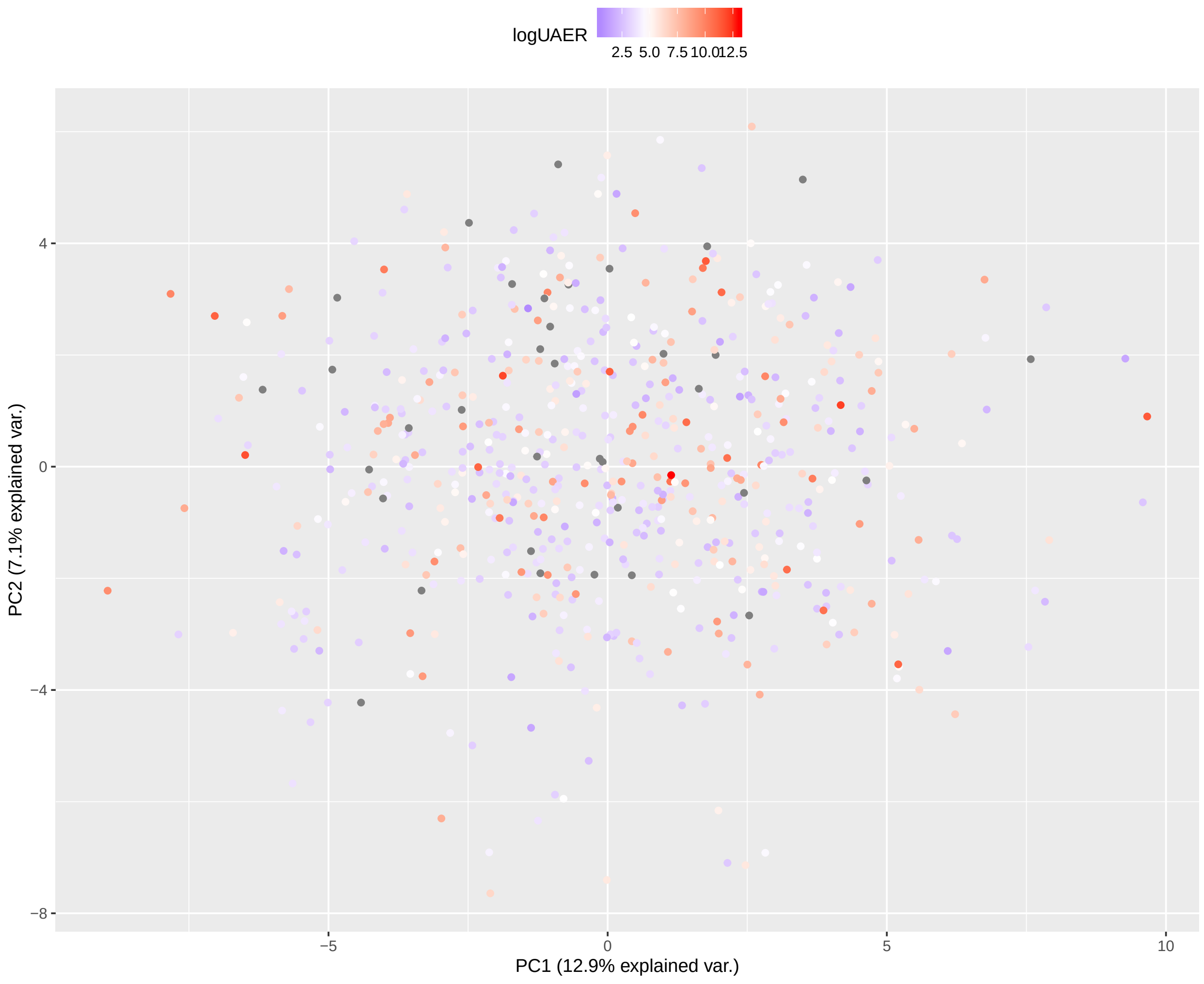
logUAER
2.5 5.0 7.5 10.0 12.5
4
0
−4
−8
PC2 (7.1% explained var.)
−5
0
5
10
PC1 (12.9% explained var.)

10 Scatter Plots

```
## `geom_smooth()` using method = 'loess' and formula 'y ~ x'
## Warning: Removed 2 rows containing non-finite values (stat_smooth).
## Warning: Removed 2 rows containing missing values (geom_point).
```

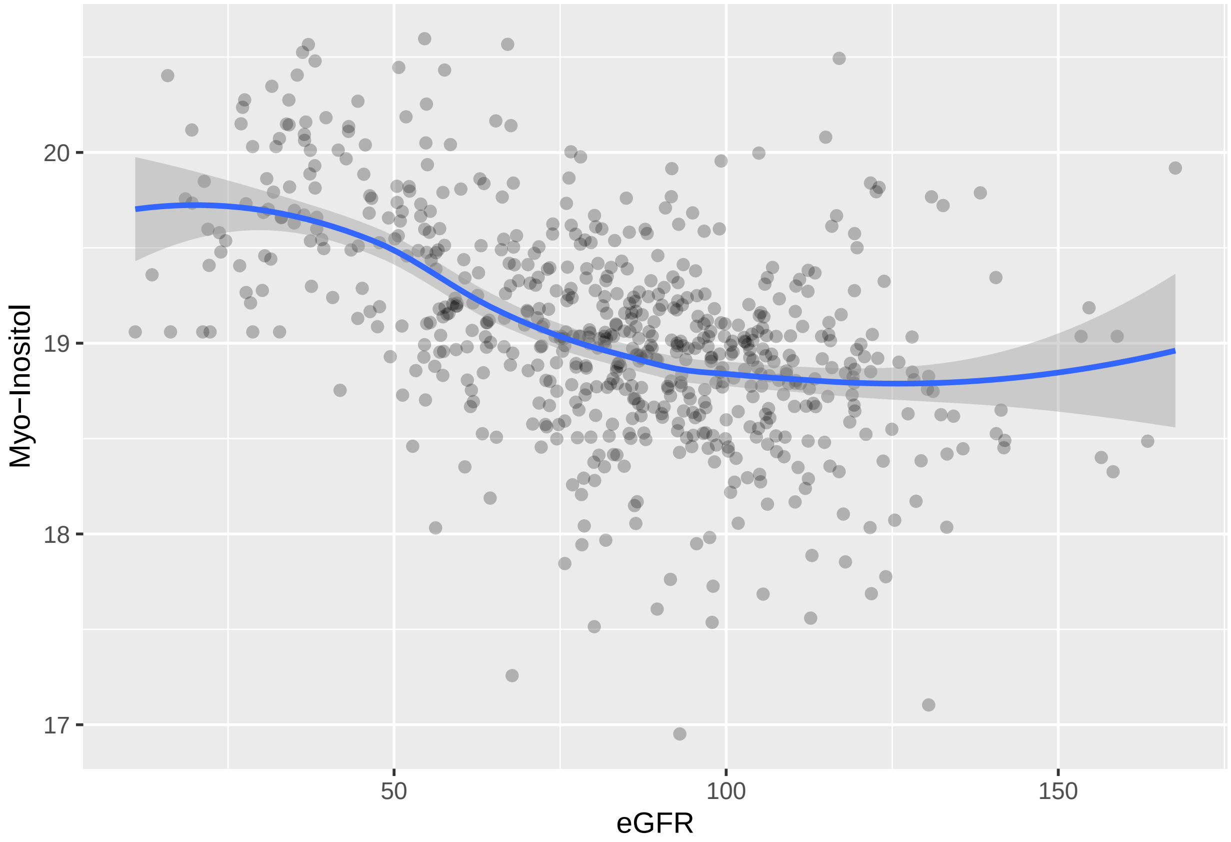


```
## `geom_smooth()` using method = 'loess' and formula 'y ~ x'
## Warning: Removed 2 rows containing non-finite values (stat_smooth).
## Warning: Removed 2 rows containing missing values (geom_point).
```

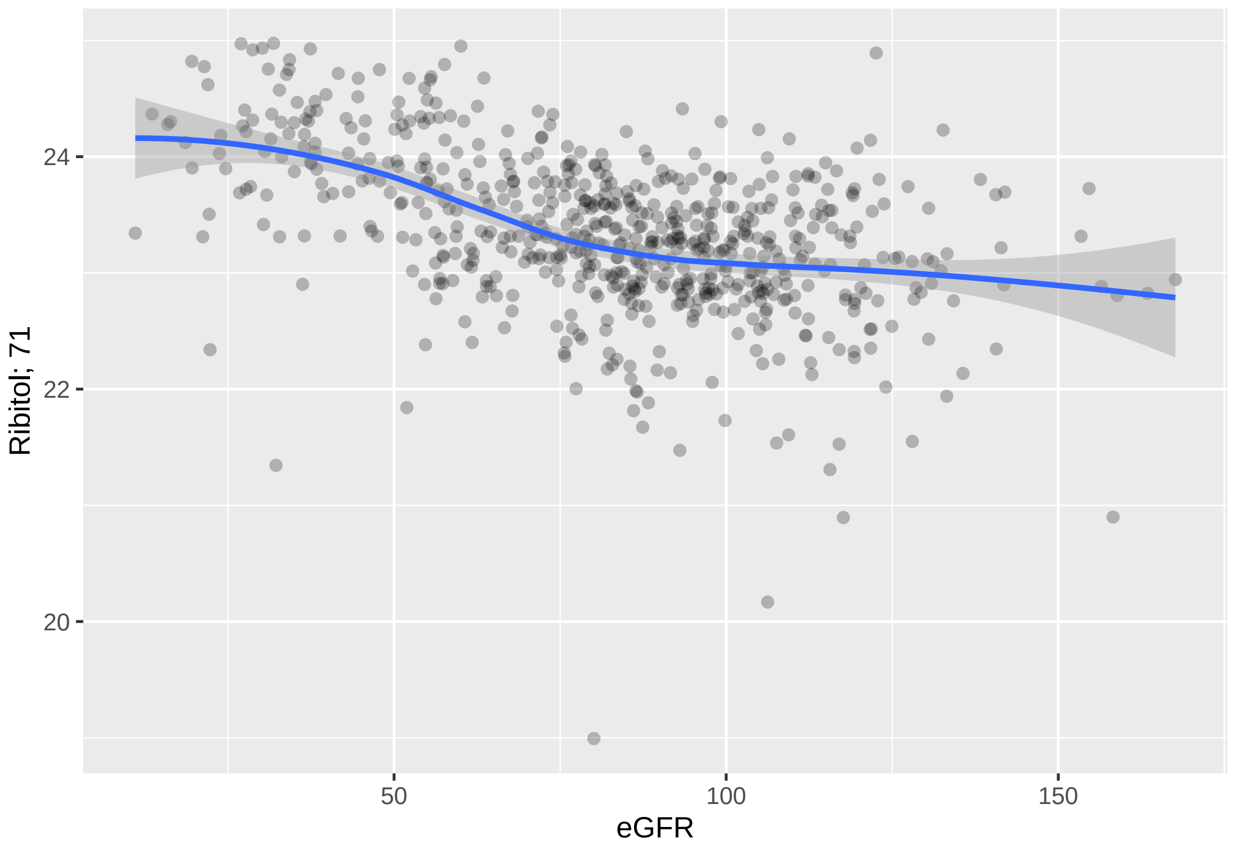


```
## `geom_smooth()` using method = 'loess' and formula 'y ~ x'

## Warning: Removed 2 rows containing non-finite values (stat_smooth).

## Warning: Removed 2 rows containing missing values (geom_point).
```

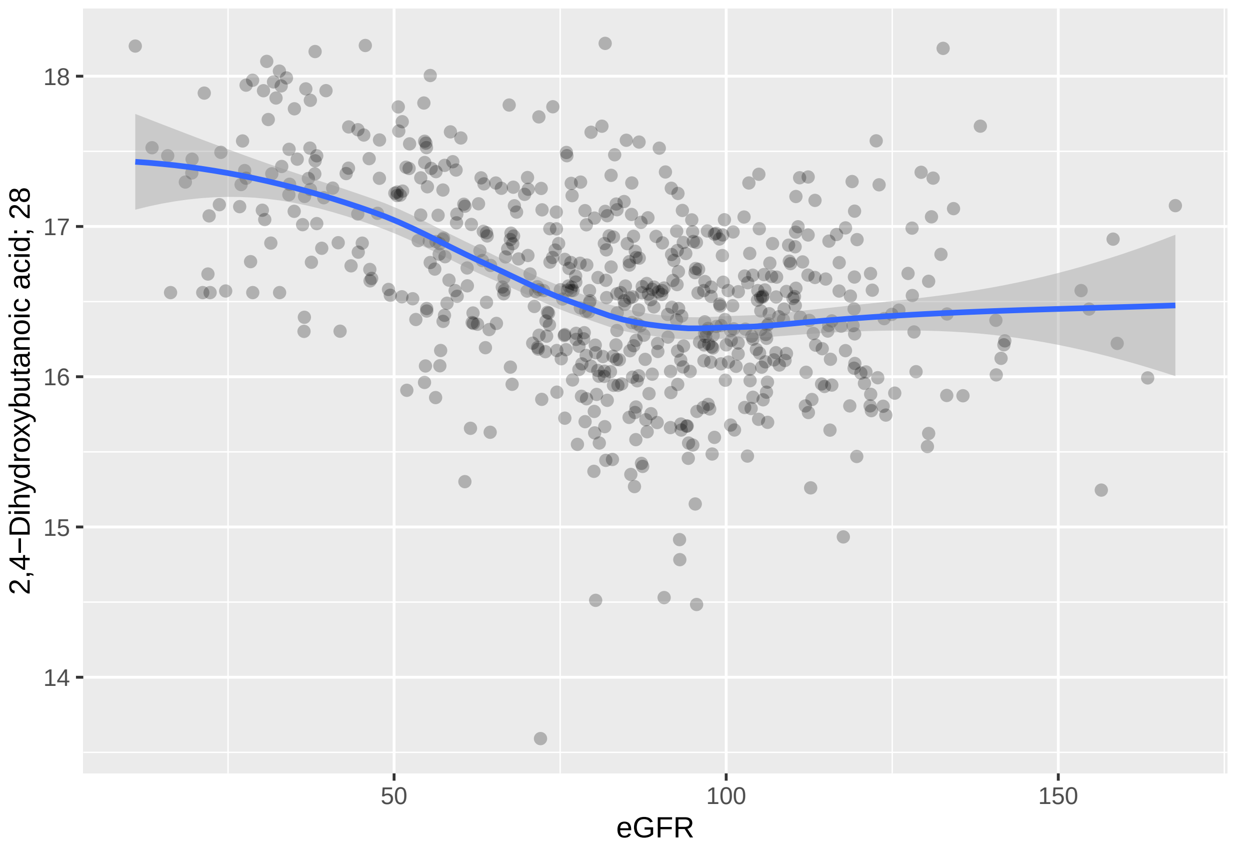


```
## `geom_smooth()` using method = 'loess' and formula 'y ~ x'
## Warning: Removed 2 rows containing non-finite values (stat_smooth).
## Warning: Removed 2 rows containing missing values (geom_point).
```

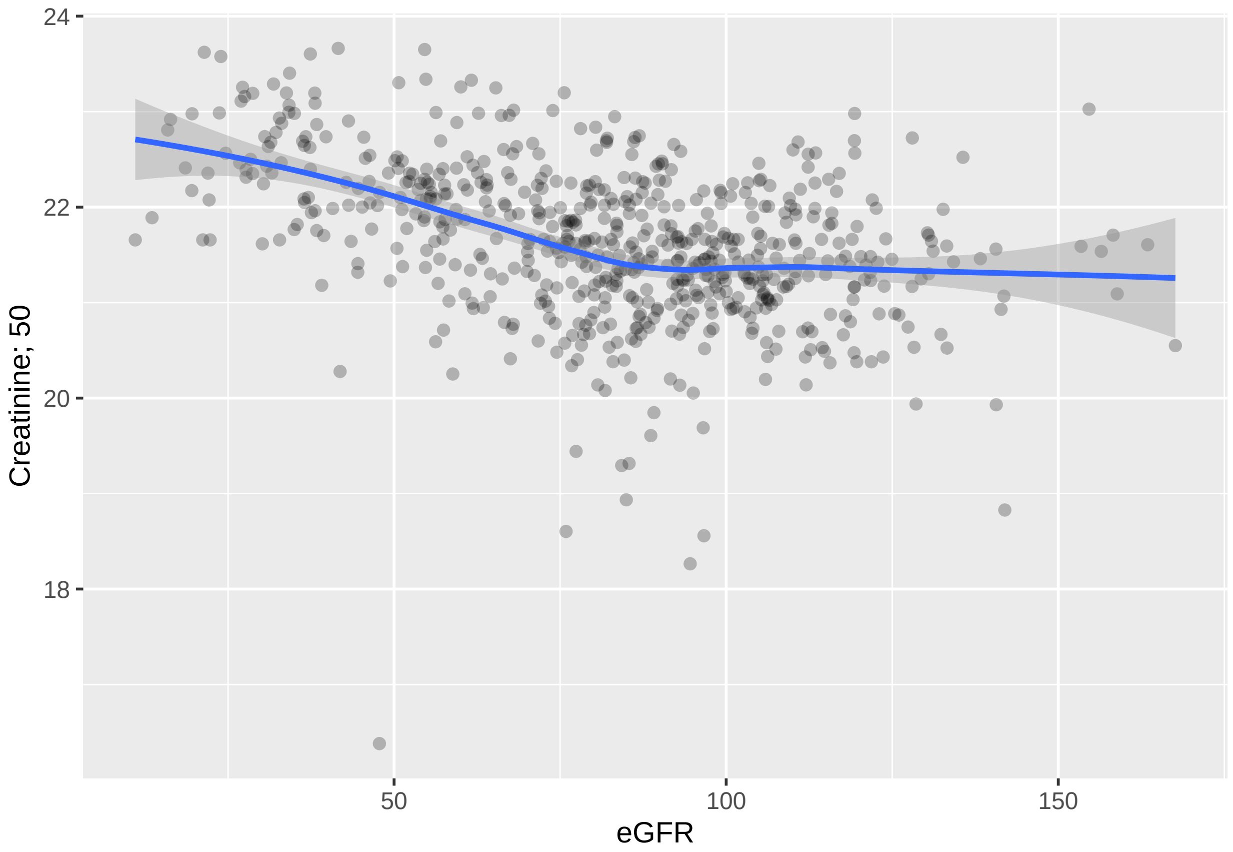


```
## `geom_smooth()` using method = 'loess' and formula 'y ~ x'
## Warning: Removed 2 rows containing non-finite values (stat_smooth).
## Warning: Removed 2 rows containing missing values (geom_point).
```

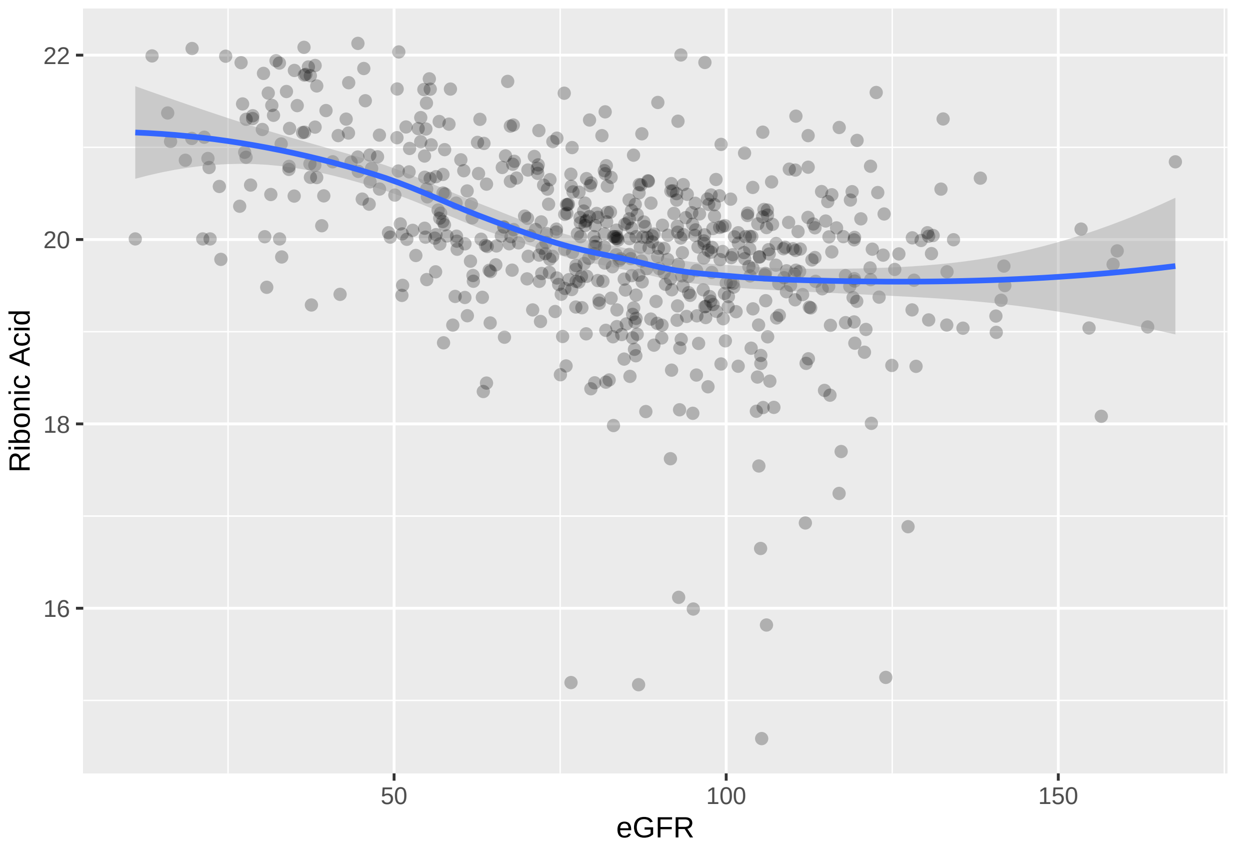


```
## `geom_smooth()` using method = 'loess' and formula 'y ~ x'
## Warning: Removed 2 rows containing non-finite values (stat_smooth).
## Warning: Removed 2 rows containing missing values (geom_point).
```

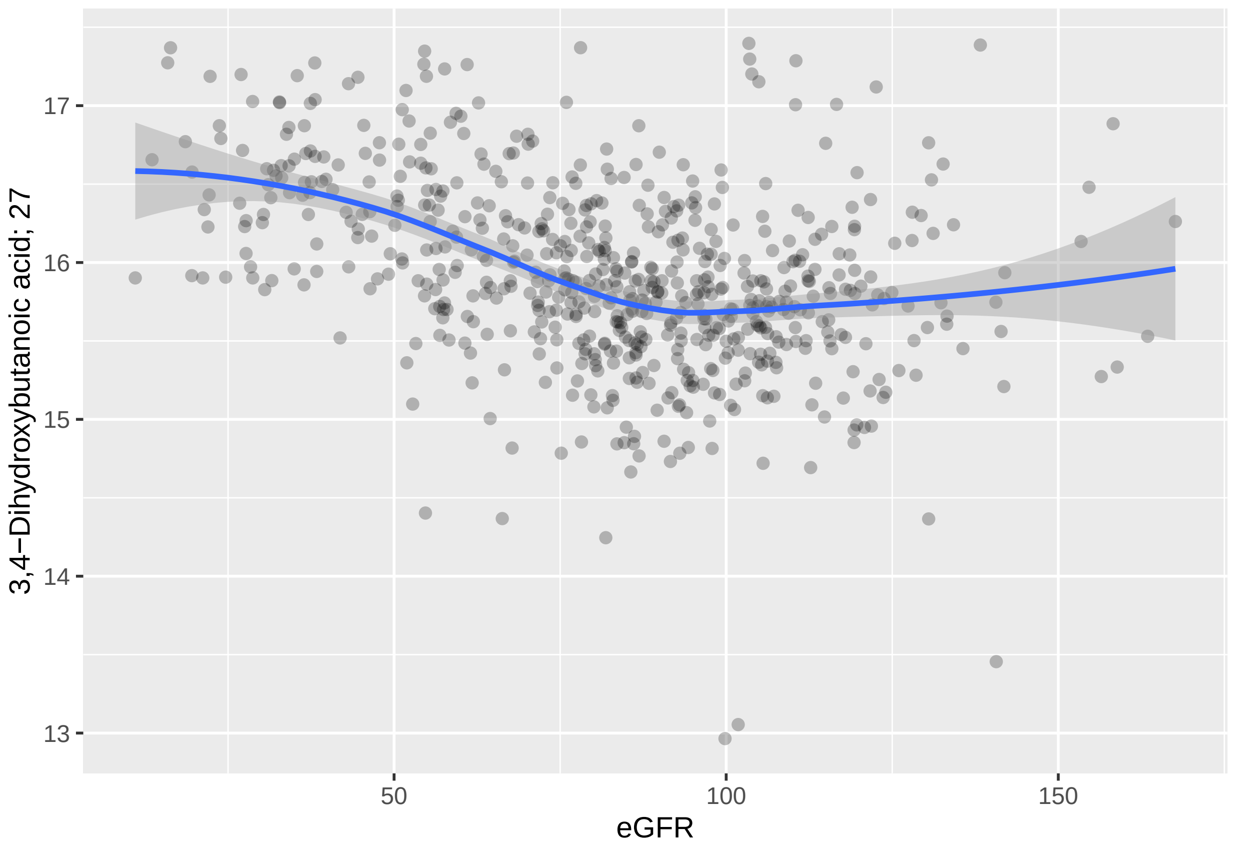


```
## `geom_smooth()` using method = 'loess' and formula 'y ~ x'
## Warning: Removed 2 rows containing non-finite values (stat_smooth).
## Warning: Removed 2 rows containing missing values (geom_point).
```

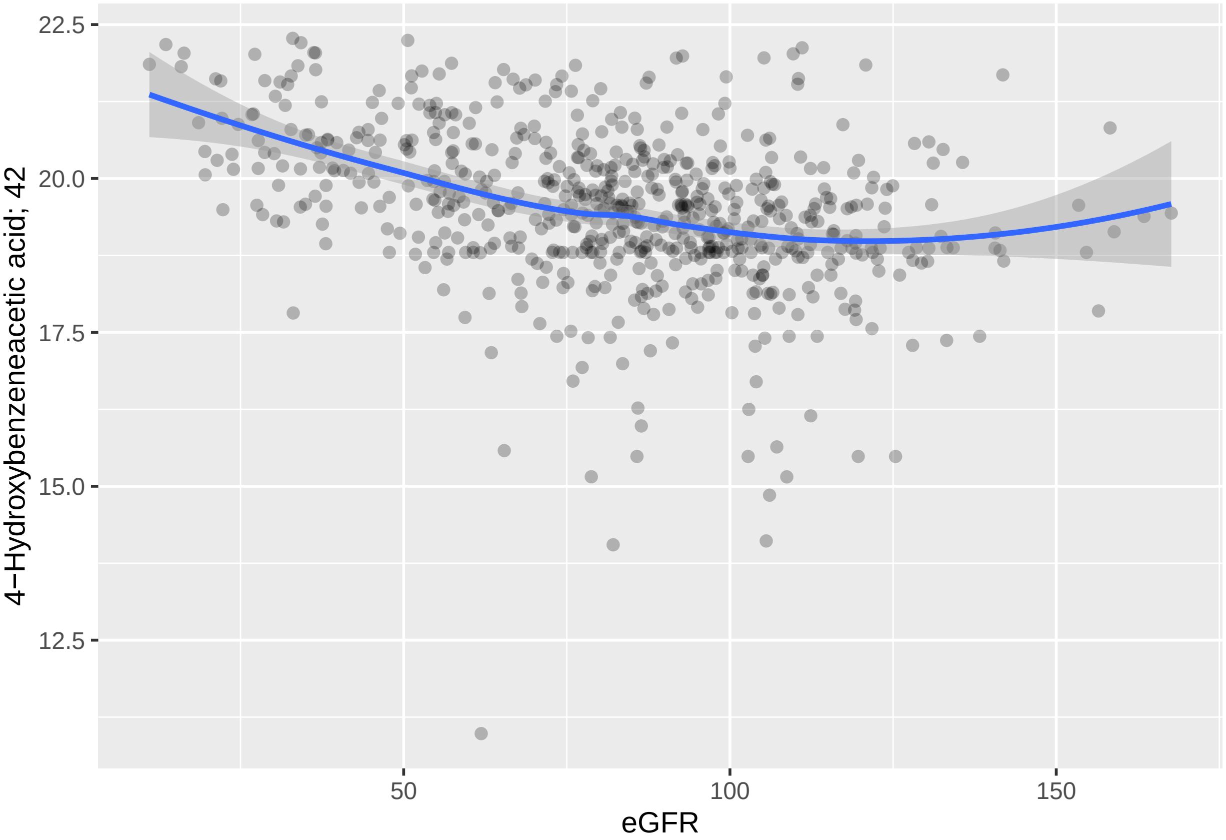


```
## `geom_smooth()` using method = 'loess' and formula 'y ~ x'
## Warning: Removed 2 rows containing non-finite values (stat_smooth).
## Warning: Removed 2 rows containing missing values (geom_point).
```

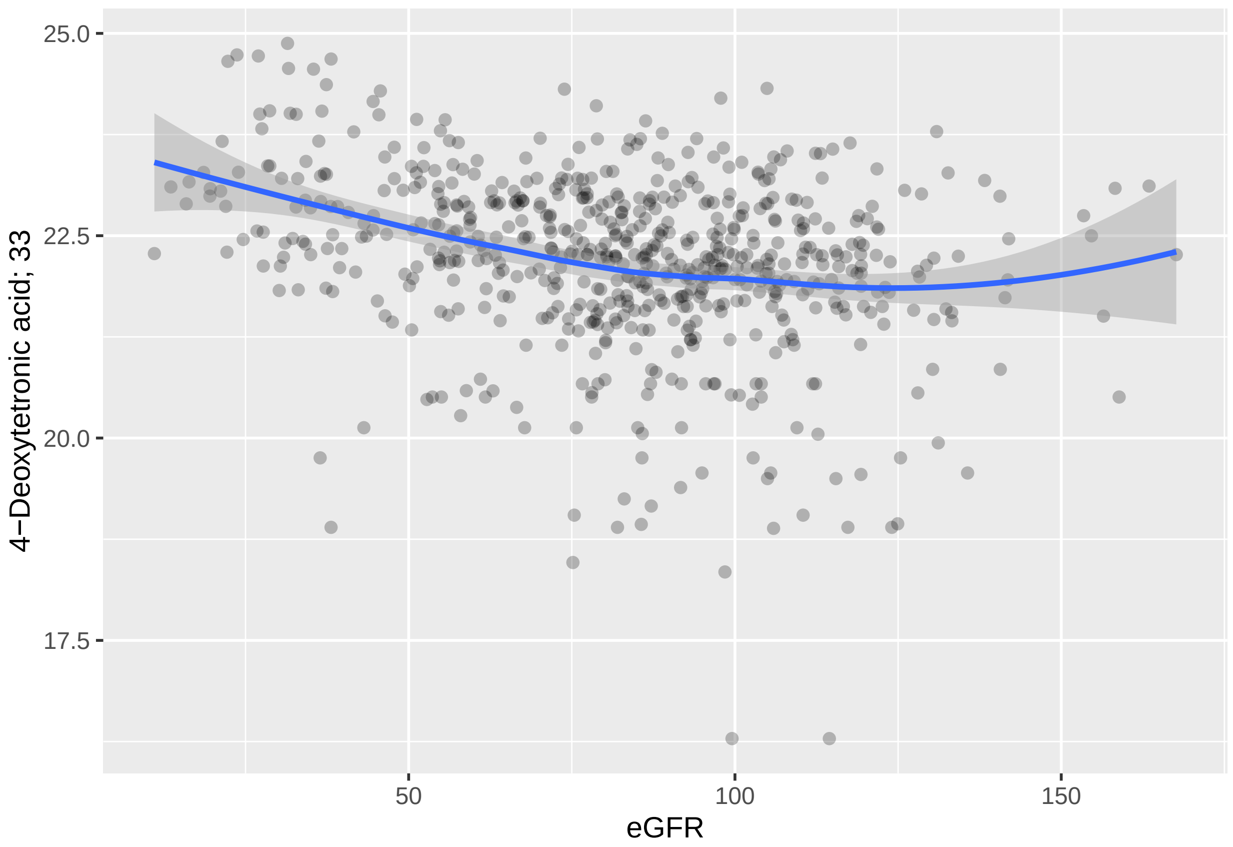


```
## `geom_smooth()` using method = 'loess' and formula 'y ~ x'

## Warning: Removed 2 rows containing non-finite values (stat_smooth).

## Warning: Removed 2 rows containing missing values (geom_point).
```

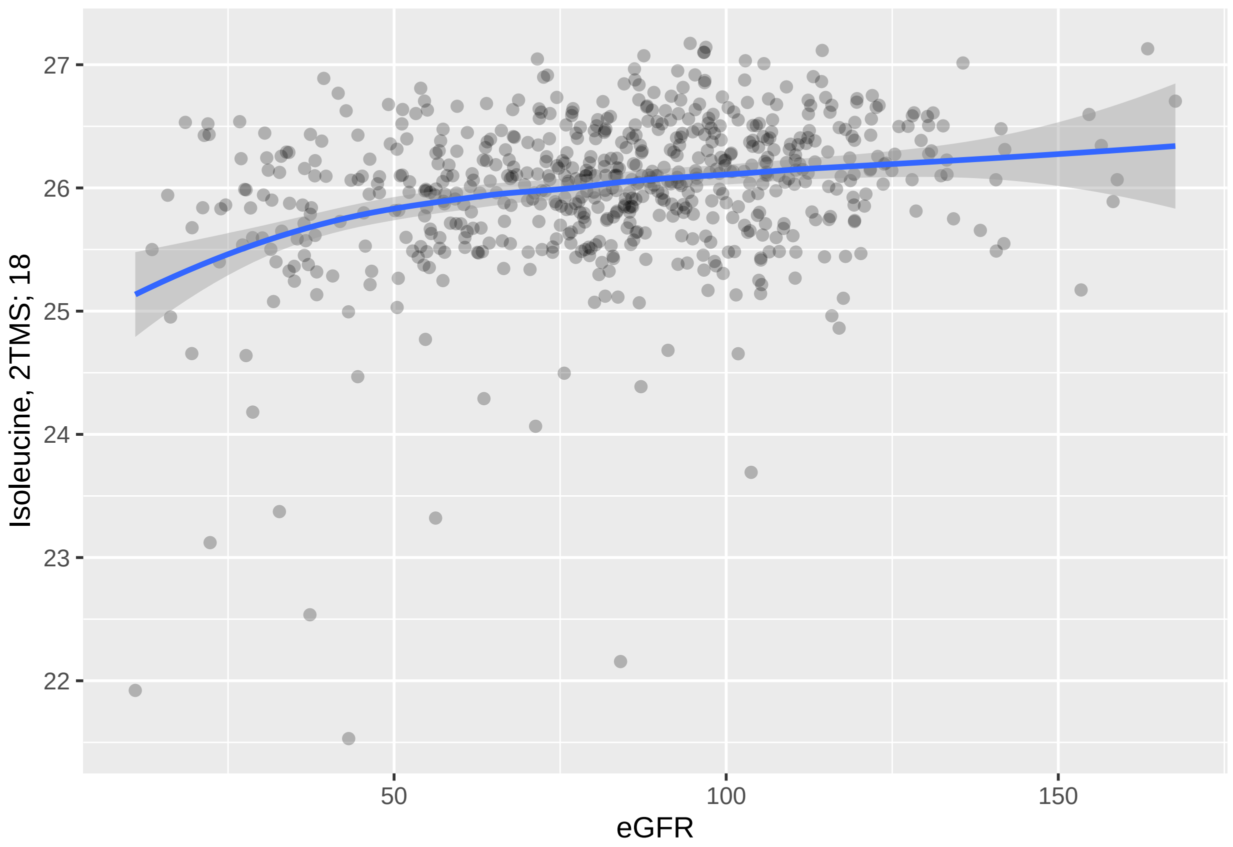


```
## `geom_smooth()` using method = 'loess' and formula 'y ~ x'

## Warning: Removed 2 rows containing non-finite values (stat_smooth).

## Warning: Removed 2 rows containing missing values (geom_point).
```

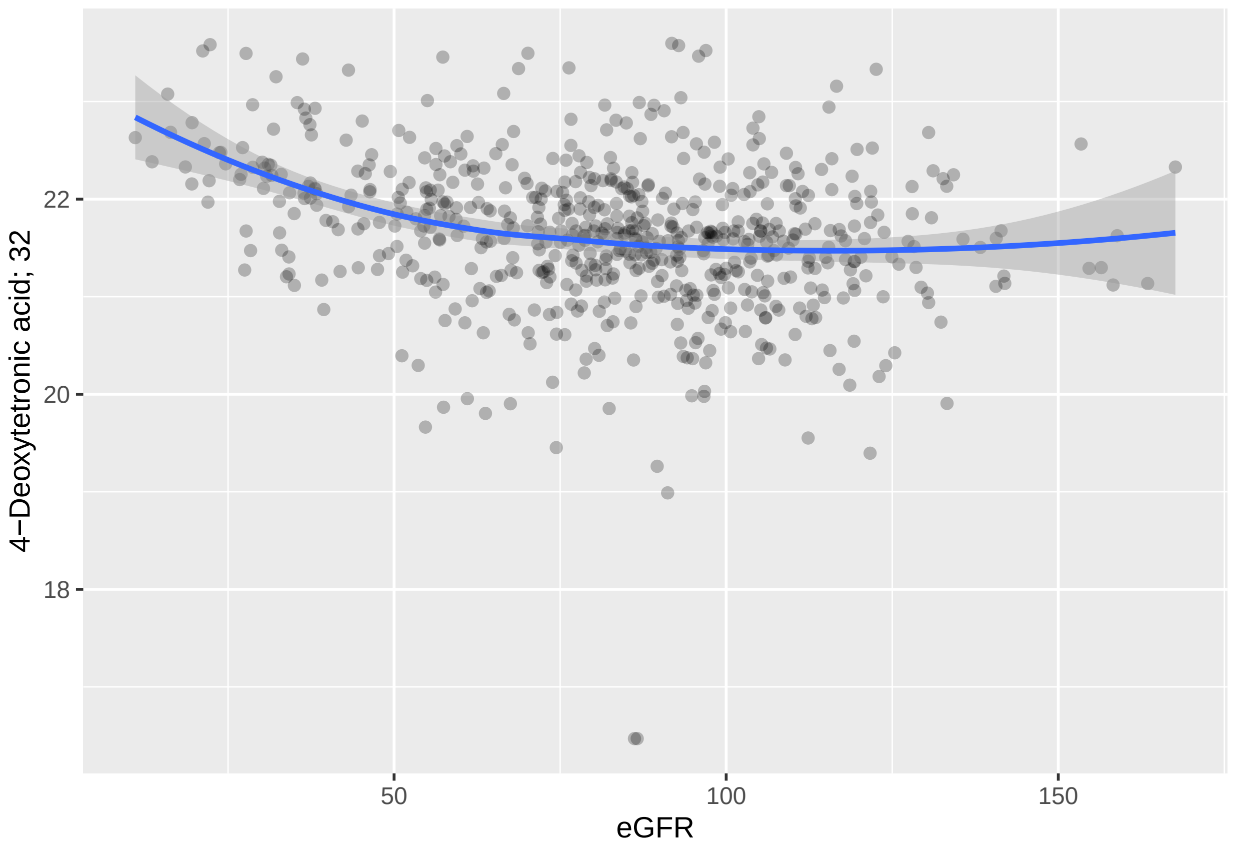


```
## `geom_smooth()` using method = 'loess' and formula 'y ~ x'
## Warning: Removed 2 rows containing non-finite values (stat_smooth).
## Warning: Removed 2 rows containing missing values (geom_point).
```

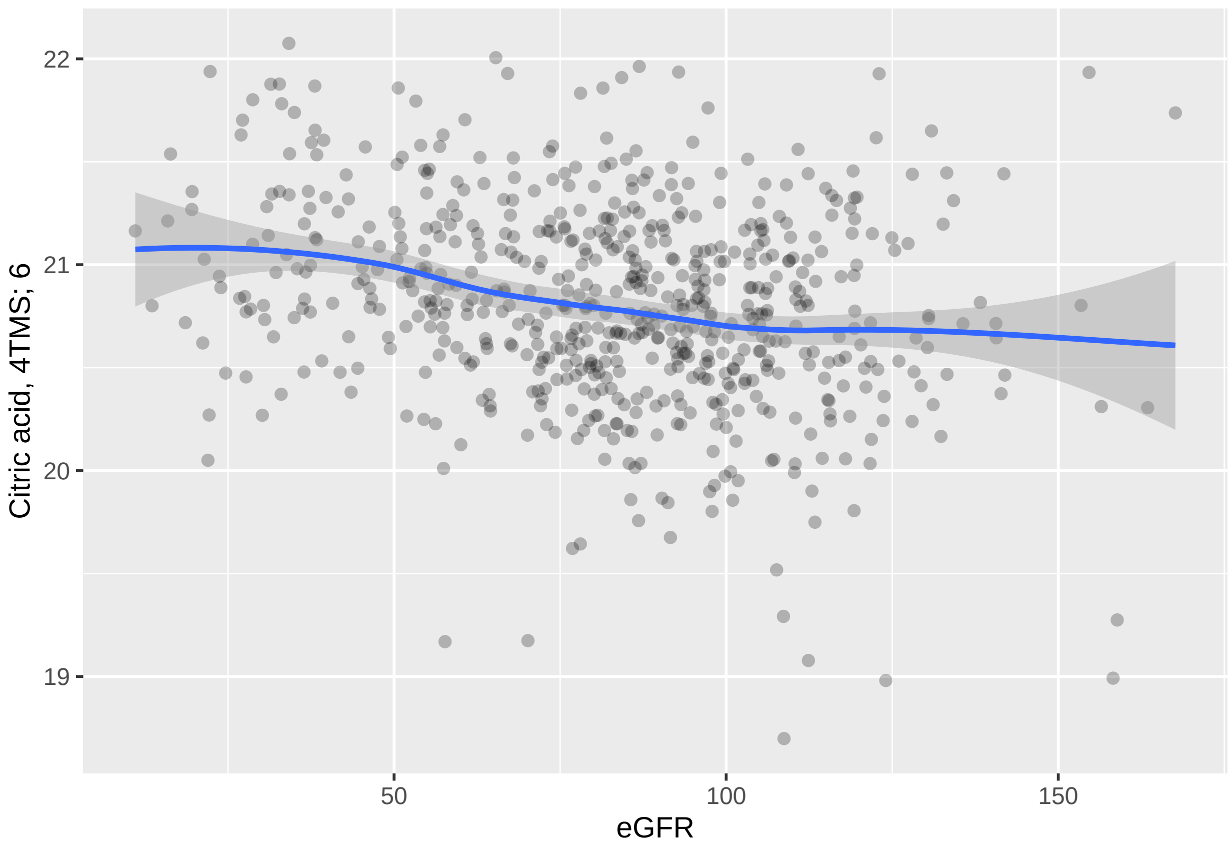


```
## `geom_smooth()` using method = 'loess' and formula 'y ~ x'

## Warning: Removed 2 rows containing non-finite values (stat_smooth).

## Warning: Removed 2 rows containing missing values (geom_point).
```

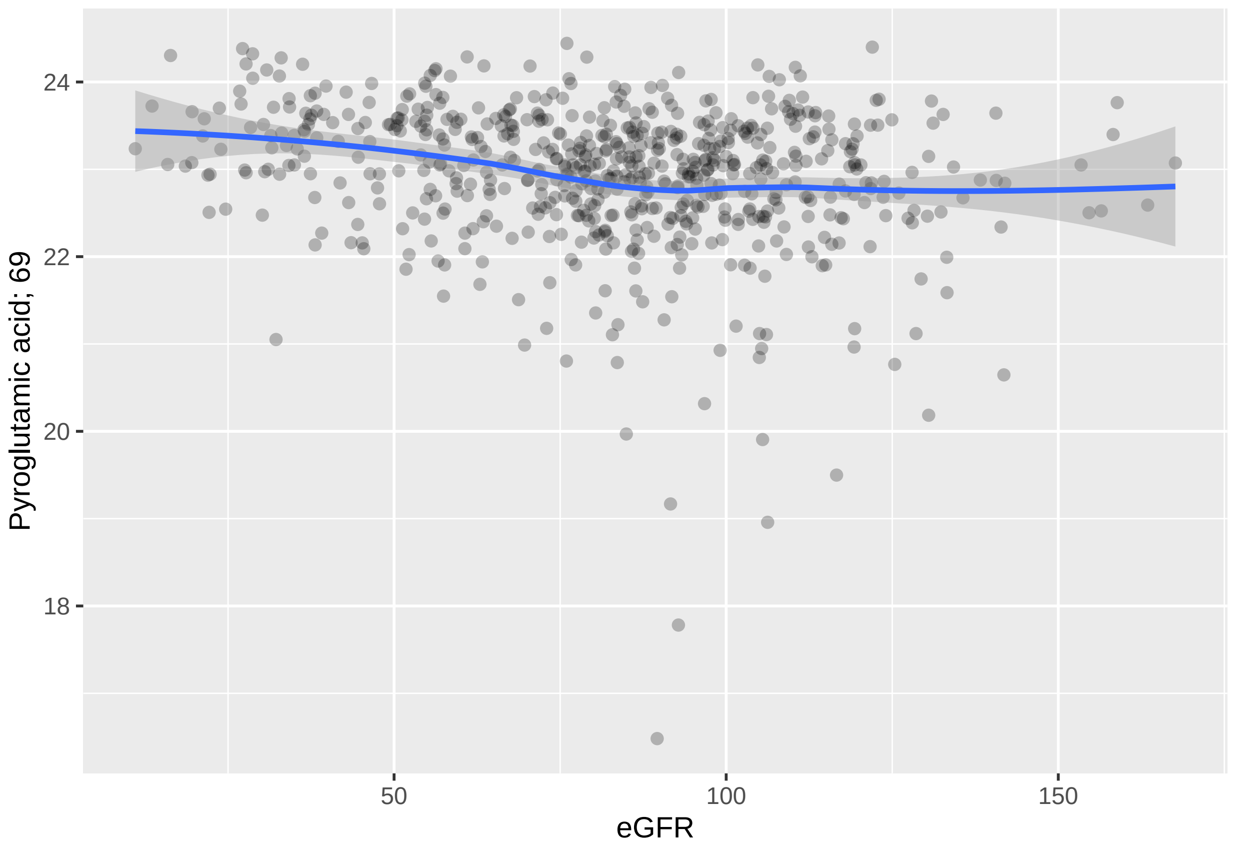


```
## `geom_smooth()` using method = 'loess' and formula 'y ~ x'
## Warning: Removed 2 rows containing non-finite values (stat_smooth).

## Warning: Removed 2 rows containing missing values (geom_point).
```

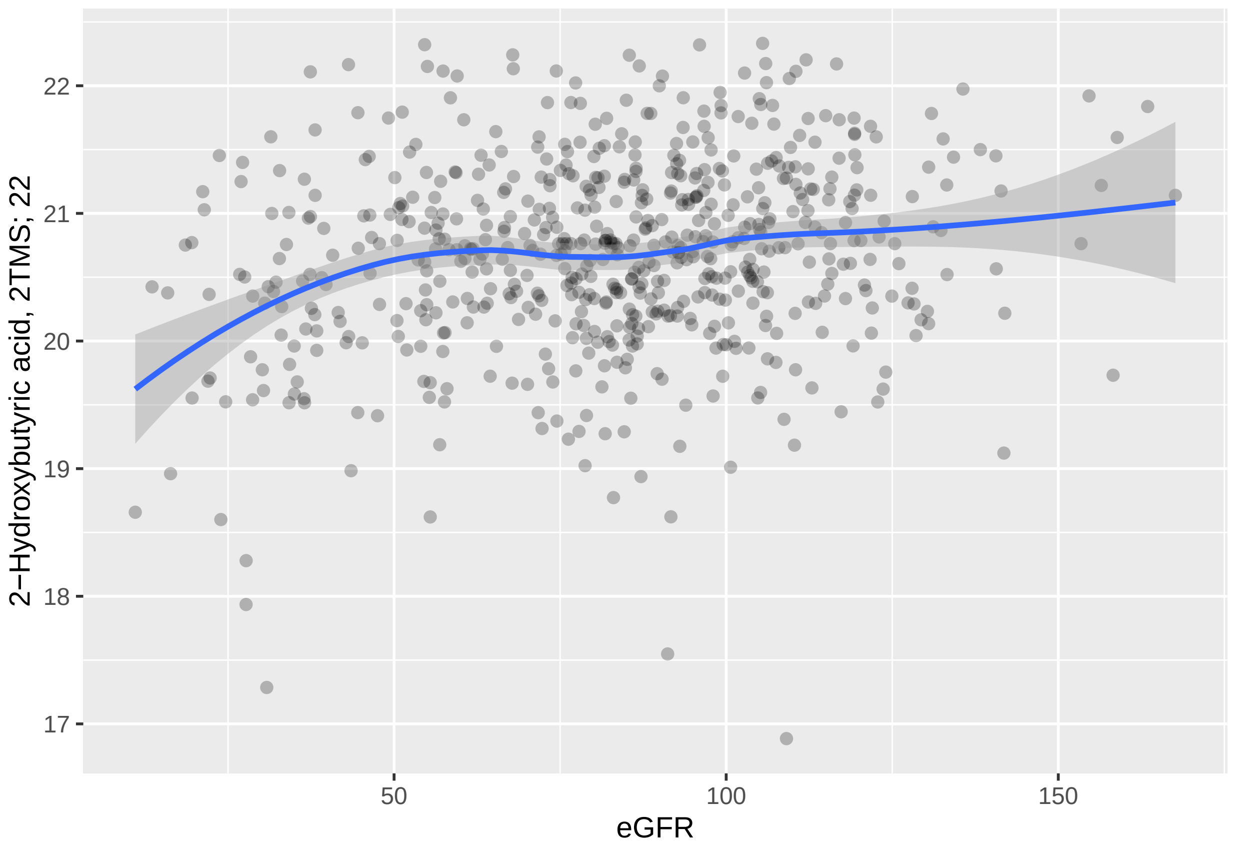


```
## `geom_smooth()` using method = 'loess' and formula 'y ~ x'
## Warning: Removed 2 rows containing non-finite values (stat_smooth).
## Warning: Removed 2 rows containing missing values (geom_point).
```

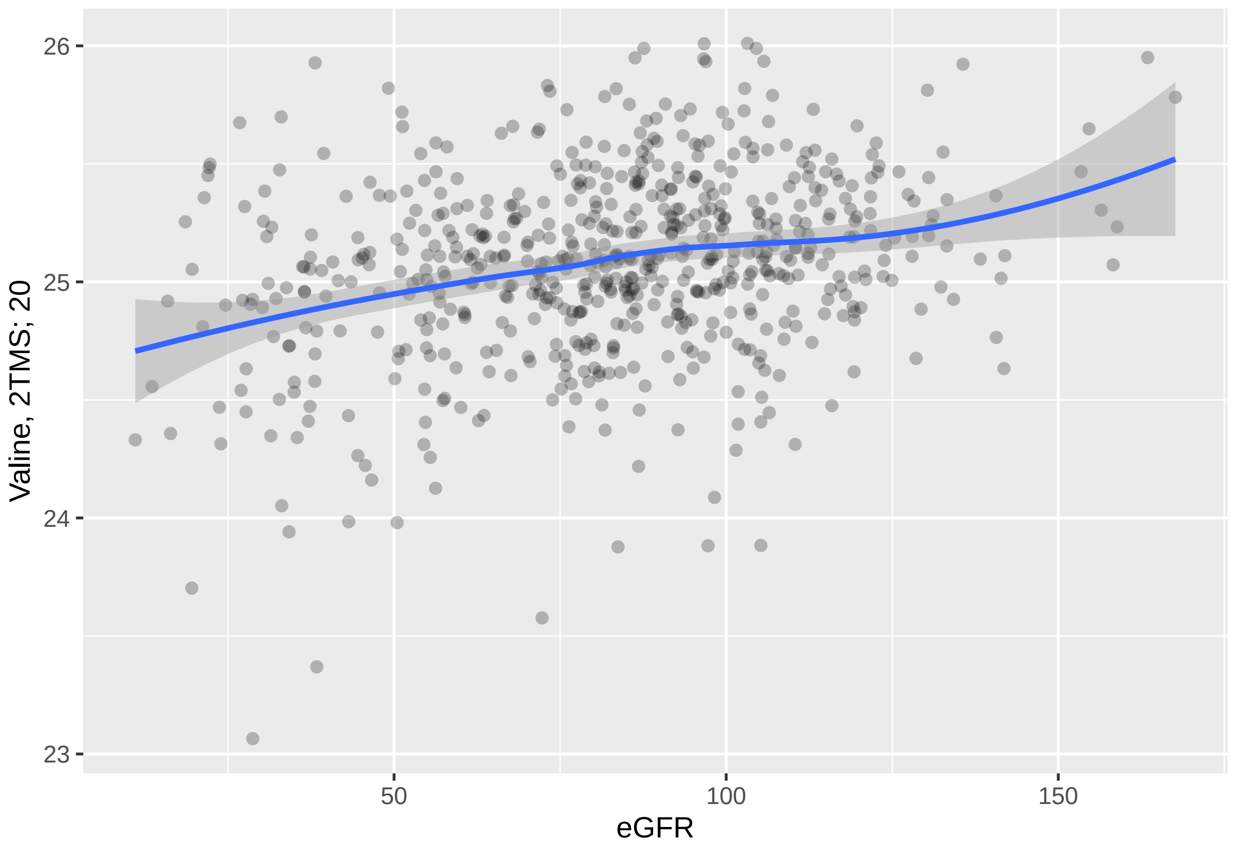


```
## `geom_smooth()` using method = 'loess' and formula 'y ~ x'
## Warning: Removed 2 rows containing non-finite values (stat_smooth).
## Warning: Removed 2 rows containing missing values (geom_point).
```

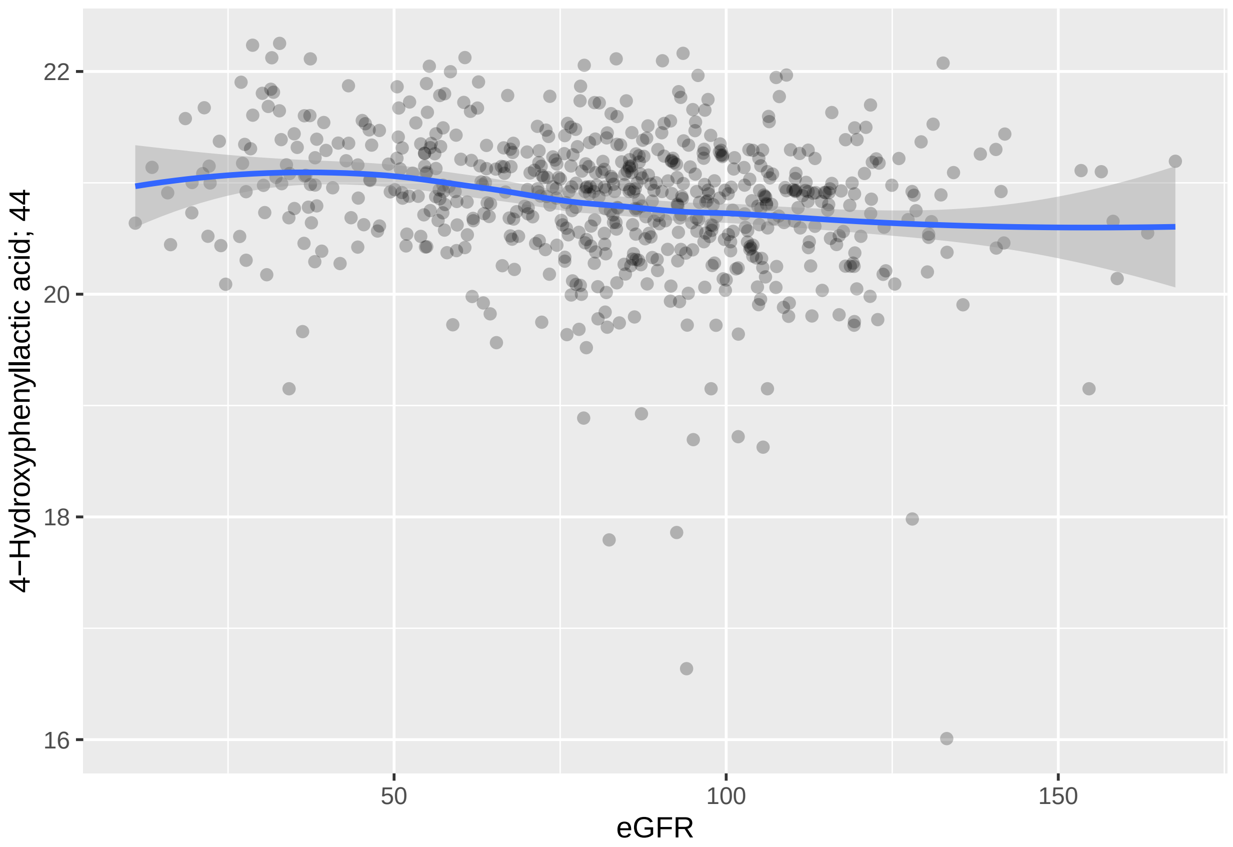


```
## `geom_smooth()` using method = 'loess' and formula 'y ~ x'
## Warning: Removed 2 rows containing non-finite values (stat_smooth).
## Warning: Removed 2 rows containing missing values (geom_point).
```

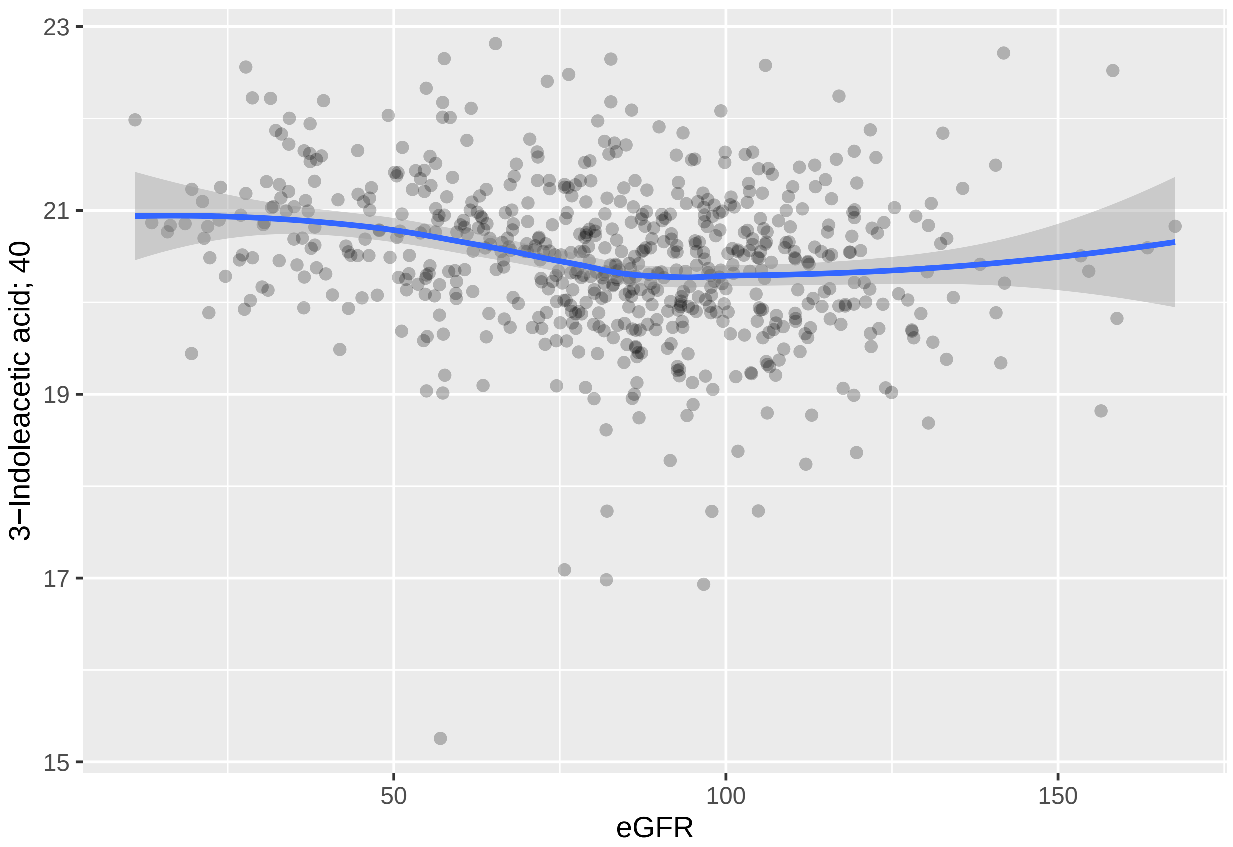


```
## `geom_smooth()` using method = 'loess' and formula 'y ~ x'
## Warning: Removed 2 rows containing non-finite values (stat_smooth).
## Warning: Removed 2 rows containing missing values (geom_point).
```

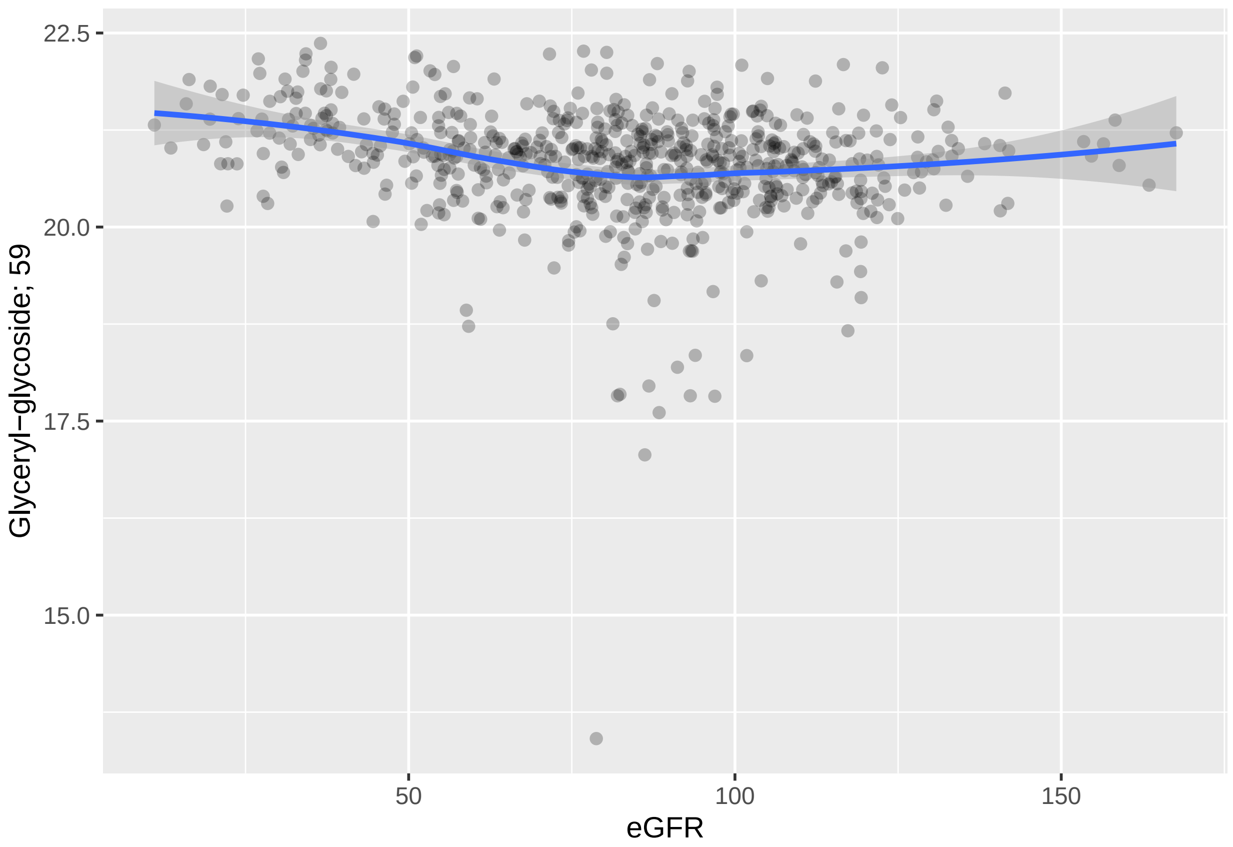


```
## `geom_smooth()` using method = 'loess' and formula 'y ~ x'
## Warning: Removed 2 rows containing non-finite values (stat_smooth).
## Warning: Removed 2 rows containing missing values (geom_point).
```

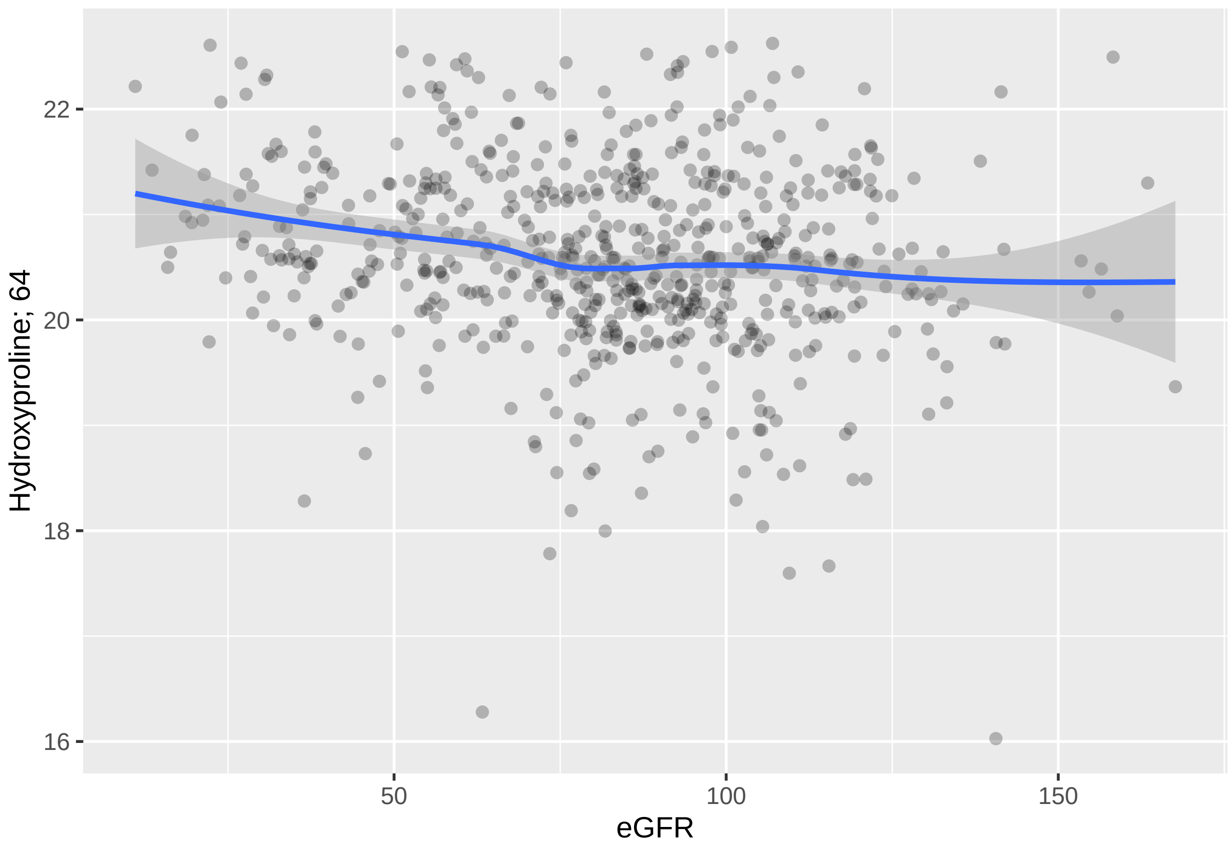


```
## `geom_smooth()` using method = 'loess' and formula 'y ~ x'
## Warning: Removed 2 rows containing non-finite values (stat_smooth).
## Warning: Removed 2 rows containing missing values (geom_point).
```

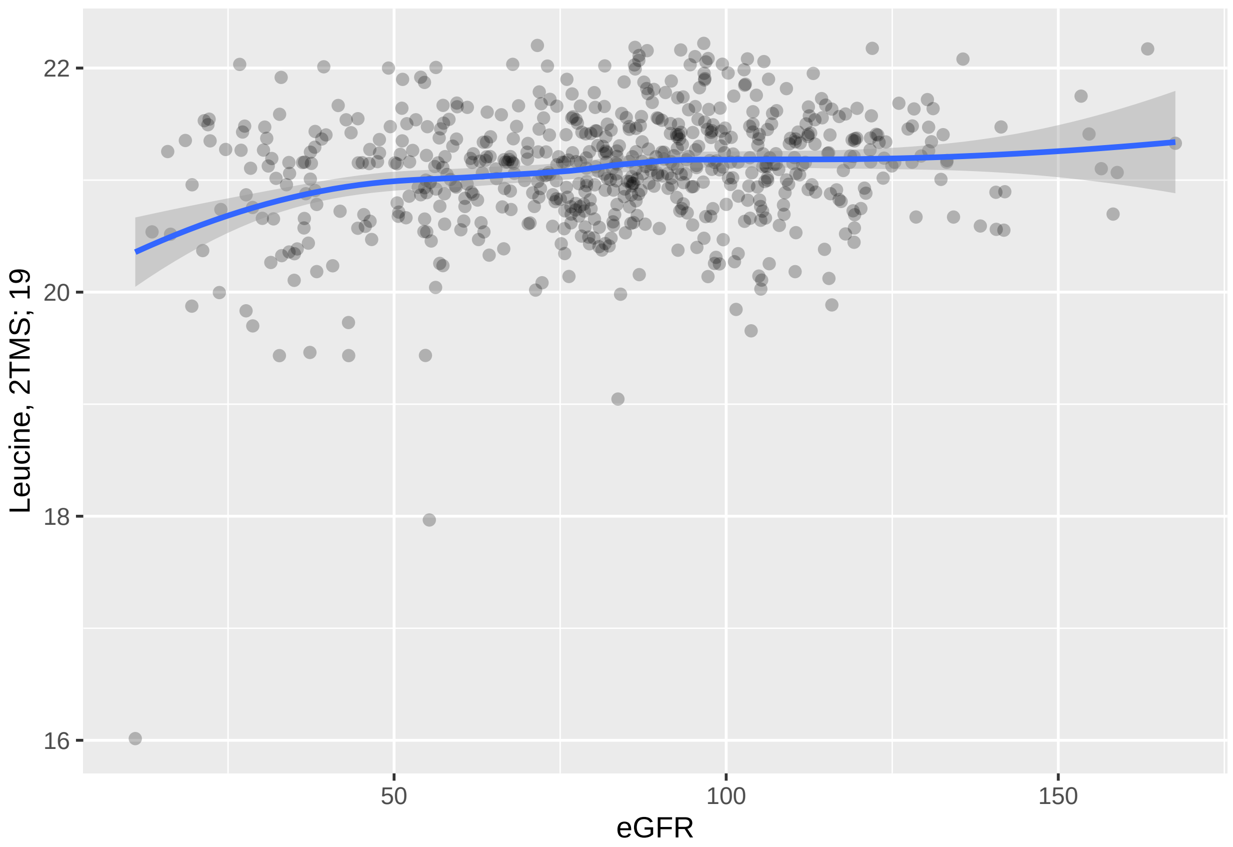


```
## `geom_smooth()` using method = 'loess' and formula 'y ~ x'
## Warning: Removed 2 rows containing non-finite values (stat_smooth).
## Warning: Removed 2 rows containing missing values (geom_point).
```

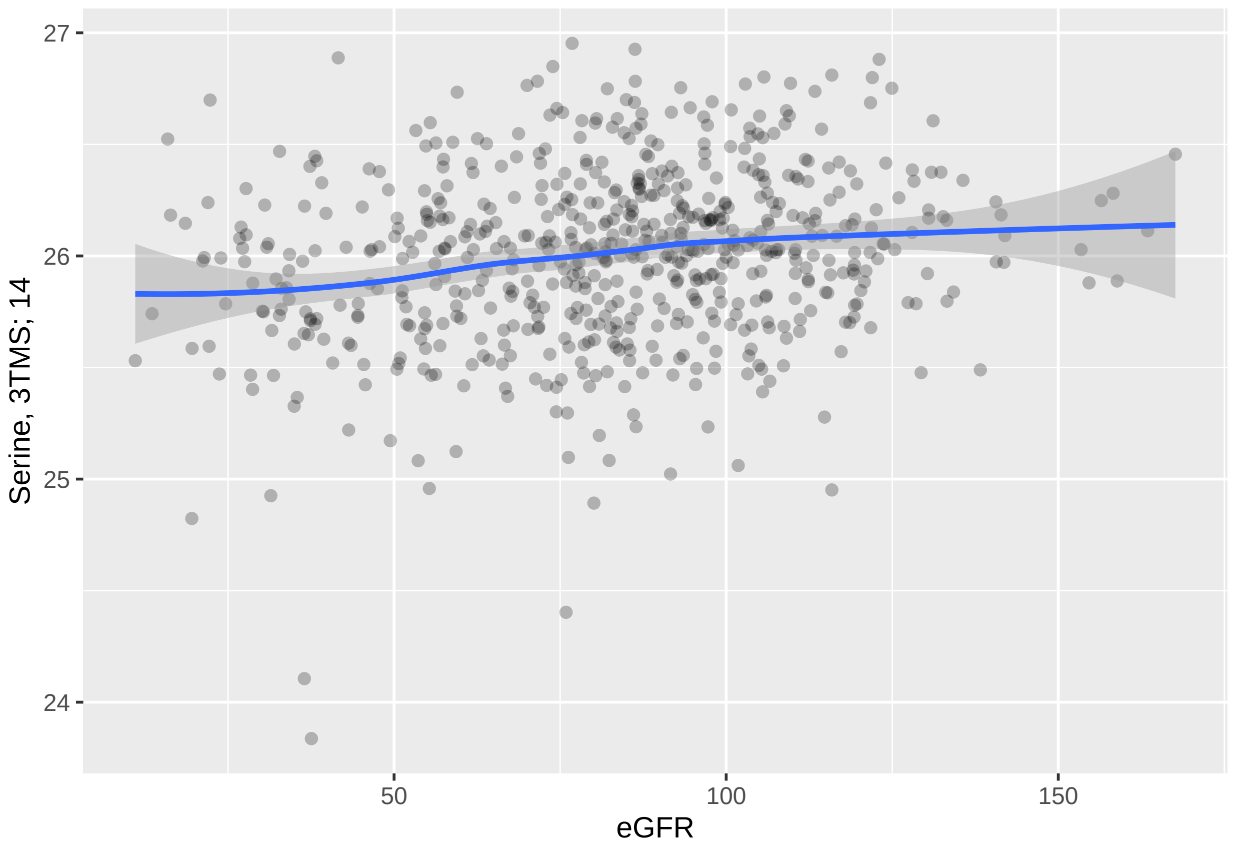


```
## `geom_smooth()` using method = 'loess' and formula 'y ~ x'
## Warning: Removed 2 rows containing non-finite values (stat_smooth).
## Warning: Removed 2 rows containing missing values (geom_point).
```

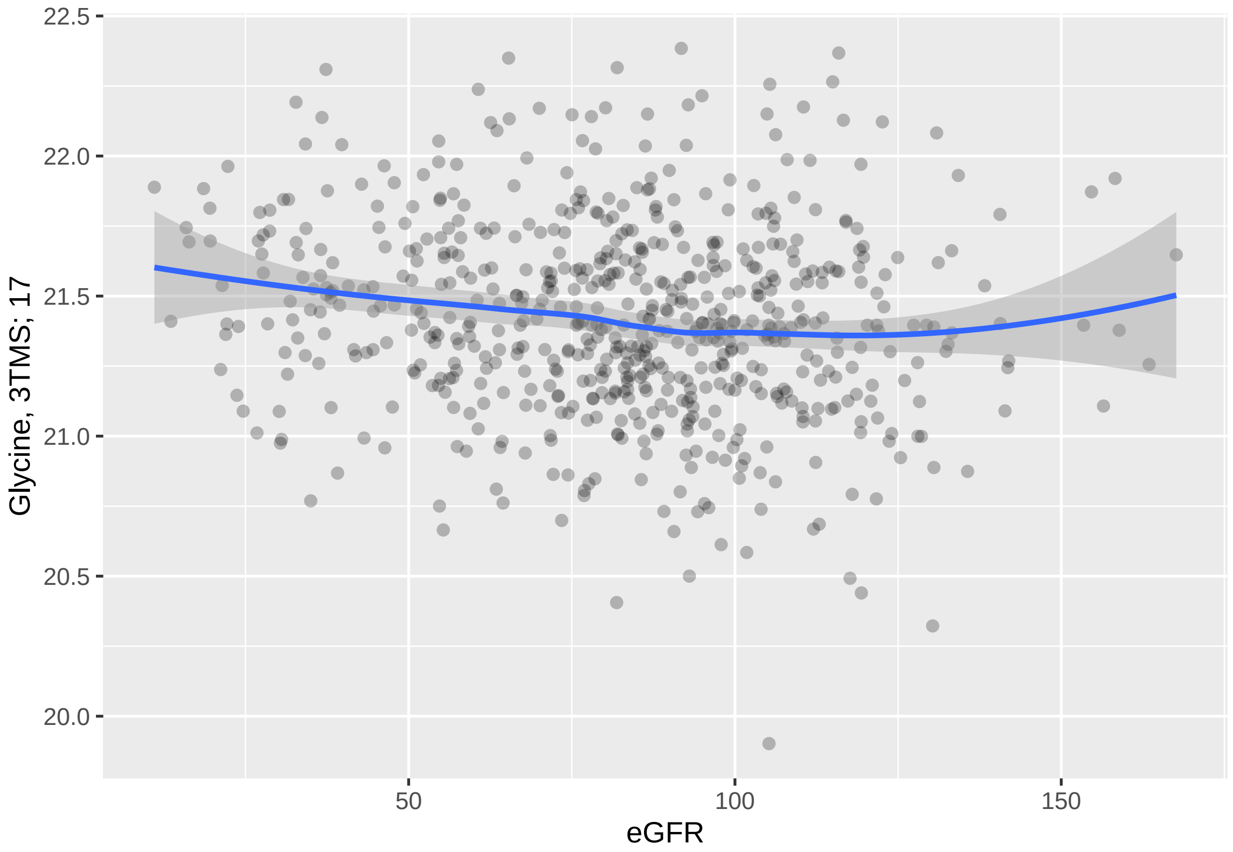


```
## `geom_smooth()` using method = 'loess' and formula 'y ~ x'
## Warning: Removed 2 rows containing non-finite values (stat_smooth).
## Warning: Removed 2 rows containing missing values (geom_point).
```

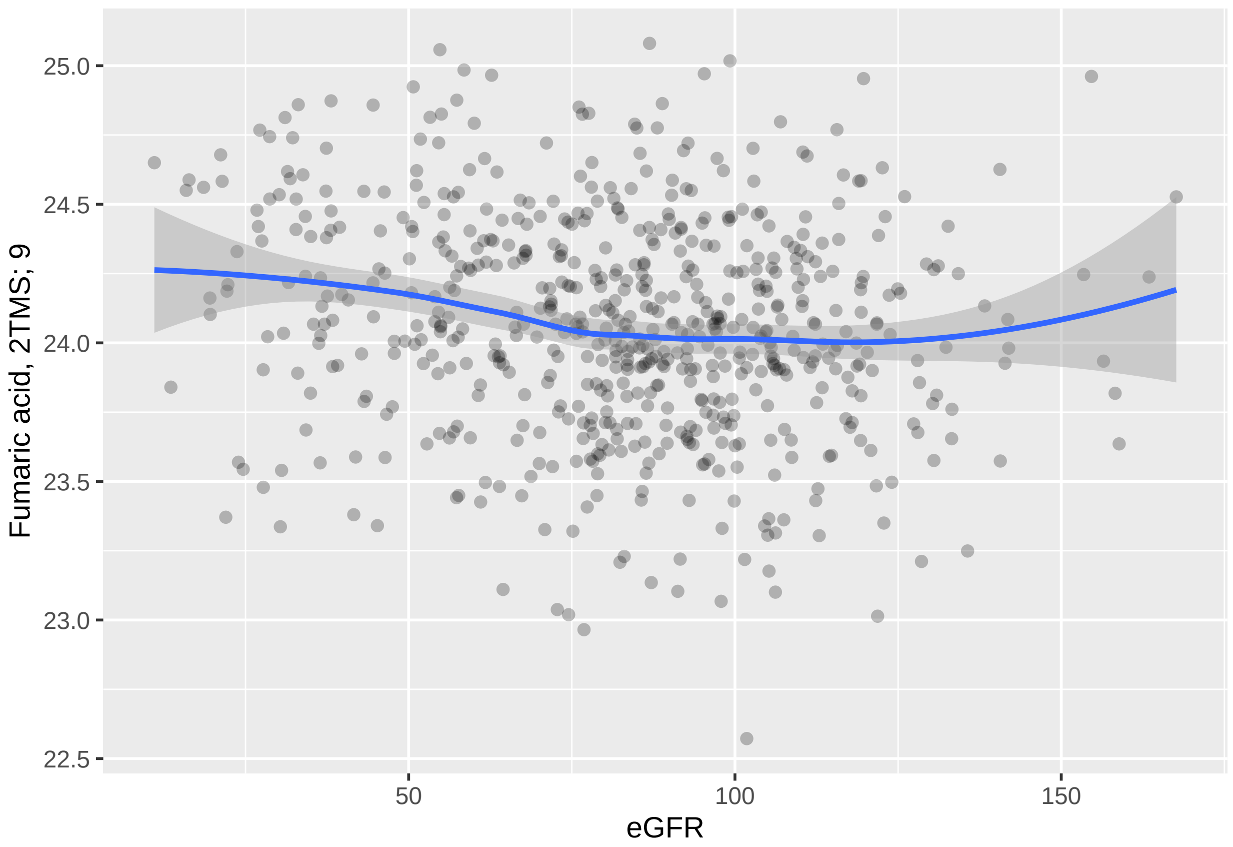


```
## `geom_smooth()` using method = 'loess' and formula 'y ~ x'
## Warning: Removed 2 rows containing non-finite values (stat_smooth).
## Warning: Removed 2 rows containing missing values (geom_point).
```

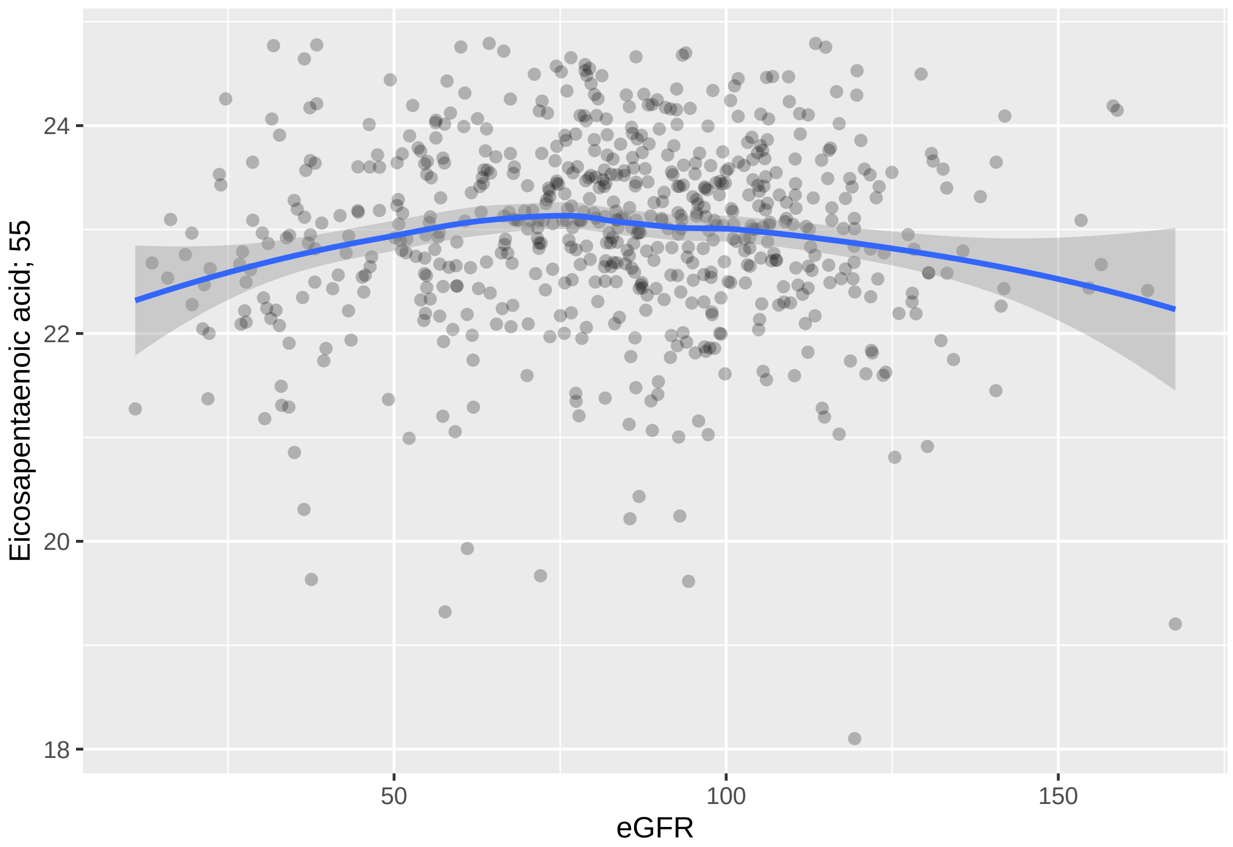


```
## `geom_smooth()` using method = 'loess' and formula 'y ~ x'

## Warning: Removed 2 rows containing non-finite values (stat_smooth).

## Warning: Removed 2 rows containing missing values (geom_point).
```

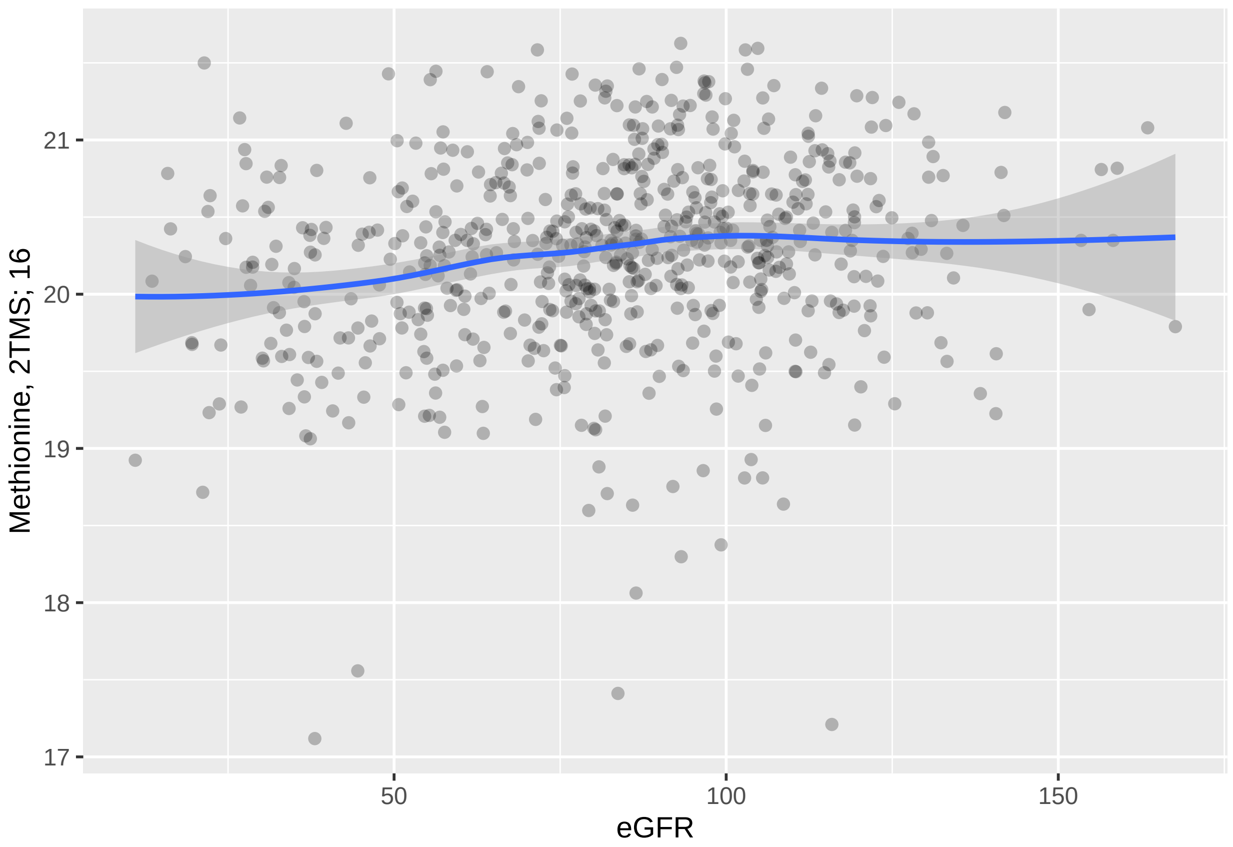

Methionine, 2TMS; 16
eGFR
17
18
19
20
21
50
100
150

11 Combined Subfigures for the Two Metabolites of Interest

```
## `geom_smooth()` using method = 'loess' and formula 'y ~ x'
## Warning: Removed 2 rows containing non-finite values (stat_smooth).
## Warning: Removed 2 rows containing missing values (geom_point).
## `geom_smooth()` using method = 'loess' and formula 'y ~ x'
## Warning: Removed 2 rows containing non-finite values (stat_smooth).
## Warning: Removed 2 rows containing missing values (geom_point).
```

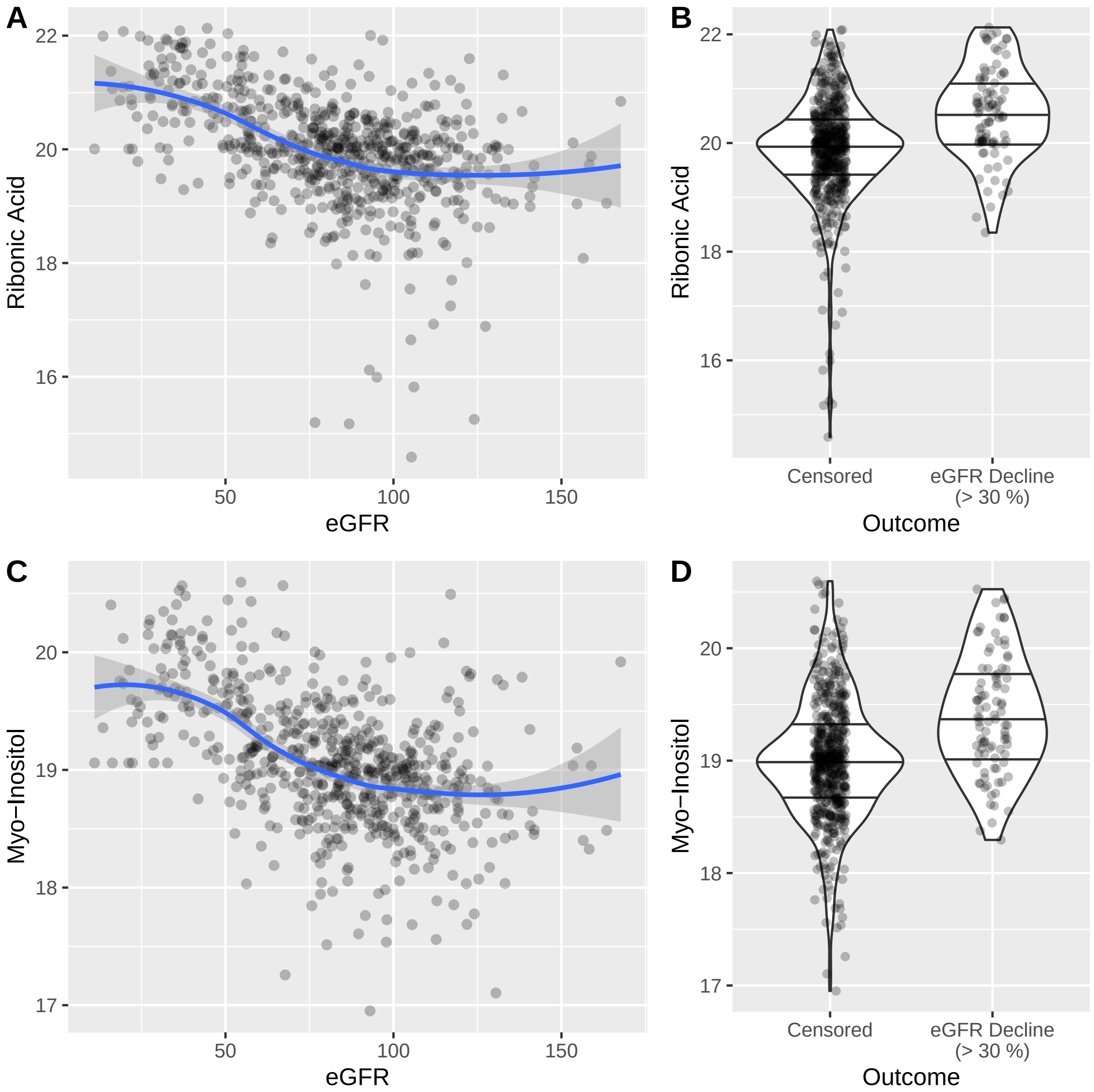

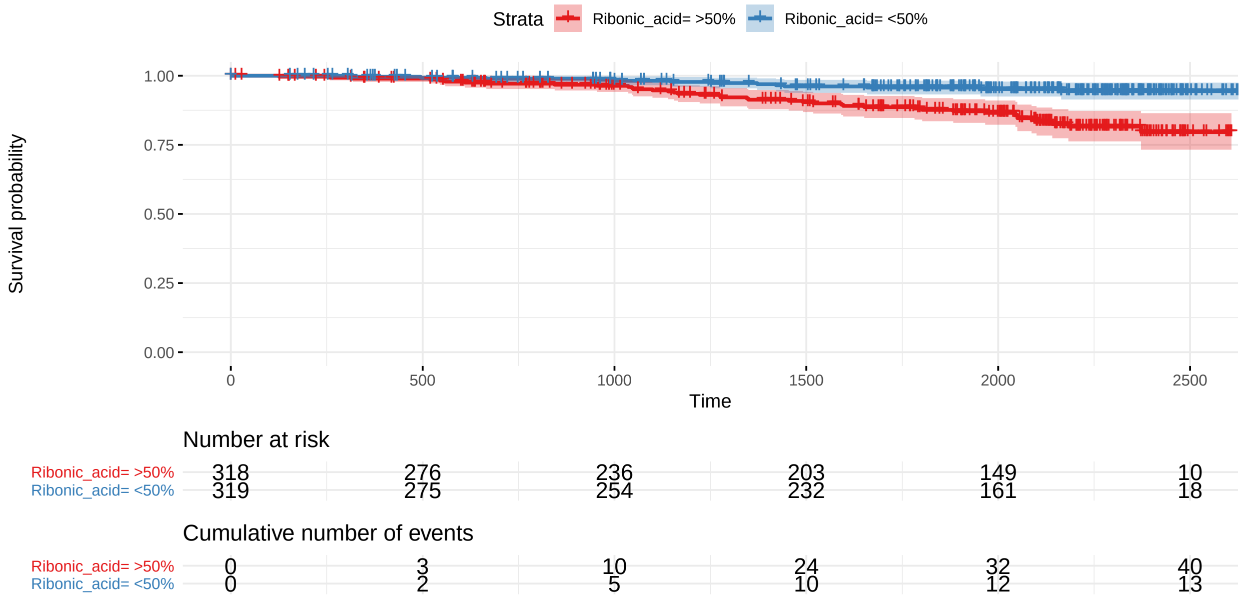
Strata
Ribonic_acid= >50%
Ribonic_acid= <50%
Survival probability
1.00
0.75
0.50
0.25
0.00
0
500
1000
1500
2000
2500
Time
Number at risk
Ribonic_acid= >50% 318 276 236 203 149 10
Ribonic_acid= <50% 319 275 254 232 161 18
Cumulative number of events
Ribonic_acid= >50% 0 3 10 24 32 40
Ribonic_acid= <50% 0 2 5 10 12 13

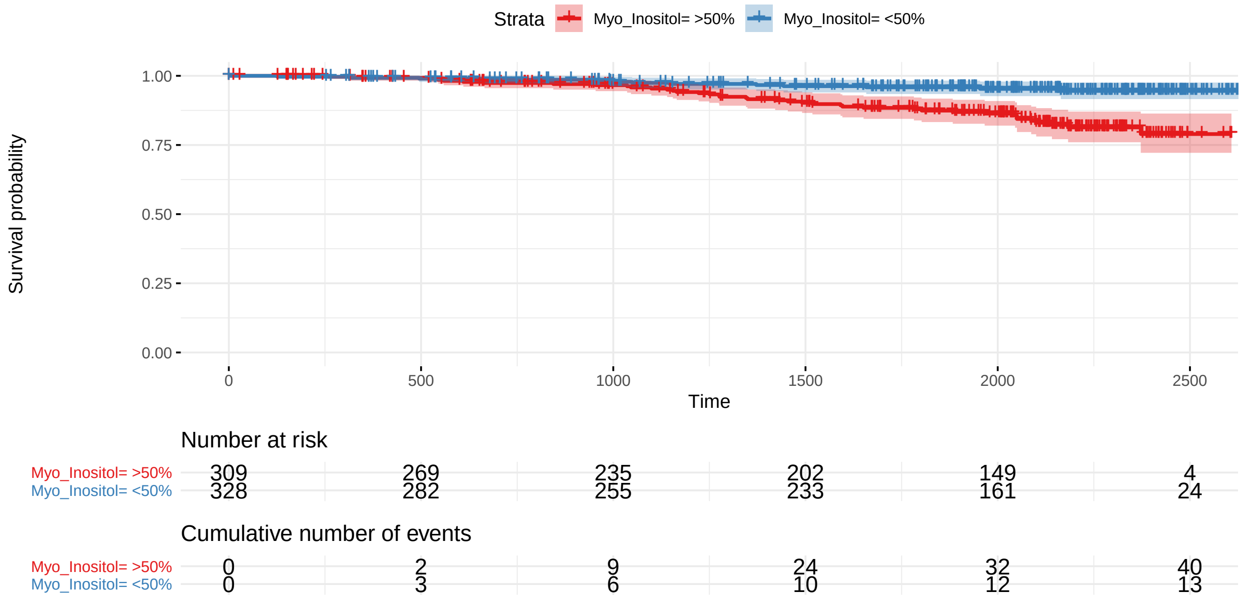
Strata
Myo_Inositol= >50%
Myo_Inositol= <50%
Survival probability
1.00
0.75
0.50
0.25
0.00
0
500
1000
1500
2000
2500
Time
Number at risk
Myo_Inositol= >50% 309 269 235 202 149 4
Myo_Inositol= <50% 328 282 255 233 161 24
Cumulative number of events
Myo_Inositol= >50% 0 2 9 24 32 40
Myo_Inositol= <50% 0 3 6 10 12 13

```
## [[1]]
## NULL
```

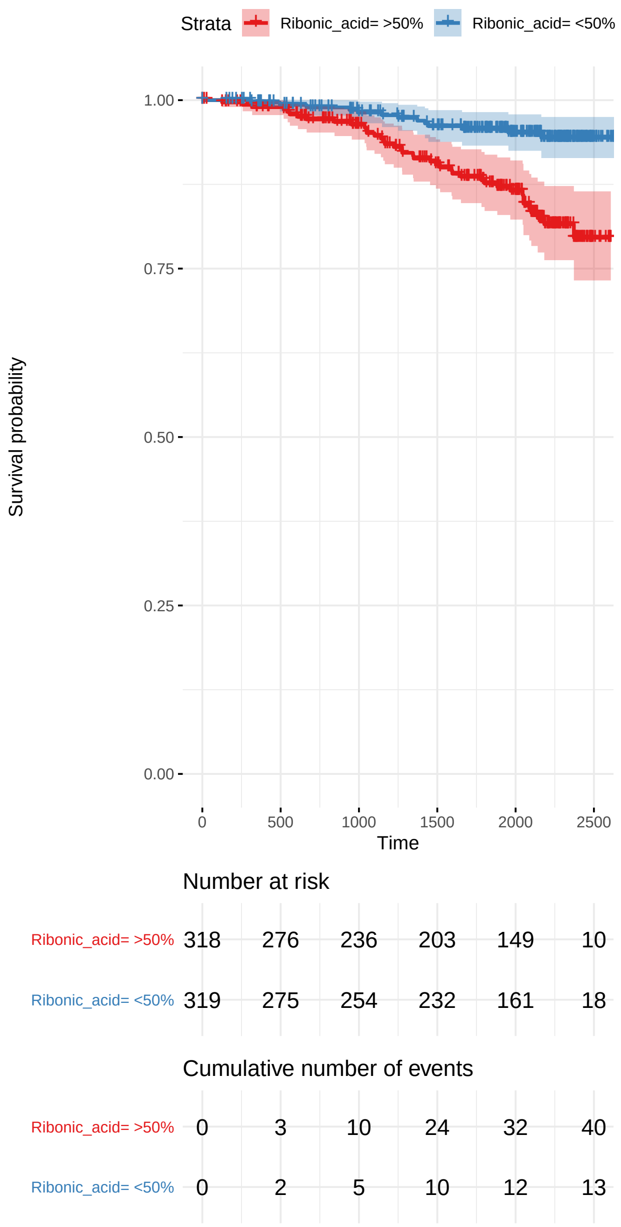
Strata
Ribonic_acid= >50%
Ribonic_acid= <50%
Survival probability
1.00
0.75
0.50
0.25
0.00
0
500
1000
1500
2000
2500
Time
Number at risk
Ribonic_acid= >50% 318 276 236 203 149 10
Ribonic_acid= <50% 319 275 254 232 161 18
Cumulative number of events
Ribonic_acid= >50% 0 3 10 24 32 40
Ribonic_acid= <50% 0 2 5 10 12 13

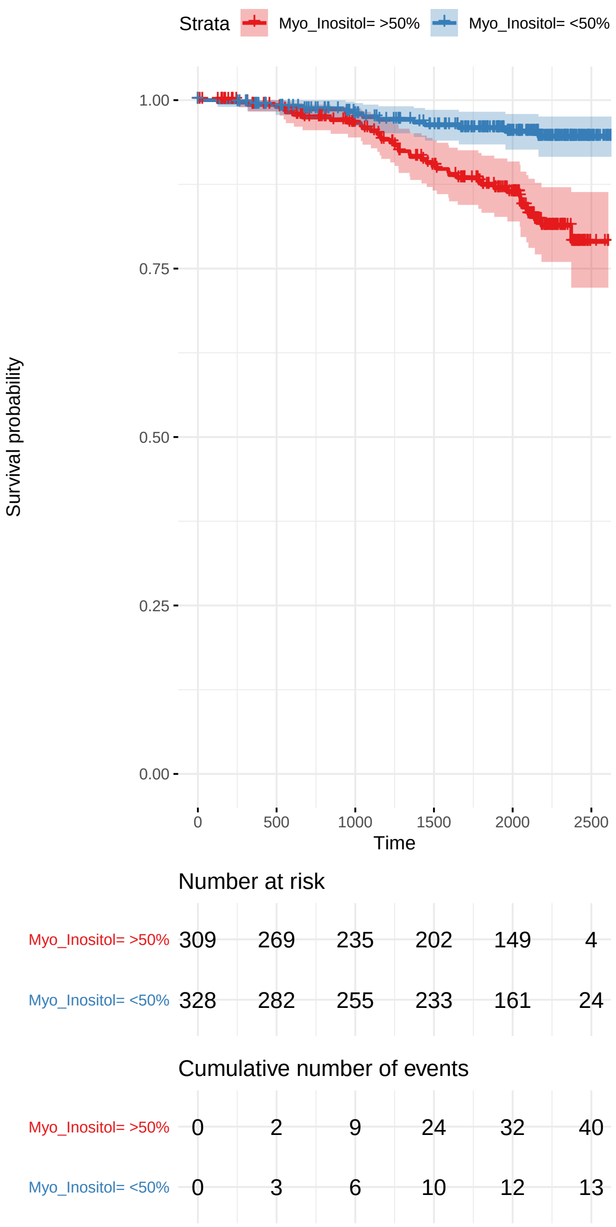
Strata
Myo_Inositol= >50%
Myo_Inositol= <50%
Survival probability
1.00
0.75
0.50
0.25
0.00
0
500
1000
1500
2000
2500
Time
Number at risk
Myo_Inositol= >50% 309 269 235 202 149 4
Myo_Inositol= <50% 328 282 255 233 161 24
Cumulative number of events
Myo_Inositol= >50% 0 2 9 24 32 40
Myo_Inositol= <50% 0 3 6 10 12 13

```
## [[1]]
## NULL
```

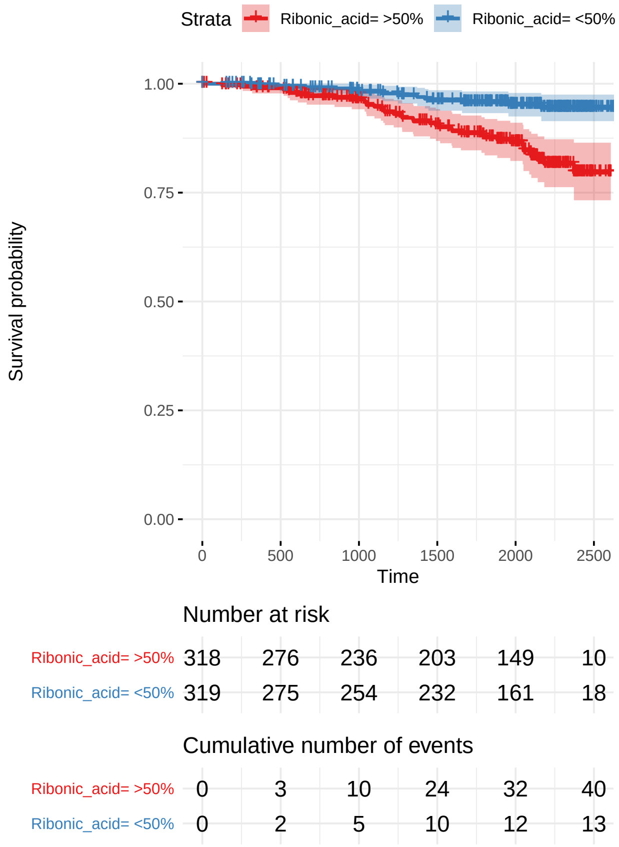
Strata
Ribonic_acid= >50%
Ribonic_acid= <50%
Survival probability
1.00
0.75
0.50
0.25
0.00
0
500
1000
1500
2000
2500
Time
Number at risk
Ribonic_acid= >50% 318 276 236 203 149 10
Ribonic_acid= <50% 319 275 254 232 161 18
Cumulative number of events
Ribonic_acid= >50% 0 3 10 24 32 40
Ribonic_acid= <50% 0 2 5 10 12 13

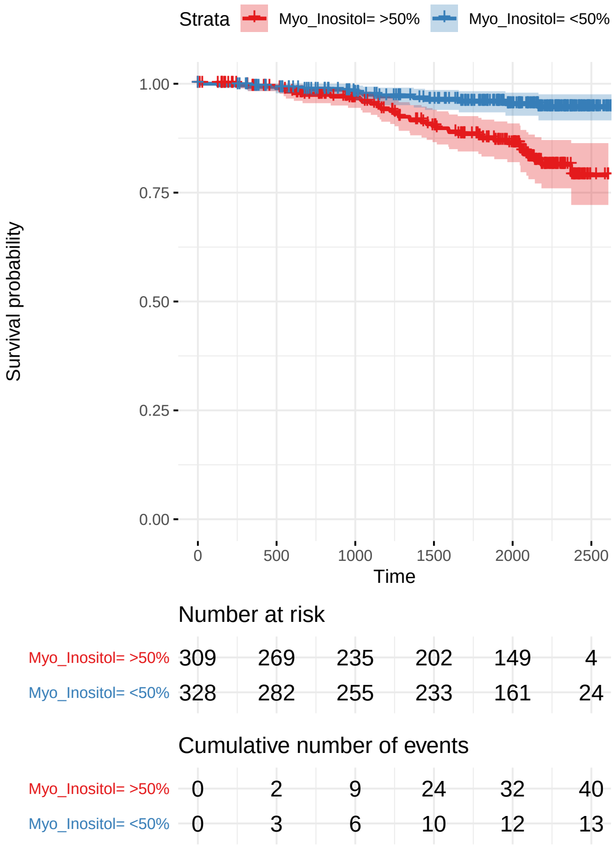
Strata
Myo_Inositol= >50%
Myo_Inositol= <50%
Survival probability
1.00
0.75
0.50
0.25
0.00
0
500
1000
1500
2000
2500
Time
Number at risk
Myo_Inositol= >50% 309 269 235 202 149 4
Myo_Inositol= <50% 328 282 255 233 161 24
Cumulative number of events
Myo_Inositol= >50% 0 2 9 24 32 40
Myo_Inositol= <50% 0 3 6 10 12 13

```
## [[1]]
## NULL
```

12 Partial Correlation Network

12.1 Full Network

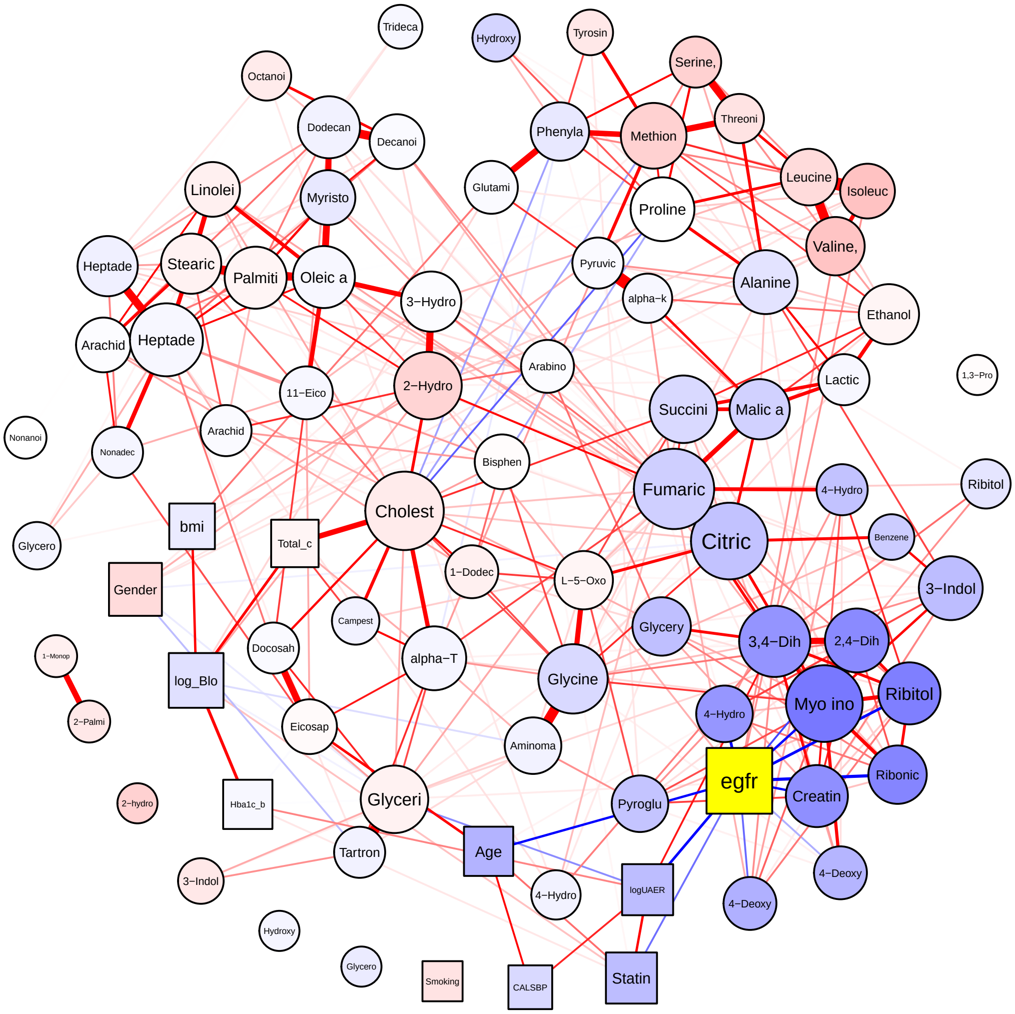

Figure 1: Visualization of the partial correlation network that was inferred with the graphical LASSO algorithm.

12.2 Connected Network

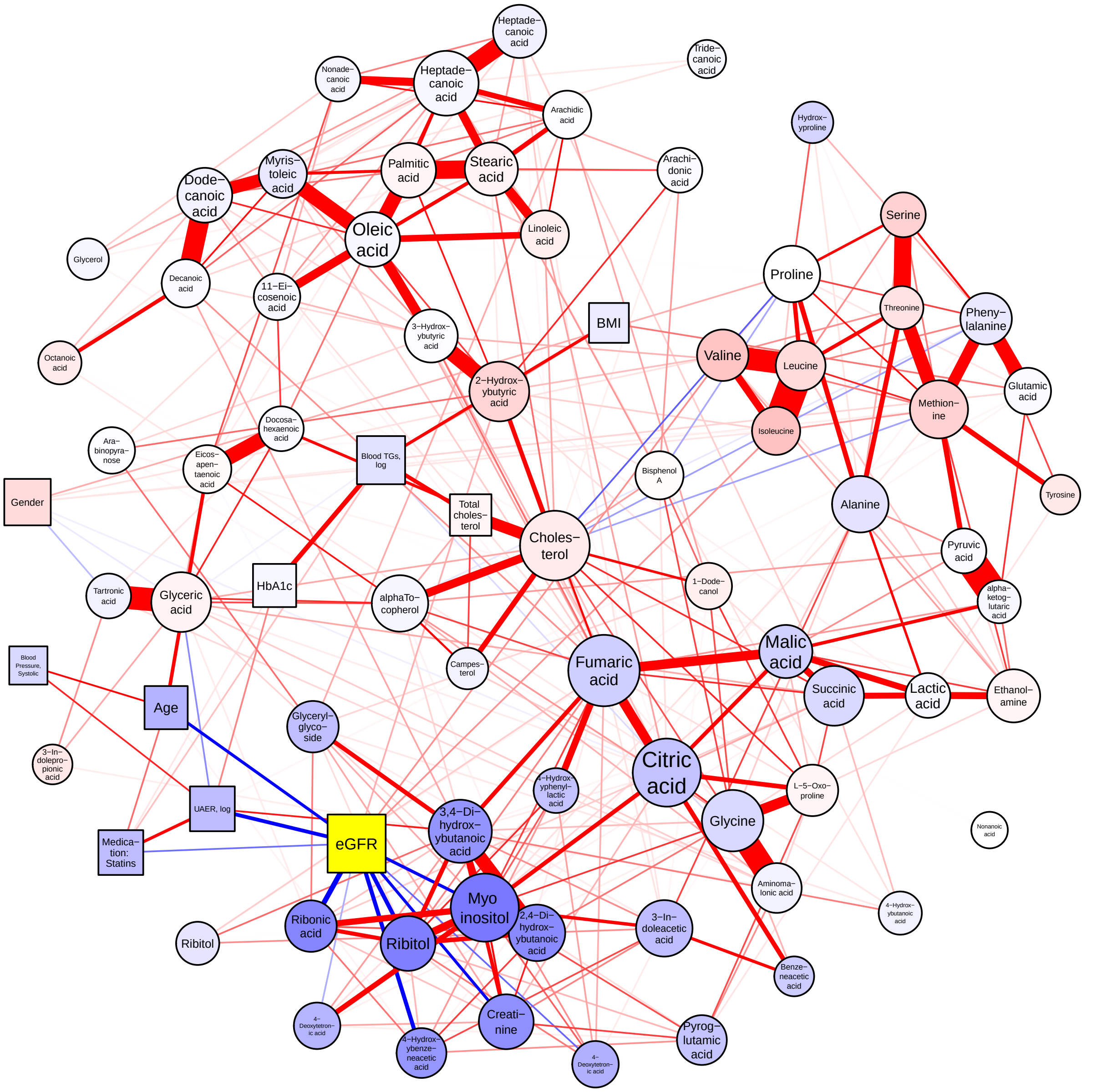

Figure 2: Visualization of the partial correlation network that was inferred with the graphical LASSO algorithm.

12.2.1 Connected Network – Publication

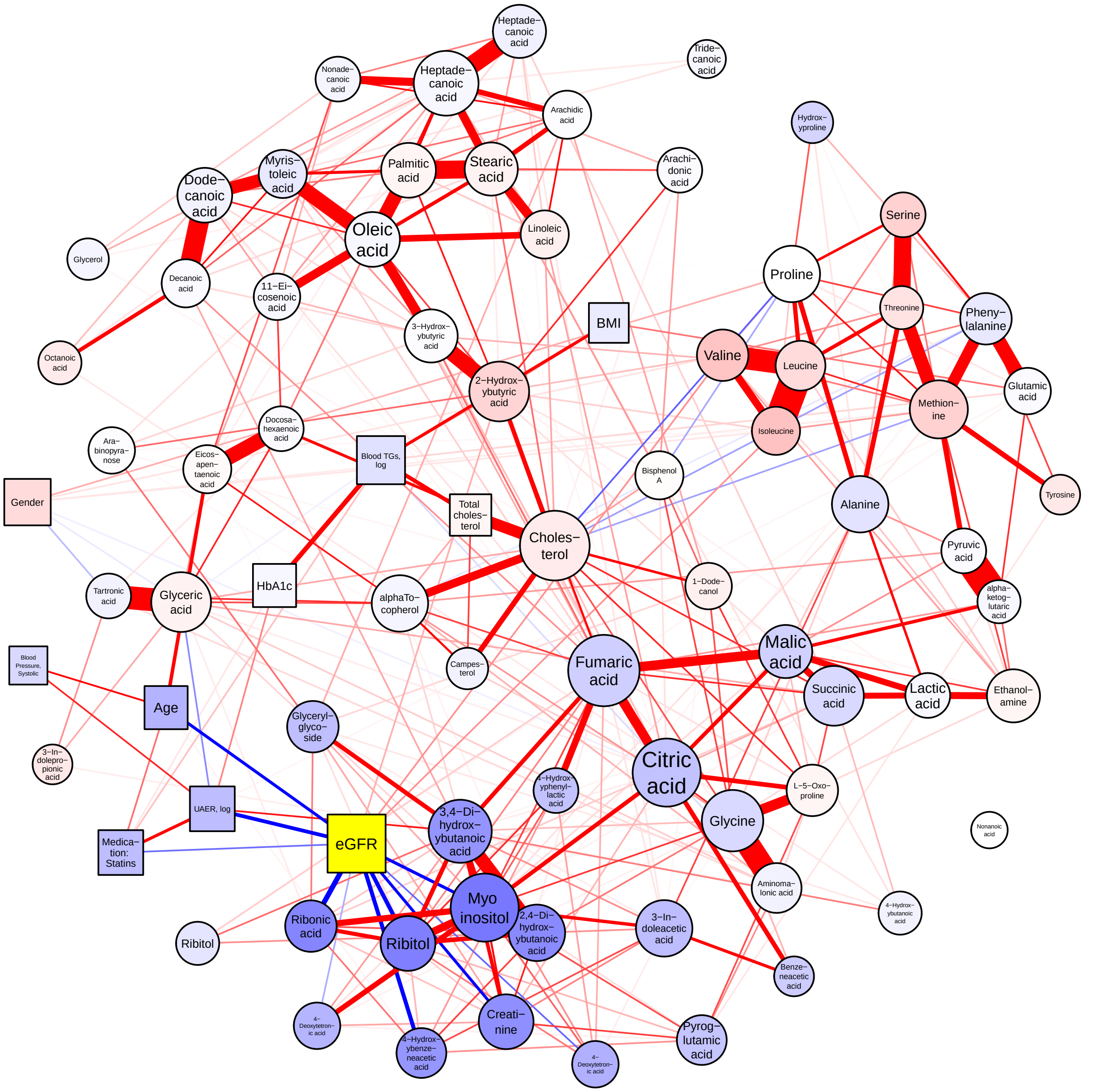

Figure 3: Visualization of the partial correlation network that was inferred with the graphical LASSO algorithm.

12.2.2 Connected Network – Publication with Legends

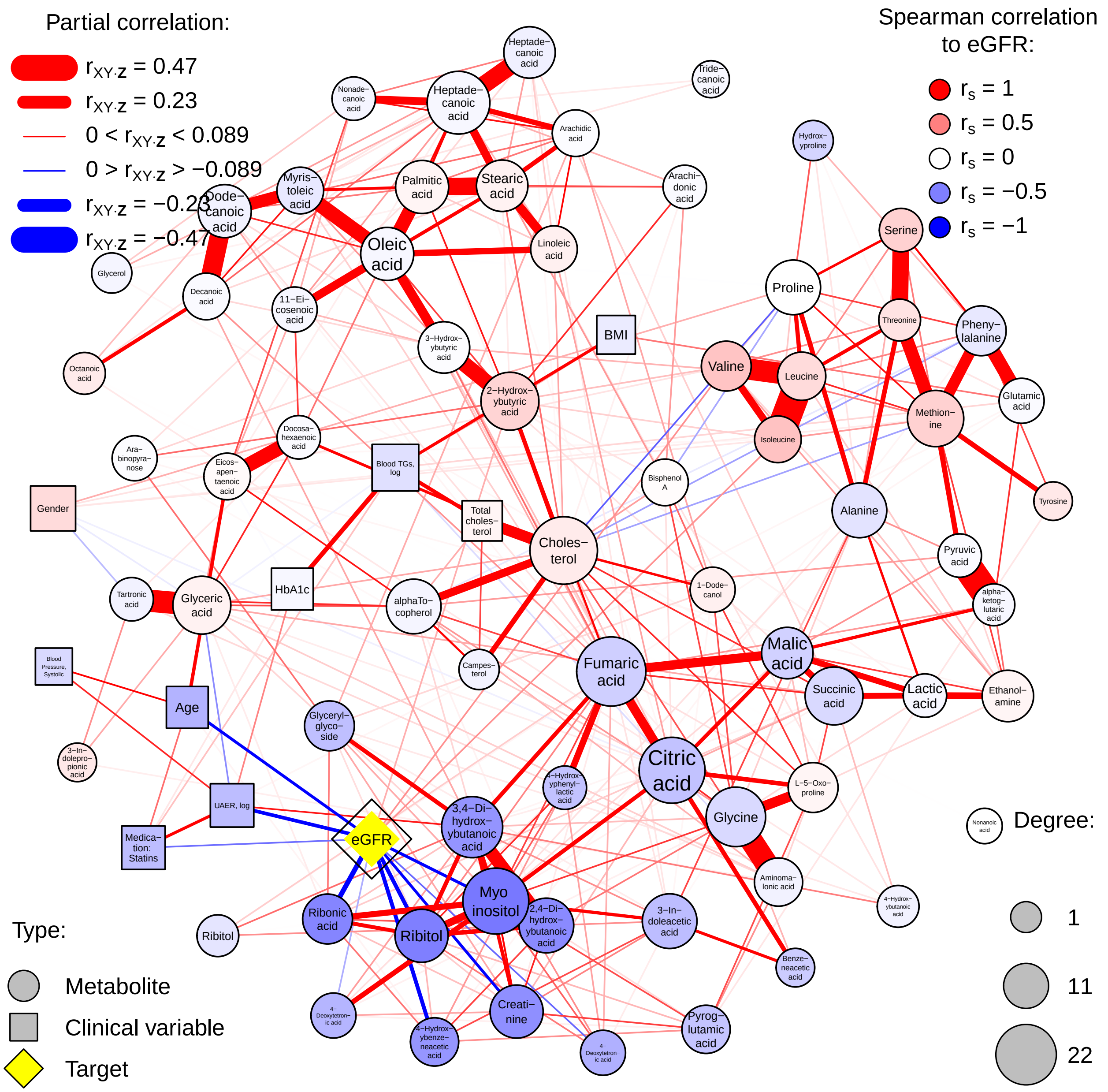

Figure 4: Visualization of the partial correlation network that was inferred with the graphical LASSO algorithm.

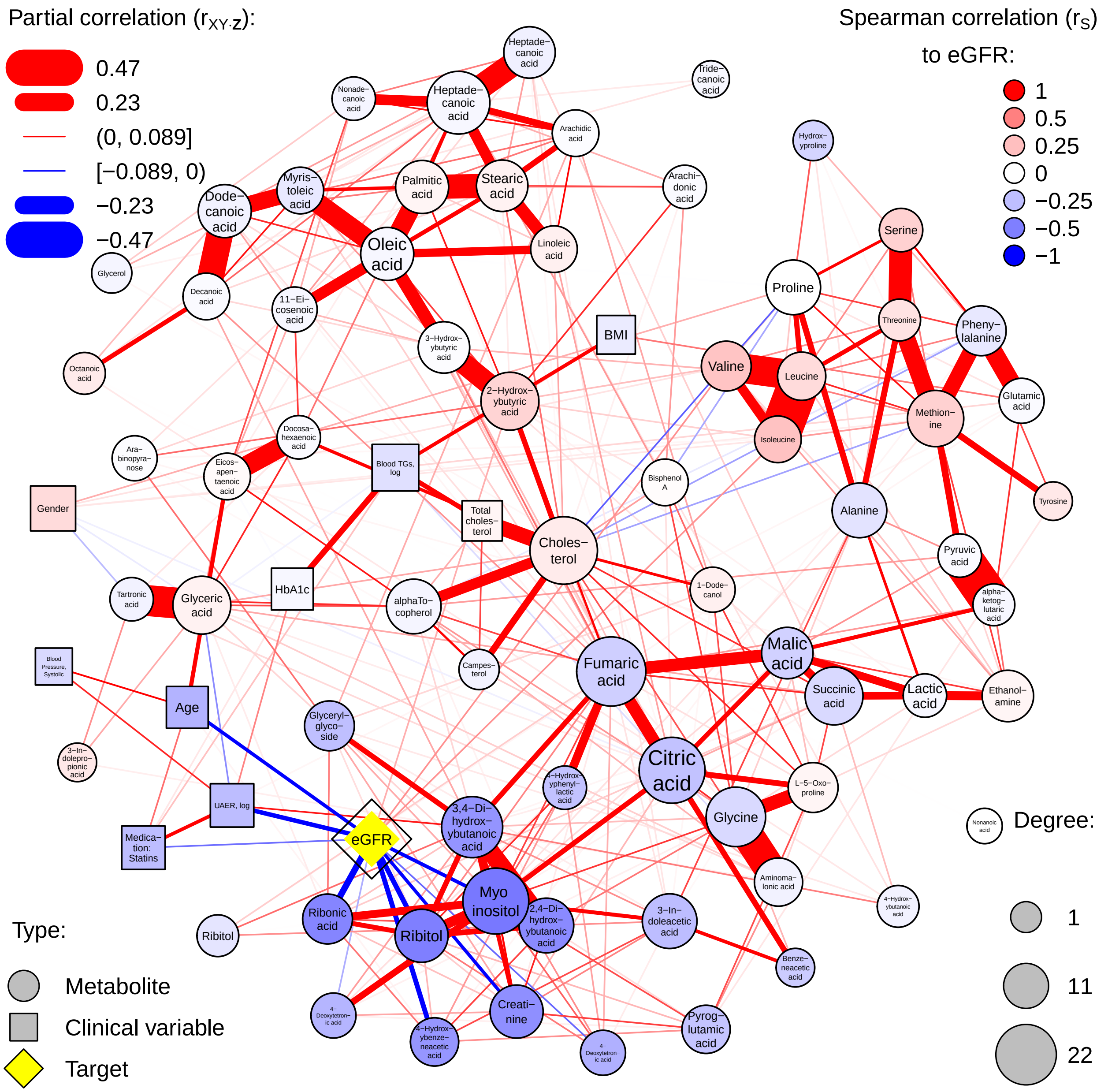

Figure 5: Visualization of the partial correlation network that was inferred with the graphical LASSO algorithm.

12.2.3 Partial Correlations with eGFR

Table 20: Non-zero partial correlations to eGFR.

Name	Partial_Correlation
Ribonic acid	-0.13
Ribitol	-0.12
4-Hydroxybenzeneacetic acid	-0.12
UAER, log	-0.12
Myo inositol	-0.11
Age	-0.11
Creatinine	-0.1
2,4-Dihydroxybutanoic acid	-0.066
4-Deoxytetronic acid	-0.053
Medication: Statins	-0.05
Valine	0.038
Isoleucine	0.038
4-Deoxytetronic acid	-0.028
2-Hydroxybutyric acid	0.028

13 Supplementary Step 1: Crude Associations of All Metabolites to Clinical Changes Over Time

13.1 eGFR Slope

```
## [1] 485   3
```

```
##      (Intercept) slope_gfr_profil      egfr
## [1,]           1        -29.33939  11.03376
## [2,]           1         16.82789 167.62905
```

```
##
##                               Overall
##   n                             485
##   slope_gfr_profil (mean (sd)) -1.36 (3.55)
##   egfr (mean (sd))             83.05 (26.75)
```

13.1.1 Table

```
##
##
## Table: slope_gfr_profil
##
## Name                            Effect        CI.L        CI.R   AveExpr    P.Value   adj.P.Val
## --------------------------  ----------  ----------  ----------  --------  ---------  ----------
## Ribitol; 71                  -3.27e-02   -0.048000   -1.74e-02      23.3   3.09e-05     0.00232
## Succinic acid, 2TMS; 7        1.41e-02    0.005960    2.23e-02      22.7   7.36e-04     0.02760
## Aminomalonic acid; 45         2.47e-02    0.009090    4.03e-02      24.3   1.98e-03     0.04950
## 3,4-Dihydroxybutanoic aci    -1.97e-02   -0.033000   -6.34e-03      15.9   3.91e-03     0.07340
## L-5-Oxoproline; 63            1.29e-02    0.003760    2.20e-02      27.5   5.76e-03     0.08640
## Octanoic acid; 68            -1.28e-02   -0.022200   -3.34e-03      24.2   8.11e-03     0.10100
## Threonine, 3TMS; 12           1.76e-02    0.004010    3.13e-02      28.6   1.13e-02     0.11800
## Heptadecanoic acid; 61        1.11e-02    0.002200    1.99e-02      23.4   1.46e-02     0.11800
## Glyceric acid; 30             1.75e-02    0.003430    3.15e-02      20.4   1.49e-02     0.11800
## Methionine, 2TMS; 16          1.96e-02    0.003720    3.56e-02      20.3   1.57e-02     0.11800
## Tridecanoic acid; 74          1.55e-02    0.002360    2.86e-02      20.0   2.08e-02     0.13000
## Ribonic acid; 72             -2.45e-02   -0.045300   -3.74e-03      20.0   2.08e-02     0.13000
## Myo inositol 6TMS; 1         -1.30e-02   -0.025000   -1.10e-03      19.0   3.23e-02     0.18600
## Bisphenol A; 48               1.44e-02    0.000302    2.86e-02      21.2   4.53e-02     0.24300
## Myristoleic acid; 65         -2.79e-02   -0.055800    8.61e-05      19.9   5.07e-02     0.24300
## 1-Dodecanol; 36               1.15e-02   -0.000096    2.31e-02      20.2   5.19e-02     0.24300
## Tartronic acid; 73            2.02e-02   -0.001480    4.19e-02      21.3   6.78e-02     0.29000
## Glycine, 3TMS; 17             8.22e-03   -0.000663    1.71e-02      21.4   6.96e-02     0.29000
## Ethanolamine; 56              9.33e-03   -0.001410    2.01e-02      22.7   8.83e-02     0.34900
## Palmitic acid, TMS; 5        -5.48e-03   -0.012100    1.13e-03      21.4   1.04e-01     0.39000
## 2-Hydroxybutyric acid, 2T    -1.41e-02   -0.032100    3.88e-03      20.7   1.24e-01     0.42200
## Glyceryl-glycoside; 59       -1.46e-02   -0.033300    4.02e-03      20.8   1.24e-01     0.42200
## Tyrosine; 75                  2.27e-02   -0.010000    5.54e-02      23.3   1.73e-01     0.56500
## Dodecanoic acid; 54          -1.09e-02   -0.027700    5.90e-03      22.7   2.03e-01     0.60200
## Oleic acid, TMS; 3           -6.85e-03   -0.017400    3.74e-03      17.1   2.04e-01     0.60200
## 3-Indolepropionic acid; 4     1.96e-02   -0.011000    5.01e-02      19.5   2.09e-01     0.60200
## Isoleucine, 2TMS; 18         -9.43e-03   -0.024500    5.64e-03      26.0   2.19e-01     0.61000
## Nonanoic acid; 67             6.30e-03   -0.004530    1.71e-02      24.4   2.54e-01     0.67900
## 2,4-Dihydroxybutanoic aci    -8.09e-03   -0.022500    6.33e-03      16.5   2.71e-01     0.70100
## Leucine, 2TMS; 19            -7.43e-03   -0.021100    6.28e-03      21.1   2.88e-01     0.71600
## 3-Indoleacetic acid; 40      -1.08e-02   -0.031000    9.45e-03      20.5   2.96e-01     0.71600
## Lactic acid; 29               4.77e-03   -0.004450    1.40e-02      25.4   3.10e-01     0.72600
## Arachidic acid; 46            4.11e-03   -0.004290    1.25e-02      21.8   3.37e-01     0.75300
## Glutamic acid, 3TMS; 8       -6.70e-03   -0.021100    7.66e-03      23.0   3.60e-01     0.75300
## Docosahexaenoic acid; 53      8.74e-03   -0.010300    2.78e-02      24.2   3.67e-01     0.75300
## 4-Deoxytetronic acid; 32      8.22e-03   -0.010300    2.67e-02      21.6   3.83e-01     0.75300
## 4-Hydroxybutanoic acid; 4     2.35e-03   -0.002990    7.68e-03      27.3   3.87e-01     0.75300
## Heptadecanoic acid; 60        5.63e-03   -0.007220    1.85e-02      21.8   3.90e-01     0.75300
## Phenylalanine, 2TMS; 13       3.75e-03   -0.004840    1.23e-02      24.3   3.92e-01     0.75300
## Hydroxyproline; 64           -9.44e-03   -0.032000    1.31e-02      20.6   4.12e-01     0.77200
## Eicosapentaenoic acid; 55     9.29e-03   -0.013600    3.22e-02      23.0   4.25e-01     0.77800
## Pyroglutamic acid; 69         7.55e-03   -0.013300    2.84e-02      22.9   4.77e-01     0.78000
## alpha-Tocopherol; 26          6.62e-03   -0.011600    2.49e-02      18.8   4.77e-01     0.78000
## 1,3-Propanediol; 34          -4.79e-03   -0.018100    8.52e-03      24.1   4.80e-01     0.78000
## Campesterol; 49              -6.47e-03   -0.024600    1.16e-02      21.8   4.83e-01     0.78000
## Arabinopyranose; 51          -6.75e-03   -0.026500    1.30e-02      20.1   5.02e-01     0.78000
```

```
## Cholesterol, TMS; 23          2.80e-03   -0.005580    1.12e-02       21.6   5.12e-01      0.78000
## 4-Deoxytetronic acid; 33     -8.22e-03   -0.033000    1.66e-02       22.2   5.15e-01      0.78000
## Serine, 3TMS; 14              3.22e-03   -0.006760    1.32e-02       26.0   5.27e-01      0.78000
## 3-Hydroxybutyric acid, 2T    -6.46e-03   -0.027600    1.47e-02       21.7   5.48e-01      0.78000
## Nonadecanoic acid; 66         4.06e-03   -0.009250    1.74e-02       20.0   5.49e-01      0.78000
## Valine, 2TMS; 20              2.82e-03   -0.006440    1.21e-02       25.1   5.50e-01      0.78000
## 11-Eicosenoic acid; 35        6.35e-03   -0.014600    2.73e-02       21.9   5.51e-01      0.78000
## Ribitol; 70                  -4.74e-03   -0.021400    1.19e-02       20.2   5.76e-01      0.80000
## Proline, 2TMS; 21            -3.67e-03   -0.017500    1.01e-02       20.8   6.02e-01      0.82100
## Pyruvic acid; 31             -6.22e-03   -0.030800    1.84e-02       19.5   6.20e-01      0.82200
## alpha-ketoglutaric acid,      6.17e-03   -0.018600    3.09e-02       20.2   6.25e-01      0.82200
## Hydroxylamine; 62             2.89e-03   -0.009400    1.52e-02       27.7   6.44e-01      0.82800
## Citric acid, 4TMS; 6          2.67e-03   -0.008930    1.43e-02       20.8   6.52e-01      0.82800
## Malic acid, 3TMS; 11          2.80e-03   -0.010500    1.61e-02       20.0   6.80e-01      0.85000
## Creatinine; 50               -3.40e-03   -0.022500    1.57e-02       21.6   7.28e-01      0.88200
## 2-Palmitoylglycerol; 39      -1.82e-03   -0.012200    8.53e-03       25.9   7.29e-01      0.88200
## 2-hydroxy Isovaleric acid    -4.86e-03   -0.036500    2.67e-02       22.2   7.63e-01      0.90800
## Stearic acid, TMS; 2          5.92e-04   -0.004310    5.49e-03       17.4   8.12e-01      0.95200
## 4-Hydroxybenzeneacetic ac     3.22e-03   -0.028300    3.47e-02       19.4   8.41e-01      0.95900
## Linoleic acid, TMS; 4         7.70e-04   -0.006990    8.53e-03       25.8   8.45e-01      0.95900
## 4-Hydroxyphenyllactic aci    -1.36e-03   -0.017500    1.47e-02       20.8   8.68e-01      0.95900
## Decanoic acid; 52            -1.17e-03   -0.016600    1.42e-02       22.4   8.82e-01      0.95900
## Glycerol; 58                  2.44e-03   -0.030000    3.49e-02       24.8   8.83e-01      0.95900
## Glycerol; 57                 -8.04e-04   -0.013400    1.18e-02       28.7   9.00e-01      0.96400
## Fumaric acid, 2TMS; 9        -5.48e-04   -0.010300    9.25e-03       24.1   9.12e-01      0.96400
## Arachidonic acid, TMS; 24    -4.37e-04   -0.010100    9.27e-03       22.7   9.30e-01      0.96400
## 1-Monopalmitin; 37           -4.67e-04   -0.012300    1.14e-02       29.2   9.38e-01      0.96400
## Alanine, 2TMS; 25            -3.16e-04   -0.010400    9.74e-03       22.8   9.51e-01      0.96400
## Benzeneacetic acid; 47       -3.12e-05   -0.024100    2.41e-02       20.4   9.98e-01      0.99800
##
##
## Table: egfr
##
## Name                            Effect        CI.L        CI.R   AveExpr     P.Value   adj.P.Val
## --------------------------  ----------  ----------  ----------  --------  ----------  ----------
## Myo inositol 6TMS; 1         -1.02e-02   -1.18e-02   -8.65e-03      19.0    0.00e+00    0.00e+00
## Ribitol; 71                  -1.18e-02   -1.38e-02   -9.76e-03      23.3    0.00e+00    0.00e+00
## Ribonic acid; 72             -1.49e-02   -1.77e-02   -1.22e-02      20.0    0.00e+00    0.00e+00
## 2,4-Dihydroxybutanoic aci    -1.03e-02   -1.22e-02   -8.36e-03      16.5    0.00e+00    0.00e+00
## 3,4-Dihydroxybutanoic aci    -8.77e-03   -1.05e-02   -7.01e-03      15.9    0.00e+00    0.00e+00
## Creatinine; 50               -1.18e-02   -1.43e-02   -9.24e-03      21.6    0.00e+00    0.00e+00
## 4-Hydroxybenzeneacetic ac    -1.61e-02   -2.03e-02   -1.20e-02      19.4    0.00e+00    0.00e+00
## 4-Deoxytetronic acid; 33     -1.22e-02   -1.55e-02   -8.88e-03      22.2    0.00e+00    0.00e+00
## Citric acid, 4TMS; 6         -4.68e-03   -6.22e-03   -3.14e-03      20.8    0.00e+00    0.00e+00
## 3-Indoleacetic acid; 40      -7.59e-03   -1.03e-02   -4.91e-03      20.5    0.00e+00    3.00e-07
## Valine, 2TMS; 20              3.40e-03    2.17e-03    4.63e-03      25.1    1.00e-07    6.00e-07
## Isoleucine, 2TMS; 18          5.36e-03    3.37e-03    7.36e-03      26.0    2.00e-07    1.30e-06
## 4-Hydroxyphenyllactic aci    -5.15e-03   -7.28e-03   -3.01e-03      20.8    2.90e-06    1.65e-05
## 4-Deoxytetronic acid; 32     -5.82e-03   -8.27e-03   -3.37e-03      21.6    4.00e-06    2.14e-05
## Glyceryl-glycoside; 59       -5.72e-03   -8.20e-03   -3.25e-03      20.8    7.10e-06    3.54e-05
## Pyroglutamic acid; 69        -6.16e-03   -8.92e-03   -3.39e-03      22.9    1.45e-05    6.80e-05
## Serine, 3TMS; 14              2.93e-03    1.61e-03    4.25e-03      26.0    1.64e-05    7.05e-05
## Hydroxyproline; 64           -6.63e-03   -9.62e-03   -3.63e-03      20.6    1.69e-05    7.05e-05
## Benzeneacetic acid; 47       -6.72e-03   -9.92e-03   -3.52e-03      20.4    4.32e-05    1.70e-04
```

```
## Leucine, 2TMS; 19            3.58e-03   1.76e-03   5.40e-03      21.1  1.23e-04   4.62e-04
## Fumaric acid, 2TMS; 9       -2.49e-03  -3.79e-03  -1.19e-03      24.1  1.89e-04   6.76e-04
## Methionine, 2TMS; 16         3.76e-03   1.64e-03   5.87e-03      20.3  5.18e-04   1.77e-03
## 2-hydroxy Isovaleric acid    7.23e-03   3.04e-03   1.14e-02      22.2  7.55e-04   2.46e-03
## 2-Hydroxybutyric acid, 2T    3.79e-03   1.40e-03   6.18e-03      20.7  1.92e-03   6.00e-03
## Ribitol; 70                 -3.49e-03  -5.69e-03  -1.28e-03      20.2  2.02e-03   6.06e-03
## Glycine, 3TMS; 17           -1.79e-03  -2.96e-03  -6.07e-04      21.4  3.06e-03   8.82e-03
## Malic acid, 3TMS; 11        -2.66e-03  -4.43e-03  -8.97e-04      20.0  3.20e-03   8.89e-03
## 3-Indolepropionic acid; 4    5.49e-03   1.44e-03   9.54e-03      19.5  8.00e-03   2.14e-02
## Succinic acid, 2TMS; 7      -1.42e-03  -2.50e-03  -3.32e-04      22.7  1.06e-02   2.74e-02
## Threonine, 3TMS; 12          2.32e-03   5.13e-04   4.13e-03      28.6  1.20e-02   2.99e-02
## Myristoleic acid; 65        -3.96e-03  -7.67e-03  -2.52e-04      19.9  3.64e-02   8.81e-02
## Tyrosine; 75                 4.60e-03   2.63e-04   8.94e-03      23.3  3.77e-02   8.82e-02
## Phenylalanine, 2TMS; 13     -1.20e-03  -2.34e-03  -6.21e-05      24.3  3.88e-02   8.82e-02
## Octanoic acid; 68            1.29e-03   3.89e-05   2.54e-03      24.2  4.33e-02   9.56e-02
## Cholesterol, TMS; 23         1.13e-03   1.75e-05   2.24e-03      21.6  4.65e-02   9.97e-02
## Stearic acid, TMS; 2         6.11e-04  -3.90e-05   1.26e-03      17.4  6.54e-02   1.36e-01
## 11-Eicosenoic acid; 35      -2.55e-03  -5.32e-03   2.26e-04      21.9  7.17e-02   1.45e-01
## 2-Palmitoylglycerol; 39      1.20e-03  -1.74e-04   2.57e-03      25.9  8.67e-02   1.71e-01
## Linoleic acid, TMS; 4        8.15e-04  -2.14e-04   1.84e-03      25.8  1.20e-01   2.31e-01
## Alanine, 2TMS; 25           -1.03e-03  -2.37e-03   3.03e-04      22.8  1.29e-01   2.43e-01
## alpha-ketoglutaric acid,    -2.48e-03  -5.76e-03   8.04e-04      20.2  1.39e-01   2.53e-01
## Oleic acid, TMS; 3          -9.58e-04  -2.36e-03   4.46e-04      17.1  1.81e-01   3.23e-01
## L-5-Oxoproline; 63           7.82e-04  -4.29e-04   1.99e-03      27.5  2.05e-01   3.58e-01
## Hydroxylamine; 62           -1.02e-03  -2.65e-03   6.11e-04      27.7  2.20e-01   3.73e-01
## Ethanolamine; 56             8.83e-04  -5.41e-04   2.31e-03      22.7  2.24e-01   3.73e-01
## Glycerol; 58                -2.62e-03  -6.92e-03   1.69e-03      24.8  2.33e-01   3.79e-01
## Glyceric acid; 30            1.11e-03  -7.55e-04   2.97e-03      20.4  2.43e-01   3.87e-01
## Lactic acid; 29              6.87e-04  -5.36e-04   1.91e-03      25.4  2.70e-01   4.16e-01
## Proline, 2TMS; 21            1.03e-03  -8.06e-04   2.86e-03      20.8  2.72e-01   4.16e-01
## Campesterol; 49             -1.30e-03  -3.70e-03   1.10e-03      21.8  2.88e-01   4.32e-01
## Tridecanoic acid; 74        -8.93e-04  -2.63e-03   8.47e-04      20.0  3.14e-01   4.61e-01
## Arachidonic acid, TMS; 24   -6.31e-04  -1.92e-03   6.57e-04      22.7  3.36e-01   4.85e-01
## Glutamic acid, 3TMS; 8      -8.06e-04  -2.71e-03   1.10e-03      23.0  4.06e-01   5.74e-01
## Heptadecanoic acid; 60      -7.02e-04  -2.41e-03   1.00e-03      21.8  4.19e-01   5.74e-01
## Dodecanoic acid; 54         -9.12e-04  -3.14e-03   1.31e-03      22.7  4.21e-01   5.74e-01
## Glycerol; 57                 6.69e-04  -9.97e-04   2.33e-03      28.7  4.30e-01   5.76e-01
## Docosahexaenoic acid; 53    -9.36e-04  -3.46e-03   1.59e-03      24.2  4.66e-01   5.89e-01
## 1-Dodecanol; 36              5.69e-04  -9.68e-04   2.11e-03      20.2  4.68e-01   5.89e-01
## Heptadecanoic acid; 61      -4.32e-04  -1.61e-03   7.43e-04      23.4  4.70e-01   5.89e-01
## Decanoic acid; 52           -7.50e-04  -2.79e-03   1.29e-03      22.4  4.72e-01   5.89e-01
## Eicosapentaenoic acid; 55    1.06e-03  -1.97e-03   4.09e-03      23.0  4.93e-01   6.02e-01
## Nonadecanoic acid; 66       -6.00e-04  -2.37e-03   1.17e-03      20.0  5.05e-01   6.02e-01
## Aminomalonic acid; 45       -7.01e-04  -2.77e-03   1.37e-03      24.3  5.06e-01   6.02e-01
## Tartronic acid; 73          -8.57e-04  -3.73e-03   2.02e-03      21.3  5.58e-01   6.50e-01
## Nonanoic acid; 67           -4.14e-04  -1.85e-03   1.02e-03      24.4  5.72e-01   6.50e-01
## 4-Hydroxybutanoic acid; 4    2.03e-04  -5.04e-04   9.11e-04      27.3  5.72e-01   6.50e-01
## Arabinopyranose; 51         -6.62e-04  -3.28e-03   1.95e-03      20.1  6.19e-01   6.93e-01
## Pyruvic acid; 31            -6.85e-04  -3.95e-03   2.58e-03      19.5  6.80e-01   7.50e-01
## alpha-Tocopherol; 26        -4.50e-04  -2.87e-03   1.97e-03      18.8  7.15e-01   7.77e-01
## Palmitic acid, TMS; 5        1.25e-04  -7.52e-04   1.00e-03      21.4  7.79e-01   8.35e-01
## Bisphenol A; 48              1.88e-04  -1.69e-03   2.06e-03      21.2  8.44e-01   8.91e-01
## 1,3-Propanediol; 34         -1.43e-04  -1.91e-03   1.62e-03      24.1  8.74e-01   9.01e-01
## 3-Hydroxybutyric acid, 2T    2.20e-04  -2.58e-03   3.02e-03      21.7  8.77e-01   9.01e-01
```

```
## Arachidic acid; 46            -6.18e-05   -1.18e-03    1.05e-03       21.8   9.13e-01    9.26e-01
## 1-Monopalmitin; 37             3.75e-05   -1.53e-03    1.61e-03       29.2   9.63e-01    9.63e-01
```

13.2 Albuminuria Slope

```
## [1] 477   3

##      (Intercept) slope_albuminuria_profil    logUAER
## [1,]           1                -1.218773  0.5849625
## [2,]           1                 1.422851 13.0138461

##
##                                        Overall
##   n                                     477
##   slope_albuminuria_profil (mean (sd)) -0.02 (0.32)
##   logUAER (mean (sd))                   4.67 (2.24)
```

13.2.1 Table

```
##
##
## Table: slope_albuminuria_profil
##
## Name                            Effect       CI.L       CI.R   AveExpr    P.Value   adj.P.Val
## --------------------------  ----------  ---------  ---------  --------  ---------  ----------
## 4-Hydroxybenzeneacetic ac     0.987000    0.62500    1.35000      19.5   1.00e-07    9.70e-06
## Myo inositol 6TMS; 1          0.298000    0.14700    0.44800      19.1   1.16e-04    3.16e-03
## 3,4-Dihydroxybutanoic aci     0.306000    0.15100    0.46200      15.9   1.26e-04    3.16e-03
## Ribonic acid; 72              0.476000    0.21900    0.73300      20.0   2.99e-04    5.61e-03
## 4-Deoxytetronic acid; 32      0.337000    0.13800    0.53700      21.7   9.75e-04    1.46e-02
## 2,4-Dihydroxybutanoic aci     0.219000    0.04960    0.38800      16.6   1.14e-02    1.42e-01
## Threonine, 3TMS; 12          -0.197000   -0.35300   -0.04110      28.6   1.34e-02    1.44e-01
## Ribitol; 71                   0.212000    0.01960    0.40500      23.3   3.09e-02    2.63e-01
## Phenylalanine, 2TMS; 13       0.105000    0.00760    0.20300      24.3   3.47e-02    2.63e-01
## 2-Palmitoylglycerol; 39      -0.124000   -0.24100   -0.00716      25.9   3.76e-02    2.63e-01
## Malic acid, 3TMS; 11          0.154000    0.00774    0.30100      20.0   3.91e-02    2.63e-01
## Creatinine; 50                0.239000    0.00861    0.47000      21.6   4.21e-02    2.63e-01
## 11-Eicosenoic acid; 35        0.212000   -0.01050    0.43500      21.9   6.18e-02    3.57e-01
## 4-Hydroxyphenyllactic aci     0.164000   -0.01440    0.34200      20.8   7.14e-02    3.65e-01
## Glutamic acid, 3TMS; 8        0.146000   -0.01560    0.30800      23.0   7.64e-02    3.65e-01
## L-5-Oxoproline; 63           -0.094900   -0.20000    0.01060      27.5   7.79e-02    3.65e-01
## Citric acid, 4TMS; 6          0.097300   -0.03820    0.23300      20.8   1.59e-01    7.00e-01
## 4-Hydroxybutanoic acid; 4    -0.041900   -0.10100    0.01770      27.3   1.68e-01    7.00e-01
## alpha-ketoglutaric acid,      0.185000   -0.09140    0.46000      20.2   1.89e-01    7.07e-01
## Oleic acid, TMS; 3            0.080600   -0.04000    0.20100      17.1   1.90e-01    7.07e-01
## Benzeneacetic acid; 47        0.177000   -0.10200    0.45600      20.4   2.12e-01    7.07e-01
## Valine, 2TMS; 20             -0.067200   -0.17700    0.04220      25.1   2.28e-01    7.07e-01
## Succinic acid, 2TMS; 7        0.056200   -0.03790    0.15000      22.7   2.41e-01    7.07e-01
## Palmitic acid, TMS; 5         0.041900   -0.03270    0.11600      21.4   2.70e-01    7.07e-01
## Aminomalonic acid; 45        -0.097400   -0.27300    0.07840      24.3   2.77e-01    7.07e-01
## Proline, 2TMS; 21             0.087400   -0.07120    0.24600      20.8   2.79e-01    7.07e-01
## Stearic acid, TMS; 2          0.030700   -0.02550    0.08680      17.4   2.84e-01    7.07e-01
## 4-Deoxytetronic acid; 33      0.157000   -0.13600    0.44900      22.2   2.93e-01    7.07e-01
## Methionine, 2TMS; 16         -0.097900   -0.28100    0.08480      20.3   2.93e-01    7.07e-01
## Bisphenol A; 48              -0.083800   -0.24300    0.07510      21.2   3.01e-01    7.07e-01
## 1-Monopalmitin; 37           -0.070600   -0.20500    0.06390      29.2   3.03e-01    7.07e-01
## Pyroglutamic acid; 69         0.123000   -0.12000    0.36600      22.9   3.20e-01    7.07e-01
## Tartronic acid; 73           -0.122000   -0.36800    0.12400      21.3   3.31e-01    7.07e-01
## Myristoleic acid; 65          0.155000   -0.16100    0.47100      19.9   3.36e-01    7.07e-01
## Campesterol; 49               0.098900   -0.11200    0.31000      21.8   3.57e-01    7.07e-01
## Serine, 3TMS; 14             -0.054500   -0.17100    0.06170      26.0   3.57e-01    7.07e-01
## Arabinopyranose; 51          -0.106000   -0.33100    0.12000      20.1   3.58e-01    7.07e-01
## Heptadecanoic acid; 60       -0.067300   -0.21100    0.07640      21.8   3.58e-01    7.07e-01
## Isoleucine, 2TMS; 18         -0.078800   -0.25500    0.09720      26.0   3.79e-01    7.21e-01
## 3-Hydroxybutyric acid, 2T     0.106000   -0.13300    0.34500      21.7   3.84e-01    7.21e-01
## Ribitol; 70                   0.076600   -0.11200    0.26500      20.2   4.25e-01    7.65e-01
## Glyceric acid; 30            -0.062000   -0.21600    0.09170      20.4   4.28e-01    7.65e-01
## Fumaric acid, 2TMS; 9         0.040600   -0.06970    0.15100      24.1   4.70e-01    8.07e-01
## Glycerol; 57                  0.048800   -0.09530    0.19300      28.7   5.06e-01    8.07e-01
## Pyruvic acid; 31              0.087800   -0.18900    0.36500      19.5   5.33e-01    8.07e-01
## Linoleic acid, TMS; 4         0.029400   -0.06430    0.12300      25.7   5.38e-01    8.07e-01
```

```
## Glycine, 3TMS; 17             -0.032200   -0.13600    0.07200      21.4   5.44e-01    8.07e-01
## Nonanoic acid; 67               0.037300   -0.08560    0.16000      24.4   5.51e-01    8.07e-01
## alpha-Tocopherol; 26           -0.061400   -0.27000    0.14700      18.8   5.62e-01    8.07e-01
## 3-Indolepropionic acid; 4      -0.096000   -0.44000    0.24800      19.6   5.83e-01    8.07e-01
## Tyrosine; 75                   -0.103000   -0.47200    0.26600      23.3   5.84e-01    8.07e-01
## Dodecanoic acid; 54             0.052600   -0.13600    0.24200      22.7   5.85e-01    8.07e-01
## Cholesterol, TMS; 23            0.025800   -0.07030    0.12200      21.7   5.98e-01    8.07e-01
## Ethanolamine; 56                0.031000   -0.09220    0.15400      22.7   6.21e-01    8.07e-01
## Nonadecanoic acid; 66           0.036400   -0.10900    0.18200      20.0   6.24e-01    8.07e-01
## Hydroxylamine; 62               0.034500   -0.10500    0.17400      27.7   6.27e-01    8.07e-01
## 3-Indoleacetic acid; 40         0.059200   -0.18500    0.30400      20.5   6.35e-01    8.07e-01
## Leucine, 2TMS; 19               0.038300   -0.12000    0.19700      21.1   6.35e-01    8.07e-01
## Glycerol; 58                    0.090400   -0.28700    0.46800      24.8   6.38e-01    8.07e-01
## Lactic acid; 29                 0.023600   -0.07700    0.12400      25.4   6.45e-01    8.07e-01
## Eicosapentaenoic acid; 55       0.054600   -0.19700    0.30700      23.0   6.70e-01    8.12e-01
## Arachidonic acid, TMS; 24       0.023400   -0.08490    0.13200      22.7   6.72e-01    8.12e-01
## Arachidic acid; 46             -0.019500   -0.11500    0.07570      21.8   6.88e-01    8.19e-01
## 2-hydroxy Isovaleric acid      -0.070300   -0.42800    0.28700      22.2   7.00e-01    8.20e-01
## Tridecanoic acid; 74            0.019800   -0.12500    0.16400      20.0   7.88e-01    8.87e-01
## Octanoic acid; 68              -0.014200   -0.12100    0.09270      24.2   7.94e-01    8.87e-01
## 1,3-Propanediol; 34            -0.019800   -0.17300    0.13300      24.1   8.00e-01    8.87e-01
## Decanoic acid; 52              -0.022200   -0.19800    0.15400      22.4   8.04e-01    8.87e-01
## Hydroxyproline; 64              0.023700   -0.23300    0.28000      20.6   8.56e-01    9.17e-01
## Glyceryl-glycoside; 59          0.019200   -0.19000    0.22900      20.8   8.57e-01    9.17e-01
## 2-Hydroxybutyric acid, 2T      -0.018000   -0.23000    0.19400      20.7   8.68e-01    9.17e-01
## 1-Dodecanol; 36                -0.009010   -0.14200    0.12400      20.2   8.94e-01    9.24e-01
## Docosahexaenoic acid; 53        0.013900   -0.20100    0.22900      24.2   8.99e-01    9.24e-01
## Heptadecanoic acid; 61         -0.004650   -0.10500    0.09570      23.4   9.27e-01    9.40e-01
## Alanine, 2TMS; 25              -0.000175   -0.11400    0.11400      22.8   9.98e-01    9.98e-01
##
##
## Table: logUAER
##
## Name                             Effect       CI.L        CI.R   AveExpr    P.Value   adj.P.Val
## -------------------------  ----------  ---------  ----------  --------  ---------  ----------
## 3,4-Dihydroxybutanoic aci    0.089900    0.06780    0.112000      15.9   0.00e+00    0.00e+00
## 2,4-Dihydroxybutanoic aci    0.077200    0.05320    0.101000      16.6   0.00e+00    0.00e+00
## Myo inositol 6TMS; 1         0.059100    0.03770    0.080400      19.1   1.00e-07    2.30e-06
## Ribitol; 71                  0.073600    0.04630    0.101000      23.3   2.00e-07    3.60e-06
## 3-Indolepropionic acid; 4   -0.115000   -0.16400   -0.066700      19.6   4.30e-06    6.51e-05
## Glyceric acid; 30           -0.050500   -0.07240   -0.028700      20.4   6.90e-06    8.62e-05
## Ribonic acid; 72             0.083400    0.04690    0.120000      20.0   8.90e-06    9.53e-05
## Glutamic acid, 3TMS; 8       0.047000    0.02400    0.070000      23.0   6.78e-05    6.35e-04
## 4-Hydroxybenzeneacetic ac    0.098000    0.04660    0.149000      19.5   2.00e-04    1.67e-03
## 4-Deoxytetronic acid; 32     0.053400    0.02500    0.081800      21.7   2.45e-04    1.84e-03
## 4-Deoxytetronic acid; 33     0.075800    0.03430    0.117000      22.2   3.67e-04    2.50e-03
## Octanoic acid; 68           -0.026200   -0.04140   -0.011000      24.2   7.53e-04    4.65e-03
## Aminomalonic acid; 45       -0.042800   -0.06780   -0.017900      24.3   8.07e-04    4.65e-03
## Creatinine; 50               0.051100    0.01830    0.083800      21.6   2.30e-03    1.23e-02
## Glyceryl-glycoside; 59       0.041100    0.01130    0.070900      20.8   6.90e-03    3.37e-02
## Arabinopyranose; 51          0.044000    0.01200    0.076000      20.1   7.19e-03    3.37e-02
## Tyrosine; 75                -0.066400   -0.11900   -0.014000      23.3   1.31e-02    5.78e-02
## Docosahexaenoic acid; 53    -0.038400   -0.06890   -0.007840      24.2   1.39e-02    5.78e-02
## Ribitol; 70                  0.032600    0.00586    0.059400      20.2   1.70e-02    6.70e-02
```

```
## Methionine, 2TMS; 16         -0.030000   -0.05590   -0.004030      20.3   2.36e-02     8.87e-02
## Fumaric acid, 2TMS; 9         0.017400    0.00169    0.033000      24.1   3.00e-02     1.04e-01
## Hydroxyproline; 64            0.040200    0.00379    0.076600      20.6   3.05e-02     1.04e-01
## Valine, 2TMS; 20             -0.016500   -0.03200   -0.000955      25.1   3.75e-02     1.22e-01
## 2-hydroxy Isovaleric acid    -0.049200   -0.10000    0.001600      22.2   5.76e-02     1.77e-01
## Threonine, 3TMS; 12          -0.021400   -0.04350    0.000816      28.6   5.90e-02     1.77e-01
## L-5-Oxoproline; 63           -0.013300   -0.02830    0.001680      27.5   8.17e-02     2.27e-01
## 3-Indoleacetic acid; 40       0.030800   -0.00391    0.065500      20.5   8.18e-02     2.27e-01
## Tartronic acid; 73           -0.030500   -0.06550    0.004390      21.3   8.64e-02     2.32e-01
## Linoleic acid, TMS; 4        -0.011300   -0.02460    0.002030      25.7   9.66e-02     2.36e-01
## Glycerol; 58                  0.045300   -0.00828    0.098900      24.8   9.73e-02     2.36e-01
## Heptadecanoic acid; 61       -0.012000   -0.02630    0.002210      23.4   9.75e-02     2.36e-01
## Serine, 3TMS; 14             -0.012200   -0.02870    0.004310      26.0   1.47e-01     3.45e-01
## 3-Hydroxybutyric acid, 2T     0.024300   -0.00965    0.058300      21.7   1.60e-01     3.64e-01
## alpha-Tocopherol; 26         -0.020500   -0.05010    0.009070      18.8   1.74e-01     3.84e-01
## Arachidic acid; 46           -0.009040   -0.02260    0.004480      21.8   1.90e-01     3.97e-01
## Myristoleic acid; 65          0.030000   -0.01490    0.074900      19.9   1.90e-01     3.97e-01
## Oleic acid, TMS; 3            0.011100   -0.00608    0.028200      17.1   2.05e-01     4.13e-01
## Tridecanoic acid; 74         -0.013100   -0.03360    0.007390      20.0   2.09e-01     4.13e-01
## Bisphenol A; 48               0.012900   -0.00967    0.035500      21.2   2.62e-01     4.95e-01
## Campesterol; 49              -0.017000   -0.04700    0.012900      21.8   2.64e-01     4.95e-01
## Heptadecanoic acid; 60       -0.010900   -0.03140    0.009480      21.8   2.93e-01     5.36e-01
## Proline, 2TMS; 21             0.011700   -0.01080    0.034300      20.8   3.08e-01     5.39e-01
## Hydroxylamine; 62             0.010200   -0.00958    0.030100      27.7   3.10e-01     5.39e-01
## Ethanolamine; 56             -0.008890   -0.02640    0.008610      22.7   3.19e-01     5.39e-01
## Malic acid, 3TMS; 11          0.010500   -0.01040    0.031300      20.0   3.23e-01     5.39e-01
## Eicosapentaenoic acid; 55    -0.017100   -0.05280    0.018700      23.0   3.49e-01     5.70e-01
## 4-Hydroxyphenyllactic aci     0.011700   -0.01360    0.037100      20.8   3.63e-01     5.80e-01
## Phenylalanine, 2TMS; 13       0.006080   -0.00778    0.019900      24.3   3.89e-01     6.05e-01
## alpha-ketoglutaric acid,      0.016900   -0.02230    0.056100      20.2   3.97e-01     6.05e-01
## Isoleucine, 2TMS; 18         -0.010600   -0.03560    0.014400      26.0   4.04e-01     6.05e-01
## Arachidonic acid, TMS; 24     0.006430   -0.00895    0.021800      22.7   4.12e-01     6.06e-01
## 1-Dodecanol; 36              -0.007650   -0.02650    0.011200      20.2   4.25e-01     6.13e-01
## Alanine, 2TMS; 25             0.006400   -0.00981    0.022600      22.8   4.38e-01     6.20e-01
## Nonanoic acid; 67            -0.006760   -0.02420    0.010700      24.4   4.47e-01     6.21e-01
## Citric acid, 4TMS; 6          0.005890   -0.01340    0.025100      20.8   5.48e-01     7.47e-01
## 11-Eicosenoic acid; 35        0.008780   -0.02280    0.040400      21.9   5.86e-01     7.82e-01
## Cholesterol, TMS; 23         -0.003690   -0.01740    0.009970      21.7   5.96e-01     7.82e-01
## 4-Hydroxybutanoic acid; 4     0.002190   -0.00628    0.010600      27.3   6.12e-01     7.82e-01
## Lactic acid; 29               0.003660   -0.01060    0.018000      25.4   6.15e-01     7.82e-01
## Nonadecanoic acid; 66         0.004970   -0.01570    0.025700      20.0   6.37e-01     7.89e-01
## Benzeneacetic acid; 47        0.009390   -0.03020    0.049000      20.4   6.41e-01     7.89e-01
## 2-Palmitoylglycerol; 39      -0.003610   -0.02020    0.013000      25.9   6.70e-01     8.11e-01
## 1,3-Propanediol; 34           0.004210   -0.01760    0.026000      24.1   7.04e-01     8.38e-01
## Palmitic acid, TMS; 5         0.001780   -0.00882    0.012400      21.4   7.42e-01     8.40e-01
## Stearic acid, TMS; 2         -0.001300   -0.00927    0.006670      17.4   7.49e-01     8.40e-01
## Decanoic acid; 52            -0.004000   -0.02900    0.021000      22.4   7.53e-01     8.40e-01
## Leucine, 2TMS; 19            -0.003480   -0.02600    0.019000      21.1   7.62e-01     8.40e-01
## Pyroglutamic acid; 69         0.005330   -0.02920    0.039800      22.9   7.62e-01     8.40e-01
## Pyruvic acid; 31             -0.005580   -0.04490    0.033700      19.5   7.81e-01     8.48e-01
## 1-Monopalmitin; 37           -0.001940   -0.02100    0.017200      29.2   8.42e-01     9.02e-01
## Glycerol; 57                  0.000989   -0.01950    0.021500      28.7   9.24e-01     9.72e-01
## Glycine, 3TMS; 17             0.000637   -0.01420    0.015400      21.4   9.33e-01     9.72e-01
## 2-Hydroxybutyric acid, 2T    -0.000791   -0.03090    0.029300      20.7   9.59e-01     9.75e-01
```

```
## Succinic acid, 2TMS; 7        -0.000290   -0.01370    0.013100       22.7   9.66e-01    9.75e-01
## Dodecanoic acid; 54           -0.000422   -0.02730    0.026400       22.7   9.75e-01    9.75e-01
```

14 Supplementary Step 2: Adjusted Associations to Clinical Changes Over Time with Prioritized Metabolites

14.1 Supplementary Step 2A: Adjusted Model for Albuminuria Slope with Prioritized Metabolites

```
## [1] 466  13
```

```
##      (Intercept) slope_albuminuria_profil      egfr    logUAER   Age
## [1,]           1                -1.218773  11.03376  0.5849625 19.39
## [2,]           1                 1.422851 167.62905 13.0138461 85.23
##      Gender Hba1c_baseline CALSBP   bmi Smoking Statin log_Blood_TGA
## [1,]      0            5.2     92 16.98       0      0     -2.643856
## [2,]      1           15.0    190 43.29       1      1      2.718088
##      Total_cholesterol
## [1,]               2.3
## [2,]               9.2
```

```
##
##                                        Overall
##   n                                       466
##   egfr (mean (sd))                      83.34 (26.77)
##   logUAER (mean (sd))                    4.64 (2.22)
##   Age (mean (sd))                       55.03 (12.07)
##   Gender (mean (sd))                     0.53 (0.50)
##   Hba1c_baseline (mean (sd))             8.04 (1.19)
##   CALSBP (mean (sd))                   131.77 (17.27)
##   bmi (mean (sd))                       25.27 (4.05)
##   Smoking (mean (sd))                    0.18 (0.39)
##   Statin (mean (sd))                     0.60 (0.49)
##   log_Blood_TGA (mean (sd))             -0.00 (0.70)
##   Total_cholesterol (mean (sd))          4.70 (0.85)
##   slope_albuminuria_profil (mean (sd))  -0.02 (0.31)
```

14.1.1 Table

```
##
##
## Table: slope_albuminuria_profil
##
## Name                          Effect      CI.L    CI.R   AveExpr    P.Value   adj.P.Val
## --------------------------  -------  --------  ------  --------  ---------  ----------
## 4-Hydroxybenzeneacetic ac    0.6460    0.2670   1.030      19.5   0.000889     0.00445
## 4-Deoxytetronic acid; 32     0.2780    0.0604   0.495      21.7   0.012400     0.03090
## Ribonic acid; 72             0.2000   -0.0605   0.460      20.0   0.132000     0.16600
## 3,4-Dihydroxybutanoic aci    0.1240   -0.0377   0.285      15.9   0.133000     0.16600
## Myo inositol 6TMS; 1         0.0952   -0.0529   0.243      19.1   0.207000     0.20700
##
##
## Table: egfr
##
## Name                            Effect       CI.L       CI.R   AveExpr    P.Value   adj.P.Val
## --------------------------  ---------  ---------  ---------  --------  ---------  ----------
## Myo inositol 6TMS; 1          -0.00877   -0.01070   -0.00685      19.1   0.00e+00    0.00e+00
## Ribonic acid; 72              -0.01230   -0.01570   -0.00889      20.0   0.00e+00    0.00e+00
## 3,4-Dihydroxybutanoic aci     -0.00732   -0.00941   -0.00523      15.9   0.00e+00    0.00e+00
## 4-Hydroxybenzeneacetic ac     -0.01180   -0.01680   -0.00691      19.5   3.10e-06    3.90e-06
## 4-Deoxytetronic acid; 32      -0.00494   -0.00776   -0.00212      21.7   6.38e-04    6.38e-04
##
##
## Table: logUAER
##
## Name                          Effect        CI.L     CI.R   AveExpr    P.Value   adj.P.Val
## --------------------------  -------  ----------  -------  --------  ---------  ----------
## 3,4-Dihydroxybutanoic aci    0.0525    2.69e-02   0.0780      15.9   6.33e-05    0.000316
## 4-Deoxytetronic acid; 32     0.0602    2.58e-02   0.0946      21.7   6.39e-04    0.001600
## Ribonic acid; 72             0.0411   -6.31e-05   0.0824      20.0   5.04e-02    0.073800
## Myo inositol 6TMS; 1         0.0226   -8.69e-04   0.0460      19.1   5.91e-02    0.073800
## 4-Hydroxybenzeneacetic ac    0.0207   -3.95e-02   0.0808      19.5   5.00e-01    0.500000
##
##
## Table: Age
##
## Name                             Effect        CI.L      CI.R   AveExpr   P.Value   adj.P.Val
## --------------------------  ----------  ----------  --------  --------  --------  ----------
## 4-Hydroxybenzeneacetic ac     0.009890   -0.000873   0.02070      19.5    0.0716       0.358
## Myo inositol 6TMS; 1          0.002930   -0.001270   0.00713      19.1    0.1710       0.427
## Ribonic acid; 72              0.003460   -0.003920   0.01080      20.0    0.3580       0.477
## 3,4-Dihydroxybutanoic aci     0.002040   -0.002540   0.00661      15.9    0.3820       0.477
## 4-Deoxytetronic acid; 32     -0.000917   -0.007080   0.00524      21.7    0.7700       0.770
##
##
## Table: Gender
##
## Name                           Effect      CI.L     CI.R   AveExpr   P.Value   adj.P.Val
## --------------------------  --------  --------  -------  --------  --------  ----------
## Myo inositol 6TMS; 1         -0.0642   -0.1560   0.0279      19.1     0.171       0.549
## 4-Hydroxybenzeneacetic ac     0.1480   -0.0884   0.3840      19.5     0.219       0.549
```

```
## Ribonic acid; 72            -0.0712   -0.2330   0.0906      20.0     0.388       0.646
## 3,4-Dihydroxybutanoic aci     0.0297   -0.0705   0.1300      15.9     0.561       0.701
## 4-Deoxytetronic acid; 32     -0.0193   -0.1540   0.1160      21.7     0.779       0.779
##
##
## Table: Hba1c_baseline
##
## Name                           Effect      CI.L      CI.R   AveExpr   P.Value   adj.P.Val
## --------------------------   --------  --------  --------  --------  --------  ----------
## 4-Deoxytetronic acid; 32     -0.0526   -0.1110   0.00602      21.7    0.0785       0.262
## 3,4-Dihydroxybutanoic aci     0.0333   -0.0102   0.07680      15.9    0.1330       0.262
## Myo inositol 6TMS; 1          0.0288   -0.0112   0.06870      19.1    0.1570       0.262
## 4-Hydroxybenzeneacetic ac     0.0464   -0.0561   0.14900      19.5    0.3740       0.406
## Ribonic acid; 72             -0.0297   -0.0999   0.04050      20.0    0.4060       0.406
##
##
## Table: CALSBP
##
## Name                             Effect       CI.L        CI.R   AveExpr   P.Value   adj.P.Val
## --------------------------   ----------  ---------  ----------  --------  --------  ----------
## 4-Deoxytetronic acid; 32     -0.004150   -0.00813   -0.000167      21.7    0.0412       0.206
## 4-Hydroxybenzeneacetic ac     0.004390   -0.00257    0.011300      19.5    0.2150       0.539
## Myo inositol 6TMS; 1          0.000981   -0.00173    0.003690      19.1    0.4780       0.611
## 3,4-Dihydroxybutanoic aci     0.000848   -0.00211    0.003800      15.9    0.5730       0.611
## Ribonic acid; 72              0.001230   -0.00353    0.006000      20.0    0.6110       0.611
##
##
## Table: bmi
##
## Name                             Effect      CI.L      CI.R   AveExpr   P.Value   adj.P.Val
## --------------------------   ----------  --------  --------  --------  --------  ----------
## Ribonic acid; 72             -0.018200   -0.0384   0.00210      20.0    0.0788       0.170
## 3,4-Dihydroxybutanoic aci    -0.011000   -0.0236   0.00152      15.9    0.0849       0.170
## Myo inositol 6TMS; 1         -0.009610   -0.0211   0.00191      19.1    0.1020       0.170
## 4-Hydroxybenzeneacetic ac     0.009210   -0.0203   0.03880      19.5    0.5410       0.676
## 4-Deoxytetronic acid; 32     -0.000748   -0.0177   0.01620      21.7    0.9310       0.931
##
##
## Table: Smoking
##
## Name                           Effect      CI.L       CI.R   AveExpr   P.Value   adj.P.Val
## --------------------------   --------  --------  ---------  --------  --------  ----------
## Ribonic acid; 72             -0.2010   -0.3990   -0.00198      20.0    0.0478       0.239
## 3,4-Dihydroxybutanoic aci     0.0766   -0.0464    0.20000      15.9    0.2220       0.487
## Myo inositol 6TMS; 1         -0.0606   -0.1740    0.05240      19.1    0.2920       0.487
## 4-Hydroxybenzeneacetic ac    -0.1130   -0.4020    0.17700      19.5    0.4460       0.557
## 4-Deoxytetronic acid; 32     -0.0406   -0.2070    0.12500      21.7    0.6300       0.630
##
##
## Table: Statin
##
## Name                           Effect     CI.L     CI.R   AveExpr   P.Value   adj.P.Val
## --------------------------   --------  -------  -------  --------  --------  ----------
## 4-Deoxytetronic acid; 32     -0.1290   -0.276   0.0189      21.7    0.0874       0.437
```

```
## Myo inositol 6TMS; 1         -0.0322   -0.133   0.0684      19.1    0.5300       0.687
## 3,4-Dihydroxybutanoic aci    -0.0279   -0.137   0.0816      15.9    0.6170       0.687
## 4-Hydroxybenzeneacetic ac    -0.0550   -0.313   0.2030      19.5    0.6750       0.687
## Ribonic acid; 72             -0.0362   -0.213   0.1400      20.0    0.6870       0.687
##
##
## Table: log_Blood_TGA
##
## Name                           Effect       CI.L    CI.R   AveExpr   P.Value   adj.P.Val
## --------------------------  --------  ---------  ------  --------  --------  ----------
## Ribonic acid; 72             0.18100    0.05440   0.307      20.0   0.00516      0.0258
## 4-Hydroxybenzeneacetic ac    0.22500    0.04090   0.410      19.5   0.01680      0.0419
## 3,4-Dihydroxybutanoic aci    0.08190    0.00354   0.160      15.9   0.04050      0.0676
## Myo inositol 6TMS; 1         0.05280   -0.01910   0.125      19.1   0.14900      0.1870
## 4-Deoxytetronic acid; 32     0.00809   -0.09750   0.114      21.7   0.88000      0.8800
##
##
## Table: Total_cholesterol
##
## Name                           Effect     CI.L         CI.R   AveExpr     P.Value   adj.P.Val
## --------------------------  --------  -------  ----------  --------  ---------  ----------
## Ribonic acid; 72             -0.1650   -0.261   -0.068200      20.0   0.000865     0.00433
## 4-Hydroxybenzeneacetic ac    -0.1480   -0.289   -0.006830      19.5   0.039900     0.07920
## 3,4-Dihydroxybutanoic aci    -0.0605   -0.120   -0.000671      15.9   0.047500     0.07920
## Myo inositol 6TMS; 1         -0.0479   -0.103    0.007050      19.1   0.087300     0.10900
## 4-Deoxytetronic acid; 32     -0.0422   -0.123    0.038500      21.7   0.305000     0.30500
```

14.2 Supplementary Step 2B: Adjusted Model for eGFR Slope with Prioritized Metabolites

```
## [1] 454  13

##      (Intercept) slope_gfr_profil      egfr    logUAER   Age Gender
## [1,]           1        -29.33939  11.03376  0.5849625 19.39      0
## [2,]           1         16.82789 167.62905 13.0138461 85.23      1
##      Hba1c_baseline CALSBP   bmi Smoking Statin log_Blood_TGA
## [1,]            5.2     92 17.03       0      0     -2.643856
## [2,]           15.0    190 43.29       1      1      2.718088
##      Total_cholesterol
## [1,]               2.3
## [2,]               9.2

##
##                                Overall
##   n                              454
##   egfr (mean (sd))             82.94 (26.90)
##   logUAER (mean (sd))           4.65 (2.23)
##   Age (mean (sd))              54.97 (12.05)
##   Gender (mean (sd))            0.54 (0.50)
##   Hba1c_baseline (mean (sd))    8.07 (1.20)
##   CALSBP (mean (sd))          132.03 (17.21)
##   bmi (mean (sd))              25.35 (4.06)
##   Smoking (mean (sd))           0.19 (0.39)
##   Statin (mean (sd))            0.60 (0.49)
##   log_Blood_TGA (mean (sd))     0.01 (0.70)
##   Total_cholesterol (mean (sd))   4.71 (0.86)
##   slope_gfr_profil (mean (sd))   -1.45 (3.49)
```

14.2.1 Table

```
##
##
## Table: slope_gfr_profil
##
## Name                             Effect       CI.L        CI.R   AveExpr    P.Value   adj.P.Val
## --------------------------  ----------  ----------  ----------  --------  ---------  ----------
## Ribitol; 71                   -3.14e-02   -0.048700   -0.014100      23.3   0.000401      0.0076
## Octanoic acid; 68             -1.89e-02   -0.029400   -0.008420      24.2   0.000434      0.0076
## Succinic acid, 2TMS; 7         1.41e-02    0.004790    0.023400      22.7   0.003050      0.0355
## Ribonic acid; 72              -3.24e-02   -0.055600   -0.009160      20.0   0.006390      0.0559
## L-5-Oxoproline; 63             1.30e-02    0.002680    0.023300      27.5   0.013700      0.0956
## Aminomalonic acid; 45          1.61e-02   -0.000897    0.033100      24.3   0.063300      0.3370
## Myo inositol 6TMS; 1          -1.25e-02   -0.025900    0.000896      19.1   0.067400      0.3370
## 4-Deoxytetronic acid; 32       1.54e-02   -0.004430    0.035200      21.6   0.128000      0.5040
## Methionine, 2TMS; 16           1.35e-02   -0.004190    0.031300      20.2   0.134000      0.5040
## Palmitic acid, TMS; 5         -5.27e-03   -0.012500    0.001990      21.4   0.155000      0.5040
## Glycine, 3TMS; 17              6.98e-03   -0.002730    0.016700      21.4   0.158000      0.5040
## 4-Hydroxybenzeneacetic ac      2.37e-02   -0.011000    0.058400      19.5   0.180000      0.5260
## 3-Indoleacetic acid; 40       -1.34e-02   -0.036000    0.009160      20.5   0.243000      0.6120
## Leucine, 2TMS; 19             -8.86e-03   -0.024100    0.006330      21.1   0.252000      0.6120
## Creatinine; 50                -1.26e-02   -0.034600    0.009450      21.6   0.262000      0.6120
## Isoleucine, 2TMS; 18          -8.57e-03   -0.025300    0.008120      26.0   0.313000      0.6210
## 4-Deoxytetronic acid; 33      -1.39e-02   -0.041100    0.013200      22.2   0.315000      0.6210
## 2-Hydroxybutyric acid, 2T     -9.71e-03   -0.029300    0.009880      20.7   0.330000      0.6210
## 3,4-Dihydroxybutanoic aci     -7.15e-03   -0.021800    0.007470      15.9   0.337000      0.6210
## 4-Hydroxyphenyllactic aci     -6.82e-03   -0.024300    0.010700      20.8   0.444000      0.7780
## Hydroxyproline; 64            -9.21e-03   -0.034100    0.015700      20.6   0.467000      0.7780
## Glyceryl-glycoside; 59        -7.22e-03   -0.027800    0.013400      20.8   0.491000      0.7810
## 2-hydroxy Isovaleric acid     -9.88e-03   -0.045500    0.025700      22.2   0.586000      0.8820
## Eicosapentaenoic acid; 55      6.29e-03   -0.017600    0.030200      23.0   0.605000      0.8820
## Pyroglutamic acid; 69          5.17e-03   -0.018600    0.029000      22.9   0.670000      0.8820
## 2,4-Dihydroxybutanoic aci     -3.30e-03   -0.018600    0.012000      16.6   0.672000      0.8820
## Tyrosine; 75                   7.71e-03   -0.029000    0.044400      23.2   0.680000      0.8820
## Stearic acid, TMS; 2           9.97e-04   -0.004490    0.006480      17.4   0.721000      0.9010
## Valine, 2TMS; 20               1.43e-03   -0.008410    0.011300      25.1   0.775000      0.9190
## Citric acid, 4TMS; 6          -1.54e-03   -0.014200    0.011100      20.8   0.810000      0.9190
## Glycerol; 57                   1.73e-03   -0.012700    0.016200      28.7   0.814000      0.9190
## Serine, 3TMS; 14               7.44e-04   -0.010500    0.012000      26.0   0.897000      0.9810
## Benzeneacetic acid; 47        -7.83e-04   -0.027600    0.026000      20.4   0.954000      0.9990
## Fumaric acid, 2TMS; 9         -1.63e-04   -0.011200    0.010900      24.1   0.977000      0.9990
## Malic acid, 3TMS; 11          -1.35e-05   -0.014600    0.014600      20.0   0.999000      0.9990
##
##
## Table: egfr
##
## Name                             Effect       CI.L        CI.R   AveExpr    P.Value   adj.P.Val
## --------------------------  ----------  ----------  ----------  --------  ---------  ----------
## Myo inositol 6TMS; 1          -0.009410   -0.011300   -0.007500      19.1   0.00e+00    0.00e+00
## Ribitol; 71                   -0.010900   -0.013400   -0.008460      23.3   0.00e+00    0.00e+00
## 2,4-Dihydroxybutanoic aci     -0.008860   -0.011000   -0.006680      16.6   0.00e+00    0.00e+00
## Ribonic acid; 72              -0.013200   -0.016500   -0.009920      20.0   0.00e+00    0.00e+00
## Creatinine; 50                -0.012100   -0.015300   -0.008990      21.6   0.00e+00    0.00e+00
```

```
## 3,4-Dihydroxybutanoic aci    -0.007460   -0.009550   -0.005380      15.9   0.00e+00    0.00e+00
## 4-Deoxytetronic acid; 33     -0.013200   -0.017100   -0.009310      22.2   0.00e+00    0.00e+00
## 4-Hydroxybenzeneacetic ac    -0.013700   -0.018700   -0.008770      19.5   1.00e-07    4.00e-07
## Citric acid, 4TMS; 6         -0.004260   -0.006060   -0.002460      20.8   4.20e-06    1.64e-05
## Isoleucine, 2TMS; 18          0.005470    0.003090    0.007850      26.0   8.20e-06    2.87e-05
## Hydroxyproline; 64           -0.007680   -0.011200   -0.004140      20.6   2.49e-05    7.94e-05
## Pyroglutamic acid; 69        -0.007200   -0.010600   -0.003810      22.9   3.63e-05    1.06e-04
## 3-Indoleacetic acid; 40      -0.006810   -0.010000   -0.003590      20.5   3.93e-05    1.06e-04
## 4-Hydroxyphenyllactic aci    -0.004910   -0.007410   -0.002420      20.8   1.26e-04    3.16e-04
## Valine, 2TMS; 20              0.002690    0.001290    0.004100      25.1   1.83e-04    4.27e-04
## 2-Hydroxybutyric acid, 2T     0.005310    0.002520    0.008100      20.7   2.10e-04    4.59e-04
## Glyceryl-glycoside; 59       -0.005540   -0.008470   -0.002610      20.8   2.31e-04    4.76e-04
## 4-Deoxytetronic acid; 32     -0.005260   -0.008090   -0.002440      21.6   2.81e-04    5.46e-04
## Serine, 3TMS; 14              0.002730    0.001120    0.004340      26.0   9.16e-04    1.69e-03
## Benzeneacetic acid; 47       -0.005920   -0.009740   -0.002090      20.4   2.49e-03    4.27e-03
## Stearic acid, TMS; 2          0.001210    0.000425    0.001990      17.4   2.56e-03    4.27e-03
## Leucine, 2TMS; 19             0.003310    0.001140    0.005470      21.1   2.83e-03    4.51e-03
## Eicosapentaenoic acid; 55     0.004600    0.001200    0.008010      23.0   8.21e-03    1.24e-02
## Fumaric acid, 2TMS; 9        -0.002130   -0.003710   -0.000545      24.1   8.52e-03    1.24e-02
## Methionine, 2TMS; 16          0.003320    0.000791    0.005840      20.2   1.02e-02    1.42e-02
## Glycine, 3TMS; 17            -0.001640   -0.003030   -0.000258      21.4   2.02e-02    2.68e-02
## Malic acid, 3TMS; 11         -0.002460   -0.004530   -0.000378      20.0   2.07e-02    2.68e-02
## Tyrosine; 75                  0.005520    0.000284    0.010700      23.2   3.88e-02    4.85e-02
## Glycerol; 57                  0.001860   -0.000201    0.003920      28.7   7.69e-02    9.28e-02
## Octanoic acid; 68             0.001280   -0.000217    0.002770      24.2   9.37e-02    1.09e-01
## 2-hydroxy Isovaleric acid     0.004230   -0.000844    0.009310      22.2   1.02e-01    1.15e-01
## Succinic acid, 2TMS; 7       -0.000932   -0.002260    0.000392      22.7   1.67e-01    1.83e-01
## Palmitic acid, TMS; 5         0.000674   -0.000361    0.001710      21.4   2.01e-01    2.13e-01
## Aminomalonic acid; 45        -0.001000   -0.003420    0.001420      24.3   4.17e-01    4.29e-01
## L-5-Oxoproline; 63            0.000396   -0.001070    0.001860      27.5   5.96e-01    5.96e-01
##
##
## Table: logUAER
##
## Name                             Effect        CI.L         CI.R    AveExpr    P.Value   adj.P.Val
## -------------------------  ----------  ---------  ----------  --------  --------  ----------
## 3,4-Dihydroxybutanoic aci     0.040500     0.01470    0.066400      15.9    0.00215      0.0397
## 4-Deoxytetronic acid; 32      0.053700     0.01870    0.088700      21.6    0.00272      0.0397
## Octanoic acid; 68            -0.027700    -0.04620   -0.009220      24.2    0.00341      0.0397
## Tyrosine; 75                 -0.064300    -0.12900    0.000586      23.2    0.05210      0.4560
## 2,4-Dihydroxybutanoic aci     0.025000    -0.00210    0.052000      16.6    0.07050      0.4930
## 2-hydroxy Isovaleric acid    -0.050000    -0.11300    0.012900      22.2    0.11900      0.6730
## Methionine, 2TMS; 16         -0.022700    -0.05400    0.008610      20.2    0.15500      0.6730
## 4-Hydroxyphenyllactic aci    -0.022200    -0.05310    0.008770      20.8    0.16000      0.6730
## Valine, 2TMS; 20             -0.012100    -0.02950    0.005310      25.1    0.17300      0.6730
## Eicosapentaenoic acid; 55     0.022300    -0.01990    0.064500      23.0    0.29900      0.9460
## Ribonic acid; 72              0.020900    -0.02010    0.062000      20.0    0.31700      0.9460
## Aminomalonic acid; 45        -0.013300    -0.04330    0.016700      24.3    0.38400      0.9460
## 2-Hydroxybutyric acid, 2T    -0.015100    -0.04970    0.019500      20.7    0.39100      0.9460
## Pyroglutamic acid; 69        -0.017900    -0.06000    0.024100      22.9    0.40200      0.9460
## Myo inositol 6TMS; 1          0.009870    -0.01380    0.033500      19.1    0.41200      0.9460
## Ribitol; 71                   0.011800    -0.01880    0.042400      23.3    0.44900      0.9460
## Palmitic acid, TMS; 5        -0.004440    -0.01730    0.008390      21.4    0.49700      0.9460
## Serine, 3TMS; 14              0.005930    -0.01400    0.025900      26.0    0.55900      0.9460
```

```
## Benzeneacetic acid; 47        0.013000   -0.03440    0.060400       20.4   0.59100        0.9460
## Stearic acid, TMS; 2          0.002370   -0.00732    0.012100       17.4   0.63100        0.9460
## Fumaric acid, 2TMS; 9         0.004380   -0.01520    0.024000       24.1   0.66100        0.9460
## Glyceryl-glycoside; 59        0.008090   -0.02830    0.044400       20.8   0.66200        0.9460
## Leucine, 2TMS; 19            -0.005720   -0.03260    0.021100       21.1   0.67600        0.9460
## Creatinine; 50                0.007820   -0.03110    0.046700       21.6   0.69300        0.9460
## Glycine, 3TMS; 17             0.003220   -0.01390    0.020400       21.4   0.71300        0.9460
## 3-Indoleacetic acid; 40       0.007070   -0.03280    0.047000       20.5   0.72800        0.9460
## Hydroxyproline; 64           -0.007470   -0.05140    0.036500       20.6   0.73900        0.9460
## Malic acid, 3TMS; 11          0.003990   -0.02180    0.029700       20.0   0.76100        0.9460
## L-5-Oxoproline; 63            0.002540   -0.01570    0.020700       27.5   0.78400        0.9460
## Isoleucine, 2TMS; 18         -0.002540   -0.03200    0.027000       26.0   0.86600        0.9590
## 4-Deoxytetronic acid; 33      0.003160   -0.04480    0.051100       22.2   0.89700        0.9590
## Citric acid, 4TMS; 6          0.000942   -0.02130    0.023200       20.8   0.93400        0.9590
## Glycerol; 57                  0.000845   -0.02470    0.026400       28.7   0.94800        0.9590
## Succinic acid, 2TMS; 7        0.000447   -0.01600    0.016900       22.7   0.95700        0.9590
## 4-Hydroxybenzeneacetic ac     0.001600   -0.05970    0.062900       19.5   0.95900        0.9590
##
##
## Table: Age
##
## Name                             Effect         CI.L       CI.R   AveExpr     P.Value   adj.P.Val
## -------------------------  ----------  ----------  --------  --------  ---------  ----------
## Eicosapentaenoic acid; 55     2.01e-02     0.012600    0.02760      23.0    3.00e-07    8.90e-06
## Aminomalonic acid; 45         6.40e-03     0.001040    0.01180      24.3    1.94e-02    2.58e-01
## 2,4-Dihydroxybutanoic aci     5.61e-03     0.000774    0.01040      16.6    2.31e-02    2.58e-01
## Octanoic acid; 68             3.67e-03     0.000367    0.00698      24.2    2.95e-02    2.58e-01
## 3-Indoleacetic acid; 40       7.51e-03     0.000379    0.01460      20.5    3.91e-02    2.73e-01
## 4-Hydroxybenzeneacetic ac     1.02e-02    -0.000749    0.02120      19.5    6.78e-02    3.15e-01
## L-5-Oxoproline; 63            3.01e-03    -0.000244    0.00626      27.5    6.97e-02    3.15e-01
## Fumaric acid, 2TMS; 9         3.03e-03    -0.000467    0.00654      24.1    8.93e-02    3.15e-01
## Palmitic acid, TMS; 5         1.95e-03    -0.000346    0.00424      21.4    9.59e-02    3.15e-01
## Glycine, 3TMS; 17             2.52e-03    -0.000547    0.00559      21.4    1.07e-01    3.15e-01
## Myo inositol 6TMS; 1          3.42e-03    -0.000807    0.00764      19.1    1.13e-01    3.15e-01
## Glycerol; 57                  3.60e-03    -0.000960    0.00816      28.7    1.21e-01    3.15e-01
## Stearic acid, TMS; 2          1.36e-03    -0.000369    0.00309      17.4    1.23e-01    3.15e-01
## Ribitol; 71                   4.27e-03    -0.001200    0.00974      23.3    1.26e-01    3.15e-01
## Hydroxyproline; 64           -5.53e-03    -0.013400    0.00232      20.6    1.67e-01    3.69e-01
## Malic acid, 3TMS; 11          3.23e-03    -0.001370    0.00783      20.0    1.69e-01    3.69e-01
## 3,4-Dihydroxybutanoic aci     2.94e-03    -0.001680    0.00755      15.9    2.12e-01    4.36e-01
## 4-Deoxytetronic acid; 33     -5.10e-03    -0.013700    0.00348      22.2    2.43e-01    4.56e-01
## Ribonic acid; 72              4.32e-03    -0.003020    0.01170      20.0    2.48e-01    4.56e-01
## Methionine, 2TMS; 16          2.82e-03    -0.002780    0.00842      20.2    3.23e-01    5.61e-01
## Tyrosine; 75                  5.68e-03    -0.005910    0.01730      23.2    3.36e-01    5.61e-01
## 2-hydroxy Isovaleric acid    -4.93e-03    -0.016200    0.00631      22.2    3.89e-01    6.04e-01
## Benzeneacetic acid; 47        3.66e-03    -0.004810    0.01210      20.4    3.97e-01    6.04e-01
## 4-Hydroxyphenyllactic aci     2.22e-03    -0.003310    0.00775      20.8    4.31e-01    6.11e-01
## Pyroglutamic acid; 69         2.74e-03    -0.004780    0.01030      22.9    4.75e-01    6.11e-01
## Succinic acid, 2TMS; 7        1.06e-03    -0.001870    0.00399      22.7    4.77e-01    6.11e-01
## Serine, 3TMS; 14              1.28e-03    -0.002280    0.00485      26.0    4.79e-01    6.11e-01
## Isoleucine, 2TMS; 18          1.85e-03    -0.003430    0.00712      26.0    4.92e-01    6.11e-01
## 2-Hydroxybutyric acid, 2T     2.09e-03    -0.004090    0.00828      20.7    5.06e-01    6.11e-01
## Citric acid, 4TMS; 6          1.24e-03    -0.002740    0.00522      20.8    5.40e-01    6.30e-01
## Creatinine; 50                1.43e-03    -0.005530    0.00838      21.6    6.87e-01    7.76e-01
```

```
## Valine, 2TMS; 20              -3.07e-04   -0.003410   0.00280      25.1   8.46e-01    9.25e-01
## Glyceryl-glycoside; 59        -3.67e-04   -0.006860   0.00613      20.8   9.12e-01    9.67e-01
## 4-Deoxytetronic acid; 32       1.51e-04   -0.006110   0.00641      21.6   9.62e-01    9.90e-01
## Leucine, 2TMS; 19             -1.78e-05   -0.004820   0.00478      21.1   9.94e-01    9.94e-01
##
##
## Table: Gender
##
## Name                            Effect      CI.L      CI.R   AveExpr    P.Value   adj.P.Val
## --------------------------  ---------  --------  --------  --------  ---------  ----------
## 4-Deoxytetronic acid; 33      0.44500    0.2560    0.6340      22.2   4.90e-06    0.000173
## Citric acid, 4TMS; 6         -0.18800   -0.2760   -0.1000      20.8   3.10e-05    0.000543
## Glycine, 3TMS; 17            -0.13100   -0.1990   -0.0635      21.4   1.59e-04    0.001490
## Methionine, 2TMS; 16          0.23800    0.1150    0.3620      20.2   1.70e-04    0.001490
## Stearic acid, TMS; 2         -0.06890   -0.1070   -0.0307      17.4   4.39e-04    0.002940
## Valine, 2TMS; 20              0.12200    0.0537    0.1910      25.1   5.04e-04    0.002940
## Hydroxyproline; 64            0.30200    0.1280    0.4750      20.6   6.79e-04    0.003400
## Palmitic acid, TMS; 5        -0.07900   -0.1300   -0.0284      21.4   2.28e-03    0.009980
## Succinic acid, 2TMS; 7       -0.08890   -0.1540   -0.0242      22.7   7.22e-03    0.028100
## Leucine, 2TMS; 19             0.14300    0.0371    0.2490      21.1   8.24e-03    0.028900
## 2-hydroxy Isovaleric acid     0.31800    0.0701    0.5660      22.2   1.21e-02    0.038400
## Aminomalonic acid; 45        -0.14900   -0.2680   -0.0311      24.3   1.34e-02    0.039100
## Isoleucine, 2TMS; 18          0.13800    0.0213    0.2540      26.0   2.05e-02    0.055200
## Benzeneacetic acid; 47       -0.17500   -0.3610    0.0122      20.4   6.69e-02    0.167000
## Tyrosine; 75                 -0.19700   -0.4530    0.0589      23.2   1.31e-01    0.304000
## Creatinine; 50                0.11500   -0.0384    0.2680      21.6   1.41e-01    0.304000
## Myo inositol 6TMS; 1         -0.06880   -0.1620    0.0244      19.1   1.47e-01    0.304000
## Glycerol; 57                 -0.06730   -0.1680    0.0333      28.7   1.89e-01    0.355000
## Pyroglutamic acid; 69         0.11000   -0.0558    0.2760      22.9   1.93e-01    0.355000
## 2-Hydroxybutyric acid, 2T    -0.07820   -0.2150    0.0583      20.7   2.61e-01    0.456000
## Ribitol; 71                   0.05880   -0.0618    0.1800      23.3   3.38e-01    0.522000
## 2,4-Dihydroxybutanoic aci     0.05150   -0.0552    0.1580      16.6   3.43e-01    0.522000
## 4-Hydroxybenzeneacetic ac     0.11600   -0.1250    0.3580      19.5   3.45e-01    0.522000
## Ribonic acid; 72             -0.07590   -0.2380    0.0861      20.0   3.58e-01    0.522000
## Octanoic acid; 68             0.02730   -0.0457    0.1000      24.2   4.63e-01    0.625000
## 3-Indoleacetic acid; 40       0.05860   -0.0988    0.2160      20.5   4.65e-01    0.625000
## L-5-Oxoproline; 63            0.02470   -0.0471    0.0965      27.5   4.99e-01    0.626000
## Serine, 3TMS; 14             -0.02600   -0.1050    0.0527      26.0   5.17e-01    0.626000
## Malic acid, 3TMS; 11         -0.03340   -0.1350    0.0682      20.0   5.19e-01    0.626000
## Eicosapentaenoic acid; 55    -0.04780   -0.2140    0.1190      23.0   5.73e-01    0.668000
## Glyceryl-glycoside; 59        0.03860   -0.1050    0.1820      20.8   5.97e-01    0.674000
## Fumaric acid, 2TMS; 9        -0.01880   -0.0960    0.0585      24.1   6.33e-01    0.693000
## 4-Deoxytetronic acid; 32     -0.02140   -0.1600    0.1170      21.6   7.61e-01    0.790000
## 4-Hydroxyphenyllactic aci     0.01840   -0.1040    0.1400      20.8   7.67e-01    0.790000
## 3,4-Dihydroxybutanoic aci     0.00438   -0.0974    0.1060      15.9   9.33e-01    0.933000
##
##
## Table: Hba1c_baseline
##
## Name                             Effect        CI.L        CI.R   AveExpr   P.Value   adj.P.Val
## --------------------------  ----------  ----------  ---------  --------  --------  ----------
## Valine, 2TMS; 20              0.049500    0.019700    0.07940      25.1   0.00118      0.0377
## Eicosapentaenoic acid; 55    -0.114000   -0.186000   -0.04130      23.0   0.00215      0.0377
## Citric acid, 4TMS; 6          0.055300    0.017100    0.09350      20.8   0.00466      0.0544
```

```
## 4-Deoxytetronic acid; 33     -0.088700   -0.171000   -0.00641       22.2   0.03470       0.2480
## Glyceryl-glycoside; 59        0.066900    0.004590    0.12900       20.8   0.03540       0.2480
## 2,4-Dihydroxybutanoic aci     0.046700    0.000272    0.09310       16.6   0.04870       0.2840
## Malic acid, 3TMS; 11          0.039100   -0.005090    0.08320       20.0   0.08280       0.3480
## 3,4-Dihydroxybutanoic aci     0.038900   -0.005330    0.08320       15.9   0.08460       0.3480
## Glycine, 3TMS; 17             0.025500   -0.003950    0.05490       21.4   0.08960       0.3480
## Myo inositol 6TMS; 1          0.033000   -0.007540    0.07350       19.1   0.11000       0.3570
## 4-Hydroxybenzeneacetic ac     0.083100   -0.022000    0.18800       19.5   0.12100       0.3570
## Leucine, 2TMS; 19             0.035300   -0.010800    0.08130       21.1   0.13300       0.3570
## 2-Hydroxybutyric acid, 2T     0.045000   -0.014300    0.10400       20.7   0.13700       0.3570
## Tyrosine; 75                  0.083100   -0.028100    0.19400       23.2   0.14300       0.3570
## Octanoic acid; 68            -0.022200   -0.053900    0.00956       24.2   0.17000       0.3970
## Serine, 3TMS; 14              0.022800   -0.011400    0.05700       26.0   0.19100       0.4180
## 4-Deoxytetronic acid; 32     -0.037500   -0.097500    0.02260       21.6   0.22100       0.4480
## Isoleucine, 2TMS; 18          0.030900   -0.019700    0.08150       26.0   0.23000       0.4480
## Benzeneacetic acid; 47        0.046400   -0.034800    0.12800       20.4   0.26200       0.4830
## Ribonic acid; 72             -0.037300   -0.108000    0.03310       20.0   0.29800       0.5210
## Glycerol; 57                  0.020800   -0.023000    0.06450       28.7   0.35200       0.5860
## Stearic acid, TMS; 2         -0.007470   -0.024100    0.00914       17.4   0.37700       0.6000
## Ribitol; 71                   0.018500   -0.034000    0.07090       23.3   0.49000       0.7320
## 4-Hydroxyphenyllactic aci    -0.018200   -0.071200    0.03490       20.8   0.50200       0.7320
## Fumaric acid, 2TMS; 9         0.010900   -0.022700    0.04450       24.1   0.52300       0.7320
## Pyroglutamic acid; 69         0.020200   -0.052000    0.09230       22.9   0.58300       0.7760
## Creatinine; 50               -0.017900   -0.084600    0.04880       21.6   0.59900       0.7760
## L-5-Oxoproline; 63            0.007810   -0.023400    0.03900       27.5   0.62300       0.7790
## 3-Indoleacetic acid; 40      -0.013900   -0.082400    0.05450       20.5   0.68900       0.8320
## Succinic acid, 2TMS; 7        0.005200   -0.022900    0.03330       22.7   0.71700       0.8360
## 2-hydroxy Isovaleric acid    -0.012500   -0.120000    0.09540       22.2   0.82000       0.9260
## Aminomalonic acid; 45         0.003370   -0.048100    0.05480       24.3   0.89800       0.9330
## Methionine, 2TMS; 16          0.003450   -0.050300    0.05720       20.2   0.90000       0.9330
## Hydroxyproline; 64           -0.004530   -0.079900    0.07080       20.6   0.90600       0.9330
## Palmitic acid, TMS; 5        -0.000805   -0.022800    0.02120       21.4   0.94300       0.9430
##
##
## Table: CALSBP
##
## Name                            Effect        CI.L         CI.R   AveExpr   P.Value   adj.P.Val
## --------------------------  ----------  ----------  ----------  --------  --------  ----------
## Serine, 3TMS; 14             -2.65e-03   -4.99e-03   -0.000303      26.0    0.0270       0.326
## 3-Indoleacetic acid; 40      -4.95e-03   -9.64e-03   -0.000255      20.5    0.0388       0.326
## Creatinine; 50               -4.81e-03   -9.38e-03   -0.000233      21.6    0.0395       0.326
## Palmitic acid, TMS; 5         1.57e-03    6.09e-05    0.003080      21.4    0.0415       0.326
## 2-Hydroxybutyric acid, 2T     4.13e-03    6.33e-05    0.008200      20.7    0.0466       0.326
## 4-Hydroxybenzeneacetic ac     6.53e-03   -6.70e-04    0.013700      19.5    0.0754       0.438
## 4-Deoxytetronic acid; 32     -3.59e-03   -7.70e-03    0.000532      21.6    0.0877       0.438
## Valine, 2TMS; 20             -1.49e-03   -3.54e-03    0.000550      25.1    0.1520       0.638
## Eicosapentaenoic acid; 55     3.52e-03   -1.44e-03    0.008480      23.0    0.1640       0.638
## Stearic acid, TMS; 2          6.90e-04   -4.49e-04    0.001830      17.4    0.2340       0.816
## Aminomalonic acid; 45        -1.95e-03   -5.48e-03    0.001570      24.3    0.2770       0.816
## L-5-Oxoproline; 63            1.14e-03   -9.99e-04    0.003280      27.5    0.2950       0.816
## Glycerol; 57                  1.40e-03   -1.60e-03    0.004400      28.7    0.3590       0.816
## Isoleucine, 2TMS; 18         -1.51e-03   -4.98e-03    0.001960      26.0    0.3940       0.816
## Fumaric acid, 2TMS; 9         9.48e-04   -1.35e-03    0.003250      24.1    0.4190       0.816
## Citric acid, 4TMS; 6          1.05e-03   -1.57e-03    0.003670      20.8    0.4320       0.816
```

```
## Glycine, 3TMS; 17            7.81e-04  -1.24e-03  0.002800     21.4  0.4470      0.816
## 2,4-Dihydroxybutanoic aci     1.21e-03  -1.97e-03  0.004390     16.6  0.4550      0.816
## Ribitol; 71                  -1.32e-03  -4.91e-03  0.002280     23.3  0.4720      0.816
## Methionine, 2TMS; 16         -1.28e-03  -4.96e-03  0.002400     20.2  0.4960      0.816
## Myo inositol 6TMS; 1          9.49e-04  -1.83e-03  0.003730     19.1  0.5020      0.816
## 3,4-Dihydroxybutanoic aci     8.83e-04  -2.15e-03  0.003920     15.9  0.5680      0.816
## Malic acid, 3TMS; 11         -8.68e-04  -3.90e-03  0.002160     20.0  0.5730      0.816
## 4-Hydroxyphenyllactic aci     1.03e-03  -2.61e-03  0.004670     20.8  0.5780      0.816
## 4-Deoxytetronic acid; 33     -1.58e-03  -7.22e-03  0.004060     22.2  0.5830      0.816
## Tyrosine; 75                 -1.74e-03  -9.37e-03  0.005880     23.2  0.6530      0.867
## Benzeneacetic acid; 47       -1.21e-03  -6.78e-03  0.004360     20.4  0.6690      0.867
## Ribonic acid; 72              9.57e-04  -3.87e-03  0.005780     20.0  0.6970      0.871
## Pyroglutamic acid; 69         8.84e-04  -4.06e-03  0.005830     22.9  0.7260      0.872
## Succinic acid, 2TMS; 7        3.16e-04  -1.61e-03  0.002250     22.7  0.7470      0.872
## Hydroxyproline; 64           -5.93e-04  -5.76e-03  0.004570     20.6  0.8210      0.927
## Glyceryl-glycoside; 59       -2.59e-04  -4.53e-03  0.004010     20.8  0.9050      0.969
## Octanoic acid; 68             1.20e-04  -2.06e-03  0.002300     24.2  0.9130      0.969
## Leucine, 2TMS; 19            -7.59e-05  -3.23e-03  0.003080     21.1  0.9620      0.974
## 2-hydroxy Isovaleric acid    -1.24e-04  -7.52e-03  0.007270     22.2  0.9740      0.974
##
##
## Table: bmi
##
## Name                            Effect       CI.L       CI.R  AveExpr   P.Value  adj.P.Val
## --------------------------  ----------  ----------  ---------  --------  ---------  ----------
## 2-Hydroxybutyric acid, 2T     0.036900    0.019800    0.05400     20.7  2.66e-05   0.000931
## Pyroglutamic acid; 69        -0.026100   -0.046800   -0.00534     22.9  1.39e-02   0.242000
## 2,4-Dihydroxybutanoic aci    -0.014500   -0.027800   -0.00112     16.6  3.37e-02   0.389000
## Valine, 2TMS; 20              0.008790    0.000217    0.01740     25.1  4.45e-02   0.389000
## 4-Hydroxyphenyllactic aci     0.014300   -0.001000    0.02950     20.8  6.70e-02   0.453000
## Leucine, 2TMS; 19             0.011400   -0.001840    0.02470     21.1  9.13e-02   0.453000
## Benzeneacetic acid; 47       -0.019700   -0.043000    0.00371     20.4  9.89e-02   0.453000
## Citric acid, 4TMS; 6         -0.009120   -0.020100    0.00187     20.8  1.04e-01   0.453000
## Isoleucine, 2TMS; 18          0.010900   -0.003700    0.02540     26.0  1.43e-01   0.557000
## Ribitol; 71                  -0.010400   -0.025500    0.00470     23.3  1.76e-01   0.569000
## Stearic acid, TMS; 2         -0.003280   -0.008060    0.00151     17.4  1.79e-01   0.569000
## Octanoic acid; 68            -0.005440   -0.014600    0.00369     24.2  2.43e-01   0.680000
## Glyceryl-glycoside; 59       -0.010500   -0.028400    0.00748     20.8  2.53e-01   0.680000
## 3,4-Dihydroxybutanoic aci    -0.006530   -0.019300    0.00621     15.9  3.14e-01   0.710000
## Glycine, 3TMS; 17            -0.004230   -0.012700    0.00424     21.4  3.27e-01   0.710000
## Hydroxyproline; 64            0.009860   -0.011800    0.03150     20.6  3.72e-01   0.710000
## 4-Hydroxybenzeneacetic ac     0.013700   -0.016500    0.04390     19.5  3.73e-01   0.710000
## 3-Indoleacetic acid; 40       0.008800   -0.010900    0.02850     20.5  3.80e-01   0.710000
## Succinic acid, 2TMS; 7        0.003460   -0.004640    0.01160     22.7  4.02e-01   0.710000
## Tyrosine; 75                  0.013200   -0.018800    0.04520     23.2  4.17e-01   0.710000
## Ribonic acid; 72             -0.008210   -0.028500    0.01200     20.0  4.26e-01   0.710000
## Aminomalonic acid; 45        -0.004480   -0.019300    0.01030     24.3  5.53e-01   0.830000
## Myo inositol 6TMS; 1         -0.003510   -0.015200    0.00815     19.1  5.55e-01   0.830000
## L-5-Oxoproline; 63           -0.002600   -0.011600    0.00637     27.5  5.69e-01   0.830000
## Creatinine; 50               -0.005100   -0.024300    0.01410     21.6  6.02e-01   0.836000
## Glycerol; 57                 -0.003170   -0.015800    0.00942     28.7  6.21e-01   0.836000
## 2-hydroxy Isovaleric acid     0.006730   -0.024300    0.03780     22.2  6.70e-01   0.869000
## Fumaric acid, 2TMS; 9        -0.001930   -0.011600    0.00774     24.1  6.95e-01   0.869000
## Malic acid, 3TMS; 11         -0.002110   -0.014800    0.01060     20.0  7.44e-01   0.889000
```

```
## 4-Deoxytetronic acid; 33     -0.003270   -0.026900    0.02040      22.2   7.86e-01    0.889000
## 4-Deoxytetronic acid; 32      0.002370   -0.014900    0.01970      21.6   7.87e-01    0.889000
## Palmitic acid, TMS; 5         0.000336   -0.005990    0.00667      21.4   9.17e-01    0.979000
## Eicosapentaenoic acid; 55    -0.000551   -0.021400    0.02030      23.0   9.59e-01    0.979000
## Serine, 3TMS; 14             -0.000199   -0.010000    0.00964      26.0   9.68e-01    0.979000
## Methionine, 2TMS; 16          0.000212   -0.015200    0.01570      20.2   9.79e-01    0.979000
##
##
## Table: Smoking
##
## Name                            Effect       CI.L        CI.R   AveExpr    P.Value   adj.P.Val
## --------------------------  ----------  --------  ----------  --------  --------  ----------
## Benzeneacetic acid; 47       -0.343000   -0.5710   -0.115000      20.4   0.00324      0.0599
## Valine, 2TMS; 20             -0.125000   -0.2090   -0.041600      25.1   0.00342      0.0599
## Citric acid, 4TMS; 6         -0.149000   -0.2560   -0.041800      20.8   0.00656      0.0766
## Malic acid, 3TMS; 11         -0.165000   -0.2890   -0.041400      20.0   0.00905      0.0792
## Octanoic acid; 68            -0.115000   -0.2040   -0.025600      24.2   0.01180      0.0824
## Ribonic acid; 72             -0.229000   -0.4270   -0.031500      20.0   0.02310      0.1350
## 3-Indoleacetic acid; 40      -0.193000   -0.3850   -0.000527      20.5   0.04940      0.2470
## Leucine, 2TMS; 19            -0.106000   -0.2350    0.023700      21.1   0.10900      0.4780
## 3,4-Dihydroxybutanoic aci     0.095100   -0.0292    0.219000      15.9   0.13300      0.5180
## Succinic acid, 2TMS; 7       -0.045800   -0.1250    0.033100      22.7   0.25500      0.8850
## 4-Hydroxyphenyllactic aci    -0.078800   -0.2280    0.070100      20.8   0.29900      0.8850
## Myo inositol 6TMS; 1         -0.059600   -0.1730    0.054100      19.1   0.30300      0.8850
## Aminomalonic acid; 45         0.066400   -0.0780    0.211000      24.3   0.36600      0.8880
## Isoleucine, 2TMS; 18         -0.064100   -0.2060    0.077900      26.0   0.37500      0.8880
## 4-Deoxytetronic acid; 33     -0.097600   -0.3280    0.133000      22.2   0.40700      0.8880
## Glycine, 3TMS; 17             0.029500   -0.0530    0.112000      21.4   0.48200      0.8880
## Eicosapentaenoic acid; 55    -0.068200   -0.2710    0.135000      23.0   0.50900      0.8880
## Ribitol; 71                  -0.048500   -0.1960    0.098700      23.3   0.51800      0.8880
## Tyrosine; 75                  0.097600   -0.2140    0.410000      23.2   0.53900      0.8880
## Pyroglutamic acid; 69        -0.056500   -0.2590    0.146000      22.9   0.58300      0.8880
## 4-Deoxytetronic acid; 32     -0.045900   -0.2140    0.123000      21.6   0.59300      0.8880
## Palmitic acid, TMS; 5         0.016000   -0.0457    0.077700      21.4   0.61100      0.8880
## Fumaric acid, 2TMS; 9        -0.022300   -0.1170    0.072000      24.1   0.64200      0.8880
## Stearic acid, TMS; 2         -0.009530   -0.0562    0.037100      17.4   0.68800      0.8880
## Methionine, 2TMS; 16         -0.030800   -0.1810    0.120000      20.2   0.68800      0.8880
## 4-Hydroxybenzeneacetic ac    -0.057600   -0.3520    0.237000      19.5   0.70100      0.8880
## 2-hydroxy Isovaleric acid    -0.053800   -0.3560    0.249000      22.2   0.72700      0.8880
## L-5-Oxoproline; 63            0.013300   -0.0742    0.101000      27.5   0.76500      0.8880
## Glyceryl-glycoside; 59       -0.026600   -0.2010    0.148000      20.8   0.76500      0.8880
## Serine, 3TMS; 14             -0.013900   -0.1100    0.082000      26.0   0.77600      0.8880
## 2,4-Dihydroxybutanoic aci     0.015100   -0.1150    0.145000      16.6   0.82000      0.8880
## 2-Hydroxybutyric acid, 2T    -0.018800   -0.1850    0.148000      20.7   0.82500      0.8880
## Hydroxyproline; 64           -0.022100   -0.2330    0.189000      20.6   0.83700      0.8880
## Creatinine; 50               -0.008600   -0.1960    0.179000      21.6   0.92800      0.9550
## Glycerol; 57                  0.000794   -0.1220    0.124000      28.7   0.99000      0.9900
##
##
## Table: Statin
##
## Name                           Effect       CI.L       CI.R   AveExpr     P.Value   adj.P.Val
## --------------------------  ---------  --------  ---------  --------  ---------  ----------
## L-5-Oxoproline; 63            -0.14100   -0.2200   -0.06230      27.5   0.000476      0.0166
```

```
## Aminomalonic acid; 45        -0.19600   -0.3260   -0.06570      24.3   0.003240      0.0464
## Glycine, 3TMS; 17            -0.10900   -0.1840   -0.03510      21.4   0.003990      0.0464
## Citric acid, 4TMS; 6         -0.13800   -0.2340   -0.04110      20.8   0.005300      0.0464
## Malic acid, 3TMS; 11         -0.11400   -0.2250   -0.00222      20.0   0.045600      0.3190
## 4-Deoxytetronic acid; 32     -0.14300   -0.2950    0.00839      21.6   0.064000      0.3430
## Fumaric acid, 2TMS; 9        -0.07880   -0.1640    0.00601      24.1   0.068500      0.3430
## Palmitic acid, TMS; 5        -0.03830   -0.0939    0.01720      21.4   0.176000      0.7690
## Serine, 3TMS; 14             -0.05260   -0.1390    0.03370      26.0   0.232000      0.9020
## Methionine, 2TMS; 16          0.07600   -0.0596    0.21200      20.2   0.272000      0.9290
## Myo inositol 6TMS; 1         -0.05230   -0.1550    0.05010      19.1   0.316000      0.9290
## Ribonic acid; 72             -0.08530   -0.2630    0.09240      20.0   0.346000      0.9290
## 3,4-Dihydroxybutanoic aci    -0.04160   -0.1530    0.07020      15.9   0.465000      0.9290
## Tyrosine; 75                  0.10200   -0.1790    0.38200      23.2   0.477000      0.9290
## 4-Deoxytetronic acid; 33      0.06630   -0.1410    0.27400      22.2   0.531000      0.9290
## Glyceryl-glycoside; 59       -0.04910   -0.2060    0.10800      20.8   0.540000      0.9290
## 3-Indoleacetic acid; 40      -0.05350   -0.2260    0.11900      20.5   0.543000      0.9290
## Benzeneacetic acid; 47       -0.06140   -0.2670    0.14400      20.4   0.557000      0.9290
## Stearic acid, TMS; 2         -0.01050   -0.0525    0.03140      17.4   0.622000      0.9290
## Valine, 2TMS; 20             -0.01860   -0.0939    0.05660      25.1   0.627000      0.9290
## Creatinine; 50               -0.03830   -0.2070    0.13000      21.6   0.655000      0.9290
## Octanoic acid; 68            -0.01770   -0.0979    0.06240      24.2   0.664000      0.9290
## Ribitol; 71                   0.02750   -0.1050    0.16000      23.3   0.684000      0.9290
## Eicosapentaenoic acid; 55     0.03440   -0.1480    0.21700      23.0   0.712000      0.9290
## Isoleucine, 2TMS; 18         -0.02230   -0.1500    0.10500      26.0   0.731000      0.9290
## 2-hydroxy Isovaleric acid    -0.04720   -0.3200    0.22500      22.2   0.733000      0.9290
## Hydroxyproline; 64            0.03050   -0.1600    0.22100      20.6   0.753000      0.9290
## 4-Hydroxybenzeneacetic ac    -0.04200   -0.3070    0.22300      19.5   0.756000      0.9290
## 2,4-Dihydroxybutanoic aci     0.01750   -0.0997    0.13500      16.6   0.769000      0.9290
## 2-Hydroxybutyric acid, 2T    -0.01630   -0.1660    0.13400      20.7   0.831000      0.9320
## Succinic acid, 2TMS; 7       -0.00763   -0.0787    0.06340      22.7   0.833000      0.9320
## Leucine, 2TMS; 19            -0.01100   -0.1270    0.10500      21.1   0.852000      0.9320
## Glycerol; 57                  0.00724   -0.1030    0.11800      28.7   0.898000      0.9460
## Pyroglutamic acid; 69         0.00891   -0.1730    0.19100      22.9   0.923000      0.9460
## 4-Hydroxyphenyllactic aci     0.00462   -0.1290    0.13900      20.8   0.946000      0.9460
##
##
## Table: log_Blood_TGA
##
## Name                            Effect        CI.L       CI.R   AveExpr    P.Value    adj.P.Val
## -------------------------  ---------  ----------  ---------  --------  ---------  ----------
## Palmitic acid, TMS; 5          0.07730    0.038000    0.11700      21.4   0.000126     0.00371
## Stearic acid, TMS; 2           0.05640    0.026700    0.08610      17.4   0.000212     0.00371
## Octanoic acid; 68              0.10200    0.045400    0.15900      24.2   0.000440     0.00513
## 2-Hydroxybutyric acid, 2T      0.17500    0.069300    0.28100      20.7   0.001240     0.01080
## Glyceryl-glycoside; 59         0.17800    0.066900    0.28900      20.8   0.001760     0.01230
## Ribonic acid; 72               0.18100    0.055000    0.30600      20.0   0.004940     0.02480
## Isoleucine, 2TMS; 18           0.13000    0.039500    0.22000      26.0   0.004950     0.02480
## 4-Hydroxybenzeneacetic ac      0.23100    0.043100    0.41800      19.5   0.016100     0.07030
## Aminomalonic acid; 45         -0.11100   -0.203000   -0.01900      24.3   0.018200     0.07070
## Valine, 2TMS; 20               0.06140    0.008190    0.11500      25.1   0.023800     0.08340
## 3,4-Dihydroxybutanoic aci      0.07990    0.000896    0.15900      15.9   0.047500     0.13900
## Fumaric acid, 2TMS; 9          0.06070    0.000673    0.12100      24.1   0.047500     0.13900
## Leucine, 2TMS; 19              0.07710   -0.005090    0.15900      21.1   0.065900     0.17700
## Glycine, 3TMS; 17             -0.04620   -0.098700    0.00631      21.4   0.084500     0.21100
```

```
## Eicosapentaenoic acid; 55    -0.10600   -0.235000    0.02290       23.0   0.107000      0.24300
## Ribitol; 71                   0.07610   -0.017600    0.17000       23.3   0.111000      0.24300
## Malic acid, 3TMS; 11          0.06030   -0.018600    0.13900       20.0   0.134000      0.27500
## Myo inositol 6TMS; 1          0.04550   -0.026800    0.11800       19.1   0.217000      0.42200
## 4-Deoxytetronic acid; 33      0.08830   -0.058600    0.23500       22.2   0.238000      0.43900
## Pyroglutamic acid; 69        -0.07350   -0.202000    0.05520       22.9   0.262000      0.45900
## 3-Indoleacetic acid; 40       0.06770   -0.054500    0.19000       20.5   0.277000      0.46100
## Serine, 3TMS; 14             -0.03110   -0.092200    0.02990       26.0   0.317000      0.50400
## Hydroxyproline; 64            0.06400   -0.070500    0.19800       20.6   0.350000      0.53300
## Glycerol; 57                  0.03310   -0.045000    0.11100       28.7   0.405000      0.59100
## Succinic acid, 2TMS; 7        0.01600   -0.034300    0.06620       22.7   0.532000      0.72200
## 2-hydroxy Isovaleric acid     0.06060   -0.132000    0.25300       22.2   0.537000      0.72200
## Tyrosine; 75                  0.05470   -0.144000    0.25300       23.2   0.589000      0.73700
## 2,4-Dihydroxybutanoic aci     0.02280   -0.060100    0.10600       16.6   0.590000      0.73700
## Benzeneacetic acid; 47       -0.03240   -0.177000    0.11300       20.4   0.661000      0.79800
## Citric acid, 4TMS; 6          0.01360   -0.054600    0.08180       20.8   0.696000      0.81200
## L-5-Oxoproline; 63            0.00946   -0.046300    0.06520       27.5   0.739000      0.81500
## Creatinine; 50                0.01970   -0.099400    0.13900       21.6   0.745000      0.81500
## 4-Hydroxyphenyllactic aci     0.01370   -0.081100    0.10800       20.8   0.777000      0.82400
## 4-Deoxytetronic acid; 32      0.01310   -0.094200    0.12000       21.6   0.811000      0.83500
## Methionine, 2TMS; 16         -0.00224   -0.098100    0.09360       20.2   0.963000      0.96300
##
##
## Table: Total_cholesterol
##
## Name                             Effect        CI.L        CI.R   AveExpr    P.Value   adj.P.Val
## --------------------------  ----------  ----------  ----------  --------  ---------  ----------
## Ribonic acid; 72              -0.164000    -0.26000    -0.06740      20.0   0.000904      0.0182
## Isoleucine, 2TMS; 18          -0.111000    -0.18000    -0.04140      26.0   0.001780      0.0182
## Glycine, 3TMS; 17             -0.063300    -0.10300    -0.02310      21.4   0.002110      0.0182
## L-5-Oxoproline; 63            -0.066100    -0.10900    -0.02350      27.5   0.002450      0.0182
## Benzeneacetic acid; 47        -0.171000    -0.28200    -0.06000      20.4   0.002600      0.0182
## Eicosapentaenoic acid; 55      0.140000     0.04130     0.23900      23.0   0.005550      0.0324
## Glyceryl-glycoside; 59        -0.117000    -0.20200    -0.03210      20.8   0.007090      0.0354
## 2,4-Dihydroxybutanoic aci     -0.083900    -0.14700    -0.02050      16.6   0.009640      0.0393
## Tyrosine; 75                  -0.200000    -0.35200    -0.04780      23.2   0.010100      0.0393
## Malic acid, 3TMS; 11          -0.072400    -0.13300    -0.01210      20.0   0.018800      0.0659
## Ribitol; 71                   -0.081600    -0.15300    -0.00985      23.3   0.025900      0.0824
## Citric acid, 4TMS; 6          -0.058000    -0.11000    -0.00584      20.8   0.029400      0.0858
## 3,4-Dihydroxybutanoic aci     -0.065700    -0.12600    -0.00517      15.9   0.033500      0.0901
## Serine, 3TMS; 14              -0.049700    -0.09640    -0.00298      26.0   0.037100      0.0928
## Methionine, 2TMS; 16          -0.076800    -0.15000    -0.00341      20.2   0.040300      0.0941
## 4-Hydroxybenzeneacetic ac     -0.138000    -0.28100     0.00575      19.5   0.059900      0.1310
## Leucine, 2TMS; 19             -0.058800    -0.12200     0.00415      21.1   0.067100      0.1380
## Myo inositol 6TMS; 1          -0.045900    -0.10100     0.00952      19.1   0.104000      0.2030
## 3-Indoleacetic acid; 40       -0.071000    -0.16500     0.02250      20.5   0.137000      0.2510
## Palmitic acid, TMS; 5          0.020700    -0.00939     0.05070      21.4   0.177000      0.3100
## Aminomalonic acid; 45         -0.046500    -0.11700     0.02390      24.3   0.195000      0.3170
## Valine, 2TMS; 20              -0.026700    -0.06740     0.01410      25.1   0.199000      0.3170
## Pyroglutamic acid; 69         -0.052100    -0.15100     0.04640      22.9   0.299000      0.4550
## Hydroxyproline; 64            -0.035500    -0.13800     0.06750      20.6   0.499000      0.7270
## 4-Deoxytetronic acid; 32      -0.026800    -0.10900     0.05530      21.6   0.521000      0.7300
## 4-Deoxytetronic acid; 33      -0.029400    -0.14200     0.08310      22.2   0.608000      0.8180
## Glycerol; 57                  -0.013100    -0.07290     0.04670      28.7   0.668000      0.8510
```

```
## Succinic acid, 2TMS; 7        0.008060   -0.03040    0.04650       22.7   0.681000       0.8510
## 4-Hydroxyphenyllactic aci    -0.012600   -0.08510    0.05990       20.8   0.733000       0.8520
## Octanoic acid; 68             0.007280   -0.03610    0.05070       24.2   0.742000       0.8520
## 2-hydroxy Isovaleric acid     0.023400   -0.12400    0.17100       22.2   0.755000       0.8520
## Creatinine; 50                0.010700   -0.08040    0.10200       21.6   0.817000       0.8670
## 2-Hydroxybutyric acid, 2T     0.009510   -0.07160    0.09060       20.7   0.818000       0.8670
## Stearic acid, TMS; 2         -0.001310   -0.02400    0.02140       17.4   0.910000       0.9370
## Fumaric acid, 2TMS; 9         0.000359   -0.04560    0.04630       24.1   0.988000       0.9880
```

15 Clinical Characteristics

```
##                                Stratified by Group
##                                 Control\nT1D
##   n                                268
##   Group (%)
##      Control\nT1D                  268 (100.0)
##      Micro-\nalbumin-\nuria\nT1D      0 (  0.0)
##      Macro-\nalbumin-\nuria\nT1D      0 (  0.0)
##   Gender = Female (%)              136 ( 50.7)
##   Age (mean (sd))                53.82 (13.13)
##   Duration_DM (mean (sd))        27.97 (17.12)
##   bmi (mean (sd))                24.72 (3.57)
##   CALSBP (mean (sd))            129.49 (16.29)
##   Cal_DIA (mean (sd))            75.12 (9.24)
##   Hba1c_baseline (mean (sd))      7.77 (1.04)
##   Total_cholesterol (mean (sd))   4.75 (0.78)
##   Blood_LDL (mean (sd))           2.50 (0.69)
##   Blood_HDL (mean (sd))           1.77 (0.57)
##   Blood_TGA (mean (sd))           1.04 (0.50)
##   egfr (mean (sd))               93.08 (21.32)
##   logUAER (mean (sd))            10.96 (15.27)
##                                Stratified by Group
##                                 Micro-\nalbumin-\nuria\nT1D
##   n                                145
##   Group (%)
##      Control\nT1D                    0 (  0.0)
##      Micro-\nalbumin-\nuria\nT1D    145 (100.0)
##      Macro-\nalbumin-\nuria\nT1D      0 (  0.0)
##   Gender = Female (%)               57 ( 39.3)
##   Age (mean (sd))                58.62 (11.67)
##   Duration_DM (mean (sd))        36.32 (15.41)
##   bmi (mean (sd))                25.65 (4.17)
##   CALSBP (mean (sd))            133.22 (18.21)
##   Cal_DIA (mean (sd))            72.48 (9.05)
##   Hba1c_baseline (mean (sd))      8.13 (1.17)
##   Total_cholesterol (mean (sd))   4.66 (0.85)
##   Blood_LDL (mean (sd))           2.44 (0.76)
##   Blood_HDL (mean (sd))           1.72 (0.52)
##   Blood_TGA (mean (sd))           1.14 (0.73)
##   egfr (mean (sd))               83.99 (27.04)
##   logUAER (mean (sd))            78.64 (374.82)
##                                Stratified by Group
##                                 Macro-\nalbumin-\nuria\nT1D p      test
##   n                                173
##   Group (%)                                                <0.001
##      Control\nT1D                    0 (  0.0)
##      Micro-\nalbumin-\nuria\nT1D      0 (  0.0)
##      Macro-\nalbumin-\nuria\nT1D    173 (100.0)
##   Gender = Female (%)               77 ( 44.5)              0.074
##   Age (mean (sd))                55.06 (10.22)              0.001
##   Duration_DM (mean (sd))        39.16 (10.72)             <0.001
##   bmi (mean (sd))                25.57 (4.57)               0.029
##   CALSBP (mean (sd))            134.58 (18.23)              0.007
```

```
##   Cal_DIA (mean (sd))              73.95 (9.70)                 0.022
##   Hba1c_baseline (mean (sd))        8.36 (1.23)                <0.001
##   Total_cholesterol (mean (sd))     4.61 (1.03)                 0.245
##   Blood_LDL (mean (sd))             2.44 (0.86)                 0.629
##   Blood_HDL (mean (sd))             1.57 (0.50)                <0.001
##   Blood_TGA (mean (sd))             1.31 (0.83)                <0.001
##   egfr (mean (sd))                 63.32 (29.12)               <0.001
##   logUAER (mean (sd))             439.56 (877.52)              <0.001
```

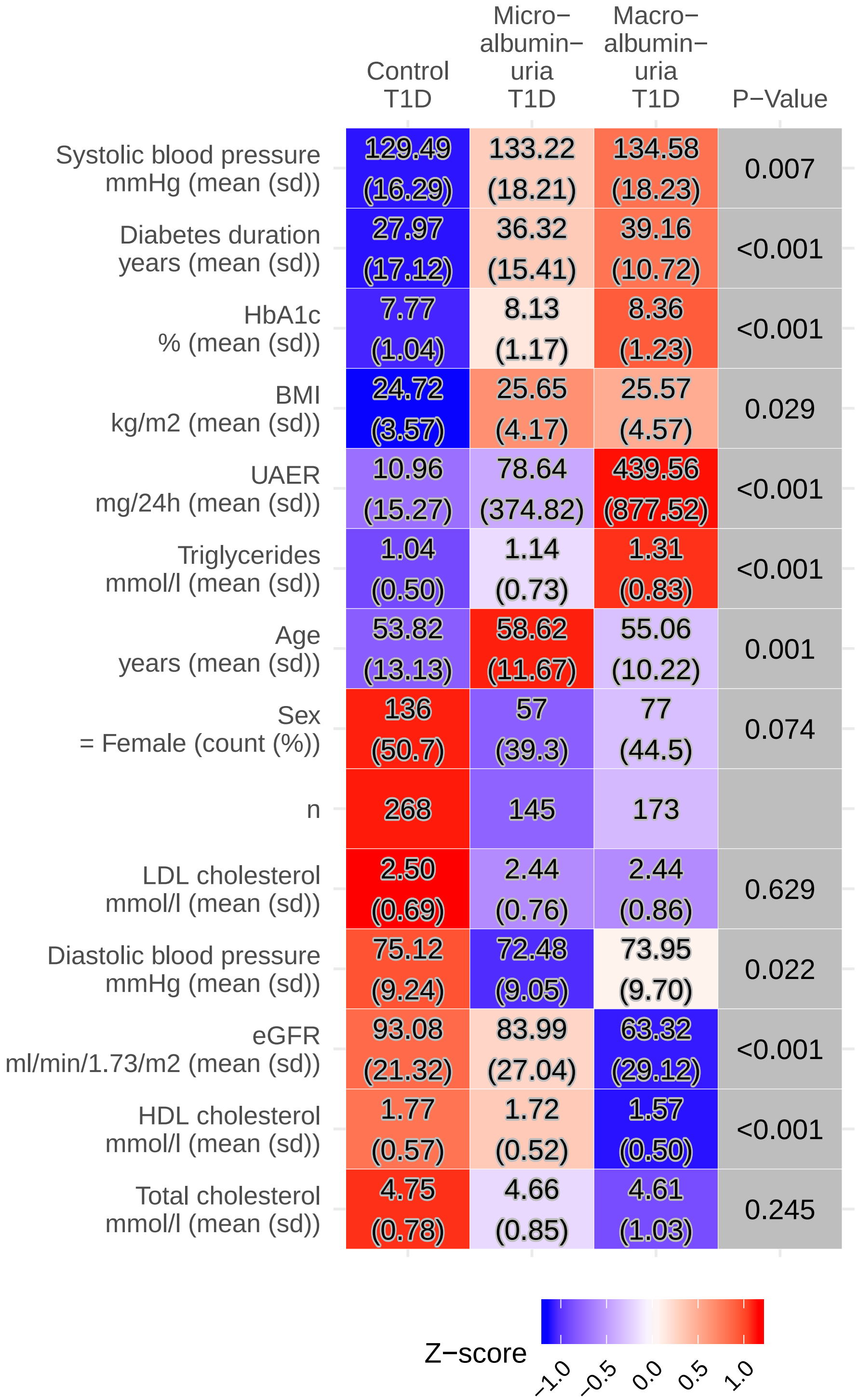

	Control T1D	Micro−albumin−uria T1D	Macro−albumin−uria T1D	P−Value
Systolic blood pressure mmHg (mean (sd))	129.49 (16.29)	133.22 (18.21)	134.58 (18.23)	0.007
Diabetes duration years (mean (sd))	27.97 (17.12)	36.32 (15.41)	39.16 (10.72)	<0.001
HbA1c % (mean (sd))	7.77 (1.04)	8.13 (1.17)	8.36 (1.23)	<0.001
BMI kg/m2 (mean (sd))	24.72 (3.57)	25.65 (4.17)	25.57 (4.57)	0.029
UAER mg/24h (mean (sd))	10.96 (15.27)	78.64 (374.82)	439.56 (877.52)	<0.001
Triglycerides mmol/l (mean (sd))	1.04 (0.50)	1.14 (0.73)	1.31 (0.83)	<0.001
Age years (mean (sd))	53.82 (13.13)	58.62 (11.67)	55.06 (10.22)	0.001
Sex = Female (count (%))	136 (50.7)	57 (39.3)	77 (44.5)	0.074
n	268	145	173	
LDL cholesterol mmol/l (mean (sd))	2.50 (0.69)	2.44 (0.76)	2.44 (0.86)	0.629
Diastolic blood pressure mmHg (mean (sd))	75.12 (9.24)	72.48 (9.05)	73.95 (9.70)	0.022
eGFR ml/min/1.73/m2 (mean (sd))	93.08 (21.32)	83.99 (27.04)	63.32 (29.12)	<0.001
HDL cholesterol mmol/l (mean (sd))	1.77 (0.57)	1.72 (0.52)	1.57 (0.50)	<0.001
Total cholesterol mmol/l (mean (sd))	4.75 (0.78)	4.66 (0.85)	4.61 (1.03)	0.245

Z−score: −1.0, −0.5, 0.0, 0.5, 1.0

	Control T1D	Micro−albumin−uria T1D	Macro−albumin−uria T1D	P−Value
Systolic blood pressure mmHg (mean (sd))	129.49 (16.29)	133.22 (18.21)	134.58 (18.23)	0.007
Diabetes duration years (mean (sd))	27.97 (17.12)	36.32 (15.41)	39.16 (10.72)	<0.001
HbA1c % (mean (sd))	7.77 (1.04)	8.13 (1.17)	8.36 (1.23)	<0.001
BMI kg/m2 (mean (sd))	24.72 (3.57)	25.65 (4.17)	25.57 (4.57)	0.029
UAER mg/24h (mean (sd))	10.96 (15.27)	78.64 (374.82)	439.56 (877.52)	<0.001
Triglycerides mmol/l (mean (sd))	1.04 (0.50)	1.14 (0.73)	1.31 (0.83)	<0.001
Age years (mean (sd))	53.82 (13.13)	58.62 (11.67)	55.06 (10.22)	0.001
Sex = Female (count (%))	136 (50.7)	57 (39.3)	77 (44.5)	0.074
n	268	145	173	
LDL cholesterol mmol/l (mean (sd))	2.50 (0.69)	2.44 (0.76)	2.44 (0.86)	0.629
Diastolic blood pressure mmHg (mean (sd))	75.12 (9.24)	72.48 (9.05)	73.95 (9.70)	0.022
eGFR ml/min/1.73/m2 (mean (sd))	93.08 (21.32)	83.99 (27.04)	63.32 (29.12)	<0.001
HDL cholesterol mmol/l (mean (sd))	1.77 (0.57)	1.72 (0.52)	1.57 (0.50)	<0.001
Total cholesterol mmol/l (mean (sd))	4.75 (0.78)	4.66 (0.85)	4.61 (1.03)	0.245

16 Quality Control Table of Metabolite Measurements

Table 21: Coefficient of variation (CV; %; also called relative standard deviation) of the metabolomic features in quality control samples and study samples. Values are shown for pooled samples from the study (Pool.Study), NIST samples (NIST), long-term quality control samples from the clinic of Steno Diabetes Center Copenahgen (Pool.Clinic), and study samples (Sample). Additionally, the ratio of CVs in the study samples and in the pooled samples from the study are shown (Ratio.Sample.v.Pool). The rows of the table are sorted by this ratio.

Name	Pool.Study	Pool.Clinic	Sample	Ratio.Sample.v.Pool
Malic acid, 3TMS; 11	28.96	32.25	40.60	1.40
Decanoic acid; 52	22.00	29.91	29.35	1.33
Ribitol; 70	39.63	66.64	52.36	1.32
Ribonic acid; 72	36.67	45.69	47.86	1.31
3-Hydroxybutyric acid, 2TMS; 1	39.78	64.72	50.97	1.28
Myristoleic acid; 65	38.03	38.22	47.32	1.24
3-Indolepropionic acid; 41	38.24	54.96	47.17	1.23
Oleic acid, TMS; 3	32.33	47.03	39.34	1.22
Dodecanoic acid; 54	35.46	32.56	41.45	1.17
2,4-Dihydroxybutanoic acid; 28	27.75	42.30	31.45	1.13
Tyrosine; 75	45.57	37.25	50.99	1.12
Glyceric acid; 30	30.30	36.98	33.35	1.10
Ribitol; 71	32.00	47.36	35.16	1.10
Phenylalanine, 2TMS; 13	22.77	20.39	24.84	1.09
Arachidic acid; 46	45.21	48.00	49.24	1.09
4-Hydroxybutanoic acid; 43	13.56	19.80	14.66	1.08
Lactic acid; 29	30.82	26.07	32.98	1.07
Heptadecanoic acid; 61	39.65	41.28	42.39	1.07
3,4-Dihydroxybutanoic acid; 27	29.53	39.08	31.28	1.06
Linoleic acid, TMS; 4	42.53	48.61	45.05	1.06
Ethanolamine; 56	32.80	27.23	34.70	1.06
Myo inositol 6TMS; 1	30.03	39.42	31.69	1.06
Proline, 2TMS; 21	29.44	28.76	30.95	1.05
Tridecanoic acid; 74	43.58	38.63	45.74	1.05
Serine, 3TMS; 14	27.16	30.14	28.46	1.05
4-Hydroxybenzeneacetic acid; 4	39.20	42.57	41.03	1.05
Benzeneacetic acid; 47	28.04	34.79	29.34	1.05
4-Deoxytetronic acid; 33	49.88	62.16	51.68	1.04
Hydroxyproline; 64	43.02	43.60	44.53	1.04
1-Dodecanol; 36	24.86	29.92	25.65	1.03
alpha-Tocopherol; 26	29.23	35.47	30.15	1.03
Citric acid, 4TMS; 6	33.00	38.81	34.03	1.03
Eicosapentaenoic acid; 55	32.98	38.83	34.00	1.03
Arabinopyranose; 51	34.33	41.06	35.36	1.03
Valine, 2TMS; 20	21.19	23.33	21.81	1.03
Docosahexaenoic acid; 53	51.29	61.38	52.77	1.03
Alanine, 2TMS; 25	22.98	20.67	23.60	1.03
4-Hydroxyphenyllactic acid; 44	34.59	36.58	35.46	1.03
11-Eicosenoic acid; 35	53.75	50.37	54.95	1.02
Pyruvic acid; 31	65.52	37.23	66.79	1.02

Name	Pool.Study	Pool.Clinic	Sample	Ratio.Sample.v.Pool
Heptadecanoic acid; 60	42.80	37.90	43.46	1.02
2-Hydroxybutyric acid, 2TMS; 2	26.26	28.93	26.60	1.01
Leucine, 2TMS; 19	25.89	24.87	26.21	1.01
Pyroglutamic acid; 69	38.82	35.46	39.02	1.01
2-hydroxy Isovaleric acid; 38	51.59	50.11	51.82	1.00
Bisphenol A; 48	62.92	35.20	63.17	1.00
Glutamic acid, 3TMS; 8	33.30	22.52	33.43	1.00
Glycerol; 57	34.27	35.81	34.36	1.00
Isoleucine, 2TMS; 18	26.43	24.45	26.36	1.00
Glyceryl-glycoside; 59	38.14	38.27	38.03	1.00
1,3-Propanediol; 34	51.97	44.00	51.54	0.99
Succinic acid, 2TMS; 7	28.99	19.25	28.75	0.99
3-Indoleacetic acid; 40	30.86	27.43	30.51	0.99
Nonadecanoic acid; 66	27.08	25.61	26.73	0.99
Creatinine; 50	46.88	37.89	46.24	0.99
Octanoic acid; 68	33.61	29.39	33.09	0.98
Cholesterol, TMS; 23	27.49	14.13	27.04	0.98
Fumaric acid, 2TMS; 9	28.25	25.46	27.76	0.98
1-Monopalmitin; 37	92.74	82.34	90.86	0.98
Glycine, 3TMS; 17	22.61	16.21	22.13	0.98
Hydroxylamine; 62	62.60	51.01	60.97	0.97
Glycerol; 58	88.34	73.84	85.51	0.97
Campesterol; 49	37.47	25.72	36.10	0.96
Palmitic acid, TMS; 5	53.36	37.83	51.35	0.96
Threonine, 3TMS; 12	28.76	21.98	27.67	0.96
Nonanoic acid; 67	36.14	26.96	34.74	0.96
4-Deoxytetronic acid; 32	45.21	30.54	43.44	0.96
2-Palmitoylglycerol; 39	78.66	59.69	75.53	0.96
Aminomalonic acid; 45	41.56	30.64	39.77	0.96
Arachidonic acid, TMS; 24	41.66	28.99	39.66	0.95
alpha-ketoglutaric acid, TMS M	82.02	43.75	77.82	0.95
L-5-Oxoproline; 63	21.84	12.00	20.71	0.95
Tartronic acid; 73	59.73	34.79	56.21	0.94
Stearic acid, TMS; 2	48.54	28.65	45.50	0.94
Methionine, 2TMS; 16	40.06	20.68	37.32	0.93

17 Appendix

```r
utils::sessionInfo()
```

```
## R version 3.4.2 (2017-09-28)
## Platform: x86_64-w64-mingw32/x64 (64-bit)
## Running under: Windows 10 x64 (build 17763)
##
## Matrix products: default
##
## locale:
## [1] LC_COLLATE=Danish_Denmark.1252  LC_CTYPE=Danish_Denmark.1252
## [3] LC_MONETARY=Danish_Denmark.1252 LC_NUMERIC=C
## [5] LC_TIME=Danish_Denmark.1252
##
## attached base packages:
## [1] grid      stats     graphics  grDevices utils     datasets  methods
## [8] base
##
## other attached packages:
## [1] scales_1.0.0  plyr_1.8.4    ggplot2_3.2.0
##
## loaded via a namespace (and not attached):
##   [1] minqa_1.2.4          colorspace_1.3-2     hyphenatr_0.3.0
##   [4] rjson_0.2.20         class_7.3-14         ggbiplot_0.55
##   [7] htmlTable_1.9        corpcor_1.6.9        base64enc_0.1-3
##  [10] ggpubr_0.1.6         lavaan_0.6-3         splines_3.4.2
##  [13] mnormt_1.5-5         knitr_1.23           glasso_1.10
##  [16] Formula_1.2-2        nloptr_1.0.4         broom_0.4.3
##  [19] km.ci_0.5-2          cluster_2.0.6        png_0.1-7
##  [22] readr_1.1.1          compiler_3.4.2       backports_1.1.1
##  [25] assertthat_0.2.0     Matrix_1.2-11        lazyeval_0.2.0
##  [28] survey_3.33          limma_3.32.10        acepack_1.4.1
##  [31] htmltools_0.3.6      tools_3.4.2          bindrcpp_0.2
##  [34] igraph_1.2.1         coda_0.19-1          gtable_0.2.0
##  [37] glue_1.2.0           reshape2_1.4.2       dplyr_0.7.4
##  [40] Rcpp_0.12.13         statnet.common_4.0.0 gdata_2.18.0
##  [43] nlme_3.1-131         psych_1.7.8          xfun_0.7
##  [46] stringr_1.2.0        network_1.13.0.1     lme4_1.1-15
##  [49] ggm_2.3              gtools_3.5.0         tableone_0.9.2
##  [52] MASS_7.3-47          zoo_1.8-0            BDgraph_2.53
##  [55] hms_0.4.0            parallel_3.4.2       huge_1.2.7
##  [58] RColorBrewer_1.1-2   yaml_2.2.0           pbapply_1.3-4
##  [61] gridExtra_2.3        KMsurv_0.1-5         labelled_1.0.1
##  [64] rpart_4.1-11         latticeExtra_0.6-28  stringi_1.1.5
##  [67] highr_0.6            corrplot_0.84        e1071_1.6-8
##  [70] sem_3.1-9            checkmate_1.8.5      caTools_1.17.1
##  [73] boot_1.3-20          d3Network_0.5.2.1    rlang_0.3.4
##  [76] pkgconfig_2.0.1      bitops_1.0-6         arm_1.10-1
##  [79] evaluate_0.14        lattice_0.20-35      purrr_0.2.4
##  [82] bindr_0.1            htmlwidgets_0.9      labeling_0.3
##  [85] cmprsk_2.2-7         shadowtext_0.0.6     cowplot_0.9.2
##  [88] tidyselect_0.2.3     magrittr_1.5         R6_2.2.2
```

```
##  [91] gplots_3.0.1         Hmisc_4.0-3          sna_2.4
##  [94] whisker_0.3-2        haven_1.1.0          foreign_0.8-69
##  [97] withr_2.0.0          survival_2.41-3      abind_1.4-5
## [100] nnet_7.3-12          tibble_1.3.4         survMisc_0.5.4
## [103] fdrtool_1.2.15       KernSmooth_2.23-15   ellipse_0.4.1
## [106] rmarkdown_1.13       jpeg_0.1-8           qgraph_1.5
## [109] data.table_1.10.4-3  pbivnorm_0.6.0       forcats_0.2.0
## [112] matrixcalc_1.0-3     digest_0.6.12        xtable_1.8-2
## [115] mi_1.0               tidyr_0.8.0          stats4_3.4.2
## [118] munsell_0.5.0        survminer_0.4.2
```
